# Structure-guided optimisation of *N*-hydroxythiazole-derived inhibitors of factor inhibiting hypoxia-inducible factor-α†

**DOI:** 10.1039/d3sc04253g

**Published:** 2023-10-27

**Authors:** Thomas P. Corner, Ryan Z. R. Teo, Yue Wu, Eidarus Salah, Yu Nakashima, Giorgia Fiorini, Anthony Tumber, Amelia Brasnett, James P. Holt-Martyn, William D. Figg, Xiaojin Zhang, Lennart Brewitz, Christopher J. Schofield

**Affiliations:** a Chemistry Research Laboratory, Department of Chemistry and the Ineos Oxford Institute for Antimicrobial Research, University of Oxford 12 Mansfield Road OX1 3TA Oxford United Kingdom lennart.brewitz@chem.ox.ac.uk christopher.schofield@chem.ox.ac.uk; b State Key Laboratory of Natural Medicines, Jiangsu Key Laboratory of Drug Design and Optimization and Department of Chemistry, China Pharmaceutical University Nanjing 211198 China zxj@cpu.edu.cn; c Institute of Natural Medicine, University of Toyama 2630-Sugitani 930-0194 Toyama Japan

## Abstract

The human 2-oxoglutarate (2OG)- and Fe(ii)-dependent oxygenases factor inhibiting hypoxia-inducible factor-α (FIH) and HIF-α prolyl residue hydroxylases 1–3 (PHD1–3) regulate the response to hypoxia in humans *via* catalysing hydroxylation of the α-subunits of the hypoxia-inducible factors (HIFs). Small-molecule PHD inhibitors are used for anaemia treatment; by contrast, few selective inhibitors of FIH have been reported, despite their potential to regulate the hypoxic response, either alone or in combination with PHD inhibition. We report molecular, biophysical, and cellular evidence that the *N*-hydroxythiazole scaffold, reported to inhibit PHD2, is a useful broad spectrum 2OG oxygenase inhibitor scaffold, the inhibition potential of which can be tuned to achieve selective FIH inhibition. Structure-guided optimisation resulted in the discovery of *N*-hydroxythiazole derivatives that manifest substantially improved selectivity for FIH inhibition over PHD2 and other 2OG oxygenases, including Jumonji-C domain-containing protein 5 (∼25-fold), aspartate/asparagine-β-hydroxylase (>100-fold) and histone *N*^ε^-lysine demethylase 4A (>300-fold). The optimised *N*-hydroxythiazole-based FIH inhibitors modulate the expression of FIH-dependent HIF target genes and, consistent with reports that FIH regulates cellular metabolism, suppressed lipid accumulation in adipocytes. Crystallographic studies reveal that the *N*-hydroxythiazole derivatives compete with both 2OG and the substrate for binding to the FIH active site. Derivatisation of the *N*-hydroxythiazole scaffold has the potential to afford selective inhibitors for 2OG oxygenases other than FIH.

## Abstract 2

Optimised *N*-hydroxythiazoles are efficient and selective inhibitors of the 2-oxoglutrate dependent oxygenase factor inhibiting HIF, which has a key role in the human hypoxic response; the inhibitors decrease lipid accumualtion in adipocytes.

## Graphical abstract

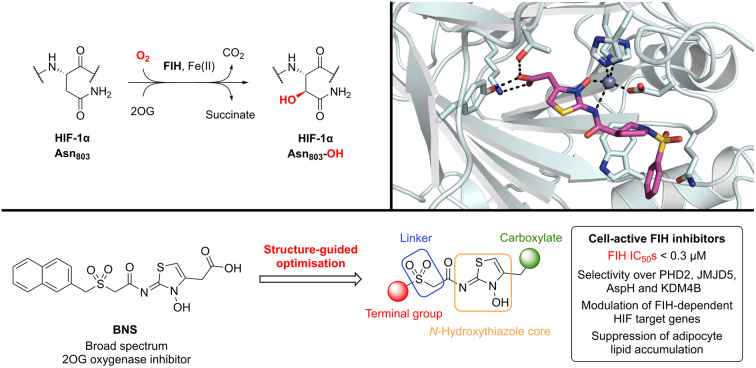

## Introduction

**Fig. 1:**
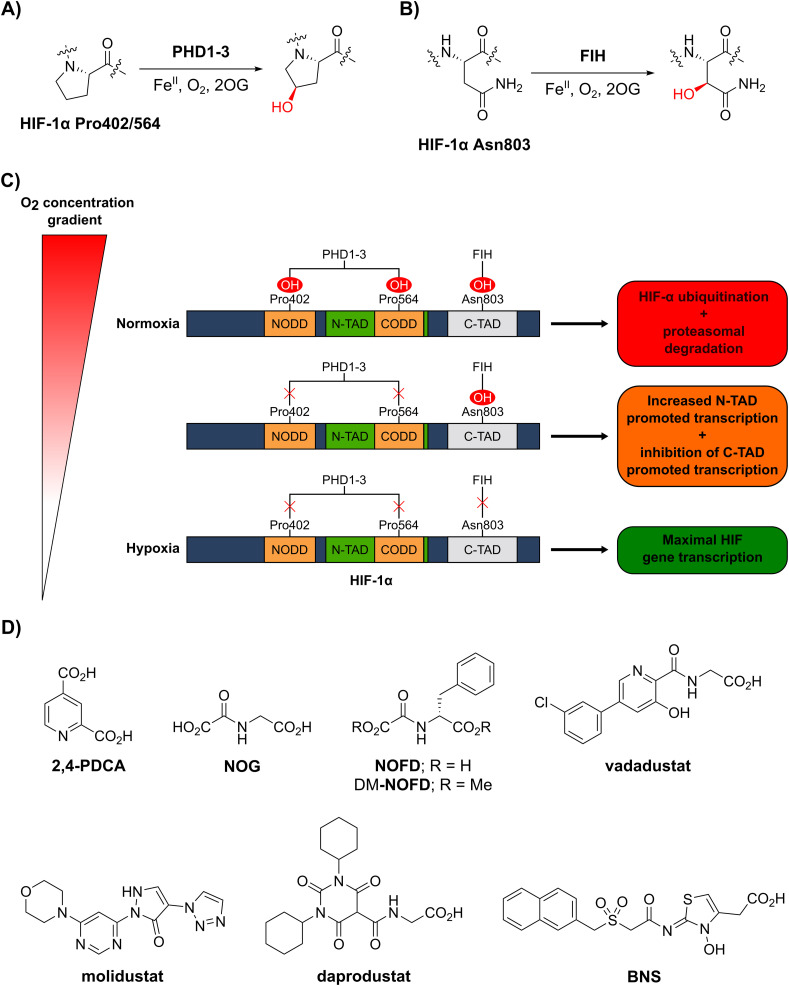
The human 2-oxoglutarate and Fe(ii)-dependent oxygenases PHD1–3 and FIH regulate the transcriptional activity of hypoxia-inducible factor (HIF) isoforms in an O_2_ availability-dependent manner by catalysing hydroxylation of HIF-α residues. (A) PHD1–3 catalyse the C4-prolyl-residue hydroxylation of HIF-α (Pro402 and Pro564 in HIF-1α), a modification that promotes HIF-α degradation *via* the ubiquitin-proteasome pathway.^9–12^ (B) FIH catalyses the C3-asparaginyl-residue hydroxylation of HIF-α (Asn803 in HIF-1α) resulting in context-dependent suppression of HIF C-terminal activation domain (C-TAD) transcription activity.^14–17^ (C) Schematic representation of the role of the PHDs and FIH in the HIF O_2_/hypoxia sensing system. (D) Reported FIH inhibitors: the broad-spectrum 2OG oxygenase inhibitors pyridine-2,4-dicarboxylic acid^27^ (2,4-PDCA) and *N*-oxalylglycine^28^ (NOG), the FIH-selective inhibitor *N*-oxalyl-d-phenylalanine (NOFD)^29^ and its dimethyl ester prodrug form dimethyl *N*-oxalyl-d-phenylalanine (DM-NOFD). Reported PHD2 inhibitors: vadadustat,^30^ molidustat,^31^ daprodustat^32^ and BNS.^17,33^

In order to maintain an adequate supply of dioxygen (O_2_) to tissues and cells, animals have evolved mechanisms to sense and respond to limiting O_2_ availability (hypoxia).^1–3^ One such mechanism involves the hypoxia-inducible factor (HIF) system, which regulates genes that work to ameliorate the effects of hypoxia and restore normal O_2_ supply.^4–6^ HIF is an α/β-heterodimeric transcription factor that plays important roles in both normal physiology and diseases, including cancer, in particular renal cell carcinoma.^7,8^ The abundance and transcriptional activity of HIF are controlled in an O_2_-dependent manner through post-translational modifications of HIF-α isoforms that are catalysed by the Fe(ii)- and 2-oxoglutarate (2OG)-dependent oxygenases prolyl residue hydroxylase domain-containing proteins 1–3 (PHD1–3) and factor inhibiting hypoxia-inducible factor-α (FIH) (Fig. 1A–C).^5,6^

PHD1–3 catalyse the C4-hydroxylation of two prolyl residues (Pro402 and Pro564 in human HIF-1α) in the N- and C-terminal oxygen-dependent degradation domains (ODDs) of HIF-α isoforms (Fig. 1A).^9^ The von Hippel-Lindau (pVHL) E3 ubiquitin ligase complex recognizes HIF-α prolyl hydroxylation, leading to polyubiquitination of HIF-α and its proteasomal degradation.^10–12^ PHD inhibitors increase HIF-α isoform levels and are used for the treatment of anaemia in chronic kidney disease.^13^ By contrast with the PHDs, FIH, which is part of a different structural subfamily of 2OG oxygenases,^14^ catalyses the C3-hydroxylation of a HIF-α asparagine residue (Gln803 in human HIF-1α) within the C-terminal activation domains (C-TADs) of HIF-1α and HIF-2α (Fig. 1B).^14,15^ HIF-α asparagine residue hydroxylation inhibits the interaction between the HIF-α C-TADs and the histone acetyl transferases and transcriptional coactivators p300/CBP resulting in suppression of C-TAD-mediated promotion of the transcriptional activity of α,β-HIF.^16,17^

O_2_ is a cosubstrate of both PHD1–3 and FIH, with the PHDs being more sensitive to O_2_ availability than FIH, as evidenced by kinetic analyses (PHD *K*_m_(O_2_) = 230–250 μM;^18^ FIH *K*_m_(O_2_) = 90 μM^19^).^20–22^ As O_2_ levels are depleted, PHD activity decreases, resulting in increased levels of HIF-α. In combination with HIF-β, HIF-α promotes the cellular hypoxic response by increasing the context-dependent expression of genes, including those encoding for erythropoietin (EPO)^23^ and vascular endothelial growth factor (VEGF).^24^ Under severe hypoxia,^25^ the activities of both the PHDs and FIH in cells are inhibited, and C-TAD-mediated promotion of HIF-α,β transcription is increased (Fig. 1C).^1,4,6,26^

Cellular studies have provided evidence that the sets of α,β-HIF target genes that are upregulated in hypoxia vary dependent on the context, including the cell type used.^17,34,35^ Exploiting the context-dependent nature of α,β-HIF gene expression is important from a therapeutic perspective, *e.g.* if anaemia treatment *via* α,β-HIF-mediated upregulation of the *EPO* gene is being targeted, concomitant upregulation of VEGF may be undesirable, as the latter has the potential to promote cancer.^36^ Interestingly, studies comparing the effects of hypoxia with those of broad spectrum 2OG oxygenase inhibitors or (at least) partially selective PHD inhibitors indicate that broad-spectrum inhibitors might better mimic hypoxia, at least in cells under laboratory conditions.^17^

Factors other than FIH, including other 2OG oxygenase related mechanisms, clearly have potential to regulate α,β-HIF target gene expression, *e.g.* the 2OG dependent JmjC histone *N*^ε^-lysine demethylase 4A (KDM4A) is reported to regulate HIF-1α abundance.^37^ The modulation of FIH activity is, however, of special interest with respect to controlling the set of α,β-HIF target genes upregulated, because, like the PHDs, it directly modifies HIF-α isoforms.^14,15^ There is good evidence that the role of FIH in the HIF system is context dependent.^17^ Thus, there is potential for the use of combinations of PHD and FIH inhibitors to ‘tune’ the set of HIF target genes upregulated, or, preferably, to have a single compound that manifests PHD and FIH inhibition activity, in a manner achieving the desired upregulation of a specific set of α,β-HIF target genes.

The rational control of FIH activity in a manner that leads to predicted physiological outcomes is, however, challenging, in part because of the general complexity of eukaryotic transcriptional regulation and the dynamic O_2_ availability-dependent nature of HIF-α levels. Further, by contrast with PHD1–3, for which substrates other than HIF-α have not yet been fully validated,^38^ there is biochemical and cellular evidence that FIH catalyses the C3-asparaginyl hydroxylation of multiple non-HIF substrates, including ankyrin repeat domain (ARD)-containing proteins NF-κB,^39,40^ apoptosis-stimulating p53-binding protein 2 (ASPP2)^41,42^ and Notch.^43^ FIH is also reported to catalyse the C3 hydroxylation of residues other than asparagine, including histidine-,^44^ tryptophan-^45,46^ and aspartate-residues.^47^

Cellular studies employing genetic methods have found that FIH also regulates small-molecule metabolism in addition to its role within the HIF system,^48^ potentially reflecting its apparent promiscuity with respect to protein substrates. Interestingly, deletion of the FIH gene in mice results in stimulation of both oxidative metabolism and glycolysis, resulting in an increase in cellular energy consumption.^48^ Furthermore, cellular peroxide inhibits hydroxylation of multiple FIH substrates at relatively low concentrations (<0.5 μM), suggesting that FIH may function as a sensor of oxidative stress.^49^ The underlying molecular mechanism of how FIH affects small-molecule metabolism and the pathophysiological relevance of the apparent pleiotropic cellular functions of FIH remain unclear. Selective small-molecule FIH inhibitors will therefore be of use to inform on the physiological functions of FIH.^50^

Whilst extensive work has focused on the development of PHD2 inhibitors,^51^ resulting *e.g.* in the clinical approval of daprodustat,^32^ vadadustat^30^ and FG-4592 (roxadustat),^52^ few small-molecule FIH inhibitors have been reported (Fig. 1D), and none have been approved for therapeutic use.^53^ The broad-spectrum 2OG oxygenase inhibitors pyridine-2,4-dicarboxylic acid (2,4-PDCA, FIH IC_50_: 5.0 μM)^54^ and *N*-oxalylglycine (NOG, FIH IC_50_: 0.36 μM), which are structural mimetics of 2OG, inhibit FIH *via* competitive displacement of 2OG at the active site.^27,28^ In addition, the reported PHD2 inhibitors vadadustat,^30^ molidustat,^31^ daprodustat^32^ and IOX4^55^ inhibit isolated recombinant FIH with weak/moderate potency (IC_50_s: 29 μM, 66 μM, 21 μM and 31 μM, respectively).^56^

A derivative of NOG has been identified with improved selectivity for FIH inhibition, *i.e. N*-oxalyl-d-phenylalanine (NOFD; FIH IC_50_: 0.24 μM).^29^NOFD manifests excellent selectivity for FIH inhibition over PHD2, and its dimethyl ester prodrug (DM-NOFD) has been used to inhibit FIH-catalysed HIF-1α Asn803 hydroxylation in cells.^17,57^ However, due to the limited options to chemically modify NOFD for further potency and selectivity optimization, and evidence that *N*-oxalyl amino acid derivatives can inhibit other 2OG oxygenases, including aspartate/asparagine-β-hydroxylase (AspH)^58^ and the JmjC KDMs,^59^ alternative lead scaffolds for FIH inhibitor development, suitable for fine tuning with respect to FIH and PHD inhibition, are required.

Here, we report mass spectrometric and crystallographic evidence that the reported *N*-hydroxythiazole-based PHD inhibitor BNS^17,33^ (Fig. 1D) is a broad-spectrum inhibitor of 2OG oxygenases, including FIH. Structure-guided SAR studies on the *N*-hydroxythiazole scaffold resulted in the discovery of potent *N*-hydroxythiazole-based small-molecule FIH inhibitors with similar potency to NOFD.^29^ Mass spectrometry (MS)-based inhibition assays reveal the potential of the *N*-hydroxythiazole scaffold to achieve high levels of selectivity with respect to inhibiting PHD2, and other 2OG oxygenases, *i.e.* Jumoni-C domain-containing protein 5 (JMJD5), AspH and KDM4A. Crystallographic studies inform on the mechanism of FIH inhibition and on the mode of metal binding at the active site. In cell-based studies, optimised *N*-hydroxythiazole-derived FIH inhibitors were found to upregulate the expression of the FIH-dependent HIF target gene *EGLN3* and to reduce adipocyte lipid accumulation, an observation which supports a proposed role of FIH in metabolic regulation.^48^ Importantly, our results imply that modifying the *N*-hydroxythiazole scaffold has potential for the design of selective inhibitors of 2OG oxygenases other than FIH.

## Results and discussion

### BNS is a broad-spectrum 2OG oxygenase inhibitor and a suitable lead scaffold for FIH-targeted inhibitor development

Considering that the PHD-selective inhibitors vadadustat^30^ and molidustat^31^ and daprodustat^32^ have been shown to moderately inhibit FIH *in vitro*,^56^ we initially investigated the effect of other reported PHD2 inhibitors on isolated recombinant human FIH (ESI Table S1†). IC_50_ values were determined using a solid-phase extraction coupled to mass spectrometry (SPE-MS)-based FIH inhibition assay, which monitors the mass change (*i.e.* +16 Da) associated with the FIH-catalysed hydroxylation of a HIF-1α-derived peptide, *i.e.* HIF-1α_788–822_.^60,61^ We observed that the reported PHD2 inhibitor BNS^17,33^ (Fig. 1D) inhibits FIH with similar potency (IC_50_ = 0.30 μM; Table 1, entry iii) as the FIH inhibitor NOFD (IC_50_ = 0.24 μM; Table 1, entry iv).^29^

**Table 1:** Comparison of 2OG oxygenase inhibition by BNS with the reported broad-spectrum 2OG oxygenase inhibitors pyridine-2,4-dicarboxylic acid (2,4-PDCA) and *N*-oxalylglycine (NOG), and the FIH selective inhibitor *N*-oxalyl-d-phenylalanine (NOFD)

Entry	Cmpd	IC_50_ [μM]^a^				
		FIH^b^	PHD2^c^	AspH^d^	KDM4A^e^	JMJD5^f^
i	2,4-PDCA	5.0 ± 2.1 (ref. 54)	5.3 ± 3.4 (ref. 54)	0.03 ± 0.01 (ref. 54)	0.10 ± 0.00	0.33 ± 0.07 (ref. 70)
ii	NOG	0.36 ± 0.03	12.3 ± 4.4	1.1 ± 0.3 (ref. 58)	22.1 ± 1.1	0.15 ± 0.02 (ref. 70)
iii	BNS	0.30 ± 0.07	0.11 ± 0.00	3.4 ± 0.1	67.4 ± 39.8	0.25 ± 0.01
iv	NOFD	0.24 ± 0.02	>100	15.5 ± 1.2 (ref. 60)	14.1 ± 0.0	>100 (ref. 70)

a Mean average ± standard deviation (SD) of two independent experiments (each composed of technical duplicates).

b Using 0.15 μM FIH, 10.0 μM 2OG and 5.0 μM of a HIF-1α C-terminal transactivation domain fragment (HIF-1α C-TAD_788–822_).^61^

c Using 0.15 μM PHD2_181–426_, 10.0 μM 2OG and 5.0 μM of a HIF-1α C-terminal oxygen-dependent degradation domain fragment (HIF-1α CODD_556–574_).^61^

d Using 0.05 μM His_6_-AspH_315–758_, 3.0 μM 2OG and 1.0 μM of a cyclic peptide based on human Factor X (hFX-CP_101–119_).^58^

e Using 0.15 μM KDM4A, 10.0 μM 2OG and 10.0 μM of a variant of histone 3 (H3_1–15_K9me3_1–15_).^69^

f Using 0.15 μM JMJD5, 2.0 μM 2OG and 2.0 μM of a 40S ribosomal protein S6 fragment (RSP6_128–148_).^70^ Inhibition assays were performed using SPE-MS as described in the ESI.

**Fig. 2:**
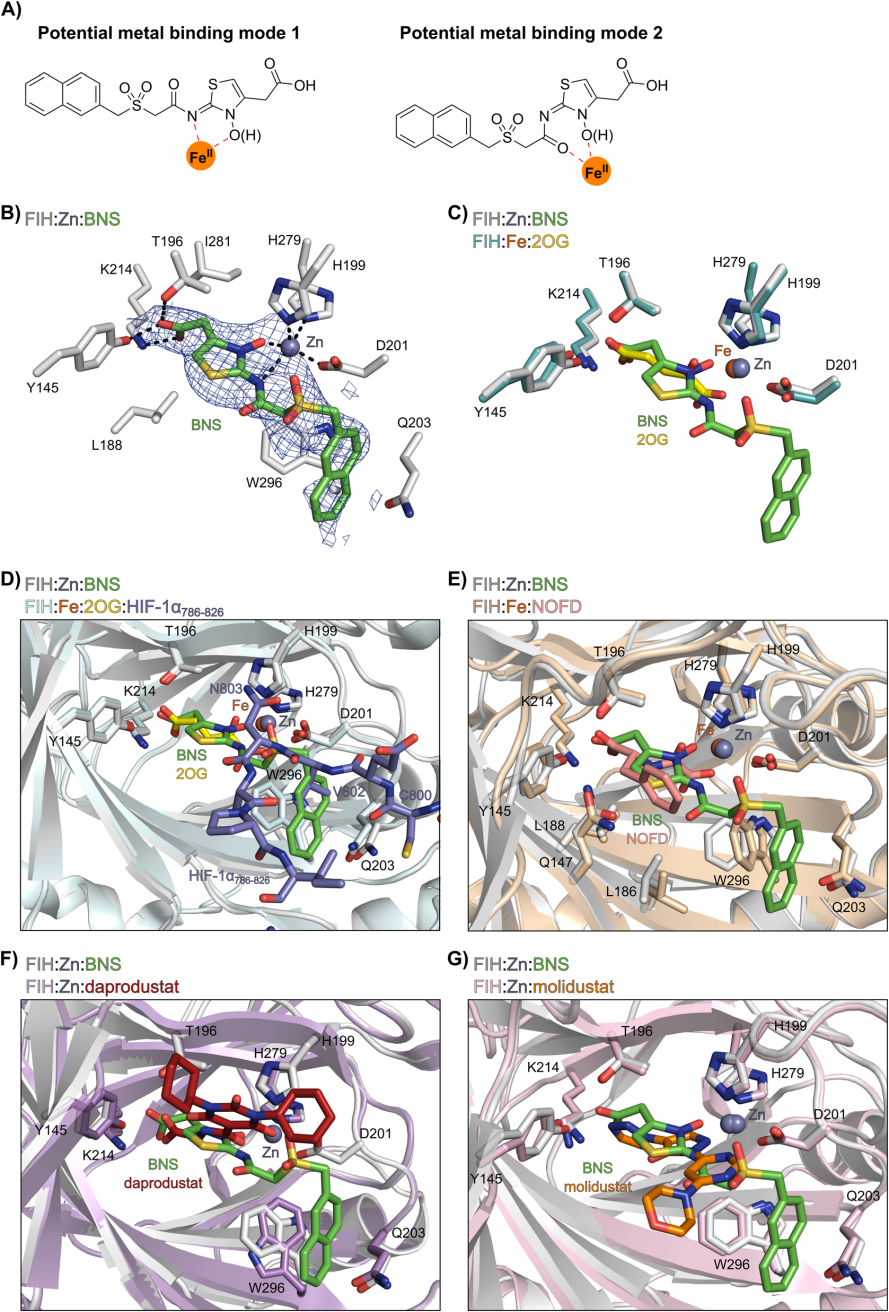
Crystallographic studies indicate that BNS binds to the FIH active site in a 2OG- and HIF-1α-competitive manner. Colour code: light grey: FIH; green: carbon-backbone of BNS; yellow: carbon-backbone of 2OG; dark grey: zinc; orange: iron; red: oxygen; blue: nitrogen and yellow: sulphur. (A) Two alternative modes have been proposed for how BNS binds to Fe(ii) in the PHD2 active site.^33^ (B) Active site view from the FIH:Zn:BNS complex structure (PDB ID: 8K71) showing the OMIT electron density map (mFo-DFc) contoured to 2.1 σ around BNS. (C–F) Superimposition of active site views from the FIH:Zn:BNS complex structure and reported (C) FIH:Fe:2OG (PDB ID: 1MZF;^66^ teal: FIH), (D) FIH:Fe:2OG:HIF-1α_786–826_ (PDB ID: 1H2L;^28^ light blue: FIH; purple: carbon-backbone of HIF-1α), (E) FIH:Fe:NOFD (PDB ID: 1YCI;^29^ ochre: FIH; pink: carbon-backbone of NOFD), (F) FIH:Zn:daprodustat (PDB ID: 5OP6;^56^ light purple: FIH; deep red: carbon-backbone of daprodustat) and (G) FIH:Zn:molidustat (PDB ID: 5OP8;^56^ light pink: FIH; orange: carbon-backbone of molidustat) complex structures.

Most reported 2OG oxygenase inhibitors, including the above mentioned PHD inhibitors, coordinate to the active site Fe(ii) and compete with 2OG for binding, as pioneered in studies on inhibition of collagen prolyl hydroxylase and plant 2OG oxygenases.^62,63^ However, it is unclear how BNS binds to the active site of the PHDs; pioneering modelling and kinetic studies with *N*-hydroxythiazole-derived PHD inhibitors suggested two possible 2OG-competitive PHD2 binding modes.^33,64^ In each of these, the terminal carboxylate of BNS was proposed to bind in a similar manner to the 2OG C5 carboxylate, *i.e.* forming hydrogen bonds with the side chains of Tyr329 and Arg383. The mode of Fe(ii) coordination was less clear, with two potential bidentate binding modes being considered – one involving coordination *via* the *N*-hydroxyl group and the exocyclic nitrogen atom of the *N*-hydroxythiazole unit (*i.e.* a 5-membered chelate ring) and the other *via* the *N*-hydroxyl group and the acetamide oxygen atom (*i.e.* a 7-membered chelate ring; Fig. 2A).

To investigate its binding mode to the active site of a 2OG oxygenase, BNS was co-crystallised with FIH in the presence of Zn(ii), which was used as a catalytically inert surrogate for Fe(ii). The structure was solved by molecular replacement (MR) using a reported FIH structure (PDB ID: 4B7K^65^) as a search model. The FIH:Zn:BNS complex structure (PDB ID: 8K71; space group: *P*4_1_2_1_2, resolution: 2.23 Å) reveals that BNS binds at the active site and coordinates Zn(ii) in a bidentate manner *via* the *N*-hydroxyl group (O–Zn distance: 2.1 Å) and the *exo*-nitrogen atom (N–Zn distance: 2.7 Å) of its *N*-hydroxythiazole unit (Fig. 2B). Thus, the structural analysis implies that BNS binds to FIH *via* the proposed metal binding mode 1 (Fig. 2A); note, that BNS might bind to the PHDs *via* different modes; however, our efforts to crystallize BNS with PHD2 were unsuccessful.

Superimposition of the FIH:Zn:BNS complex structure with reported FIH:Fe:2OG (PDB ID: 1MZF;^66^ Cα RMSD = 0.30 Å) FIH:Fe:2OG:HIF-1α_786–826_ (PDB ID: 1H2L;^28^ Cα RMSD = 0.28 Å) complex structures indicates that BNS competes with both 2OG and HIF-1α for binding FIH (Fig. 2C and D). Notably, the orientation of the metal coordination mode of BNS is different to that observed crystallographically for 2OG (Fig. 2C) and NOFD (PDB ID: 1YCI;^29^Fig. 2E), as previously observed in FIH structures in complex with daprodustat (PDB ID: 5OP6;^56^Fig. 2F), molidustat (PDB ID: 5OP8;^56^Fig. 2G) and vadadustat (PDB ID: 5OPC;^56^ ESI Fig. S5†).^56,67^ The hydroxyl group of BNS binds Zn(ii) in the same manner as the 2OG ketone carbonyl (*i.e. trans* to Asp201); however, the interaction between Zn(ii) and the *N*-hydroxythiazole *exo*-nitrogen, which occupies the coordination site *trans* to His279, is perpendicular to that observed for the C1 carboxylate of 2OG, which binds *trans* to His199. The carboxylate of BNS is positioned to interact with the side chains of FIH residues Tyr145 (2.2 Å), Thr196 (3.0 Å), and Lys214 (2.5 Å), mimicking the interactions of the 2OG C5 carboxylate with FIH (Fig. 2C). In complex with FIH, daprodustat has also been found to engage in hydrogen bonding with Tyr145, Thr196 and Lys214 through its carboxylate group (Fig. 2F).^56^ By contrast, in the FIH:Zn:vadadustat complex structure, the carboxylate of vadadustat orients away from the side chains of Tyr145 and Thr196, and only interacts with Lys214 (ESI Fig. S5†).^56^ The triazole ring of molidustat is observed crystallographically to engage in hydrogen bonds with Tyr145 and Lys214; however, not with Thr196 (Fig. 2G).^56^

The FIH structure in complex with BNS reveals that the naphthalene group of BNS extends into the HIF-1α substrate binding pocket and is positioned to engage in π–π and amide-π stacking interactions with the side chains of Trp296 (face-to-face) and Gln203 (edge-to-face), respectively (Fig. 2D). As a result, the binding of BNS to FIH likely disrupts the hydrophobic interaction between the indole group of Trp296 and the conserved hydrophobic substrate residue (*e.g.* Val802 in HIF-1α) that is adjacent to the asparagine residue undergoing hydroxylation, which biochemical studies have shown is essential for efficient FIH catalysis.^68^ Analysis of a reported FIH:Zn:2OG:HIF-1α_786–826_ complex structure (PDB ID: 1H2L;^28^Fig. 2D) indicates that the primary amide side chain of Gln203 is involved in substrate recognition, by forming a hydrogen bond with the backbone amide oxygen atom of HIF-1α Cys800. Interestingly, neither daprodustat or molidustat appear to interact with Gln203 and/or Trp296 in complex with FIH (Fig. 2F and G). Given the importance of Gln203 and, in particular, Trp296 for substrate binding,^68^ the hydrophobic interactions between the naphthalene unit of BNS, Gln203 and Trp296 are potentially contributing factors behind the observed increased in FIH inhibitory activity of BNS compared with other PHD inhibitors (>50-fold, as judged by IC_50_ comparison, ESI Table S1†),^56^ in addition to the three hydrogen bonds made by the BNS carboxylate with FIH, unlike molidustat and vadadustat (ESI Fig. S5†).

To investigate the potential of BNS for selective FIH inhibition, its selectivity was investigated with respect to other 2OG-dependent protein oxidising enzymes, that is PHD2, Jumonji-C domain-containing protein 5 (JMJD5), aspartate/asparagine-β-hydroxylase (AspH) and KDM4A, using reported SPE-MS inhibition assays.^58,61,69,70^ In addition to PHD2 and KDM4A, we included AspH and JMJD5 in our selectivity studies, because AspH, like FIH, catalyses protein aspartate- and asparagine-residue hydroxylation (although in epidermal growth factor-like domains),^71,72^ and because JMJD5 is structurally closely related to FIH.^73,74^ The results reveal that, in addition to being a potent PHD2 (IC_50_ = 0.11 μM; Table 1, entry iii) and FIH inhibitor, BNS inhibits JMJD5 (IC_50_ = 0.25 μM) and AspH (IC_50_ = 3.36 μM), whereas inhibition of KDM4A was not observed (IC_50_ > 100 μM).

The combined results thus indicate that BNS is in fact a relatively broad-spectrum inhibitor of 2OG oxygenases, similar to 2,4-PDCA and NOG (Table 1, entries i and ii). Importantly, however, the results also reveal that the selectivity profile of BNS for 2OG oxygenase inhibition differs from that of both 2,4-PDCA and NOG. For example, 2,4-PDCA inhibits JMJD5, AspH, and KDM4A substantially more efficiently that PHD2 and FIH, whereas NOG manifests more potent inhibition of FIH, JMJD5 and AspH than PHD2 and KDM4A. This observation is of interest as it indicates that the use of particular broad-spectrum 2OG oxygenase inhibitors could result in different biological outcomes due to the inhibition of a different subset of 2OG oxygenases.

**Fig. 3:**
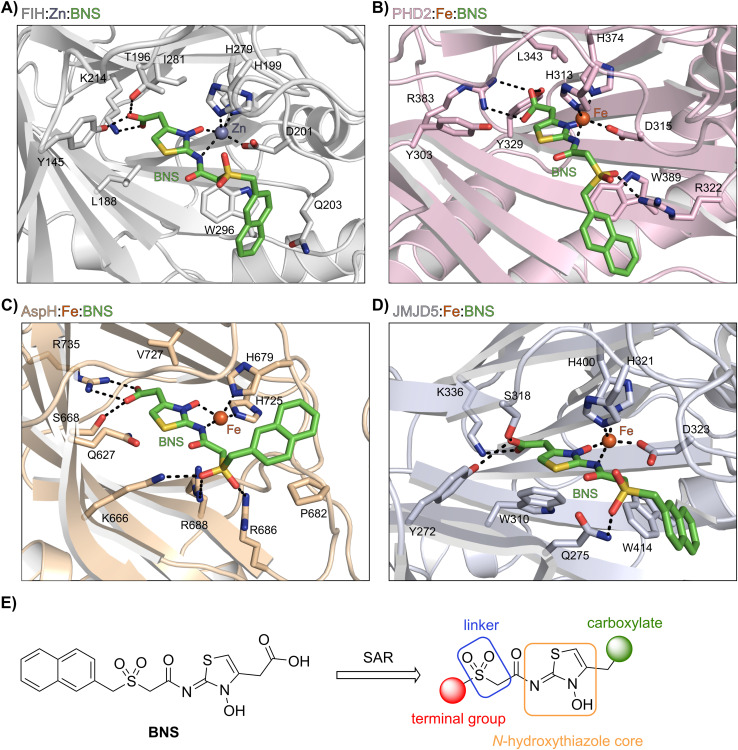
Docking studies predict that BNS may bind to the active sites of PHD2, AspH and JMJD5 with similar binding modes to that observed in the FIH:Zn:BNS complex structure. Colour code: green: carbon-backbone of BNS; dark grey: zinc; orange: iron; red: oxygen; blue: nitrogen and yellow: sulphur. (A) Active site view from the FIH:Zn:BNS complex structure (PDB ID: 8K71; light grey: FIH). (B–D) Active site views from: (B) the PHD2:Fe:BNS docking prediction (pink: PHD2), (C) the AspH:Fe:BNS docking prediction (ochre: AspH) and (D) the JMJD5:Fe:BNS docking prediction (light blue: JMJD5). (E) Outline of the strategies used for selectivity optimisation of the *N*-hydroxythiazole scaffold.

BNS likely does not inhibit KDM4A because of the different geometry of the KDM4A active site compared to those of FIH, PHD2, JMJD5 and AspH.^75^ Thus, superimposition of the FIH:Zn:BNS complex structure and a reported KDM4A:Ni:2OG complex structure (PDB ID: 6H8P^76^) indicates that the naphthalene group of BNS likely clashes with KDM4A residues Thr289 and Asn290, which form part of β-strand VIII of the rigid β-barrel core fold of the KDM4A active site, therefore preventing efficient binding of BNS to KDM4A (ESI Fig. S6†). By contrast, modelling studies predict that BNS can likely adopt binding modes within the PHD2, JMJD5 and AspH active sites that are analogous to the FIH:Zn:BNS complex structure (Fig. 3A–D).

The *N*-hydroxythiazole core of BNS is predicted to bind within the 2OG binding pockets of PHD2, AspH and JMJD5 and to coordinate to the metal in a bidentate manner, consistent with the observation that *N*-hydroxythiazoles inhibit recombinant PHD2 *via* a 2OG-competitive mechanism.^64^ The BNS carboxylate group is predicted to form hydrogen bond interactions with residues that interact with the 2OG C5 carboxylate (Tyr329 and Arg383 in PHD2;^77^ Ser668 and Arg735 in AspH;^78^ Tyr272, Ser318 and Lys336 in JMJD5;^74^Fig. 3B–D), as observed in the FIH:Zn:BNS complex structure, and the naphthalene unit likely extends into the respective peptide substrate binding pockets.

The docking results also indicate that the sulfone moiety of BNS will be positioned to form hydrogen bonds with the side chains of active site residues in PHD2, AspH and JMJD5 (Arg322 in PHD2; Lys666, Arg686 and Arg688 in AspH; Gln275 in JMJD5), an observation that contrasts with the FIH:Zn:BNS complex structure, in which the oxygen atoms of the sulfone group are solvent exposed. The residues that are predicted to interact with the sulfone of BNS in, at least, AspH^78^ and PHD2,^79^ have been observed by crystallography to be involved in substrate recognition, suggesting that the sulfone of BNS may be important to prevent productive substrate binding to AspH and PHD2.

Notably, the BNS modelling study implied that the orientation of the naphthalene unit of BNS may be different for BNS in complex with PHD2, AspH and JMJD5, as compared with that observed in the FIH:Zn:BNS complex structure (Fig. 3A–D). The difference in naphthalene binding may be due to the flexibility of the sulfone and methylene units that connect the *N*-hydroxythiazole core and naphthalene ring of BNS, and likely contributes to BNS being a relatively broad spectrum 2OG oxygenase inhibitor compared to more structurally rigid 2OG-competitive inhibitors such as daprodustat.

Overall, the above described results indicate that the *N*-hydroxythiazole scaffold of BNS is attractive for the development of selective FIH inhibitors, because (i) the FIH:Zn:BNS complex structure can be used to guide inhibitor design, (ii) the structure of BNS is modular and can be chemically modified, (iii) the BNS*N*-hydroxythiazole scaffold has potential for fine tuning inhibition of FIH and the PHDs, and (iv) BNS is active in cell-based studies.^17,80^ We therefore carried out SAR studies directed at optimising the selectivity of BNS for FIH inhibition. As part of the SAR study, the four main structural features of BNS, *i.e.* the *N*-hydroxythiazole core, the carboxylate group, the linker region and the terminal naphthalene moiety, were systematically varied (Fig. 3E).

### Structure–activity relationship studies towards optimised *N*-hydroxythiazole-based FIH inhibitors

#### Variations on the *N*-hydroxythiazole core of BNS

**Scheme 1:**
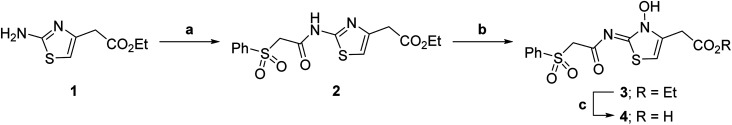
Synthesis of *N*-hydroxythiazole derivative 4.^*a a*^Reagents and conditions: (a) 2-(phenylsulfonyl)acetic acid, T3P,^82^*i*Pr_2_NEt, DMF, 0 °C to rt, 82%; (b) mCPBA, CHCl_3_, rt, 71%; (c) LiOH, MeOH/H_2_O, 0 °C to rt, 43%.

To facilitate the planned SAR study, we initially synthesized *N*-hydroxythiazole derivative 4 in which the 2-naphthylmethyl moiety of BNS was substituted for a phenyl group, because 2-((phenyl)sulfonyl)acetic acid was commercially available, unlike 2-((2-naphthylmethyl)sulfonyl)acetic acid. *N*-Hydroxythiazole 4 was prepared in three steps from thiazole 1 in an overall yield of 25%, according to a modified literature procedure (Scheme 1).^33,81^ Initially, 1 was coupled with 2-(phenylsulfonyl)acetic acid using T3P^82^ to generate amide 2. Subsequent *meta*-chloroperbenzoic acid (mCPBA)-mediated thiazole *N*-oxidation and lithium hydroxide-mediated ester saponification afforded carboxylic acid 4. Compound 4 and all related *N*-hydroxythiazole derivatives described herein (see below) have been putatively assigned as the (*Z*)-isomers based on a previously reported *N*-hydroxythiazole small-molecule crystal structure^33^ and based on the crystal structures of *N*-hydroxythiazole derivatives in complex with FIH reported herein (*e.g.*, Fig. 2B).

Importantly, the naphthyl to phenyl group substitution of BNS did not affect the potency of FIH inhibition; however, it appeared that 4 inhibited PHD2, AspH and JMJD5 less efficiently than BNS, thus supporting our proposal that changes in the BNS structure can alter its selectivity profile (Table 2, entries i and ii). To investigate the effect of the *N*-hydroxyl group of 4 in FIH inhibition, we synthesized derivative 5 which lacks the *N*-hydroxyl group (ESI Scheme S1†). Notably, 5 did not inhibit the tested 2OG oxygenases (Table 2, entry iii), highlighting the importance of metal chelation for efficient inhibition by *N*-hydroxythiazoles, as indicated by the FIH:Zn:BNS complex structure (Fig. 2B).

**Table 2:** Inhibition of 2OG human oxygenases by selected thiazole and *N*-hydroxythiazole analogues

Entry	Cmpd^a^	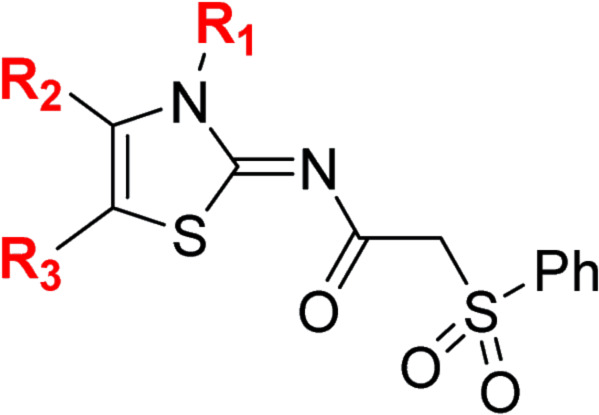			IC_50_ [μM]^b^				
		R_1_	R_2_	R_3_	FIH^c^	PHD2^d^	AspH^e^	KDM4A^f^	JMJD5^g^
i	BNS^h^	OH	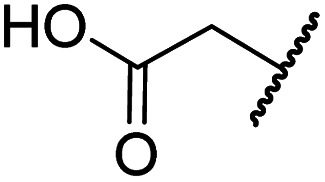	H	0.30 ± 0.07	0.11 ± 0.00	3.4 ± 0.1	67.4 ± 39.8	0.25 ± 0.01
ii	4	OH	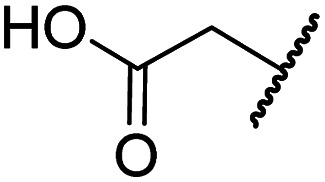	H	0.28 ± 0.00	0.50 ± 0.05	9.6 ± 1.3	>100	0.48 ± 0.01
iii	5	H	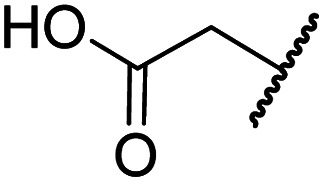	H	>100	>100	>100	>100	>100
iv	6	OH	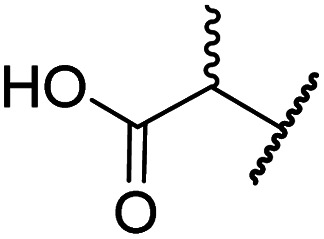	H	61.0 ± 3.1	5.2 ± 2.6	25.6 ± 12.7	>100	60.5 ± 1.0
v	7	OH	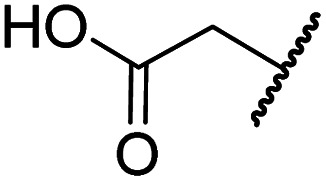	Me	36.7 ± 4.9	3.1 ± 1.4	24.3 ± 12.3	>100	>100
vi	8	OH	H	H	>100	>100	>100	>100	>100
vii	9	OH	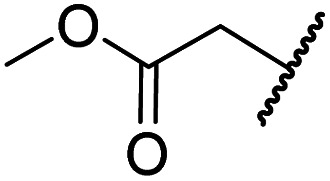	H	>100	>100	96.7 ± 3.2	>100	>100
viii	3	OH	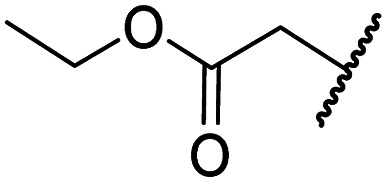	H	>100	>100	68.0 ± 26.8	>100	>100
ix	10	OH	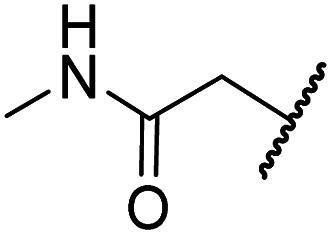	H	>100	>100	43.6 ± 3.7	>100	77.5 ± 23.8
x	11	OH	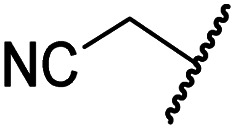	H	>100	>100	>100	>100	>100
xi	12	OH	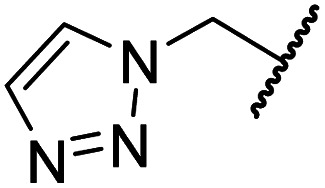	H	>100	>100	83.3 ± 6.7	>100	>100
xii	13	OH	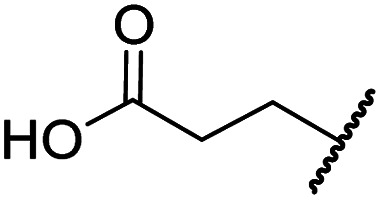	H	2.2 ± 0.6	3.0 ± 1.3	47.3 ± 4.7	>100	>100

a All chiral *N*-hydroxythiazole derivatives were prepared as racemic mixtures.

b Mean average ± SD of two independent experiments (each composed of technical duplicates).

c Using 0.15 μM FIH, 10.0 μM 2OG and 5.0 μM HIF-1α C-TAD_788–822_.^61^

d Using 0.15 μM PHD2_181–426_, 10.0 μM 2OG and 5.0 μM HIF-1α CODD_556–574_.^61^

e Using 0.05 μM His6-AspH_315–758_, 3.0 μM 2OG and 1.0 μM hFX-CP_101–119_.^58^

f Using 0.15 μM KDM4A, 10.0 μM 2OG and 10.0 μM H3_1–15_K9me3_1–15_.^69^

g Using 0.15 μM JMJD5, 2.0 μM 2OG and 2.0 μM RSP6_128–148_.^70^

h BNS has a 2-naphthylmethyl sulfonyl group rather than a phenyl sulfonyl group. Inhibition assays were performed using SPE-MS as described in the ESI.

In the FIH:Zn:BNS complex structure, the C5 position of the *N*-hydroxythiazole ring of BNS and the methylene unit that connects the *N*-hydroxythiazole ring with the terminal carboxylate are positioned adjacent to the hydrophobic side chains of Leu188 and Ile281, respectively (Fig. 2B). To investigate whether substituents at these positions affect inhibitor potency and selectivity by enhancing hydrophobic interactions with Leu188 and/or Ile281, the corresponding methyl-substituted derivatives of 4 were synthesised, *i.e.*6 and 7 (ESI Scheme S2†). 6 and 7 showed substantially reduced levels of FIH inhibition compared to 4 (>100-fold; Table 2, entries iv and v), indicating a potential steric clash with the side chains of Leu188 and/or Ile281. Hence, further substitutions at these positions were not explored. Note that, while 7 inhibited PHD2 ∼ 30-fold less efficiently than 4, its selectivity for PHD2 inhibition over FIH inhibition increased by ∼20-fold compared to that of 4 (12-fold for 7*vs.* not PHD2 selective for 4). 7 also showed no inhibition of JMJD5 and KDM4A (IC_50_ > 100 μM), an observation which may be relevant for the future development of optimised *N*-hydroxythiazole-based PHD2 inhibitors, including with an improved selectivity profile.

#### Variations on the BNS carboxylate

BNS and many other reported 2OG oxygenase inhibitors contain carboxylate groups that mimic binding of the 2OG C5 carboxylate (Fig. 2D).^62^ Although BNS is active in cells,^17,80^ the presence of carboxylates in small-molecule inhibitors can reduce cellular efficacy^83,84^ and often requires derivatisation to ester pro-drugs to improve cell-wall permeability.^85^ Therefore, 2OG oxygenase inhibitors have been developed in which the carboxylate is replaced with a suitable bioisostere,^31,86,87^ which has also been shown to alter the selectivity profile of the inhibitor as different 2OG oxygenases employ different residues to interact with the 2OG C5 carboxylate.^31,62,67,86^ Consequently, the *N*-hydroxythiazole analogues 8–12 were prepared (ESI Scheme S3a–e†) to investigate whether removing or replacing the carboxylate group of BNS with groups that could engage in similar hydrogen bonding interactions with Tyr145, Thr196 and Lys214, as observed in the FIH:Zn:BNS complex structure (Fig. 2B), affects FIH inhibition and selectivity.

The removal of the *N*-hydroxythiazole carboxylate side chain (as in 8), as well as the substitution of the carboxylate with bioisosteres such as amide (as in 10), nitrile (as in 11) and triazole (as in 12) groups resulted in the complete loss of FIH inhibition (Table 2, entries vi–xi), highlighting the importance of the hydrogen bonding interactions between the carboxylate and the FIH active site residues. Increasing the distance between the carboxylate and *N*-hydroxythiazole ring, as *e.g.* in compound 13 (ESI Scheme S3f†), led to a ∼10-fold reduction in both PHD2 and FIH inhibition relative to 4 (FIH IC_50_: 2.2 μM; Table 2, entry xii). Hence, further modifications of the BNS carboxylate group or thiazole C4 substituent were not investigated.

#### Variations on the BNS methylene sulfone linker

Docking of BNS into the PHD2, AspH and JMJD5 active site indicates that its sulfone may interact with the side chains of residues, which, at least for PHD2 and AspH, are involved in substrate binding, *i.e.* Arg322 (PHD2), Lys666, Arg686 and Arg688 (AspH), and Gln275 (JMJD5) (Fig. 3B–D). Since equivalent interactions were not observed in the FIH:Zn:BNS complex structure (Fig. 2B), modification of the methylene sulfone linker, which connects the *N*-hydroxythiazole and phenyl groups of 4, was explored as a potential strategy to enhance selectivity for FIH inhibition. Thus, *N*-hydroxythiazole analogues 14–23 that contain alternative linkers, including amide, sulfonamide, aniline, and ether groups, were prepared *via* modification of the route used for the synthesis of 4, using alternative carboxylic acid and/or acid chloride coupling partners in the initial amide formation step (ESI Scheme S4†).

Whilst the truncated *N*-acetylated BNS derivative 14 was >200-fold less efficient in inhibiting FIH than 4 (IC_50_: 52.4 μM; Table 3, entry ii), compounds 15–23 were all relatively efficient FIH inhibitors (IC_50_ < 10 μM; Table 3, entries iii–xi). This observation indicates that the terminal phenyl substituent is important for FIH inhibition. Sulfones 4 (IC_50_: 0.28 μM) and 15 (IC_50_: 0.30 μM), and sulfonamide 21 (IC_50_: 0.36 μM) were the most potent FIH inhibitors within this series (Table 3, entries i, iii and ix), an observation which may reflect their increased ability to engage in hydrophobic contacts with the side chains of Gln203 and Trp296, as observed in the FIH:Zn:BNS complex structure (Fig. 2B). Both 4 and 21 were also efficient PHD2 inhibitors (IC_50_s: 0.50 μM and 0.57 μM, respectively). By contrast, 15, in which an ethylene group connects the *N*-hydroxythiazole core and the sulfone linker, showed decreased inhibition of PHD2 (IC_50_: 2.7 μM), JMJD5 (IC_50_: 3.3 μM) and AspH (IC_50_: 33.0 μM), relative to 4.

**Table 3:** Inhibition of human 2OG oxygenases by selected derivatives of *N*-hydroxythiazole 4 with modifications to the methylene sulfone linker

Entry	Cmpd	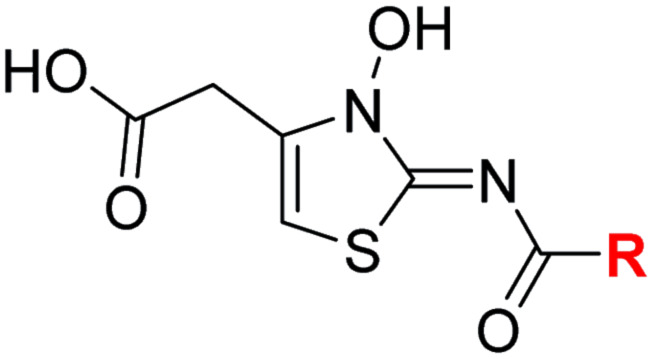	IC_50_ [μM]^a^				
		R	FIH^b^	PHD2^c^	AspH^d^	KDM4A^e^	JMJD5^f^
i	4	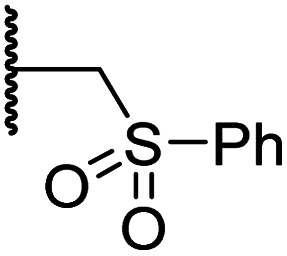	0.28 ± 0.00	0.50 ± 0.05	9.6 ± 1.3	>100	0.48 ± 0.01
ii	14	Me	52.4 ± 30.4	>100	>100	>100	3.2 ± 0.7
iii	15	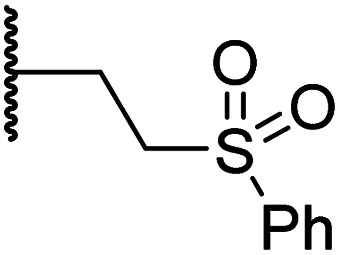	0.30 ± 0.05	2.7 ± 0.2	33.0 ± 12.1	>100	3.3 ± 1.5
iv	16	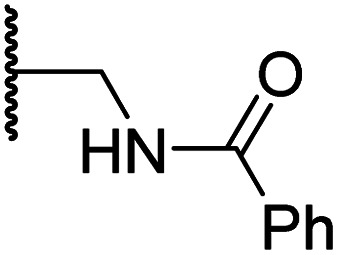	1.2 ± 0.2	1.2 ± 0.2	2.4 ± 1.1	>100	1.7 ± 0.3
v	17	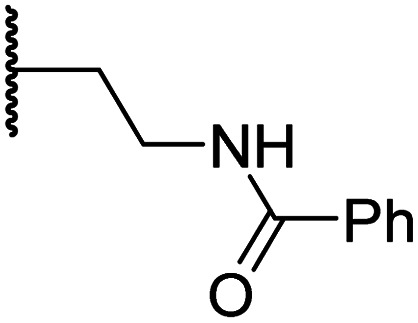	4.6 ± 0.7	3.6 ± 0.4	28.3 ± 6.5	>100	4.6 ± 1.7
vi	18	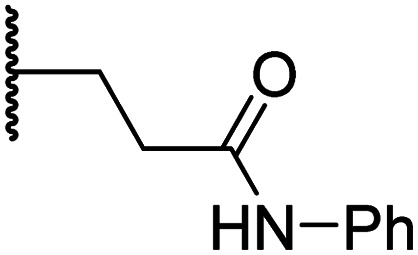	6.0 ± 1.2	2.3 ± 0.1	3.7 ± 1.5	>100	8.4 ± 2.7
vii	19	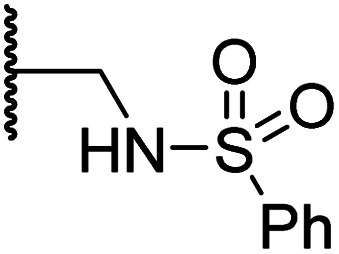	3.7 ± 1.5	15.5 ± 4.6	3.1 ± 0.4	17.4 ± 0.2	12.0 ± 0.6
viii	20	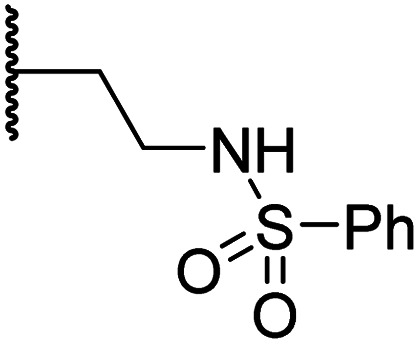	1.0 ± 0.1	6.5 ± 1.8	23.3 ± 10.1	>100	13.4 ± 6.5
ix	21	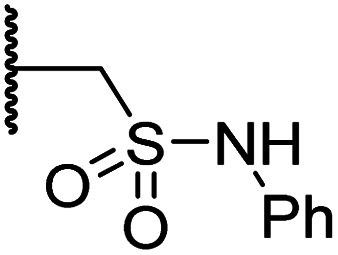	0.36 ± 0.21	0.57 ± 0.18	0.76 ± 0.10	>100	2.8 ± 0.9
x	22	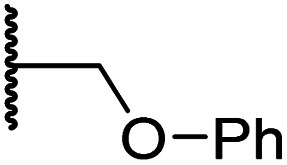	0.75 ± 0.23	2.6 ± 0.2	20.2 ± 6.6	>100	1.2 ± 0.2
xi	23	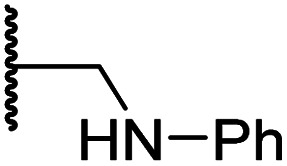	3.2 ± 1.1	10.3 ± 2.9	11.1 ± 0.4	71.6 ± 11.8	4.7 ± 1.1

a Mean average ± SD of two independent experiments (each composed of technical duplicates).

b Using 0.15 μM FIH, 10.0 μM 2OG and 5.0 μM HIF-1α C-TAD_788–822_.^61^

c Using 0.15 μM PHD2_181–426_ and 5.0 μM HIF-1α CODD_556–574_.^61^

d Using 0.05 μM His_6_-AspH_315–758_, 3.0 μM 2OG and 1.0 μM hFX-CP_101–119_.

e Using 0.15 μM KDM4A, 10.0 μM 2OG and 10.0 μM H3_1–15_K9me3_1–15_.^69^

f Using 0.15 μM JMJD5, 2.0 μM 2OG and 2.0 μM RSP6_128–148_.^70^ Inhibition assays were performed using SPE-MS as described in the ESI.

Sulfonamide 20, which, like sulfone 15, contains an ethylene chain that bridges the *N*-hydroxythiazole core and sulfonamide linker, and ether 22 also retained potent FIH inhibition (IC_50_s: 1.0 μM and 0.75 μM, respectively; Table 3, entries viii and x). Furthermore, 20 and 22 did not substantially inhibit KDM4A or AspH, and 20 displayed ∼6-fold selectivity for FIH inhibition over PHD2 inhibition and ∼13-fold selectivity over JMJD5 inhibition, which is ∼3-fold and ∼8-fold greater than that of 4, respectively. This observation indicates that extending the alkylene unit connecting the *N*-hydroxythiazole unit and linker group may be a viable approach to increase selectivity for FIH inhibition, potentially resulting from disruption of the predicted hydrogen bonding interactions by the BNS sulfone in the PHD2, JMJD5 and AspH substrate binding sites (Fig. 3B–D). Replacement of the sulfonamide of 20 with an amide (17; IC_50_: 4.6 μM) and truncation of the ethylene chain (19; IC_50_: 3.7 μM) led to a ∼5-fold and ∼4-fold loss in FIH inhibition, respectively (Table 3, entries v and vii).

Interestingly, sulfonamide 21 was the most active inhibitor of AspH among the tested *N*-hydroxythiazole derivatives (IC_50_: 0.76 μM; Table 3, entry ix), being ∼5-fold more potent than 4. Docking studies indicate that the sulfonamide moiety of 21 may be appropriately positioned to interact with the polar side chains of Arg686, Arg688 and Glu617 (ESI Fig. S10†). AspH is a proposed medicinal chemistry target^54,88^ associated with the pathologies of various cancers,^89,90^ including hepatocellular carcinoma^88,91^ and pancreatic cancer,^93^ hence, derivatisation of 21 to develop potent AspH inhibitors warrants further investigation.^92^

#### Linker rigidification improves selectivity for FIH inhibition

The ethylene chain connecting the *N*-hydroxythiazole core and the sulfone/sulfonamide linker groups of compounds 15 and 20 is likely conformationally flexible in solution. As predicted for the naphthalene group of BNS, this flexibility may enable the terminal phenyl unit to occupy different conformations within the substrate binding pockets of FIH, PHD, JMJD5 and AspH (Fig. 3), thus potentially limiting selectivity. We therefore investigated whether rigidification of the linker chain *via* cyclisation would increase selectivity for FIH inhibition. Sulfonamide 20 was considered more appropriate to initially test this hypothesis than 15 due to the commercial availability of suitable cyclic β-amino acid derivatives that enable facile modification of 20. Thus, we co-crystallised FIH with 20 to guide further SAR studies (FIH:Zn:20; PDB ID: 8K72; space group: *P*4_1_2_1_2, resolution: 2.25 Å).

**Fig. 4:**
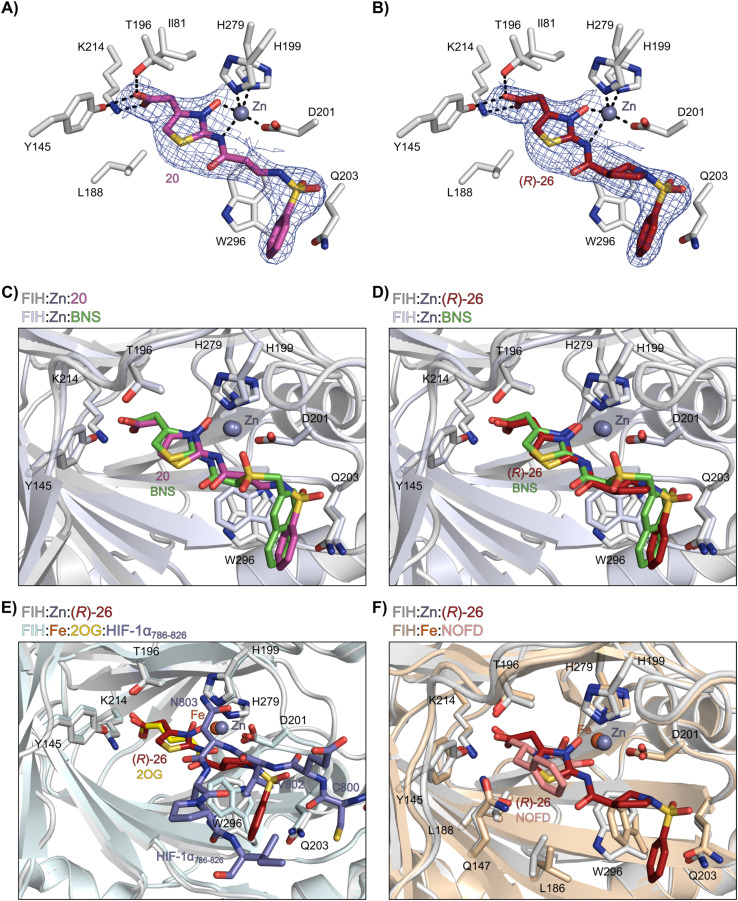
*N*-Hydroxythiazole derivatives 20 and 26 bind to the FIH active site in a similar manner to BNS. Colour code: light grey: FIH; purple: carbon-backbone of 20; ruby: carbon-backbone of (*R*)-26; green: carbon-backbone of BNS; dark grey: zinc; orange: iron; red: oxygen; blue: nitrogen and yellow: sulphur. (A) View from the FIH:Zn:20 complex structure (PDB ID: 8K72) showing the OMIT electron density map (mFo-DFc) contoured to 2.1 σ around 20. (B) View from the FIH:Zn:26 complex structure (PDB ID: 8K73) showing the OMIT electron density map (mFo-DFc) contoured to 2.1 σ around 26. (C) Superimposition of active site views from the FIH:Zn:20 and the FIH:Zn:BNS (PDB ID: 8K71, FIH: light blue) complex structures. (D–F) Superimposition of active site views from the FIH:Zn:26 complex structure and (D) the FIH:Zn:BNS complex structures (PDB ID: 8K71, FIH: light blue), (E) a reported FIH:Fe:2OG:HIF-1α_786–826_ complex structure (PDB ID: 1H2L;^28^ light blue: FIH; yellow: carbon-backbone of 2OG; purple: carbon-backbone of HIF-1α_786–826_), and (F) a reported FIH:Fe:NOFD complex structure (PDB ID: 1YCI;^29^ ochre: FIH; pink: carbon-backbone of NOFD).

Analysis of the FIH:Zn:20 complex structure revealed that 20 adopts an FIH binding pose similar to that of BNS (Fig. 4A and C). Notably, the phenyl ring of 20 did not engage in a π-stacking interaction with the side chain of Trp296, as observed for the naphthalene unit of BNS in complex with FIH, but instead forms a face-to-face amide-π interaction with the primary amide group of Gln203. The reduced potency of FIH inhibitor 20 compared with 4 and BNS (*i.e.* ∼3-fold), may be a result of the lack of interaction with Trp296. Importantly, however, there appeared to be sufficient space adjacent to the ethylene unit of 20 in the FIH:Zn:20 complex structure to potentially allow for its substitution with a carbocycle, which may also interact productively with the indole side chain of Trp296. Thus, *N*-hydroxythiazole derivatives 24–30, in which carbocycles substitute for the ethylene chain of 20, were synthesised to test this proposal (ESI Scheme S5†) and their inhibition of FIH, PHD2, JMJD5, AspH and KDM4A was determined (Table 4).

**Table 4:** Inhibition of human 2OG oxygenases by *N*-hydroxythiazole analogues bearing carbocyclic linkers

Entry	Cmpd^a^	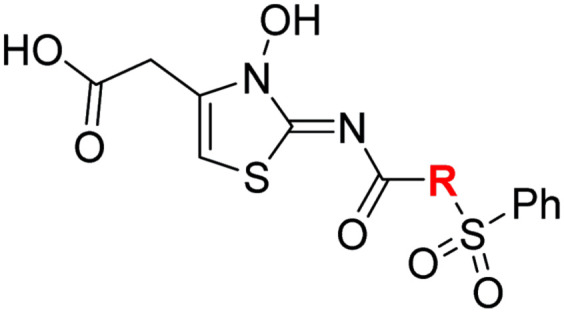	IC_50_ [μM]^b^				
		R	FIH^c^	AspH^d^	PHD2^e^	KDM4A^f^	JMJD5^g^
i	20	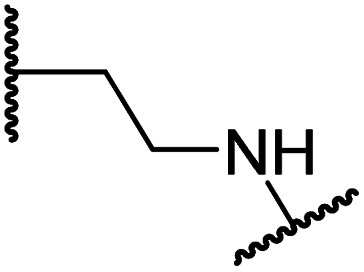	1.0 ± 0.0	23.3 ± 10.1	6.5 ± 1.8	>100	13.4 ± 6.5
ii	24	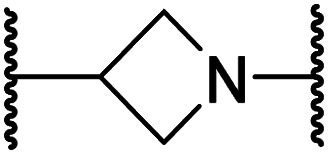	0.45 ± 0.17	60.9 ± 24.1	5.2 ± 0.7	>100	29.1 ± 4.4
iii	25	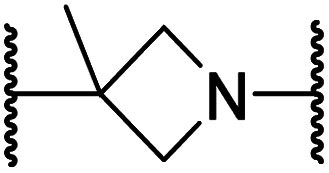	1.2 ± 0.2	>100	33.5 ± 1.0	>100	32.6 ± 6.7
iv	26	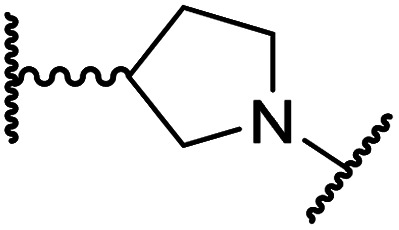	0.50 ± 0.02	37.8 ± 11.3	12.4 ± 0.8	>100	12.5 ± 1.0
v	27	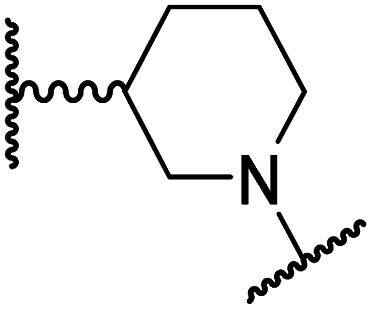	0.87 ± 0.04	49.1 ± 1.7	11.0 ± 1.6	>100	13.4 ± 1.0
vi	28	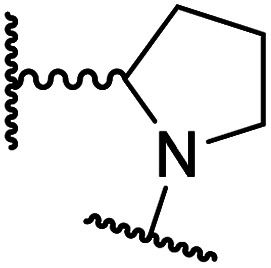	2.1 ± 1.5	24.9 ± 12.0	12.7 ± 1.8	>100	2.6 ± 0.2
vii	29	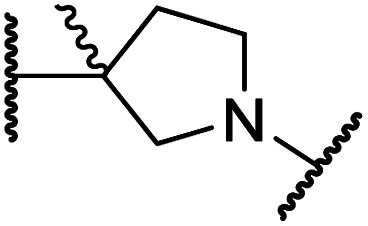	>100	>100	>100	>100	>100
viii	30	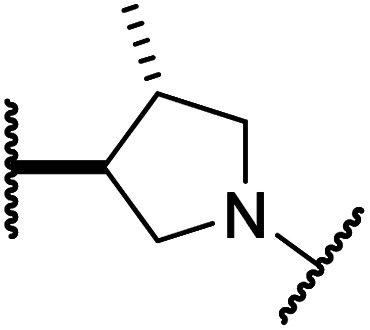	1.7 ± 0.4	>100	71.7 ± 26.6	>100	25.6 ± 0.6

a All chiral *N*-hydroxythiazole derivatives were prepared as racemic mixtures.

b Mean average ± SD of two independent experiments (each composed of technical duplicates).

c Using 0.15 μM FIH, 10.0 μM 2OG and 5.0 μM HIF-1α C-TAD_788–822_.^61^

d Using 0.05 μM His_6_-AspH_315–758_, 3.0 μM 2OG and 1.0 μM hFX-CP_101–119_.^58^

e Using 0.15 μM PHD2_181–426_, 10.0 μM 2OG and 5.0 μM HIF-1α CODD_556–574_.^61^

f Using 0.15 μM KDM4A, 10.0 μM 2OG and 10.0 μM H3_1–15_K9me3_1–15_.^69^

g Using 0.15 μM JMJD5, 2.0 μM 2OG and 2.0 μM RSP6_128–148_.^70^ Inhibition assays were performed using SPE-MS as described in the ESI.

The use of a β-pyrrolidine linker (26; Table 4, entry iv) increased FIH inhibition potency by ∼2-fold relative to 20 (IC_50_: 0.50 μM) and enhanced selectivity for FIH inhibition over PHD2 (∼25-fold), JMJD5 (∼25-fold) and AspH (∼75-fold) inhibition; 26 did not inhibit KDM4A (IC_50_ > 100 μM). The increased rigidity of the pyrrolidine ring of 26, compared with the ethylene unit of 20, is likely responsible for the increased selectivity of 26, by limiting the potential binding modes of the terminal phenyl ring. Decreasing the linker ring size from a β-pyrrolidine to an azetidine (24; Table 4, entry ii) had no effect on FIH inhibition (IC_50_: 0.45 μM); however, the selectivity of compound 24 for FIH over PHD2 inhibition (12-fold) was reduced by ∼2-fold compared to 26. 3-Methylazetidine (25; Table 4, entry iii) and β-piperidine (27; Table 3, entry v) analogues showed decreased FIH inhibition (IC_50_s: 1.2 and 0.87 μM, respectively) relative to 26.

Analysis of a crystal structure of FIH complexed with β-pyrrolidine derivative 26 (FIH:Zn:26, PDB ID: 8K73; space group: *P*4_1_2_1_2, resolution: 2.25 Å) reveals that 26 binds to the FIH active site in a similar manner to ethylamine derivative 20, with its phenyl sulfonamide moiety adopting an almost identical conformation for both compounds (Fig. 4A and B), that is engaging in a face-to-face amide-π stacking interaction with the side chain of Gln203. Notably, the β-pyrrolidine ring of 26 is positioned to form hydrophobic interactions with Trp296, as observed with the naphthalene ring of BNS (Fig. 2B), which likely contributes to the increased FIH inhibition by 26, compared with 20, in addition to the reduced entropic cost of binding. Analysis of the electron density maps for the FIH:Zn:26 structure indicates that only (*R*)-26 binds to FIH, despite 26 being present as a racemic mixture, suggesting that the inhibitory activity of the two 26 enantiomers likely differs. Thus, the structural analyses support our proposal that the observed increase in selectivity for FIH inhibition may be a result of stabilising the conformation that binds to the FIH active site.

A substantial decrease in inhibition potency was observed for the α-pyrrolidine analogue of 26 (*i.e.*28; IC_50_: 2.1 μM, Table 4, entry vi), potentially reflecting a steric clash with the FIH active site, in particular with Trp296, and/or loss of the favourable amide-π stacking interaction between the phenyl ring and Gln203, as observed in the FIH:Zn:26 complex (Fig. 4B), due to the geometry of the α-pyrrolidine ring. These results indicate that a β-pyrrolidine ring, as in 26, is preferred for achieving both FIH inhibition and selectivity over PHD2, JMJD5, AspH and KDM4A. The addition of a methyl substituent at the C3 position of the β-pyrrolidine ring (29; Table 4, entry vii) results in a complete loss of FIH inhibition (IC_50_ > 100 μM), while the *trans*-4-methylpyrrolidine derivative (30; Table 4, entry viii) shows decreased levels of FIH inhibition (IC_50_: 1.7 μM), but improved selectivity for FIH inhibition over PHD2 and AspH relative to 26.

#### Variations on the terminal sulfonamide substituent

**Scheme 2:**
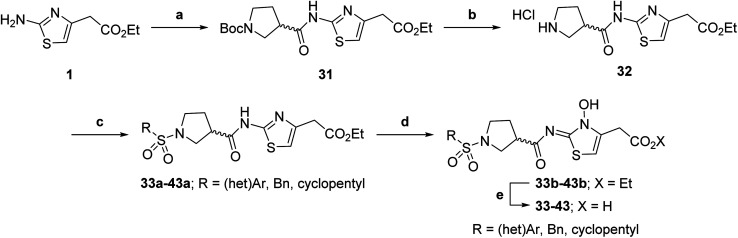
Synthesis of *N*-hydroxythiazole derivatives 33–43.^*a,b a*^(a) *N*-Boc-pyrrolidine-3-carboxylic acid, T3P,^82^*i*Pr_2_NEt, DMF, 0 °C to rt, 99%; (b) HCl/dioxane, 0 °C to rt, 83%; (c) PhSO_2_Cl, Et_3_N, CH_2_Cl_2_, 0 °C to rt, 78–95%; (d) mCPBA, CHCl_3_, rt, 24–67%; (e) LiOH, MeOH/H_2_O, 0 °C to rt, 28–82%. ^*b*^Chemical structures of R groups are shown in Table 5.

Having identified compound 26 as a potent FIH inhibitor that displays >25-fold selectivity with respect to PHD2, JMJD5, AspH and KDM4A inhibition, structurally related *N*-hydroxythiazoles 33-43 were prepared to investigate the effect of modifying the terminal phenyl group on FIH inhibitor potency (Scheme 2).

The introduction of a chlorine substituent at the *ortho*- (33; IC_50_: 0.39 μM), *meta*- (34; IC_50_: 0.44 μM) or *para*-position (35; IC_50_: 0.46 μM) of the phenyl ring of 26 had negligible effects on inhibitor potency (Table 5, entries ii–iv). Similarly, FIH inhibition was not affected by electron donating (36; IC_50_: 0.46 μM) or electron withdrawing (37; IC_50_: 0.47 μM) substituents at the *para*-position of the phenyl ring (Table 5, entries v and vi), although selectivity over JMJD5 was enhanced ∼5-fold for trifluoromethyl-substituted 37, relative to 26. Cyclopentyl derivative 43 manifested reduced levels of FIH inhibition (IC_50_: 0.81 μM, Table 5; entry xii), which might relate to the loss of the amide-π stacking interaction between the phenyl ring of 26 and Gln203, as observed in the FIH:Zn:26 complex structure.

**Table 5:** Inhibition of human 2OG oxygenases by *N*-hydroxythiazole analogues bearing different terminal sulfonamide substituents

Entry	Cmpd^a^	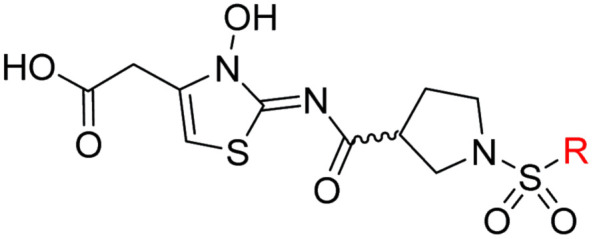	IC_50_ [μM]^b^				
		R	FIH^c^	PHD2^d^	AspH^e^	KDM4A^f^	JMJD5^g^
i	26	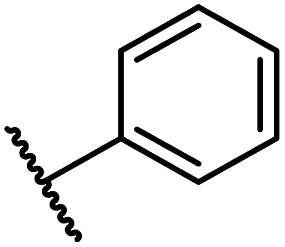	0.45 ± 0.17	12.4 ± 0.8	37.8 ± 11.3	>100	12.5 ± 1.0
ii	33	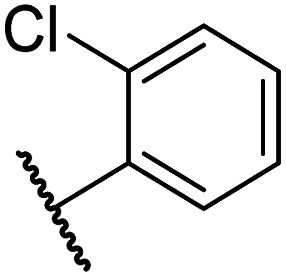	0.39 ± 0.04	8.0 ± 0.2	30.3 ± 12.6	>100	11.9 ± 1.8
iii	34	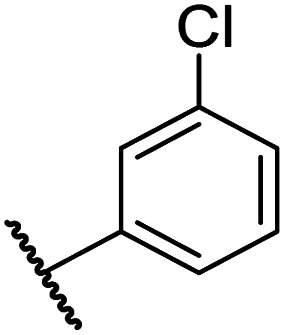	0.44 ± 0.03	14.6 ± 3.2	35.0 ± 0.0	>100	18.5 ± 1.0
iv	35	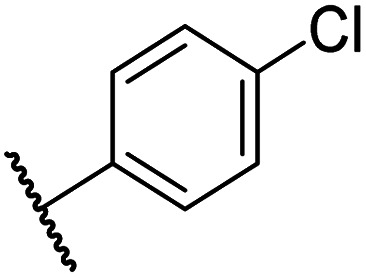	0.46 ± 0.01	9.2 ± 0.4	28.8 ± 8.9	>100	37.7 ± 0.9
v	36	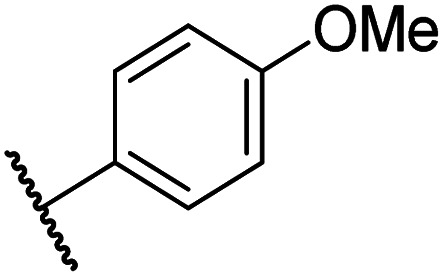	0.46 ± 0.01	9.8 ± 0.5	36.6 ± 2.8	>100	16.0 ± 6.8
vi	37	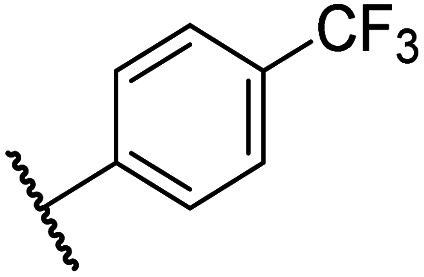	0.47 ± 0.07	15.2 ± 3.6	36.0 ± 0.7	>100	63.2 ± 6.1
vii	38	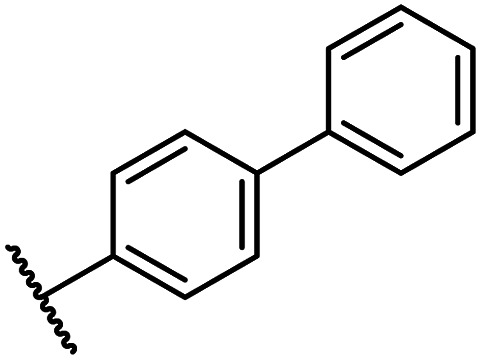	0.26 ± 0.01	3.1 ± 0.1	30.2 ± 10.9	>100	31.9 ± 2.0
viii	39	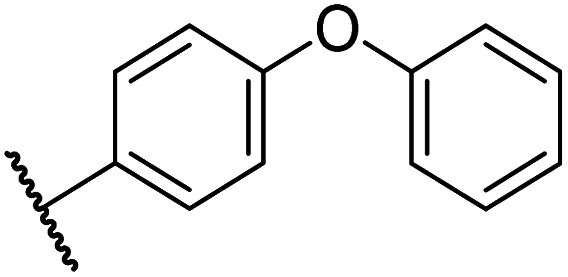	0.29 ± 0.03	3.1 ± 0.2	29.8 ± 4.3	>100	8.6 ± 0.2
ix	40	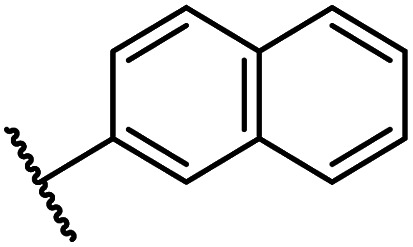	0.45 ± 0.08	5.2 ± 1.1	40.1 ± 2.2	>100	62.8 ± 0.4
x	41	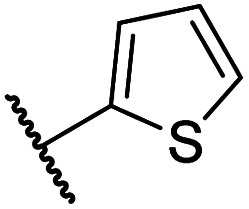	0.47 ± 0.07	11.2 ± 2.3	30.3 ± 8.0	>100	15.4 ± 7.1
xi	42	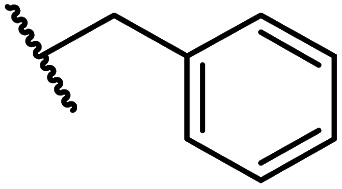	0.28 ± 0.02	6.9 ± 0.5	43.1 ± 18.7	>100	7.2 ± 2.8
xii	43	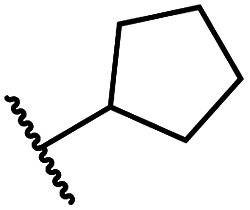	0.81 ± 0.18	9.9 ± 0.5	35.5 ± 3.2	>100	17.4 ± 6.0

a All chiral *N*-hydroxythiazole derivatives were prepared as racemic mixtures.

b Mean average ± SD of two independent experiments (each composed of technical duplicates).

c Using 0.15 μM FIH, 10.0 μM 2OG and 5.0 μM HIF-1α C-TAD_788–822_.^61^

d Using 0.15 μM PHD2_181–426_, 10.0 μM 2OG and 5.0 μM HIF-1α CODD_556–574_.^61^

e Using 0.05 μM His_6_-AspH_315–758_, 3.0 μM 2OG and 1.0 μM hFX-CP_101–119_.^58^

f Using 0.15 μM KDM4A, 10.0 μM 2OG and 10.0 μM H3_1–15_K9me3_1–15_.^69^

g Using 0.15 μM JMJD5, 2.0 μM 2OG and 2.0 μM RSP6_128–148_.^70^ Inhibition assays were performed using SPE-MS as described in the ESI.

The most potent FIH inhibitors identified from this series were biphenyl sulfonamide derivative 38 (IC_50_: 0.26 μM), ether 39 (IC_50_: 0.29 μM), and the benzyl derivative 42 (IC_50_: 0.28 μM) (Table 5; entries vii, viii and xi). These compounds displayed comparable levels of FIH inhibition to the (partially) selective FIH inhibitor NOFD (IC_50_: 0.24 μM) and the broad-spectrum 2OG oxygenase inhibitor NOG (IC_50_: 0.36 μM), and were ∼10-fold more potent than 2,4-PDCA (IC_50_: 5.0 μM)^54^ (Table 6, entries i–iii). Excellent selectivity for FIH inhibition over AspH and KDM4A inhibition was also observed (>100-fold and >300-fold, respectively); 42 manifested ∼25-fold selectivity for FIH inhibition over PHD2 and JMJD5 inhibition.

**Table 6:** Comparison of human 2OG oxygenase inhibition by optimised *N*-hydroxythiazole derivatives and BNS with the reported FIH inhibitors 2,4-PDCA, NOG and NOFD^29^

Entry	Cmpd	IC_50_ [μM]^a^				
		FIH^b^	PHD2^c^	AspH^d^	KDM4A^e^	JMJD5^f^
i	2,4-PDCA	5.0 ± 2.1 (ref. 54)	5.3 ± 3.4 (ref. 54)	0.03 ± 0.01 (ref. 54)	0.10 ± 0.00	0.33 ± 0.07 (ref. 70)
ii	NOG	0.36 ± 0.03	12.3 ± 4.4	1.1 ± 0.3 (ref. 58)	22.1 ± 1.1	0.15 ± 0.02 (ref. 70)
iii	NOFD	0.24 ± 0.02	>100	15.5 ± 1.2 (ref. 60)	14.1 ± 0.0	>100 (ref. 70)
iv	BNS	0.30 ± 0.07	0.11 ± 0.00	3.4 ± 0.1	67.4 ± 39.8	0.25 ± 0.01
v	26	0.45 ± 0.17	12.4 ± 0.8	37.8 ± 11.3	>100	12.5 ± 1.0
vi	38	0.26 ± 0.01	3.1 ± 0.1	30.2 ± 10.9	>100	31.9 ± 2.0
vii	39	0.29 ± 0.03	3.1 ± 0.2	29.8 ± 4.3	>100	8.6 ± 0.2
viii	42	0.28 ± 0.02	6.9 ± 0.5	43.1 ± 18.7	>100	7.2 ± 2.8

a Mean average ± SD of two independent experiments (each composed of technical duplicates).

b Using 0.15 μM FIH, 10.0 μM 2OG and 5.0 μM HIF-1α C-TAD_788–822_.^61^

c Using 0.15 μM PHD2_181–426_, 10.0 μM 2OG and 5.0 μM HIF-1α CODD_556–574_.^61^

d Using 0.05 μM His_6_-AspH_315–758_, 3.0 μM 2OG and 1.0 μM hFX-CP_101–119_.^58^

e Using 0.15 μM KDM4A, 10.0 μM 2OG and 10.0 μM H3_1–15_K9me3_1–15_.^69^

f Using 0.15 μM JMJD5, 2.0 μM 2OG and 2.0 μM RSP6_128–148_.^70^ Inhibition assays were performed using SPE-MS as described in the ESI.

Interestingly, the results indicate that 42 exhibits a different selectivity profile compared to NOFD with respect to the other 2OG oxygenases tested. NOFD displays greater selectivity for inhibition of FIH over PHD2 and JMJD5 (NOFD: >300-fold selective; 42: ∼25-fold selective, as judged by IC_50_ values; Table 6). Whereas, 42 manifests improved selectivity for AspH (NOFD: ∼65-fold selective; 42: >100-fold selective) and KDM4A (NOFD: ∼60-fold selective; 42: >300-fold selective). The difference in 2OG oxygenase selectivity likely reflects the different binding modes of NOFD compared with the *N*-hydroxythiazole-derived FIH inhibitors, as indicated by superimposition of the FIH:Fe:NOFD and FIH:Zn:26 and complex structures (Fig. 4F).

Crystallographic studies have shown that the benzyl side chain of NOFD, which occupies a hydrophobic pocket in the FIH active site (formed by Tyr102, Tyr145, Gln147 and Leu186; ESI Fig. S11†), which has not yet been observed in the active sites of some other 2OG oxygenases, including PHD2, is responsible for the selectivity of NOFD for FIH inhibition over PHD2. In the reported PHD2:Mn:NOG complex structure (PDB ID: 5L9R),^79^ the pro-*R* methylene hydrogen atom of NOG is orientated towards the side chain of Leu343, which would likely clash with the benzyl substituent of NOFD, so preventing efficient binding of NOFD to PHD2. By contrast, we propose that the FIH selectivity of 26 and 42 arises, at least in part, due to the rigidity of the β-pyrrolidine ring and the formation of productive interactions with the side chains of residues involved in FIH substrate recognition, *i.e.* Gln203 and Trp296.

### Cellular studies

#### *N*-Hydroxythiazole-based FIH inhibitors modulate cellular expression of FIH-dependent HIF target genes

At least in some cells, the FIH-catalysed asparagine residue hydroxylation in the C-TAD of HIF-α isoforms inhibits the interaction between HIF-α and the histone acetyl transferases/transcriptional coactivators p300 and CBP, thus resulting in context-dependent suppression of C-TAD-mediated promotion of HIF target gene expression.^17^ The prolyl hydroxylase domain-containing protein 3 gene (*EGLN3*) is a HIF target gene, which is apparently negatively regulated by FIH catalysis, potentially in a context-dependent manner.^17,56^ We therefore investigated the effect of the *N*-hydroxythiazole-based FIH selective inhibitors 38, 39 and 42 (Table 5), as well as the corresponding ethyl ester analogue of 42 (*i.e.*42b), on *EGLN3* expression levels using DM-NOFD^17^ as a positive FIH inhibition control.

**Fig. 5:**
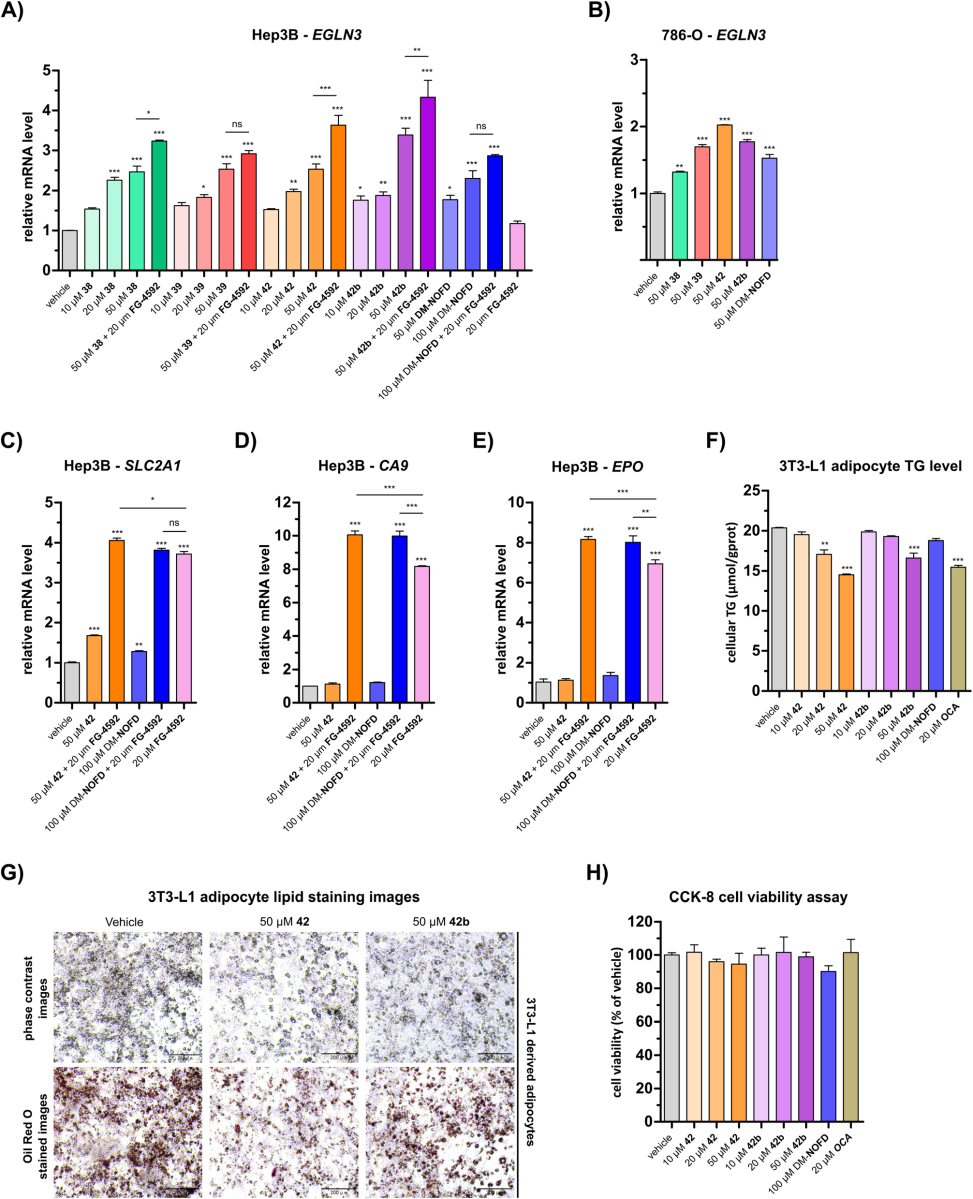
*N*-Hydroxythiazole-based FIH inhibitors modulate HIF target gene expression in cells and reduce adipocyte lipid accumulation. (A and B) Quantitative Real Time PCR (qRT-PCR) analyses showing the effects of *N*-hydroxythiazole-based FIH inhibitors 38, 39, 42 and the ethyl ester prodrug form of 42 (*i.e.*42b), dimethyl *N*-oxalyl-d-phenylalanine (DM-NOFD) and FG-4592 on prolyl hydroxylase domain-containing protein 3 (*EGLN3*) expression levels in (A) Hep3B and (B) 786-O cells relative to a negative inhibition control (DMSO). (C–E) qRT-PCR analyses showing the effects of 42, DM-NOFD, and FG-4592 on (C) solute carrier family 2 member 1 (*SLC2A1*), (D) carbonic anhydrase 9 (*CA9*) and (E) erythropoietin (EPO) expression levels in Hep3B cells relative to negative inhibition control (DMSO). (F) The cellular triglyceride (TG) levels of 3T3-L1 derived adipocytes following treatment with 42, 42b, DM-NOFD, the reported farnesoid X receptor (FXR) agonist obeticholic acid (OCA) and a negative inhibition control (DMSO), as determined by a triglyceride colorimetric assay. (G) Representative images of 3T3-L1 derived adipocytes treated with 42 and 42b, after staining with Oil Red O. Scale bar, 200 μm. (H) The effects of 42, 42b, DM-NOFD and OCA on 3T3-L1 derived adipocyte cell viability, as measured using the CCK-8 cell viability assay.^95^ Values shown are percentages (%) relative to the negative inhibition control (DMSO). Mean average ± SD of three independent experiments are shown. Results were analysed with one-way ANOVA followed by Tukey's multiple comparison test (ns, *p* > 0.05; *, *p* < 0.05; **, *p* < 0.01; ***, *p* < 0.001). Cell-based studies were performed as described in the ESI.†

Quantitative real time PCR (qRT-PCR) analyses revealed a dose-dependent increase in *EGLN3* mRNA levels following the treatment of human hepatocyte carcinoma-derived Hep3B cells with 38, 39, 42, or 42b (Fig. 5A). At 50 μM, carboxylic acids 38, 39 and 42 increased *EGLN3* expression by ∼2.5-fold relative to the negative inhibition control (*i.e.* DMSO). An apparently greater increase (∼3.4-fold) was observed for the ethyl ester 42b at 50 μM. By comparison, use of 50 μM DM-NOFD increased *EGLN3* levels by ∼1.8-fold, indicating the *N*-hydroxythiazole derivatives manifest similar, if not greater, cellular efficacy compared to DM-NOFD (Fig. 5A). Since they also display weak PHD2 inhibition (Table 6), it cannot be ruled out that 38, 39, 42 or 42b cause weak HIF-α upregulation, that, along with FIH inhibition, contributes to the upregulation of *EGLN3* levels observed in their presence. Note, however, that NOFD upregulates *EGLN3* levels and has little, if any, PHD2 inhibition activity (Table 6).

We investigated the effect of 38, 39, 42 and 42b in the presence of the PHD inhibitor FG-4592 (FG-4592, roxadustat),^52^ which stimulates an increase in HIF-α levels, to explore whether the effect on *EGLN3* expression would alter. When co-administered with 38, 42 or 42b, FG-4592 caused an greater increase in *EGLN3* upregulation relative to the effect of 38, 42 or 42b alone (Fig. 5A); FG-4592 alone had no effect on Hep3B *EGLN3* levels. These results support the proposal that FIH catalysis negatively regulates expression of the HIF target gene *EGLN3* through a HIF-dependent mechanism. Interestingly, no additional effect on *EGLN3* levels was observed for FG-4592 with either 39 or DM-NOFD, even at 100 μM DM-NOFD, compared to use of 39 or DM-NOFD alone, an observation requiring further investigation, and one which illustrates the complexity of the biochemistry of HIF transcriptional regulation. We also investigated the effect of the *N*-hydroxythiazole-derived FIH inhibitors 38, 39, 42 and 42b, and DM-NOFD on *EGLN3* gene expression in VHL-deficient renal cell carcinoma-derived 786-O cells. Due to the absence of VHL, PHD-mediated HIF degradation does not operate in 786-O cells leading to elevated HIF levels. As with Hep3B cells, we observed upregulation of *EGLN3* with all compounds tested, including DM-NOFD (Fig. 5B).

The effect of 42 and DM-NOFD on the expression of three additional proposed HIF target genes in Hep3B cells,^17^*i.e.* solute carrier family 2 member 1 (*SLC2A1*; Fig. 5C), carbonic anhydrase 9 (*CA9*; Fig. 5D) and erythropoietin (*EPO*; Fig. 5E), with and without FG-4592, was subsequently investigated by qPCR. *SLC2A1* levels were increased significantly with FG-4592 (∼4-fold), and, to a lesser extent with 42 (∼1.5-fold) and DM-NOFD (∼1.3-fold). *EPO* and *CA9* were also strongly upregulated by FG-4592 (∼7-fold and ∼8-fold, respectively) and showed no response to treatment with either 42 or DM-NOFD. By contrast, an increase in *CA9* and, to a lesser extent, *EPO* levels was observed when 42 and DM-NOFD were administered in combination with FG-4592, compared with when FG-4592 was used alone. By contrast, no synergistic effect was observed for 42 and DM-NOFD in combination with FG-4592 on *SLC2A1* gene expression.

Overall, the cellular results are in agreement with previous work that implying dual inhibition of both FIH and PHDs may be necessary to induce substantial expression of some, but not necessarily all, HIF target genes. Note, it cannot be ruled out that other mechanisms, including inhibition of the PHDs or other 2OG oxygenases potentially involved in HIF target gene expression by 42 or DM-NOFD, or (*e.g.*) inhibitor mediated effects on the location or the availability of HIF-α isoforms, may contribute towards the observed effects.

#### FIH inhibitors reduce lipid accumulation in 3T3-L1-derived adipocytes

Genetic knockout of FIH is reported to stimulate a shift in cellular metabolism that, unusually, causes an increase in both glycolysis and oxidative metabolism, leading to increased energy consumption.^48^ To inform on the proposed role of FIH in metabolic regulation, we investigated the effects of 42, 42b and DM-NOFD on lipid accumulation in 3T3-L1-derived adipocytes. Following treatment with 42 and 42b, a dose-dependent decrease in cellular triglyceride (TG) levels was observed, as determined using a triglyceride colorimetric assay (Fig. 5F). By contrast, DM-NOFD had no effect on TG levels at 100 μM. At 50 μM, 42 and 42b caused a ∼30% and ∼20% reduction in cellular TG levels, respectively, which was comparable to the effect of treatment with 20 μM obeticholic acid (25% reduction; OCA), a farnesoid X receptor (FXR) agonist that is used to treat metabolic-related conditions including primary biliary cirrhosis and non-alcoholic fatty liver disease.^94^ The effect of 42 and 42b on lipid accumulation could also be observed using Oil Red O staining (Fig. 5G). Neither 42 and 42b exhibited apparent cytotoxicity at the concentrations used (Fig. 5H). Although further investigation is necessary, our observations are consistent with the involvement of FIH in the regulation of cellular metabolism.

## Conclusions

The physiological roles of FIH are incompletely understood, including how its substrate and, potentially, its 2-oxo acid cosubstrate^60^ promiscuity relate to its role in hypoxia sensing. Potent and selective small-molecule FIH inhibitors will be useful for *in vivo* functional assignment studies. Reported studies on the cellular roles of FIH have employed a prodrug diester form of the small-molecule NOFD,^17,29^ which is a derivative of the broad-spectrum 2OG oxygenase inhibitor NOG. However, we have previously shown that NOFD inhibits other 2OG oxygenases than FIH, including AspH,^58^ albeit substantially less efficiently. Nonetheless, this observation indicates that NOFD may not be perfectly selective for FIH inhibition considering that ∼60–70 2OG oxygenases are present in humans.^96^ Thus, we were interested in identifying alternative scaffolds suitable for optimisation as *in vivo* active FIH inhibitors.

We investigated reported PHD inhibitors for FIH inhibition on the basis that PHD inhibitors such as daprodustat are reported to inhibit both PHDs and FIH,^56^ to identify novel lead structures for the development of potent and selective FIH inhibitors (ESI Table S1†). Interestingly, we observed that the reported *N*-hydroxythiazole-based PHD inhibitor BNS^33^ inhibits other 2OG oxygenases than the PHDs, including FIH, and can thus be considered as a relatively broad-spectrum 2OG oxygenase inhibitor (Table 1, entry iii). This observation suggests that previous cellular studies performed with BNS(-derivatives) should be interpreted with care,^17,80^ including with respect to potential toxicity.

*N*-Hydroxythiazoles are chemically interesting metalloenzyme inhibitors because of the heavily functionalised and polar nature of the core heterocycle. Thus, BNS was an attractive lead structure for the development of selective FIH inhibitors, *inter alia* because its scaffold can be modified at multiple positions. Rigidification of the BNS structure and replacement of its sulfone and naphthalene groups afforded *N*-hydroxythiazoles that display potent FIH inhibition (IC_50_ < 0.3 μM) and a substantially improved selectivity for FIH inhibition over PHD2 compared with BNS (∼25-fold for 42*vs.* ∼0.4-fold for BNS). High levels of selectivity were also achieved over JMJD5 (∼25-fold), KDM4A (>300-fold) and AspH (∼100-fold), the latter being of interest as AspH catalyses, like FIH, the β-hydroxylation of asparaginyl residues.^71,72^

Importantly, MS studies with isolated recombinant human 2OG oxygenases reveal that the *N*-hydroxythiazole-based FIH inhibitors show a different selectivity profile for inhibiting 2OG oxygenases than does NOFD (Table 6),^77^ an observation that reflects the differences in their FIH binding modes, as observed in co-crystal structures with FIH (Fig. 2 and 4). Analysis of the FIH:Zn:26 complex structure suggests that the FIH inhibition selectivity of 26, and by implication 42, is as a result, at least in part, of interactions formed between 26 and residues involved in FIH substrate recognition, *i.e.* Gln203 and Tyr296. By contrast, NOFD achieves selectivity for FIH through its benzyl side chain, which occupies a hydrophobic pocket formed by FIH residues Tyr102, Tyr145, Gln147 and Leu186 (ESI Fig. S11†).^29^ This observation implies there are, at least, two complementary strategies that can be employed to achieve selectivity for FIH inhibition over other 2OG oxygenases.

The *N*-hydroxythiazole-based FIH inhibitors have potential to help identify the physiologically relevant phenotypes of FIH inhibition, together with other inhibitors such as DM-NOFD. We showed that the FIH inhibitors 38, 39 and 42, and the ethyl ester prodrug form of 42 (*i.e.*42b) induce a dose-dependent increase in *EGLN3* expression, a reported FIH-dependent HIF target gene,^17,56^ which was comparable to that induced by DM-NOFD, the dimethyl ester prodrug of NOFD (Fig. 5A and B). It also appeared that both 42 and 42b reduced lipid accumulation in 3T3-L1 derived adipocytes (Fig. 5F and G), an observation which is consistent with the proposed function of FIH as a regulator of cellular metabolism.^48^ The combined results thus indicate that the *N*-hydroxythiazole-based FIH inhibitors have potential for enabling cell-based and *in vivo* studies directed at investigating the potential therapeutic benefit of FIH inhibition. However, given the complexity of HIF and FIH biochemistry in cells, it cannot be ruled out that other mechanisms contribute to the biologically observed effects.

Crystallographic analyses in combination with docking studies guided compound design and will be valuable for future FIH inhibitor development programs. The FIH:Zn:*N*-hydroxythiazole complex structures indicate that the *N*-hydroxythiazole-based inhibitors bind at the FIH active site *via* competitive displacement of 2OG and its HIF-α substrate. The limited inhibitory activity of truncated *N*-hydroxythiazole derivative 14 suggests that the formation of productive interactions with the HIF-1α substrate binding site, for example with the side chains of Gln203 and Trp296, as observed in the FIH:Zn:26 structure, is important for efficient FIH inhibition (Fig. 4B). Efficient binding to residues that engage the HIF-1α substrate may be responsible for the increased FIH potency of *N*-hydroxythiazoles compared with other PHD inhibitors, such as daprodustat, that appear to bind FIH in a similar manner.^56^

Given the modular structure and broad-spectrum 2OG oxygenase inhibitory activity of BNS, it is likely that the *N*-hydroxythiazole scaffold can be modified to generate selective inhibitors of other 2OG oxygenases including PHD2, JMJD5 or, at least some, JmjC KDMs. The latter is of interest because of potential roles for JmjC KDMs in the hypoxic response.^97^ Note that the addition of substituents to the broad-spectrum 2OG oxygenase inhibitors NOG and 2,4-PDCA has resulted in development of inhibitors of FIH^29^ and JMJD5,^98^ respectively, which display an improved selectivity profile with respect to the parent compounds.

The FIH:Zn:*N*-hydroxythiazole complex structures also showed that *N*-hydroxythiazoles can bind to the active site metal of FIH in a bidentate manner through the exocyclic nitrogen atom and nucleophilic hydroxyl group of the *N*-hydroxythiazole unit. Although previous studies have demonstrated the promising biological activity of *N*-hydroxythiazole-derived compounds, in addition to PHD2 inhibition,^33^ for example antibacterial properties^99^ and inhibition of metallo-β-lactamases (MBLs),^81^ their ability to bind metals in this manner had not yet been observed in crystallographic studies. It is likely that this metal coordination motif may be useful in the development of inhibitors for other metal-containing enzymes, including for 2OG oxygenases and beyond.

## Abbreviations

FIH: Factor inhibiting hypoxia-inducible factor-α
HIF: Hypoxia-inducible factor
2OG: 2-Oxoglutarate
PHD: Prolyl hydroxylase domain-containing protein
AspH: Aspartate/asparagine-β-hydroxylase
KDM: *N* ^ε^-Lysine demethylase
C-TAD: C-Terminal activation domain
NOG: *N*-Oxalylglycine
EPO: Erythropoietin
PDCA: Pyridine-2,4-dicarboxylic acid
(DM)-NOFD: (Dimethyl) *N*-oxalyl-d-phenylalanine

## Data availability

The crystal structure data for the FIH:Zn:BNS, FIH:Zn:20 and FIH:Zn:26 complex structures have been deposited in the protein data bank under PDB accession codes: 8K71 (FIH:Zn:BNS), 8K72 (FIH:Zn:20), and 8K73 (FIH:Zn:26).

## Author contributions

T. P. C., and R. Z. R. T. synthesised the inhibitors, with assistance from J. H. M. T. P. C. carried out modelling studies/analysis of crystal structures. T. P. C., A. T., and L. B. performed the SPE-MS assays; T. P. C., R. Z. R. T., and W. F. performed the FIH:*N*-hydroxythiazole co-crystallizations. Y. N. solved and refined the FIH:*N*-hydroxythiazole complex structures. E. S, L. B., G. F., and A. B. produced and purified recombinant proteins. Y. W. performed the cell-based experiments. L. B., X. Z., and C. J. S. supervised the research. T. P. C. (original draft), L. B., and C. J. S. wrote the manuscript.

## Conflicts of interest

The authors declare no competing interests.

## Supplementary Material

SC-014-D3SC04253G-s001
