# Supplementary material for: Structure-guided optimisation of N-hydroxythiazole-derived inhibitors of factor inhibiting hypoxia-inducible factor-α
Source: Chem Sci. 2023 Oct 27;14(43):12098–120. doi: 10.1039/d3sc04253g (PMC10631261; doi:10.1039/d3sc04253g)
Supplement: SC-014-D3SC04253G-s001 [file SC-014-D3SC04253G-s001.pdf]

Electronic Supplementary Material (ESI) for Chemical Science.
This journal is © The Royal Society of Chemistry 2023

Supporting Information

Structure-guided optimisation of *N*-hydroxythiazole-derived inhibitors of Factor Inhibiting Hypoxia-Inducible Factor-α (FIH)

Thomas P. Corner[1], Ryan Z. R. Teo[1], Yue Wu[2], Eidarus Salah[1], Yu Nakashima[3], Giorgia Fiorini[1], Anthony Tumber[1], Amelia Brasnett[1], James P. Holt-Martyn[1], William D. Figg Jr.[1], Xiaojin Zhang[2,*], Lennart Brewitz[1,*], and Christopher J. Schofield[1,*]

Author affiliations

[1]Chemistry Research Laboratory, Department of Chemistry and the Ineos Oxford Institute for Antimicrobial Resistance, University of Oxford, 12 Mansfield Road, OX1 3TA, Oxford, United Kingdom.

[2]State Key Laboratory of Natural Medicines, Jiangsu Key Laboratory of Drug Design and Optimization and Department of Chemistry, China Pharmaceutical University, Nanjing 211198, China.

[3]Institute of Natural Medicine, University of Toyama, 2630-Sugitani, 930-0194, Toyama, Japan.

*Email: lennart.brewitz@chem.ox.ac.uk, zxj@cpu.edu.cn, or christopher.schofield@chem.ox.ac.uk

Table of contents

1. Supporting figures 3

2. Supporting synthetic schemes 20

3. Biochemical procedures 28

Protein production and purification 28

FIH, PHD2, AspH, JMJD5 and KDM4A inhibition assays 28

Crystallography 28

4. Methods for cell studies 29

Cell culture 29

Quantitative PCR Analysis of Gene Expression 29

3T3-L1 derived adipocytes. 29

Viability Assay for 3T3-L1 derived adipocytes. 29

Cellular TG level assay for 3T3-L1 derived adipocytes. 30

Oil Red O staining for 3T3-L1 derived adipocytes. 30

5. Computational methods 31

General protein structure preparation procedure 31

Gold docking procedure 31

6. Supporting tables 32

7. General synthesis information 34

8. General synthetic procedures 35

9. Synthetic procedures and compound characterisations 36

10. ^{1}H and ^{13}C NMR spectra of novel compounds prepared for this study 81

11. References 200

1. Supporting figures

Supporting Figure S1. Representative dose-response curves used to determine IC_{50} values for the inhibition of FIH by *N*-hydroxythiazole derivatives (continues on the following page). SPE-MS inhibition assays were performed as described in Section 3 of the Supporting Information using recombinant FIH (0.15 µM), 2OG (10 µM), Fe(II) (10 µM), LAA (100 µM), and HIF-1α C-terminal transactivation domain fragment (HIF-1α C-$TAD_{788-812}$; 5 µM).[1, 2] Dose-response curves are a mean of two technical duplicates (n = 2; mean ± standard deviation, SD). The mean of two independent duplicates each composed of technical duplicates was used to determine IC_{50} values.

A) **BNS**[3, 4]: pink circles, **Desidustat**[5]: green squares, **TP0463518**[6]: blue triangles, **GSK360A**[7]: purple inverse triangles, **JNJ-42041935**[8]: magenta diamonds, **Enarodustat**[9]: orange hexagons, **MK-8617**[10]: beige half-filled circles.

B) **4**: pink circles, **5**: green squares, **6**: blue triangles, **7**: purple inverse triangles, **8**: magenta diamonds, **9**: orange hexagons, **3**: beige half-filled circles.

C) **10**: pink circles, **11**: green squares, **12**: blue triangles, **13**: purple inverse triangles, **14**: magenta diamonds, **15**: orange hexagons, **16**: beige half-filled circles.

D) **17**: pink circles, **18**: green squares, **19**: blue triangles, **20**: purple inverse triangles, **21**: magenta diamonds, **22**: orange hexagons, **23**: beige half-filled circles.

E) **24**: pink circles, **25**: green squares, **26**: blue triangles, **27**: purple inverse triangles, **28**: magenta diamonds, **29**: orange hexagons, **30**: beige half-filled circles.

F) **33**: pink circles, **34**: green squares, **35**: blue triangles, **36**: purple inverse triangles, **37**: magenta diamonds, **38**: orange hexagons, **39**: beige half-filled circles.

G) **40**: pink circles, **41**: green squares, **42**: blue triangles, **43**: purple inverse triangles, **2,4-PDCA**: magenta diamonds, **NOG**: orange hexagons, **NOFD**[11]: beige half-filled circles.

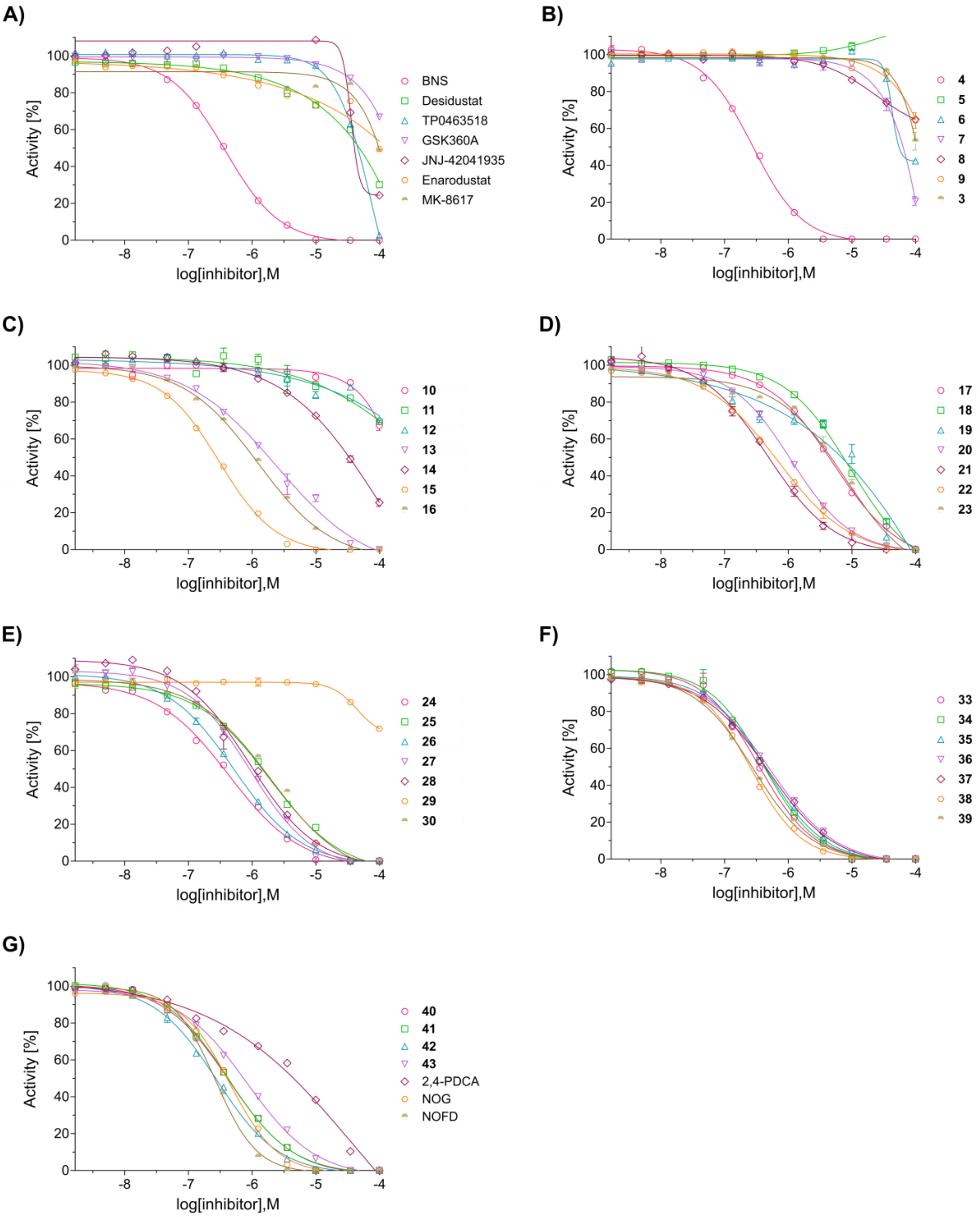
A)
B)
C)
D)
E)
F)
G)
Activity [%]
log[inhibitor],M
BNS
Desidustat
TP0463518
GSK360A
JNJ-42041935
Enarodustat
MK-8617
4
5
6
7
8
9
3
10
11
12
13
14
15
16
17
18
19
20
21
22
23
24
25
26
27
28
29
30
33
34
35
36
37
38
39
40
41
42
43
2,4-PDCA
NOG
NOFD

Supporting Figure S2. Robustness of the FIH SPE-MS inhibition assays. (**A**) Z'-factors[12] and (**B**) signal-to-noise (S/N) ratios for the FIH inhibition assay plates analysed to determine IC_{50} values. The Z'-factors >0.5 (grey line) indicate a stable and robust assay of high quality. Z'-factors and S/N ratios were determined as reported using Microsoft Excel.[12]

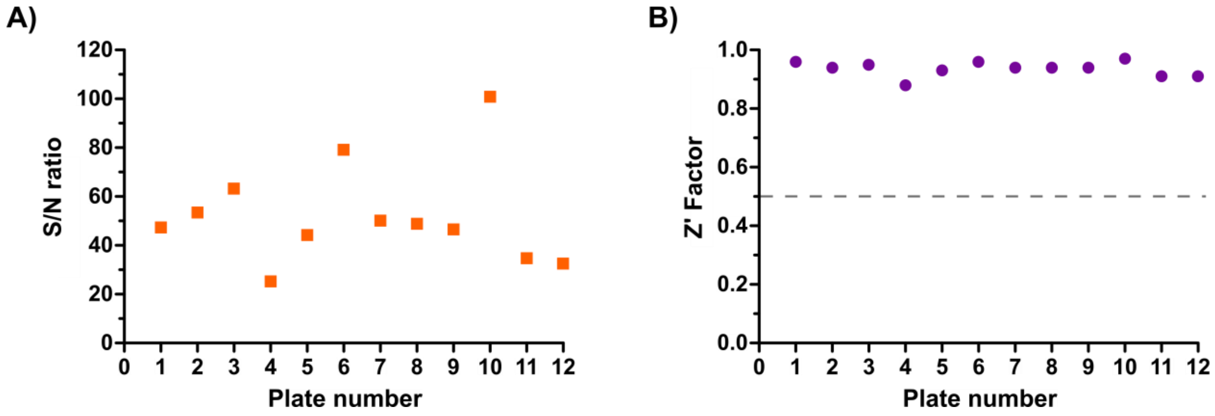

Supporting Figure S3. Homodimerization and overall fold architecture of the FIH:Zn:BNS complex structure. Colours: green: carbon-backbone of BNS[3, 4]; grey: Zn; red: oxygen; blue: nitrogen; gold: sulphur.

A) View from the FIH:Zn:BNS complex crystal structure (PDB ID: 8K71) showing the homodimerization of FIH, as has been observed in solution and previous FIH crystal structures, and which is functionally relevant.[13-15] The two monomers of FIH (red: monomer A; light blue: monomer B) in the FIH dimer are associated at a dimerization interface formed of FIH α-helices α6-α8. [13-15]

B) Views from the FIH:Zn:BNS complex crystal structure (PDB ID: 8K71) show the secondary structure elements of FIH (red: α-helices; yellow: β-sheets). The active site of FIH is composed of a core double-stranded β-helix (DSBH) fold containing eight β-strands (β7–β14),[13-15] which is highly conserved across the 2OG oxygenase enzyme family.[16, 17]

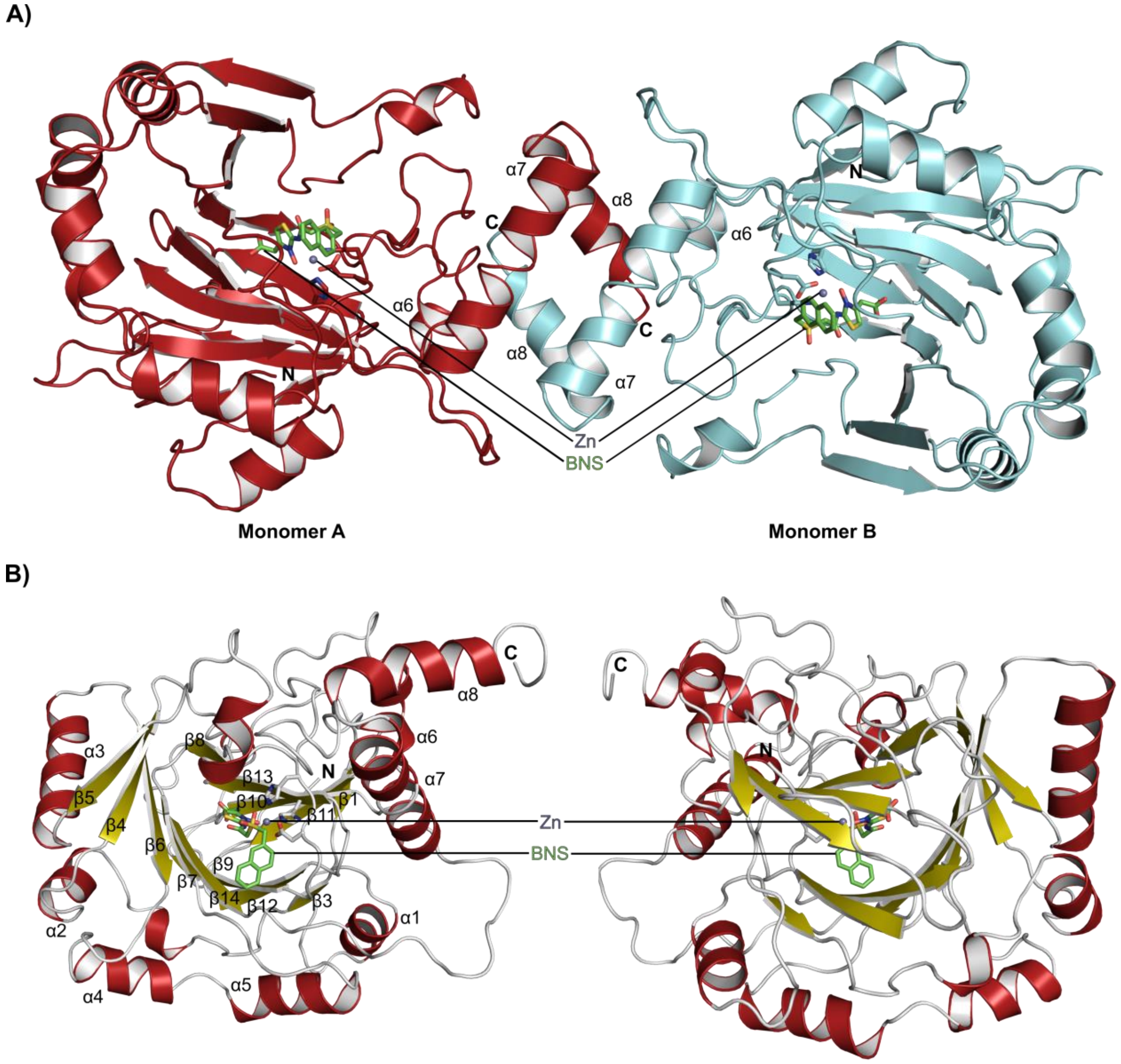

Supporting Figure S4. FIH has a very similar fold in the FIH:Zn:BNS complex structure as in reported FIH:Fe:2OG and FIH:Fe:2OG:HIF-1$\alpha_{786\text{-}826}$ complex structures. Colours: green: carbon-backbone of BNS[3, 4]; yellow: carbon-backbone of 2OG; purple: carbon-backbone of HIF-1$\alpha_{786\text{-}826}$; yellow: carbon-backbone of 2OG; orange: Fe; grey: Zn; red: oxygen; blue: nitrogen; gold: sulphur.

A) Superimposition of views from the FIH:Zn:BNS (grey: FIH; PDB ID: 8K71) and a reported FIH:Fe:2OG (light blue: FIH; PDB ID: 1MZF[14]) complex structures reveals that the overall fold of FIH in both crystal structures is very similar (Cα RMSD = 0.304 Å).

B) Superimposition of views from the FIH:Zn:BNS (grey: FIH; PDB ID: 8K71) and a reported FIH:Fe:2OG: HIF-1$\alpha_{786\text{-}826}$ (ochre: FIH; PDB ID: 1H2L[15]) complex structures reveals that the overall fold of FIH in both crystal structures is very similar (Cα RMSD = 0.281 Å).

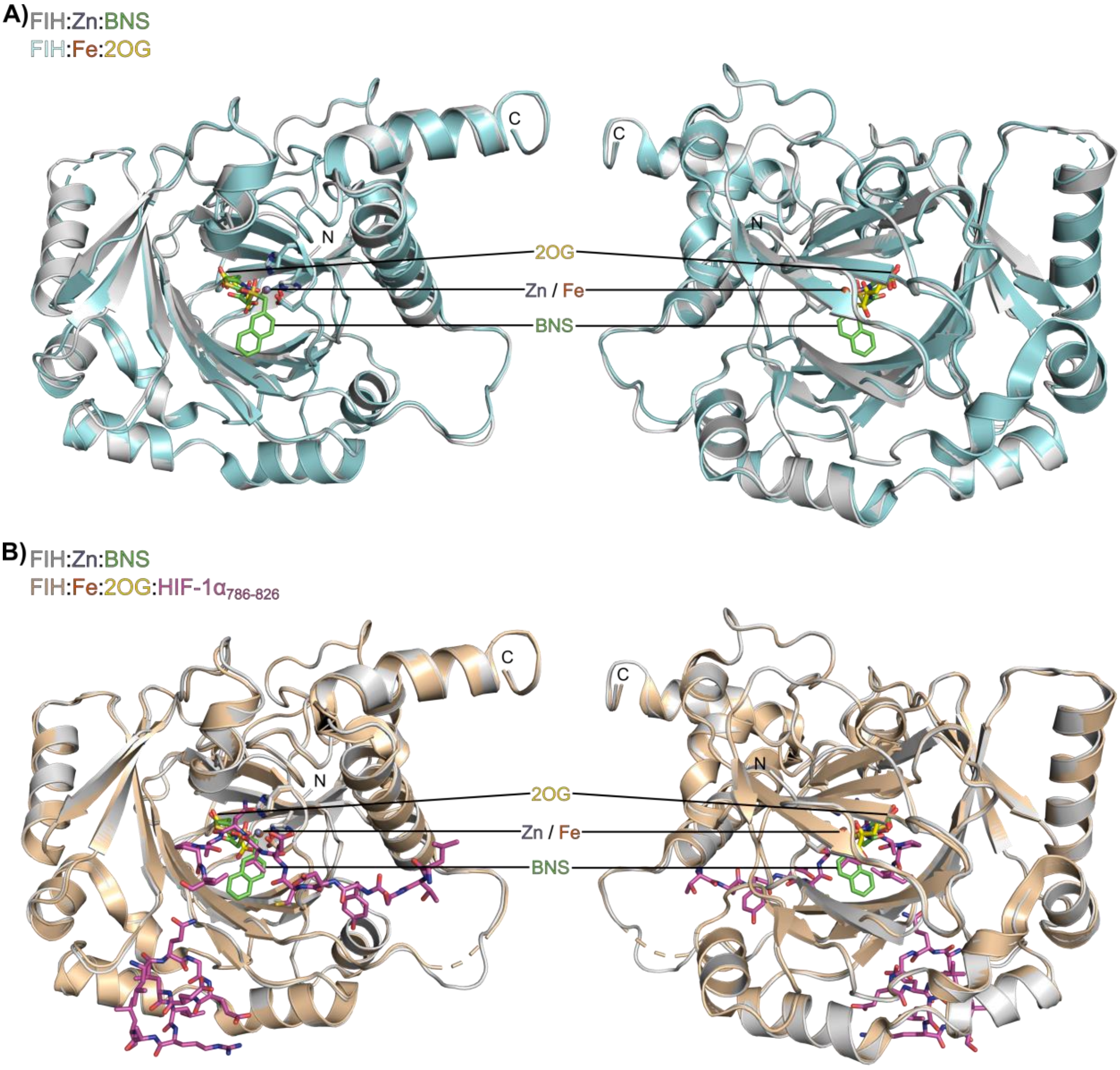

Supporting Figure S5. Comparison of vadadustat and BNS binding to FIH. Colours: green: carbon-backbone of BNS[3, 4]; purple: carbon-backbone of vadadustat[18]; yellow: carbon-backbone of 2OG; violet: carbon-backbone of HIF-1α$_{786\text{-}826}$; orange: Fe; grey: Zn; red: oxygen; blue: nitrogen; gold: sulphur.

A) Superimposition of views from the FIH:Zn:BNS complex structure (grey: FIH; PDB ID: 8K71) and a reported FIH:Zn:vadadustat complex structure (ochre: FIH; PDB: 5OPC[19]) reveals that BNS and vadadustat bind to FIH in a similar manner via bidentate coordination to Zn(II). The orientations of the metal coordination modes are very similar, with one coordinating group binding *trans* to Asp201 and the other coordinating group binding *trans* to His279. Note, however, that the carboxylate of BNS is positioned to interact with the side chains of Tyr145, Thr196 and Lys214, whereas the carboxylate of vadadustat is orientated away from the side chains of Tyr145 and Thr196, and is positioned to interact with the side chain of Lys214 only.

B) Superimposition of views from a reported FIH:Zn:vadadustat complex structure (ochre: FIH; PDB: 5OPC[19]) and a FIH:Fe:2OG:HIF-1α$_{786\text{-}826}$ complex structure (light green: FIH; PDB ID: 1H2L[15]) indicates that vadadustat competes with 2OG for binding to FIH. Note, however, that the metal coordination mode of vadadustat is different to that of 2OG. The amide carbonyl group of vadadustat is predicted to coordinate the active site metal in the same position as the 2OG ketone carbonyl (*i.e. trans* to Asp201), but the predicted orientation of the metal coordination mode of the pyridine nitrogen atom is perpendicular (*i.e. trans* to His279) to that observed for the C1 carboxylate of 2OG (*i.e. trans* to His199). The 3-chlorophenyl group of vadadustat is observed to extend into the HIF-1α substrate binding pocket, an observation which implies vadadustat will impair binding of the HIF-α substrates to FIH.

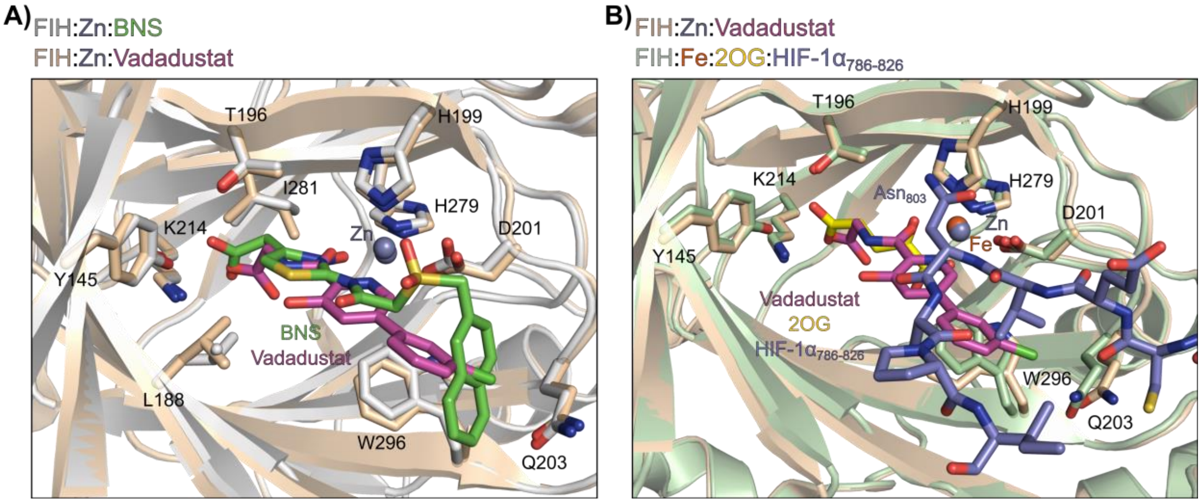

Supporting Figure S6. The different geometry of the KDM4A active site compared to that of FIH is predicted to prevent efficient binding of BNS to KDM4A. Colours: grey: FIH; salmon pink: KDM4A; green: carbon-backbone of BNS; yellow: carbon-backbone of 2OG; dark grey: Zn; lime green: Ni; red: oxygen; blue: nitrogen; gold: sulphur. w: water.

A and **B**) Superimposition of views from the FIH:Zn:BNS (PDB ID: 8K71) and a reported KDM4A:Ni:2OG (PDB ID: 5TVR[20]) complex structures indicates that the naphthalene group of BNS would likely clash with KDM4A residues Thr289, Asn290 and Phe291, which form part of β-strand VIII of the rigid β-barrel core fold of the KDM4A active site, therefore preventing efficient binding of BNS to KDM4A.

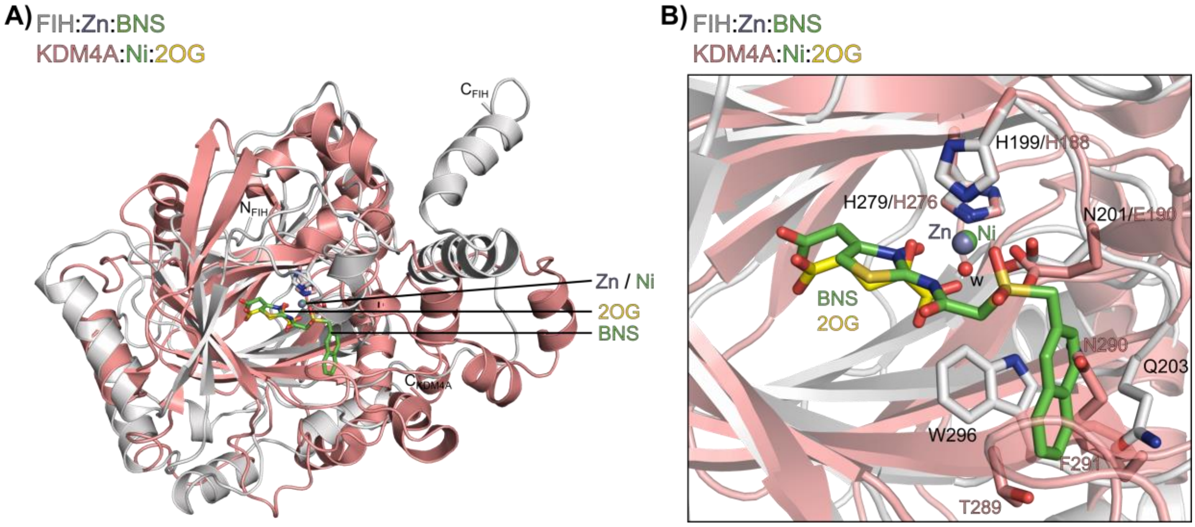

Supporting Figure S7. Docking studies predict that BNS likely binds to the active site of PHD2 in a 2OG-competing manner (continues on the following page). Colours: grey: $PHD2_{181-426}$; green: carbon-backbone of BNS[3, 4]; yellow: carbon-backbone of 2OG; light blue: carbon-backbone of HIF-$1\alpha_{556-574}$; orange: Fe; purple: Mn; red: oxygen; blue: nitrogen; gold: sulphur. w: water. Molecular docking studies were performed using GOLD 5.1[21] and a reported $PHD2_{181-426}$ (PDB ID: 4BQY[22]) crystal structure, as described in Section 5 of the Supporting Information.

A) View of BNS docked into $PHD2_{181-426}$. BNS is predicted to bind at the active site of PHD2 and coordinate to the catalytic Fe(II) ion of PHD2 in a bidentate manner through its *N*-hydroxyl group and the *exo*-nitrogen atom of its *N*-hydroxythiazole unit. The terminal carboxylate of BNS is predicted to be positioned to interact with the side chains of Arg383 and Tyr329 and the sulfone is predicted to form hydrogen bonds with the side chain of Arg322.

B) View from a $PHD2_{181-426}$:Mn:2OG complex structure (PDB ID: 6YW1[23]). Comparison of (**A**) and (**B**) indicates that BNS is predicted to compete with 2OG for binding to PHD2, as indicated by previous *N*-hydroxythiazole mechanistic and computational studies.[3, 24] The carboxylate group of BNS is predicted to interact with PHD2 in a similar manner to the C5 carboxylate of 2OG.

C and **D**) Superimposition of views from the $PHD2_{181-426}$:BNS docking prediction and a reported $PHD2_{181-426}$:Mn:2OG:HIF-$1\alpha_{556-574}$ complex structure (light pink: $PHD2_{181-426}$; PDB ID: 5L9B[25]) indicates that the naphthalene group of BNS is predicted to extend into the HIF-$1\alpha_{556-574}$ substrate binding pocket. The sidechain of PHD2 residue Arg322, which is predicted to form a hydrogen bond interaction with the sulfone of BNS, is positioned to interact with the backbone carbonyl of HIF-$1\alpha_{556-574}$ Pro564 (*i.e.* the HIF-1α Pro-residue that undergoes PHD2-catalysed C5 hydroxylation). The superimposition therefore indicates that BNS will likely impair binding of the HIF-α substrates to PHD2, in addition to competing with 2OG.

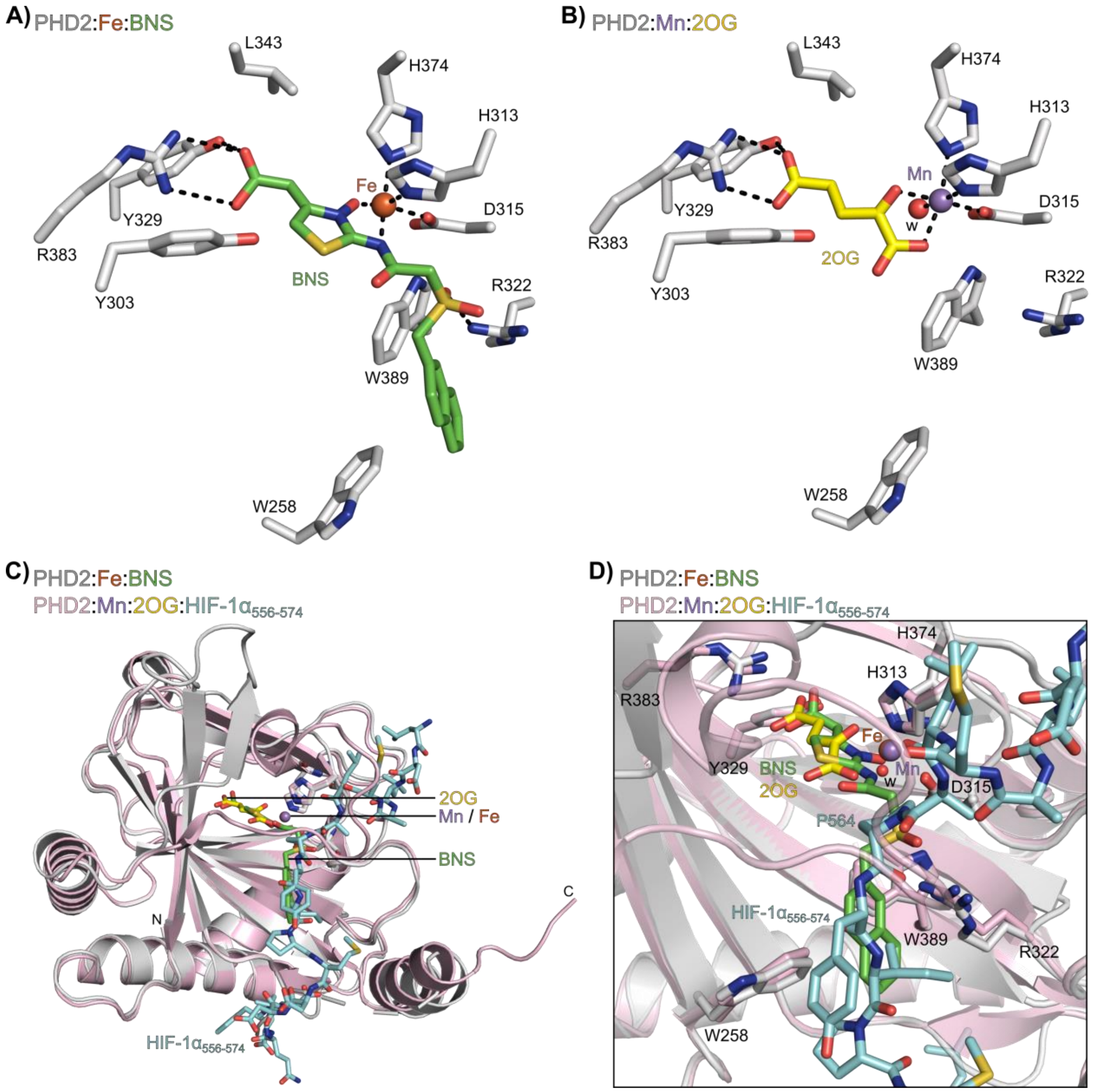

A) PHD2:Fe:BNS
L343
H374
H313
Fe
D315
Y329
R383
Y303
BNS
R322
W389
W258
B) PHD2:Mn:2OG
L343
H374
H313
Mn
D315
Y329
R383
w
2OG
Y303
R322
W389
W258
C) PHD2:Fe:BNS
PHD2:Mn:2OG:HIF-1α556-574
2OG
Mn / Fe
BNS
N
C
HIF-1α556-574
D) PHD2:Fe:BNS
PHD2:Mn:2OG:HIF-1α556-574
H374
H313
R383
Fe
Y329
BNS
2OG
Mn
w
D315
P564
HIF-1α556-574
W389
R322
W258

Supporting Figure S8. Docking studies predict that BNS likely binds to the active site of AspH in a 2OG-competing manner (continues on the following page). Colours: grey: $AspH_{315-758}$; green: carbon-backbone of BNS[3, 4]; yellow: carbon-backbone of 2OG; light blue: carbon-backbone of hFX-$EGFD_{186-224}$; orange: Fe; purple: Mn; red: oxygen; blue: nitrogen; gold: sulphur. w: water. Molecular docking studies were performed using GOLD 5.1[21] and the reported $AspH_{315-758}$ (PDB IDs: 6YYX, 6YYU[26]) crystal structures, as described in Section 5 of the Supporting Information.

A) View of BNS docked into $AspH_{315-758}$. BNS is predicted to bind at the active site of AspH and coordinate to the catalytic Fe(II) ion of AspH in a bidentate manner through its *N*-hydroxyl group and the *exo*-nitrogen atom of its *N*-hydroxythiazole unit. The terminal carboxylate of BNS is predicted to be positioned to interact with the side chains of Arg735 and Ser668 and the sulfone is predicted to form hydrogen bonds with the side chains of Lys666, Arg686 and Arg688.

B) View from an $AspH_{316-758}$:Mn:2OG complex structure (PDB ID: 6YYU[26]). Comparison of (**A**) and (**B**) indicates that BNS is predicted to compete with 2OG for binding to AspH and that the carboxylate group of BNS may interact with AspH in a similar manner as the C5 carboxylate group of 2OG. Note, however, that the *N*-hydroxyl group of BNS is predicted to coordinate Fe(II) in the same position as the 2OG ketone carbonyl, as observed by crystallography, but the predicted orientation of the metal coordination mode of the exo-nitrogen atom is perpendicular (*i.e. trans* to His725) to that observed for the C1 carboxylate of 2OG (*i.e. trans* to His679).

C and **D**) Superimposition of views from the $AspH_{315-758}$:BNS docking prediction and a reported $AspH_{315-758}$:Mn:2OG:hFX-$EGFD_{186-224}$ complex structure (ochre: $AspH_{315-758}$; PDB ID: 6YYW[26]) indicates that the naphthalene group of BNS is predicted to extend into the hFX-$EGFD_{186-224}$ substrate binding pocket. The superimposition therefore indicates that BNS will likely impair binding of the hFX-EGFD substrate to AspH, in addition to competing with 2OG.

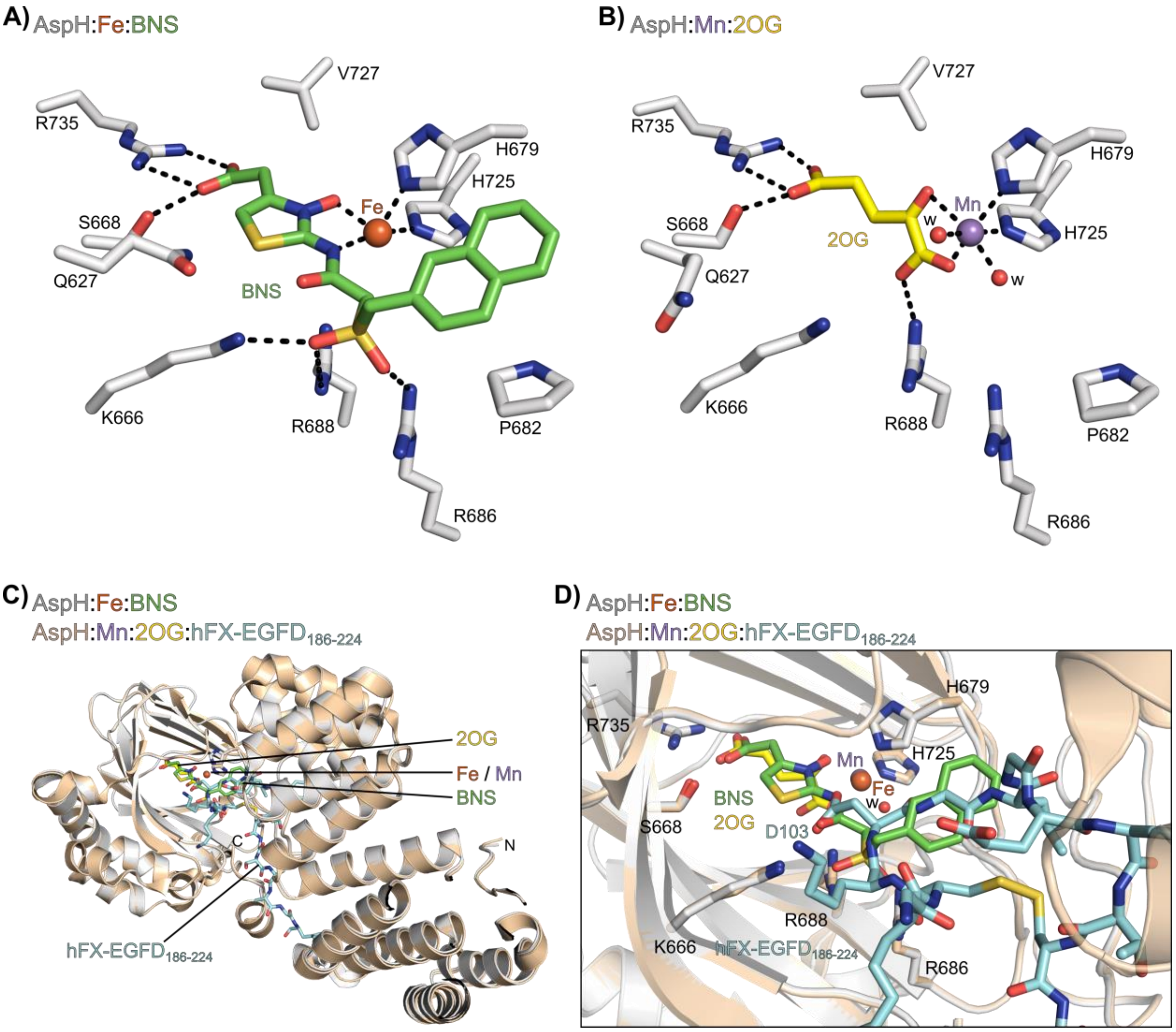
A) AspH:Fe:BNS
V727
R735
H679
H725
Fe
S668
Q627
BNS
K666
R688
P682
R686
B) AspH:Mn:2OG
V727
R735
H679
Mn
S668
w
H725
2OG
Q627
w
K666
R688
P682
R686
C) AspH:Fe:BNS
AspH:Mn:2OG:hFX-EGFD186-224
2OG
Fe / Mn
BNS
C
N
hFX-EGFD186-224
D) AspH:Fe:BNS
AspH:Mn:2OG:hFX-EGFD186-224
H679
R735
H725
Mn
Fe
BNS
w
2OG
S668
D103
R688
K666
hFX-EGFD186-224
R686

Supporting Figure S9. Docking studies predict that BNS likely binds to the active site of JMJD5 in a 2OG-competing manner (continues on the following page). Colours: grey: JMJD5; green: carbon-backbone of BNS[3, 4]; yellow: carbon-backbone of 2OG; salmon pink: carbon-backbone of NOG; purple: carbon-backbone of R137 of $RPS6_{129\text{-}144}$; orange: Fe; pink: Co; red: oxygen; blue: nitrogen; gold: sulphur. w: water. Molecular docking studies were performed using GOLD 5.1[21] and a reported JMJD5 (PDB ID: 4GJZ[27]) crystal structure, as described in Section 5 of the Supporting Information.

A) View of BNS docked into JMJD5. BNS is predicted to bind at the active site of JMJD5 and coordinate to the catalytic Fe(II) ion of JMJD5 in a bidentate manner through its *N*-hydroxyl group and the *exo*-nitrogen atom of its *N*-hydroxythiazole unit. The terminal carboxylate of BNS is predicted to be positioned to interact with the side chains of Tyr272, Ser318 and Lys336, and the sulfone is predicted to form a hydrogen bond with the side chains of Gln275.

B) View from a JMJD5:Co:2OG complex structure (PDB ID: 4GJZ[27]). Comparison of (**A**) and (**B**) indicates that BNS is predicted to compete with 2OG for binding to JMJD5 and that the carboxylate group of BNS may interact with JMJD5 in a similar manner as the C5 carboxylate group of 2OG. Note, however, that the *N*-hydroxyl group of BNS is predicted to coordinate Fe(II) in the same position as the 2OG ketone carbonyl (*i.e. trans* to Asp323), as observed by crystallography, but the predicted orientation of the metal coordination mode of the exo-nitrogen atom is perpendicular (*i.e. trans* to His400) to that observed for the C1 carboxylate of 2OG (*i.e. trans* to His321).

C and **D**) Superimposition of views from the JMJD5:BNS docking prediction and a reported JMJD5:Fe:NOG:$RPS6_{129\text{-}144}$ complex structure (light blue: JMJD5; PDB ID: 6F4P[28]) indicates that the naphthalene group of BNS is predicted to extend into the $RSP6_{129\text{-}144}$ substrate binding pocket. The superimposition therefore indicates that BNS will likely impair binding of the RPS6 substrate to JMJD5, in addition to competing with 2OG. Note that RSP6 Arg137 is observed in two alternative conformations in the JMJD5:NOG:$RSP6_{129\text{-}144}$ complex structure.

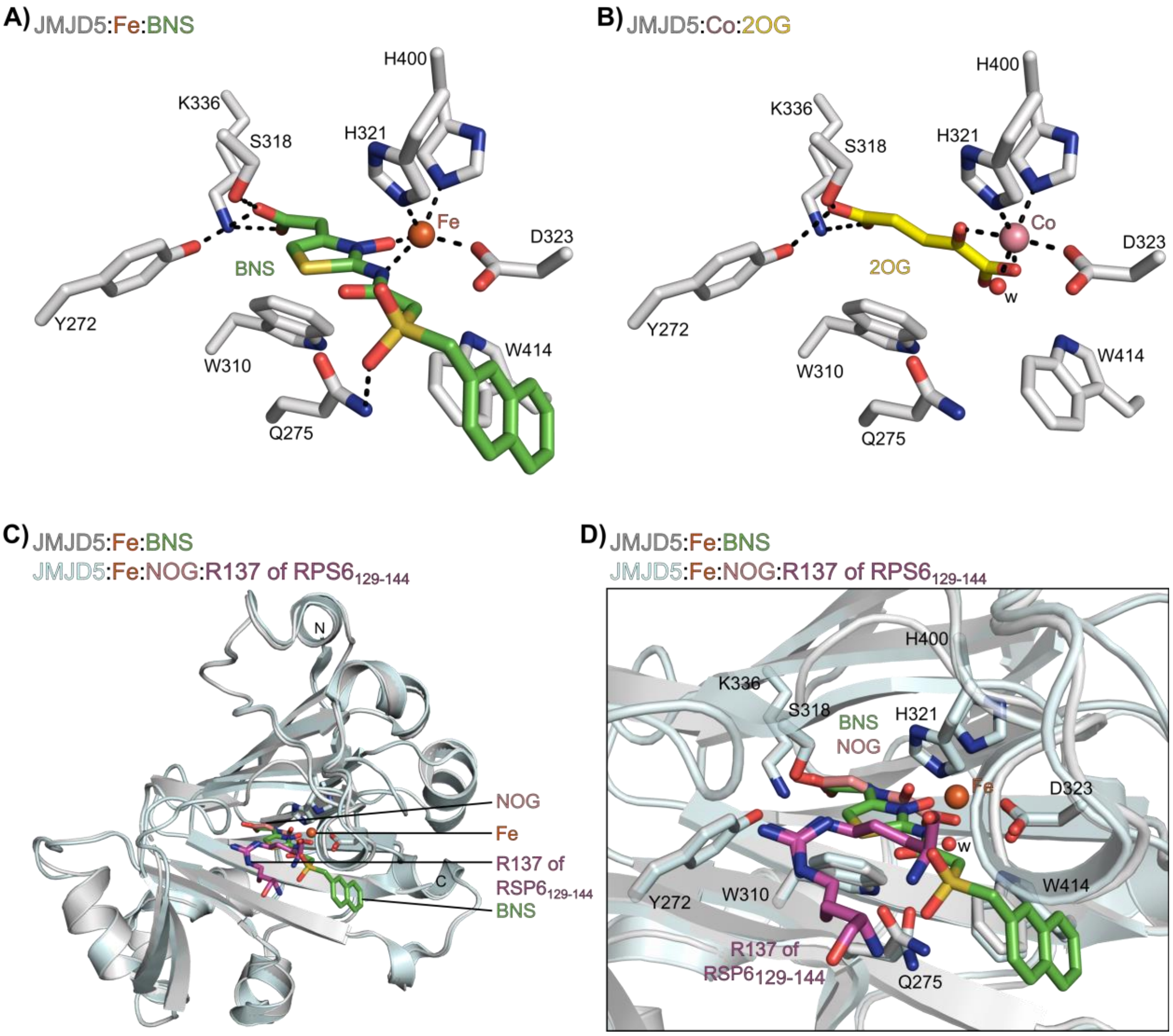
A) JMJD5:Fe:BNS
H400
K336
S318
H321
Fe
D323
BNS
Y272
W310
W414
Q275
B) JMJD5:Co:2OG
H400
K336
S318
H321
Co
D323
2OG
w
Y272
W310
W414
Q275
C) JMJD5:Fe:BNS
JMJD5:Fe:NOG:R137 of RPS6129-144
N
C
NOG
Fe
R137 of
RSP6129-144
BNS
D) JMJD5:Fe:BNS
JMJD5:Fe:NOG:R137 of RPS6129-144
H400
K336
S318
BNS
NOG
H321
Fe
D323
w
Y272
W310
W414
R137 of
RSP6129-144
Q275

Supporting Figure S10. Docking studies predict that the sulfonamide group of 21 may form hydrogen bonds with the side chains of AspH substrate-interacting residues (continues on the following page). Colours: grey: $AspH_{315-758}$; light blue: carbon-backbone of **21**; green: carbon-backbone of Factor X substrate peptide ($hFX\text{-}EGFD_{86-124}$); yellow: carbon-backbone of 2OG; orange: Fe; purple: Mn; red: oxygen; blue: nitrogen; gold: sulphur. w: water. Molecular docking studies were performed using GOLD 5.1[21] and the reported $AspH_{315-758}$ (PDB IDs: 6YYX, 6YYU[26]) crystal structures, as described in Section 5 of the Supporting Information.

A) View of **21** docked into $AspH_{315-758}$. **21** is predicted to bind to AspH in a similar manner to that predicted for BNS[3, 4] (**Supporting Figure S8**) via bidentate metal chelation and hydrogen bond interactions with Ser668, Arg686, Arg688 and Arg735. In addition, the sulfonamide group of **21** is predicted to interact with the side chain of Glu617.

B) View from an $AspH_{315-758}$:Mn:2OG complex structure (PDB ID: 6YYU[26]). Comparison of (**A**) and (**B**) indicates that **21** is predicted to compete with 2OG for binding to AspH; note, the *N*-hydroxyl group of **21** is predicted to coordinate Fe(II) in the same position as the 2OG ketone carbonyl, as observed by crystallography, but the predicted orientation of the metal coordination mode of the exo-nitrogen atom is perpendicular (*i.e. trans* to His725) to that observed for the C1 carboxylate of 2OG (*trans* to His679).

C) View from an AspH:Mn:2OG:$hFX\text{-}EGFD_{186-124}$ complex structure (PDB ID: 6YYW[26]). The side chains of Glu617 and Arg686 are positioned to form hydrogen bonds with the backbone of $hFX\text{-}EGFD_{86-124}$. The side chain of Arg688 is positioned to interact with carboxylate side chain of $hFX\text{-}EGFD_{86-124}$ Asp103 (*i.e.* the hFX-EGFD Asp-residue that undergoes AspH-catalysed C3 hydroxylation). As **21** is predicted to also form hydrogen bonds with the side chains of Glu617, Arg686 and Arg688, this observation indicates that **21** will likely prevent productive binding of the hFX-EGFD substrate to AspH.

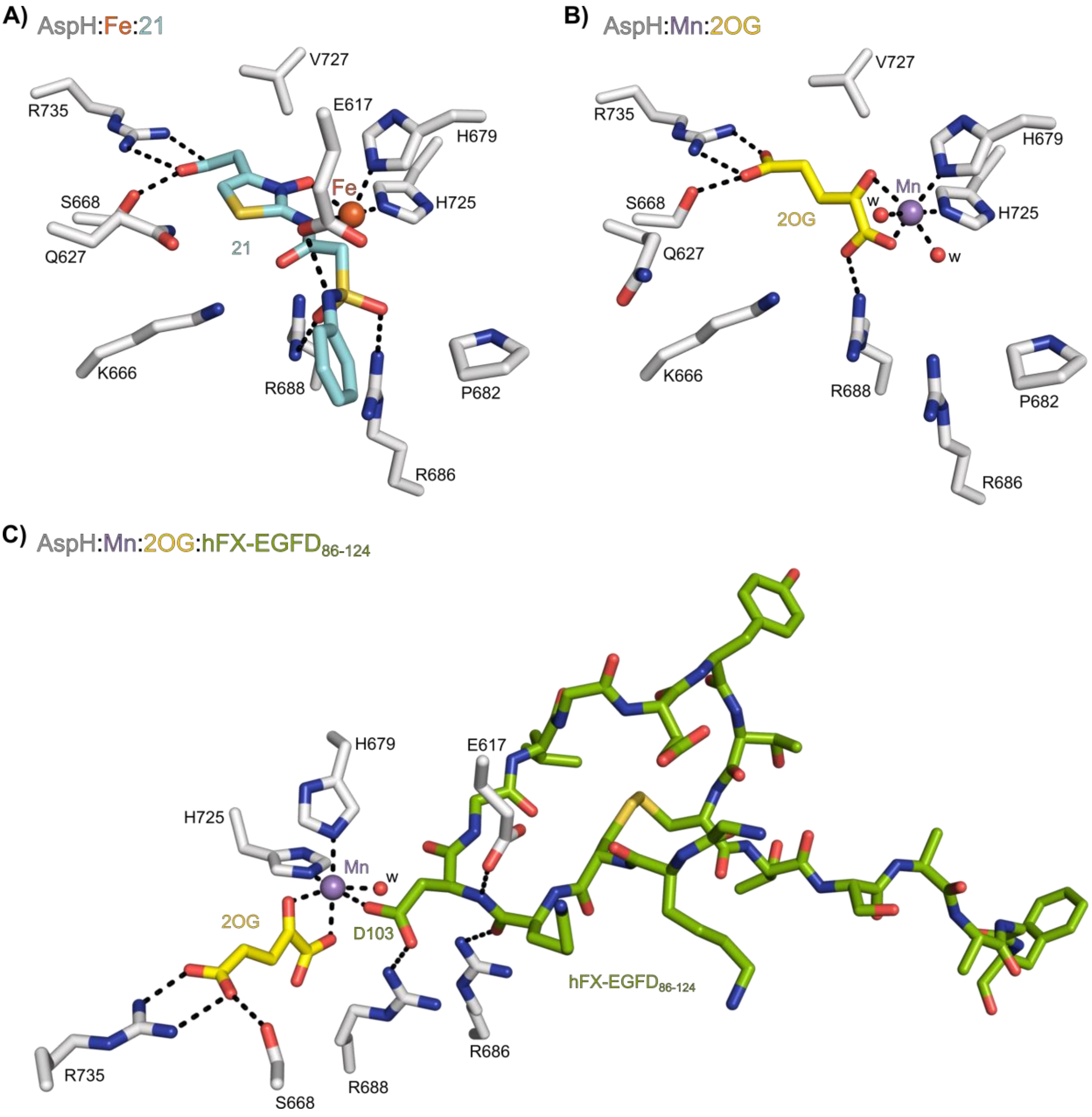

A) AspH:Fe:21
V727
E617
R735
H679
Fe
S668
H725
Q627
21
K666
R688
P682
R686
B) AspH:Mn:2OG
V727
R735
H679
S668
Mn
w
2OG
H725
Q627
w
K666
R688
P682
R686
C) AspH:Mn:2OG:hFX-EGFD$_{86-124}$
H679
E617
H725
Mn
w
2OG
D103
hFX-EGFD$_{86-124}$
R735
R686
S668
R688

Supporting Figure S11. Rational for the selectivity of NOFD for FIH inhibition over that of PHD2 (continued on the following page). Colours: light green: FIH; light pink: $PHD2_{181\text{-}426}$; yellow: carbon-backbone of 2OG; green: carbon-backbone of NOG; salmon pink: carbon-backbone of NOFD[11]; orange: Fe; purple: Mn; red: oxygen; blue: nitrogen. w: water.

A) View from a reported FIH:Fe:2OG complex structure (PDB ID: 1MZF[14]) reveals that 2OG binds at the FIH active site and coordinates to Fe in a bidentate manner through its C1 carboxylate and C2 ketone carbonyl group. The C5 carboxylate group of 2OG is positioned to interact with the side chains of Tyr145, Thr196 and Lys214.

B) View from a reported $PHD2_{181\text{-}426}$:Mn:NOG complex structure (PDB ID: 5L9R[25]) reveals that NOG binds at the PHD2 active site and coordinates to Mn in a bidentate manner through its C1 carboxylate and C2 amide carbonyl group. The C5 carboxylate group of NOG is positioned to interact with the side chains of Tyr329 and Arg383.

Comparison of (**A**) and (**B**) indicates that the opening to active site of FIH is wider than that in PHD2.[29] In the PHD2:Mn:NOG complex structure, the side chain of Leu343 is in close proximity with the C4 carbon atom of NOG. By contrast, in the FIH:Fe:2OG complex structure, there is a vacant pocket adjacent to the C4 carboxylate of 2OG.

C) Superimposition of views from a reported FIH:Fe:2OG (PDB ID: 1MZF[14]) and a FIH:Fe:NOFD (PDB ID: 1YCI[11]) complex structures reveals that the core *N*-oxalyl glycine unit of NOFD binds to FIH in a similar manner to 2OG via bidentate coordination to Mn. The benzyl side chain of NOFD is observed to occupy a vacant hydrophobic pocket adjacent to the C4 carbon atom of 2OG, which is formed by FIH active site residues Tyr102, Tyr145, Gln147 and Leu186.

D) View from the active site of a reported FIH:Fe:NOFD complex structure (PDB ID: 1YCI[11]) reveals that NOFD coordinates to Fe in a bidentate manner through its C1 carboxylate and C2 amide carbonyl group. The carboxylate group of NOFD is positioned to interact with the side chains of Tyr145, Thr196 and Lys214, in a manner similar to that observed for the C5 carboxylate of 2OG in the FIH:Fe:2OG complex structure.

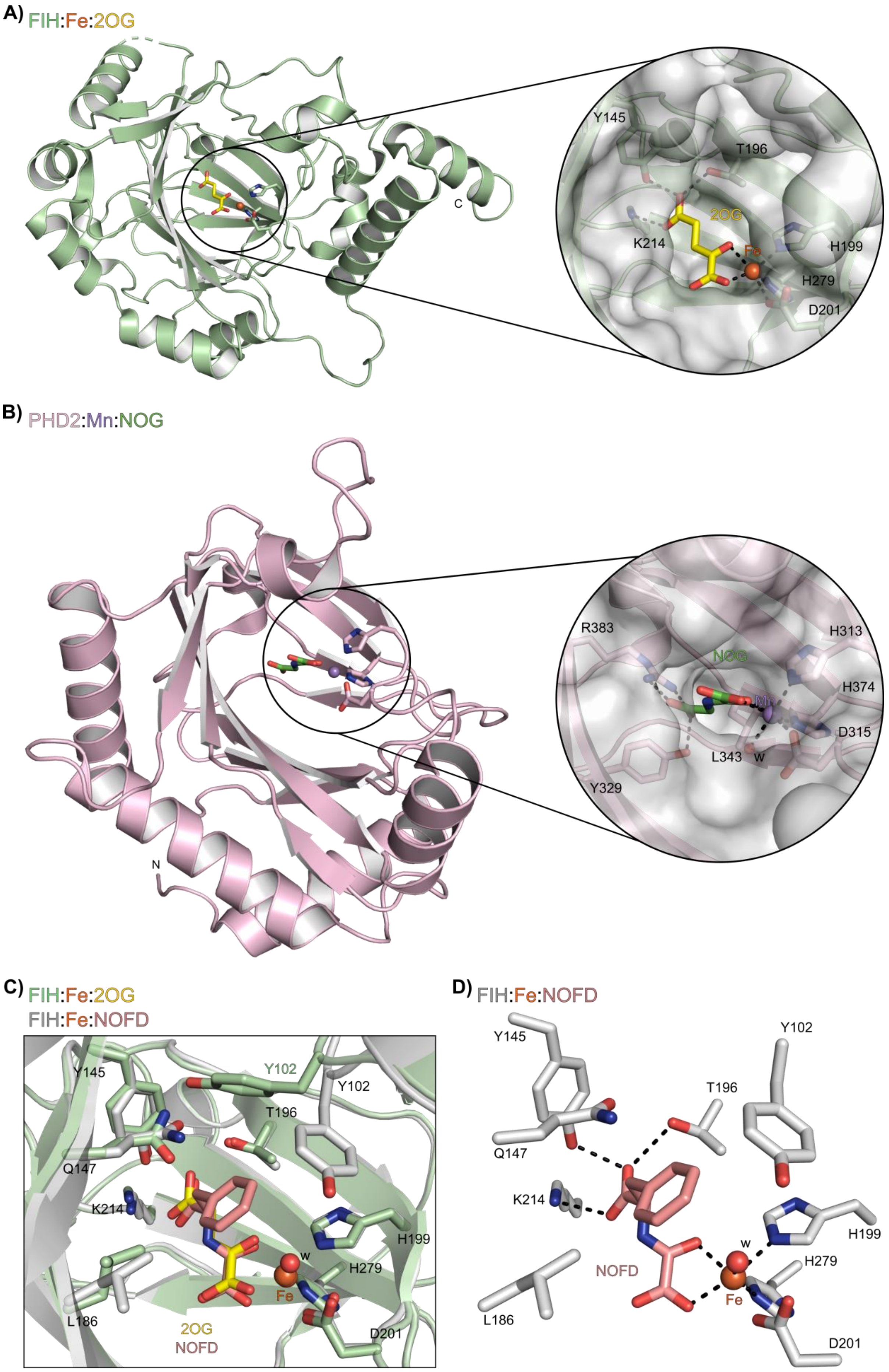
A) FIH:Fe:2OG
C
Y145
T196
2OG
K214
Fe
H199
H279
D201
B) PHD2:Mn:NOG
N
R383
H313
NOG
H374
Mn
D315
L343
W
Y329
C) FIH:Fe:2OG
FIH:Fe:NOFD
Y102
Y145
Y102
T196
Q147
K214
H199
W
H279
Fe
L186
2OG
NOFD
D201
D) FIH:Fe:NOFD
Y145
Y102
T196
Q147
K214
H199
W
NOFD
H279
Fe
L186
D201

2. Supporting synthetic schemes

Supporting Scheme S1. Synthesis of thiazole derivative 5.[a]

Thiazole **5** was synthesised via lithium hydroxide-mediated saponification of ethyl ester **2** (75% yield).

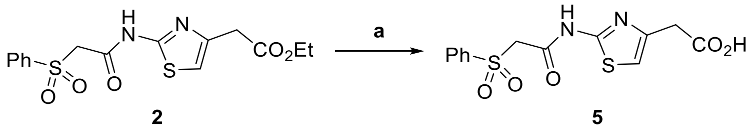

[a]Reagents and conditions: (a) LiOH, MeOH/H_2O, 0 °C to rt, 75%

Supporting Scheme S2. Synthesis of *N*-hydroxythiazole derivatives 6 and 7.[a]

N-Hydroxythiazoles **6** and **7** were synthesised in four steps, via a modified literature procedure,[3, 30] from β-ketoesters **6a** and **7a**, respectively (8% and 7% yields over four steps). **6** was prepared as a racemic mixture. Initially, **6a** and **7a** were brominated and subsequently treated with thiourea to give thiazoles **6b** and **7b**, which were then coupled with 2-phenylsulfonyl)acetic acid using T3P[31] as a coupling reagent to generate **6c** and **7c**. Carboxylic acids **6** and **7** were obtained following mCPBA-mediated thiazole *N*-oxidation and lithium hydroxide-mediated ester saponification.

N-Hydroxythiazoles **6**, **7**, **6d** and **7d** have been putatively assigned as the (*Z*)-configuration based on a previously reported *N*-hydroxythiazole small-molecule crystal structure[3] and the FIH:*N*-hydroxythiazole complex structures described in this work.

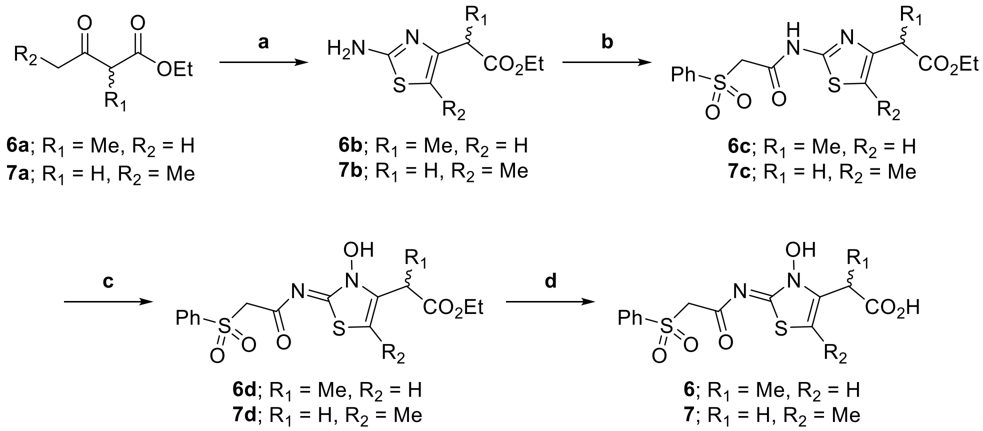

[a]Reagents and conditions: (a) pyridinium tribromide, $CHCl_3$, 40 °C; then, thiourea, EtOH, reflux, 45-69%; (b) 2-(phenylsulfonyl)acetic acid, T3P[31], *i*Pr_2NEt, DMF, 0 °C to rt, 83-86%; (c) mCPBA, $CHCl_3$, rt, 39-43%; (d) LiOH, MeOH/H_2O, 0 °C to rt, 27-58%.

Supporting Scheme S3. Synthesis of *N*-hydroxythiazole derivatives 8-13 (continued on the following page).[a]

A) For the synthesis of *N*-hydroxythiazole **8**, 2-aminothiazole **8a** was coupled with commercially-sourced 2-chloroacetylchloride to give amide **8b**, followed by chloride displacement with sodium phenyl sulfinate to generate phenyl sulfone **8c**. Finally, thiazole *N*-oxidation with mCPBA gave the desired *N*-hydroxythiazole **8**.

B) Methyl ester **9** was synthesised via transesterification of ethyl ester **3** with sodium methoxide in 58% yield.

C) *N*-Methyl amide **10** was obtained via amide coupling of carboxylic acid **5** and methylamine hydrochloride using HATU[32] as a coupling reagent, followed by mCPBA-mediated thiazole *N*-oxidation (14% yield over two steps).

D) Nitrile **11** was obtained in three steps from ethyl ester **3** (7% yield over three steps). Initially, ethyl ester **3** was converted to **11a** using 7N methanolic ammonia. Then, dehydration of **11b** to generate nitrile **11c** was performed using the Burgess reagent,[33] followed by mCPBA-mediated thiazole *N*-oxidation to give target compound **11**.

E) Triazole **12** was prepared in three steps from commercially-sourced 2-amino-4-(chloromethyl)thiazole hydrochloride **12a** (2% yield over 3 steps). Amide coupling with (phenylsulfonyl)acetic acid using EDC·HCl and HOBt gave amide **12b**. Introduction of the triazole moiety was achieved using 1*H*-1,2,3-triazole and K_2CO_3 to afford **12c** in 28% yield. The 1*H*-1,2,5-triazole regioisomer was also obtained in 18% yield, however, this isomer was successfully removed by column chromatography. Thiazole *N*-oxidation using mCPBA gave target compound **12**.

F) **13** was synthesised in four steps from 4-oxopentanoic acid **13a** (5% yield over 4 steps). Esterification and bromination of **13a** was carried out using Br_2 under reflux in MeOH. Subsequent condensation with thiourea gave thiazole **13b** in 30% yield. Side products resulting from undesired C3 bromination of **13a** were also observed, however, these were successfully removed via column chromatography. **13b** was then coupled with 2-phenylsulfonyl)acetic acid using T3P[31] as a coupling reagent to generate **13c**. Carboxylic acid **13** was obtained following mCPBA-mediated thiazole *N*-oxidation and lithium hydroxide-mediated ester saponification.

N-Hydroxythiazoles **8**-**13** and **13d** have been putatively assigned as the (*Z*)-configuration based on a previously reported *N*-hydroxythiazole small-molecule crystal structure[3] and the FIH:*N*-hydroxythiazole complex structures described in this work.

A)

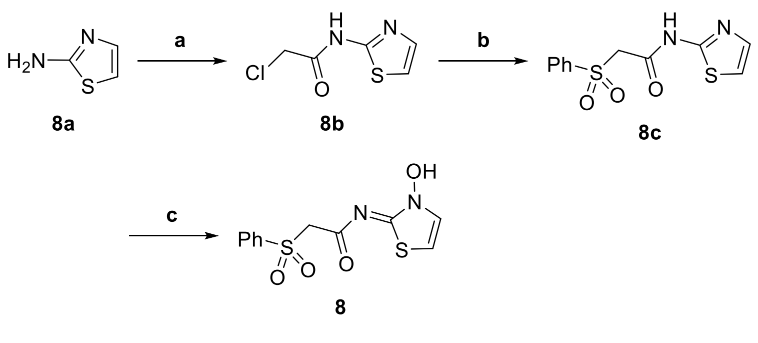

B)

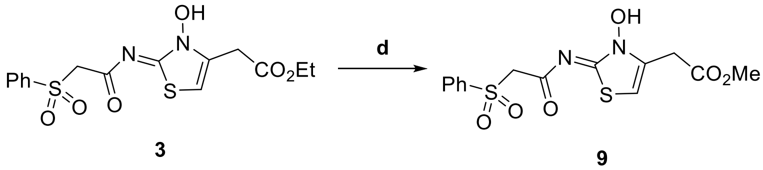

C)

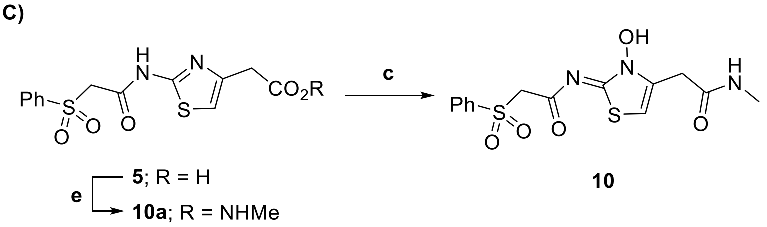

D)

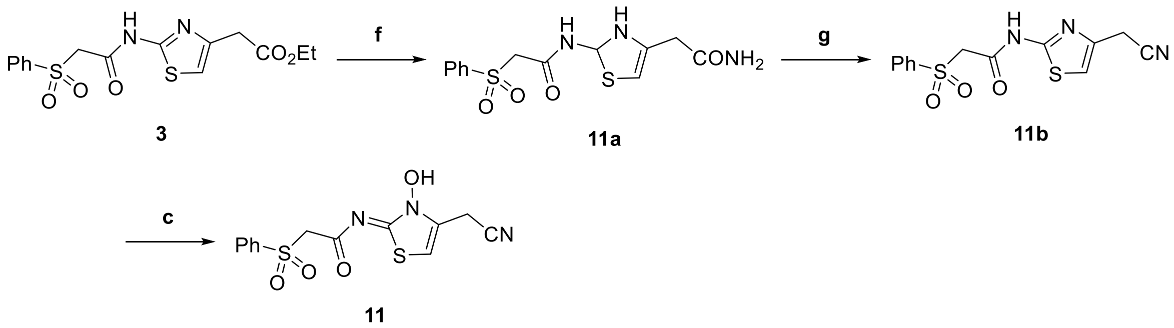

E)

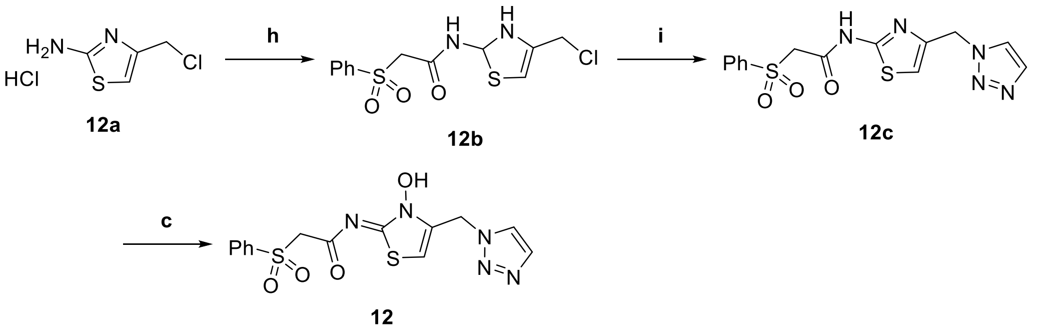

F)

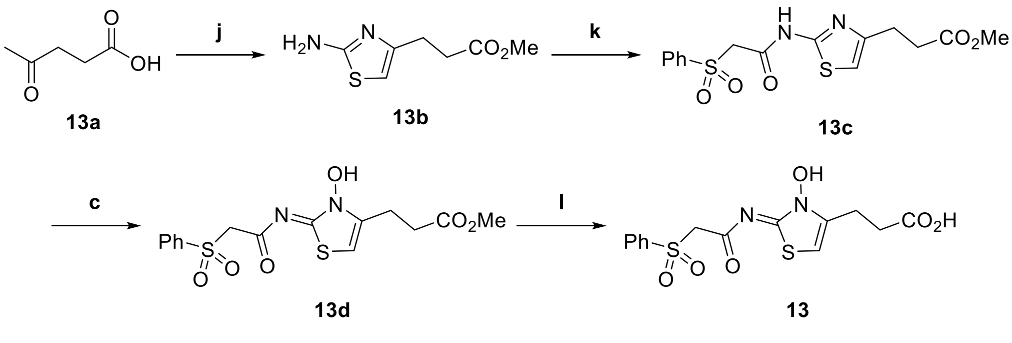

[a]Reagents and conditions: (a) 2-chloroacetyl chloride, K_2CO_3, CH_2Cl_2, 0 °C to rt, 63%; (b) sodium benzenesulfinate, EtOH, reflux, 12%; (c) mCPBA, $CHCl_3$, rt, 25-50%; (d) NaOMe, MeOH, 0 °C to rt, 58%; (e) NH_2Me.HCl, HATU[32], *i*PrNEt$_2$, DMF, 0 °C to rt, 46%; (f) NH_3/MeOH, MeOH, rt, 60%; (g) Burgess reagent[33], 0 °C to rt, 23%; (h) 2-(phenylsulfonyl)acetic acid, EDC.HCl, HOBt, *i*Pr$_2$NEt, CH_2Cl_2, 0 °C to rt, 21%; (i) 1*H*-1,2,3-triazole, K_2CO_3, DMF, 80 °C, 28%; (j) Br_2, MeOH, reflux; then, thiourea, EtOH, reflux, 30%; (k) T3P[31], *i*Pr$_2$NEt, DMF, 0 °C to rt, 82%; (l) LiOH, MeOH/H_2O, 0 °C to rt, 49%.

Supporting Scheme S4. Synthesis of *N*-hydroxythiazole derivatives 14-23 (continued on the following page).[a]

A) *N*-Hydroxythiazole **14** was synthesised in three steps from thiazole **1** (4% yield over 3 steps). Reaction with acetic anhydride in the presence of 4-DMAP gave amide **14a**. Thiazole *N*-oxidation with mCPBA, followed by lithium hydroxide-mediated ester hydrolysis generated **14**.

B) The synthesis of *N*-hydroxythiazoles **15** and **21-23** was performed according to a modified literature procedure.[3] First, thiazole **1** was coupled with carboxylic acids using T3P[31] as a coupling reagent, followed by mCPBA-mediated thiazole *N*-oxidation to afford **15b** and **21b**-**23b**. Deprotection of *N*-Boc protected aniline **23b** was achieved using TFA. Finally, carboxylic acids **15** and **21**-**23** were obtained following lithium hydroxide-mediated ester hydrolysis (overall yields: 9-49% over 3/4 steps).

C) *N*-Hydroxythiazoles **16**, **17**, **19** and **20** were prepared in five steps from thiazole **1** (8-26% yields over 5 steps). **1** was coupled with the corresponding *N*-Boc protected amino acid using T3P[31] as a coupling reagent, followed by acid-mediated *N*-Boc deprotection to generate **16b** and **17b**. Then, either sulfonamide or amide coupling gave **16c**, **17c**, **19a** and **20a**. Carboxylic acids **16**, **17**, **19** and **20** were obtained following mCPBA-mediated thiazole *N*-oxidation and lithium hydroxide-mediated ester saponification.

D) *N*-Hydroxythiazole **18** was synthesised in four steps from thiazole **1** (10% yield over 4 stpes). **1** was reacted with succinic anhydride, followed by amide coupling with aniline using T3P[31] as a coupling reagent to generate amide **18b**. Carboxylic acid **18** was obtained following mCPBA-mediated thiazole *N*-oxidation and lithium hydroxide-mediated ester saponification.

N-Hydroxythiazoles **14**-**20**, **14b**, **15b**, **16d**, **17d**, **18c**, **19b**-**23b** and **23c** have been putatively assigned as the (*Z*)-configuration based on a previously reported *N*-hydroxythiazole small-molecule crystal structure[3] and the FIH:*N*-hydroxythiazole complex structures described in this work.

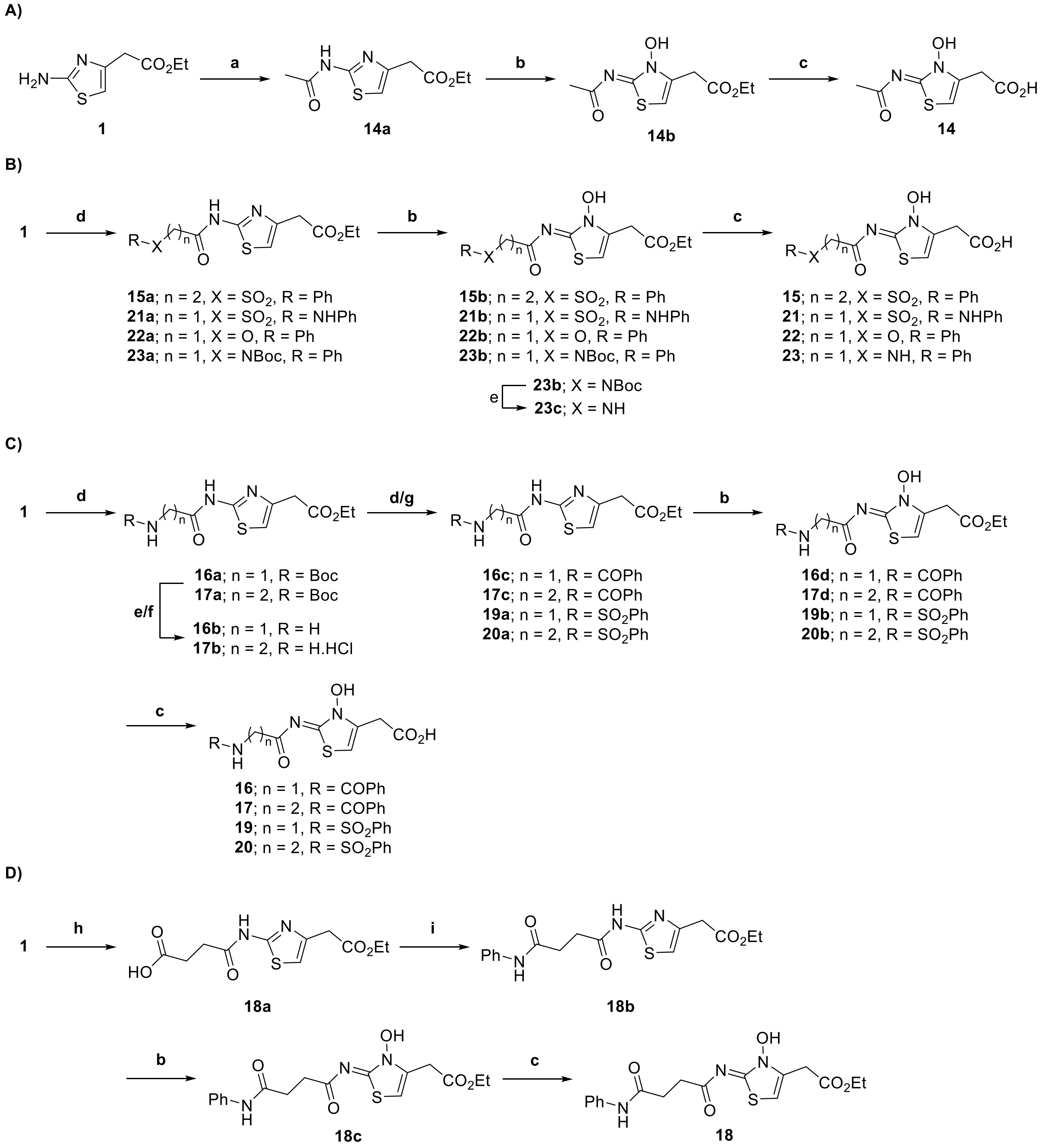

[a](a) Ac_2O, 4-DMAP, THF, reflux, 58%; (b) mCPBA, $CHCl_3$, 0 °C to rt, 42-82%; (c) LiOH, MeOH/H_2O, 0 °C to rt, 10-73%; (d) carboxylic acid, T3P[31], *i*Pr_2NEt, DMF, 0 °C to rt, 54-88%; (e) TFA, CH_2Cl_2, 0 °C to rt, 75%; (f) HCl/dioxane, 0 °C to rt, 99%; (g) $PhSO_2Cl$, Et_3N, CH_2Cl_2, 0 °C to rt, 69-79%; (h) succinic anhydride, THF, reflux, 77%; (i) aniline, T3P[31], *i*Pr_2NEt, DMF, 0 °C to rt, 81%.

Supporting Scheme S5. Synthesis of *N*-hydroxythiazole derivatives 24-30 (continued on the following page).[a]

A) *N*-Hydroxythiazoles **24**-**27** and **29** were prepared in five steps from thiazole **1** (2-16% yields over 5 steps). **26**, **27** and **29** were prepared as racemic mixtures. First, **1** was coupled with the corresponding *N*-Boc protected amino acid using T3P[31] as a coupling reagent, followed by HCl-mediated *N*-Boc deprotection to generate amine HCl salts **24b**, **25b**, **27b**, **29b** and **32**. Reaction of **24b**, **25b**, **27b**, **29b** and **32** with benzene sulfonyl chloride gave **24c**, **25c**, **27c**, **29c** and **26a**, respectively**.** Carboxylic acids **24**-**27** and **29** were then obtained following mCPBA-mediated thiazole *N*-oxidation and lithium hydroxide-mediated ester saponification.

B) *N*-Hydroxythiazole **28** was prepared as racemic mixture in five steps from thiazole **1** (6% yield over 5 steps). First, **1** was coupled with commercially-sourced (±)-1-(*tert*-butoxycarbonyl)pyrrolidine-2-carboxylic acid using T3P[31] as a coupling reagent, followed by HCl-mediated *N*-Boc deprotection to generate amine HCl salt **28b**. Reaction of **28b** with benzene sulfonyl chloride gave **28c**. Carboxylic acid **28** was then obtained following mCPBA-mediated thiazole *N*-oxidation and lithium hydroxide-mediated ester saponification.

C) Preparation of racemic *trans* 3,4-substituted derivative **30** required the synthesis of amine intermediate **30c**, via a [3+2] cycloaddition and subsequent *N*-debenzylation, as previously reported.[34] **30c** was reacted with benzene sulfonyl chloride and hydrolysed using LiOH to generate carboxylic acid **30e**, which was subsequently coupled with 2-aminothiazole using T3P[31] as a coupling reagent to give amide **30f**. Carboxylic acid **30** was obtained following mCPBA-mediated thiazole *N*-oxidation and lithium hydroxide-mediated ester saponification (overall yield: 1% over 7 steps). To avoid epimerization of the pyrrolidine ring during the ester hydrolysis steps, it was necessary to use THF/H_2O as the solvent system.

N-Hydroxythiazoles **24**-**30**, **24d**, **25d**, **26b**, **27d**-**29d** and **30g** have been putatively assigned as the (*Z*)-configuration based on a previously reported *N*-hydroxythiazole small-molecule crystal structure[3] and the FIH:*N*-hydroxythiazole complex structures described in this work.

A)

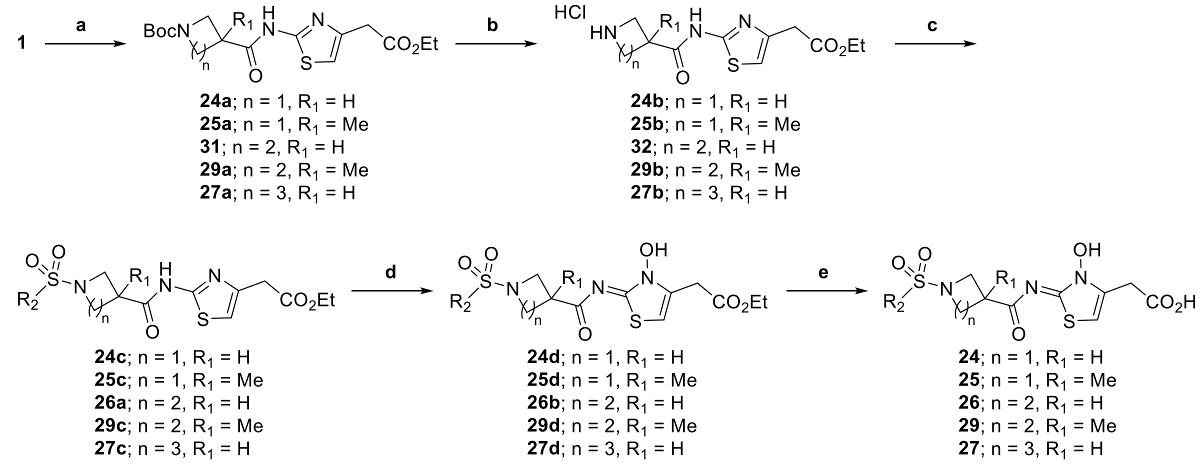

B)

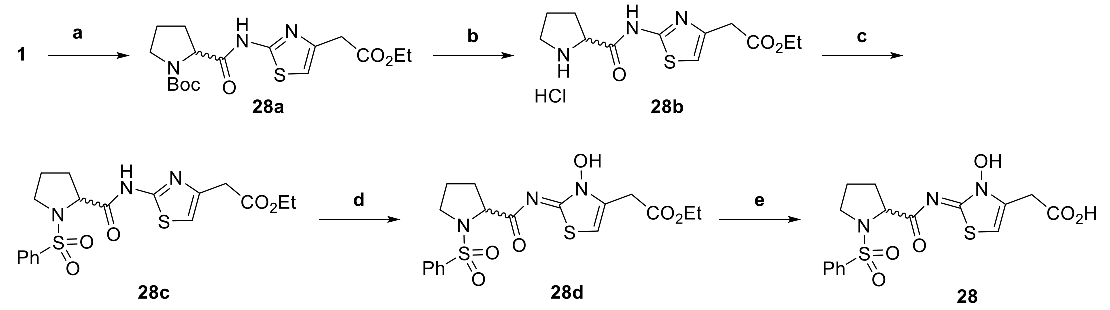

C)

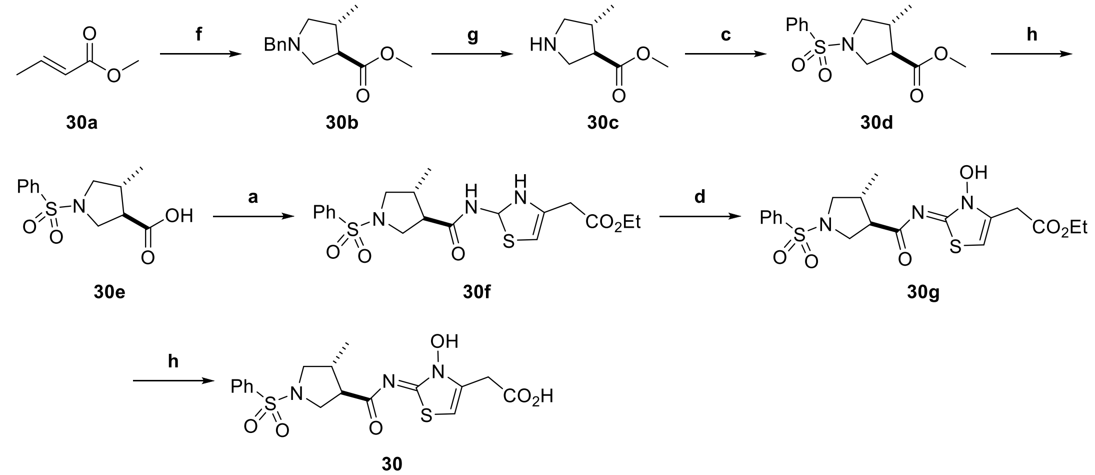

[a](a) Carboxylic acid, T3P[31], *i*Pr_2NEt, DMF, 0 °C to rt, 35-99%; (b) HCl/dioxane, 0 °C to rt, 54-99%; (c) $PhSO_2Cl$, Et_3N, CH_2Cl_2, 0 °C to rt, 34-82%; (d) mCPBA, $CHCl_3$, 0 °C to rt, 43-73%; (e) LiOH, MeOH/H_2O, 0 °C to rt, 17-69%; (f) *N*-(methoxymethyl)-*N*-(trimethylsilylmethyl)benzylamine, TFA, CH_2Cl_2, 0 °C to rt, 67%; (g) NH_4HCO_2; $Pd(OH)_2/C$; MeOH, reflux, 37%; (h) LiOH, THF/H_2O, 0 °C to rt, 19%.

3. Biochemical procedures

Protein production and purification

FIH,[2] PHD2$_{181-426}$,[1] AspH$_{315-758}$,[35] JMJD5[36] and KDM4A[37] were prepared to high purity (>90% by SDS-PAGE analysis) according to established procedures.

FIH, PHD2, AspH, JMJD5 and KDM4A inhibition assays

The *in vitro* FIH,[1, 2] PHD2,[1] AspH,[38] JMJD5[36] and KDM4A[39] inhibition assays were performed as previously described using recombinant human enzymes (FIH, PHD2$_{181-426}$, AspH$_{315-758}$, JMJD5 and KDM4A). Synthetic peptide substrates were used: HIF-1α C-terminal transactivation domain fragment (HIF-1α C-TAD$_{788-822}$) for FIH;[1, 2] HIF-1α C-terminal oxygen-dependent degradation domain fragment (HIF-1α CODD$_{556-574}$) for PHD2;[1] human Factor X cyclic peptide fragment (hFX-CP$_{101-119}$) for AspH;[38] 40S ribosomal protein S6 fragment (RSP6$_{128-148}$); histone 3 (H3) variant fragment (H3$_{1-15}$K9me3$_{1–15}$ with H3Lys9 bearing three methyl groups at the N^{ϵ} position, H3Lys4 substituted by an Ala and H3Lys14 substituted by an Ile residue) for KDM4A.[39] Peptides were prepared as C-terminal amides by GL Biochem (Shanghai) Ltd. Peptide hydroxylation in the case of FIH, PHD2, AspH and JMJD5 (+16 Da mass shift) or peptide demethylation in the case of KDM4A (-14 Da mass shift) was monitored by SPE-MS.

Crystallography

FIH crystallography was carried out as reported.[19] N-Terminally His$_6$-tagged FIH (0.27 mM, final concentration) was mixed with zinc acetate (0.5 mM) in 50 mM Tris buffer (pH 7.5) and incubated at 4 °C for 5 min. A *N*-hydroxythiazole derivative (final concentration: 2 mM) was added and the mixture was incubated at 4 °C for a further 15 min. The FIH-inhibitor mixture was then centrifuged with a MicroCL 21R (Thermo Fisher Scientific) at 14,000 rpm (18,800 xg) at 4 °C for 10 min.

Crystallisations were performed in 96-well, three-subwell, low profile Intelliplates (Art Robbins Instruments) using a Mosiquito LCP (SPT Labtech) dispensing robot with 1.6 M ammonium sulfate, 6%$_{w/v}$ PEG 400, and 0.1 M HEPES buffer (pH 7.5) as the precipitant solution.

FIH crystals were grown using the sitting-drop vapor diffusion method at 20 °C in 300 nL sitting drops with 2:1, 1:1, or 1:2 sample:precipitant solution ratios. Crystals were cryo-protected using mother liquor supplemented with 25%$_{v/v}$ glycerol before manual loop cryo-cooling in liquid N_2.

Data were collected at I03 beamline at Diamond Light Source (UK). Data were indexed, integrated, and scaled using the Xia2[40] strategy of the beamline auto-processing pipeline (**Supporting Table S2**).

The FIH crystal structures were determined by molecular replacement (MR) using the AutoMR (PHASER)[41] subroutine in PHENIX[42] based on a reported FIH crystal structure (PDB ID: 4B7K[43]). The structural model was improved by COOT[44] and phenix.refine[42] (**Supporting Table S2**).

Crystal structure data for FIH complexed to Zn, *N*-hydroxythiazole derivatives are deposited in the protein data bank with PDB accession codes: **8K71** (FIH:Zn:BNS), **8K72** (FIH:Zn:**20**), and **8K73** (FIH:Zn:**26**). PyMOL (version 4.6.0)[45] was used for the generation of graphical representations; omit maps were calculated using Polder Maps[46] in PHENIX (version 1.18.2).[42]

4. Methods for cell studies

Cell culture

All cells were maintained in a 10% CO_2 environment at 37 °C. Hep3B cells (SCSP-5045) were maintained in Minimum Essential Medium (MEM, GIBCO) with 10% fetal bovine serum (FBS, BI), 100 U/mL penicillin and 100 μg/mL streptomycin (1 × P/S, NCM Biotech). 786-O cells (SCSP-5059) were maintained in RPMI 1640 Medium (GIBCO) with 10% fetal bovine serum (FBS, BI), 100 U/mL penicillin and 100 μg/mL streptomycin (1 × P/S, NCM Biotech).

Quantitative PCR Analysis of Gene Expression

To investigate the effect of inhibitors on HIF target gene expression, Hep3B and 786-O cells were treated with vehicle (DMSO) or an inhibitor (at the concentrations indicated) for 12 h. Total RNA was isolated from cells and tissues using Trizol regents (Vazyme, China). 1 μg RNA was used for reverse transcription using HiScript III RT SuperMix for qPCR (Vazyme, China). cDNAs were amplified in a ChamQ Universal SYBR qPCR Master Mix (Vazyme, China). Quantitative PCR (qPCR) was performed on a ABI QuantStudio 3 system. PCR conditions: 3 min at 95 °C; 45 cycles of 10 s at 95 °C and 30 s at 60 °C; 15 s at 95 °C, 1 min at 60 °C and 15 s at 95 °C. The relative amount of mRNA was calculated after normalization to HPRT.[19]

Genes	Primer sequence	
	Forward	Reverse
HPRT	5'-GACCAGTCAACAGGGGACAT-3'	5'-AACACTTCGTGGGGTCCTTTTC-3'
EGLN3	5'-CTGGTCCTCTACTGCGGGA-3'	5'-AGCCACCATTGCCTTAGACCTC-3'
SLC2A1	5'-GCCAAGAGTGTGCTAAAGAAGC-3'	5'-GCCGACTCTCTTCCTTCATCTC-3'
CA9	5'-AGCACAGAAGGGGAACCAAAG-3'	5'-ATGAGCAGGACAGGACAGTTAC-3'
EPO	5'-GAGCCCAGAAGGAAGCCATC-3'	5'-CGGAAAGTGTCAGCAGTGATTG-3'

3T3-L1 derived adipocytes.

3T3-L1 cells (SCSP-5038) were maintained in Dulbecco's Modified Eagle Medium (DMEM, GIBCO) with 10% newborn calf serum (NCS, GIBCO), 100 U/mL penicillin and 100 μg/mL streptomycin (1 × P/S, NCM Biotech) in a 10% CO_2 environment at 37 °C. The 3T3-L1 cells were strictly subcultured before they reached a density of 6×10^4 viable cells/cm^2. Subcultures of 3T3-L1 cells were routinely cultured in DMEM medium containing 10% NCS for two days, and then the cells were cultured in DMEM containing 10% FBS, 0.5 mM 3-isobutyl-1-methylxanthine, 1 μM dexamethasone, 10 μg/mL insulin and 2 μM rosiglitazone for another two days. Subsequently, the cells were cultured in DMEM containing 10% FBS and 10 μg/mL insulin for two days. After these procedures, the cells were maintained in DMEM containing 10% FBS. The last induction procedure was repeated 2-3 times until lipid droplets appeared.[47]

Viability Assay for 3T3-L1 derived adipocytes.

To investigate the effect of inhibitors on adipocytes, 3T3-L1-derived adipocytes were treated with vehicle (DMSO) or with an inhibitor (at the concentrations indicated) for 48 h. Cell viability was

accessed by the Cell Counting Kit-8 (CCK-8) assay (Enogene, China).[48] In brief, the 3T3-L1-derived adipocytes in 96-well plates were treated with compounds as indicated. After 48 h, the cells were incubated with CCK-8 at 37 °C for 1-4 h. The cell viability was detected using SPARK Multi-Mode Microplate Reader (Tecan) at OD 450 nm.

Cellular TG level assay for 3T3-L1 derived adipocytes.

The 3T3-L1-derived adipocytes were washed with PBS, then the cells were lysed. And then, the cellular TG level was determined by using a cellular triglyceride assay kit (Applygen, China).

Oil Red O staining for 3T3-L1 derived adipocytes.

The 3T3-L1-derived adipocytes were washed with PBS, then fixed for 10 min with $4\%_{v/v}$ paraformaldehyde (PFA, #P0099, Beyotime). Subsequently, the fixed cells were incubated with Oil Red O for 15 min, then washed with the wash buffer (#C0158S, Beyotime).

5. Computational methods

General protein structure preparation procedure

PHD2 (PDB ID: 4BQY[22]), JMJD5 (PDB ID: 4GJZ[27]) and AspH (PDB IDs: 6YYX, 6YYU[26]) crystal structures were downloaded from the Protein Data Bank (https://www.rcsb.org/).[49] Hydrogen atoms were added, and Asn/Gln/His residues checked for flips with REDUCE,[50] using the MolProbity server.[51] Missing side chain atoms were added using the 'Mutagenesis' tool in Pymol (version 4.6.0).[45] The pKa values of all ionizable groups were calculated using PropKa[52] and protonated using Pymol at pH 7.5. The catalytic domain active site metal ion was replaced with Fe^{II}. Alternative side chain conformations, bound ligands, and all crystallographic waters were removed using Pymol.

Gold docking procedure

Molecular docking studies were performed using the protein-ligand docking software Gold (version 5.1).[21] For PHD2 and JMJD5, a single receptor structure was used (PDB IDs: 4BQY[22] and 4GJZ[27], respectively). For AspH, an ensemble of two receptor structures were used (PDB IDs: 6YYX, 6YYU[26]). For each ligand, 100 genetic algorithm (GA) runs were carried out and the ChemScore scoring function was used to evaluate the predicted ligand binding poses. For each GA run, a maximum of 125,000 operations was performed. The binding site was defined as all atoms within 20 Å of the catalytic Fe^{II}. The following ligand flexibility parameters were enabled: Flip pyramidal N, Detect internal H bonds, and Flip ring corners. The 'Allow early termination' option was disabled. The coordination geometry of the Fe^{II} ion was set as octahedral. All other settings were used as the default.

6. Supporting tables

Supporting Table S1. Inhibition of FIH by selected PHD2 inhibitors, the reported broad spectrum 2OG oxygenase inhibitors 2,4-pyridinedicarboxylic acid (2,4-PDCA) and *N*-oxalylglycine (NOG), and the reported FIH selective inhibitor *N*-oxalyl-ᴅ-phenylalanine (NOFD).

Entry	Cmpd	FIH IC_{50} [µM][a,b]
**1**	2,4-PDCA	5.0 ± 2.1[53]
**2**	NOG	0.36 ± 0.03
**3**	NOFD[11]	0.24 ± 0.02
**4**	BNS[3, 4]	0.30 ± 0.07
**5**	Desidustat[5]	41.4 ± 1.1
**6**	TP0463518[6]	39.7 ± 0.6
**7**	GSK360A[7]	>100
**8**	JNJ-42041935[8]	56.4 ± 1.6
**9**	Enarodustat[9]	>100
**10**	MK-8617[10]	>100
**11**	FG-4592[54]	>100[19]
**12**	Daprodustat[55]	21[19]
**13**	Molidustat[56]	66[19]
**14**	IOX4[57]	31[19]
**15**	Vadadustat[18]	29[19]

[a] Mean average of two independent runs (n = 2; mean ± SD). [b] Using 150 nM FIH and 5.0 µM HIF-1α C-terminal transactivation domain fragment (HIF-$1\alpha_{788\text{-}822}$). Enzyme inhibition assays were performed as described in Section 3 of the Supporting information.

Supporting Table S2. Crystallization conditions, data collection, and refinement statistics for the FIH:inhibitor complexes [a].

	FIH·Zn^{II}·BNS (FIH:BNS)	FIH·Zn^{II}·**20** (FIH:**20**)	FIH·Zn^{II}·**26** (FIH:**26**)
**PDB ID**	8K71	8K72	8K73
**Crystallization**			
Precipitation conditions	0.27 mM FIH, 0.5 mM zinc acetate, 2 mM BNS, 0.1 M HEPES, pH 7.5, 6%$_{w/v}$ PEG400, 1.6 M ammonium sulfate	0.27 mM FIH, 0.5 mM zinc acetate, 2 mM **20**, 0.1 M HEPES, pH 7.5, 6%$_{w/v}$ PEG400, 1.6 M ammonium sulfate	0.27 mM FIH, 0.5 mM zinc acetate, 2 mM **26**, 0.1 M HEPES, pH 7.5, 6%$_{w/v}$ PEG400, 1.6 M ammonium sulfate
**Data collection**			
Space group	$P4_12_12$	$P4_12_12$	$P4_12_12$
Cell dimensions:			
a, *b*, *c* (Å)	86.59, 86.59, 146.50	86.61, 86.61, 145.05	86.61, 86.61, 145.26
α, β, γ (°)	90.00, 90.00, 90.00	90.00, 90.00, 90.00	90.00, 90.00, 90.00
X-Ray source[b]	Synchrotron (DLS I03)	Synchrotron (DLS I03)	Synchrotron (DLS I03)
Resolution (Å)[c]	56.59-2.23 (2.27-2.23)	72.53-2.45 (2.49-2.45)	74.39-2.02 (2.07-2.02)
R_{merge}	0.127 (4.050)	0.168 (5.078)	0.0787 (4.315)
I / σI	13.5 (0.5)	15.1 (0.6)	21.8 (0.4)
CC (1/2)	0.999 (0.370)	1.000 (0.533)	1.000 (0.395)
Total number of reflections	732894 (36387)	557824 (26550)	982479 (95111)
Total number unique reflections	27886 (1363)	21056 (1039)	36992 (3604)
Completeness (%)	100.0 (99.5)	100.0 (99.9)	99.81 (98.61)
Multiplicity	26.3 (26.7)	26.5 (25.6)	26.6 (26.4)
**Refinement**			
R_{work} / R_{free}	0.200 / 0.236	0.200 / 0.239	0.201 / 0.223
No. atoms:	2958	2937	2993
B-factors:	83.0	92.8	76.5
R.m.s. deviations:			
Bond lengths (Å)	0.002	0.003	0.003
Bond angles (°)	0.550	0.564	0.589

[a] Experimental details are described in Section 3 of the Supporting Information. [b] DLS: Diamond Light Source. [c] Values in parentheses are for highest-resolution shell.

7. General synthesis information

All reagents were purchased from commercial sources (Sigma-Aldrich, Inc.; Fluorochem Ltd; Tokyo Chemical Industries) and used as received. Anhydrous solvents (Sigma-Aldrich, Inc.) were kept under an atmosphere of nitrogen. Purifications were performed using a Biotage Isolera One purification machine or a Biotage Selekt purification machine (wavelengths monitored: 254 and 280 nm) equipped with pre-packed Biotage® Sfär Duo flash chromatography cartridges. The cartridge type and size as well as solvent gradients (in column volumes, CV) used, are specified in the individual experimental procedures. HPLC grade solvents (Sigma-Aldrich Inc.) were used for purifications, reaction work-ups, and extractions.

Thin layer chromatography (TLC) was carried out using Merck silica gel 60 F254 TLC plates and visualized using UV light. Melting points (m.p.) were determined using a Stuart SMP-40 automated melting point apparatus. Infrared (IR) spectroscopy was performed using a Bruker Tensor-27 Fourier transform infrared (FT-IR) spectrometer. High-resolution mass spectrometry (HRMS) was performed using electrospray ionization (ESI) mass spectrometry (MS) in the positive or negative ionization mode employing a Thermo Scientific Exactive mass spectrometer (ThermoFisher Scientific); data are presented as a mass-to-charge ratio (*m/z*).

Nuclear magnetic resonance (NMR) spectroscopy was performed using a Bruker AVANCE AVIIIHD 600 machine equipped with a 5 mm BB-F/1H Prodigy N_2 cryoprobe. Chemical shifts for ^{1}H NMR are reported in parts per million (ppm) downfield from tetramethylsilane and are referenced to the residual protium in the NMR solvent ($CDCl_3$: δ = 7.26 ppm; DMSO-d_6: δ = 2.50 ppm). For ^{13}C NMR, chemical shifts are reported in the scale relative to the NMR solvent ($CDCl_3$: δ = 77.2 ppm; DMSO-d_6: δ = 39.5 ppm). For ^{19}F NMR, chemical shifts are reported in the scale relative to $CFCl_3$. NMR data are reported as follows: chemical shift, multiplicity (s: singlet, d: doublet, dd: doublet of doublets, t: triplet, q: quartet, m: multiplet, br: broad signal), coupling constant (*J*, Hz; accurate to 0.5 Hz), and integration. All compounds are >95% pure by NMR, NMR spectra are shown in Section 10 of the Supporting Information.

2-(*N*-Phenylsulfamoyl)acetic acid[58] and *N*-(*tert*-butoxycarbonyl)-*N*-phenylglycine[59] were synthesised as previously reported.

8. General synthetic procedures

General Procedure A. To a solution of carboxylic acid (1.2 equiv.) and amine (1.0 equiv.) in anhydrous dimethylformamide (0.2 M) were sequentially added redistilled anhydrous *N*,*N*-diisopropylethylamine (3.0 equiv.) and 1-propanephosphonic anhydride[31] (T3P, 50%$_{w/w}$ in ethyl acetate, 1.3 eq.) dropwise at 0 °C under an atmosphere of N_2 gas. The reaction mixture was stirred and allowed to slowly warm to ambient temperature overnight (12-14 h). The solvent was removed under reduced pressure (water bath temperature = 50 °C) and the crude residue was redissolved with ethyl acetate and washed with 1M aqueous HCl solution, saturated aqueous $NaHCO_3$ solution and brine, dried over anhydrous Na_2SO_4, filtered, and evaporated. The crude residue was purified using column chromatography to afford the desired amide.

General Procedure B. To a solution of thiazole (1.0 equiv.) in chloroform (0.1 M; HPLC grade) was added 3-chloroperbenzoic acid (mCPBA, 2.2 equiv.) under an ambient atmosphere at room temperature. The reaction mixture was stirred vigorously for 2 h. The solvent was removed under reduced pressure and the crude residue was purified using column chromatography to afford the desired *N*-hydroxythiazole.

The *N*-hydroxythiazoles described herein are putatively assigned as the (*Z*)-configuration based on a previously reported *N*-hydroxythiazole small-molecule crystal structure[3] and the FIH:*N*-hydroxythiazole complex structures described in this work.

General Procedure C. To a solution of ethyl ester (1.0 equiv.) in methanol (0.2 M; HPLC grade) was added 0.4 M aqueous lithium hydroxide solution (2.5 equiv.) under an ambient atmosphere at 0 °C. The reaction mixture was allowed to warm to ambient temperature and stirred for 2 h. The methanol was removed under reduced pressure and the remaining aqueous reaction mixture was extracted three times with dichloromethane (the organic extracts were discarded). The aqueous phase was acidified (pH 4 to 5) with the dropwise addition of 1 N aqueous HCl solution. The water was removed under reduced pressure and the crude residue was purified using either reverse-phase column chromatography or trituration to afford the desired carboxylic acid.

General Procedure D. To *N*-Boc protected amine (1.0 equiv.) under an atmosphere of argon gas at 0 °C was added 4M HCl/dioxane (10.0 equiv.) The reaction mixture was allowed to warm to ambient temperature and stirred for 1 h. The solvent was removed under reduced pressure to afford the desired amine HCl salt, which was used in the subsequent step without further purification.

General Procedure E. To a solution of amine HCl salt (1.0 equiv.) in anhydrous dichloromethane (0.2 M) at 0 °C under an atmosphere of N_2 gas were sequentially added anhydrous triethylamine (2.2 equiv.) and sulfonyl chloride (1.2 equiv.). The reaction mixture was stirred and allowed to slowly warm to ambient temperature overnight (12-14 h). The crude reaction mixture was diluted with ethyl acetate and washed with 1M aqueous HCl solution, saturated aqueous $NaHCO_3$ solution and brine, dried over anhydrous Na_2SO_4, filtered, and evaporated. The crude residue was purified using column chromatography to afford the desired sulfonamide.

9. Synthetic procedures and compound characterisations

Ethyl 2-(2-(2-(phenylsulfonyl)acetamido)thiazol-4-yl)acetate (2)

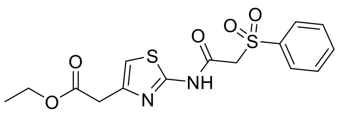

According to General Procedure A, amide **2** (1.81 g, 4.9 mmol, 82 %) was obtained from 2-(phenylsulfonyl)acetic acid (1.44 g, 7.2 mmol) and ethyl 2-(2-aminothiazol-4-yl)acetate **1** (1.12 g, 6.0 mmol), following column chromatography (50 g Sfär Silica D; 100 mL/min; 100% cyclohexane (2 CV), followed by a linear gradient (12 CV): 0%→35% ethyl acetate in cyclohexane).

White solid, m.p.: 91-92 °C; ^{1}H NMR (600 MHz, 300K, $CDCl_3$): δ = 9.35 (br s, 1H), 7.93 (d, *J* = 7.5 Hz, 2H), 7.67 (t, *J* = 7.5 Hz, 1H), 7.57 – 7.53 (m, 2H), 6.83 (s, 1H), 4.35 (s, 2H), 4.16 (q, *J* = 7.0 Hz, 2H), 3.71 (s, 2H), 1.24 (t, *J* = 7.0 Hz, 3H) ppm; ^{13}C NMR (151 MHz, 300K, $CDCl_3$): δ = 170.5, 159.0, 157.0, 143.8, 138.0, 134.8, 129.6, 128.6, 111.7, 62.1, 61.4, 36.9, 14.3 ppm; IR (film): $\tilde{\nu}$ = 3267, 2984, 1732, 1689, 1556, 1311, 1155 cm^{-1}; HRMS (ESI): *m/z* calcd for $C_{15}H_{17}O_5N_2S_2$ [*M*+H]$^+$: 369.0573, found: 369.0568.

Ethyl (*Z*)-2-(3-hydroxy-2-((2-(phenylsulfonyl)acetyl)imino)-2,3-dihydrothiazol-4-yl)acetate (3)

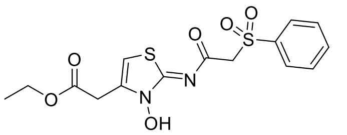

According to General Procedure B, *N*-hydroxythiazole **3** (201 mg, 0.52 mmol, 71%) was obtained from thiazole **2** (268 mg, 0.73 mmol), following column chromatography (10 g Sfär Silica D; 35 mL/min; 100% dichloromethane (3 CV), followed by a linear gradient (20 CV): 0%→4% methanol in dichloromethane).

Note: The *N*-hydroxythiazole C2 signal was not visible in the ^{13}C spectrum, due to low sample concentration. The corresponding signal (at 147.8 ppm) was assigned using HMBC.

Yellow solid, m.p.: 93-95 °C; ^{1}H NMR (600 MHz, DMSO-d_6): δ - 7.92 (d, *J* = 7.0 Hz, 2H), 7.78 – 7.72 (m, 1H), 7.67 – 7.64 (m, 2H), 7.18 (s, 1H), 4.72 (s, 2H), 4.10 (q, *J* = 7.0 Hz, 2H), 3.77 (s, 2H), 1.18 (t, *J* = 7.0 Hz, 3H) ppm; ^{13}C NMR (151 MHz, DMSO-d_6): δ = 168.4, 163.4 (br), 147.8*, 139.6, 135.3, 134.0, 129.2, 128.0, 107.4, 61.7, 60.7, 31.9, 14.0 ppm; IR (film): $\tilde{\nu}$ = 2985, 1735, 1689, 1562, 1355, 1156 cm^{-1}; HRMS (ESI): *m/z* calcd for $C_{15}H_{17}O_6N_2S_2$ [*M*+H]$^+$: 385.0523, found: 385.0516.

(*Z*)-2-(3-Hydroxy-2-((2-(phenylsulfonyl)acetyl)imino)-2,3-dihydrothiazol-4-yl)acetic acid (4)

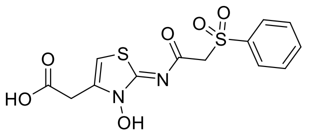

According to General Procedure C, carboxylic acid **4** (40 mg, 0.11 mmol, 43%) was obtained from ethyl ester **3** (100 mg, 0.26 mmol), following trituration with H_2O (3 × 5 mL; Milli-Q® Ultrapure grade) and MeOH (3 × 5 mL; HPLC grade).

White solid, m.p.: 179-181 °C; ^{1}H NMR (600 MHz, 300K, DMSO-d_6): δ = 7.91 (d, *J* = 8.0 Hz, 2H), 7.75 (t, *J* = 8.0 Hz, 1H), 7.68 – 7.64 (m, 2H), 7.18 (s, 1H), 4.74 (s, 2H), 3.71 (s, 2H) ppm; ^{13}C NMR (151 MHz, 300K, DMSO-d_6): δ = 169.6, 163.2 (br), 147.4 (br), 139.5, 135.6, 134.0, 129.2, 128.0, 107.4, 61.6, 32.2 ppm; IR (film): $\tilde{\nu}$ = 3005, 1691, 1562, 1308, 1233, 1164 cm^{-1}; HRMS (ESI): *m/z* calcd for $C_{13}H_{13}O_6N_2S_2$ [*M*+H]$^+$: 357.0210, found: 357.0211.

Ethyl 2-(2-amino-5-methylthiazol-4-yl)acetate (5)

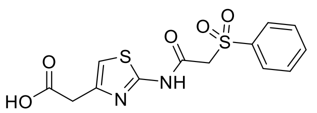

To a solution of ethyl ester **2** (2.90 g, 7.9 mmol, 1.0 equiv.) in methanol (50 mL; HPLC grade) under an ambient atmosphere at 0 °C was added lithium hydroxide (472 mg, 19.7 mmol, 2.5 equiv.) in water (50 mL; Milli-

Q® Ultrapure grade). The reaction mixture was allowed to warm to ambient temperature and stirred for 14 h. The methanol was removed under reduced pressure and the remaining aqueous solution was washed three times with dichloromethane (the organic extracts were discarded). The aqueous phase was acidified (pH 4 – 5) by the dropwise addition of 4 N aqueous HCl solution. The aqueous phase was extracted three times with chloroform/2-propanol (3:1). The combined organic extracts were dried over anhydrous Na_2SO_4, filtered, and evaporated. The crude residue was purified using reverse-phase column chromatography (60 g Sfär C18 Duo; 50 mL/min; 100% water (+ 0.1% (*v/v*) formic acid) (4 CV), followed by a linear gradient (25 CV): 0%→100% acetonitrile (+ 0.1% (*v/v*) formic acid) in water (+ 0.1% (*v/v*) formic acid)) and lyophilized to afford carboxylic acid **5** (2.02 g, 5.93 mmol, 75%).

White solid, m.p.: 180-182 °C; ^{1}H NMR (600 MHz, 300K, DMSO-d_6): δ = 12.45 (br s, 2H), 7.94 – 7.86 (m, 2H), 7.77 (t, *J* = 7.5 Hz, 1H), 7.69 – 7.64 (m, 2H), 7.01 (s, 1H), 4.59 (s, 2H), 3.60 (s, 2H) ppm; ^{13}C NMR (151 MHz, 300K, DMSO-d_6): δ = 171.5, 159.7, 156.6, 144.5, 139.1, 134.2, 129.3, 128.0, 110.9, 60.7, 36.7 ppm; IR (film): $\tilde{\nu}$ = 2972, 1688, 1572, 1308, 1157, 1084 cm^{-1}; HRMS (ESI): *m/z* calcd for $C_{13}H_{13}N_2O_5S_2$ [*M*+H]$^+$: 341.0260, found: 341.0272.

(±)-Ethyl 2-(2-aminothiazol-4-yl)propanoate (6b)

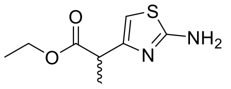

To a solution of ethyl 2-methyl-3-oxobutanoate **6a** (1.31 mL, 1.44 g, 10.0 mmol, 1.0 equiv.) in chloroform (25 mL; HPLC grade) under an ambient atmosphere at room temperature was added pyridinium tribromide (3.20 g, 10.0 mmol, 1.0 equiv.). The reaction mixture was stirred at 40 °C in the dark for 2 h. The solvent was removed under reduced pressure. The crude residue was re-dissolved in ethanol (25 mL; HPLC grade) and thiourea (761 mg, 10.0 mmol, 1.0 equiv.) was added. The reaction mixture was heated under reflux for 2 h before the solvent was removed under reduced pressure. The residue was dissolved in ethyl acetate and washed twice with saturated aqueous $NaHCO_3$ solution and brine, dried over anhydrous Na_2SO_4, filtered, and evaporated. The crude residue was purified using column chromatography (50 g Sfär Silica D; 120 mL/min; 100% cyclohexane (2 CV), followed by a linear gradient (12 CV): 0%→50% ethyl acetate (+1% (*v/v*) Et_3N) in cyclohexane) to afford racemic 2-aminothiazole **6b** (901 mg, 4.51 mmol, 45%).

Yellow solid, m.p.: 106-107 °C; ^{1}H NMR (600 MHz, 300K, DMSO-d_6): δ = 6.93 (s, 2H), 6.27 (s, 1H), 4.10 – 3.98 (m, 2H), 3.58 (q, *J* = 7.0 Hz, 1H), 1.31 (d, *J* = 7.0 Hz, 3H), 1.15 (t, *J* = 7.0 Hz, 3H) ppm; ^{13}C NMR (151 MHz, 300K, DMSO-d_6): δ = 173.0, 168.3, 150.3, 101.3, 60.1, 41.6, 16.5, 14.1 ppm; IR (film): $\tilde{\nu}$ = 3419, 3133, 1722, 1630, 1531, 1193 cm^{-1}; HRMS (ESI): *m/z* calcd for $C_8H_{13}O_2N_2S$ [*M*+H]$^+$: 201.0692, found: 201.0691.

The analytical data is consistent with the literature.[60]

(±)-Ethyl 2-(2-(2-(phenylsulfonyl)acetamido)thiazol-4-yl)propanoate (6c)

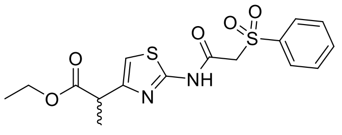

According to General Procedure A, racemic amide **6c** (395 mg, 1.0 mmol, 83%) was obtained from 2-aminothiazole **6b** (250 mg, 1.3 mmol) and 2-(phenylsulfonyl)acetic acid (300 mg, 1.5 mmol), following column chromatography (25 g Sfär Silica D; 60 mL/min; 100% cyclohexane (2 CV), followed by a linear gradient (12 CV): 0%→40% ethyl acetate in cyclohexane).

Yellow oil; ^{1}H NMR (600 MHz, 300K, $CDCl_3$): δ = 7.94 (d, *J* = 7.5 Hz, 2H), 7.70 (t, *J* = 7.5 Hz, 1H), 7.61 – 7.56 (m, 2H), 6.81 (s, 1H), 4.32 (s, 2H), 4.22 – 4.14 (m, 2H), 3.92 – 3.83 (m, 1H), 1.54 (d, *J* = 7.0 Hz, 3H), 1.26 (t, *J* = 7.0 Hz, 3H) ppm; ^{13}C NMR (151 MHz, 300K, $CDCl_3$): δ = 173.3, 158.9, 157.1, 149.2, 138.0,

134.9, 129.8, 128.5, 109.9, 62.0, 61.4, 41.9, 17.1, 14.3 ppm; IR (film): ṽ = 2982, 1729, 1690, 1554, 1326, 1156, 1083 cm^{-1}; HRMS (ESI): *m/z* calcd for $C_{16}H_{19}N_2O_5S_2$ [*M*+H]$^+$: 383.0730, found: 383.0739.

(±)-Ethyl (*Z*)-2-(3-hydroxy-2-((2-(phenylsulfonyl)acetyl)imino)-2,3-dihydrothiazol-4-yl)propanoate (6d)

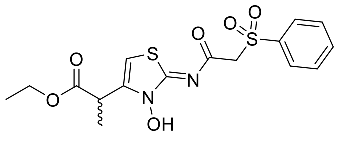

According to General Procedure B, racemic *N*-hydroxythiazole **6d** (146 mg, 0.37 mmol, 39%) was obtained from thiazole **6c** (359 mg, 0.94 mmol), following column chromatography (10 g Sfär Silica D; 35 mL/min; 100% dichloromethane (2 CV), followed by a linear gradient (20 CV): 0%→5% methanol in dichloromethane).

Yellow solid, m.p.: 143-144 °C; ^{1}H NMR (600 MHz, 300K, DMSO-d_6): δ = 7.95 – 7.88 (m, 2H), 7.75 (t, *J* = 7.5 Hz, 1H), 7.67 – 6.63 (m, 2H), 7.16 (s, 1H), 4.73 (s, 2H), 4.10 – 4.06 (m, 2H), 3.92 (q, *J* = 7.0 Hz, 1H), 1.44 (d, *J* = 7.5 Hz, 3H), 1.15 (t, *J* = 7.0 Hz, 3H) ppm; ^{13}C NMR (151 MHz, 300K, DMSO-d_6): δ = 171.4, 163.2 (br), 147.0 (br), 140.7, 139.5, 134.0, 129.2, 128.0, 105.8, 61.6, 60.6, 37.6, 14.8, 14.0 ppm; IR (film): ṽ = 2981, 1735, 1687, 1586, 1327, 1157, 1084 cm^{-1}; HRMS (ESI): *m/z* calcd for $C_{16}H_{18}N_2O_6S_2K$ [*M*+K]$^+$: 437.0238, found: 437.0244.

(±)-(*Z*)-2-(3-Hydroxy-2-((2-(phenylsulfonyl)acetyl)imino)-2,3-dihydrothiazol-4-yl)propanoic acid (6)

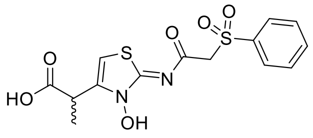

According to General Procedure C, racemic carboxylic acid **6** (28 mg, 0.076 mmol, 58%) was obtained from ethyl ester **6d** (50 mg, 0.13 mmol), following trituration with H_2O (3 × 5 mL; Milli-Q® Ultrapure grade) and MeOH (3 × 5 mL; HPLC grade).

White solid, m.p.: 204-207 °C; ^{1}H NMR (600 MHz, 300K, DMSO-d_6): δ = 7.96 – 7.88 (m, 2H), 7.75 (t, *J* = 7.5 Hz, 1H), 7.68 – 7.64 (m, 2H), 7.16 (s, 1H), 4.76 (s, 2H), 3.90 (q, *J* = 7.0 Hz, 1H), 1.43 (d, *J* = 7.5 Hz, 3H) ppm; ^{13}C NMR (151 MHz, 300K, DMSO-d_6): δ = 172.5, 163.0 (br), 147.1 (br), 140.8, 139.5, 134.1, 129.2, 128.0, 105.8, 61.5, 37.7, 14.7 ppm; IR (film): ṽ = 2926, 1697, 1563, 1310, 1227, 1162 cm^{-1}; HRMS (ESI): *m/z* calcd for $C_{14}H_{14}N_2O_6S_2Na$ [*M*+Na]$^+$: 393.0186, found: 393.0195.

Ethyl 2-(2-amino-5-methylthiazol-4-yl)acetate (7b)

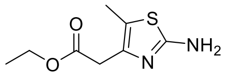

To a solution of ethyl 3-oxopentanoate **7a** (1.42 mL, 1.44 g, 10.0 mmol, 1.0 equiv.) in chloroform (25 mL; HPLC grade) under an ambient atmosphere at room temperature was added pyridinium tribromide (3.20 g, 10.0 mmol, 1.0 equiv.). The reaction mixture was stirred at 40 °C in the dark for 2 h before the solvent was removed under reduced pressure. The crude residue was re-dissolved in ethanol (25 mL; HPLC grade) and thiourea (761 mg, 10.0 mmol, 1.0 equiv.) was added. The reaction mixture was stirred vigorously under reflux for 2 h. The solvent was removed under reduced pressure. The crude residue was dissolved in ethyl acetate and washed twice with saturated aqueous $NaHCO_3$ solution and brine, dried over anhydrous Na_2SO_4, filtered, and evaporated. The crude residue was purified using column chromatography (50 g Sfär Silica D; 120 mL/min; 100% cyclohexane (2 CV), followed by a linear gradient (12 CV): 0%→50% ethyl acetate in cyclohexane) to afford 2-aminothiazole **7b** (1.39 g, 6.94 mmol, 69%).

Yellow solid, m.p.: 118-120 °C; ^{1}H NMR (600 MHz, 300K, $CDCl_3$): δ = 4.16 (q, *J* = 7.0 Hz, 2H), 3.48 (s, 2H), 2.22 (s, 3H), 1.26 (t, *J* = 7.0 Hz, 3H) ppm; ^{13}C NMR (151 MHz, 300K, $CDCl_3$): δ = 170.6, 164.6, 138.9, 118.9, 61.1, 34.8, 14.3, 11.2 ppm; IR (film): ṽ = 3409, 3141, 1722, 1630, 1531, 1273, 1032 cm^{-1}; HRMS (ESI): *m/z* calcd for $C_8H_{13}N_2O_2S$ [M+H]$^+$: 201.0692, found: 201.0692.

The analytical data is consistent with the literature.[60]

Ethyl 2-(5-methyl-2-(2-(phenylsulfonyl)acetamido)thiazol-4-yl)acetate (7c)

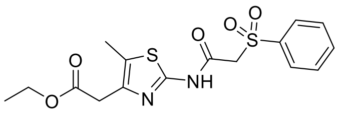

According to General Procedure A, amide **7c** (410 mg, 1.1 mmol, 86%) was obtained from 2-aminothiazole **7b** (250 mg, 1.3 mmol) and 2-(phenylsulfonyl)acetic acid (300 mg, 1.5 mmol), following column chromatography (25 g Sfär Silica D; 60 mL/min; 10% ethyl acetate in cyclohexane (2 CV), followed by a linear gradient (12 CV): 10%→60% ethyl acetate in cyclohexane).

Yellow oil; ^{1}H NMR (600 MHz, 300K, DMSO-d_6): δ = 12.33 (s, 1H), 7.89 (d, *J* = 7.5 Hz, 2H), 7.77 (t, *J* = 7.5 Hz, 1H), 7.69 – 7.65 (m, 2H), 4.56 (s, 2H), 4.06 (q, *J* = 7.0 Hz, 2H), 3.61 (s, 2H), 2.27 (s, 3H), 1.17 (t, *J* = 7.0 Hz, 3H) ppm; ^{13}C NMR (151 MHz, 300K, DMSO-d_6): δ = 170.0, 159.4, 153.1, 139.4, 139.0, 134.2, 129.3, 128.0, 122.7, 60.7, 60.3, 34.3, 14.1, 10.3 ppm; IR (film): $\tilde{\nu}$ = 2981, 1733, 1687, 1562, 1267, 1157 cm^{-1}; HRMS (ESI): *m/z* calcd for $C_{16}H_{18}N_2O_5S_2Na$ [*M*+Na]$^+$: 405.0549, found: 405.0557.

Ethyl (*Z*)-2-(3-hydroxy-5-methyl-2-((2-(phenylsulfonyl)acetyl)imino)-2,3-dihydrothiazol-4-yl)acetate (7d)

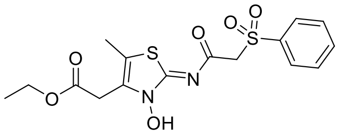

According to General Procedure B, *N*-hydroxythiazole **7d** (161 mg, 0.40 mmol, 43%) was obtained from thiazole **7c** (356 mg, 0.93 mmol), following column chromatography (10 g Sfär Silica D; 35 mL/min; 100% dichloromethane (2 CV), followed by a linear gradient (20 CV): 0%→5% methanol in dichloromethane).

Note: ^{1}H and ^{13}C NMR spectra were acquired at 323 K due to significant peak broadening at ambient temperature. The *N*-hydroxythiazole *C*2 signal was not visible in the ^{13}C spectrum due to low sample concentration. The corresponding signal in other *N*-hydroxythiazole compounds is very broad in DMSO-d_6 and is observed between 140 and 160 ppm.

White solid, m.p.: 170-172 °C; ^{1}H NMR (600 MHz, 323 K, DMSO-d_6): δ = 7.93 (d, *J* = 7.5 Hz, 2H), 7.73 (t, *J* = 7.5 Hz, 1H), 7.64 – 7.60 (m, 2H), 4.63 (s, 2H), 4.12 – 3.99 (m, 2H), 3.73 (s, 2H), 2.24 (s, 3H), 1.17 (t, *J* = 7.0 Hz, 3H) ppm; ^{13}C NMR (151 MHz, 323 K, DMSO-d_6): δ = 168.3, 164.1 (br), 139.6, 133.6, 130.6 (br), 128.9, 127.8, 117.3 (br), 62.3 (br), 60.4, 29.7, 13.8, 11.4 ppm; IR (film): $\tilde{\nu}$ = 2971, 1741, 1684, 1557, 1326, 1156, 1084 cm^{-1}; HRMS (ESI): *m/z* calcd for $C_{16}H_{18}N_2O_6S_2Na$ [*M*+Na]$^+$: 421.0499, found: 421.0509.

(*Z*)-2-(3-Hydroxy-5-methyl-2-((2-(phenylsulfonyl)acetyl)imino)-2,3-dihydrothiazol-4-yl)acetic acid (7)

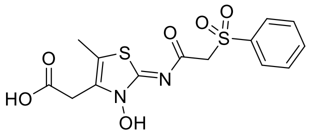

According to General Procedure C, carboxylic acid **7** (9 mg, 0.024 mmol, 27%) was obtained from ethyl ester **7d** (36 mg, 0.09 mmol), following trituration with H_2O (3 × 5 mL; Milli-Q® Ultrapure grade) and MeOH (3 × 5 mL; HPLC grade).

White solid, m.p.: 181-182 °C; ^{1}H NMR (600 MHz, 300K, DMSO-d_6): δ = 7.91 (d, *J* = 7.5 Hz, 2H), 7.75 (t, *J* = 7.5 Hz, 1H), 7.67 – 7.64 (m, 2H), 4.71 (s, 2H), 3.69 (s, 2H), 2.28 (s, 3H) ppm; ^{13}C NMR (151 MHz, 300K, DMSO-d_6): δ = 169.7, 163.0 (br), 144.3 (br), 139.5, 134.0, 131.7 (br), 129.2, 128.0, 118.1 (br), 61.6, 30.2, 11.5 ppm; IR (film): $\tilde{\nu}$ = 3072, 1697, 1566, 1311, 1227, 1151 cm^{-1}; HRMS (ESI): *m/z* calcd for $C_{14}H_{15}N_2O_6S_2$ [*M*+H]$^+$: 371.0366, found: 371.0376.

2-Chloro-*N*-(thiazol-2-yl)acetamide (8b)

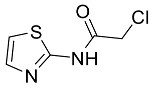

To a solution of 2-aminothiazole **8a** (1.00 g, 10.0 mmol, 1.0 equiv.) and potassium carbonate (3.46 g, 25.0 mmol, 2.5 equiv.) in anhydrous dichloromethane (40 mL) under an atmosphere of N_2 gas at 0 °C was added 2-chloroacetyl chloride (0.88 mL, 1.24 g, 11.0 mmol, 1.1 equiv.) dropwise. The reaction mixture was allowed to warm to ambient temperature and stirred for 14 h. The reaction mixture was diluted with dichloromethane and washed with water and brine. The organic phase was dried over anhydrous Na_2SO_4, filtered, and evaporated. The crude residue was purified using column chromatography (50 g Sfär Silica D; 120 mL/min; 100% cyclohexane (2 CV), followed by a linear gradient (12 CV): 0% → 30% acetone in cyclohexane) to afford 2-chloroacetamide **8b** (1.11 g, 6.27 mmol, 63%).

White solid, m.p.: 196-197 °C; ^{1}H NMR (600 MHz, 300K, $CDCl_3$): δ = 7.52 (d, *J* = 3.5 Hz, 1H), 7.06 (d, *J* = 3.5 Hz, 1H), 4.28 (s, 2H) ppm; ^{13}C NMR (151 MHz, 300K, $CDCl_3$): δ = 164.1, 157.9, 137.6, 114.6, 42.1 ppm; IR (film): $\tilde{\nu}$ = 2951, 1703, 1583, 1329, 1192, 1165 cm^{-1}; HRMS (ESI): *m/z* calcd for $C_5H_6ClN_2OS$ $[M+H]^+$: 176.9884, found: 176.9885.

The analytical data is consistent with the literature.[61]

2-(Phenylsulfonyl)-*N*-(thiazol-2-yl)acetamide (8c)

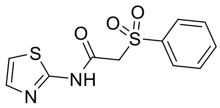

A mixture of 2-chloroacetamide **8b** (1.10 g, 6.3 mmol, 1.0 equiv.) and sodium benzenesulfinate (2.06 g, 12.5 mmol, 2.5 equiv.) in anhydrous ethanol (25 mL) under an atmosphere of N_2 gas was heated under reflux for 14 h. The solvent was removed under reduced pressure and the residue was dissolved in ethyl acetate (50 mL), washed with H_2O and saturated NaCl solution, dried over anhydrous Na_2SO_4, filtered, and evaporated. The crude residue was purified using column chromatography (50 g Sfär Silica D; 120 mL/min; 100% cyclohexane (2 CV), followed by a linear gradient (12 CV): 0% → 40% acetone in cyclohexane) to afford sulfone **8c** (221 mg, 0.78 mmol, 12%).

White solid, m.p.: 230-233 °C; ^{1}H NMR (600 MHz, 300K, DMSO-d_6): δ = 12.42 (br s, 1H), 7.90 (d, *J* = 8.0 Hz, 2H), 7.77 (t, *J* = 8.0 Hz, 1H), 7.69 – 7.65 (m, 2H), 7.50 (d, *J* = 3.5 Hz, 1H), 7.27 (d, *J* = 3.5 Hz, 1H), 4.63 (s, 2H) ppm; ^{13}C NMR (151 MHz, 300K, DMSO-d_6): δ = 159.7, 157.1, 139.1, 137.9, 134.2, 129.3, 128.0, 114.2, 60.7 ppm; IR (film): $\tilde{\nu}$ = 2981, 1685, 1567, 1309, 1159, 1084 cm^{-1}; HRMS (ESI): *m/z* calcd for $C_{11}H_{10}N_2O_3S_2Na$ $[M+Na]^+$: 305.0025, found: 305.0035.

(*Z*)-*N*-(3-Hydroxythiazol-2(3*H*)-ylidene)-2-(phenylsulfonyl)acetamide (8)

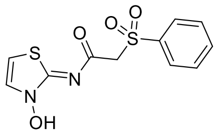

According to General Procedure B, *N*-hydroxythiazole **8** (21 mg, 0.07 mmol, 25%) was obtained from thiazole **8c** (80 mg, 0.28 mmol), following reverse-phase column chromatography (12 g Sfär C18 Duo; 12 mL/min; 100% water (+ 0.1% (*v/v*) formic acid) (4 CV), followed by a linear gradient (25 CV): 0%→100% acetonitrile (+ 0.1% (*v/v*) formic acid) in water (+ 0.1% (*v/v*) formic acid)).

Note: The *N*-hydroxythiazole C2 signal was not visible in the ^{13}C spectrum, due to low sample concentration. The corresponding signal (at 155.6 ppm) was assigned using HMBC.

Yellow solid, m.p.: 89-93 °C; ^{1}H NMR (600 MHz, 300K, DMSO-d_6): δ = 7.91 (d, *J* = 8.0 Hz, 2H), 7.73 (t, *J* = 8.0 Hz, 1H), 7.70 (d, *J* = 4.5 Hz, 1H), 7.66 – 7.61 (m, 2H), 7.15 (d, *J* = 4.5 Hz, 1H), 4.59 (s, 2H) ppm; ^{13}C NMR (151 MHz, 300K, DMSO-d_6): δ = 166.5 (br), 155.6*, 139.8, 133.8, 129.1, 128.6 (br), 128.0, 108.3,

63.2 ppm; IR (film): ṽ = 1684, 1567, 1352, 1323, 1155, 1084 cm^{-1}; HRMS (ESI): *m/z* calcd for $C_{11}H_{11}N_2O_4S_2$ [*M*+H]$^+$: 299.0155, found: 299.0163.

Methyl (*Z*)-2-(3-hydroxy-2-((2-(phenylsulfonyl)acetyl)imino)-2,3-dihydrothiazol-4-yl)acetate (9)

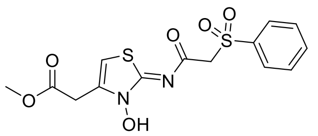

To a solution of ethyl ester **3** (174 mg, 0.50 mmol, 1.0 equiv.) in anhydrous methanol (2.5 mL) under an atmosphere of N_2 gas at 0 °C was added sodium methoxide (54 mg, 1.0 mmol, 2.0 equiv.). The reaction mixture was allowed to warm to ambient temperature and stirred for 14 h. The methanol was removed under reduced pressure and the crude residue was purified using reverse-phase column chromatography (30 g Sfär C18 Duo; 25 mL/min; 100% water (+ 0.1% (*v/v*) formic acid) (4 CV), followed by a linear gradient (25 CV): 0%→100% acetonitrile (+ 0.1% (*v/v*) formic acid) in water (+ 0.1% (*v/v*) formic acid)) and lyophilized to afford methyl ester **9** (109 mg, 0.29 mmol, 58%).

White solid, m.p.: 81-84 °C; ^{1}H NMR (400 MHz, 300K, $CDCl_3$): δ = 7.86 (d, *J* = 8.0 Hz, 2H), 7.62 (t, *J* = 8.0 Hz, 1H), 7.50 – 7.45 (m, 2H), 7.05 (s, 1H), 4.61 (s, 2H), 3.94 (s, 2H), 3.77 (s, 3H) ppm; ^{13}C NMR (151 MHz, 300K, $CDCl_3$): δ = 168.9, 160.5 (br), 143.8 (br), 138.4, 136.8 (br), 134.5, 129.4, 128.7, 108.1, 62.0, 52.8, 32.1 ppm; IR (film): ṽ = 1739, 1686, 1552, 1325, 1155, 1084 cm^{-1}; HRMS (ESI): *m/z* calcd for $C_{14}H_{15}N_2O_6S_2$ [*M*+H]$^+$: 371.0366, found: 371.0370.

***N*-Methyl-2-(2-(2-(phenylsulfonyl)acetamido)thiazol-4-yl)acetamide (10a)**

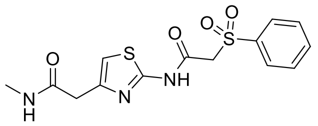

To a solution of carboxylic acid **5** (340 mg, 1.0 mmol, 1.0 equiv.) and methylamine hydrochloride (81 mg, 1.2 mmol, 1.2 equiv.) in anhydrous *N*,*N*-dimethylformamide (5.0 mL) under an atmosphere of N_2 gas at 0 °C was added redistilled anhydrous *N*,*N*-diisopropylethylamine (0.52 mL, 388 mg, 3.0 mmol, 3.0 equiv.) and HATU[32] (494 mg, 1.3 mmol, 1.3 equiv.). The reaction mixture was allowed to warm to ambient temperature and stirred for 14 h. The solvent was removed under reduced pressure. The residue was dissolved in ethyl acetate and washed with 1N HCl solution, saturated aqueous $NaHCO_3$ solution and brine, dried over anhydrous Na_2SO_4, filtered, and evaporated. The crude residue was triturated with H_2O (3 × 5 mL; Milli-Q® Ultrapure grade) and MeOH (3 × 5 mL; HPLC grade) to afford *N*-methyl amide **10a** (163 mg, 0.46 mmol, 46%).

White solid, m.p.: 233-235 °C; ^{1}H NMR (600 MHz, 300K, DMSO-*d*$_6$): δ = 12.46 (br s, 1H), 7.90 (d, *J* = 8.0 Hz, 2H), 7.83 (q, *J* = 4.5 Hz, 1H), 7.77 (t, *J* = 8.0 Hz, 1H), 7.69 – 7.65 (m, 2H), 6.94 (s, 1H), 4.59 (s, 2H), 3.44 (s, 2H), 2.58 (d, *J* = 4.5 Hz, 3H) ppm; ^{13}C NMR (151 MHz, 300K, DMSO-*d*$_6$): δ = 169.2, 159.6, 156.5, 145.8, 139.1, 134.2, 129.3, 128.0, 110.4, 60.7, 38.3, 25.7 ppm; IR (film): ṽ = 3181, 1695, 1648, 1560, 1407, 1325, 1163, 1083 cm^{-1}; HRMS (ESI): *m/z* calcd for $C_{14}H_{15}N_3O_4S_2Na$ [*M*+Na]$^+$: 376.0396, found: 376.0410.

(*Z*)-2-(3-Hydroxy-2-((2-(phenylsulfonyl)acetyl)imino)-2,3-dihydrothiazol-4-yl)-*N*-methylacetamide (10)

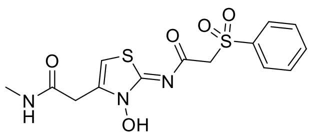

According to General Procedure B, *N*-hydroxythiazole **10** (20 mg, 0.054 mmol, 39%) was obtained from thiazole **10a** (50 mg, 0.14 mmol), following reverse-phase column chromatography (12 g Sfär C18 Duo; 12 mL/min; 100% water (+ 0.1% (*v/v*) formic acid) (4 CV), followed by a linear gradient (25 CV): 0%→100% acetonitrile (+ 0.1% (*v/v*) formic acid) in water (+ 0.1% (*v/v*) formic acid)).

White solid, m.p.: 186-189 °C; ^{1}H NMR (600 MHz, 300K, DMSO-d_6): δ = 8.15 (q, J = 5.0 Hz, 1H), 7.91 (d, J = 8.0 Hz, 2H), 7.74 (t, J = 8.0 Hz, 1H), 7.67 – 7.62 (m, 2H), 7.07 (s, 1H), 4.68 (s, 2H), 3.55 (s, 2H), 2.59 (d, J = 4.5 Hz, 3H) ppm; ^{13}C NMR (151 MHz, 300K, DMSO-d_6): δ = 167.5, 163.6 (br), 148.5 (br), 139.6, 136.2 (br), 133.9, 129.2, 128.0, 106.6, 61.9, 33.3, 25.7 ppm; IR (film): $\tilde{\nu}$ = 2933, 1691, 1641, 1543, 1326, 1159, 1082 cm^{-1}; HRMS (ESI): m/z calcd for $C_{14}H_{16}N_3O_5S_2$ [M+H]$^+$: 370.0526, found: 370.0539.

***N*-(4-(2-Amino-2-oxoethyl)thiazol-2-yl)-2-(phenylsulfonyl)acetamide (11a)**

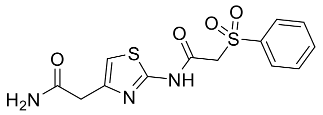

To a solution of ethyl ester **3** (3.68 g, 10.0 mmol, 1.0 equiv.) in methanol (50 mL; HPLC grade) under an ambient atmosphere at room temperature was added 35 $\%_{w/w}$ aqueous ammonia solution (55 mL, 1.0 mol, 100 equiv.). The reaction mixture was stirred at room temperature for 3 days. The solvent was removed under reduced pressure. The crude residue was purified using column chromatography (50 g Sfär Silica D; 120 mL/min; 100% dichloromethane (2 CV), followed by a linear gradient (20 CV): 0%→20% methanol in dichloromethane) to afford primary amide **11a** (2.02g, 5.96 mmol, 60%).

White solid; m.p.: > 160 °C (decomposition); ^{1}H NMR (600 MHz, 300K, DMSO-d_6): δ = 12.48 (br s, 1H), 7.90 (d, J = 8.0 Hz, 2H), 7.76 (t, J = 8.0 Hz, 1H), 7.69 – 7.65 (m, 2H), 7.38 (s, 1H), 6.99 – 6.93 (m, 2H), 4.60 (s, 2H), 3.44 (s, 2H) ppm; ^{13}C NMR (151 MHz, 300K, DMSO-d_6): δ = 171.0, 159.7, 156.6, 145.8, 139.1, 134.3, 129.4, 128.0, 110.4, 60.8, 38.1 ppm; IR (film): $\tilde{\nu}$ = 3418, 2981, 1737, 1673, 1592, 1399, 1308, 1150 cm^{-1}; HRMS (ESI): m/z calcd for $C_{13}H_{14}O_4N_3S_2$ [M+H]$^+$: 340.0420, found: 340.0421.

***N*-(4-(Cyanomethyl)thiazol-2-yl)-2-(phenylsulfonyl)acetamide (11b)**

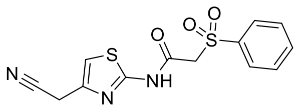

To a solution of primary amide **11a** (1.02 g, 3.0 mmol, 1.0 equiv.) in anhydrous tetrahydrofuran (15 mL) under an atmosphere of Ar gas at 0 °C was added the Burgess reagent[33] (1.79 g, 7.5 mmol, 2.5 equiv.). The reaction mixture was allowed to warm to ambient temperature and stirred for 2 h under an atmosphere of Ar gas. The solvent was removed under reduced pressure. The crude residue was purified using reverse-phase column chromatography (30 g Sfär C18 Duo; 25 mL/min; 100% water (+ 0.1% (v/v) formic acid) (4 CV), followed by a linear gradient (20 CV): 0%→100% acetonitrile (+ 0.1% (v/v) formic acid) in water (+ 0.1% (v/v) formic acid)) and lyophilized to afford nitrile **11b** (221 mg, 0.69 mmol, 23%).

Yellow solid; m.p.: > 170 °C (decomposition); ^{1}H NMR (600 MHz, 300K, DMSO-d_6): δ = 12.63 (br s, 1H), 7.90 (d, J = 8.0 Hz, 2H), 7.77 (t, J = 8.0 Hz, 1H), 7.70 – 7.65 (m, 2H), 7.13 (s, 1H), 4.60 (s, 2H), 4.04 (s, 2H) ppm; ^{13}C NMR (151 MHz, 300K, DMSO-d_6): δ = 160.0, 157.8, 140.4, 139.0, 134.2, 129.3, 128.0, 118.0, 111.2, 60.7, 19.4 ppm; IR (film): $\tilde{\nu}$ = 2923, 2360, 1688, 1571, 1405, 1324, 1156, 1025 cm^{-1}; HRMS (ESI): m/z calcd for $C_{13}H_{12}O_3N_3S_2$ [M+H]$^+$: 322.0315, found: 322.0314.

(*Z*)-*N*-(4-(Cyanomethyl)-3-hydroxythiazol-2(3*H*)-ylidene)-2-(phenyl sulfonyl)acetamide (11)

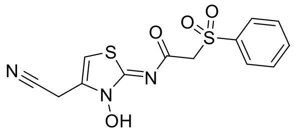

According to General Procedure B, *N*-hydroxythiazole **11** (26 mg, 0.08 mmol, 50%) was obtained from thiazole **11b** (50 mg, 0.16 mmol, 1.0 equiv.), following reverse-phase column chromatography (12 g Sfär C18 Duo; 12 mL/min; 100% water (+ 0.1% (v/v) formic acid) (4 CV), followed by a linear gradient (20 CV): 0%→100% acetonitrile (+ 0.1% (v/v) formic acid) in water (+ 0.1% (v/v) formic acid)).

White solid; m.p.: > 150 °C (decomposition); ^{1}H NMR (600 MHz, 300K, DMSO-d_6): δ = 7.91 (d, J = 8.0 Hz, 2H), 7.76 (t, J = 8.0 Hz, 1H), 7.69 – 7.63 (m, 2H), 7.34 (s, 1H), 4.78 (s, 2H), 4.09 (s, 2H) ppm; ^{13}C NMR

(151 MHz, 300K, DMSO-d_6): δ = 163.2 (br), 146.4 (br), 139.9, 134.6, 132.8 (br), 129.7, 128.5, 116.8, 108.5, 61.7, 16.2 ppm; IR (film): $\tilde{\nu}$ = 2999, 2224, 1436, 1312, 1047 cm^{-1}; HRMS (ESI): *m/z* calcd for $C_{13}H_{12}O_4N_3S_2$ [*M*+H]$^+$: 338.0264, found: 338.0265.

N-(4-(Chloromethyl)thiazol-2-yl)-2-(phenylsulfonyl)acetamide (12b)

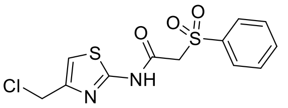

To a solution of 2-amino-4-(chloromethyl)thiazole hydrochloride **12a** (1.67 g, 9.0 mmol, 1.0 equiv.) in anhydrous dichloromethane (45 mL) under an atmosphere of N_2 gas at 0 °C was added redistilled anhydrous *N*,*N*-diisopropylethylamine (3.15 mL, 2.33 g, 18.0 mmol, 2.0 equiv.), 1-hydroxybenzotriazole hydrate (2.07 g, 13.5 mmol, 1.5 equiv.) and *N*-(3-dimethylaminopropyl)-*N*′-ethylcarbodiimide hydrochloride (2.59 g, 13.5 mmol, 1.5 equiv.). The reaction mixture was stirred at 0 °C for 30 min before the addition of (phenylsulfonyl)acetic acid (1.98 g, 9.9 mmol, 1.1 equiv.). The reaction mixture was allowed to warm to room temperature and stirred for 14 h before the solvent was removed under reduced pressure. The crude residue was dissolved in ethyl acetate and washed with 1 M aqueous HCl solution, saturated aqueous $NaHCO_3$ solution, and brine, dried over anhydrous Na_2SO_4, filtered, and evaporated. The crude residue was purified using column chromatography (50 g Sfär Silica D; 120 mL/min; 100% dichloromethane (2 CV), followed by a linear gradient (30 CV): 0%→40% ethyl acetate in dichloromethane) to afford amide **12b** (622 mg, 1.88 mmol, 21%).

White solid; m.p.: 172-175 °C; ^{1}H NMR (600 MHz, 300K, $CDCl_3$): δ = 10.58 (br s, 1H), 7.96 (d, *J* = 8.0 Hz, 2H), 7.70 (t, *J* = 8.0 Hz, 1H), 7.61 – 7.57 (m, 2H), 6.97 (s, 1H), 4.58 (s, 2H), 4.33 (s, 2H) ppm; ^{13}C NMR (151 MHz, 300K, $CDCl_3$): δ = 159.0, 157.5, 147.3, 137.7, 135.0, 129.8, 128.6, 113.0, 62.2, 41.0 ppm; IR (film): $\tilde{\nu}$ = 2924, 1691, 1659, 1556, 1325, 1155, 1084 cm^{-1}; HRMS (ESI): *m/z* calcd for $C_{12}H_{12}O_3N_2S_2Cl$ [*M*+H]$^+$: 330.9972, found: 330.9972.

N-(4-((1H-1,2,3-Triazol-1-yl)methyl)thiazol-2-yl)-2-(phenylsulfonyl) acetamide (12c)

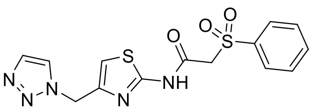

To a solution of 4-(chloromethyl)thiazole **12b** (200 mg, 0.60 mmol, 1.0 equiv.) and potassium carbonate (168 mg, 1.2 mmol, 2.0 equiv.) in anhydrous *N*,*N*-dimethylformamide (3.0 mL) under at atmosphere of N_2 gas at 0 °C was added 1*H*-1,2,3-triazole (46 mg, 0.66 mmol, 1.1 equiv.). The reaction mixture was allowed to warm to room temperature and heated for 14 h under at atmosphere of N_2 gas at 80 °C. The solvent was removed under reduced pressure. The residue was dissolved in ethyl acetate, washed with H_2O, and brine, dried over Na_2SO_4, filtered, and evaporated. The crude residue was purified using column chromatography (10 g Sfär Silica D; 35 mL/min; 100% cyclohexane (2 CV), followed by a linear gradient (30 CV): 0%→75% acetone in cyclohexane) to afford triazole **12c** (61 mg, 0.17 mmol, 28%).

Clear oil; ^{1}H NMR (600 MHz, 300K, DMSO-d_6): δ = 12.54 (s, 1H), 8.09 (d, *J* = 1.0 Hz, 1H), 7.89 – 7.86 (m, 2H), 7.79 – 7.74 (m, 1H), 7.73 (d, *J* = 1.0 Hz, 1H), 7.68 – 7.64 (m, 2H), 7.21 (s, 1H), 5.61 (s, 2H), 4.58 (s, 2H) ppm; ^{13}C NMR (151 MHz, 300K, DMSO-d_6): δ = 160.0, 157.7, 145.3, 139.0, 134.2, 133.3, 129.3, 128.0, 125.0, 112.3, 60.7, 48.8 ppm; IR (film): $\tilde{\nu}$ = 2981, 1688, 1661, 1560, 1324, 1157, 1084 cm^{-1}; HRMS (ESI): *m/z* calcd for $C_{14}H_{14}O_3N_5S_2$ [*M*+H]$^+$: 364.0533, found: 364.0527.

(*Z*)-*N*-(4-((1*H*-1,2,3-Triazol-1-yl)methyl)-3-hydroxythiazol-2(3*H*)-ylidene)-2-(phenylsulfonyl)acetamide (12)

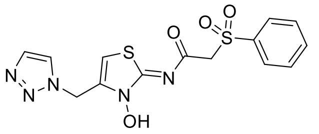

According to General Procedure B, *N*-hydroxythiazole **12** (13 mg, 0.03 mmol, 26%) was obtained from thiazole **12c** (48 mg, 0.13 mmol, 1.0 equiv.), following reverse-phase column chromatography (12 g Sfär C18 Duo; 12 mL/min; 100% water (+ 0.1% (*v/v*) formic acid) (4 CV), followed by a linear gradient (20 CV): 0%→100% acetonitrile (+ 0.1% (*v/v*) formic acid) in water (+ 0.1% (*v/v*) formic acid)).

White solid; m.p.: > 150 °C (decomposition); ^{1}H NMR (600 MHz, 300K, DMSO-*d*$_6$): δ = 8.22 (s, 1H), 7.90 (d, *J* = 8.0 Hz, 2H), 7.78 – 7.72 (m, 2H), 7.68 – 7.62 (m, 2H), 7.29 (s, 1H), 5.69 (s, 2H), 4.75 (s, 2H) ppm; ^{13}C NMR (151 MHz, 300K, DMSO-*d*$_6$): δ = 163.0 (br), 146.5 (br), 139.4, 136.1 (br), 134.1, 133.4, 129.3, 128.0, 125.6, 109.7, 61.3, 44.3 ppm; IR (film): $\tilde{\nu}$ = 3102, 1691, 1679, 1551, 1314, 1156, 1082 cm^{-1}; HRMS (ESI): *m/z* calcd for $C_{14}H_{14}O_4N_5S_2$ [*M*+H]$^+$: 380.0482, found: 380.0479.

Methyl 3-(2-aminothiazol-4-yl)propanoate (13b)

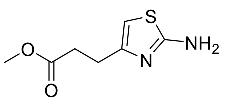

To a solution of 4-oxopentanoic acid **13a** (1.00 g, 8.6 mmol, 1.0 equiv.) in methanol (25 mL; HPLC grade) under an ambient atmosphere at room temperature was added bromine (1.38 g, 8.6 mmol, 1.0 equiv.). The reaction mixture was heated under reflux in the dark for 3 h. The solvent was removed under reduced pressure. The crude residue was redissolved in ethanol (25 mL; HPLC grade) and thiourea (761 mg, 10.0 mmol, 1.0 equiv.) was added. The reaction mixture was heated under reflux for 2 h. The solvent was removed under reduced pressure. The crude residue was dissolved in ethyl acetate and washed twice with saturated aqueous $NaHCO_3$ solution and brine, dried over anhydrous Na_2SO_4, filtered, and evaporated. The crude residue was purified using column chromatography (50 g Sfär Silica D; 120 mL/min; 100% cyclohexane (2 CV), followed by a linear gradient (12 CV): 0% → 50% ethyl acetate in cyclohexane) to afford 2-aminothiazole **13b** (483 mg, 2.59 mmol, 30%).

Yellow solid, m.p.: 70-73 °C; ^{1}H NMR (400 MHz, 300K, $CDCl_3$): δ = 6.10 (s, 1H), 5.22 (br s, 2H), 3.65 (s, 3H), 2.83 (t, *J* = 7.5 Hz, 2H), 2.63 (t, *J* = 7.5 Hz, 2H) ppm; ^{13}C NMR (101 MHz, 300K, $CDCl_3$): δ = 173.5, 168.0, 151.1, 102.7, 51.7, 33.3, 26.9 ppm; IR (film): $\tilde{\nu}$ = 3348, 2981, 1726, 1621, 1523, 1339, 1163 cm^{-1}; HRMS (ESI): *m/z* calcd for $C_7H_{10}N_2O_2SNa$ [*M*+Na]$^+$: 209.0355, found: 209.0363.

The analytical data is consistent with the literature.[62]

Methyl 3-(2-(2-(phenylsulfonyl)acetamido)thiazol-4-yl)propanoate (13c)

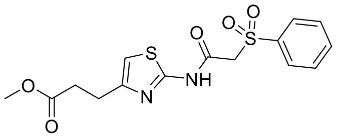

According to General Procedure A, amide **13c** (603 mg, 1.6 mmol, 82%) was obtained from 2-aminothiazole **13b** (375 mg, 2.0 mmol) and 2-(phenylsulfonyl)acetic acid (480 mg, 2.4 mmol), following column chromatography (25 g Sfär Silica D; 60 mL/min; 100% cyclohexane (2 CV), followed by a linear gradient (16 CV): 0%→40% acetone in cyclohexane).

Yellow oil; ^{1}H NMR (600 MHz, 300K, $CDCl_3$): δ = 7.93 (d, *J* = 8.0 Hz, 2H), 7.70 (t, *J* = 8.0 Hz, 1H), 7.60 – 7.55 (m, 2H), 6.64 (s, 1H), 4.28 (s, 2H), 3.69 (s, 3H), 3.00 (t, *J* = 7.5 Hz, 2H), 2.72 (t, *J* = 7.5 Hz, 2H) ppm; ^{13}C NMR (151 MHz, 300K, $CDCl_3$): δ = 173.3, 158.6, 156.7, 149.9, 138.0, 135.0, 129.8, 128.4, 109.2, 62.1, 51.9, 33.3, 26.5 ppm; IR (film): $\tilde{\nu}$ = 3271, 2981, 1733, 1689, 1558, 1325, 1156 cm^{-1}; HRMS (ESI): *m/z* calcd for $C_{15}H_{17}N_2O_5S_2$ [*M*+H]$^+$: 369.0573, found: 369.0580.

Methyl (*Z*)-3-(3-hydroxy-2-((2-(phenylsulfonyl)acetyl)imino)-2,3-dihydrothiazol-4-yl)propanoate (13d)

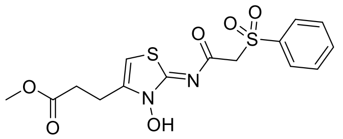

According to General Procedure B, *N*-hydroxythiazole **13d** (125 mg, 0.33 mmol, 40%) was obtained from thiazole **13c** (300 mg, 0.81 mmol), following column chromatography (10 g Sfär Silica D; 35 mL/min; 100% dichloromethane (2 CV), followed by a linear gradient (20 CV): 0%→5% methanol in dichloromethane).

White solid, m.p.: 78-81 °C; ^{1}H NMR (600 MHz, 300K, $CDCl_3$): δ = 7.87 (d, *J* = 8.0 Hz, 2H), 7.62 (t, *J* = 8.0 Hz, 1H), 7.52 – 7.47 (m, 2H), 6.74 (s, 1H), 4.63 (s, 2H), 3.70 (s, 3H), 3.17 (t, *J* = 7.0 Hz, 2H), 2.83 (t, *J* = 7.0 Hz, 2H) ppm; ^{13}C NMR (151 MHz, 300K, $CDCl_3$): δ = 172.6, 160.6, 144.5, 142.4, 138.4, 134.5, 129.3, 128.7, 105.8, 62.0, 52.0, 31.2, 22.4 ppm; IR (film): $\tilde{\nu}$ = 2981, 1734, 1685, 1552, 1326, 1156, 1084 cm^{-1}; HRMS (ESI): *m/z* calcd for $C_{15}H_{17}N_2O_6S_2$ [*M*+H]$^+$: 385.0523, found: 385.0534.

(*Z*)-3-(3-Hydroxy-2-((2-(phenylsulfonyl)acetyl)imino)-2,3-dihydrothiazol-4-yl)propanoic acid (13)

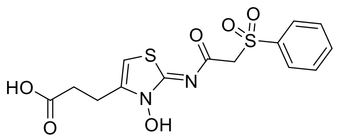

According to General Procedure C, carboxylic acid **13** (18 mg, 0.05 mmol, 49%) was obtained from methyl ester **13d** (40 mg, 0.10 mmol), following trituration with H_2O (3 × 5 mL; Milli-Q® Ultrapure grade) and MeOH (3 × 5 mL; HPLC grade).

White solid, m.p.: 173-176 °C; ^{1}H NMR (600 MHz, 300K, DMSO-*d*$_6$): δ = 7.93 – 7.89 (m, 2H), 7.77 – 7.71 (m, 1H), 7.67 – 7.63 (m, 2H), 6.92 (s, 1H), 4.69 (s, 2H), 2.83 (t, *J* = 7.5 Hz, 2H), 2.62 (t, *J* = 7.5 Hz, 2H) ppm; ^{13}C NMR (151 MHz, 300K, DMSO-*d*$_6$): δ = 173.2, 164.1 (br), 150.0 (br), 140.1 (br), 139.6, 134.0, 129.2, 128.0, 104.0, 62.1, 30.7, 21.8 ppm; IR (film): $\tilde{\nu}$ = 2981, 1698, 1551, 1327, 1151, 1084 cm^{-1}; HRMS (ESI): *m/z* calcd for $C_{14}H_{15}N_2O_6S_2$ [*M*+H]$^+$: 371.0366, found: 371.0370.

Ethyl 2-(2-acetamidothiazol-4-yl)acetate (14a)

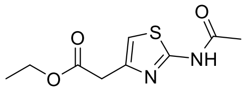

To a solution of ethyl 2-(2-aminothiazol-4-yl)acetate **1** (344 mg, 2.0 mmol, 1.0 equiv.) and 4-(dimethylamino)pyridine (257 mg, 2.1 mmol, 1.05 equiv.) in anhydrous tetrahydrofuran (8.0 mL) under an atmosphere of N_2 gas at ambient temperature was added acetic anhydride (0.20 mL, 214 mg, 2.1 mmol, 1.05 equiv.). The reaction mixture was heated under reflux for 1 h. The reaction mixture was allowed to cool to room temperature and the solvent evaporated under reduced pressure. The residue was redissolved in ethyl acetate and washed with 1N aqueous HCl solution, saturated aqueous $NaHCO_3$ solution and brine. The organic phase was dried over anhydrous Na_2SO_4, filtered and evaporated. The crude residue was purified using column chromatography (25 g Sfär Silica D; 60 mL/min; 100% dichloromethane (2 CV), followed by a linear gradient (20 CV): 0%→5% methanol in dichloromethane) to afford amide **14a** (263 mg, 1.15 mmol, 58%).

White solid, m.p.: 110-112 °C; ^{1}H NMR (400 MHz, 300K, DMSO-*d*$_6$): δ = 12.10 (s, 1H), 6.94 (s, 1H), 4.07 (q, *J* = 6.5 Hz, 2H), 3.66 (s, 2H), 2.11 (s, 3H), 1.18 (t, *J* = 6.5 Hz, 3H) ppm; ^{13}C NMR (101 MHz, 300K, DMSO-*d*$_6$): δ = 170.0, 168.3, 157.7, 143.5, 110.1, 60.3, 36.7, 22.4, 14.1 ppm; IR (film): $\tilde{\nu}$ = 2982, 1733, 1692, 1546, 1371, 1284, 1161, 1032 cm^{-1}; HRMS (ESI): *m/z* calcd for $C_9H_{13}O_3N_2S$ [*M*+H]$^+$: 229.0641, found: 229.0643.

The analytical data is consistent with the literature.[63]

Ethyl (*Z*)-2-(2-(acetylimino)-3-hydroxy-2,3-dihydrothiazol-4-yl)acetate (14b)

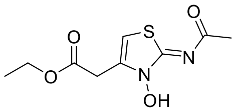

According to General Procedure B, *N*-hydroxythiazole **14b** (144 mg, 0.59 mmol, 67%) was obtained from thiazole **14a** (200 mg, 0.88 mmol), following column chromatography (10 g Sfär Silica D; 35 mL/min; 100% dichloromethane (3 CV), followed by a linear gradient (20 CV): 0%→5% methanol in dichloromethane).

White solid, m.p. = 171-173 °C; ^{1}H NMR (600 MHz, 300K, $CDCl_3$): δ = 6.93 (s, 1H), 4.19 (q, *J* = 7.0 Hz, 2H), 3.82 (s, 2H), 2.24 (s, 3H), 1.28 (d, *J* = 7.0 Hz, 3H) ppm; ^{13}C NMR (151 MHz, 300K, $CDCl_3$): δ = 169.2, 168.6, 142.9, 136.8, 107.3, 61.7, 32.2, 22.9, 14.3 ppm; IR (film): $\tilde{\nu}$ = 2981, 1721, 1681, 1542, 1371, 1213, 1016 cm^{-1}; HRMS (ESI): *m/z* calcd for $C_9H_{13}O_4N_2S$ [*M*+H]$^+$: 245.0591, found: 245.0591.

(*Z*)-2-(2-(Acetylimino)-3-hydroxy-2,3-dihydrothiazol-4-yl)acetic acid (14)

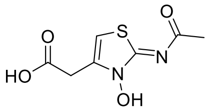

According to General Procedure C, carboxylic acid **14** (7 mg, 0.04 mmol, 10%) was obtained from ethyl ester **14b** (75 mg, 0.31 mmol), following reverse-phase column chromatography (12 g Sfär C18 Duo; 12 mL/min; 100% water (+ 0.1% (*v/v*) formic acid) (4 CV), followed by a linear gradient (20 CV): 0%→15% methanol (+ 0.1% (*v/v*) formic acid) in water (+ 0.1% (*v/v*) formic acid)).

White solid, m.p. = 190-193 °C; ^{1}H NMR (600 MHz, 300K, DMSO-*d*$_6$): δ = 7.25 (s, 1H), 3.73 (s, 2H), 2.24 (s, 3H) ppm; ^{13}C NMR (151 MHz, 300K, DMSO-*d*$_6$): δ = 169.6, 169.3, 142.0, 136.5, 107.8, 33.0, 22.5 ppm; IR (film): $\tilde{\nu}$ = 3084, 2980, 1705, 1551, 1337, 1230, 1179, 1118 cm^{-1}; HRMS (ESI): *m/z* calcd for $C_7H_9O_4N_2S$ [*M*+H]$^+$: 217.0278, found: 217.0278.

Ethyl 2-(2-(3-(phenylsulfonyl)propanamido)thiazol-4-yl)acetate (15a)

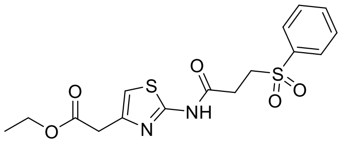

According to General Procedure A, amide **15a** (620 mg, 1.6 mmol, 54%) was obtained from ethyl 2-(2-aminothiazol-4-yl)acetate **1** (559 mg, 3.0 mmol) and 3-(phenylsulfonyl)propanoic acid (771 mg, 3.6 mmol), following column chromatography (25 g Sfär Silica D; 60 mL/min; 100% dichloromethane (2 CV), followed by a linear gradient (14 CV): 0%→30% ethyl acetate in dichloromethane).

Brown oil; ^{1}H NMR (600 MHz, 300K, $CDCl_3$): δ = 7.92 (d, *J* = 8.0 Hz, 2H), 7.64 (t, *J* = 8.0 Hz, 1H), 7.56 – 7.52 (m, 2H), 6.76 (s, 1H), 4.15 (q, *J* = 7.0 Hz, 2H), 3.69 (s, 2H), 3.56 (t, *J* = 7.5 Hz, 2H), 2.94 (t, *J* = 7.5 Hz, 2H), 1.24 (t, *J* = 7.0 Hz, 3H) ppm; ^{13}C NMR (151 MHz, 300K, $CDCl_3$): δ = 170.8, 167.3, 157.7, 143.3, 138.6, 134.2, 129.6, 128.2, 111.3, 61.4, 51.4, 37.0, 29.1, 14.2 ppm; IR (film): $\tilde{\nu}$ = 3271, 2984, 1732, 1691, 1550, 1289, 1151 cm^{-1}; HRMS (ESI): *m/z* calcd for $C_{16}H_{19}O_5N_2S_2$ [*M*+H]$^+$: 383.0730, found: 383.0732.

Ethyl (*Z*)-2-(3-hydroxy-2-((3-(phenylsulfonyl)propanoyl)imino)-2,3-dihydrothiazol-4-yl)acetate (15b)

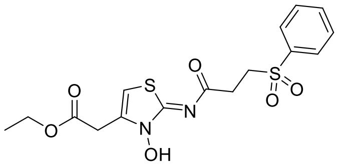

According to General Procedure B, *N*-hydroxythiazole **15b** (197 mg, 0.49 mmol, 63%) was obtained from thiazole **15a** (300 mg, 0.79 mmol), following column chromatography (10 g Sfär Silica D; 35 mL/min; 100% dichloromethane (3 CV), followed by a linear gradient (20 CV): 0%→3% methanol in dichloromethane).

Yellow solid, m.p.: 157-159 °C; ^{1}NMR (600 MHz, 300K, DMSO-*d*$_6$): δ = 7.90 (d, *J* = 8.0 Hz, 2H), 7.74 (t, *J* = 8.0 Hz, 1H), 7.67 – 7.63 (m, 2H), 7.20 (s, 1H), 4.09 (q, *J* = 7.0 Hz, 2H), 3.76 (s, 2H), 3.62 (t, *J* = 7.5 Hz,

2H), 2.92 (t, *J* = 7.5 Hz, 2H), 1.18 (t, *J* = 7.0 Hz, 3H) ppm; ^{13}C NMR (151 MHz, 300K, DMSO-d_6): δ = 169.3 (br), 168.5, 142.3 (br), 138.5, 136.3 (br), 133.9, 129.4, 127.9, 107.7, 60.6, 50.5, 31.8, 28.9, 14.0 ppm; IR (film): $\tilde{\nu}$ = 2984, 1735, 1684, 1549, 1307, 1147 cm^{-1}; HRMS (ESI): *m/z* calcd for $C_{16}H_{19}O_6N_2S_2$ [*M*+H]$^+$: 399.0679, found: 399.0679.

(*Z*)-2-(3-Hydroxy-2-((3-(phenylsulfonyl)propanoyl)imino)-2,3-dihydrothiazol-4-yl)acetic acid (15)

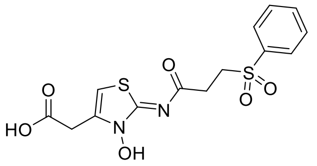

According to General Procedure C, carboxylic acid **15** (48 mg, 0.13 mmol, 59%) was obtained from ethyl ester **15b** (85 mg, 0.22 mmol), following trituration with H_2O (3 × 5 mL; Milli-Q® Ultrapure grade) and MeOH (3 × 5 mL; HPLC grade).

White solid, m.p.: 182-184 °C; ^1H NMR (600 MHz, 300K, DMSO-d_6): δ = 7.90 (d, *J* = 7.5 Hz, 2H), 7.74 (t, *J* = 7.5 Hz, 1H), 7.68 – 7.63 (m, 2H), 7.22 (s, 1H), 3.72 (s, 2H), 3.63 (t, *J* = 7.0 Hz, 2H), 2.94 (t, *J* = 7.0 Hz, 2H) ppm; ^{13}C NMR (151 MHz, 300K, DMSO-d_6): δ = 169.6, 169.3 (br), 143.2 (br), 138.5, 136.2 (br), 133.9, 129.4, 127.9, 107.7, 50.5, 32.7, 28.9 ppm; IR (film): $\tilde{\nu}$ = 3093, 2980, 1704, 1553, 1378, 1141, 1084 cm^{-1}; HRMS (ESI): *m/z* calcd for $C_{14}H_{15}O_6N_2S_2$ [*M*+H]$^+$: 371.0366, found: 371.0363.

Ethyl 2-(2-(2-((*tert*-butoxycarbonyl)amino)acetamido)thiazol-4-yl)acetate (16a)

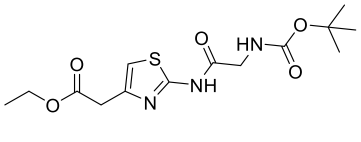

According to General Procedure A, amide **16a** (3.83 g, 11.2 mmol, 56%) was obtained from ethyl 2-(2-aminothiazol-4-yl)acetate (4.10 g, 20.0 mmol) **1** and *N*-(tert-butoxycarbonyl)glycine (4.20 g, 24.0 mmol), following column chromatography (100 g Sfär Silica D; 120 mL/min; 5% ethyl acetate in dichloromethane (2 CV), followed by a linear gradient (12 CV): 5%→35% ethyl acetate in dichloromethane).

White solid, m.p.: 155-156 °C; ^1H NMR (600 MHz, 300K, $CDCl_3$): δ = 6.73 (s, 1H), 5.28 (br s, 1H), 4.09 (q, *J* = 7.0 Hz, 2H), 4.01 (br s, 2H), 3.61 (s, 2H), 1.40 (s, 9H), 1.18 (t, *J* = 7.0 Hz, 3H) ppm; ^{13}C NMR (151 MHz, 300K, $CDCl_3$): δ = 170.5, 168.0, 157.6, 156.2, 143.4, 111.2, 81.0, 61.3, 44.4, 37.2, 28.4, 14.3 ppm; IR (film): $\tilde{\nu}$ = 2979, 1715, 1551, 1368, 1274, 1162, 1031 cm^{-1}; HRMS (ESI): *m/z* calcd for $C_{14}H_{22}O_5N_3S$ [*M*+H]$^+$: 344.1275, found: 344.1278.

The analytical data is consistent with the literature.[64]

Ethyl 2-(2-(2-aminoacetamido)thiazol-4-yl)acetate (16b)

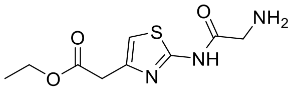

To a stirred solution of *N*-Boc protected amine **16a** (3.83 g, 11.2 mmol, 1.0 equiv.) in dichloromethane (20 mL; HPLC grade) at 0 °C under an ambient atmosphere was added trifluoroacetic acid (8.57 mL, 12.8 g, 112 mmol, 10.0 equiv.). The reaction mixture was allowed to warm to room temperature and stirred for 2 h before the solvent was removed under reduced pressure. The crude residue was redissolved with ethyl acetate and washed with saturated aqueous $NaHCO_3$ solution and brine, dried over anhydrous Na_2SO_4, filtered, and evaporated. The crude residue was purified using column chromatography (50 g Sfär Silica D; 100 mL/min; 100% dichloromethane (2 CV), followed by a linear gradient (12 CV): 0%→10% methanol (+1% (*v/v*) Et_3N) in dichloromethane) to afford amine **16b** (2.05 g, 8.4 mmol, 75%).

White solid, m.p.: 122-124 °C; ^1H NMR (600 MHz, CD_3OD): δ = 6.90 (s, 1H), 4.15 (q, *J* = 7.0 Hz, 2H), 3.69 (s, 2H), 3.51 (s, 2H), 1.25 (t, *J* = 7.0 Hz, 3H) ppm; ^{13}C NMR (151 MHz, CD_3OD): δ = 173.2, 172.3, 159.6,

145.0, 111.8, 62.1, 45.0, 37.6, 14.4 ppm; IR (film): $\tilde{\nu}$ = 3190, 1731, 1694, 1546, 1193 cm^{-1}; HRMS (ESI): *m/z* calcd for $C_9H_{14}O_3N_3S$ [*M*+H]$^+$: 244.0750, found: 244.0750.

Ethyl 2-(2-(2-benzamidoacetamido)thiazol-4-yl)acetate (16c)

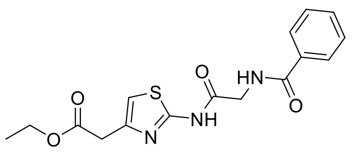

According to General Procedure A, amide **16c** (388 mg, 1.1 mmol, 54%) was obtained from amine **16b** (500 mg, 2.1 mmol) and benzoic acid (302 mg, 2.5 mmol), following column chromatography (25 g Sfär Silica D; 60 mL/min; 5% ethyl acetate in dichloromethane (2 CV), followed by a linear gradient (12 CV): 5%→50% ethyl acetate in dichloromethane).

White solid, m.p.: 185-186 °C; ^{1}H NMR (600 MHz, 300K, DMSO-*d*$_6$): δ = 12.31 (s, 1H), 8.90 (t, *J* = 6.0 Hz, 1H), 7.91 (d, *J* = 7.5 Hz, 2H), 7.57 (t, *J* = 7.5 Hz, 1H), 7.52 – 7.47 (m, 2H), 6.99 (s, 1H), 4.12 (d, *J* = 6.0 Hz, 2H), 4.09 (q, *J* = 7.0 Hz, 2H), 3.70 (s, 2H), 1.19 (t, *J* = 7.0 Hz, 3H) ppm; ^{13}C NMR (151 MHz, 300K, DMSO-*d*$_6$): δ = 170.0, 168.1, 166.6, 157.5, 143.6, 133.7, 131.4, 128.3, 127.3, 110.4, 60.3, 42.5, 36.6, 14.1 ppm; IR (film): $\tilde{\nu}$ = 3188, 1734, 1681, 1642, 1571, 1538, 1192, 1154 cm^{-1}; HRMS (ESI): *m/z* calcd for $C_{16}H_{18}O_4N_3S$ [*M*+H]$^+$: 348.1013, found: 348.1014.

Ethyl (*Z*)-2-(2-((benzoylglycyl)imino)-3-hydroxy-2,3-dihydrothiazol-4-yl)acetate (16d)

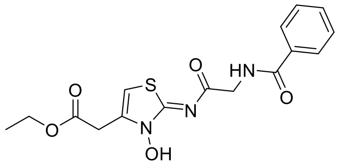

According to General Procedure B, *N*-hydroxythiazole **16d** (150 mg, 0.43 mmol, 57 %) was obtained from thiazole **16c** (250 mg, 0.75 mmol), following column chromatography (10 g Sfär Silica D; 35 mL/min; 100% dichloromethane (3 CV), followed by a linear gradient (20 CV): 0%→8% methanol in dichloromethane).

White solid, m.p.: 164-166 °C; ^{1}H NMR (600 MHz, 300K, DMSO-*d*$_6$): δ = 8.81 (br s, 1H), 7.90 (d, *J* = 7.5 Hz, 2H), 7.55 (t, *J* = 7.5 Hz, 1H), 7.50 – 7.46 (m, 2H), 7.08 (br s, 1H), 4.26 (s, 2H), 4.08 (q, *J* = 7.0 Hz, 2H), 3.76 (s, 2H), 1.17 (t, *J* = 7.0 Hz, 3H) ppm; ^{13}C NMR (151 MHz, 300K, DMSO-*d*$_6$): δ = 171.1 (br), 168.7, 166.5, 146.2 (br), 135.4 (br), 133.9, 131.4, 128.4, 127.3, 106.3 (br), 60.6, 43.8, 32.0, 14.1 ppm; IR (film): $\tilde{\nu}$ = 3013, 1738, 1542, 1372, 1217 cm^{-1}; HRMS (ESI): *m/z* calcd for $C_{16}H_{18}O_5N_3S$ [*M*+H]$^+$: 364.0962, found: 364.0962.

(*Z*)-2-(2-((Benzoylglycyl)imino)-3-hydroxy-2,3-dihydrothiazol-4-yl)acetic acid (16)

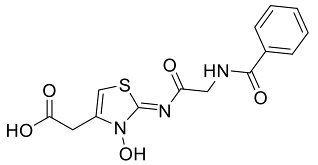

According to General Procedure C, carboxylic acid **16** (61 mg, 0.18 mmol, 65%) was obtained from ethyl ester **16d** (100 mg, 0.28 mmol), following trituration with H_2O (3 × 5 mL; Milli-Q® Ultrapure grade) and MeOH (3 × 5 mL; HPLC grade).

White solid, m.p.: 181-183 °C; ^{1}H NMR (600 MHz, 300K, DMSO-*d*$_6$): δ = 8.89 (t, *J* = 6.0 Hz, 1H), 7.89 (d, *J* = 7.5 Hz, 2H), 7.56 (t, *J* = 7.5 Hz, 1H), 7.51 – 7.47 (m, 2H), 7.22 (s, 1H), 4.28 (d, *J* = 6.0 Hz, 2H), 3.74 (s, 2H) ppm; ^{13}C NMR (151 MHz, 300K, DMSO-*d*$_6$): δ = 169.8 (br), 169.7, 166.7, 144.1 (br), 136.1, 133.7, 131.5, 128.4, 127.3, 107.5, 43.1, 32.7 ppm; IR (film): $\tilde{\nu}$ = 3092, 1711, 1655, 1566, 1512, 1381, 1184 cm^{-1}; HRMS (ESI): *m/z* calcd for $C_{14}H_{14}O_5N_3S$ [*M*+H]$^+$: 336.0649, found: 336.0652.

Ethyl 2-(2-(3-((*tert*-butoxycarbonyl)amino)propanamido)thiazol-4-yl)acetate (17a)

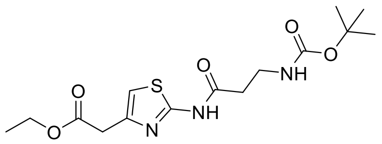

According to General Procedure A, amide **17a** (3.79 g, 10.6 mmol, 71%) was obtained from ethyl 2-(2-aminothiazol-4-yl)acetate **1** (2.79 g, 15.0 mmol) and 3-((*tert*-butoxycarbonyl)amino)propanoic acid (3.41 g, 18.0 mmol), following column chromatography (100 g Sfär Silica D; 120 mL/min; 5% ethyl acetate in dichloromethane (2 CV), followed by a linear gradient (12 CV): 5%→40% ethyl acetate in dichloromethane).

Yellow oil; ^{1}H NMR (600 MHz, 300K, DMSO-d_6): δ = 12.10 (br s, 1H), 6.95 (s, 1H), 6.85 (br s, 1H), 4.07 (q, *J* = 7.0 Hz, 2H), 3.66 (s, 2H), 3.22 (q, *J* = 6.5 Hz, 2H), 2.54 (t, *J* = 7.0 Hz, 2H), 1.36 (s, 9H), 1.18 (t, *J* = 7.0 Hz, 3H) ppm; ^{13}C NMR (151 MHz, 300K, DMSO-d_6): δ = 170.0, 169.5, 157.5, 155.5, 143.5, 110.2, 77.6, 60.3, 36.7, 36.1, 35.4, 28.2, 14.1 ppm; IR (film): $\tilde{\nu}$ = 2979, 1715, 1551, 1456, 1368, 1162, 1031 cm^{-1}; HRMS (ESI): *m/z* calcd for $C_{15}H_{24}O_5N_3S$ [M+H]$^+$: 358.1437, found: 358.1438.

Ethyl 2-(2-(3-aminopropanamido)thiazol-4-yl)acetate hydrochloride (17b)

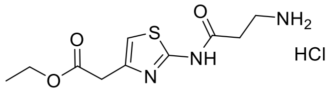

According to General Procedure D, amine HCl salt **17b** (810 mg, 2.8 mmol, 99%) was obtained from *N*-Boc protected amine **17a** (1.00 g, 2.8 mmol). **17b** was used in the subsequent reaction without further purification.

Yellow solid, m.p.: > 220 °C (decomposition); ^{1}H NMR (600 MHz, D_2O): δ = 7.10 (s, 1H), 4.23 (q, *J* = 7.0 Hz, 2H), 3.85 (s, 2H), 3.42 (t, *J* = 6.5 Hz, 2H), 3.04 (t, *J* = 6.5 Hz, 2H), 1.28 (t, *J* = 7.0 Hz, 3H) ppm; ^{13}C NMR (151 MHz, D_2O): δ = 172.9, 170.2, 159.0, 141.2, 112.6, 62.5, 35.6, 34.9, 32.1, 13.2 ppm; IR (film): $\tilde{\nu}$ = 3214, 1716, 1607, 1566, 1196, 1126, 1093 cm^{-1}; HRMS (ESI): *m/z* calcd for $C_{10}H_{16}O_3N_3S$ [M-Cl]$^+$: 258.0907, found: 258.0905.

Ethyl 2-(2-(3-benzamidopropanamido)thiazol-4-yl)acetate (17c)

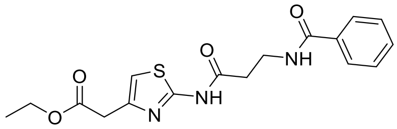

According to General Procedure A*, amide **17c** (261 mg, 4.9 mmol, 88%) was obtained from amine HCl salt **17b** (212 mg, 0.82 mmol) and phenylacetic acid (121 mg, 0.98 mmol), following column chromatography (10 g Sfär Silica D; 35 mL/min; 100% dichloromethane (2 CV), followed by a linear gradient (12 CV): 0%→10% methanol in dichloromethane).

*Note: 4.0 Equivalents (relative to amine HCl salt **17b**) of *N*,*N*-diisopropylethylamine (0.57 mL, 3.3 mmol) was used.

White solid, m.p.: 172-174 °C; ^{1}H NMR (600 MHz, 300K, $CDCl_3$): δ = 7.76 (d, *J* = 7.0 Hz, 2H), 7.47 (t, *J* = 7.0 Hz, 1H), 7.42 – 7.37 (m, 2H), 7.09 (t, *J* = 6.0 Hz, 1H), 6.80 (s, 1H), 4.16 (q, *J* = 7.0 Hz, 2H), 3.84 (q, *J* = 6.0 Hz, 2H), 3.68 (s, 2H), 2.89 (t, *J* = 6.0 Hz, 2H), 1.24 (t, *J* = 7.0 Hz, 3H) ppm; ^{13}C NMR (151 MHz, 300K, $CDCl_3$): δ = 170.4, 170.1, 168.0, 157.8, 143.0, 134.2, 131.8, 128.7, 127.2, 111.0, 61.4, 37.0, 35.8, 35.6, 14.3 ppm; IR (film): $\tilde{\nu}$ = 3058, 1728, 1553, 1328, 1160 cm^{-1}; HRMS (ESI): *m/z* calcd for $C_{17}H_{20}O_4N_3S$ [M+H]$^+$: 362.1169, found: 362.1158.

Ethyl (*Z*)-2-(2-((3-benzamidopropanoyl)imino)-3-hydroxy-2,3-dihydrothiazol-4-yl)acetate (17d)

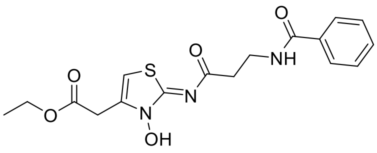

According to General Procedure B, *N*-hydroxythiazole **17d** (125 mg, 0.33 mmol, 73%) was obtained from thiazole **17c** (164 mg, 0.45 mmol), following column chromatography (10 g Sfär Silica D; 35 mL/min; 100% dichloromethane (3 CV), followed by a linear gradient (20 CV): 0%→10% methanol in dichloromethane).

Yellow solid, m.p.: 151-153 °C; ^{1}H NMR (600 MHz, 300K, DMSO-d_6): δ = 8.55 (t, *J* = 5.5 Hz, 1H), 7.82 (d, *J* = 7.5 Hz, 2H), 7.51 (t, *J* = 7.5 Hz, 1H), 7.46 – 7.41 (m, 2H), 7.23 (s, 1H), 4.09 (q, *J* = 7.0 Hz, 2H), 3.76 (s, 2H), 3.56 (q, *J* = 7.0 Hz, 2H), 2.85 (t, *J* = 7.0 Hz, 2H), 1.18 (t, *J* = 7.0 Hz, 3H) ppm; ^{13}C NMR (151 MHz, 300K, DMSO-d_6): δ = 170.6 (br), 168.6, 166.2, 140.9 (br), 136.7 (br), 134.4, 131.1, 128.2, 127.2, 107.8, 60.5, 35.6, 35.1, 31.8, 14.0 ppm; IR (film): $\tilde{\nu}$ = 3109, 1722, 1681, 1637, 1579, 1359, 1201 cm^{-1}; HRMS (ESI): *m/z* calcd for $C_{17}H_{20}O_5N_3S$ [*M*+H]$^+$: 378.1118, found: 378.1121.

(*Z*)-2-(2-((3-Benzamidopropanoyl)imino)-3-hydroxy-2,3-dihydrothiazol-4-yl)acetic acid (17)

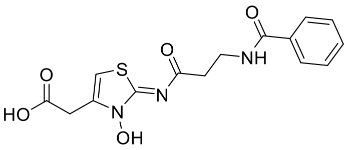

According to General Procedure C, carboxylic acid **17** (16 mg, 0.05 mmol, 42%) was obtained from ethyl ester **17d** (42 mg, 0.11 mmol), following trituration with H_2O (3 × 5 mL; Milli-Q® Ultrapure grade) and MeOH (3 × 5 mL; HPLC grade).

White solid, m.p.: 180-183 °C; ^{1}H NMR (600 MHz, 300K, DMSO-d_6): δ = 8.56 (t, *J* = 6.5 Hz, 1H), 7.82 (d, *J* = 7.5 Hz, 2H), 7.51 (t, *J* = 7.5 Hz, 1H), 7.47 – 7.43 (m, 2H), 7.26 (s, 1H), 3.74 (s, 2H), 3.56 (q, *J* = 6.5 Hz, 2H), 2.87 (t, *J* = 6.5 Hz, 2H) ppm; ^{13}C NMR (151 MHz, 300K, DMSO-d_6): δ = 170.6 (br), 169.6, 166.3, 142.2 (br), 136.4, 134.4, 131.1, 128.3, 127.2, 107.9, 35.6, 35.1, 32.9 ppm; IR (film): $\tilde{\nu}$ = 3100, 1710, 1643, 1526, 1401, 1378, 1163 cm^{-1}; HRMS (ESI): *m/z* calcd for $C_{15}H_{16}O_5N_3S$ [*M*+H]$^+$: 350.0805, found: 350.0804.

4-((4-(2-Ethoxy-2-oxoethyl)thiazol-2-yl)amino)-4-oxobutanoic acid (18a)

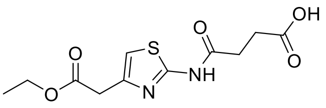

A solution of ethyl 2-(2-aminothiazol-4-yl)acetate **1** (1.86 g, 10.0 mmol, 1.0 equiv.) and succinic anhydride (1.00 g, 10.0 mmol, 1.0 equiv.) in anhydrous tetrahydrofuran (20 mL) was heated under reflux under a N_2 atmosphere for 14 h. The reaction mixture was cooled to room temperature and the solvent was removed under reduced pressure. The resultant white solid was triturated with H_2O (2 x 10 mL; Milli-Q® Ultrapure grade) and diethyl ether (2 x 10 mL; HPLC grade) and dried under high vacuum to yield carboxylic acid **18a** (2.21 g, 7.7 mmol, 77%).

White solid, m.p.: 172-174 °C; ^{1}H NMR (600 MHz, 300K, DMSO-d_6): δ = 12.15 (br s, 2H), 6.94 (s, 1H), 4.07 (q, *J* = 7.0 Hz, 2H), 3.67 (s, 2H), 2.63 (t, *J* = 6.5, 2H), 2.55 (t, *J* = 6.5 Hz, 2H), 1.18 (t, *J* = 7.0 Hz, 3H) ppm; ^{13}C NMR (151 MHz, 300K, DMSO-d_6): δ = 173.6, 170.3, 170.1, 157.6, 143.5, 110.2, 60.3, 36.7, 29.9, 28.4, 14.1 ppm; IR (film): $\tilde{\nu}$ = 3055, 1726, 1683, 1585, 1430, 1371, 1189, 1161 cm^{-1}; HRMS (ESI): *m/z* calcd for $C_{11}H_{15}O_5N_2S$ [*M*+H]$^+$: 287.0696, found: 287.0696.

Ethyl 2-(2-(2-((*tert*-butoxycarbonyl)amino)acetamido)thiazol-4-yl)acetate (18b)

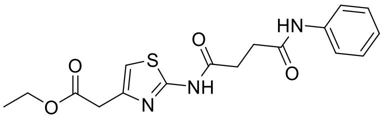

According to General Procedure A, amide **18b** (292 mg, 0.81 mmol, 81%) was obtained from aniline (91 µL, 93 mg, 1.0 mmol) and carboxylic acid **18a** (343 mg, 1.20 mmol), following column

chromatography (10 g Sfär Silica D; 35 mL/min; 10% ethyl acetate in dichloromethane (2 CV), followed by a linear gradient (12 CV): 10%→60% ethyl acetate in dichloromethane).

White solid, m.p. 150-152 °C; ^{1}H NMR (600 MHz, 300K, $CDCl_3$): δ = 10.77 (br s, 1H), 8.36 (s, 1H), 7.45 (d, *J* = 8.0 Hz, 2H), 7.24 – 7.20 (m, 2H), 7.03 (t, *J* = 8.0 Hz, 1H), 6.72 (s, 1H), 4.13 (q, *J* = 7.0 Hz, 2H), 3.68 (s, 2H), 2.85 (t, *J* = 7.0 Hz, 2H), 2.75 (t, *J* = 7.0 Hz, 2H), 1.22 (t, *J* = 7.0 Hz, 3H) ppm; ^{13}C NMR (101 MHz, 300K, $CDCl_3$): δ = 170.9(4), 170.9(2), 170.6, 158.0, 143.4, 138.0, 129.0, 124.4, 120.1, 110.9, 61.3, 37.1, 31.9, 31.3, 14.2 ppm; IR (film): $\tilde{\nu}$ = 3201, 2921, 1731, 1666, 1542, 1273, 1159 cm^{-1}; HRMS (ESI): *m/z* calcd for $C_{17}H_{20}O_4N_3S$ [*M*+H]$^+$: 362.1169, found: 362.1170.

Ethyl (*Z*)-2-(3-hydroxy-2-((4-oxo-4-(phenylamino)butanoyl)imino)-2,3-dihydrothiazol-4-yl)acetate (18c)

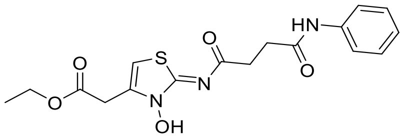

According to General Procedure B, *N*-hydroxythiazole **18c** (70 mg, 0.19 mmol, 42%) was obtained from thiazole **18b** (160 mg, 0.44 mmol), following column chromatography (10 g Sfär Silica D; 35 mL/min; 100% dichloromethane (3 CV), followed by a linear gradient (20 CV): 0%→5% methanol in dichloromethane).

White solid, m.p. 179-182 °C; ^{1}H NMR (400 MHz, 300K, DMSO-*d*$_6$): δ = 9.98 (s, 1H), 7.57 (d, *J* = 8.0 Hz, 2H), 7.30 – 7.24 (m, 3H), 7.01 (t, *J* = 7.5 Hz, 1H), 4.09 (q, *J* = 7.0 Hz, 2H), 2.88 (t, *J* = 6.5 Hz, 2H), 2.67 (t, *J* = 6.5 Hz, 2H), 1.19 (t, *J* = 7.0 Hz, 3H) ppm; ^{13}C NMR (101 MHz, 300K, DMSO-*d*$_6$): δ = 171.4, 170.0, 168.5, 140.6 (br), 139.2, 136.8, 128.6, 122.9, 118.9, 107.7, 60.5, 31.8, 30.8, 30.0, 14.0 ppm; IR (film): $\tilde{\nu}$ = 2921, 1700, 1662, 1540, 1362, 1295, 1171 cm^{-1}; HRMS (ESI): *m/z* calcd for $C_{17}H_{20}O_5N_3S$ [*M*+H]$^+$: 378.1118, found: 378.1120.

(*Z*)-2-(3-Hydroxy-2-((4-oxo-4-(phenylamino)butanoyl)imino)-2,3-dihydrothiazol-4-yl)acetic acid (18)

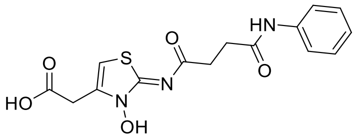

According to General Procedure C, carboxylic acid **18** (16 mg, 0.05 mmol, 37%) was obtained from ethyl ester **18c** (50 mg, 0.13 mmol), following reverse-phase column chromatography (12 g Sfär C18 Duo; 12 mL/min; 100% water (+ 0.1% (*v/v*) formic acid) (4 CV), followed by a linear gradient (20 CV): 0%→50% methanol (+ 0.1% (*v/v*) formic acid) in water (+ 0.1% (*v/v*) formic acid)).

White solid, m.p. 168-170 °C; ^{1}H NMR (400 MHz, 300K, DMSO-*d*$_6$): δ = 10.00 (s, 1H), 7.57 (d, *J* = 7.5 Hz, 2H), 7.30 – 7.25 (m, 2H), 7.21 (s, 1H), 7.01 (t, *J* = 7.5 Hz, 1H), 3.73 (s, 2H), 2.88 (t, *J* = 7.0 Hz, 2H), 2.67 (t, *J* = 7.0 Hz, 2H) ppm; ^{13}C NMR (151 MHz, 300K, DMSO-*d*$_6$): δ = 172.3 (br), 170.1, 169.8, 143.7 (br), 139.3, 136.2, 128.7, 122.9, 118.9, 107.2 (br), 33.4, 30.9, 30.5 ppm; IR (film): $\tilde{\nu}$ = 3097, 2980, 1732, 1691, 1546, 1370, 1212, 1161 cm^{-1}; HRMS (ESI): *m/z* calcd for $C_{15}H_{16}O_5N_3S$ [*M*+H]$^+$: 350.0805, found: 350.0805.

Ethyl 2-(2-(2-(phenylsulfonamido)acetamido)thiazol-4-yl)acetate (19a)

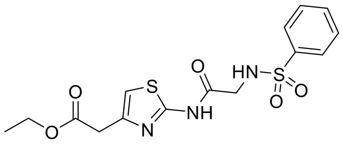

According to General Procedure E*, sulfonamide **19a** (528 mg, 1.4 mmol, 69%) was obtained from amine **16b** (487 mg, 2.0 mmol) and benzenesulfonyl chloride (0.31 mL, 2.4 mmol), following column chromatography (10 g Sfär Silica D; 35 mL/min; 100% dichloromethane (2 CV), followed by a linear gradient (12 CV): 0%→40% ethyl acetate in dichloromethane).

*Note: 1.2 Equivalents (relative to amine **16b**) of triethylamine (0.34 mL, 2.4 mmol) was used.

White solid, m.p.: 145-146 °C; ^{1}H NMR (600 MHz, 300K, $CDCl_3$): δ = 7.85 (d, *J* = 8.0 Hz, 2H), 7.55 (t, *J* = 8.0 Hz, 1H), 7.50 – 7.45 (m, 2H), 6.79 (s, 1H), 6.67 (t, *J* = 5.5 Hz, 1H), 4.12 (q, *J* = 7.0 Hz, 2H), 3.93 (d, *J* = 5.5 Hz, 2H), 3.66 (s, 2H), 1.23 (t, *J* = 7.0 Hz, 3H) ppm; ^{13}C NMR (151 MHz, 300K, $CDCl_3$): δ = 170.6, 166.9, 157.5, 143.3, 139.1, 133.3, 129.4, 127.3, 111.6, 61.4, 45.9, 36.9, 14.3 ppm; IR (film): $\tilde{\nu}$ = 3068, 1734, 1676, 1572, 1304, 1162 cm^{-1}; HRMS (ESI): *m/z* calcd for $C_{15}H_{18}O_5N_3S_2$ [*M*+H]$^+$: 384.0682, found: 384.0681.

Ethyl (*Z*)-2-(3-hydroxy-2-(((phenylsulfonyl)glycyl)imino)-2,3-dihydrothiazol-4-yl)acetate (19b)

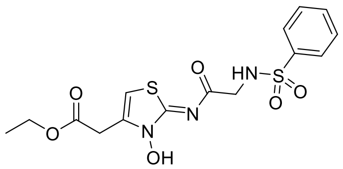

According to General Procedure B, *N*-hydroxythiazole **19b** (164 mg, 0.41 mmol, 82%) was obtained from thiazole **19a** (192 mg, 0.50 mmol), following column chromatography (10 g Sfär Silica D; 35 mL/min; 100% dichloromethane (2 CV), followed by a linear gradient (12 CV): 0%→5% methanol in dichloromethane).

Yellow solid, m.p.: 144-145 °C; ^{1}H NMR (600 MHz, 300K, DMSO-*d*$_6$): δ = 8.07 (s, 1H), 7.79 (d, *J* = 8.0 Hz, 2H), 7.63 – 7.52 (m, 3H), 7.10 (s, 1H), 4.09 (q, *J* = 7.0 Hz, 2H), 3.86 (s, 2H), 3.74 (s, 2H), 1.18 (t, *J* = 7.0 Hz, 3H) ppm; ^{13}C NMR (151 MHz, 300K, DMSO-*d*$_6$): δ = 169.2 (br), 168.4, 145.3 (br), 140.4, 135.5 (br), 132.4, 129.1, 126.5, 107.4, 60.6, 45.6, 31.8, 14.0 ppm; IR (film): $\tilde{\nu}$ = 3107, 1731, 1552, 1359, 1329, 1160, 1094 cm^{-1}; HRMS (ESI): *m/z* calcd for $C_{15}H_{18}O_6N_3S_2$ [*M*+H]$^+$: 400.0632, found: 400.0632.

(*Z*)-2-(3-Hydroxy-2-(((phenylsulfonyl)glycyl)imino)-2,3-dihydrothiazol-4-yl)acetic acid (19)

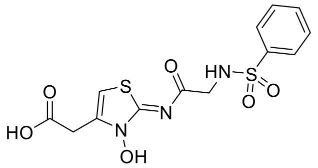

According to General Procedure C, carboxylic acid **19** (44 mg, 0.12 mmol, 66%) was obtained from ethyl ester **19b** (70 mg, 0.18 mmol), following trituration with H_2O (3 × 5 mL; Milli-Q® Ultrapure grade) and MeOH (3 × 5 mL; HPLC grade).

White solid, m.p.: 178-180 °C; ^{1}H NMR (600 MHz, 300K, DMSO-*d*$_6$): δ = 8.11 (t, *J* = 6.0 Hz, 1H), 7.81 (d, *J* = 7.5 Hz, 2H), 7.61 (t, *J* = 7.5 Hz, 1H), 7.58 – 7.54 (m, 2H), 7.17 (s, 1H), 3.91 (d, *J* = 6.0 Hz, 2H), 3.71 (s, 2H) ppm; ^{13}C NMR (151 MHz, 300K, DMSO-*d*$_6$): δ = ^{13}C NMR (151 MHz, DMSO) δ = 169.6, 169.1 (br), 145.4 (br), 140.4, 135.7 (br), 132.4, 129.1, 126.5, 107.2, 45.6, 32.5 ppm; IR (film): $\tilde{\nu}$ = 3269, 3106, 1730, 1703, 1552, 1330, 1158 cm^{-1}; HRMS (ESI): *m/z* calcd for $C_{13}H_{12}O_6N_3S_2$ [*M*-H]$^-$: 370.0173, found: 370.0168.

Ethyl 2-(2-(3-(phenylsulfonamido)propanamido)thiazol-4-yl)acetate (20a)

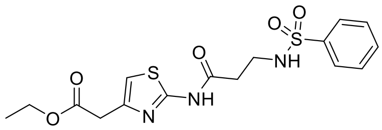

According to General Procedure E, sulfonamide **20a** (402 mg, 1.0 mmol, 79%) was obtained from amine HCl salt **17b** (329 mg, 1.3 mmol) and benzenesulfonyl chloride (0.20 mL, 1.5 mmol), following column chromatography (10 g Sfär Silica D; 35 mL/min; 100% dichloromethane (2 CV), followed by a linear gradient (12 CV): 0%→40% ethyl acetate in dichloromethane).

Yellow oil; ^{1}H NMR (600 MHz, 300K, $CDCl_3$): δ = 7.89 (d, *J* = 8.0 Hz, 2H), 7.58 – 7.52 (m, 1H), 7.51 – 7.46 (m, 2H), 7.21 (t, *J* = 6.5 Hz, 1H), 6.72 (s, 1H), 4.08 (q, *J* = 7.0 Hz, 2H), 3.64 (s, 2H), 3.41 (q, *J* = 6.5 Hz, 2H), 2.72 (t, *J* = 6.5 Hz, 2H), 1.19 (t, *J* = 7.0 Hz, 3H) ppm; ^{13}C NMR (151 MHz, 300K, $CDCl_3$): δ = 170.3, 169.6, 158.1, 142.8, 140.6, 132.7, 129.3, 127.0, 111.1, 61.2, 39.5, 37.7, 36.9, 14.2 ppm; IR (film): $\tilde{\nu}$ = 3271,

1732, 1687, 1548, 1325, 1158, 1093 cm^{-1}; HRMS (ESI): *m/z* calcd for $C_{16}H_{20}O_5N_3S_2$ [*M*+H]$^+$: 398.0839, found: 398.0832.

Ethyl (*Z*)-2-(2-((*N*-(tert-butoxycarbonyl)-*N*-phenylglycyl)imino)-3-hydroxy-2,3-dihydrothiazol-4-yl)acetate (20b)

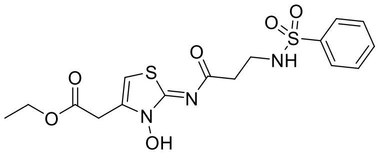

According to General Procedure B, *N*-hydroxythiazole **20b** (72 mg, 0.17 mmol, 59 %) was obtained from thiazole **20a** (114 mg, 0.29 mmol), following column chromatography (10 g Sfär Silica D; 35 mL/min; 100% dichloromethane (3 CV), followed by a linear gradient (20 CV): 0%→10% methanol in dichloromethane).

Yellow oil; ^{1}H NMR (600 MHz, 300K, DMSO-d_6): δ = 7.82 – 7.77 (m, 2H), 7.73 (s, 1H), 7.66 – 7.61 (m, 1H), 7.60 – 7.56 (m, 2H), 7.22 (s, 1H), 4.09 (q, *J* = 7.0 Hz, 2H), 3.81 – 3.72 (m, 2H), 3.09 – 2.99 (m, 2H), 2.72 (t, *J* = 7.0 Hz, 2H), 1.18 (t, *J* = 7.0 Hz, 3H) ppm; ^{13}C NMR (151 MHz, 300K, DMSO-d_6): δ = 170.0 (br), 168.5, 141.3 (br), 140.2, 136.6 (br), 132.4, 129.2, 126.5, 107.7, 60.5, 38.6, 35.4, 31.8, 14.0 ppm; IR (film): $\tilde{\nu}$ = 3111, 1734, 1687, 1546, 1326, 1157 cm^{-1}; HRMS (ESI): *m/z* calcd for $C_{16}H_{20}O_6N_3S_2$ [*M*+H]$^+$: 414.0788, found 414.0788.

(*Z*)-2-(3-Hydroxy-2-((3-(phenylsulfonamido)propanoyl)imino)-2,3-dihydrothiazol-4-yl)acetic acid (20)

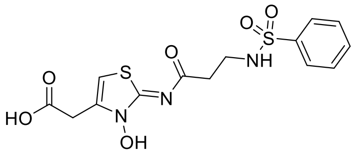

According to General Procedure C, carboxylic acid **20** (29 mg, 0.08, 68%) was obtained from ethyl ester **20b** (45 mg, 0.11 mmol), following trituration with H_2O (3 × 5 mL; Milli-Q® Ultrapure grade) and MeOH (3 × 5 mL; HPLC grade).

White solid, m.p.: 178-180 °C; ^{1}H NMR (600 MHz, 300K, DMSO-d_6): δ = 7.79 (d, *J* = 7.5 Hz, 2H), 7.74 (t, *J* = 6.5 Hz, 1H), 7.63 (t, *J* = 7.5 Hz, 1H), 7.61 – 7.56 (m, 2H), 7.25 (s, 1H), 3.73 (s, 2H), 3.05 (q, *J* = 6.5 Hz, 2H), 2.74 (t, *J* = 6.5 Hz, 2H) ppm; ^{13}C NMR (151 MHz, 300K, DMSO-d_6): δ = 170.0 (br), 169.6, 142.4 (br), 140.2, 136.3, 132.4, 129.2, 126.5, 107.9, 38.5, 35.4, 32.8 ppm; IR (film): $\tilde{\nu}$ = 3097, 1694, 1550, 1371, 1327, 1047, 1020 cm^{-1}; HRMS (ESI): *m/z* calcd for $C_{14}H_{16}O_6N_3S_2$ [*M*+H]$^+$: 386.0475, found: 386.0477.

Ethyl 2-(2-(2-(*N*-phenylsulfamoyl)acetamido)thiazol-4-yl)acetate (21a)

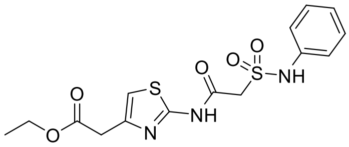

According to General Procedure A, amide **21a** (426 mg, 1.1 mmol, 56%) was obtained from ethyl 2-(2-aminothiazol-4-yl)acetate **1** (372 mg, 2.0 mmol) and 2-(*N*-phenylsulfamoyl)acetic acid[58] (516 mg, 2.4 mmol), following column chromatography (25 g Sfär Silica D; 35 mL/min; 10% ethyl acetate in cyclohexane (2 CV), followed by a linear gradient (12 CV): 10%→50% ethyl acetate in cyclohexane).

White solid, m.p.: 159-161 °C; ^{1}H NMR (600 MHz, 300K, DMSO-d_6): δ = 12.54 (br s, 1H), 10.16 (br s, 1H), 7.36 – 7.31 (m, 2H), 7.26 (d, *J* = 7.5 Hz, 2H), 7.12 (t, *J* = 7.5 Hz, 1H), 7.05 (s, 1H), 4.27 (s, 2H), 4.08 (q, *J* = 7.0 Hz, 2H), 3.69 (s, 2H), 1.18 (t, *J* = 7.0 Hz, 3H) ppm; ^{13}C NMR (151 MHz, 300K, DMSO-d_6): δ = 169.9, 160.2, 156.9, 143.9, 137.5, 129.2, 124.2, 120.4, 111.1, 60.3, 56.0, 36.6, 14.1 ppm; IR (film): $\tilde{\nu}$ = 2984, 1727, 1561, 1348, 1162, 1030 cm^{-1}; HRMS (ESI): *m/z* calcd for $C_{15}H_{18}O_5N_3S_2$[*M*+H]$^+$: 384.0682, found: 384.0680.

Ethyl (*Z*)-2-(3-hydroxy-2-((2-(*N*-phenylsulfamoyl)acetyl)imino)-2,3-dihydrothiazol-4-yl)acetate (21b)

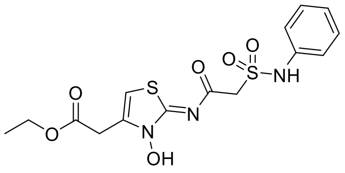

According to General Procedure B, *N*-hydroxythiazole **21b** (211 mg, 0.53 mmol, 59%) was obtained from thiazole **21a** (340 mg, 0.89 mmol), following column chromatography (10 g Sfär Silica D; 35 mL/min; 100% dichloromethane (2 CV), followed by a linear gradient (20 CV): 0%→3% methanol in dichloromethane).

Yellow solid, m.p.: 127-129 °C; ^{1}H NMR (600 MHz, 300K, DMSO-d_6): δ = 9.83 (s, 1H), 7.36 – 7.23 (m, 4H), 7.10 (t, *J* = 6.0 Hz, 1H), 7.01 (s, 1H), 4.31 (s, 2H), 4.10 (q, *J* = 7.0 Hz, 2H), 3.74 (s, 2H), 1.18 (t, *J* = 7.0 Hz, 3H) ppm; ^{13}C NMR (151 MHz, 300K, DMSO-d_6): δ = 168.5, 163.8 (br), 149.9 (br), 137.7, 134.9 (br), 129.1, 124.3, 120.7, 107.3, 60.7, 56.9, 31.9, 14.0 ppm; IR (film): $\tilde{\nu}$ = 2980, 1734, 1689, 1382, 1252, 1153, 1073 cm^{-1}; HRMS (ESI): *m/z* calcd for $C_{15}H_{18}O_6N_3S_2$ [*M*+H]$^+$: 400.0632, found: 400.0627.

(*Z*)-2-(3-Hydroxy-2-((*N*-methyl-*N*-(methylsulfonyl)glycyl)imino)-2,3-dihydrothiazol-4-yl)acetic acid (21)

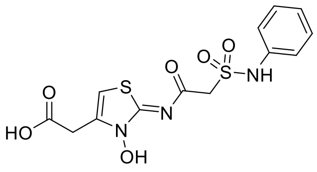

According to General Procedure C, carboxylic acid **21** (26 mg, 0.07 mmol, 28%) was obtained from ethyl ester **21b** (100 mg, 0.25 mmol), following reverse-phase column chromatography (12 g Sfär C18 Duo; 12 mL/min; 100% water (+ 0.1% (*v/v*) formic acid) (4 CV), followed by a linear gradient (20 CV): 0%→15% methanol (+ 0.1% (*v/v*) formic acid) in water (+ 0.1% (*v/v*) formic acid)).

White solid, m.p.: 181-183 °C; ^{1}H NMR (600 MHz, 300K, DMSO-d_6): δ = 9.93 (s, 1H), 7.36 – 7.32 (m, 2H), 7.31 – 7.28 (m, 2H), 7.17 (s, 1H), 7.13 (t, *J* = 7.0 Hz, 1H), 4.35 (s, 2H), 3.74 (s, 2H) ppm; ^{13}C NMR (151 MHz, 300K, DMSO-d_6): δ = 169.8, 164.3 (br), 148.9 (br), 137.7, 135.1 (br), 129.2, 124.3, 120.8, 107.1, 56.9, 32.3 ppm; IR (film): $\tilde{\nu}$ = 3103, 1702, 1558, 1354, 1158 cm^{-1}; HRMS (ESI): *m/z* calcd for $C_{13}H_{12}O_6N_3S_2$ [*M*-H]$^-$: 370.0173, found: 370.0169.

Ethyl 2-(2-(2-phenoxyacetamido)thiazol-4-yl)acetate (22a)

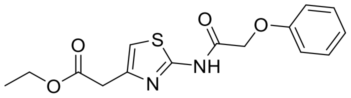

According to General Procedure A, amide **22a** (541 mg, 1.7 mmol, 85%) was obtained from ethyl 2-(2-aminothiazol-4-yl)acetate **1** (372 mg, 2.0 mmol) and phenoxyacetic acid (365 mg, 2.4 mmol), following column chromatography (25 g Sfär Silica D; 60 mL/min; 5% ethyl acetate in cyclohexane (2 CV), followed by a linear gradient (12 CV): 5%→40% ethyl acetate in cyclohexane).

White solid, m.p.: 129-130 °C; ^{1}H NMR (600 MHz, 300K, $CDCl_3$): δ = 9.86 (br s, 1H), 7.36 – 7.31 (m, 2H), 7.05 (t, *J* = 7.5 Hz, 1H), 6.94 (d, *J* = 7.5 Hz, 2H), 6.85 (s, 1H), 4.70 (s, 2H), 4.18 (q, *J* = 7.0 Hz, 2H), 3.71 (s, 2H), 1.26 (t, *J* = 7.0 Hz, 3H) ppm; ^{13}C NMR (151 MHz, 300K, $CDCl_3$): δ = 170.3, 166.3, 156.8, 156.7, 143.8, 130.0, 122.7, 114.8, 111.5, 66.8, 61.3, 37.2, 14.3 ppm; IR (film): $\tilde{\nu}$ = 2935, 1732, 1691, 1540, 1288, 1239, 1173, 1031 cm^{-1}; HRMS (ESI): *m/z* calcd for $C_{15}H_{17}O_4N_2S$ [*M*+H]$^+$: 321.0904, found: 321.0903.

Ethyl (*Z*)-2-(3-hydroxy-2-((2-phenoxyacetyl)imino)-2,3-dihydrothiazol-4-yl)acetate (22b)

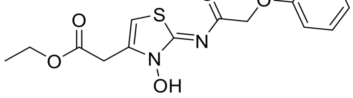

According to General Procedure B, *N*-hydroxythiazole **22b** (182 mg, 0.54 mmol, 82%) was obtained from thiazole **22a** (320 mg, 0.66 mmol), following column chromatography (10 g Sfär Silica D; 35

mL/min; 100% dichloromethane (2 CV), followed by a linear gradient (12 CV): 0%→4% methanol in dichloromethane).

White solid, m.p.: 150-151 °C; ^{1}H NMR (600 MHz, 300K, $CDCl_3$): δ = 7.34 – 7.29 (m, 2H), 7.04 (t, *J* = 7.5 Hz, 1H), 7.01 (s, 1H), 6.97 (d, *J* = 7.5 Hz, 2H), 4.78 (s, 2H), 4.22 (q, *J* = 7.0 Hz, 2H), 3.85 (s, 2H), 1.28 (t, *J* = 7.0 Hz, 3H) ppm; ^{13}C NMR (151 MHz, 300K, $CDCl_3$): δ = 168.5, 166.4, 156.9, 140.2, 137.6, 130.0, 122.7, 114.9, 107.3, 66.8, 61.7, 32.1, 14.3 ppm; IR (film): $\tilde{\nu}$ = 2981, 1737, 1552, 1372, 1215, 1161, 1083 cm^{-1}; HRMS (ESI): *m/z* calcd for $C_{15}H_{17}O_5N_2S$ $[M+H]^+$: 337.0853, found: 337.0853.

(*Z*)-2-(3-Hydroxy-2-((2-phenoxyacetyl)imino)-2,3-dihydrothiazol-4-yl)acetic acid (22)

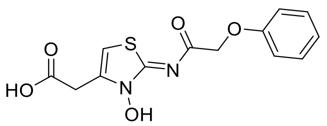

According to General Procedure C, carboxylic acid **22** (65 mg, 0.21 mmol, 70%) was obtained from ethyl ester **22b** (100 mg, 0.30 mmol), following trituration with H_2O (3 × 5 mL; Milli-Q® Ultrapure grade) and MeOH (3 x 5 m; HPLC grade).

White solid, m.p.: 175-177 °C; ^{1}H NMR (600 MHz, 300K, DMSO-*d*$_6$): δ = 7.31 – 7.27 (m, 2H), 7.14 (s, 1H), 6.95 (t, *J* = 7.5 Hz, 1H), 6.92 (d, *J* = 7.5 Hz, 2H), 4.92 (s, 2H), 3.72 (s, 2H) ppm; ^{13}C NMR (151 MHz, 300K, DMSO-*d*$_6$): δ = 170.3 (br), 169.7, 157.9, 147.9 (br), 135.2 (br), 129.5, 121.0, 114.4, 106.7, 67.0, 32.5 ppm; IR (film): $\tilde{\nu}$ = 2981, 1739, 1381, 1251, 1152, 1073 cm^{-1}; HRMS (ESI): *m/z* calcd for $C_{13}H_{13}O_5N_2S$ $[M+H]^+$: 309.0540, found: 309.0540.

Ethyl 2-(2-(2-((*tert*-butoxycarbonyl)(phenyl)amino)acetamido)thiazol-4-yl)acetate (23a)

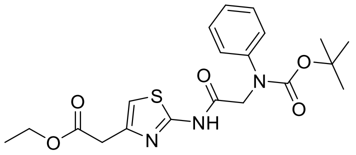

According to General Procedure A, amide **23a** (1.56 g, 3.7 mmol, 76%) was obtained from ethyl 2-(2-aminothiazol-4-yl)acetate **1** (912 mg, 4.9 mmol) and *N*-(*tert*-butoxycarbonyl)-*N*-phenylglycine[59] (1.48 g, 5.9 mmol), following column chromatography (25 g Sfär Silica D; 60 mL/min; 15% ethyl acetate in cyclohexane (3 CV), followed by a linear gradient (12 CV): 15%→50% ethyl acetate in cyclohexane).

White solid, m.p.: 107-110 °C; ^{1}H NMR (400 MHz, 300K, $CDCl_3$): δ = 7.37 – 7.28 (m, 4H), 7.24 – 7.16 (m, 1H), 6.78 (s, 1H), 4.46 (s, 2H), 4.16 (q, *J* = 7.0 Hz, 2H), 3.69 (s, 2H), 1.42 (s, 9H), 1.24 (t, *J* = 7.0 Hz, 3H) ppm; ^{13}C NMR (101 MHz, 300K, $CDCl_3$): δ = 170.6, 167.5, 157.5, 155.0, 143.4, 142.6, 129.1, 126.7, 126.3, 111.2, 82.1, 61.2, 54.2, 37.1, 28.3, 14.3 ppm; IR (film): $\tilde{\nu}$ = 2979, 1697, 1549, 1368, 1272, 1153 cm^{-1}; HRMS (ESI): *m/z* calcd for $C_{20}H_{26}O_5N_3S$ $[M+H]^+$: 420.1588, found: 420.1583.

Ethyl (*Z*)-2-(2-((*N*-(tert-butoxycarbonyl)-*N*-phenylglycyl)imino)-3-hydroxy-2,3-dihydrothiazol-4-yl)acetate (23b)

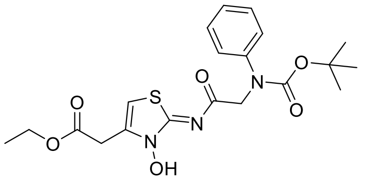

According to General Procedure B, *N*-hydroxythiazole **23b** (320 mg, 0.74 mmol, 62%) was obtained from thiazole **23a** (500 mg, 1.2 mmol), following column chromatography (10 g Sfär Silica D; 35 mL/min; 100% dichloromethane (3 CV), followed by a linear gradient (20 CV): 0%→4% methanol in dichloromethane).

Yellow solid, m.p.: 163-165 °C; ^{1}H NMR (600 MHz, 300K, $CDCl_3$): δ = 7.36 – 7.30 (m, 4H), 7.21 – 7.18 (m, 1H), 6.98 (s, 1H), 4.68 (s, 2H), 4.16 (q, *J* = 7.0 Hz, 2H), 3.83 (s, 2H), 1.42 (s, 9H), 1.25 (t, *J* = 7.0 Hz, 3H) ppm; ^{13}C NMR (151 MHz, 300K, $CDCl_3$): δ = 168.5 (br), 168.4, 154.7 (br), 144.3 (br), 142.6, 136.7, 129.0, 126.7, 126.6, 107.5, 81.7, 61.7, 53.9, 32.1, 28.3, 14.3 ppm; IR (film): $\tilde{\nu}$ = 2978, 1738, 1697, 1550, 1369, 1156 cm^{-1}; HRMS (ESI): *m/z* calcd for $C_{20}H_{26}O_6N_3S$ $[M+H]^+$: 436.1537, found: 436.1535.

Ethyl (*Z*)-2-(3-hydroxy-2-((phenylglycyl)imino)-2,3-dihydrothiazol-4-yl)acetate (23c)

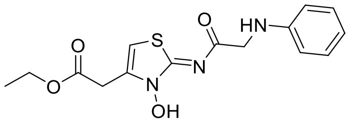

To a stirred solution of *N*-Boc protected aniline **23b** (250 mg, 0.57 mmol, 1.0 equiv.) in dichloromethane (2.0 mL; HPLC grade) at 0 °C under an ambient atmosphere was added trifluoroacetic acid (0.44 mL, 650 mg, 5.7 mmol, 10.0 equiv.). The reaction mixture was allowed to warm to room temperature and stirred for 2 h before the solvent was removed under reduced pressure. The crude residue was redissolved with ethyl acetate and washed with saturated aqueous $NaHCO_3$ solution and brine, dried over anhydrous Na_2SO_4, filtered, and evaporated. The crude residue was purified using column chromatography (10 g Sfär Silica D; 35 mL/min; 100% dichloromethane (2 CV), followed by a linear gradient (20 CV): 0%→4% methanol in dichloromethane) to afford aniline **23c** (2.05 g, 8.4 mmol, 75%).

White solid, m.p.: 143-145 °C; ^{1}H NMR (600 MHz, 300K, $CDCl_3$): δ = 7.17 – 7.12 (m, 2H), 6.96 (s, 1H), 6.74 (t, *J* = 7.5 Hz, 1H), 6.54 (d, *J* = 7.5 Hz, 2H), 4.15 (q, *J* = 7.0 Hz, 2H), 4.06 (s, 2H), 3.81 (s, 2H), 1.24 (t, *J* = 7.0 Hz, 3H) ppm; ^{13}C NMR (151 MHz, 300K, $CDCl_3$): δ = 170.4, 168.7, 146.8, 142.8, 137.0, 129.5, 119.0, 113.2, 107.5, 61.8, 48.2, 32.2, 14.2 ppm; IR (film): $\tilde{\nu}$ = 3108, 1734, 1694, 1604, 1547, 1358, 1177 cm^{-1}; HRMS (ESI): *m/z* calcd for $C_{15}H_{18}O_4N_3S$ [*M*+H]$^+$: 336.1018, found: 336.1013.

(*Z*)-2-(3-Hydroxy-2-((phenylglycyl)imino)-2,3-dihydrothiazol-4-yl)acetic acid (23)

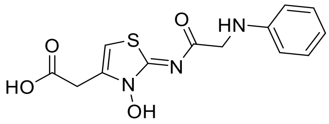

According to General Procedure C, carboxylic acid **23** (67 mg, 0.22 mmol, 73%) was obtained from ethyl ester **23c** (100 mg, 0.30 mmol), following trituration with H_2O (3 × 5 mL; Milli-Q® Ultrapure grade) and MeOH (3 × 5 mL; HPLC grade).

White solid, m.p.: 180-183 °C; ^{1}H NMR (600 MHz, 300K, DMSO-d_6): δ = 7.24 (s, 1H), 7.12 – 7.07 (m, 2H), 6.60 – 6.56 (m, 3H), 6.09 (br s, 1H), 4.11 (s, 2H), 3.73 (s, 2H) ppm; ^{13}C NMR (151 MHz, 300K, DMSO-d_6): δ = 171.0 (br), 169.6, 148.0, 142.9 (br), 136.4, 128.9, 116.6, 112.3, 107.6, 46.5, 32.5 ppm; IR (film): $\tilde{\nu}$ = 3089, 1705, 1603, 1558, 1506, 1368, 1242, 1181 cm^{-1}; HRMS (ESI): *m/z* calcd for $C_{13}H_{14}O_4N_3S$ [*M*+H]$^+$: 308.0700, found: 308.0699.

***tert*-Butyl 3-((4-(2-ethoxy-2-oxoethyl)thiazol-2-yl)carbamoyl)azetidine-1-carboxylate (24a)**

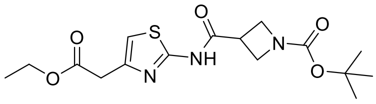

According to General Procedure A, amide **24a** (1.83 g, 5.0 mmol, 99%) was obtained from ethyl 2-(2-aminothiazol-4-yl)acetate **1** (931 mg, 5.0 mmol) and 1-(*tert*-butoxycarbonyl)azetidine-3-carboxylic acid (1.21 g, 6.0 mmol), following column chromatography (50 g Sfär Silica D; 120 mL/min; 100% cyclohexane (2 CV), followed by a linear gradient (15 CV): 0%→30% acetone in cyclohexane).

Clear oil; ^{1}H NMR (600 MHz, 300K, $CDCl_3$): δ = 6.77 (s, 1H), 4.28 – 4.01 (m, 6H), 3.66 (s, 2H), 3.53 – 3.47 (m, 1H), 1.42 (s, 9H), 1.22 (t, *J* = 7.0 Hz, 3H) ppm; ^{13}C NMR (151 MHz, 300K, $CDCl_3$): δ = 170.9, 170.1, 157.9, 156.3, 143.5, 111.2, 80.1, 61.4, 51.6, 37.3, 33.1, 28.5, 14.2 ppm; IR (film): $\tilde{\nu}$ = 2980, 1695, 1551, 1367, 1268, 1145, 1031 cm^{-1}; HRMS (ESI): *m/z* calcd for $C_{16}H_{24}O_5N_3S$ [*M*+H]$^+$: 370.1431, found: 370.1424.

Ethyl 2-(2-(azetidine-3-carboxamido)thiazol-4-yl)acetate hydrochloride (24b)

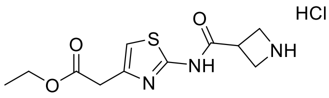

According to General Procedure D, amine HCl salt **24b** (1.50 g, 4.9 mmol, 83%) was obtained from *N*-Boc protected amine **24a** (2.20 g, 5.9 mmol). **24b** was used in the subsequent reaction without further purification.

White solid; m.p.: 115-118 °C; ^{1}H NMR (400 MHz, D_2O): δ = 7.15 (s, 1H), 4.36 – 4.30 (m, 3H), 4.11 (q, *J* = 7.0 Hz, 2H), 3.84 – 3.79 (m, 2H), 3.64 (s, 2H), 3.60 – 3.47 (m, 1H), 1.15 (t, *J* = 7.0 Hz, 3H) ppm; ^{13}C NMR (151 MHz, D_2O): δ = 171.9, 170.2, 159.6, 138.8, 113.2, 62.6, 47.6, 35.6, 34.7, 13.3 ppm; IR (film): $\tilde{\nu}$ = 2854, 2633, 1729, 1699, 1567, 1181, 1120 cm^{-1}; HRMS (ESI): *m/z* calcd for $C_{11}H_{16}O_3N_3S$ [*M*-Cl]$^{+}$: 270.0907, found: 270.0905.

Ethyl 2-(2-(1-(phenylsulfonyl)azetidine-3-carboxamido)thiazol-4-yl)acetate (24c)

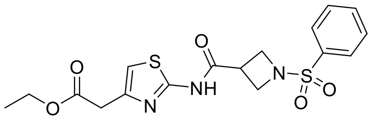

According to General Procedure E, sulfonamide **24c** (245 mg, 0.60 mmol, 34%) was obtained from amine HCl salt **24b** (539 mg, 2.0 mmol) and benzenesulfonyl chloride (0.31 mL, 424 mg, 2.4 mmol), following column chromatography (25 g Sfär Silica D; 30 mL/min; 100% dichloromethane (2 CV), followed by a linear gradient (15 CV): 0%→50% ethyl acetate in dichloromethane).

Yellow oil; ^{1}H NMR (600 MHz, 300K, $CDCl_3$): δ = 7.86 (d, *J* = 8.0 Hz, 2H), 7.67 (t, *J* = 8.0 Hz, 1H), 7.61 – 7.57 (m, 2H), 6.77 (s, 1H), 4.16 (q, *J* = 7.0 Hz, 2H), 4.07 – 4.04 (m, 2H), 4.02 – 3.99 (m, 2H), 3.66 (s, 2H), 3.44 – 3.38 (m, 1H), 1.25 (t, *J* = 7.0 Hz, 3H) ppm; ^{13}C NMR (151 MHz, 300K, $CDCl_3$): δ = 170.6, 168.3, 157.5, 143.4, 134.4, 133.7, 129.5, 128.5, 111.4, 61.4, 52.8, 37.1, 33.1, 14.3 ppm; IR (film): $\tilde{\nu}$ = 2981, 1734, 1686, 1557, 1342, 1161, 1028 cm^{-1}; HRMS (ESI): *m/z* calcd for $C_{17}H_{20}O_5N_3S_2$ [*M*+H]$^{+}$: 410.0839, found: 410.0837.

Ethyl (*Z*)-2-(3-hydroxy-2-((1-(phenylsulfonyl)azetidine-3-carbonyl)imino)-2,3-dihydrothiazol-4-yl)acetate (24d)

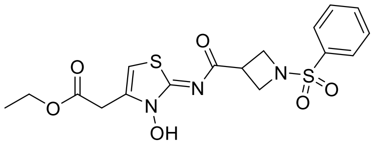

According to General Procedure B, *N*-hydroxythiazole **24d** (104 mg, 0.24 mmol, 56%) was obtained from thiazole **24c** (180 mg, 0.44 mmol) following column chromatography (10 g Sfär Silica D; 35 mL/min; 100% dichloromethane (2 CV), followed by a linear gradient (20 CV): 0%→10% methanol in dichloromethane).

Yellow oil; ^{1}H NMR (600 MHz, 300K, DMSO-d_6): δ = 7.83 (d, *J* = 7.5 Hz, 2H), 7.80 – 7.75 (m, 1H), 7.72 – 7.68 (m, 2H), 7.17 (s, 1H), 4.08 (q, *J* = 7.0 Hz, 2H), 3.90 – 3.86 (m, 2H), 3.83 (dd, *J* = 8.0, 6.5 Hz, 2H), 3.74 (s, 2H), 3.66 – 3.58 (m, 1H), 1.17 (t, *J* = 7.0 Hz, 3H) ppm; ^{13}C NMR (151 MHz, DMSO-d_6) δ 171.2 (br), 168.4, 144.6 (br), 135.8, 133.6 (2C), 129.5, 128.2, 107.5, 60.6, 52.8, 32.5, 31.8, 14.0 ppm; IR (film): $\tilde{\nu}$ = 2981, 1735, 1653, 1558, 1342, 1162 cm^{-1}; HRMS (ESI): *m/z* calcd for $C_{17}H_{20}O_6N_3S_2$ [*M*+H]$^{+}$: 426.0788, found: 426.0793.

(*Z*)-2-(3-Hydroxy-2-((1-(phenylsulfonyl)azetidine-3-carbonyl)imino)-2,3-dihydrothiazol-4-yl)acetic acid (24)

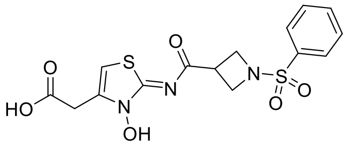

According to General Procedure C, carboxylic acid **24** (28 mg, 0.07 mmol, 44%) was obtained from ethyl ester **24d** (68 mg, 0.16 mmol), following trituration with H_2O (3 × 5 mL; Milli-Q® Ultrapure grade) and MeOH (3 × 5 mL; HPLC grade).

White solid; m.p.: 170-173 °C; ^{1}H NMR (600 MHz, 300K, DMSO-d_6): δ = 7.83 (d, *J* = 8.0 Hz, 2H), 7.78 (t, *J* = 8.0 Hz, 1H), 7.72 – 7.68 (m, 2H), 7.18 (s, 1H), 3.91 – 3.87 (m, 2H), 3.85 – 3.82 (m, 2H), 3.70 (s, 2H), 3.66 – 3.61 (m, 1H) ppm; ^{13}C NMR (151 MHz, 300K, DMSO-d_6): δ = 171.1 (br), 169.6, 145.0 (br), 135.8 (br), 133.6, 133.6, 129.5, 129.2, 128.2, 107.5, 52.7, 32.5, 32.4 ppm; IR (film): $\tilde{\nu}$ = 3250, 2980, 1686, 1545, 1361, 1161, 1092 cm^{-1}; HRMS (ESI): *m/z* calcd for $C_{15}H_{16}O_6N_3S_2$ [*M*+H]$^+$: 398.0475, found: 398.0474.

***tert*-Butyl 3-((4-(2-ethoxy-2-oxoethyl)thiazol-2-yl)carbamoyl)-3-methylazetidine-1-carboxylate (25a)**

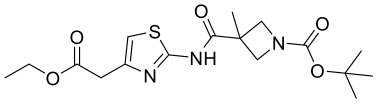

According to General Procedure A, amide **25a** (1.04 g, 2.7 mmol, 68%) was obtained from ethyl 2-(2-aminothiazol-4-yl)acetate **1** (745 mg, 4.0 mmol) and 1-(*tert*-butoxycarbonyl)-3-methylazetidine-3-carboxylic acid (1.03 g, 4.8 mmol) following column chromatography (50 g Sfär Silica D; 100 mL/min; 100% cyclohexane (2 CV), followed by a linear gradient (15 CV): 0%→30% acetone in cyclohexane).

White solid; m.p.: 160-161 °C; ^{1}H NMR (600 MHz, 300K, $CDCl_3$): δ = 6.76 (s, 1H), 4.26 (d, *J* = 8.5 Hz, 2H), 4.14 (q, *J* = 7.0 Hz, 2H), 3.75 (d, *J* = 9.0 Hz, 2H), 3.65 (s, 2H), 1.60 (s, 3H), 1.40 (s, 9H), 1.22 (t, *J* = 7.0 Hz, 3H) ppm; ^{13}C NMR (151 MHz, 300K, $CDCl_3$): δ = 172.5, 170.7, 157.7, 156.5, 143.6, 111.2, 80.2, 61.3, 58.2 (br), 39.5, 37.2, 28.4, 22.7, 14.2 ppm; IR (film): $\tilde{\nu}$ = 2978, 1679, 1548, 1411, 1164 cm^{-1}; HRMS (ESI): *m/z* calcd for $C_{17}H_{26}O_5N_3S$ [*M*+H]$^+$: 384.1588, found: 384.1603.

Ethyl 2-(2-(3-methylazetidine-3-carboxamido)thiazol-4-yl)acetate hydrochloride (25b)

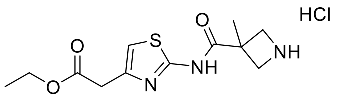

According to General Procedure D, amine HCl salt **25b** (810 mg, 2.5 mmol, 99%) was obtained from *N*-Boc protected amine **25a** (976 mg, 2.6 mmol). **25b** was used in the subsequent reaction without further purification.

Clear oil; ^{1}H NMR (600 MHz, D_2O): δ = 7.12 (s, 1H), 4.59 (d, *J* = 11.5 Hz, 2H), 4.23 (q, *J* = 7.0 Hz, 2H), 4.10 (d, *J* = 11.5 Hz, 2H), 3.86 (s, 2H), 1.78 (s, 3H), 1.29 (t, *J* = 7.0 Hz, 3H) ppm; ^{13}C NMR (151 MHz, D_2O): δ = 173.2, 173.1, 159.1, 142.2, 112.9, 62.5, 53.7, 42.5, 35.9, 21.9, 13.3 ppm; IR (film): $\tilde{\nu}$ = 2855, 1733, 1560, 1308, 1202, 1147 cm^{-1}; HRMS (ESI): *m/z* calcd for $C_{12}H_{18}O_3N_3S$ [*M*-Cl]$^+$: 284.1063, found: 284.1067.

Ethyl 2-(2-(3-methyl-1-(phenylsulfonyl)azetidine-3-carboxamido)thiazol-4-yl)acetate (25c)

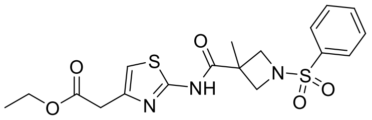

According to General Procedure E, sulfonamide **25c** (779 mg, 1.8 mmol, 60%) was obtained from amine HCl salt **25b** (979 mg, 3.1 mmol) and benzenesulfonyl chloride (0.47 mL, 648 mg, 3.7 mmol) following column chromatography (25 g Sfär Silica D; 60 mL/min;

100% dichloromethane (2 CV), followed by a linear gradient (15 CV): 0%→30% ethyl acetate in dichloromethane).

Yellow oil; ^{1}H NMR (600 MHz, 300K, $CDCl_3$): δ = 7.88 – 7.84 (m, 2H), 7.69 – 7.63 (m, 1H), 7.61 – 7.56 (m, 2H), 6.80 (s, 1H), 4.19 (q, *J* = 7.0 Hz, 2H), 4.13 (d, *J* = 8.5 Hz, 2H), 3.71 – 3.66 (m, 4H), 1.47 (s, 3H), 1.27 (t, *J* = 7.0 Hz, 3H) ppm; ^{13}C NMR (151 MHz, 300K, $CDCl_3$): δ = 171.1, 170.6, 157.5, 143.5, 134.3, 133.8, 129.5, 128.5, 111.6, 61.4, 59.1, 39.3, 37.2, 22.3, 14.3 ppm; IR (film): $\tilde{\nu}$ = 3273, 1734, 1686, 1547, 1341, 1208 cm^{-1}; HRMS (ESI): *m/z* calcd for $C_{18}H_{22}O_5N_3S_2$ [*M*+H]$^+$: 424.0995, found: 424.0996.

Ethyl (*Z*)-2-(3-hydroxy-2-((3-methyl-1-(phenylsulfonyl)azetidine-3-carbonyl)imino)-2,3-dihydrothiazol-4-yl)acetate (25d)

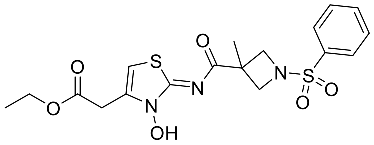

According to General Procedure B, *N*-hydroxythiazole **25d** (538 mg, 1.2 mmol, 68%) was obtained from thiazole **25c** (759 mg, 1.8 mmol), following column chromatography (25 g Sfär Silica D; 60 mL/min; 100% cyclohexane (2 CV), followed by a linear gradient (15 CV): 0% → 100% acetone in cyclohexane).

Yellow oil; ^{1}H NMR (600 MHz, 300K, DMSO-*d*$_6$): δ = 7.86 – 7.78 (m, 2H), 7.71 (t, *J* = 7.5 Hz, 1H), 7.67 – 7.63 (m, 2H), 7.00 (s, 1H), 4.11 (q, *J* = 7.0 Hz, 2H), 4.03 (d, *J* = 8.5 Hz, 2H), 3.76 (s, 2H), 3.57 (d, *J* = 8.5 Hz, 2H), 1.24 (s, 3H), 1.19 (t, *J* = 7.0 Hz, 3H) ppm; ^{13}C NMR (151 MHz, 300K, DMSO-*d*$_6$): δ = 178.4 (br), 168.4, 154.8 (br), 133.6, 133.5, 133.3 (br), 129.4, 128.1, 105.8, 60.8, 59.4, 40.1, 32.0, 23.0, 14.0 ppm; IR (film): $\tilde{\nu}$ = 2986, 1736, 1686, 1544, 1350, 1166, 1092 cm^{-1}; HRMS (ESI): *m/z* calcd for $C_{18}H_{22}O_6N_3S_2$ [*M*+H]$^+$: 440.0945, found: 440.0950.

(*Z*)-2-(3-Hydroxy-2-((3-methyl-1-(phenylsulfonyl)azetidine-3-carbonyl)imino)-2,3-dihydrothiazol-4-yl)acetic acid (25)

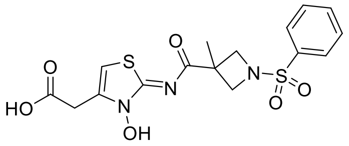

According to General Procedure C, carboxylic acid **25** (247 mg, 0.60 mmol, 57%) was obtained from ethyl ester **25d** (460 mg, 1.1 mmol), following trituration with H_2O (3 × 5 mL; Milli-Q® Ultrapure grade) and MeOH (3 × 5 mL; HPLC grade).

White solid; m.p.: 156-158 °C; ^{1}H NMR (600 MHz, 300K, DMSO-*d*$_6$): δ = 7.85 – 7.79 (m, 2H), 7.74 – 7.68 (m, 1H), 7.68 – 7.63 (m, 2H), 6.99 (s, 1H), 4.04 (d, *J* = 8.5 Hz, 2H), 3.69 (s, 2H), 3.57 (d, *J* = 8.5 Hz, 2H), 1.25 (s, 3H) ppm; ^{13}C NMR (151 MHz, 300K, DMSO-*d*$_6$): δ = 178.2 (br), 169.7, 154.7 (br), 133.8 (br), 133.6, 133.5, 129.4, 128.1, 107.5, 59.4, 48.6, 32.3, 23.0 ppm; IR (film): $\tilde{\nu}$ = 2917, 1687, 1554, 1344, 1165, 1093 cm^{-1}; HRMS (ESI): *m/z* calcd for $C_{16}H_{18}O_6N_3S_2$ [*M*+H]$^+$: 412.0632, found: 412.0634.

(±)-Ethyl 2-(2-(1-(phenylsulfonyl)pyrrolidine-3-carboxamido)thiazol-4-yl)acetate (26a)

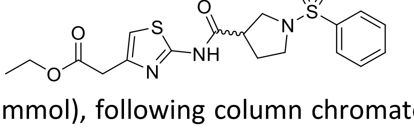

According to General Procedure E, racemic sulfonamide **26a** (560 mg, 1.3 mmol, 75%) was obtained from amine HCl salt **32** (567 mg, 2.0 mmol) and benzenesulfonyl chloride (0.31 mL, 424 mg, 2.4 mmol), following column chromatography (25 g Sfär Silica D; 60 mL/min; 100% dichloromethane (2 CV), followed by a linear gradient (15 CV): 0%→50% ethyl acetate in dichloromethane).

Yellow oil; ^{1}H NMR (600 MHz, 300K, $CDCl_3$): δ = 7.84 (d, *J* = 8.0 Hz, 2H), 7.63 – 7.57 (m, 1H), 7.55 – 7.51 (m, 2H), 6.79 (s, 1H), 4.18 (q, *J* = 7.0 Hz, 2H), 3.72 – 3.66 (m, 3H), 3.46 – 3.33 (m, 3H), 3.07 – 3.03 (m, 1H), 2.19 – 2.06 (m, 2H), 1.27 (t, *J* = 7.0 Hz, 3H) ppm; ^{13}C NMR (151 MHz, 300K, $CDCl_3$): δ = 170.5, 169.5, 157.4, 143.4, 136.3, 133.2, 129.4, 127.8, 111.4, 61.4, 50.5, 47.6, 44.1, 37.1, 28.9, 14.3 ppm; IR (film): $\tilde{\nu}$

= 3280, 2981, 1734, 1686, 1547, 1341, 1162, 1027 cm^{-1}; HRMS (ESI): *m/z* calcd for $C_{18}H_{22}O_5N_3S_2$ [*M*+H]$^+$: 424.0995, found: 424.0988.

(±)-Ethyl (*Z*)-2-(3-hydroxy-2-((1-(phenylsulfonyl)pyrrolidine-3-carbonyl)imino)-2,3-dihydrothiazol-4-yl)acetate (26b)

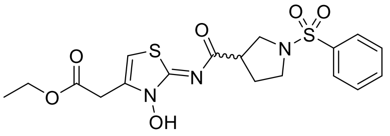

According to General Procedure B, racemic *N*-hydroxythiazole **26b** (316 mg, 0.72 mmol, 56%) was obtained from thiazole **26a** (548 mg, 1.3 mmol), following column chromatography (10 g Sfär Silica D; 35 mL/min; 100% dichloromethane (2 CV), followed by a linear gradient (20 CV): 0%→10% methanol in dichloromethane).

White solid, m.p.: 67-70 °C; ^{1}H NMR (600 MHz, 300K, DMSO-d_6): δ = 7.80 (d, *J* = 8.0 Hz, 2H), 7.70 (t, *J* = 8.0 Hz, 1H), 7.64 – 7.60 (m, 2H), 7.19 (s, 1H), 4.09 (q, *J* = 7.0 Hz, 2H), 3.75 (s, 2H), 3.51 (dd, *J* = 10.0, 7.5 Hz, 1H), 3.39 – 3.14 (m, 4H), 2.04 – 1.98 (m, 1H), 1.93 – 1.87 (m, 1H), 1.18 (t, *J* = 7.0 Hz, 3H) ppm; ^{13}C NMR (151 MHz, 300K, DMSO-d_6): δ = 172.3 (br), 168.5, 143.3 (br), 136.0 (br), 135.7, 133.1, 129.4, 127.3, 107.6, 60.6, 50.4, 47.6, 43.0, 31.8, 28.5, 14.0; IR (film): $\tilde{\nu}$ = 2981, 1736, 1686, 1547, 1345, 1162 cm^{-1}; HRMS (ESI): *m/z* calcd for $C_{18}H_{22}O_6N_3S_2$ [*M*+H]$^+$: 440.0945, found: 440.0942.

(±)-(*Z*)-2-(3-Hydroxy-2-((1-(phenylsulfonyl)pyrrolidine-3-carbonyl)imino)-2,3-dihydrothiazol-4-yl)acetic acid (26)

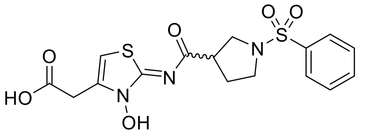

According to General Procedure C, racemic carboxylic acid **26** (70 mg, 0.17 mmol, 41%) was obtained from ethyl ester **26b** (182 mg, 0.41 mmol).

White solid, m.p.: 185-187 °C; ^{1}H NMR (600 MHz, 300K, DMSO-d_6): δ = 7.82 (d, *J* = 8.0 Hz, 2H), 7.73 – 7.68 (m, 1H), 7.64 – 7.61 (m, 2H), 7.21 (s, 1H), 3.72 (s, 2H), 3.54 – 3.49 (m, 1H), 3.39 – 3.35 (m, 1H), 3.33 – 3.29 (m, 1H), 3.29 – 3.23 (m, 1H), 3.25 – 3.16 (m, 1H), 2.05 – 1.99 (m, 1H), 1.94 – 1.88 (m, 1H) ppm; ^{13}C NMR (151 MHz, 300K, DMSO-d_6): δ = 172.2 (br), 169.7, 144.2 (br), 136.0 (br), 135.7, 133.1, 129.4, 127.4, 107.7, 50.4, 47.6, 43.0, 32.6, 28.5 ppm; IR (film): $\tilde{\nu}$ = 2981, 1734, 1701, 1556, 1338, 1157 cm^{-1}; HRMS (ESI): *m/z* calcd for $C_{16}H_{18}O_6N_3S_2$ [*M*+H]$^+$: 412.0632, found: 412.0630.

(±)-*tert*-Butyl 3-((4-(2-ethoxy-2-oxoethyl)thiazol-2-yl)carbamoyl)piperidine-1-carboxylate (27a)

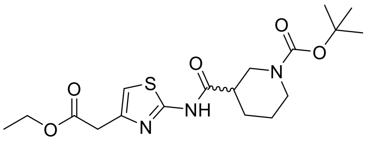

According to General Procedure A, racemic amide **27a** (1.60 g, 4.0 mmol, 80%) was obtained from ethyl 2-(2-aminothiazol-4-yl)acetate **1** (931 mg, 5.0 mmol) and 1-(*tert*-butoxycarbonyl)-3-piperidinecarboxylic acid (1.38 g, 6.0 mmol), following column chromatography (50 g Sfär Silica D; 120 mL/min; 100% cyclohexane (2 CV), followed by a linear gradient (12 CV): 0%→20% acetone in cyclohexane).

Yellow oil; ^{1}H NMR (600 MHz, 300K, $CDCl_3$): δ = 6.77 (s, 1H), 4.16 (q, *J* = 7.0 Hz, 2H), 4.07 – 3.97 (m, 1H), 3.90 – 3.74 (m, 1H), 3.66 (s, 2H), 3.24 (dd, *J* = 13.5, 9.5 Hz, 1H), 2.96 (s, 1H), 2.55 (s, 1H), 2.06 – 1.79 (m, 2H), 1.79 – 1.62 (m, 1H), 1.49 – 1.42 (m, 10H), 1.24 (t, *J* = 7.0 Hz, 3H) ppm; ^{13}C NMR (151 MHz, 300K, $CDCl_3$): δ = 171.2, 170.6, 157.6, 154.9 (br), 143.5, 111.0, 80.4, 61.2, 45.7 (br), 44.5 (br), 42.8, 37.2, 28.5, 27.7 (br), 24.1, 14.3 ppm; IR (film): $\tilde{\nu}$ = 2980, 1735, 1685, 1544, 1257, 1165, 1032 cm^{-1}; HRMS (ESI): *m/z* calcd for $C_{18}H_{28}O_5N_3S$ [*M*+H]$^+$: 398.1744, found: 398.1741.

(±)-Ethyl 2-(2-(piperidine-3-carboxamido)thiazol-4-yl)acetate hydrochloride (27b)

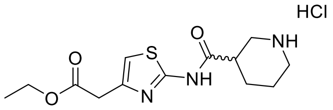

According to General Procedure D, racemic amine HCl salt **27b** (740 mg, 2.2 mmol, 55%) was obtained from *N*-Boc protected amine **27a** (1.60 g, 4.0 mmol) **27b** was used in the subsequent reaction without further purification.

Yellow solid; m.p.: 64-66 °C; ^{1}H NMR (600 MHz, D_2O): δ = 7.11 (s, 1H), 4.20 (q, *J* = 7.0 Hz, 2H), 3.84 (s, 2H), 3.53 – 3.48 (m, 1H), 3.38 – 3.31 (m, 2H), 3.18 – 3.10 (m, 2H), 2.21 – 2.14 (m, 1H), 2.02 – 1.79 (m, 3H), 1.25 (t, *J* = 7.0 Hz, 3H) ppm; ^{13}C NMR (151 MHz, D_2O): δ = 174.3, 172.7, 159.2, 140.6, 112.8, 62.5, 44.0, 43.8, 38.8, 35.3, 25.3, 20.4, 13.2 ppm; IR (film): $\tilde{\nu}$ = 3382, 2853, 2633, 1729, 1563, 1180, 1120 cm^{-1}; HRMS (ESI): *m/z* calcd for $C_{13}H_{20}O_3N_3S$ [*M*-Cl]$^+$: 298.1220, found: 298.1217.

(±)-Ethyl 2-(2-(1-(phenylsulfonyl)piperidine-3-carboxamido)thiazol-4-yl)acetate (27c)

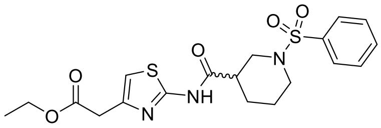

According to General Procedure E, racemic sulfonamide **27c** (436 mg, 1.0 mmol, 56%) was obtained from amine HCl salt **27b** (595 mg, 2.0 mmol) and benzenesulfonyl chloride (0.31 mL, 424 mg, 2.4 mmol), following column chromatography (25 g Sfär Silica D; 60 mL/min; 100% dichloromethane (2 CV), followed by a linear gradient (15 CV): 0%→50% ethyl acetate in dichloromethane).

White solid, m.p.: 146-148 °C; ^{1}H NMR (600 MHz, 300K, $CDCl_3$): δ = 7.76 (d, *J* = 8.0 Hz, 2H), 7.61 (t, *J* = 8.0 Hz, 1H), 7.56 – 7.52 (m, 2H), 6.82 (s, 1H), 4.19 (q, *J* = 7.0 Hz, 2H), 3.91 – 3.85 (m, 1H), 3.74 – 3.67 (m, 3H), 2.73 – 2.68 (m, 1H), 2.62 – 2.58 (m, 1H), 2.40 – 2.35 (m, 1H), 1.97 – 1.93 (m, 1H), 1.85 – 1.82 (m, 1H), 1.75 – 1.65 (m, 1H), 1.58 – 1.52 (m, 1H), 1.27 (t, *J* = 7.0 Hz, 3H) ppm; ^{13}C NMR (151 MHz, 300K, $CDCl_3$): δ = 170.7, 170.5, 157.3, 143.6, 136.0, 133.1, 129.4, 127.8, 111.4, 61.4, 47.9, 46.4, 42.7, 37.2, 27.1, 24.0, 14.3 ppm; IR (film): $\tilde{\nu}$ = 3275, 2939, 1734, 1684, 1545, 1332, 1170 cm^{-1}; HRMS (ESI): *m/z* calcd for $C_{19}H_{24}O_5N_3S_2$ [*M*+H]$^+$: 438.1152, found: 438.1147.

(±)-Ethyl (*Z*)-2-(3-hydroxy-2-((1-(phenylsulfonyl)piperidine-3-carbonyl) imino)-2,3-dihydrothiazol-4-yl)acetate (27d)

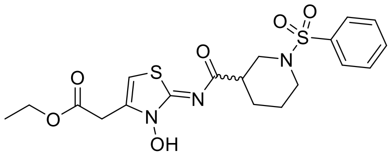

According to General Procedure B, racemic *N*-hydroxythiazole **27d** (161 mg, 0.36 mmol, 73%) was obtained from thiazole **27c** (213 mg, 0.49 mmol), following column chromatography (10 g Sfär Silica D; 35 mL/min; 100% dichloromethane (2 CV), followed by a linear gradient (20 CV): 0%→10% methanol in dichloromethane).

White solid, 101-104 °C; ^{1}H NMR (600 MHz, 300K, $CDCl_3$): δ = 7.30 – 7.24 (m, 2H), 7.15 – 7.09 (m, 1H), 7.08 – 7.01 (m, 2H), 6.58 (s, 1H), 3.72 (q, *J* = 7.0 Hz, 2H), 3.50 – 3.37 (m, 3H), 3.24 – 3.17 (m, 1H), 2.88 – 2.81 (m, 1H), 2.13 – 2.07 (m, 1H), 1.88 – 1.82 (m, 1H), 1.65 – 1.58 (m, 1H), 1.43 – 1.20 (m, 2H), 1.09 – 0.95 (m, 1H), 0.80 (t, *J* = 7.0 Hz, 3H) ppm; ^{13}C NMR (151 MHz, 300K, $CDCl_3$): δ = 172.5, 168.4, 146.2, 136.6, 136.1, 133.0, 129.4, 127.8, 108.0, 61.8, 47.9, 46.4, 42.0, 32.2, 27.1, 24.0, 14.3 ppm; IR (film): $\tilde{\nu}$ = 2981, 1735, 1653, 1542, 1355, 1172, 1093 cm^{-1}; HRMS (ESI): *m/z* calcd for $C_{19}H_{24}O_6N_3S_2$ [*M*+H]$^+$: 454.1101, found: 454.1096.

(±)-(*Z*)-2-(3-Hydroxy-2-((1-(phenylsulfonyl)piperidine-3-carbonyl)imino)-2,3-dihydrothiazol-4-yl)acetic acid (27)

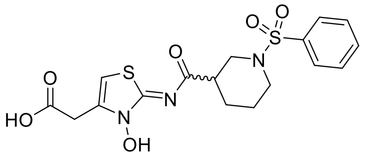

According to General Procedure C, racemic carboxylic acid **27** (30 mg, 0.07 mmol, 42%) was obtained from ethyl ester **27d** (77 mg, 0.17 mmol, 1.0 equiv.), following trituration with H_2O (3 × 5 mL; Milli-Q® Ultrapure grade) and MeOH (3 × 5 mL; HPLC grade).

White solid; m.p.: 181-184 °C; ^{1}H NMR (600 MHz, 300K, DMSO-d_6): δ = 7.79 – 7.70 (m, 3H), 7.68 – 7.64 (m, 2H), 7.21 (s, 1H), 3.81 – 3.76 (m, 1H), 3.72 (s, 2H), 3.59 – 3.56 (m, 1H), 3.05 – 2.95 (m, 1H), 2.35 – 2.31 (m, 1H), 2.24 – 2.20 (m, 1H), 1.91 – 1.85 (m, 1H), 1.79 – 1.75 (m, 1H), 1.54 – 1.44 (m, 1H), 1.35 – 1.28 (m, 1H) ppm; ^{13}C NMR (151 MHz, 300K, DMSO-d_6): δ = 172.9 (br), 169.7, 143.9 (br), 136.1, 135.3, 133.2, 129.4, 127.4, 107.7, 47.8, 46.0, 41.3, 32.7, 26.5, 23.5 ppm; IR (film): $\tilde{\nu}$ = 3198, 2980, 1731, 1686, 1543, 1333, 1165, 1106 cm^{-1}; HRMS (ESI): *m/z* calcd for $C_{17}H_{20}O_6N_3S_2$ [*M*+H]$^+$: 426.0788, found: 426.0783.

(±)-*tert*-Butyl 2-((4-(2-ethoxy-2-oxoethyl)thiazol-2-yl)carbamoyl)pyrrolidine-1-carboxylate (28a)

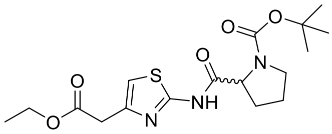

According to General Procedure A, racemic amide **28a** (1.13 g, 3.0 mmol, 37%) was obtained from ethyl 2-(2-aminothiazol-4-yl)acetate **1** (1.49 g, 8.0 mmol) and 1-(*tert*-butoxycarbonyl)pyrrolidine-2-carboxylic acid (2.07 g, 9.6 mmol), following column chromatography (25 g Sfär Silica D; 60 mL/min; 100% cyclohexane (2 CV), followed by a linear gradient (15 CV): 0%→30% acetone in cyclohexane).

Note: Several signals in the ^{13}C NMR spectrum appear as doublets, due to restricted rotation of the *N*-Boc group.

Yellow oil; ^{1}H NMR (600 MHz, 300K, DMSO-d_6): δ = 12.30 – 12.14 (m, 1H), 6.98 (s, 1H), 4.36 – 4.29 (m, 1H), 4.13 – 4.02 (m, 2H), 3.68 (s, 2H), 3.44 – 3.39 (m, 1H), 3.36 – 3.32 (m, 1H), 2.23 – 2.15 (m, 1H), 1.91 – 1.72 (m, 3H), 1.39 (s, 3H), 1.23 (s, 6H), 1.20 – 1.15 (m, 3H) ppm; ^{13}C NMR (151 MHz, 300K, DMSO-d_6): δ = 171.5 (d), 170.0, 157.5 (d), 153.3 (d), 143.7 (d), 110.5 (d), 78.8 (d), 60.3 (d), 59.3 (d), 46.6 (d), 36.7, 30.5 (d), 28.0 (d), 23.7 (d), 14.1 (d) ppm; IR (film): $\tilde{\nu}$ = 2979, 1735, 1698, 1557, 1395, 1265, 1160, 1028 cm^{-1}; HRMS (ESI): *m/z* calcd for $C_{17}H_{26}O_5N_3S$ [*M*+H]$^+$: 384.1588, found: 384.1584.

(±)-Ethyl 2-(2-(pyrrolidine-2-carboxamido)thiazol-4-yl)acetate hydrochloride (28b)

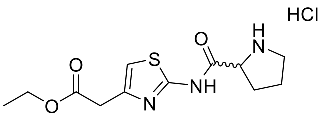

According to General Procedure D, racemic amine HCl salt **28b** (740 mg, 2.3 mmol, 66%) was obtained from *N*-Boc protected amine **28a** (1.34 g, 3.5 mmol). **28b** was used in the subsequent reaction without further purification.

White solid, m.p.: 136-139 °C; ^{1}H NMR (600 MHz, D_2O): δ = 7.12 (s, 1H), 4.70 – 4.64 (m, 1H), 4.18 (q, *J* = 7.0 Hz, 2H), 3.82 (s, 2H), 3.55 – 3.48 (m, 1H), 3.48 – 3.42 (m, 1H), 2.59 – 2.51 (m, 1H), 2.24 – 2.16 (m, 1H), 2.16 – 2.01 (m, 2H), 1.23 (t, *J* = 7.0 Hz, 3H) ppm; ^{13}C NMR (151 MHz, D_2O): δ = 172.5, 168.0, 159.2, 140.6, 113.2, 62.5, 60.1, 46.7, 35.3, 29.4, 23.6, 13.2 ppm; IR (film): $\tilde{\nu}$ = 2981, 2650, 1737, 1708, 1560, 1284, 1204, 1033 cm^{-1}; HRMS (ESI): *m/z* calcd for $C_{12}H_{18}O_3N_3S$ [*M*-Cl]$^+$: 284.1063, found: 284.1060.

(±)-Ethyl 2-(2-(1-(phenylsulfonyl)pyrrolidine-2-carboxamido)thiazol-4-yl)acetate (28c)

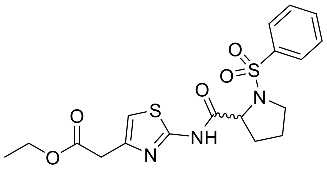

According to General Procedure E, racemic sulfonamide **28c** (406 mg, 0.96 mmol, 77%) was obtained from amine HCl salt **28b** (400 mg, 1.3 mmol) and benzenesulfonyl chloride (0.19 mL, 265 mg, 1.5 mmol), following column chromatography (25 g Sfär Silica D; 60 mL/min; 100% dichloromethane (2 CV), followed by a linear gradient (12 CV): 0%→30% ethyl acetate in dichloromethane).

White solid; m.p.: 183-186 °C; ^{1}H NMR (600 MHz, 300K, DMSO-d_6): δ = 12.35 (s, 1H), 7.93 – 7.84 (m, 2H), 7.76 – 7.70 (m, 1H), 7.67 – 7.63 (m, 2H), 7.03 (s, 1H), 4.34 – 4.28 (m, 1H), 4.09 (q, *J* = 7.0 Hz, 2H), 3.71 (s, 2H), 3.52 – 3.46 (m, 1H), 3.23 – 3.17 (m, 1H), 1.96 – 1.81 (m, 3H), 1.52 – 1.47 (m, 1H), 1.19 (t, *J* = 7.0 Hz, 3H) ppm; ^{13}C NMR (151 MHz, 300K, DMSO-d_6): δ = 170.2, 170.0, 157.3, 143.8, 136.7, 133.3, 129.5, 127.3, 110.9, 60.7, 60.3, 49.1, 36.6, 31.0, 24.4, 14.1 ppm; IR (film): $\tilde{\nu}$ = 2981, 1738, 1540, 1337, 1195, 1165 cm^{-1}; HRMS (ESI): *m/z* calcd for $C_{18}H_{22}O_5N_3S_2$ [*M*+H]$^+$: 424.0995, found: 424.0992.

(±)-Ethyl (*Z*)-2-(3-hydroxy-2-(((phenylsulfonyl)prolyl)imino)-2,3-dihydrothiazol-4-yl)acetate (28d)

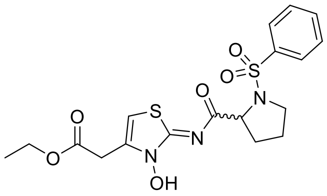

According to General Procedure B, racemic *N*-hydroxythiazole **28d** (111 mg, 0.25 mmol, 43%) was obtained from thiazole **28c** (250 mg, 0.59 mmol), following column chromatography (10 g Sfär Silica D; 35 mL/min; 100% dichloromethane (2 CV), followed by a linear gradient (20 CV): 0%→10% methanol in dichloromethane).

White solid; m.p.: > 170 °C (decomposition); ^{1}H NMR (600 MHz, 300K, $CDCl_3$): δ = 7.93 – 7.88 (m, 2H), 7.66 – 7.61 (m, 1H), 7.58 – 7.54 (m, 2H), 7.01 (s, 1H), 4.46 – 4.43 (m, 1H), 4.23 (q, *J* = 7.0 Hz, 2H), 3.93 – 3.81 (m, 2H), 3.61 – 3.57 (m, 1H), 3.33 – 3.27 (m, 1H), 2.24 – 2.18 (m, 1H), 1.96 – 1.82 (m, 2H), 1.79 – 1.69 (m, 1H), 1.30 (t, *J* = 7.0 Hz, 3H) ppm; ^{13}C NMR (151 MHz, 300K, $CDCl_3$): δ = 169.5, 168.7, 140.6, 137.6, 136.3, 133.7, 129.6, 128.1, 107.2, 61.8, 61.7, 49.9, 32.0, 30.5, 24.8, 14.3 ppm; IR (film): $\tilde{\nu}$ = 2981, 1737, 1697, 1547, 1349, 1161, 1026 cm^{-1}; HRMS (ESI): *m/z* calcd for $C_{18}H_{22}O_6N_3S_2$ [*M*+H]$^+$: 440.0945, found: 440.0949.

(±)-(*Z*)-2-(3-Hydroxy-2-(((phenylsulfonyl)prolyl)imino)-2,3-dihydrothiazol-4-yl)acetic acid (28)

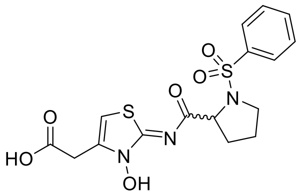

According to General Procedure C, racemic carboxylic acid **28** (47 mg, 0.11 mmol, 69%) was obtained from ethyl ester **28d** (73 mg, 0.17 mmol), following trituration with H_2O (3 × 5 mL; Milli-Q® Ultrapure grade) and MeOH (3 × 5 mL; HPLC grade).

White solid; m.p.: > 190 °C (decomposition); ^{1}H NMR (600 MHz, 300K, DMSO-d_6): δ = 7.91 (d, *J* = 8.0 Hz, 2H), 7.75 – 7.71 (m, 1H), 7.68 – 7.62 (m, 2H), 7.27 (s, 1H), 4.77 – 4.73 (m, 1H), 3.74 (s, 2H), 3.47 – 3.43 (m, 1H), 3.21 – 3.17 (m, 1H), 1.90 - 1.81 (m, 3H), 1.58 – 1.49 (m, 1H) ppm; ^{13}C NMR (151 MHz, 300K, DMSO-d_6): δ = 171.8 (br), 169.7, 143.6 (br), 137.0, 136.3, 133.2, 129.4, 127.3, 107.9, 60.7, 49.0, 32.7, 31.1, 24.3 ppm; IR (film): $\tilde{\nu}$ = 3329, 3095, 1695, 1547, 1323, 1115, 1093 cm^{-1}; HRMS (ESI): *m/z* calcd for $C_{16}H_{18}O_6N_3S_2$ [*M*+H]$^+$: 412.0632, found: 412.0629.

(±)-*tert*-Butyl 3-((4-(2-ethoxy-2-oxoethyl)thiazol-2-yl)carbamoyl)-3-methylpyrrolidine-1-carboxylate (29a)

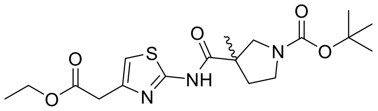

According to General Procedure A, racemic amide **29a** (483 mg, 1.2 mmol, 35%) was obtained from ethyl 2-(2-aminothiazol-4-yl)acetate **1** (652 mg, 3.5 mmol) and 1-(*tert*-butoxycarbonyl)-3-methylpyrrolidine-3-carboxylic acid (963 mg, 4.2 mmol), following column chromatography (25 g Sfär Silica D; 60 mL/min; 100% cyclohexane (2 CV), followed by a linear gradient (15 CV): 0%→30% acetone in cyclohexane).

Note: Several signals in the ^{13}C NMR spectrum appear as doublets, due to restricted rotation of the *N*-Boc group.

Clear oil; ^{1}H NMR (600 MHz, 300K, DMSO-d_6): δ = 12.16 – 12.12 (m, 1H), 7.00 (s, 1H), 4.08 (q, *J* = 7.0 Hz, 2H), 3.78 – 3.73 (m, 1H), 3.69 (s, 2H), 3.26 – 3.13 (m, 2H), 2.43 – 2.24 (m, 1H), 1.93 – 1.83 (m, 1H), 1.41 – 1.37 (m, 9H), 1.35 (s, 3H), 1.18 (t, *J* = 7.0 Hz, 3H) ppm; ^{13}C NMR (151 MHz, 300K, DMSO-d_6): δ = 173.6, 170.0, 158.0, 153.5 (d), 143.6, 110.7, 78.4, 60.3, 54.0 (d), 48.7 (d), 44.4 (d), 36.6, 34.1 (d), 28.1, 21.7, 14.1 ppm; IR (film): $\tilde{\nu}$ = 2977, 1738, 1691, 1547, 1405, 1162, 1029 cm^{-1}; HRMS (ESI): *m/z* calcd for $C_{18}H_{28}O_5N_3S$ [*M*+H]$^+$: 398.1744, found: 398.1743.

(±)-Ethyl 2-(2-(3-methylpyrrolidine-3-carboxamido)thiazol-4-yl)acetate (29b)

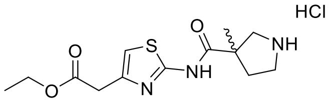

According to General Procedure D, racemic amine HCl salt **29b** (373 mg, 1.1 mmol, 99%) was obtained from *N*-Boc protected amine **29a** (447 mg, 1.1 mmol). **29b** was used in the subsequent reaction without further purification.

Clear oil; ^{1}H NMR (600 MHz, D_2O): δ = 6.97 (s, 1H), 4.13 (q, *J* = 7.0 Hz, 2H), 3.93 – 3.85 (m, 1H), 3.76 – 3.70 (m, 2H), 3.50 – 3.43 (m, 1H), 3.36 – 3.30 (m, 1H), 3.20 – 3.10 (m, 1H), 2.55 – 2.47 (m, 1H), 2.15 – 2.05 (m, 1H), 1.53 – 1.47 (m, 3H), 1.18 (t, *J* = 7.0 Hz, 3H) ppm; ^{13}C NMR (151 MHz, D_2O): δ = 173.4, 159.1, 142.8, 112.6, 112.5, 62.4, 52.9, 49.5, 44.9, 36.2, 34.8, 21.1, 13.2 ppm; IR (film): $\tilde{\nu}$ = 2980, 1736, 1560, 1395, 1202, 1168, 1121 cm^{-1}; HRMS (ESI): *m/z* calcd for $C_{13}H_{20}O_3N_3S$ [*M*-Cl]$^+$: 298.1220, found: 298.1215.

(±)-Ethyl 2-(2-(3-methyl-1-(phenylsulfonyl)pyrrolidine-3-carboxamido) thiazol-4-yl)acetate (29c)

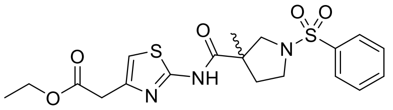

According to General Procedure E, racemic sulfonamide **29c** (345 mg, 0.79 mmol, 60%) was obtained from amine HCl salt **29b** (440 mg, 1.3 mmol) and benzenesulfonyl chloride (0.20 mL, 280 mg, 1.6 mmol), following column chromatography (25 g Sfär Silica D; 60 mL/min; 100% dichloromethane (2 CV), followed by a linear gradient (15 CV): 0%→50% ethyl acetate in dichloromethane).

Colourless oil; ^{1}H NMR (600 MHz, 300K, $CDCl_3$): δ = 9.11 (br s, 1H), 7.84 (d, *J* = 8.0 Hz, 2H), 7.59 – 7.54 (m, 1H), 7.53 – 7.48 (m, 2H), 6.79 (s, 1H), 4.19 (q, *J* = 7.0 Hz, 2H), 3.74 – 3.62 (m, 3H), 3.48 – 3.43 (m, 1H), 3.42 – 3.37 (m, 1H), 3.26 (d, *J* = 10.5 Hz, 1H), 2.36 – 2.31 (m, 1H), 1.83 – 1.77 (m, 1H), 1.33 (s, 3H), 1.28 (t, *J* = 7.0 Hz, 3H) ppm; ^{13}C NMR (151 MHz, 300K, $CDCl_3$): δ = 172.1, 170.5, 157.4, 143.7, 136.2, 133.1, 129.3, 127.7, 111.5, 61.3, 56.7, 49.2, 46.9, 37.2, 35.7, 22.6, 14.3 ppm; IR (film): $\tilde{\nu}$ = 2926, 1735, 1679, 1546, 1337, 1160, 1027 cm^{-1}; HRMS (ESI): *m/z* calcd for $C_{19}H_{24}O_5N_3S_2$ [*M*+H]$^+$: 438.1152, found: 438.1150.

(±)-Ethyl (*Z*)-2-(3-hydroxy-2-((3-methyl-1-(phenylsulfonyl)pyrrolidine-3-carbonyl)imino)-2,3-dihydrothiazol-4-yl)acetate (29d)

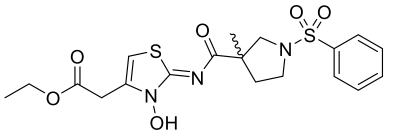

According to General Procedure B, racemic *N*-hydroxythiazole **29d** (245 mg, 0.54 mmol, 69%) was obtained from thiazole **29c** (345 mg, 0.79 mmol, 1.0 equiv.), following column chromatography (10 g Sfär Silica D; 35 mL/min; 100% dichloromethane (2 CV), followed by a linear gradient (20 CV): 0%→10% methanol in dichloromethane).

Yellow oil; ^{1}H NMR (600 MHz, 300K, $CDCl_3$): δ = 7.86 – 7.81 (m, 2H), 7.59 – 7.53 (m, 1H), 7.53 – 7.48 (m, 2H), 6.96 (s, 1H), 4.23 (q, *J* = 7.0 Hz, 2H), 3.84 (s, 2H), 3.72 (d, *J* = 10.5 Hz, 1H), 3.51 – 3.45 (m, 1H), 3.42 – 3.33 (m, 1H), 3.29 (d, *J* = 10.5 Hz, 1H), 2.40 – 2.34 (m, 1H), 1.90 – 1.84 (m, 1H), 1.36 (s, 3H), 1.30 (t, *J* = 7.0 Hz, 3H) ppm; ^{13}C NMR (151 MHz, 300K, $CDCl_3$): δ = 172.4, 168.5, 141.6, 137.2, 136.4, 133.0, 129.3, 127.7, 107.4, 61.8, 56.6, 49.5, 46.9, 35.7, 32.0, 22.5, 14.3 ppm; IR (film): $\tilde{\nu}$ = 2980, 2932, 1737, 1680, 1538, 1478, 1446, 1348, 1160, 1094, 1056, 1026 cm^{-1}; HRMS (ESI): *m/z* calcd for $C_{19}H_{24}O_6N_3S_2$ [*M*+H]$^+$: 454.1101, found: 454.1098.

(±)-(*Z*)-2-(3-Hydroxy-2-((3-methyl-1-(phenylsulfonyl)pyrrolidine-3-carbonyl) imino)-2,3-dihydrothiazol-4-yl)acetic acid (29)

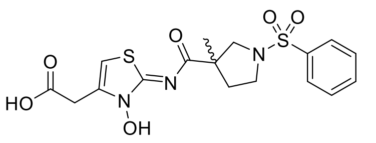

According to General Procedure C, racemic carboxylic acid **29** (32 mg, 0.08 mmol, 17%) was obtained from ethyl ester **29d** (196 mg, 0.44 mmol), following trituration with H_2O (3 × 5 mL; Milli-Q® Ultrapure grade) and MeOH (3 × 5 mL; HPLC grade).

White solid, m.p.: 158-161 °C; ^{1}H NMR (600 MHz, 300K, DMSO-d_6): δ = 7.76 (d, *J* = 7.0 Hz, 2H), 7.60 – 7.49 (m, 3H), 6.93 (s, 1H), 3.86 (d, *J* = 10.5 Hz, 1H), 3.76 – 3.66 (m, 2H), 3.30 – 3.24 (m, 1H), 3.20 – 3.15 (m, 1H), 3.10 (d, *J* = 10.5 Hz, 1H), 2.36 – 2.31 (m, 1H), 1.68 – 1.62 (m, 1H), 1.10 (s, 3H) ppm; ^{13}C NMR (151 MHz, 300K, DMSO-d_6): δ = 179.5 (br), 169.8, 155.7 (br), 136.0, 133.3, 132.7, 129.1, 127.2, 105.2, 56.8, 50.4, 46.9, 34.8, 32.4, 22.5 ppm; IR (film): $\tilde{\nu}$ = 2981, 1690, 1552, 1339, 1163, 1093 cm^{-1}; HRMS (ESI): *m/z* calcd for $C_{17}H_{20}O_6N_3S_2$ [*M*+H]$^+$: 426.0788, found: 426.0785.

(±)-*trans*-Methyl 1-benzyl-4-methylpyrrolidine-3-carboxylate (30b)

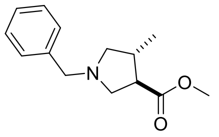

To a solution of methyl (*E*)-but-2-enoate **30a** (2.12 mL, 2.0 g, 20 mmol, 1.0 equiv.) and *N*-(methoxymethyl)-*N*-(trimethylsilylmethyl)benzylamine (6.14 mL, 5.7 g, 24 mmol, 1.2 equiv.) in dichloromethane (40 mL; HPLC grade) under an ambient atmosphere at 0 °C was added trifluoroacetic acid (0.15 mL, 228 mg, 2.0 mmol, 0.1 equiv.) dropwise. The reaction mixture was allowed to warm to ambient temperature and stirred for 3 h. The reaction mixture was diluted with ethyl acetate and washed with saturated aqueous $NaHCO_3$ solution and brine, dried over anhydrous Na_2SO_4, filtered, and evaporated. The crude residue was purified using column chromatography (100 g Sfär Silica D; 120 mL/min; 100% cyclohexane (2 CV), followed by a linear gradient (15 CV): 0%→50% diethyl ether in cyclohexane) to afford racemic pyrrolidine **30b** (3.14 g, 13 mmol, 67%).

Clear oil; ^{1}H NMR (600 MHz, 300K, $CDCl_3$): δ = 7.35 – 7.28 (m, 4H), 7.27 – 7.21 (m, 1H), 3.68 (s, 3H), 3.65 (d, *J* = 13.0 Hz, 1H), 3.58 (d, *J* = 13.0 Hz, 1H), 2.88 (dd, *J* = 9.5, 6.5 Hz, 1H), 2.85 – 2.77 (m, 2H), 2.61 – 2.57 (m, 1H), 2.55 – 2.50 (m, 1H), 2.23 (dd, *J* = 9.0, 6.5 Hz, 1H), 1.14 (d, *J* = 6.5 Hz, 3H) ppm; ^{13}C NMR (151 MHz, 300K, $CDCl_3$): δ = 175.2, 138.9, 128.9, 128.4, 127.1, 61.7, 60.2, 56.8, 51.9, 50.6, 36.9, 19.9

ppm; IR (film): $\tilde{\nu}$ = 2794, 1736, 1454, 1201 cm^{-1}; HRMS (ESI): *m/z* calcd for $C_{14}H_{19}O_2NNa$ [*M*+Na]$^+$: 256.1308, found: 256.1308.

The analytical data is consistent with the literature.[65]

(±)-*trans*-Methyl 4-methylpyrrolidine-3-carboxylate (30c)

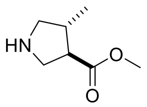

To a solution of *N*-benzyl protected pyrrolidine **30b** (3.14 g, 13 mmol, 1.0 equiv.) and ammonium formate (2.55 g, 40 mmol, 3.0 equiv.) in anhydrous methanol (30 mL) under an ambient atmosphere was added 20% $Pd(OH)_2$/C (113 mg, 0.67 mmol, 0.05 equiv.). The reaction mixture was stirred vigorously under reflux for 1.5 h. The reaction mixture was allowed to cool to ambient temperature and filtered through Celite. The Celite was washed with diethyl ether, and 2 M aqueous ammonia was added to the filtrate (168 mL, 25 equiv). The two layers were separated, and the aqueous layer was extracted twice with diethyl ether; the combined organic extracts were dried over Na_2SO_4, filtered, and evaporated to afford racemic pyrrolidine **30c** (718 mg, 5.0 mmol, 37%), which was used in the subsequent step without further purification.

Yellow oil; ^{1}H NMR (600 MHz, 300K, $CDCl_3$): δ = 3.66 (s, 3H), 3.22 – 3.07 (m, 3H), 2.47 – 2.37 (m, 2H), 2.34 – 2.26 (m, 1H), 1.08 (d, *J* = 6.5 Hz, 3H) ppm; ^{13}C NMR (151 MHz, 300K, $CDCl_3$): δ = 175.9, 55.8, 52.2, 51.9, 51.2, 40.0, 18.5 ppm; IR (film): $\tilde{\nu}$ = 2958, 1732, 1537, 1435, 1372, 1200, 1174 cm^{-1}; HRMS (ESI): *m/z* calcd for $C_7H_{14}O_2N$ [*M*+H]$^+$: 144.1019, found: 144.1026.

(±)-*trans*-Methyl 4-methyl-1-(phenylsulfonyl)pyrrolidine-3-carboxylate (30d)

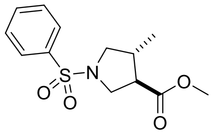

To a solution of amine **30c** (718 mg, 5.0 mmol, 1.0 equiv.) in anhydrous dichloromethane (25 mL) at 0 °C under an atmosphere of N_2 gas were sequentially added redistilled anhydrous *N*,*N*-diisopropylethylamine (2.62 mL, 1.9 g, 15 mmol, 3.0 equiv.) and benzenesulfonyl chloride (0.70 mL, 974 mg, 5.5 mmol, 1.1 equiv.) The reaction mixture was allowed to warm to room temperature and stirred for 14 h. The crude reaction mixture was diluted with ethyl acetate and washed with 1M aqueous HCl solution, saturated aqueous $NaHCO_3$ solution and brine, dried over anhydrous Na_2SO_4, filtered, and evaporated. The crude residue was purified using column chromatography (50 g Sfär Silica D; 120 mL/min; 100% cyclohexane (2 CV), followed by a linear gradient (15 CV): 0%→50% diethyl ether in cyclohexane) to afford racemic sulfonamide **30d** (1.16 g, 4.1 mmol, 82%).

Clear oil; ^{1}H NMR (600 MHz, 300K, $CDCl_3$): δ = 7.86 – 7.81 (m, 2H), 7.64 – 7.58 (m, 1H), 7.57 – 7.52 (m, 2H), 3.65 – 3.61 (m, 4H), 3.50 (dd, *J* = 10.0, 7.5 Hz, 1H), 3.43 (dd, *J* = 10.0, 8.5 Hz, 1H), 2.90 (dd, *J* = 10.0, 8.0 Hz, 1H), 2.52 (q, *J* = 8.5 Hz, 1H), 2.40 – 2.30 (m, 1H), 1.03 (d, *J* = 6.5 Hz, 3H) ppm; ^{13}C NMR (151 MHz, 300K, $CDCl_3$): δ = 172.4, 136.7, 133.0, 129.3, 127.7, 54.5, 52.3, 50.2, 50.1, 37.2, 17.2 ppm; IR (film): $\tilde{\nu}$ = 2957, 1735, 1446, 1344, 1165, 1092, 1023 cm^{-1}; HRMS (ESI): *m/z* calcd for $C_{13}H_{18}O_4NS$ [*M*+H]$^+$: 284.0951, found: 284.0961.

(±)-*trans*-4-Methyl-1-(phenylsulfonyl)pyrrolidine-3-carboxylic acid (30e)

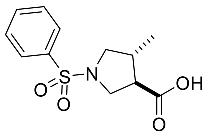

To a solution of methyl ester **30d** (591 mg, 2.1 mmol, 1.0 equiv.) in tetrahydrofuran (5.0 mL; HPLC grade) at 0 °C under an ambient atmosphere added 0.4 M aqueous lithium hydroxide solution (5.7 mL, 2.3 mmol, 1.1 equiv.). The reaction mixture was allowed to warm to ambient temperature and stirred for 1 h. The pH of the solution was adjusted to pH 6 – 7 by the dropwise addition of 1 N aqueous HCl solution. The tetrahydrofuran was removed under reduced pressure and the remaining aqueous solution basified

with aqueous lithium hydroxide solution (pH 10 – 11). The aqueous solution was washed thrice with dichloromethane (the organic extracts were discarded). The aqueous phase was then re-acidified (pH 4 – 5) by the dropwise addition of 1 N aqueous HCl solution. The aqueous solution was extracted thrice with ethyl acetate; the combined organic extracts were dried over Na_2SO_4, filtered, and evaporated to afford racemic carboxylic acid **30d** (407 mg, 1.5 mmol, 73%).

White solid; m.p.: 139-143 °C; ^{1}H NMR (600 MHz, 300K, DMSO-d_6): δ = 12.50 (br s, 1H), 7.84 – 7.79 (m, 2H), 7.72 (t, *J* = 7.5 Hz, 1H), 7.67 – 7.63 (m, 2H), 3.50 – 3.41 (m, 2H), 3.29 (dd, *J* = 10.0, 8.0 Hz, 1H), 2.74 (dd, *J* = 10.0, 8.5 Hz, 1H), 2.50 – 2.45 (m, 1H), 2.18 – 2.11 (m, 1H), 0.90 (d, *J* = 6.5 Hz, 3H) ppm; ^{13}C NMR (151 MHz, 300K, DMSO-d_6): δ = 173.2, 135.8, 133.2, 129.4, 127.3, 54.3, 49.7, 49.2, 36.6, 16.6 ppm; IR (film): $\tilde{\nu}$ = 2967, 1711, 1337, 1311, 1163, 1092 cm^{-1}; HRMS (ESI): *m/z* calcd for $C_{12}H_{16}O_4NS$ [*M*+H]$^+$: 270.0795, found: 270.0792.

(±)-*trans*-Ethyl 2-(2-(4-methyl-1-(phenylsulfonyl)pyrrolidine-3-carboxamido)thiazol-4-yl)acetate (30f)

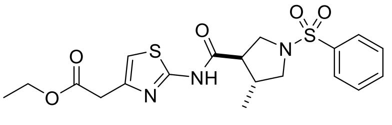

According to General Procedure A*, racemic amide **30f** (88 mg, 0.20 mmol, 45%) was obtained from ethyl 2-(2-aminothiazol-4-yl)acetate **1** (99 mg, 0.53 mmol, 1.2 equiv.) and carboxylic acid **30e** (119 mg, 0.44 mmol, 1.0 equiv.), following column chromatography (10 g Sfär Silica D; 35 mL/min; 100% dichloromethane (2 CV), followed by a linear gradient (15 CV): 0%→30% ethyl acetate in dichloromethane).

*Note: An excess of ethyl 2-(2-aminothiazol-4-yl)acetate **1** (1.2 equiv.) was used relative to carboxylic acid **30e**.

Clear oil; ^{1}H NMR (600 MHz, 300K, DMSO-d_6): δ = 12.31 (s, 1H), 7.82 (d, *J* = 7.5 Hz, 2H), 7.73 (t, *J* = 7.5 Hz, 1H), 7.67 – 7.63 (m, 2H), 6.98 (s, 1H), 4.07 (q, *J* = 7.0 Hz, 2H), 3.67 (s, 2H), 3.61 (dd, *J* = 10.0, 8.0 Hz, 1H), 3.48 (dd, *J* = 10.0, 7.5 Hz, 1H), 3.28 – 3.23 (m, 1H), 2.85 – 2.77 (m, 1H), 2.72 (q, *J* = 7.0 Hz, 1H), 2.26 – 2.21 (m, 1H), 1.17 (t, *J* = 7.0 Hz, 3H), 0.87 (d, *J* = 7.0 Hz, 3H) ppm; ^{13}C NMR (151 MHz, 300K, DMSO-d_6): δ = 170.0, 169.6, 157.3, 143.6, 135.9, 133.2, 129.4, 127.3, 110.6, 60.3, 54.1, 50.3, 49.9, 37.1, 36.6, 16.1, 14.1 ppm; IR (film): $\tilde{\nu}$ = 3281, 2925, 1735, 1687, 1547, 1340, 1163 cm^{-1}; HRMS (ESI): *m/z* calcd for $C_{19}H_{24}O_5N_3S_2$ [*M*+H]$^+$: 438.1152, found: 438.1159.

(±)-*trans*-Ethyl 2-((*Z*)-3-hydroxy-2-((4-methyl-1-(phenylsulfonyl)pyrrolidine-3-carbonyl)imino)-2,3-dihydrothiazol-4-yl)acetate (30g)

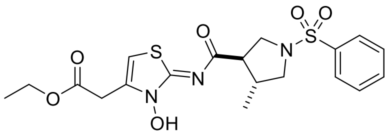

According to General Procedure B, racemic *N*-hydroxythiazole **30g** (54 mg, 0.12 mmol, 69%) was obtained from thiazole **30f** (75 mg, 0.17 mmol, 1.0 equiv.), following column chromatography (10 g Sfär Silica D; 35 mL/min; 100% cyclohexane (2 CV), followed by a linear gradient (15 CV): 0%→100% acetone in cyclohexane).

Note: The *N*-hydroxythiazole *C*2 signal was not visible in the ^{13}C spectrum due to low sample concentration. The corresponding signal in other *N*-hydroxythiazole compounds is very broad in DMSO-d_6 and is observed between 140 and 160 ppm.

Yellow oil; ^{1}H NMR (600 MHz, 300K, DMSO-d_6): δ = 7.82 (d, *J* = 7.5 Hz, 2H), 7.72 (t, *J* = 7.5 Hz, 1H), 7.66 – 7.62 (m, 2H), 7.13 (s, 1H), 4.08 (q, *J* = 7.0 Hz, 2H), 3.73 (s, 2H), 3.59 – 3.54 (m, 1H), 3.46 (dd, *J* = 9.5, 7.5 Hz, 1H), 3.31 – 3.25 (m, 1H), 2.98 – 2.92 (m, 1H), 2.82 – 2.75 (m, 1H), 2.32 – 2.17 (m, 1H), 1.18 (t, *J* = 7.0 Hz, 3H), 0.87 (d, *J* = 6.0 Hz, 3H) ppm; ^{13}C NMR (151 MHz, 300K, DMSO-d_6): δ = 172.1 (br), 168.6,

135.9 (2C), 133.1, 129.4, 127.3, 107.2 (br), 60.6, 54.2, 50.6, 50.3, 37.1, 31.9, 16.3, 14.1 ppm; IR (film): $\tilde{\nu}$ = 2920, 1738, 1547, 1340, 1164 cm^{-1}; HRMS (ESI): *m/z* calcd for $C_{19}H_{24}O_6N_3S_2$ [*M*+H]$^+$: 454.1101, found: 454.1100.

(±)-*trans*-2-((*Z*)-3-Hydroxy-2-((4-methyl-1-(phenylsulfonyl)pyrrolidine-3-carbonyl)imino)-2,3-dihydrothiazol-4-yl)acetic acid (30)

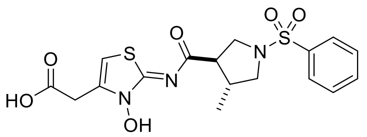

To a solution of methyl ester **30g** (46 mg, 0.10 mmol, 1.0 equiv.) in tetrahydrofuran (1.0 mL; HPLC grade) at 0 °C under an ambient atmosphere was added 0.4 M aqueous lithium hydroxide solution (0.28 mL, 0.11 mmol, 1.1 equiv.). The reaction mixture was allowed to warm to ambient temperature and stirred for 1 h. The pH of the solution was adjusted to pH 6 – 7 by the dropwise addition of 1 N aqueous HCl solution. The tetrahydrofuran was removed under reduced pressure and the remaining aqueous solution basified with aqueous lithium hydroxide solution (pH 10 – 11). The aqueous solution was washed thrice with dichloromethane (the organic extracts were discarded). The aqueous phase was then neturalised (pH 6 – 7) with the dropwise addition of 1 N aqueous HCl solution. The solvent was evaporated under reduced pressure and crude residue was triturated with H_2O (3 × 5 mL; Milli-Q® Ultrapure grade) and MeOH (3 × 5 mL; HPLC grade) to afford racemic carboxylic acid **30** (8 mg, 0.02 mmol, 19%).

White solid; m.p.: 180-182 °C; ^{1}H NMR (600 MHz, 300K, DMSO-d_6): δ = 7.87 – 7.78 (m, 2H), 7.76 – 7.69 (m, 1H), 7.68 – 7.62 (m, 2H), 7.20 (s, 1H), 3.71 (s, 2H), 3.57 (dd, *J* = 10.0, 8.0 Hz, 1H), 3.50 – 3.44 (m, 1H), 3.29 (dd, *J* = 10.0, 8.0 Hz, 1H), 3.02 (q, *J* = 7.0 Hz, 1H), 2.81 (dd, *J* = 10.0, 8.0 Hz, 1H), 2.30 – 2.22 (m, 1H), 0.87 (d, *J* = 7.0 Hz, 3H) ppm; ^{13}C NMR (151 MHz, 300K, DMSO-d_6): δ = 171.8 (br), 169.7, 144.3 (br), 136.1, 135.9, 133.2, 129.5, 127.4, 107.7, 54.2, 50.5, 50.0, 37.1, 32.6, 16.3 ppm; IR (film): $\tilde{\nu}$ = 3117, 1729, 1695, 1551, 1337, 1152, 1020 cm^{-1}; HRMS (ESI): *m/z* calcd for $C_{17}H_{20}O_6N_3S_2$ [*M*+H]$^+$: 426.0788, found: 426.0796.

(±)-*tert*-Butyl 3-((4-(2-ethoxy-2-oxoethyl)thiazol-2-yl)carbamoyl)pyrrolidine-1-carboxylate (31)

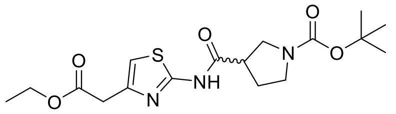

According to General Procedure A, racemic amide **31** (1.54 g, 4.0 mmol, 81%) was obtained from ethyl 2-(2-aminothiazol-4-yl)acetate **1** (931 mg, 5.0 mmol) and 1-(*tert*-butoxycarbonyl)-3-pyrrolidinecarboxylic acid (1.29 g, 6.0 mmol), following column chromatography (50 g Sfär Silica D; 120 mL/min; 100% cyclohexane (2 CV), followed by a linear gradient (15 CV): 0%→30% acetone in cyclohexane).

Note: Several signals in the ^{13}C NMR spectrum appear as doublets, due to restricted rotation of the *N*-Boc group.

Yellow oil; ^{1}H NMR (600 MHz, 300K, DMSO-d_6): δ = 12.31 – 12.27 (m, 1H), 6.98 (s, 1H), 4.08 (q, *J* = 7.0 Hz, 2H), 3.68 (s, 2H), 3.56 – 3.47 (m, 1H), 3.40 – 3.33 (m, 2H), 3.27 – 3.20 (m, 2H), 2.14 – 2.09 (m, 1H), 2.08 – 1.94 (m, 1H), 1.40 (s, 9H), 1.18 (t, *J* = 7.0 Hz, 3H) ppm; ^{13}C NMR (151 MHz, 300K, DMSO-d_6): δ = 171.1 (d), 170.0, 157.5, 153.3, 143.6, 110.5, 78.4, 60.3, 48.0 (d), 45.2 (d), 42.7 (d), 36.6, 28.9, 28.1, 14.1 ppm; IR (film): $\tilde{\nu}$ = 2980, 1734, 1685, 1548, 1416, 1165 cm^{-1}; HRMS (ESI): *m/z* calcd for $C_{17}H_{26}O_5N_3S$ [*M*+H]$^+$: 384.1588, found: 384.1582.

(±)-Ethyl 2-(2-(pyrrolidine-3-carboxamido)thiazol-4-yl)acetate hydrochloride (32)

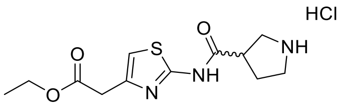

According to General Procedure D, racemic amine HCl salt **32** (701 mg, 2.2 mmol, 54%) was obtained from *N*-Boc protected amine **31** (1.54 g, 4.0 mmol). **32** was used in the subsequent reaction without further purification.

White solid; m.p.: > 200 °C (decomposition); ^{1}H NMR (600 MHz, D_2O): δ = 7.13 (s, 1H), 4.20 (q, *J* = 7.0 Hz, 2H), 3.85 (s, 2H), 3.75 – 3.60 (m, 2H), 3.63 – 3.56 (m, 2H), 3.49 – 3.41 (m, 2H), 2.52 – 2.43 (m, 1H), 2.33 – 2.25 (m, 1H), 1.25 (t, *J* = 7.0 Hz, 3H) ppm; ^{13}C NMR (151 MHz, D_2O): δ = 172.3, 172.1, 159.6, 139.6, 112.9, 62.6, 47.0, 45.4, 42.5, 35.0, 28.6, 13.2 ppm; IR (film): $\tilde{\nu}$ = 2853, 2633, 1730, 1699, 1566, 1256, 1120 cm^{-1}; HRMS (ESI): *m/z* calcd for $C_{12}H_{18}O_3N_3S$ [*M*-Cl]$^+$: 284.1063, found: 284.1060.

(±)-Ethyl 2-(2-(1-((2-chlorophenyl)sulfonyl)pyrrolidine-3-carboxamido)thiazol-4-yl)acetate (33a)

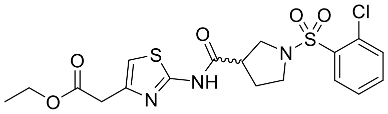

According to General Procedure E, racemic sulfonamide **33a** (278 mg, 0.61 mmol, 78%) was obtained from amine HCl salt **32** (250 mg, 0.78 mmol, 1.0 equiv.) and 2-chlorobenzenesulfonyl chloride (0.13 mL, 198 mg, 0.94 mmol, 1.2 equiv.), following column chromatography (25 g Sfär Silica D; 60 mL/min; 100% cyclohexane (2 CV), followed by a linear gradient (12 CV): 0%→60% ethyl acetate in cyclohexane).

Yellow oil; ^{1}H NMR (600 MHz, 300K, $CDCl_3$): δ = 8.08 (d, *J* = 8.0 Hz, 1H), 7.56 – 7.46 (m, 2H), 7.43 – 7.37 (m, 1H), 6.80 (s, 1H), 4.18 (q, *J* = 7.0 Hz, 2H), 3.83 (dd, *J* = 10.0, 8.0 Hz, 1H), 3.69 (s, 2H), 3.64 – 3.58 (m, 2H), 3.57 – 3.51 (m, 1H), 3.21 – 3.16 (m, 1H), 2.33 – 2.21 (m, 2H), 1.27 (t, *J* = 7.0 Hz, 3H) ppm; ^{13}C NMR (151 MHz, 300K, $CDCl_3$): δ = 170.5, 169.5, 157.5, 143.2, 136.6, 133.9, 132.5, 132.3, 132.1, 127.2, 111.5, 61.5, 50.1, 47.5, 44.6, 37.1, 29.5, 14.3 ppm; IR (film): $\tilde{\nu}$ = 3205, 2981, 1673, 1636, 1548, 1327, 1151, 1085 cm^{-1}; HRMS (ESI): *m/z* calcd for $C_{18}H_{21}ClN_3O_5S_2$ [*M*+H]$^+$: 458.0606, found: 458.0607.

(±)-Ethyl (*Z*)-2-(2-((1-((2-chlorophenyl)sulfonyl)pyrrolidine-3-carbonyl)imino)-3-hydroxy-2,3-dihydrothiazol-4-yl)acetate (33b)

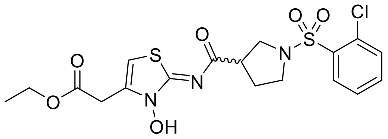

According to General Procedure B, racemic *N*-hydroxythiazole **33b** (88 mg, 0.19 mmol, 30%) was obtained from thiazole **33a** (279 mg, 0.63 mmol), following column chromatography (10 g Sfär Silica D; 35 mL/min; 100% dichloromethane (2 CV), followed by a linear gradient (20 CV): 0%→5% methanol in dichloromethane).

White solid, m.p.: 83-85 °C; ^{1}H NMR (600 MHz, 300K, $CDCl_3$): δ = 8.06 (dd, *J* = 8.0, 1.5 Hz, 1H), 7.54 – 7.45 (m, 2H), 7.42 – 7.35 (m, 1H), 6.99 (s, 1H), 4.21 (q, *J* = 7.0 Hz, 2H), 3.84 – 3.75 (m, 3H), 3.65 – 3.55 (m, 4H), 2.28 – 2.20 (m, 2H), 1.28 (t, *J* = 7.0 Hz, 3H) ppm; ^{13}C NMR (151 MHz, 300K, $CDCl_3$): δ = 171.0, 168.4, 142.8, 136.9, 136.8, 133.8, 132.5, 132.3, 132.1, 127.2, 107.9, 61.9, 50.2, 47.6, 44.0, 32.1, 29.7, 14.3 ppm; IR (film): $\tilde{\nu}$ = 2980, 1735, 1697, 1548, 1340, 1154, 1024 cm^{-1}; HRMS (ESI): *m/z* calcd for $C_{18}H_{21}ClN_3O_6S_2$ [*M*+H]$^+$: 474.0555, found: 474.0578.

(±)-(*Z*)-2-(2-((1-((2-Chlorophenyl)sulfonyl)pyrrolidine-3-carbonyl)imino)-3-hydroxy-2,3-dihydrothiazol-4-yl)acetic acid (33)

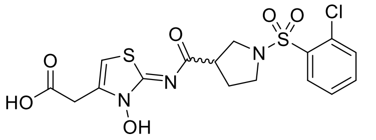

According to General Procedure C, racemic carboxylic acid **33** (28 mg, 0.063 mmol, 57%) was obtained from ethyl ester **33b** (50 mg, 0.11 mmol), following reverse-phase column chromatography (12 g Sfär C18 Duo; 12 mL/min; 100% water (+ 0.1% (*v/v*) formic acid) (4 CV), followed by a linear gradient (20 CV): 0%→100% acetonitrile (+ 0.1% (*v/v*) formic acid) in water (+ 0.1% (*v/v*) formic acid)).

White solid, m.p.: 147-150 °C; ^{1}H NMR (600 MHz, 300K, DMSO-d_6): δ = 7.98 (dd, *J* = 8.0, 1.5 Hz, 1H), 7.72 – 7.63 (m, 2H), 7.55 (ddd, *J* = 8.0, 7.0, 1.5 Hz, 1H), 7.22 (s, 1H), 3.72 (s, 2H), 3.66 – 3.60 (m, 1H), 3.56 – 3.40 (m, 3H), 3.38 – 3.34 (m, 1H), 2.20 – 2.14 (m, 1H), 2.11 – 2.05 (m, 1H) ppm; ^{13}C NMR (151 MHz, 300K, DMSO-d_6): δ = 172.5 (br), 169.7, 144.6 (br), 136.0, 135.9, 134.3, 132.3, 131.3, 130.9, 127.8, 107.5, 50.0, 47.4, 43.5, 32.8, 29.0 ppm; IR (film): $\tilde{\nu}$ = 3121, 1702, 1552, 1348, 1160, 1042 cm^{-1}; HRMS (ESI): *m/z* calcd for $C_{16}H_{17}ClN_3O_6S_2$ [*M*+H]$^+$: 446.0242, found: 446.0247.

(±)-Ethyl 2-(2-(1-((3-chlorophenyl)sulfonyl)pyrrolidine-3-carboxamido)thiazol-4-yl)acetate (34a)

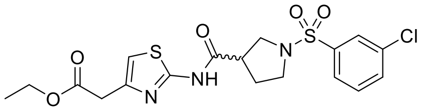

According to General Procedure E, racemic sulfonamide **34a** (273 mg, 0.60 mmol, 78%) was obtained from amine HCl salt **32** (250 mg, 0.78 mmol) and 3-chlorobenzenesulfonyl chloride (0.13 mL, 198 mg, 0.94 mmol), following column chromatography (25 g Sfär Silica D; 60 mL/min; 100% cyclohexane (2 CV), followed by a linear gradient (12 CV): 0%→60% ethyl acetate in cyclohexane).

Yellow oil; ^{1}H NMR (600 MHz, 300K, $CDCl_3$): δ = 7.83 (s, 1H), 7.72 (d, *J* = 8.0 Hz, 1H), 7.56 (d, *J* = 8.0 Hz, 1H), 7.49 – 7.45 (m, 1H), 6.79 (s, 1H), 4.18 (q, *J* = 7.0 Hz, 2H), 3.73 – 3.63 (m, 3H), 3.47 – 3.42 (m, 2H), 3.40 – 3.35 (m, 1H), 3.12 – 3.06 (m, 1H), 2.25 – 2.07 (m, 2H), 1.26 (t, *J* = 7.0 Hz, 3H) ppm; ^{13}C NMR (151 MHz, 300K, $CDCl_3$): δ = 170.7, 169.5, 157.4, 143.5, 138.3, 135.6, 133.3, 130.6, 127.7, 125.8, 111.5, 61.5, 50.5, 47.7, 44.0, 37.1, 29.0, 14.3 ppm; IR (film): $\tilde{\nu}$ = 3272, 2981, 1734, 1687, 1546, 1338, 1161 cm^{-1}; HRMS (ESI): *m/z* calcd for $C_{18}H_{21}ClN_3O_5S_2$ [*M*+H]$^+$: 458.0606, found: 458.0601.

(±)-Ethyl (*Z*)-2-(2-((1-((3-chlorophenyl)sulfonyl)pyrrolidine-3-carbonyl)imino)-3-hydroxy-2,3-dihydrothiazol-4-yl)acetate (34b)

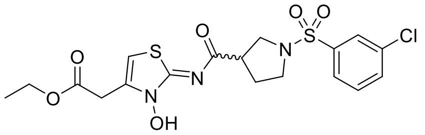

According to General Procedure B, racemic *N*-hydroxythiazole **34b** (79 mg, 0.17 mmol, 25%) was obtained from thiazole **34a** (300 mg, 0.68 mmol), following column chromatography (10 g Sfär Silica D; 35 mL/min; 100% dichloromethane (2 CV), followed by a linear gradient (20 CV): 0%→5% methanol in dichloromethane).

White solid, m.p.: 84-87 °C; ^{1}H NMR (600 MHz, 300K, DMSO-d_6): δ = 7.82 – 7.74 (m, 3H), 7.67 – 7.63 (m, 1H), 7.20 (s, 1H), 4.09 (q, *J* = 7.0 Hz, 2H), 3.75 (s, 2H), 3.58 – 3.49 (m, 1H), 3.40 – 3.31 (m, 2H), 3.31 – 3.17 (m, 2H), 2.11 – 1.98 (m, 1H), 1.97 – 1.91 (m, 1H), 1.18 (t, *J* = 7.0 Hz, 3H) ppm; ^{13}C NMR (151 MHz, 300K, DMSO-d_6): δ = 172.3 (br), 168.5, 143.6 (br), 137.9, 136.0, 134.1, 133.1, 131.4, 126.8, 126.1, 107.6, 60.6, 50.5, 47.6, 43.1, 31.8, 28.6, 14.0 ppm; IR (film): $\tilde{\nu}$ = 2980, 1732, 1692, 1548, 1342, 1156, 1025 cm^{-1}; HRMS (ESI): *m/z* calcd for $C_{18}H_{21}ClN_3O_6S_2$ [*M*+H]$^+$: 474.0555, found: 474.0554.

(±)-(*Z*)-2-(2-((1-((3-Chlorophenyl)sulfonyl)pyrrolidine-3-carbonyl)imino)-3-hydroxy-2,3-dihydrothiazol-4-yl)acetic acid (34)

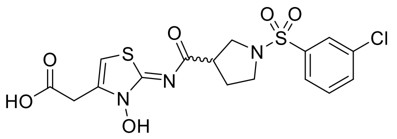

According to General Procedure C, racemic carboxylic acid **34** (26 mg, 0.06 mmol, 53%) was obtained from ethyl ester **34b** (50 mg, 0.11 mmol), following trituration with H_2O (3 × 5 mL; Milli-Q® Ultrapure grade) and MeOH (3 × 5 mL; HPLC grade).

White solid, m.p.: 178-182 °C; ^{1}H NMR (600 MHz, 300K, DMSO-*d*$_6$): δ = 7.82 – 7.74 (m, 3H), 7.66 – 7.63 (m, 1H), 7.20 (s, 1H), 3.71 (s, 2H), 3.58 – 3.50 (m, 1H), 3.43 – 3.32 (m, 2H), 3.33 – 3.19 (m, 2H), 2.08 – 2.03 (m, 1H), 1.99 – 1.92 (m, 1H) ppm; ^{13}C NMR (151 MHz, 300K, DMSO-*d*$_6$): δ = 172.4 (br), 169.7, 144.4 (br), 137.9, 135.9, 134.1, 133.1, 131.4, 126.8, 126.1, 107.5, 50.4, 47.6, 43.1, 32.7, 28.6 ppm; IR (film): $\tilde{\nu}$ = 2981, 1733, 1702, 1551, 1343, 1160, 1025 cm^{-1}; HRMS (ESI): *m/z* calcd for $C_{16}H_{17}ClN_3O_6S_2$ [*M*+H]$^+$: 446.0242, found: 446.0246.

(±)-Ethyl 2-(2-(1-((4-chlorophenyl)sulfonyl)pyrrolidine-3-carboxamido)thiazol-4-yl)acetate (35a)

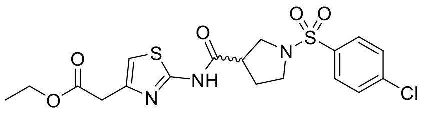

According to General Procedure E, racemic sulfonamide **35a** (293 mg, 0.64 mmol, 82%) was obtained from amine HCl salt **32** (250 mg, 0.78 mmol) and 4-chlorobenzenesulfonyl chloride (0.13 mL, 198 mg, 0.94 mmol), following column chromatography (25 g Sfär Silica D; 60 mL/min; 100% cyclohexane (2 CV), followed by a linear gradient (12 CV): 0%→60% ethyl acetate in cyclohexane).

Yellow oil; ^{1}H NMR (600 MHz, 300K, $CDCl_3$): δ = 7.78 (d, *J* = 8.5 Hz, 2H), 7.48 (d, *J* = 8.5 Hz, 2H), 6.79 (s, 1H), 4.18 (q, *J* = 7.0 Hz, 2H), 3.73 – 3.62 (m, 3H), 3.44 – 3.39 (m, 2H), 3.36 – 3.31 (m, 1H), 3.12 – 3.05 (m, 1H), 2.17 – 2.10 (m, 2H), 1.26 (t, *J* = 7.0 Hz, 3H) ppm; ^{13}C NMR (151 MHz, 300K, $CDCl_3$): δ = 170.8, 169.6, 157.4, 143.5, 139.8, 134.9, 129.7, 129.1, 111.5, 61.5, 50.6, 47.6, 44.0, 37.2, 28.9, 14.3 ppm; IR (film): $\tilde{\nu}$ = 3268, 1734, 1688, 1545, 1488, 1343, 1243, 1154, 1028 cm^{-1}; HRMS (ESI): *m/z* calcd for $C_{18}H_{21}ClN_3O_5S_2$ [*M*+H]$^+$: 458.0606, found: 458.0613.

(±)-Ethyl (*Z*)-2-(2-((1-((4-chlorophenyl)sulfonyl)pyrrolidine-3-carbonyl)imino)-3-hydroxy-2,3-dihydrothiazol-4-yl)acetate (35b)

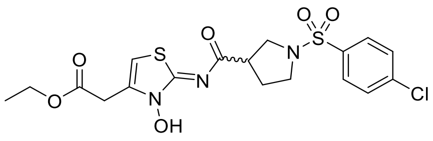

According to General Procedure B, racemic *N*-hydroxythiazole **35b** (84 mg, 0.18 mmol, 33%) was obtained from thiazole **35a** (245 mg, 0.55 mmol), following column chromatography (10 g Sfär Silica D; 35 mL/min; 100% dichloromethane (2 CV), followed by a linear gradient (20 CV): 0%→5% methanol in dichloromethane).

White solid, m.p.: 83-85 °C; ^{1}H NMR (600 MHz, 300K, $CDCl_3$): δ = 7.76 (d, *J* = 8.5 Hz, 2H), 7.49 (d, *J* = 8.5 Hz, 2H), 7.97 (s, 1H), 4.20 (q, *J* = 7.0 Hz, 2H), 3.82 – 3.73 (m, 2H), 3.68 – 3.63 (m, 1H), 3.60 – 3.51 (m, 1H), 3.49 – 3.45 (m, 1H), 3.39 – 3.35 (m, 1H), 3.33 – 3.27 (m, 1H), 2.20 – 2.05 (m, 2H), 1.29 (t, *J* = 7.0 Hz, 3H) ppm; ^{13}C NMR (151 MHz, 300K, $CDCl_3$): δ = 171.1, 168.4, 142.9, 139.6, 136.9, 135.1, 129.6, 129.2, 108.0, 61.9, 50.7, 47.6, 43.5, 32.1, 29.1, 14.3 ppm; IR (film): $\tilde{\nu}$ = 2981, 1730, 1692, 1549, 1342, 1156, 1026 cm^{-1}; HRMS (ESI): *m/z* calcd for $C_{18}H_{21}ClN_3O_6S_2$ [*M*+H]$^+$: 474.0555, found: 474.0562.

(±)-(*Z*)-2-(2-((1-((4-Chlorophenyl)sulfonyl)pyrrolidine-3-carbonyl)imino)-3-hydroxy-2,3-dihydrothiazol-4-yl)acetic acid (35)

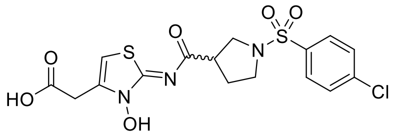

According to General Procedure C, racemic carboxylic acid **35** (30 mg, 0.07 mmol, 59%) was obtained from ethyl ester **35b** (50 mg, 0.11 mmol), following trituration with H_2O (3 × 5 mL; Milli-Q® Ultrapure grade) and MeOH (3 × 5 mL; HPLC grade).

White solid, m.p.: 195-197 °C; ^{1}H NMR (600 MHz, 300K, DMSO-d_6): δ = 7.81 (d, *J* = 8.5 Hz, 2H), 7.67 (d, *J* = 8.5 Hz, 2H), 7.19 (s, 1H), 3.71 (s, 2H), 3.56 – 3.48 (m, 1H), 3.37 - 3.33 (m, 2H), 3.29 – 3.16 (m, 2H), 2.06 – 2.00 (m, 1H), 1.97 – 1.90 (m, 1H) ppm; ^{13}C NMR (151 MHz, 300K, DMSO-d_6): δ = 172.8 (br), 170.2, 144.8 (br), 138.6, 136.4, 135.2, 130.0, 129.8, 108.1, 51.0, 48.1, 43.5, 33.1, 29.0 ppm; IR (film): $\tilde{\nu}$ = 2980, 1736, 1698, 1548, 1340, 1152, 1024 cm^{-1}; HRMS (ESI): *m/z* calcd for $C_{16}H_{17}ClN_3O_6S_2$ [*M*+H]$^+$: 446.0242, found: 446.0244.

(±)-Ethyl 2-(2-(1-((4-methoxyphenyl)sulfonyl)pyrrolidine-3-carboxamido)thiazol-4-yl)acetate (36a)

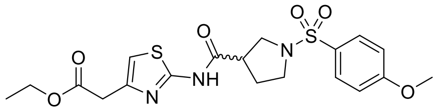

According to General Procedure E, racemic sulfonamide **36a** (326 mg, 0.72 mmol, 92%) was obtained from amine HCl salt **32** (250 mg, 0.78 mmol) and 4-methoxybenzenesulfonyl chloride (194 mg, 0.94 mmol), following column chromatography (25 g Sfär Silica D; 60 mL/min; 100% cyclohexane (2 CV), followed by a linear gradient (12 CV): 0%→60% ethyl acetate in cyclohexane).

Yellow oil; ^{1}H NMR (600 MHz, 300K, $CDCl_3$): δ = 7.76 (d, *J* = 8.0 Hz, 2H), 6.96 (d, *J* = 8.0 Hz, 2H), 6.77 (s, 1H), 4.16 (q, *J* = 7.0 Hz, 2H), 3.85 (s, 3H), 3.68 – 3.64 (m, 3H), 3.42 – 3.25 (m, 3H), 3.12 – 3.05 (m, 1H), 2.13 – 2.07 (m, 2H), 1.25 (t, *J* = 7.0 Hz, 3H) ppm; ^{13}C NMR (151 MHz, 300K, $CDCl_3$): δ = 170.9, 169.9, 163.3, 157.5, 143.5, 129.9, 127.7, 114.5, 111.3, 61.4, 55.7, 50.6, 47.6, 43.9, 37.2, 28.8, 14.3 ppm; IR (film): $\tilde{\nu}$ = 2972, 1734, 1687, 1548, 1262, 1157, 1026 cm^{-1}; HRMS (ESI): *m/z* calcd for $C_{19}H_{24}N_3O_6S_2$ [*M*+H]$^+$: 454.1101, found: 454.1100.

(±)-Ethyl (*Z*)-2-(3-hydroxy-2-((1-((4-methoxyphenyl)sulfonyl)pyrrolidine-3-carbonyl)imino)-2,3-dihydrothiazol-4-yl)acetate (36b)

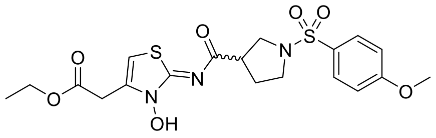

According to General Procedure B, racemic *N*-hydroxythiazole **36b** (133 mg, 0.28 mmol, 44%) was obtained from thiazole **36a** (280 mg, 0.64 mmol), following column chromatography (10 g Sfär Silica D; 35 mL/min; 100% dichloromethane (2 CV), followed by a linear gradient (20 CV): 0%→5% methanol in dichloromethane).

White solid, m.p.: 90-92 °C; ^{1}H NMR (600 MHz, 300K, $CDCl_3$): δ = 7.75 (d, *J* = 8.0 Hz, 2H), 7.00 - 6.95 (m, 3H), 4.21 (q, *J* = 7.0 Hz, 2H), 3.86 (s, 3H), 3.79 (s, 2H), 3.66 – 3.63 (m, 1H), 3.59 – 3.52 (m, 1H), 3.50 – 3.39 (m, 1H), 3.30 – 3.24 (m, 2H), 2.20 – 2.01 (m, 2H), 1.29 (t, *J* = 7.0 Hz, 3H) ppm; ^{13}C NMR (151 MHz, 300K, $CDCl_3$): δ = 171.2, 168.5, 163.3, 142.8, 136.9, 129.9, 128.0, 114.4, 107.9, 61.9, 55.8, 50.7, 47.6, 43.5, 32.1, 28.9, 14.3 ppm; IR (film): $\tilde{\nu}$ = 3208, 1732, 1693, 1548, 1338, 1258, 1153, 1025 cm^{-1}; HRMS (ESI): *m/z* calcd for $C_{19}H_{24}N_3O_7S_2$ [*M*+H]$^+$: 470.1050, found: 470.1060.

(±)-(*Z*)-2-(3-Hydroxy-2-((1-((4-methoxyphenyl)sulfonyl)pyrrolidine-3-carbonyl)imino)-2,3-dihydrothiazol-4-yl)acetic acid (36)

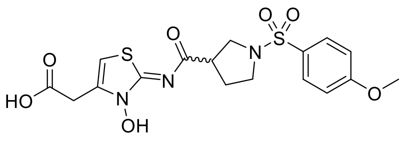

According to General Procedure C, racemic carboxylic acid **36** (54 mg, 0.12 mmol, 82%) was obtained from ethyl ester **36b** (70 mg, 0.15 mmol), following trituration with H_2O (3 × 5 mL; Milli-Q® Ultrapure grade) and MeOH (3 × 5 mL; HPLC grade).

White solid, m.p.: 180-183 °C; ^{1}H NMR (600 MHz, 300K, DMSO-d_6): δ = 7.73 (d, *J* = 8.0 Hz, 2H), 7.21 (s, 1H), 7.12 (d, *J* = 8.0 Hz, 2H), 3.84 (s, 3H), 3.72 (s, 2H), 3.50 – 3.46 (m, 1H), 3.38 – 3.32 (m, 1H), 3.30 – 3.23 (m, 1H), 3.23 – 3.12 (m, 2H), 2.04 – 1.98 (m, 1H), 1.93 – 1.87 (m, 1H) ppm; ^{13}C NMR (151 MHz, 300K, DMSO-d_6): δ = 172.3 (br), 169.7, 162.7, 144.1 (br), 136.1, 129.7, 127.2, 114.5, 107.7, 55.7, 50.5, 47.6, 42.9, 32.6, 28.4 ppm; IR (film): $\tilde{\nu}$ = 3127, 1740, 1698, 1550, 1337, 1266, 1148, 1025 cm^{-1}; HRMS (ESI): *m/z* calcd for $C_{17}H_{20}N_3O_7S_2$ [*M*+H]$^+$: 442.0737, found: 442.0744.

(±)-Ethyl 2-(2-(1-((4-(trifluoromethyl)phenyl)sulfonyl)pyrrolidine-3-carboxamido)thiazol-4-yl)acetate (37a)

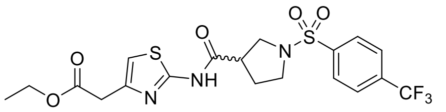

According to General Procedure E, racemic sulfonamide **37a** (300 mg, 0.61 mmol, 78%) was obtained from amine HCl salt **32** (250 mg, 0.78 mmol) and 4-(trifluoromethyl)benzenesulfonyl chloride (230 mg, 0.94 mmol), following column chromatography (25 g Sfär Silica D; 60 mL/min; 100% cyclohexane (2 CV), followed by a linear gradient (12 CV): 0%→60% ethyl acetate in cyclohexane).

Yellow oil; ^{1}H NMR (600 MHz, 300K, $CDCl_3$): δ = 7.97 (d, *J* = 8.0 Hz, 2H), 7.79 (d, *J* = 8.0 Hz, 2H), 6.77 (s, 1H), 4.17 (q, *J* = 7.0 Hz, 2H), 3.77 – 3.64 (m, 3H), 3.51 – 3.42 (m, 2H), 3.38 – 3.33 (m, 1H), 3.12 – 3.07 (m, 1H), 2.18 – 2.10 (m, 2H), 1.26 (t, *J* = 7.0 Hz, 3H) ppm; ^{13}C NMR (151 MHz, 300K, $CDCl_3$): δ = 170.9, 169.5, 157.3, 143.6, 140.2, 134.8 (q, *J* = 33.0 Hz), 128.2, 126.5 (q, *J* = 3.5 Hz), 123.4 (q, *J* = 273.0 Hz), 111.5, 61.5, 50.5, 47.6, 43.9, 37.2, 29.0, 14.3 ppm; ^{19}F NMR (565 MHz, 300K, $CDCl_3$): δ = –63.1 ppm; IR (film): $\tilde{\nu}$ = 2981, 1734, 1689, 1546, 1323, 1165, 1063 cm^{-1}; HRMS (ESI): *m/z* calcd for $C_{19}H_{21}F_3N_3O_5S_2$ [*M*+H]$^+$: 492.0875, found: 492.0877.

(±)-Ethyl (*Z*)-2-(3-hydroxy-2-((1-((4-(trifluoromethyl)phenyl)sulfonyl)pyrrolidine-3-carbonyl)imino)-2,3-dihydrothiazol-4-yl)acetate (37b)

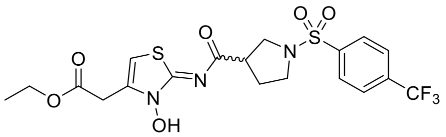

According to General Procedure B, racemic *N*-hydroxythiazole **37b** (93 mg, 0.18 mmol, 43%) was obtained from thiazole **37a** (209 mg, 0.43 mmol), following column chromatography (10 g Sfär Silica D; 35 mL/min; 100% dichloromethane (2 CV), followed by a linear gradient (20 CV): 0%→5% methanol in dichloromethane).

White solid, m.p.: 95-98 °C; ^{1}H NMR (600 MHz, 300K, DMSO-d_6): δ = 8.02 (d, *J* = 8.5 Hz, 2H), 7.98 (d, *J* = 8.5 Hz, 2H), 7.19 (s, 1H), 4.09 (q, *J* = 7.0 Hz, 2H), 3.75 (s, 2H), 3.55 (dd, *J* = 10.0, 7.5 Hz, 1H), 3.41 – 3.34 (m, 2H), 3.32 – 3.20 (m, 2H), 2.08 – 2.02 (m, 1H), 2.00 – 1.89 (m, 1H), 1.18 (t, *J* = 7.0 Hz, 3H) ppm; ^{13}C NMR (151 MHz, 300K, DMSO-d_6): δ = 172.4 (br), 168.5, 143.7 (br), 139.9, 135.9, 132.7 (q, *J* = 32.0 Hz), 128.3, 126.5 (q, *J* = 3.5 Hz), 123.5 (q, *J* = 273.0 Hz), 107.5, 60.6, 50.4, 47.6, 43.1, 31.8, 28.6, 14.0 ppm; ^{19}F NMR (565 MHz, 300K, DMSO-d_6): δ = –61.6 ppm; IR (film): $\tilde{\nu}$ = 2980, 1730, 1693, 1548, 1342, 1157, 1025 cm^{-1}; HRMS (ESI): *m/z* calcd for $C_{19}H_{21}F_3N_3O_6S_2$ [*M*+H]$^+$: 508.0818, found: 508.0836.

(±)-(*Z*)-2-(3-Hydroxy-2-((1-((4-(trifluoromethyl)phenyl)sulfonyl)pyrrolidine-3-carbonyl)imino)-2,3-dihydrothiazol-4-yl)acetic acid (37)

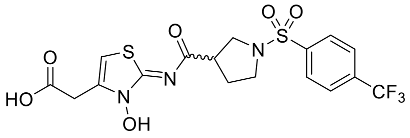

According to General Procedure C, racemic carboxylic acid **37** (28 mg, 0.06 mmol, 59%) was obtained from ethyl ester **37b** (50 mg, 0.10 mmol), following trituration with H_2O (3 × 5 mL; Milli-Q® Ultrapure grade) and MeOH (3 × 5 mL; HPLC grade).

White solid, m.p.: 183-185 °C; ^{1}H NMR (600 MHz, 300K, DMSO-d_6): δ = 8.02 (d, *J* = 8.5 Hz, 2H), 7.99 (d, *J* = 8.5 Hz, 2H), 7.19 (s, 1H), 3.71 (s, 2H), 3.60 – 3.52 (m, 1H), 3.44 – 3.33 (m, 2H), 3.33 – 3.22 (m, 2H), 2.11 – 2.02 (m, 1H), 2.02 – 1.91 (m, 1H) ppm; ^{13}C NMR (151 MHz, 300K, DMSO-d_6): δ = 172.5 (br), 169.6, 144.6 (br), 139.9, 135.9, 132.7 (q, *J* = 32.0 Hz), 128.3, 126.5 (q, *J* = 4.0 Hz), 123.5 (q, *J* = 273.0 Hz), 107.5, 50.4, 47.6, 43.1, 32.6, 28.6 ppm; ^{19}F NMR (565 MHz, 300K, DMSO-d_6): δ = –61.6 ppm; IR (film): $\tilde{\nu}$ = 3085, 1689, 1547, 1346, 1322, 1164, 1131, 1062 cm^{-1}; HRMS (ESI): *m/z* calcd for $C_{17}H_{17}F_3N_3O_6S_2$ [*M*+H]$^+$: 480.0505, found: 480.0513.

(±)-Ethyl 2-(2-(1-([1,1'-biphenyl]-4-ylsulfonyl)pyrrolidine-3-carboxamido)thiazol-4-yl)acetate (38a)

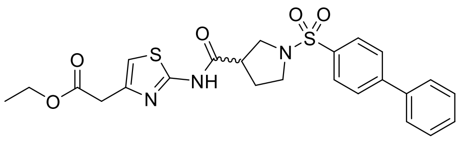

According to General Procedure E, racemic sulfonamide **38a** (316 mg, 0.63 mmol, 81%) was obtained from amine HCl salt **32** (250 mg, 0.78 mmol) and 4-biphenylsulfonyl chloride (238 mg, 0.94 mmol), following column chromatography (25 g Sfär Silica D; 60 mL/min; 100% cyclohexane (2 CV), followed by a linear gradient (12 CV): 0%→60% ethyl acetate in cyclohexane).

White solid, m.p.: 134-136 °C; ^{1}H NMR (600 MHz, 300K, $CDCl_3$): δ = 7.90 (d, *J* = 8.0 Hz, 2H), 7.72 (d, *J* = 8.0 Hz, 2H), 7.60 (d, *J* = 8.0 Hz, 2H), 7.50 – 7.46 (m, 2H), 7.42 (t, *J* = 8.0 Hz, 1H), 6.73 (s, 1H), 4.17 (q, *J* = 7.0 Hz, 2H), 3.73 (dd, *J* = 10.0, 8.0 Hz, 1H), 3.67 (s, 2H), 3.52 – 3.36 (m, 3H), 3.15 – 3.08 (m, 1H), 2.22 – 2.04 (m, 2H), 1.25 (t, *J* = 7.0 Hz, 3H) ppm; ^{13}C NMR (151 MHz, 300K, $CDCl_3$): δ = 170.6, 169.6, 157.5, 146.1, 143.3, 139.4, 134.8, 129.2, 128.7, 128.3, 127.9, 127.5, 111.4, 61.4, 50.6, 47.7, 44.1, 37.1, 28.9, 14.3 ppm; IR (film): $\tilde{\nu}$ = 2972, 1734, 1558, 1341, 1162 cm^{-1}; HRMS (ESI): *m/z* calcd for $C_{24}H_{25}N_3O_6S_2Na$ [*M*+Na]$^+$: 522.1128, found: 522.1143.

(±)-Ethyl (*Z*)-2-(2-((1-([1,1'-biphenyl]-4-ylsulfonyl)pyrrolidine-3-carbonyl)imino)-3-hydroxy-2,3-dihydrothiazol-4-yl)acetate (38b)

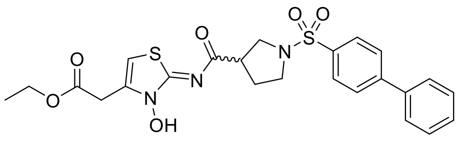

According to General Procedure B, racemic *N*-hydroxythiazole **38b** (100 mg, 0.19 mmol, 67%) was obtained from thiazole **38a** (138 mg, 0.29 mmol), following column chromatography (10 g Sfär Silica D; 35 mL/min; 100% dichloromethane (2 CV), followed by a linear gradient (20 CV): 0%→5% methanol in dichloromethane).

White solid, m.p.: 88-90 °C; ^{1}H NMR (600 MHz, 300K, $CDCl_3$): δ = 7.89 (d, *J* = 8.0 Hz, 2H), 7.72 (d, *J* = 8.2 Hz, 2H), 7.61 (d, *J* = 8.0 Hz, 2H), 7.51 – 7.46 (m, 2H), 7.42 (t, *J* = 8.0 Hz, 1H), 6.87 (s, 1H), 4.20 (q, *J* = 7.0 Hz, 2H), 3.77 (s, 2H), 3.72 (dd, *J* = 10.0, 7.5 Hz, 1H), 3.52 – 3.47 (m, 2H), 3.46 – 3.35 (m, 2H), 2.26 – 2.06 (m, 2H), 1.28 (t, *J* = 7.0 Hz, 3H) ppm; ^{13}C NMR (151 MHz, 300K, $CDCl_3$): δ = 170.8, 168.4, 145.9, 142.8, 139.4, 136.9, 135.1, 129.2, 128.7, 128.3, 127.9, 127.5, 107.8, 61.9, 50.7, 47.7, 43.6, 32.1, 29.0, 14.3 ppm; IR (film): $\tilde{\nu}$ = 2980, 1736, 1692, 1548, 1341, 1156, 1025 cm^{-1}; HRMS (ESI): *m/z* calcd for $C_{24}H_{25}N_3O_6S_2Na$ [*M*+Na]$^+$: 538.1077, found: 538.1079.

(±)-(*Z*)-2-(2-((1-([1,1'-Biphenyl]-4-ylsulfonyl)pyrrolidine-3-carbonyl)imino)-3-hydroxy-2,3-dihydrothiazol-4-yl)acetic acid (38)

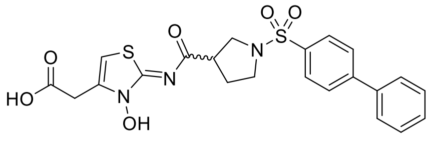

According to General Procedure C, racemic carboxylic acid **38** (28 mg, 0.06 mmol, 57%) was obtained from ethyl ester **38b** (50 mg, 0.10 mmol), following trituration with H_2O (3 × 5 mL; Milli-Q® Ultrapure grade) and MeOH (3 × 5 mL; HPLC grade).

White solid, m.p.: 180-183 °C; ^{1}H NMR (600 MHz, 300K, DMSO-d_6): δ = 7.96 – 7.84 (m, 4H), 7.75 (d, *J* = 7.5 Hz, 2H), 7.54 – 7.49 (m, 2H), 7.45 (t, *J* = 7.5 Hz, 1H), 7.15 (s, 1H), 3.70 (s, 2H), 3.56 (dd, *J* = 10.0, 7.5 Hz, 1H), 3.45 – 3.32 (m, 2H), 3.33 – 3.19 (m, 2H), 2.12 – 2.00 (m, 1H), 2.01 – 1.84 (m, 1H) ppm; ^{13}C NMR (151 MHz, 300K, DMSO-d_6): δ = 172.8 (br), 170.1, 144.9, 144.6, 138.8, 136.5, 135.0, 129.6, 129.1, 128.6, 127.9, 127.6, 108.1, 50.9, 48.1, 43.5, 33.1, 29.0 ppm; IR (film): $\tilde{\nu}$ = 2980, 1736, 1698, 1548, 1341, 1156, 1026 cm^{-1}; HRMS (ESI): *m/z* calcd for $C_{22}H_{22}N_3O_6S_2$ [*M*+H]$^+$: 448.0945, found: 448.0951.

(±)-Ethyl 2-(2-(1-((4-phenoxyphenyl)sulfonyl)pyrrolidine-3-carboxamido)thiazol-4-yl)acetate (39a)

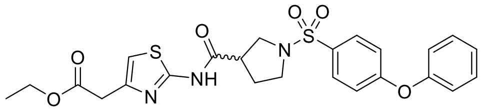

According to General Procedure E, racemic sulfonamide **39a** (341 mg, 0.66 mmol, 85%) was obtained from amine HCl salt **32** (250 mg, 0.78 mmol) and 4-phenoxybenzenesulfonyl chloride (253 mg, 0.94 mmol), following column chromatography (25 g Sfär Silica D; 60 mL/min; 100% cyclohexane (2 CV), followed by a linear gradient (12 CV): 0%→60% ethyl acetate in cyclohexane).

Yellow oil; ^{1}H NMR (600 MHz, 300K, $CDCl_3$): δ = 7.77 (d, *J* = 7.5 Hz, 2H), 7.44 – 7.39 (m, 2H), 7.22 (t, *J* = 7.5 Hz, 1H), 7.08 (d, *J* = 7.5 Hz, 2H), 7.03 (d, *J* = 7.5 Hz, 2H), 6.77 (s, 1H), 4.16 (q, *J* = 7.0 Hz, 2H), 3.75 – 3.61 (m, 3H), 3.44 – 3.34 (m, 2H), 3.33 – 3.28 (m, 1H), 3.15 – 3.08 (m, 1H), 2.21 – 2.03 (m, 2H), 1.25 (t, *J* = 7.0 Hz, 3H) ppm; ^{13}C NMR (151 MHz, 300K, $CDCl_3$): δ = 170.9, 169.9, 162.0, 157.5, 155.1, 143.5, 130.3, 130.0, 129.7, 125.1, 120.5, 117.7, 111.4, 61.4, 50.6, 47.6, 43.9, 37.2, 28.8, 14.2 ppm; IR (film): $\tilde{\nu}$ = 3098, 1737, 1688, 1547, 1347, 1156, 1026 cm^{-1}; HRMS (ESI): *m/z* calcd for $C_{24}H_{26}N_3O_6S_2$ [*M*+H]$^+$: 516.1258, found: 516.1266.

(±)-Ethyl (*Z*)-2-(3-hydroxy-2-((1-((4-phenoxyphenyl)sulfonyl)pyrrolidine-3-carbonyl)imino)-2,3-dihydrothiazol-4-yl)acetate (39b)

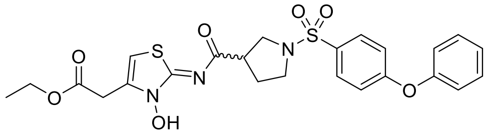

According to General Procedure B, racemic *N*-hydroxythiazole **39b** (160 mg, 0.30 mmol, 50%) was obtained from thiazole **39a** (300 mg, 0.60 mmol), following column chromatography (10 g Sfär Silica D; 35 mL/min; 100% dichloromethane (2 CV), followed by a linear gradient (20 CV): 0%→5% methanol in dichloromethane).

White solid, m.p.: 69-72 °C; ^{1}H NMR (600 MHz, 300K, $CDCl_3$): δ = 7.76 (d, *J* = 8.0 Hz, 2H), 7.43 – 7.39 (m, 2H), 7.22 (t, *J* = 7.5 Hz, 1H), 7.09 (d, *J* = 7.5 Hz, 2H), 7.04 (d, *J* = 7.5 Hz, 2H), 6.98 (s, 1H), 4.21 (q, *J* = 7.0 Hz, 2H), 3.80 (s, 2H), 3.67 (dd, *J* = 10.0, 7.5 Hz, 1H), 3.60 – 3.53 (m, 1H), 3.49 – 3.43 (m, 1H), 3.34 – 3.26 (m, 2H), 2.25 – 2.13 (m, 1H), 2.13 – 2.04 (m, 1H), 1.29 (t, *J* = 7.0 Hz, 3H) ppm; ^{13}C NMR (151 MHz, 300K, $CDCl_3$): δ = 171.1, 168.4, 161.9, 155.3, 143.0, 137.0, 130.3, 130.0(4), 130.0(2), 125.1, 120.5, 117.8, 107.9, 61.9, 50.6, 47.6, 43.5, 32.1, 29.1, 14.3 ppm; IR (film): $\tilde{\nu}$ = 2980, 1735, 1699, 1549, 1340, 1152, 1024 cm^{-1}; HRMS (ESI): *m/z* calcd for $C_{24}H_{26}N_3O_7S_2$ [*M*+H]$^+$: 532.1207, found: 532.1221.

(±)-(*Z*)-2-(3-Hydroxy-2-((1-((4-phenoxyphenyl)sulfonyl)pyrrolidine-3-carbonyl)imino)-2,3-dihydrothiazol-4-yl)acetic acid (39)

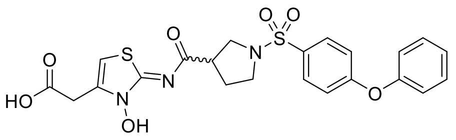

According to General Procedure C, racemic carboxylic acid **39** (27 mg, 0.05 mmol, 28%) was obtained from ethyl ester **39b** (100 mg, 0.19 mmol), following trituration with H_2O (3 × 5 mL; Milli-Q® Ultrapure grade) and MeOH (3 × 5 mL; HPLC grade).

White solid, m.p.: 179-183 °C; ^{1}H NMR (600 MHz, 300K, DMSO-d_6): δ = 7.80 (d, *J* = 8.0 Hz, 2H), 7.50 – 7.45 (m, 2H), 7.27 (t, *J* = 7.5 Hz, 1H), 7.21 (s, 1H), 7.17 (d, *J* = 7.5 Hz, 2H), 7.12 (d, *J* = 7.5 Hz, 2H), 3.71 (s, 2H), 3.49 (dd, *J* = 10.0, 8.0 Hz, 1H), 3.42 – 3.35 (m, 1H), 3.33 – 3.28 (m, 1H), 3.28 – 3.15 (m, 2H), 2.09 – 1.99 (m, 1H), 1.99 – 1.87 (m, 1H) ppm; ^{13}C NMR (151 MHz, 300K, DMSO-d_6): δ = 172.9 (br), 170.2, 161.5, 155.2, 144.8 (br), 136.5, 130.9, 130.5, 130.1, 125.5, 120.7, 118.0, 108.0, 50.9, 48.1, 43.5, 33.2, 29.0 ppm; IR (film): $\tilde{\nu}$ = 2980, 1735, 1699, 1550, 1340, 1153, 1023 cm^{-1}; HRMS (ESI): *m/z* calcd for $C_{22}H_{22}N_3O_7S_2$ [*M*+H]$^+$: 504.0894, found: 504.0892.

(±)-Ethyl 2-(2-(1-(naphthalen-2-ylsulfonyl)pyrrolidine-3-carboxamido)thiazol-4-yl)acetate (40a)

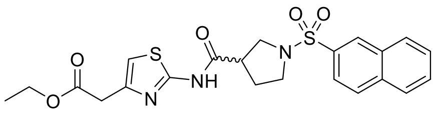

According to General Procedure E, racemic sulfonamide **40a** (352 mg, 0.74 mmol, 95%) was obtained from amine HCl salt **32** (250 mg, 0.78 mmol) and naphthalene-2-sulfonyl chloride (213 mg, 0.94 mmol), following column chromatography (25 g Sfär Silica D; 60 mL/min; 100% cyclohexane (2 CV), followed by a linear gradient (12 CV): 0%→60% ethyl acetate in cyclohexane).

Yellow oil; ^{1}H NMR (600 MHz, 300K, $CDCl_3$): δ = 8.40 (s, 1H), 7.98 – 7.95 (m, 2H), 7.91 (d, *J* = 8.0 Hz, 1H), 7.82 (dd, *J* = 8.5, 2.0 Hz, 1H), 7.66 – 7.57 (m, 2H), 6.74 (s, 1H), 4.17 (q, *J* = 7.0 Hz, 2H), 3.74 (dd, *J* = 10.5, 8.0 Hz, 1H), 3.66 (s, 2H), 3.54 – 3.39 (m, 3H), 3.08 – 3.02 (m, 1H), 2.20 – 2.02 (m, 2H), 1.26 (t, *J* = 7.0 Hz, 3H) ppm; ^{13}C NMR (151 MHz, 300K, $CDCl_3$): δ = 170.5, 169.6, 157.5, 143.2, 135.2, 133.5, 132.4, 129.6, 129.5, 129.1, 129.0, 128.1, 127.7, 123.0, 111.4, 61.4, 50.6, 47.7, 44.1, 37.0, 28.9, 14.3 ppm; IR (film): $\tilde{\nu}$ = 3274, 2984, 1733, 1687, 1546, 1341, 1159, 1029 cm^{-1}; HRMS (ESI): *m/z* calcd for $C_{22}H_{24}N_3O_5S_2$ [*M*+H]$^+$: 474.1152, found: 474.1159.

(±)-Ethyl (*Z*)-2-(3-hydroxy-2-((1-(naphthalen-2-ylsulfonyl)pyrrolidine-3-carbonyl)imino)-2,3-dihydrothiazol-4-yl)acetate (40b)

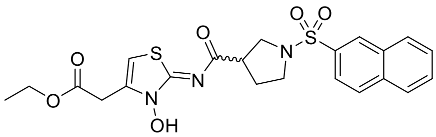

According to General Procedure B, racemic *N*-hydroxythiazole **40b** (72 mg, 0.15 mmol, 43%) was obtained from thiazole **40a** (157 mg, 0.34 mmol), following column chromatography (10 g Sfär Silica D; 35 mL/min; 100% dichloromethane (2 CV), followed by a linear gradient (20 CV): 0%→5% methanol in dichloromethane).

White solid, m.p.: 95-98 °C; ^{1}H NMR (600 MHz, 300K, $CDCl_3$): δ = 8.38 (s, 1H), 7.96 (dd, *J* = 8.5, 6.0 Hz, 2H), 7.91 (d, *J* = 8.0 Hz, 1H), 7.82 (d, *J* = 9.0 Hz, 1H), 7.68 – 7.53 (m, 2H), 6.85 (s, 1H), 4.18 (q, *J* = 7.0 Hz, 2H), 3.77 – 3.68 (m, 3H), 3.55 – 3.48 (m, 2H), 3.47 – 3.37 (m, 2H), 2.19 – 2.03 (m, 2H), 1.27 (t, *J* = 7.0 Hz, 3H) ppm; ^{13}C NMR (151 MHz, 300K, $CDCl_3$): δ = 171.0, 168.4, 142.7, 136.8, 135.1, 133.7, 132.4, 129.5, 129.5, 129.1, 128.9, 128.1, 127.7, 123.1, 107.8, 61.9, 50.8, 47.7, 43.5, 32.0, 29.0, 14.3 ppm; IR (film): $\tilde{\nu}$ = 2980, 1732, 1692, 1548, 1342, 1156, 1026 cm^{-1}; HRMS (ESI): *m/z* calcd for $C_{22}H_{24}N_3O_6S_2$ [*M*+H]$^+$: 490.1101, found: 490.1114.

(±)-(*Z*)-2-(3-Hydroxy-2-((1-(naphthalen-2-ylsulfonyl)pyrrolidine-3-carbonyl)imino)-2,3-dihydrothiazol-4-yl)acetic acid (40)

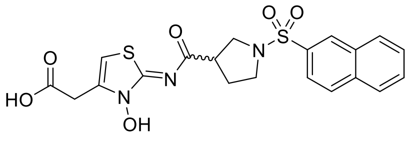

According to General Procedure C, racemic carboxylic acid **40** (15 mg, 0.03 mmol, 33%) was obtained from ethyl ester **40b** (50 mg, 0.10 mmol), following trituration with H_2O (3 × 5 mL; Milli-Q® Ultrapure grade) and MeOH (3 × 5 mL; HPLC grade).

White solid, m.p.: 181-184 °C; ^{1}H NMR (600 MHz, 300K, DMSO-d_6): δ = 8.48 (s, 1H), 8.19 (d, *J* = 8.0 Hz, 1H), 8.13 (d, *J* = 8.5 Hz, 1H), 8.05 (d, *J* = 8.0 Hz, 1H), 7.82 (dd, *J* = 8.5, 2.0 Hz, 1H), 7.73 – 7.65 (m, 2H), 7.14 (s, 1H), 3.70 (s, 2H), 3.59 (dd, *J* = 10.5, 8.0 Hz, 1H), 3.43 – 3.37 (m, 1H), 3.37 – 3.20 (m, 3H), 2.05 – 1.96 (m, 1H), 1.94 – 1.88 (m, 1H) ppm; ^{13}C NMR (151 MHz, 300K, DMSO-d_6): δ = 172.3 (br), 169.7, 144.2 (br), 135.9, 134.5, 133.0, 131.8, 129.4, 129.3, 128.9, 128.5, 127.8, 127.6, 122.8, 107.6, 50.6, 47.7, 43.1, 32.7, 28.5 ppm; IR (film): $\tilde{\nu}$ = 2980, 1736, 1698, 1548, 1337, 1154, 1025 cm^{-1}; HRMS (ESI): *m/z* calcd for $C_{20}H_{20}N_3O_6S_2$ $[M+H]^+$: 462.0788, found: 462.0789.

(±)-Ethyl 2-(2-(1-(thiophen-2-ylsulfonyl)pyrrolidine-3-carboxamido)thiazol-4-yl)acetate (41a)

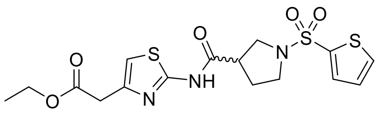

According to General Procedure E, racemic sulfonamide **41a** (301 mg, 0.70 mmol, 90%) was obtained from amine HCl salt **32** (250 mg, 0.78 mmol) and thiophene-2-sulfonyl chloride (172 mg, 0.94 mmol), following column chromatography (25 g Sfär Silica D; 60 mL/min; 100% cyclohexane (2 CV), followed by a linear gradient (12 CV): 0%→60% ethyl acetate in cyclohexane).

Yellow oil; ^{1}H NMR (600 MHz, 300K, $CDCl_3$): δ = 7.62 – 7.59 (m, 2H), 7.12 (dd, *J* = 5.0, 4.0 Hz, 1H), 6.78 (s, 1H), 4.17 (q, *J* = 7.0 Hz, 2H), 3.73 – 3.65 (m, 3H), 3.49 – 3.36 (m, 3H), 3.11 – 3.05 (m, 1H), 2.15 – 2.10 (m, 2H), 1.25 (t, *J* = 7.0 Hz, 3H) ppm; ^{13}C NMR (151 MHz, 300K, $CDCl_3$): δ = 170.9, 169.6, 157.5, 143.5, 136.1, 132.8, 132.4, 127.9, 111.4, 61.5, 50.7, 47.9, 44.0, 37.2, 28.9, 14.3 ppm; IR (film): $\tilde{\nu}$ = 2981, 1733, 1687, 1546, 1347, 1156, 1028 cm^{-1}; HRMS (ESI): *m/z* calcd for $C_{16}H_{20}N_3O_5S_3$ $[M+H]^+$: 430.0560, found: 430.0566.

(±)-Ethyl (*Z*)-2-(3-hydroxy-2-((1-(thiophen-2-ylsulfonyl)pyrrolidine-3-carbonyl)imino)-2,3-dihydrothiazol-4-yl)acetate (41b)

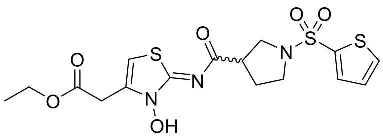

According to General Procedure B, racemic *N*-hydroxythiazole **41b** (55 mg, 0.12 mmol, 24%) was obtained from thiazole **41a** (212 mg, 0.49 mmol), following column chromatography (10 g Sfär Silica D; 35 mL/min; 100% dichloromethane (2 CV), followed by a linear gradient (20 CV): 0%→5% methanol in dichloromethane).

White solid, m.p.: 87-90 °C; ^{1}H NMR (600 MHz, 300K, DMSO-d_6): δ = 8.01 (dd, *J* = 5.0, 1.5 Hz, 1H), 7.69 (dd, *J* = 3.5, 1.5 Hz, 1H), 7.26 (dd, *J* = 5.0, 3.5 Hz, 1H), 7.19 (s, 1H), 4.09 (q, *J* = 7.0 Hz, 2H), 3.76 (s, 2H), 3.57 – 3.49 (m, 1H), 3.42 – 3.27 (m, 3H), 3.26 – 3.21 (m, 1H), 2.07 – 2.01 (m, 1H), 1.95 – 1.89 (m, 1H), 1.18 (t, *J* = 7.0 Hz, 3H) ppm; ^{13}C NMR (151 MHz, 300K, DMSO-d_6): δ = 172.3 (br), 168.5, 143.6 (br), 136.0, 135.3, 133.5, 132.8, 128.2, 107.6, 60.6, 50.5, 47.9, 43.1, 31.8, 28.6, 14.0 ppm; IR (film): $\tilde{\nu}$ = 3029, 1732, 1692, 1548, 1342, 1156, 1025 cm^{-1}; HRMS (ESI): *m/z* calcd for $C_{16}H_{20}N_3O_6S_3$ $[M+H]^+$: 446.0509, found: 446.0528.

(±)-(*Z*)-2-(3-Hydroxy-2-((1-(thiophen-2-ylsulfonyl)pyrrolidine-3-carbonyl)imino)-2,3-dihydrothiazol-4-yl)acetic acid (41)

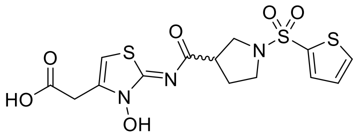

According to General Procedure C, racemic carboxylic acid **41** (19 mg, 0.05 mmol, 58%) was obtained from ethyl ester **41b** (50 mg, 0.08 mmol), following trituration with H_2O (3 × 5 mL; Milli-Q® Ultrapure grade) and MeOH (3 × 5 mL; HPLC grade).

White solid, m.p.: 178-180 °C; ^{1}H NMR (600 MHz, 300K, DMSO-d_6): δ = 8.01 (dd, *J* = 5.0, 1.5 Hz, 1H), 7.69 (dd, *J* = 3.5, 1.5 Hz, 1H), 7.26 (dd, *J* = 5.0, 3.5 Hz, 1H), 7.20 (s, 1H), 3.71 (s, 2H), 3.56 – 3.50 (m, 1H), 3.43 – 3.19 (m, 4H), 2.09 – 2.03 (m, 1H), 1.97 – 1.88 (m, 1H) ppm; ^{13}C NMR (151 MHz, 300K, DMSO-d_6): δ = 172.3 (br), 169.7, 144.4 (br), 136.0, 135.3, 133.5, 132.8, 128.2, 107.6, 50.5, 47.9, 43.1, 32.6, 28.6 ppm; IR (film): $\tilde{\nu}$ = 3190, 1734, 1699, 1552, 1342, 1152, 1023 cm^{-1}; HRMS (ESI): *m/z* calcd for $C_{14}H_{16}N_3O_6S_3$ [*M*+H]$^+$: 418.0196, found: 418.0199.

(±)-Ethyl 2-(2-(1-(benzylsulfonyl)pyrrolidine-3-carboxamido)thiazol-4-yl)acetate (42a)

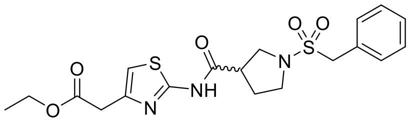

According to General Procedure E, racemic sulfonamide **42a** (294 mg, 0.67 mmol, 86%) was obtained from amine HCl salt **32** (250 mg, 0.78 mmol) and phenylmethanesulfonyl chloride (179 mg, 0.94 mmol), following column chromatography (25 g Sfär Silica D; 60 mL/min; 100% cyclohexane (2 CV), followed by a linear gradient (12 CV): 0%→60% ethyl acetate in cyclohexane).

Yellow oil; ^{1}H NMR (600 MHz, 300K, $CDCl_3$): δ = 7.45 – 7.38 (m, 2H), 7.36 – 7.32 (m, 3H), 6.80 (s, 1H), 4.31 (s, 2H), 4.17 (q, *J* = 7.0 Hz, 2H), 3.69 (s, 2H), 3.61 (dd, *J* = 10.0, 7.5 Hz, 1H), 3.45 (dd, *J* = 10.0, 7.0 Hz, 1H), 3.36 – 3.32 (m, 1H), 3.28 – 3.24 (m, 1H), 3.13 – 3.07 (m, 1H), 2.21 – 2.04 (m, 2H), 1.25 (t, *J* = 7.0 Hz, 3H) ppm; ^{13}C NMR (151 MHz, 300K, $CDCl_3$): δ = 170.8, 170.0, 157.6, 143.5, 130.9, 129.1, 128.9, 128.9, 111.4, 61.4, 56.8, 50.4, 47.9, 44.4, 37.2, 29.6, 14.3 ppm; IR (film): $\tilde{\nu}$ = 3274, 2981, 1733, 1686, 1546, 1331, 1151, 1030 cm^{-1}; HRMS (ESI): *m/z* calcd for $C_{19}H_{24}N_3O_5S_2$ [*M*+H]$^+$: 438.1152, found: 438.1165.

(±)-Ethyl (*Z*)-2-(2-((1-(benzylsulfonyl)pyrrolidine-3-carbonyl)imino)-3-hydroxy-2,3-dihydrothiazol-4-yl)acetate (42b)

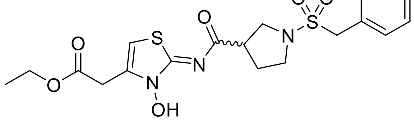

According to General Procedure B, racemic *N*-hydroxythiazole **42b** (65 mg, 0.14 mmol, 33%) was obtained from thiazole **42a** (187 mg, 0.44 mmol), following column chromatography (10 g Sfär Silica D; 35 mL/min; 100% dichloromethane (2 CV), followed by a linear gradient (20 CV): 0%→5% methanol in dichloromethane).

White solid, m.p.: 79-81 °C; ^{1}H NMR (600 MHz, 300K, DMSO-d_6): δ = 7.44 – 7.40 (m, 2H), 7.40 – 7.32 (m, 3H), 7.23 (s, 1H), 4.46 (s, 2H), 4.10 (q, *J* = 7.0 Hz, 2H), 3.82 – 3.72 (m, 2H), 3.58 – 3.44 (m, 2H), 3.44 – 3.34 (m, 1H), 3.33 – 3.25 (m, 1H), 3.23 – 3.18 (m, 1H), 2.18 – 2.00 (m, 2H), 1.19 (t, *J* = 7.0 Hz, 3H) ppm; ^{13}C NMR (151 MHz, 300K, DMSO-d_6): δ = 172.4 (br), 168.5, 143.2 (br), 136.2, 130.9, 129.8, 128.3, 128.1, 107.7, 60.6, 53.6, 50.1, 47.4, 43.5, 31.8, 29.1, 14.0 ppm; IR (film): $\tilde{\nu}$ = 2980, 1736, 1694, 1547, 1339, 1155, 1025 cm^{-1}; HRMS (ESI): *m/z* calcd for $C_{19}H_{24}N_3O_6S_2$ [*M*+H]$^+$: 454.1101, found: 454.1102.

(±)-(*Z*)-2-(2-((1-(Benzylsulfonyl)pyrrolidine-3-carbonyl)imino)-3-hydroxy-2,3-dihydrothiazol-4-yl)acetic acid (42)

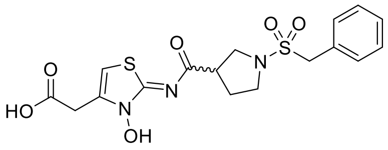

According to General Procedure C, racemic carboxylic acid **42** (24 mg, 0.06 mmol, 51%) was obtained from ethyl ester **42b** (50 mg, 0.11 mmol), following trituration with H_2O (3 × 5 mL; Milli-Q® Ultrapure grade) and MeOH (3 × 5 mL; HPLC grade).

White solid, m.p.: 194-197 °C; ^{1}H NMR (600 MHz, 300K, DMSO-d_6): δ = 7.45 – 7.40 (m, 2H), 7.40 – 7.32 (m, 3H), 7.24 (s, 1H), 4.46 (s, 2H), 3.78 – 3.68 (m, 2H), 3.58 – 3.48 (m, 2H), 3.43 – 3.35 (m, 1H), 3.32 – 3.27 (m, 1H), 3.23 – 3.18 (m, 1H), 2.18 – 1.98 (m, 2H) ppm; ^{13}C NMR (151 MHz, 300K, DMSO-d_6): δ = 172.4 (br), 169.7, 144.0 (br), 136.1, 130.9, 129.8, 128.3, 128.1, 107.7, 53.6, 50.1, 47.4, 43.5, 32.6, 29.0 ppm; IR (film): $\tilde{\nu}$ = 2980, 1733, 1701, 1550, 1339, 1153, 1024 cm^{-1}; HRMS (ESI): *m/z* calcd for $C_{17}H_{20}N_3O_6S_2$ [*M*+H]$^+$: 426.0788, found: 426.0790.

(±)-Ethyl 2-(2-(1-(cyclopentylsulfonyl)pyrrolidine-3-carboxamido)thiazol-4-yl)acetate (43a)

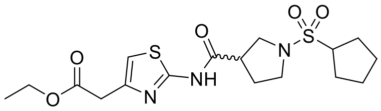

According to General Procedure E, racemic sulfonamide **43a** (272 mg, 0.64 mmol, 84%) was obtained from amine HCl salt **32** (250 mg, 0.78 mmol) and cyclopentanesulfonyl chloride (0.12 mL, 158 mg, 0.94 mmol), following column chromatography (25 g Sfär Silica D; 60 mL/min; 100% cyclohexane (2 CV), followed by a linear gradient (12 CV): 0%→60% ethyl acetate in cyclohexane).

Yellow oil; ^{1}H NMR (600 MHz, 300K, $CDCl_3$): δ = 6.80 (s, 1H), 4.18 (q, *J* = 7.0 Hz, 2H), 3.80 (dd, *J* = 9.0, 8.0 Hz, 1H), 3.69 (s, 2H), 3.64 – 3.46 (m, 4H), 3.25 – 3.18 (m, 1H), 2.25 (q, *J* = 7.0 Hz, 2H), 2.05 – 1.99 (m, 4H), 1.83 – 1.76 (m, 2H), 1.70 – 1.55 (m, 2H), 1.26 (t, *J* = 7.0 Hz, 3H) ppm; ^{13}C NMR (151 MHz, 300K, $CDCl_3$): δ = 170.8, 170.3, 157.6, 143.5, 111.4, 61.4, 61.3, 50.4, 47.8, 44.5, 37.2, 29.9, 28.1, 25.8, 14.3 ppm; IR (film): $\tilde{\nu}$ = 3268, 2957, 1734, 1687, 1546, 1318, 1269, 1142, 1030 cm^{-1}; HRMS (ESI): *m/z* calcd for $C_{17}H_{25}N_3O_5S_2Na$ [*M*+Na]$^+$: 438.1128, found: 438.1142.

(±)-Ethyl (*Z*)-2-(2-((1-(cyclopentylsulfonyl)pyrrolidine-3-carbonyl)imino)-3-hydroxy-2,3-dihydrothiazol-4-yl)acetate (43b)

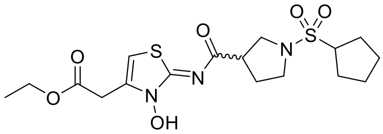

According to General Procedure B, racemic *N*-hydroxythiazole **43b** (64 mg, 0.15 mmol, 46%) was obtained from thiazole **43a** (127 mg, 0.32 mmol), following column chromatography (10 g Sfär Silica D; 35 mL/min; 100% dichloromethane (2 CV), followed by a linear gradient (20 CV): 0%→5% methanol in dichloromethane).

White solid, m.p.: 77-80 °C; ^{1}H NMR (600 MHz, 300K, DMSO-d_6): δ = 7.22 (s, 1H), 4.10 (q, *J* = 7.0 Hz, 2H), 3.77 (s, 2H), 3.74 – 3.68 (m, 1H), 3.59 (dd, *J* = 9.5, 8.0 Hz, 1H), 3.56 – 3.51 (m, 1H), 3.46 – 3.36 (m, 2H), 3.36 – 3.30 (m, 1H), 2.20 – 2.15 (m, 1H), 2.12 – 2.06 (m, 1H), 2.00 – 1.87 (m, 2H), 1.85 – 1.77 (m, 2H), 1.71 – 1.62 (m, 2H), 1.60 – 1.51 (m, 2H), 1.19 (t, *J* = 7.0 Hz, 3H) ppm; ^{13}C NMR (151 MHz, 300K, DMSO-d_6): δ = 172.6 (br), 168.5, 143.1 (br), 136.2, 107.7, 60.6, 59.1, 50.1, 47.3, 43.5, 31.8, 29.2, 27.5, 25.2, 14.0 ppm; IR (film): $\tilde{\nu}$ = 2980, 1736, 1697, 1648, 1340, 1156, 1025 cm^{-1}; HRMS (ESI): *m/z* calcd for $C_{17}H_{25}N_3O_6S_2Na$ [*M*+Na]$^+$: 454.1077, found: 454.1083.

(±)-(*Z*)-2-(2-((1-(Cyclopentylsulfonyl)pyrrolidine-3-carbonyl)imino)-3-hydroxy-2,3-dihydrothiazol-4-yl)acetic acid (43)

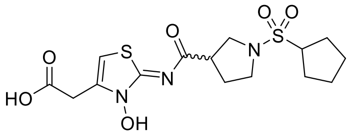

According to General Procedure C, racemic carboxylic acid **43** (24 mg, 0.06 mmol, 50%) was obtained from ethyl ester **43b** (50 mg, 0.12 mmol), following trituration with H_2O (3 × 5 mL; Milli-Q® Ultrapure grade) and MeOH (3 × 5 mL; HPLC grade).

White solid, m.p.: 177-180 °C; ^{1}H NMR (600 MHz, 300K, DMSO-d_6): δ = 7.24 (s, 1H), 3.78 – 3.66 (m, 3H), 3.64 – 3.50 (m, 2H), 3.49 – 3.30 (m, 3H), 2.21 – 2.16 (m, 1H), 2.13 – 2.06 (m, 1H), 2.01 – 1.90 (m, 2H), 1.85 – 1.78 (m, 2H), 1.70 – 1.63 (m, 2H), 1.60 – 1.50 (m, 2H) ppm; ^{13}C NMR (151 MHz, 300K, DMSO-d_6): δ = 172.6 (br), 169.6, 143.9 (br), 136.2, 107.8, 59.1, 50.1, 47.3, 43.4, 32.6, 29.2, 27.6, 25.2 ppm; IR (film): $\tilde{\nu}$ = 2981, 1732, 1703, 1552, 1333, 1153, 1027 cm^{-1}; HRMS (ESI): *m/z* calcd for $C_{15}H_{22}N_3O_6S_2$ [*M*+H]$^+$: 404.0945, found: 404.0949.

^{1}H and ^{13}C NMR spectra of novel compounds prepared for this study

Ethyl 2-(2-(2-(phenylsulfonyl)acetamido)thiazol-4-yl)acetate (2)

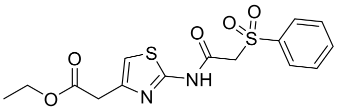

^{1}H NMR (600 MHz, 300 K, $CDCl_3$):

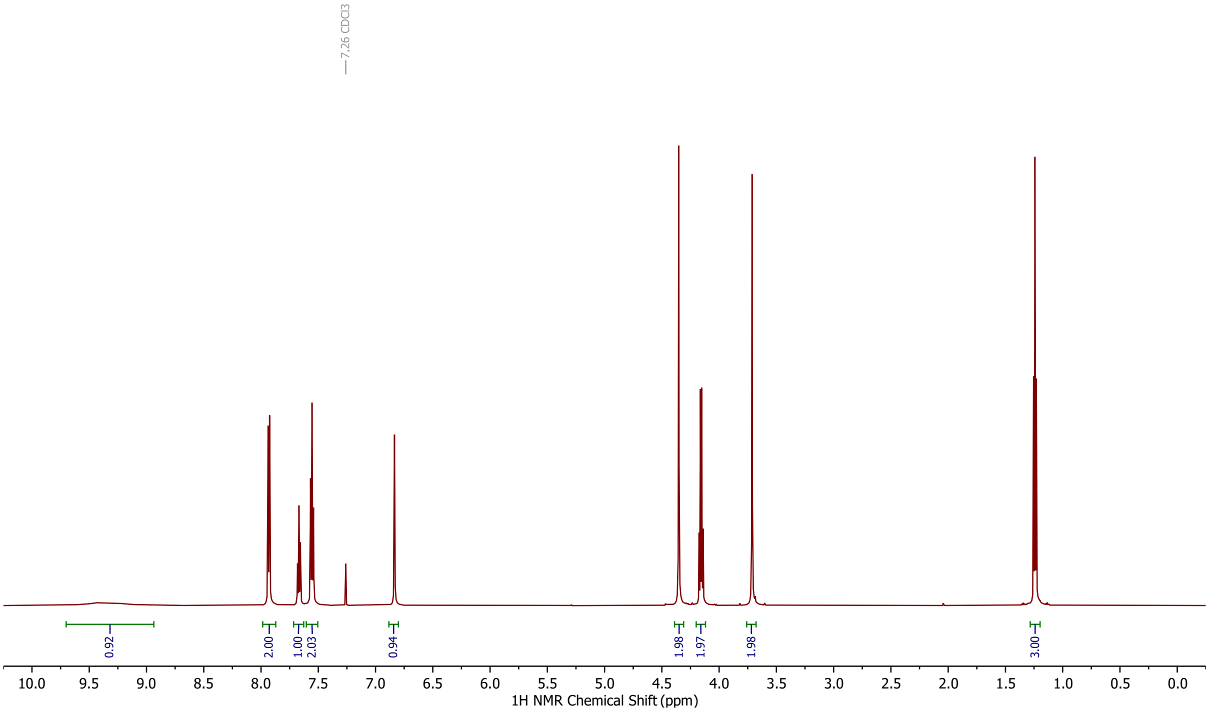

^{13}C NMR (151 MHz, 300 K, $CDCl_3$):

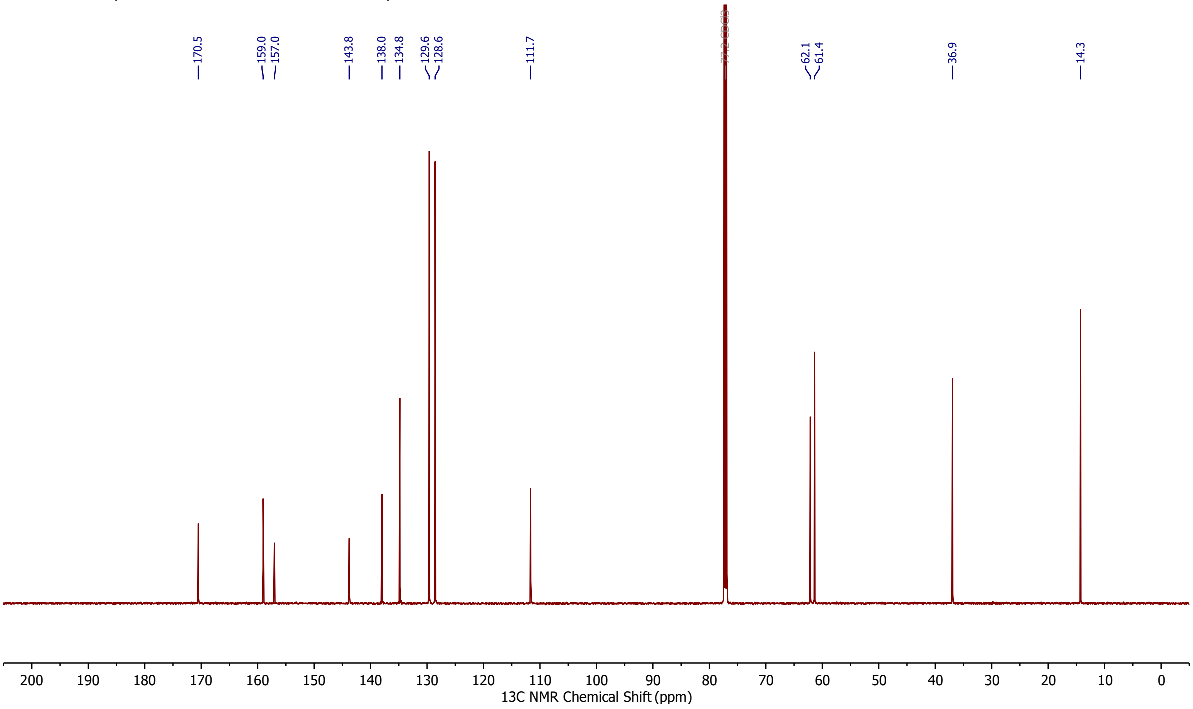

Ethyl (*Z*)-2-(3-hydroxy-2-((2-(phenylsulfonyl)acetyl)imino)-2,3-dihydrothiazol-4-yl)acetate (3)

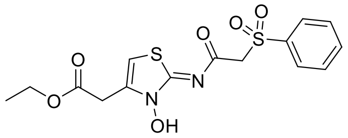

^{1}H NMR (600 MHz, 300 K, DMSO-d_6):

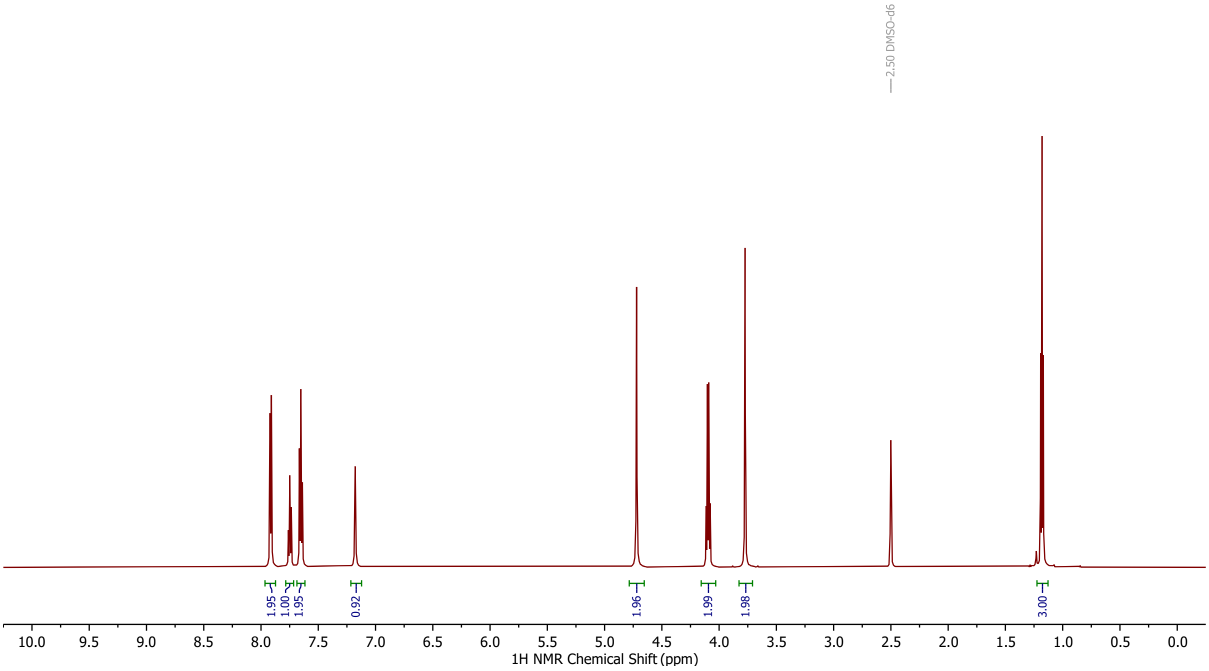

^{13}C NMR (151 MHz, 300 K, DMSO-d_6):

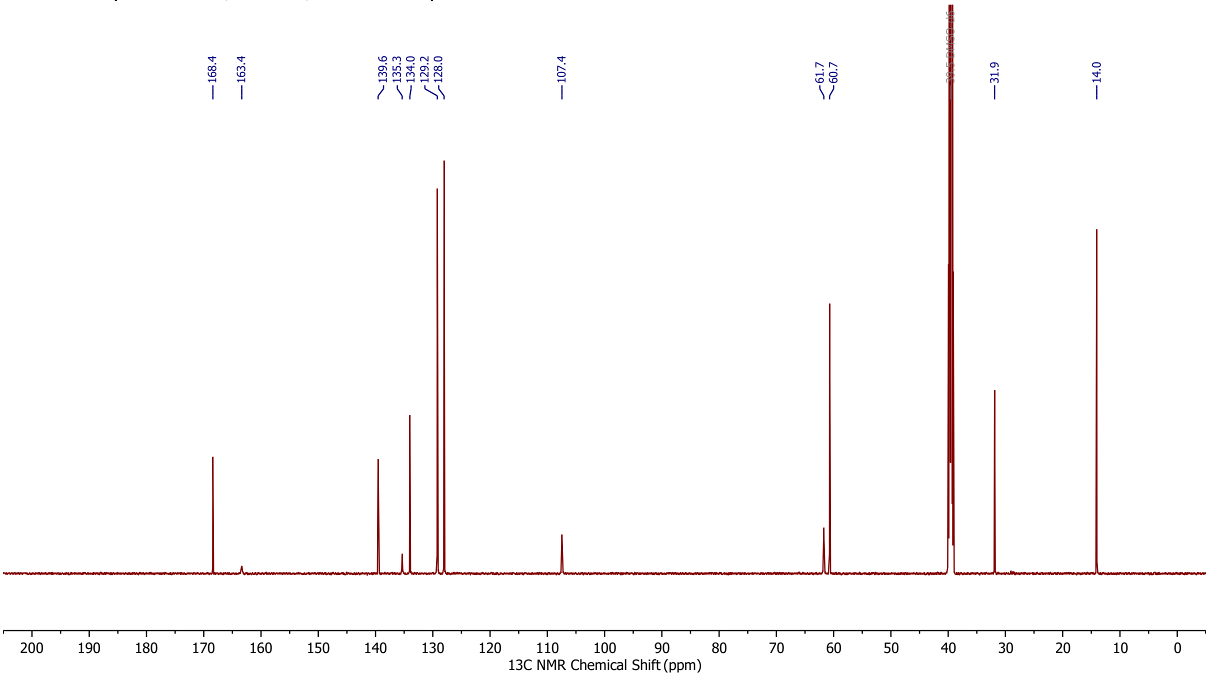

(*Z*)-2-(3-Hydroxy-2-((2-(phenylsulfonyl)acetyl)imino)-2,3-dihydrothiazol-4-yl)acetic acid (4)

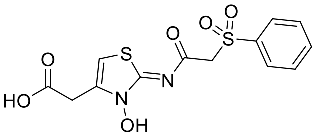

^1H NMR (600 MHz, 300 K, DMSO-d_6):

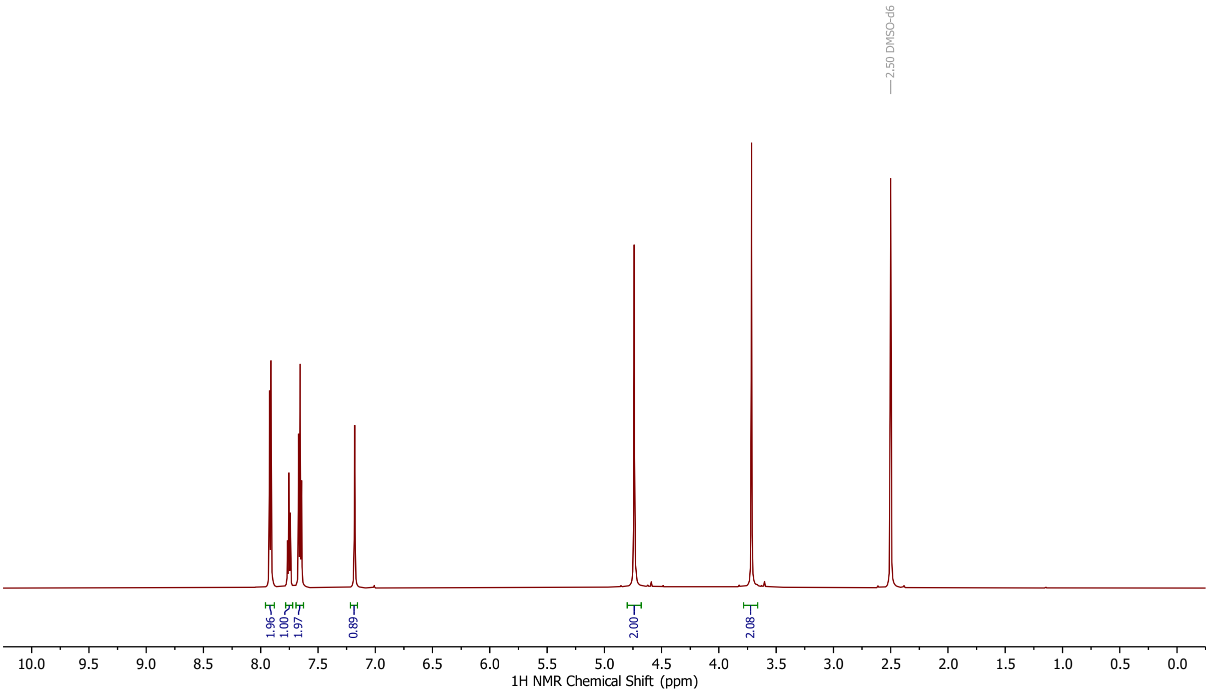

^{13}C NMR (151 MHz, 300 K, DMSO-d_6):

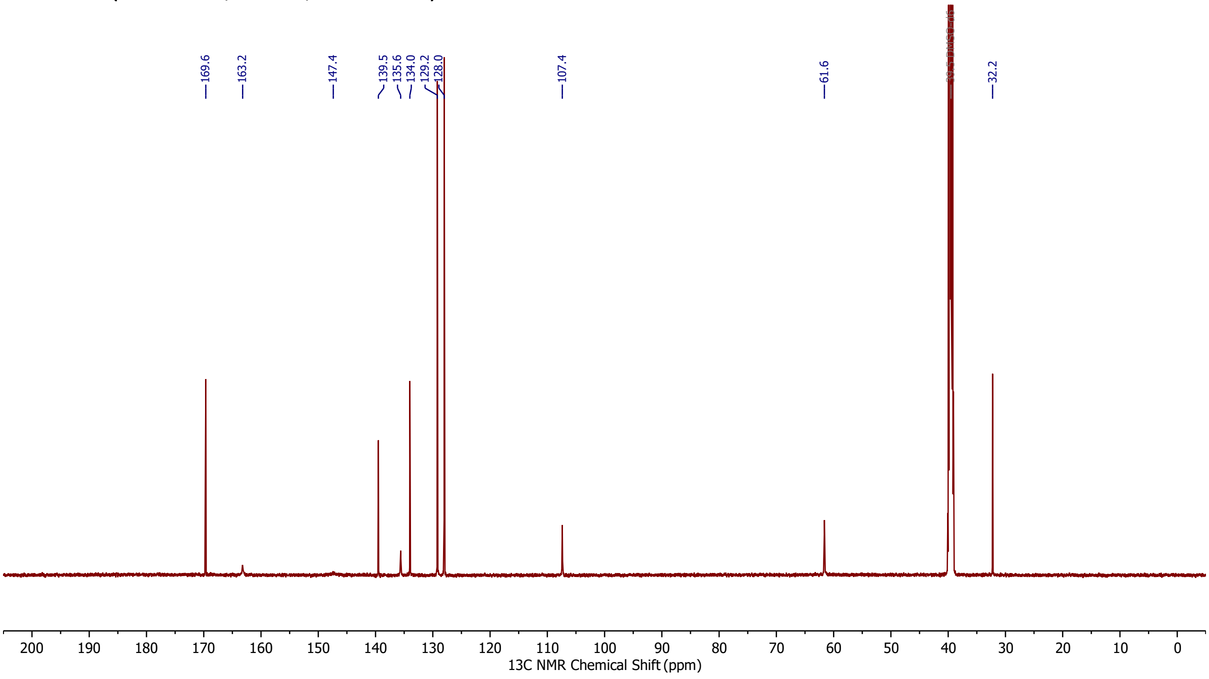

Ethyl 2-(2-amino-5-methylthiazol-4-yl)acetate (5)

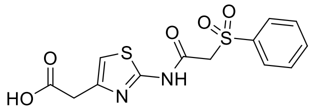

^{1}H NMR (600 MHz, 300 K, DMSO-d_6):

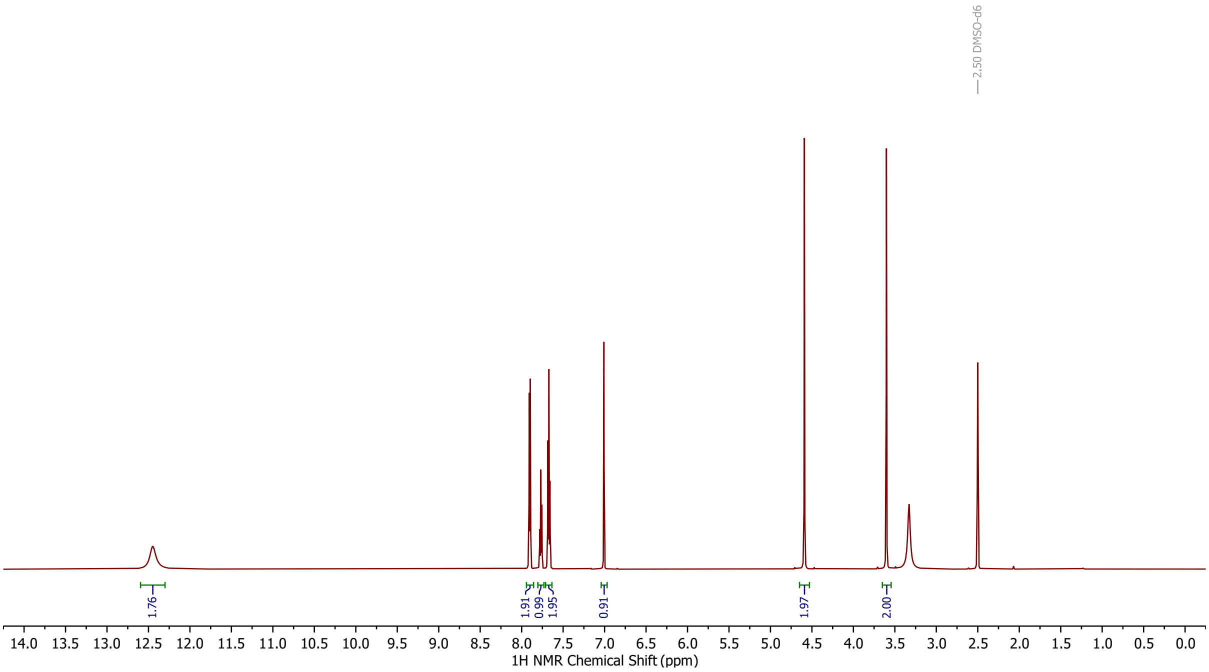

^{13}C NMR (151 MHz, 300 K, DMSO-d_6):

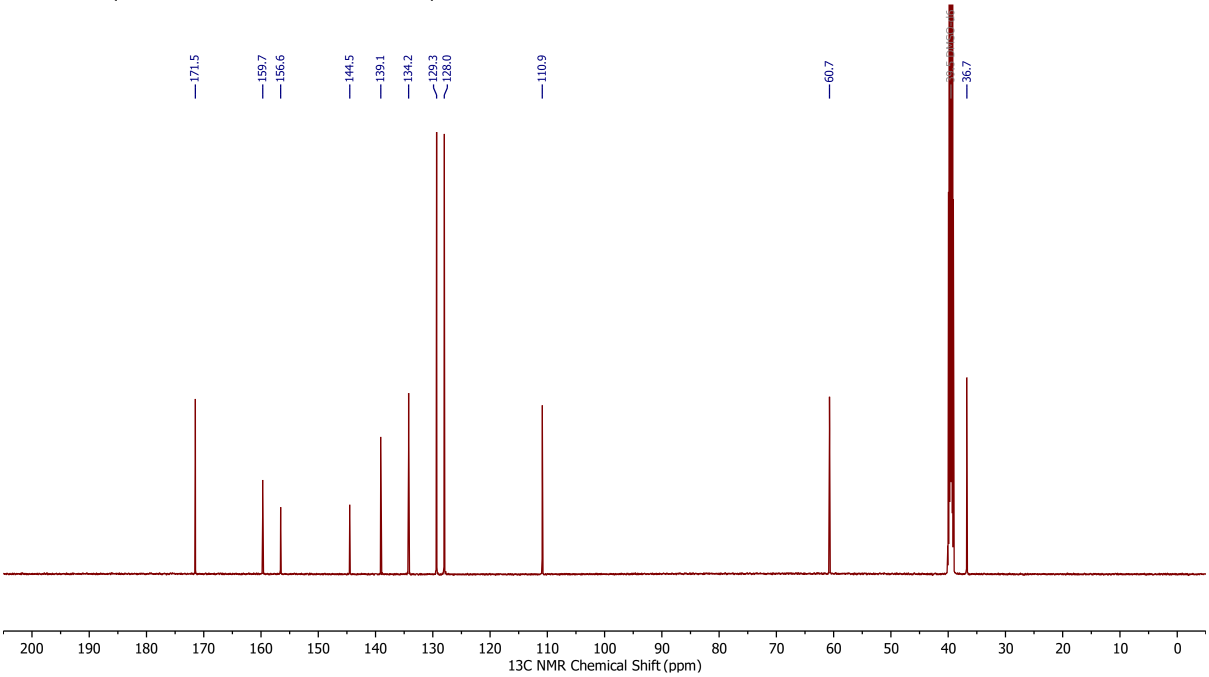

(±)-Ethyl 2-(2-(2-(phenylsulfonyl)acetamido)thiazol-4-yl)propanoate (6c)

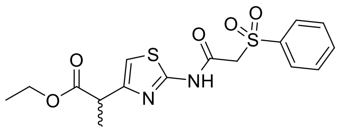

^{1}H NMR (600 MHz, 300 K, $CDCl_3$):

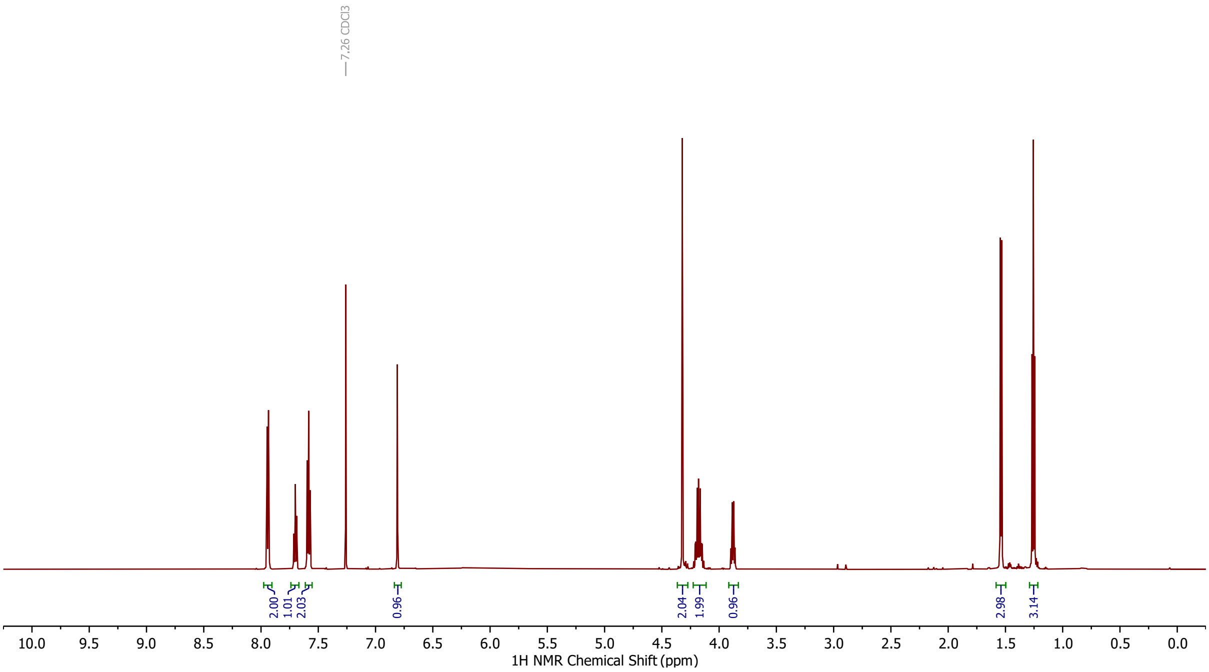

^{13}C NMR (151 MHz, 300 K, $CDCl_3$):

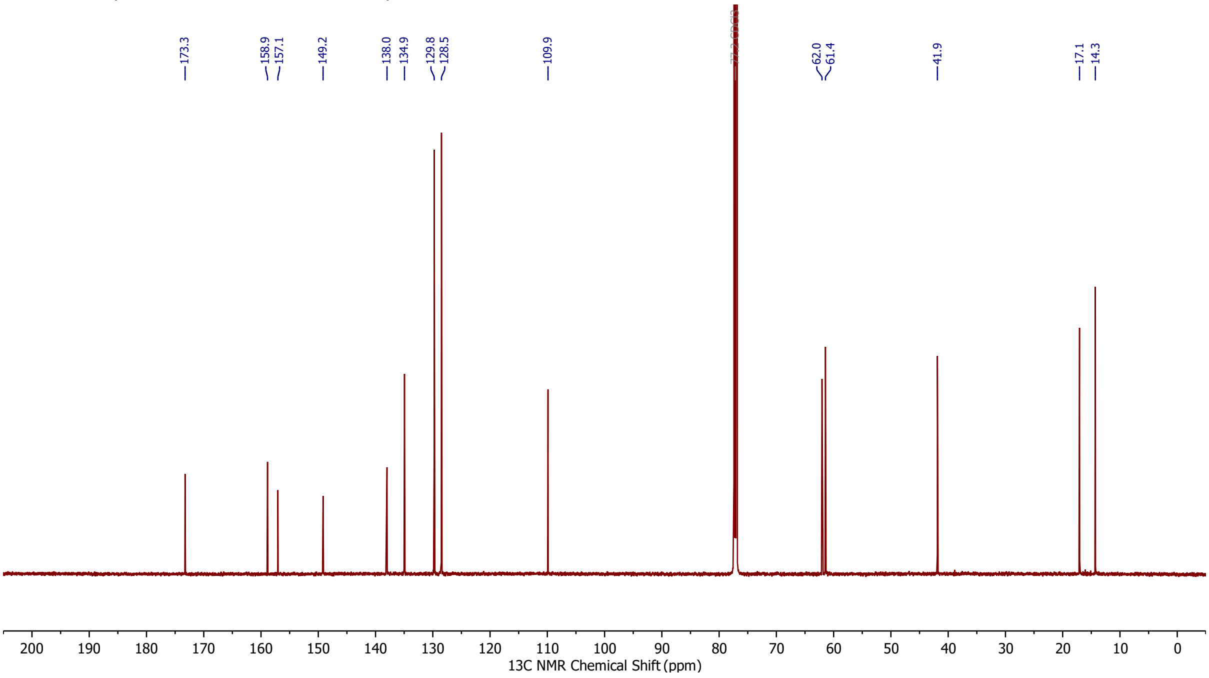

(±)-Ethyl (*Z*)-2-(3-hydroxy-2-((2-(phenylsulfonyl)acetyl)imino)-2,3-dihydrothiazol-4-yl)propanoate (6d)

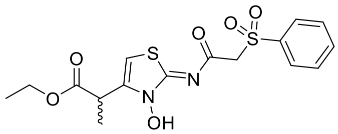

^{1}H NMR (600 MHz, 300 K, $CDCl_3$):

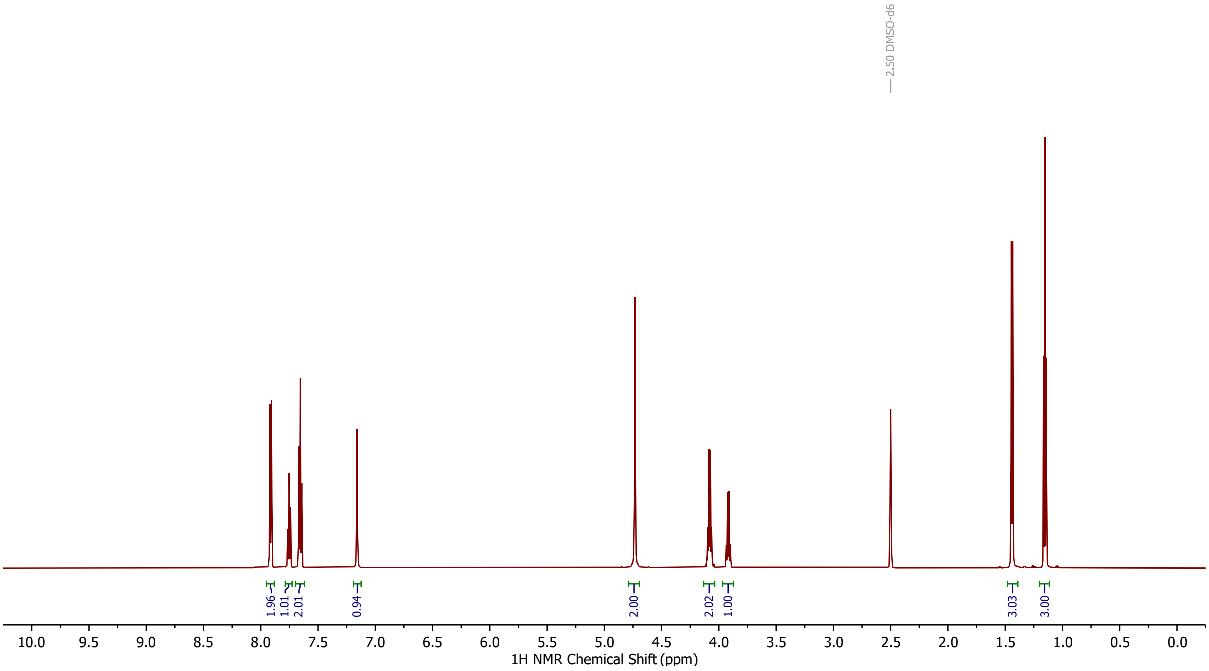

^{13}C NMR (151 MHz, 300 K, $CDCl_3$):

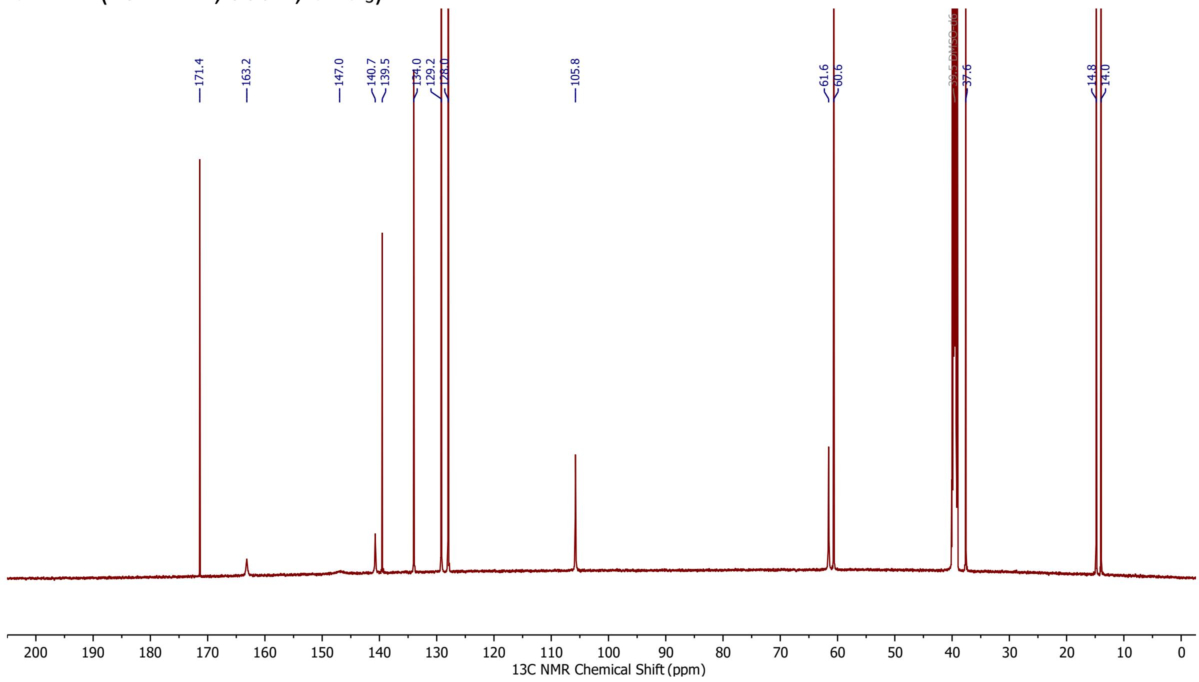

(±)-(*Z*)-2-(3-Hydroxy-2-((2-(phenylsulfonyl)acetyl)imino)-2,3-dihydrothiazol-4-yl)propanoic acid (6)

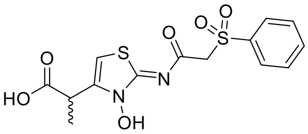

^{1}H NMR (600 MHz, 300 K, DMSO-d_6):

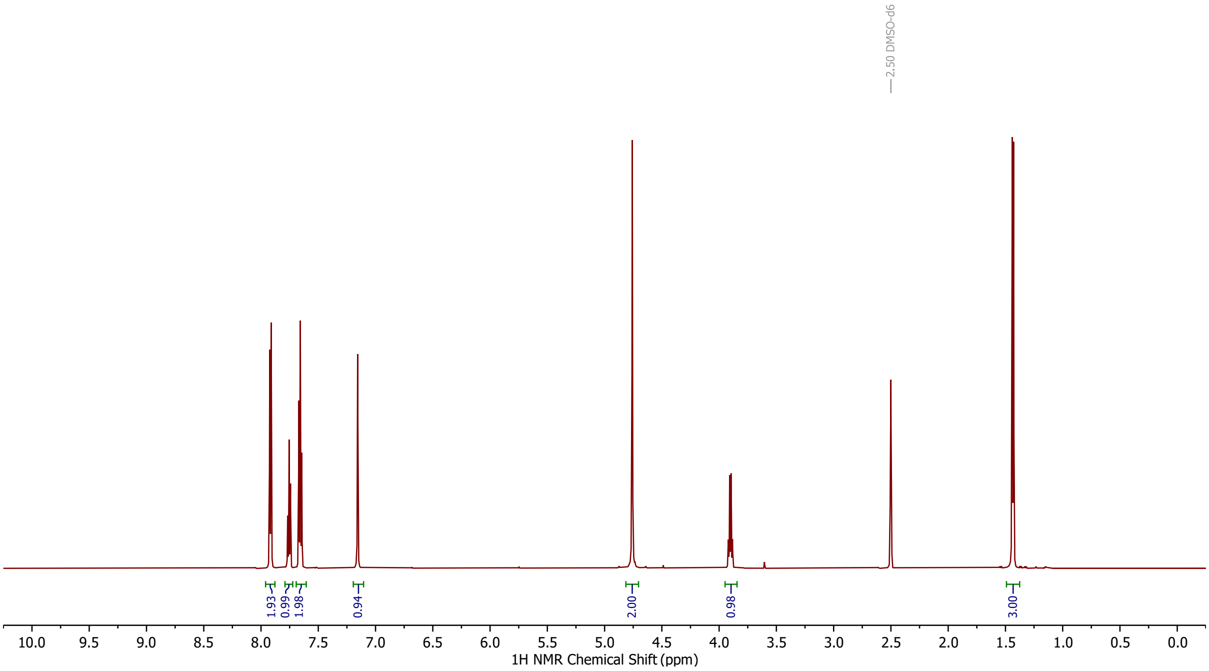

^{13}C NMR (151 MHz, 300 K, DMSO-d_6):

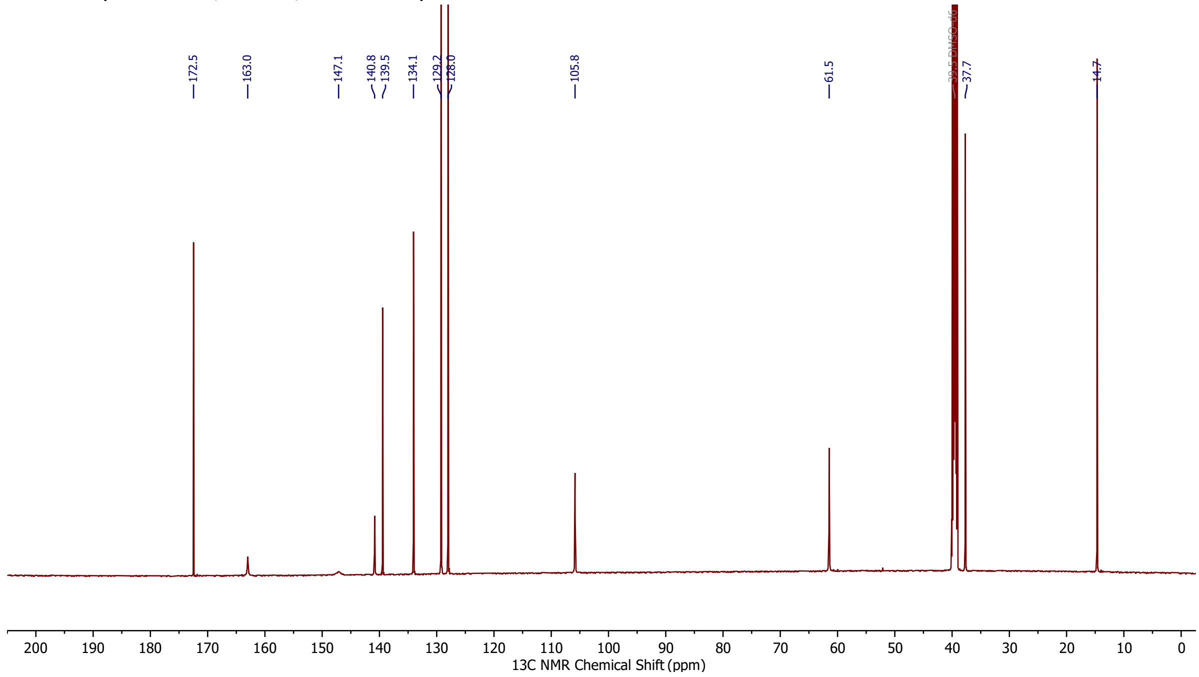

Ethyl 2-(5-methyl-2-(2-(phenylsulfonyl)acetamido)thiazol-4-yl)acetate (7c)

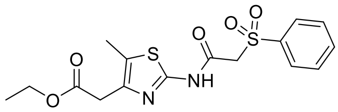

^{1}H NMR (600 MHz, 300 K, DMSO-d_6):

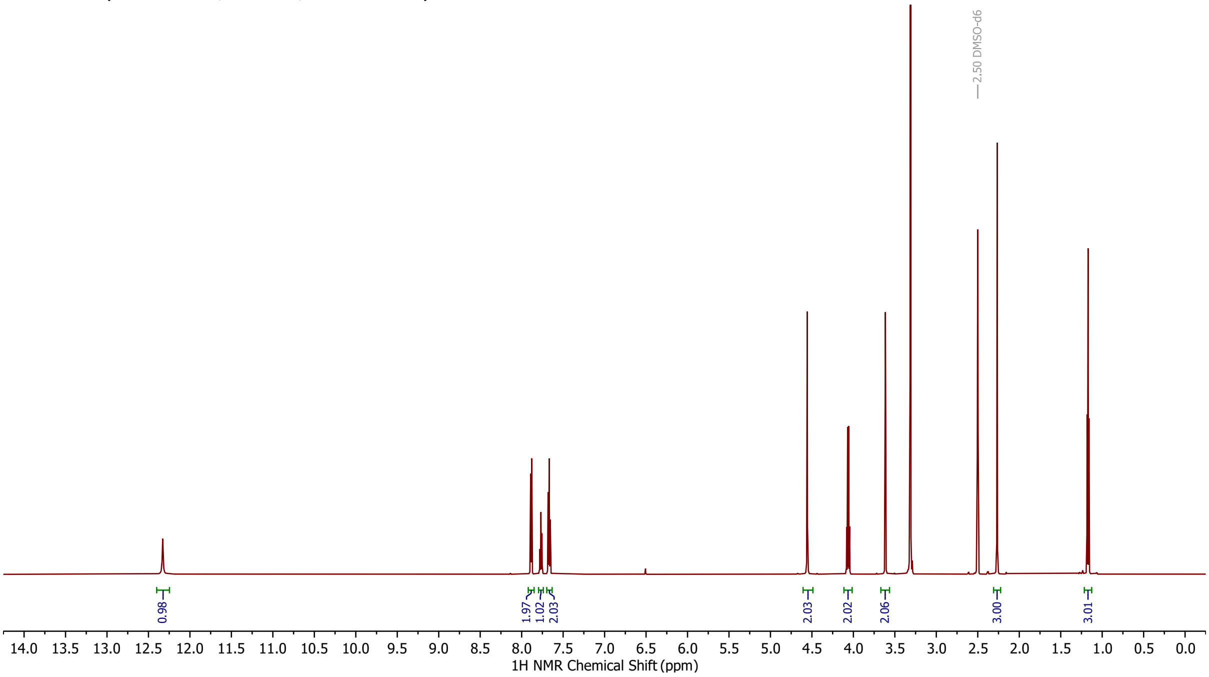

^{13}C NMR (151 MHz, 300 K, DMSO-d_6):

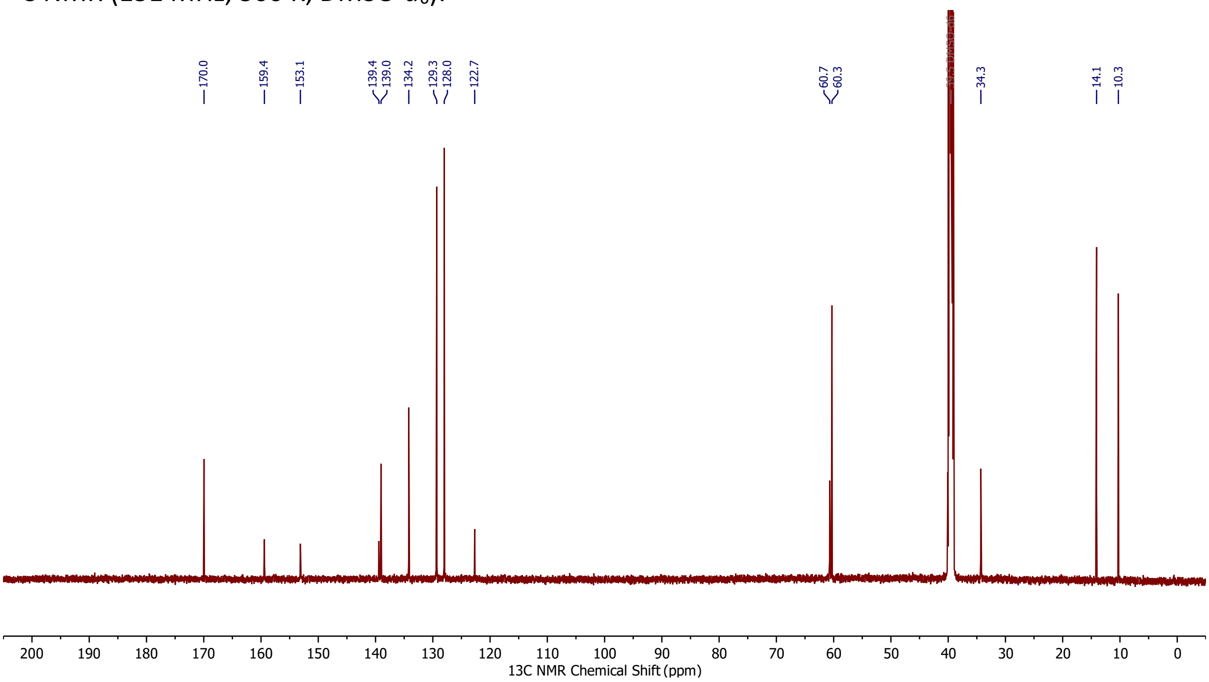

Ethyl (*Z*)-2-(3-hydroxy-5-methyl-2-((2-(phenylsulfonyl)acetyl)imino)-2,3-dihydrothiazol-4-yl)acetate (7d)

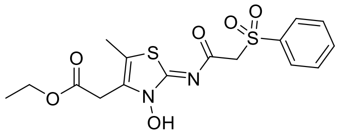

^{1}H NMR (600 MHz, 323 K, DMSO-d_6):

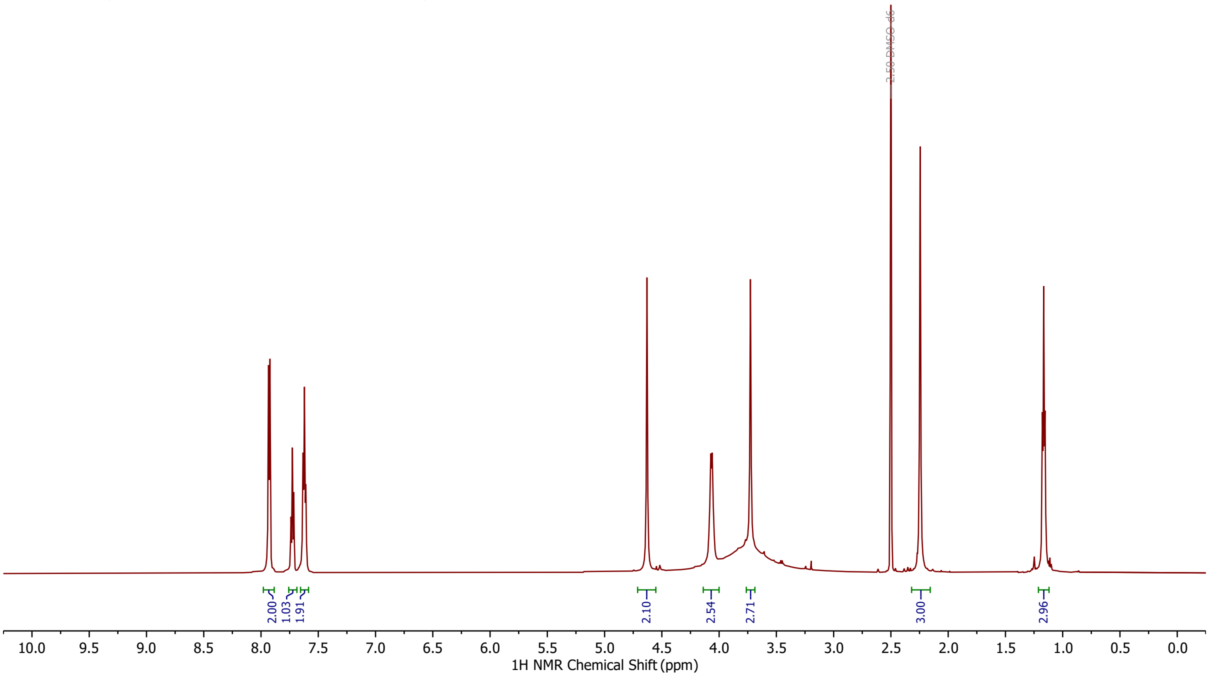

^{13}C NMR (151 MHz, 323 K, DMSO-d_6):

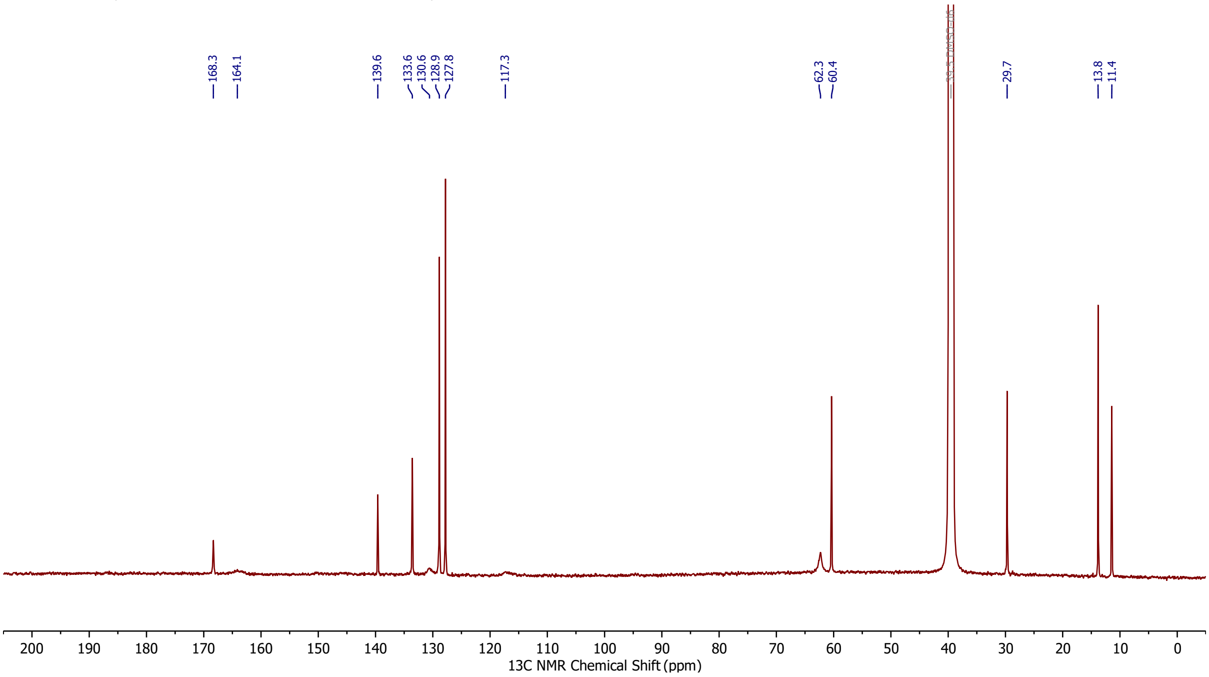

(*Z*)-2-(3-Hydroxy-5-methyl-2-((2-(phenylsulfonyl)acetyl)imino)-2,3-dihydrothiazol-4-yl)acetic acid (7)

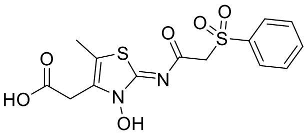

^{1}H NMR (600 MHz, 300 K, DMSO-d_6):

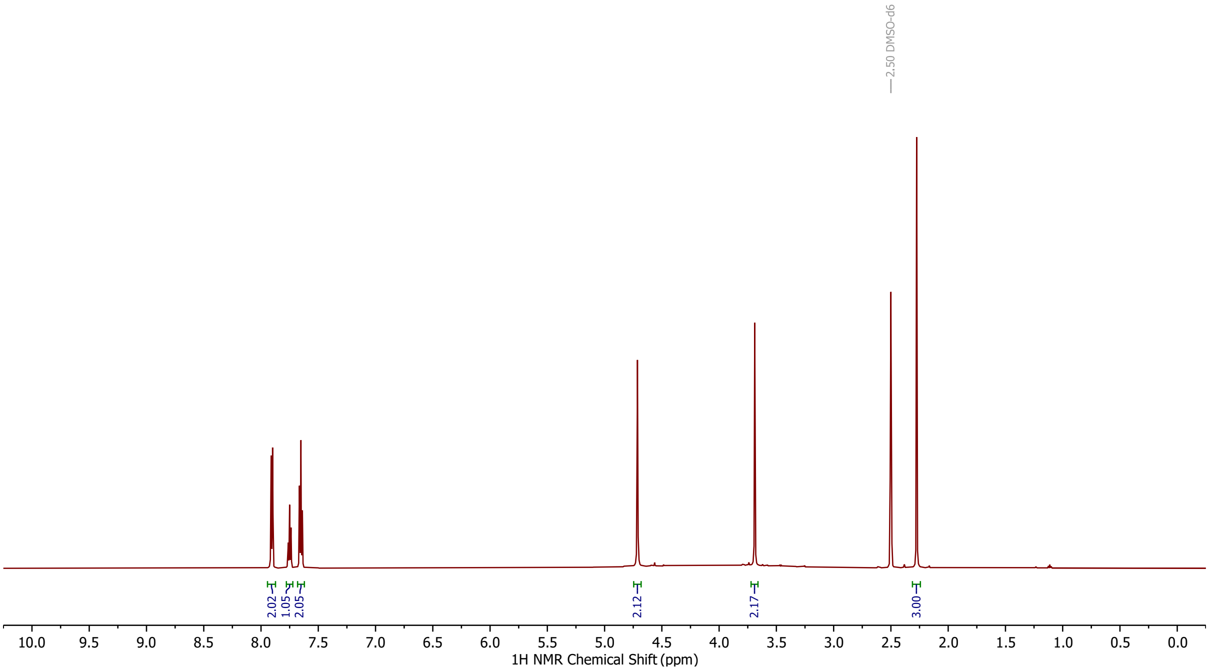

^{13}C NMR (151 MHz, 300 K, DMSO-d_6):

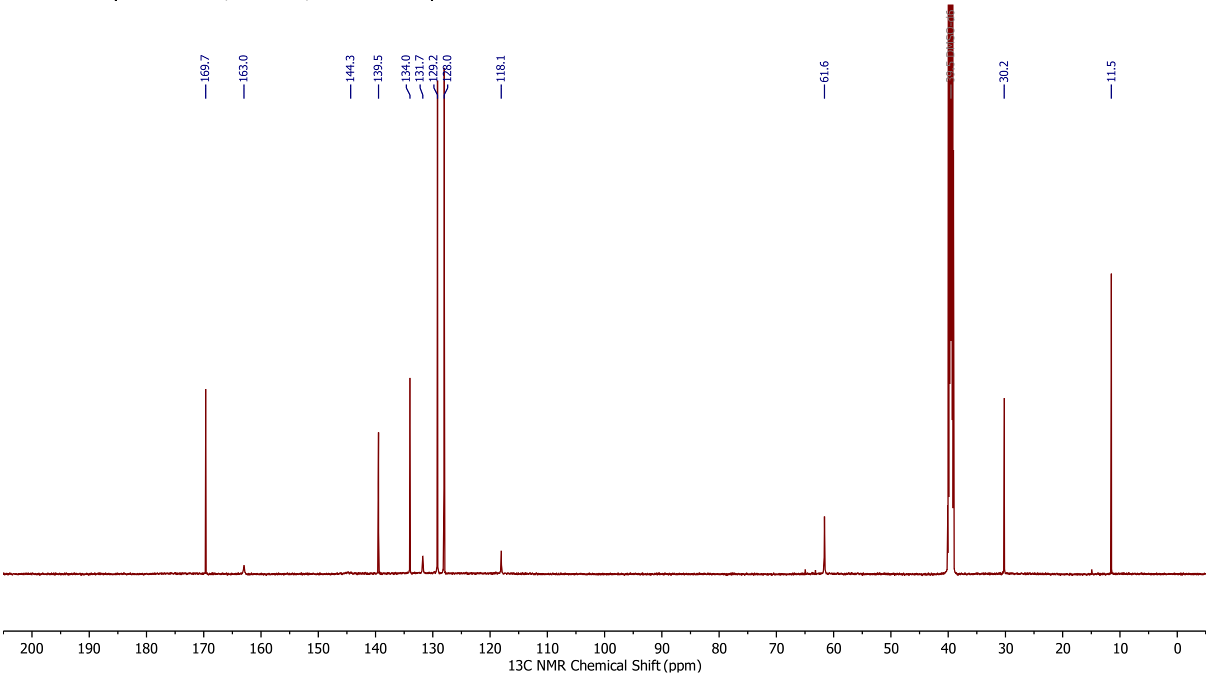

2-(Phenylsulfonyl)-*N*-(thiazol-2-yl)acetamide (8c)

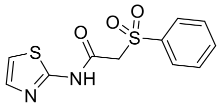

^{1}H NMR (600 MHz, 300 K, DMSO-d_6):

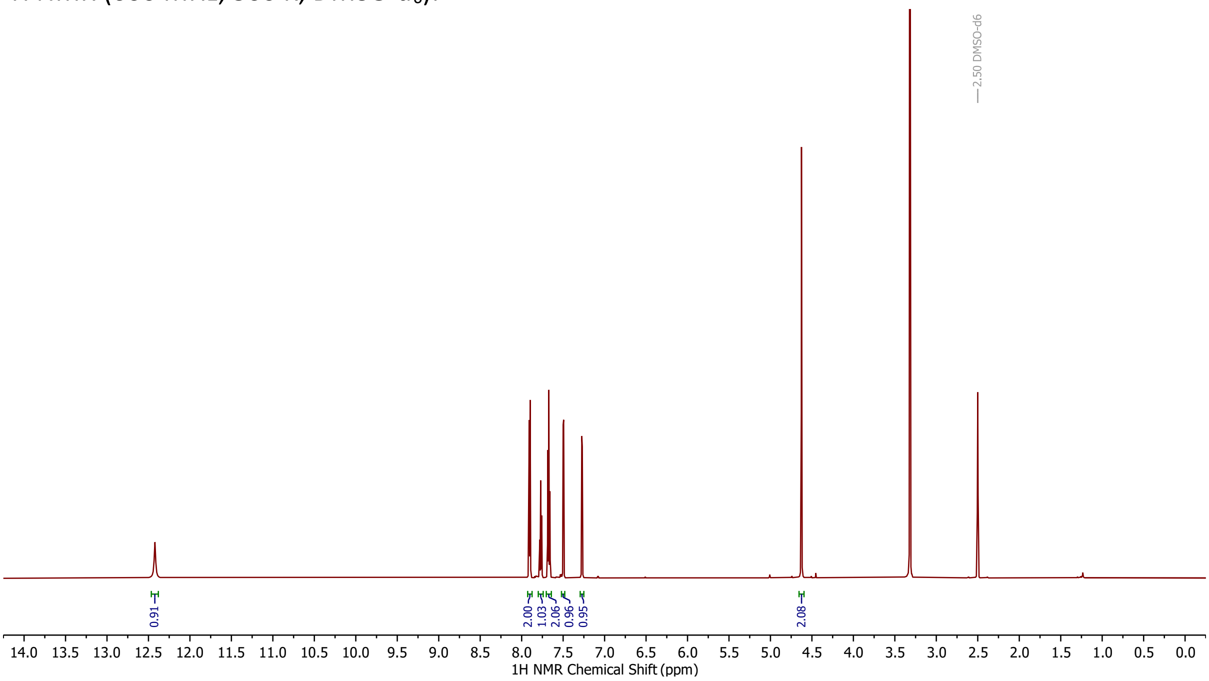

^{13}C NMR (151 MHz, 300 K, DMSO-d_6):

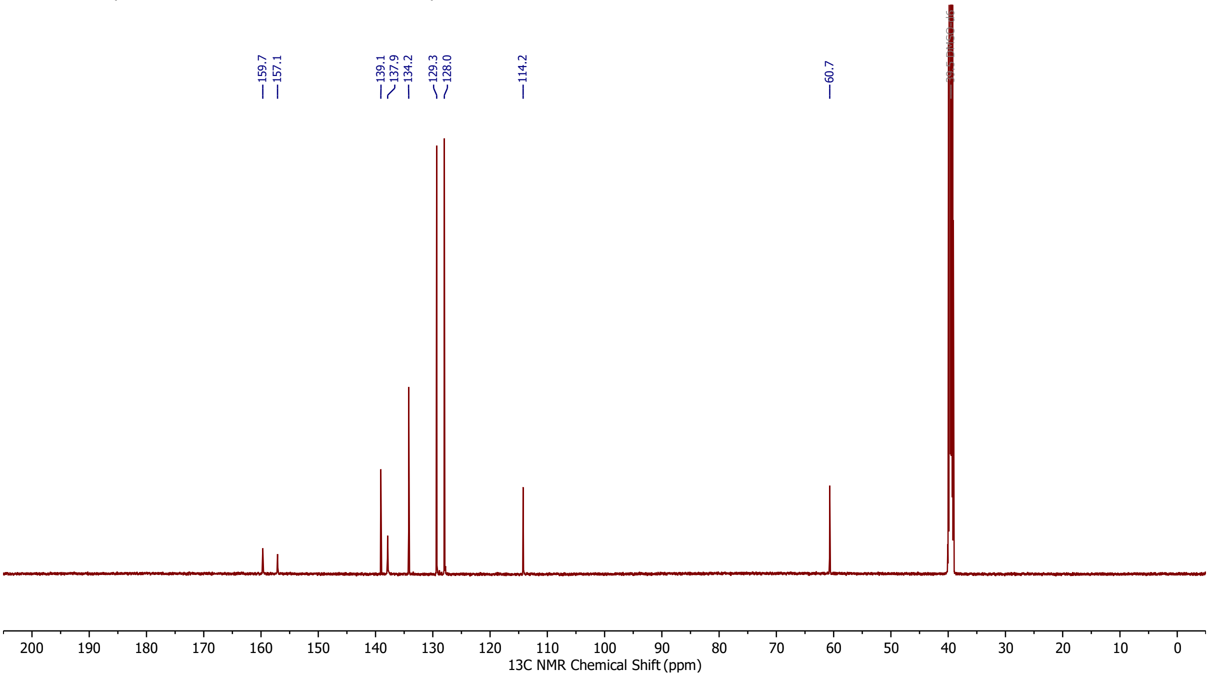

(*Z*)-*N*-(3-Hydroxythiazol-2(3*H*)-ylidene)-2-(phenylsulfonyl)acetamide (8)

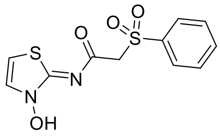

^{1}H NMR (600 MHz, 300 K, DMSO-d_6):

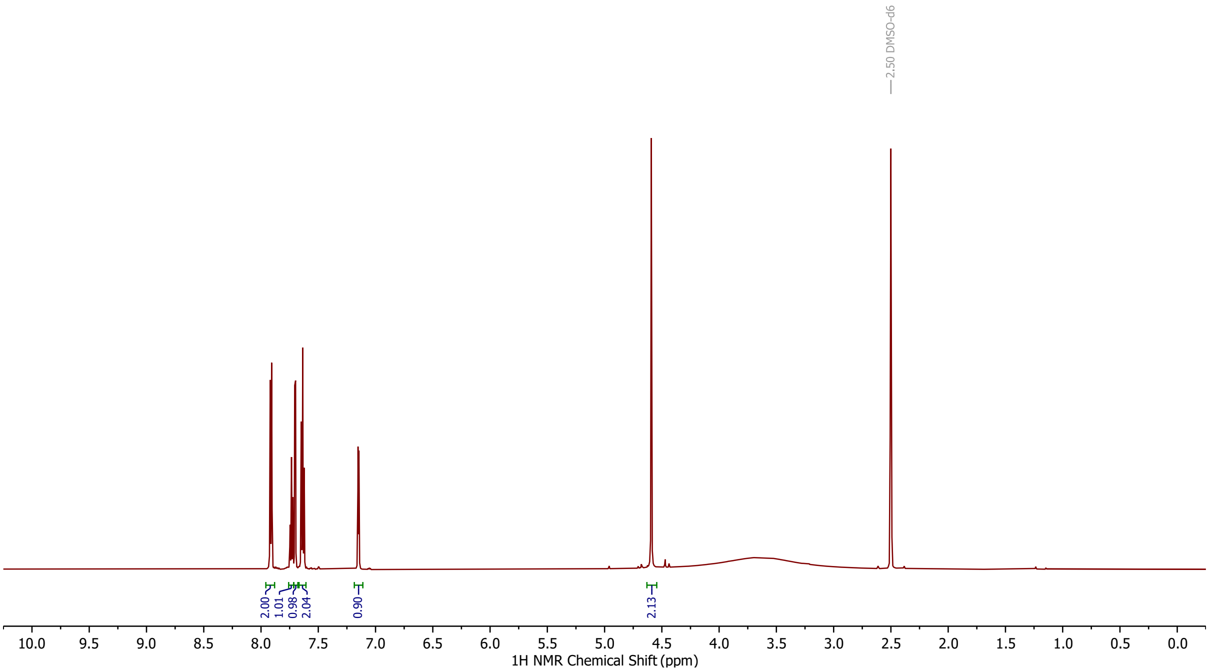

^{13}C NMR (151 MHz, 300 K, DMSO-d_6):

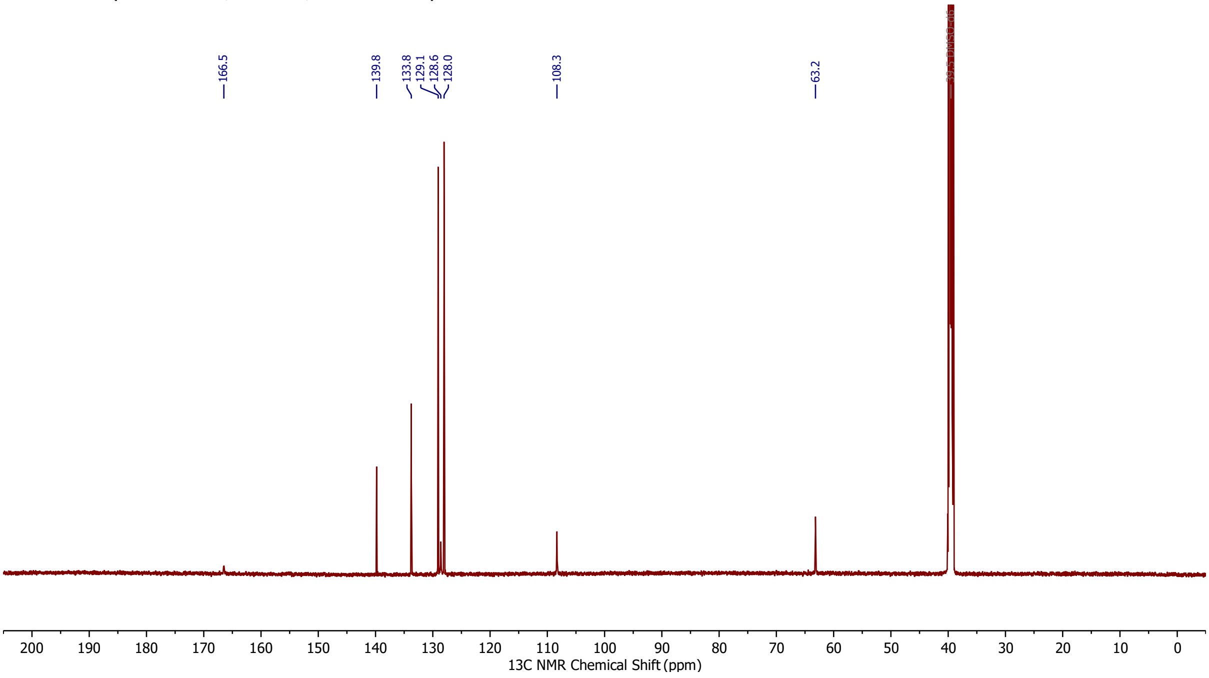

Methyl (*Z*)-2-(3-hydroxy-2-((2-(phenylsulfonyl)acetyl)imino)-2,3-dihydrothiazol-4-yl)acetate (9)

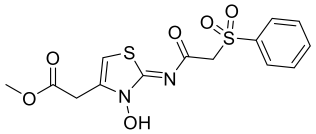

^{1}H NMR (600 MHz, 300 K, $CDCl_3$):

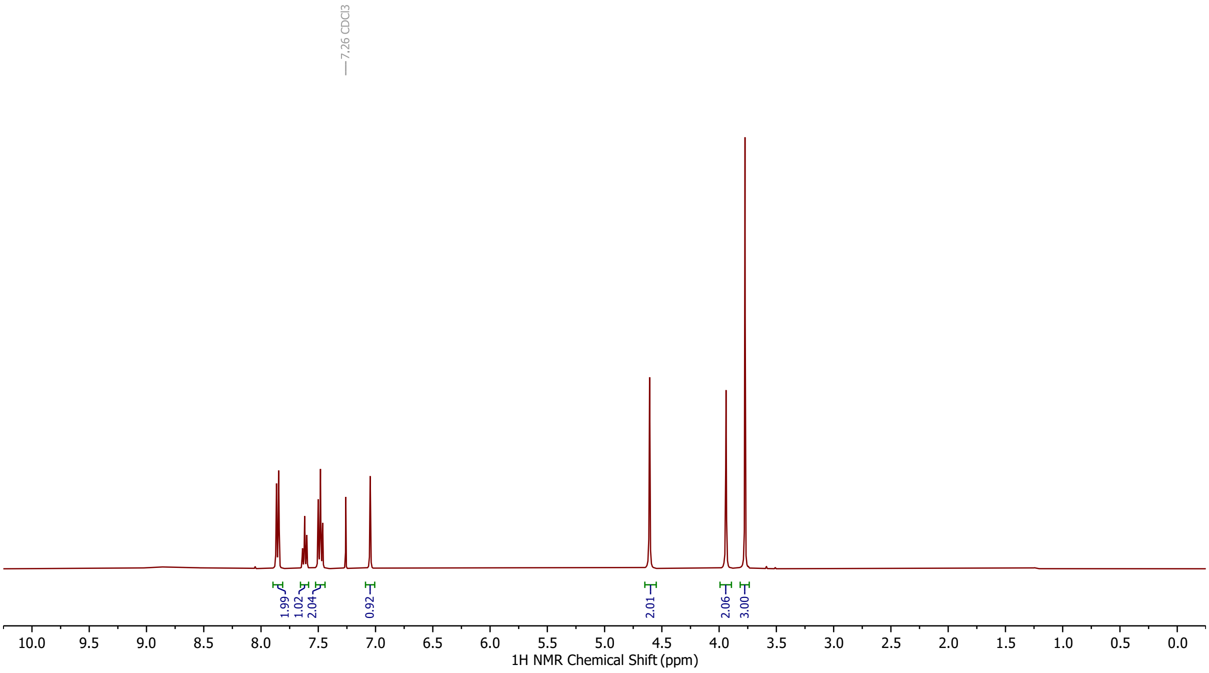

^{13}C NMR (151 MHz, 300 K, $CDCl_3$):

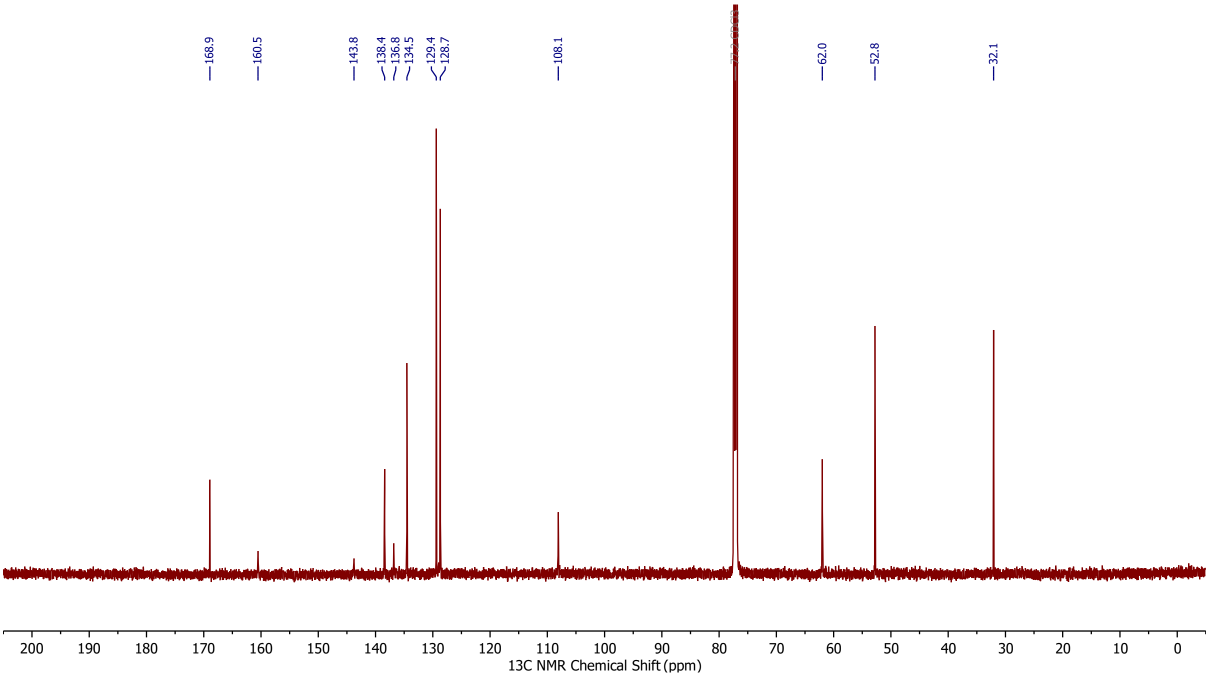

_N_-Methyl-2-(2-(2-(phenylsulfonyl)acetamido)thiazol-4-yl)acetamide (10a)

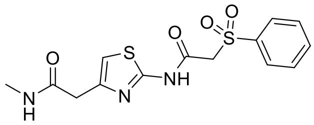

^{1}H NMR (600 MHz, 300 K, DMSO-d_6):

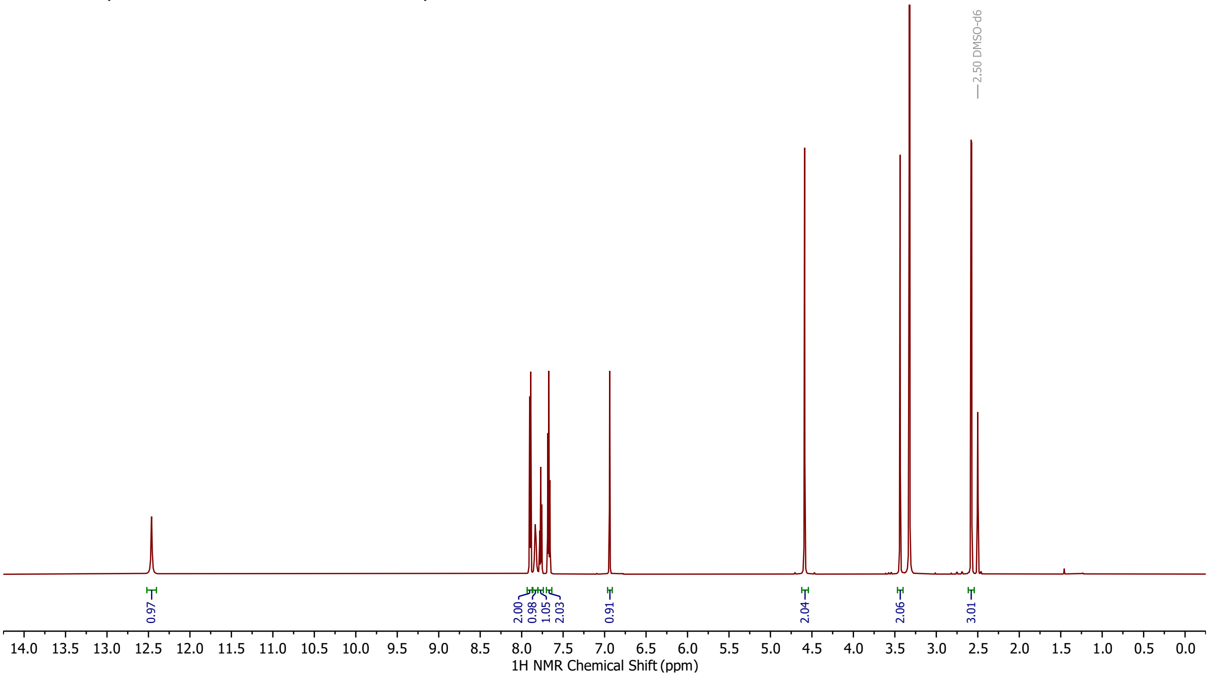

^{13}C NMR (151 MHz, 300 K, DMSO-d_6):

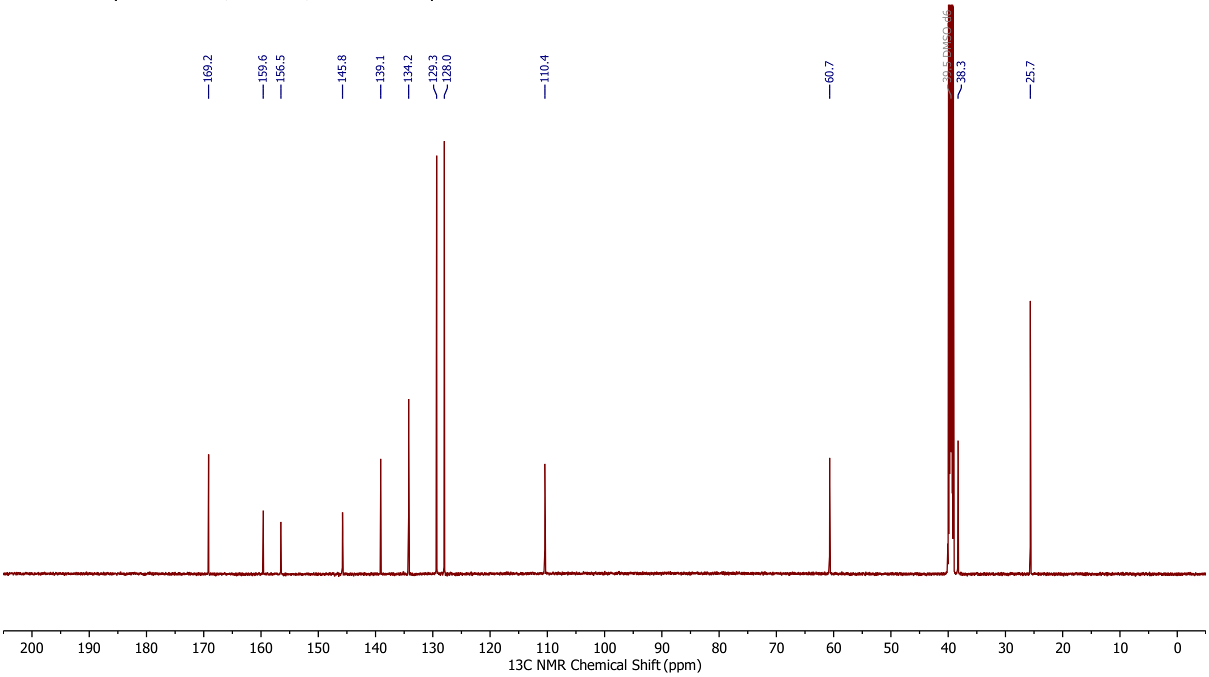

(*Z*)-2-(3-Hydroxy-2-((2-(phenylsulfonyl)acetyl)imino)-2,3-dihydrothiazol-4-yl)-*N*-methylacetamide (10)

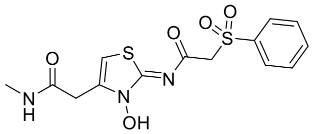

^{1}H NMR (600 MHz, 300 K, DMSO-d_6):

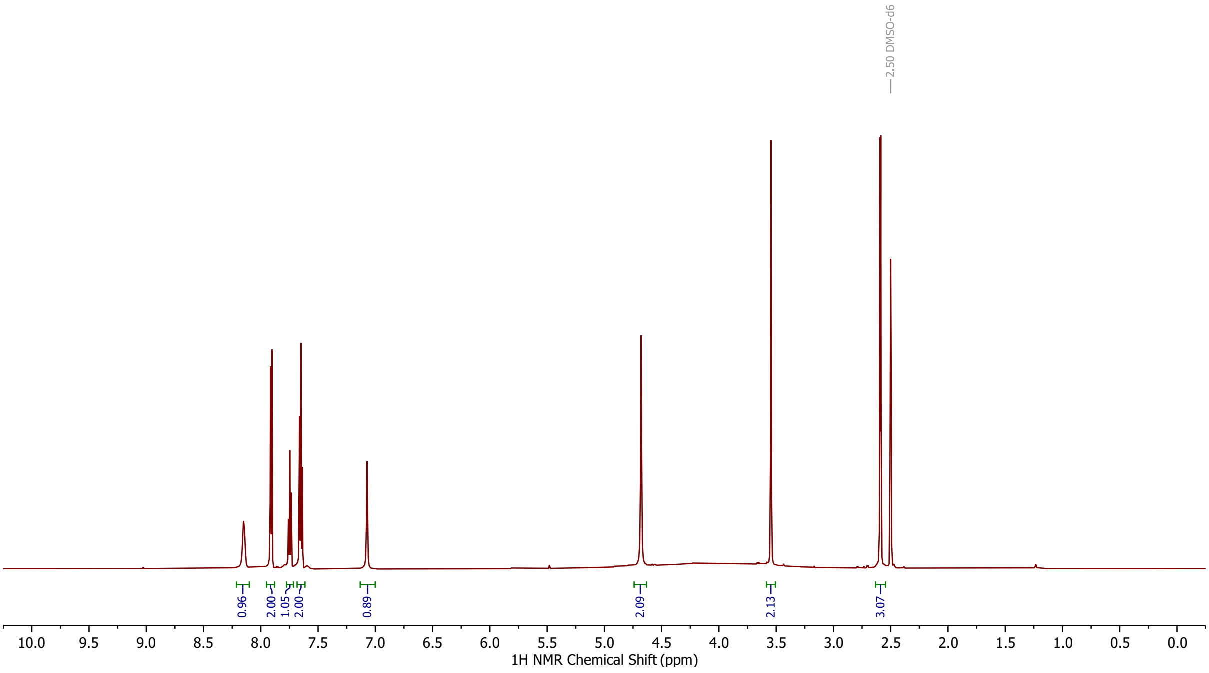

^{13}C NMR (151 MHz, 300 K, DMSO-d_6):

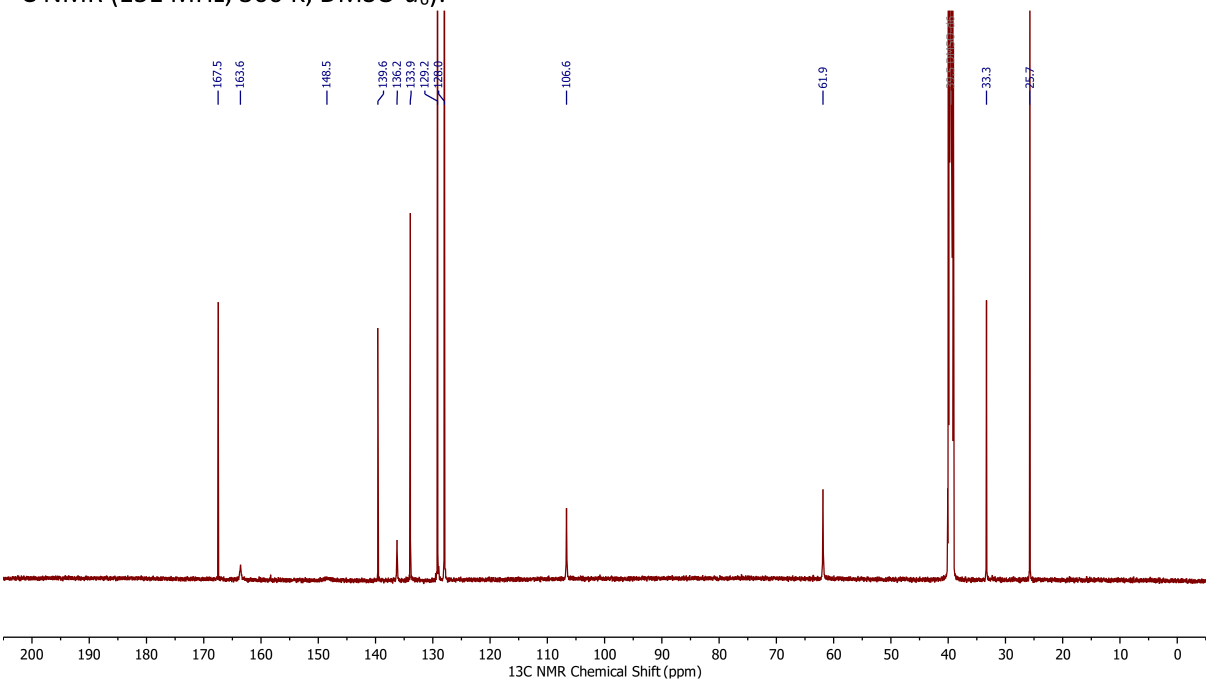

N-(4-(2-Amino-2-oxoethyl)thiazol-2-yl)-2-(phenylsulfonyl)acetamide (11a)

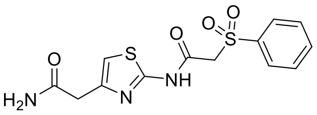

^{1}H NMR (600 MHz, 300 K, DMSO-d_6):

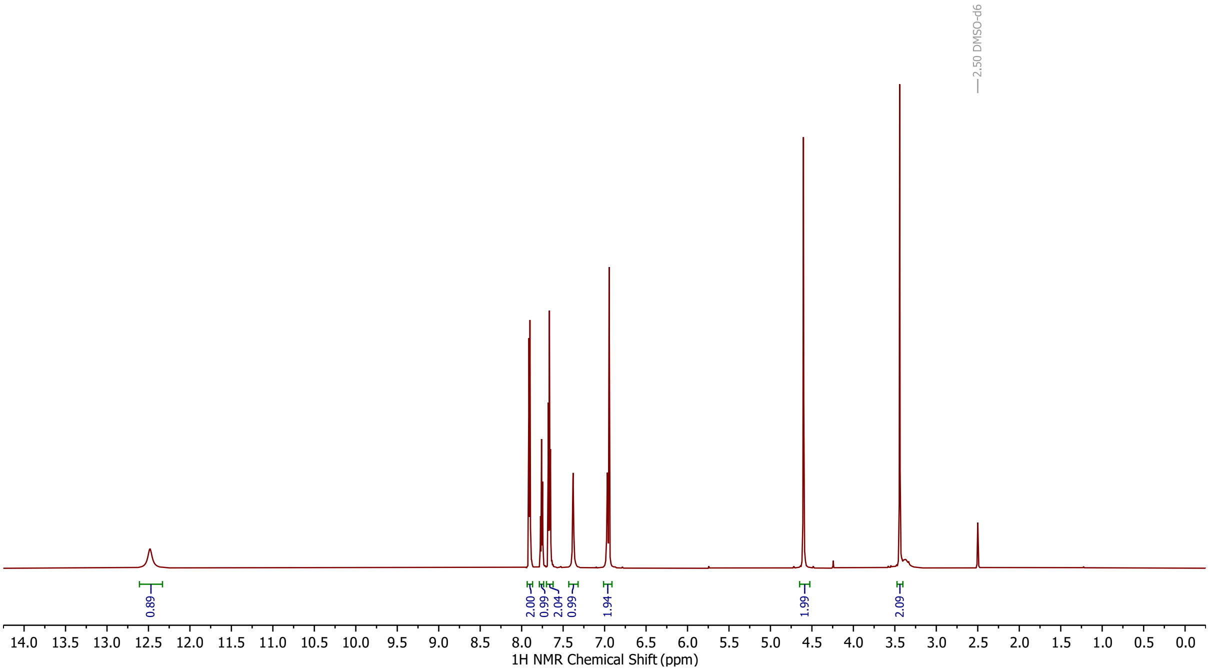

^{13}C NMR (151 MHz, 300 K, DMSO-d_6):

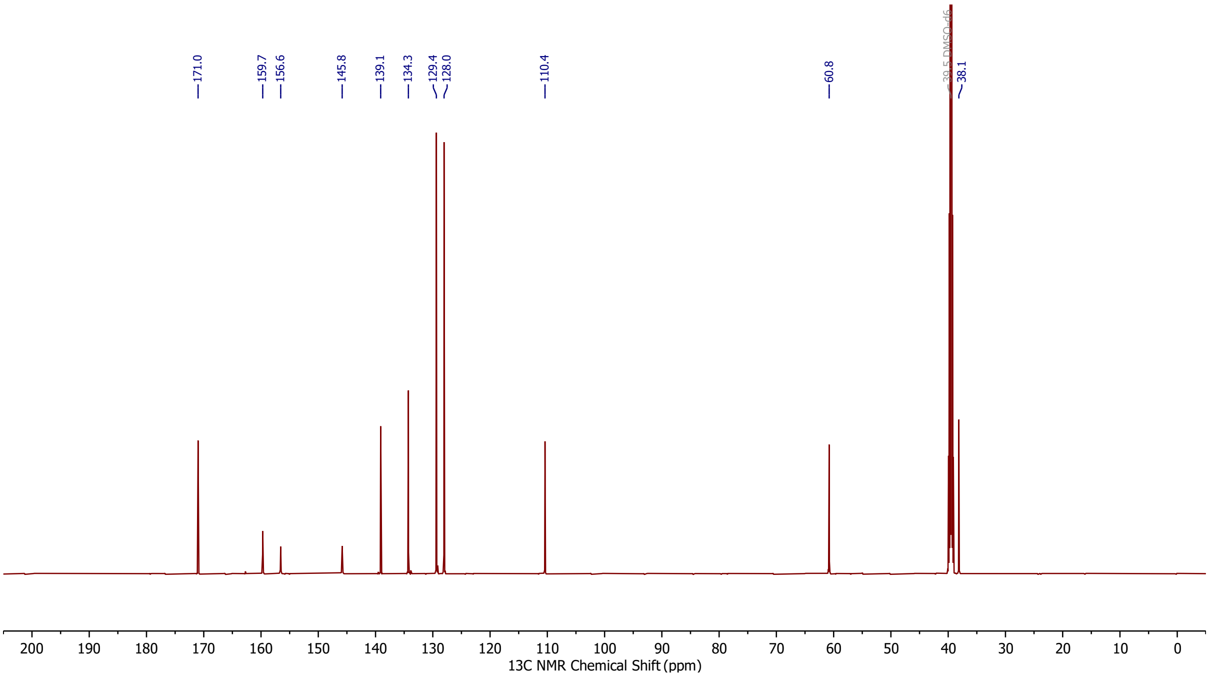

_N_-(4-(Cyanomethyl)thiazol-2-yl)-2-(phenylsulfonyl)acetamide (11b)

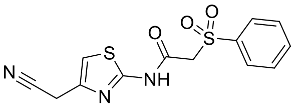

^{1}H NMR (600 MHz, 300 K, DMSO-d_6):

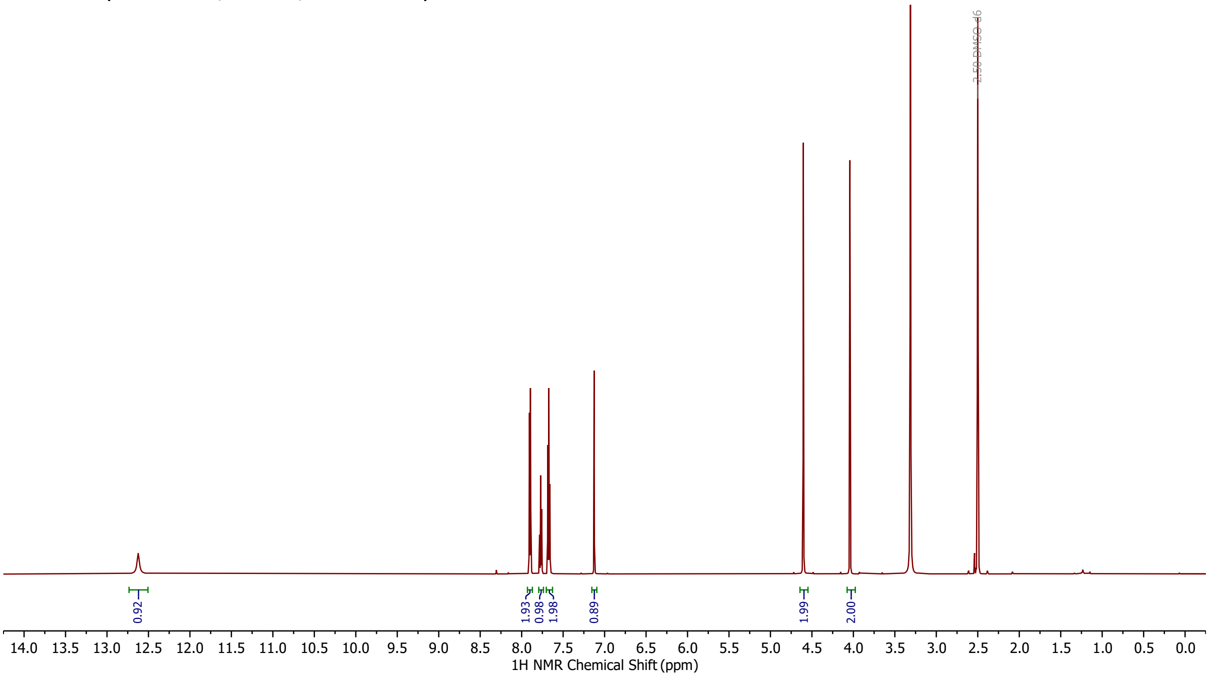

^{13}C NMR (151 MHz, 300 K, DMSO-d_6):

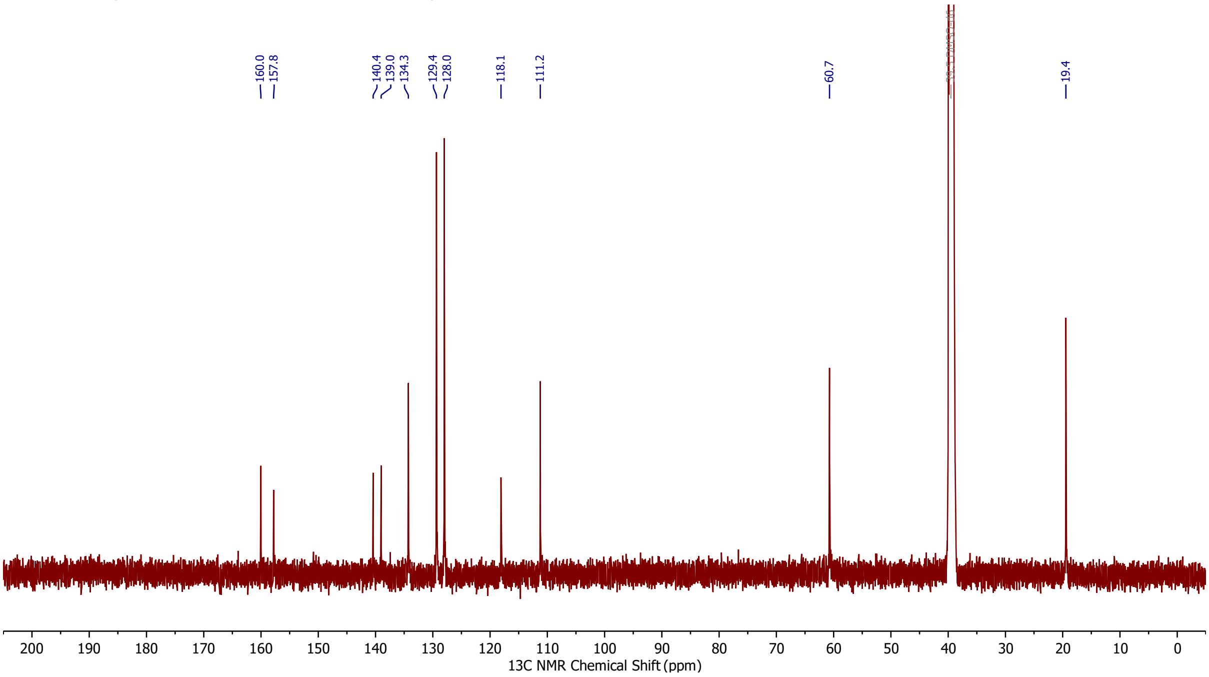

(*Z*)-*N*-(4-(Cyanomethyl)-3-hydroxythiazol-2(3*H*)-ylidene)-2-(phenyl sulfonyl)acetamide (11)

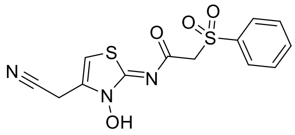

^{1}H NMR (600 MHz, 300 K, DMSO-d_6):

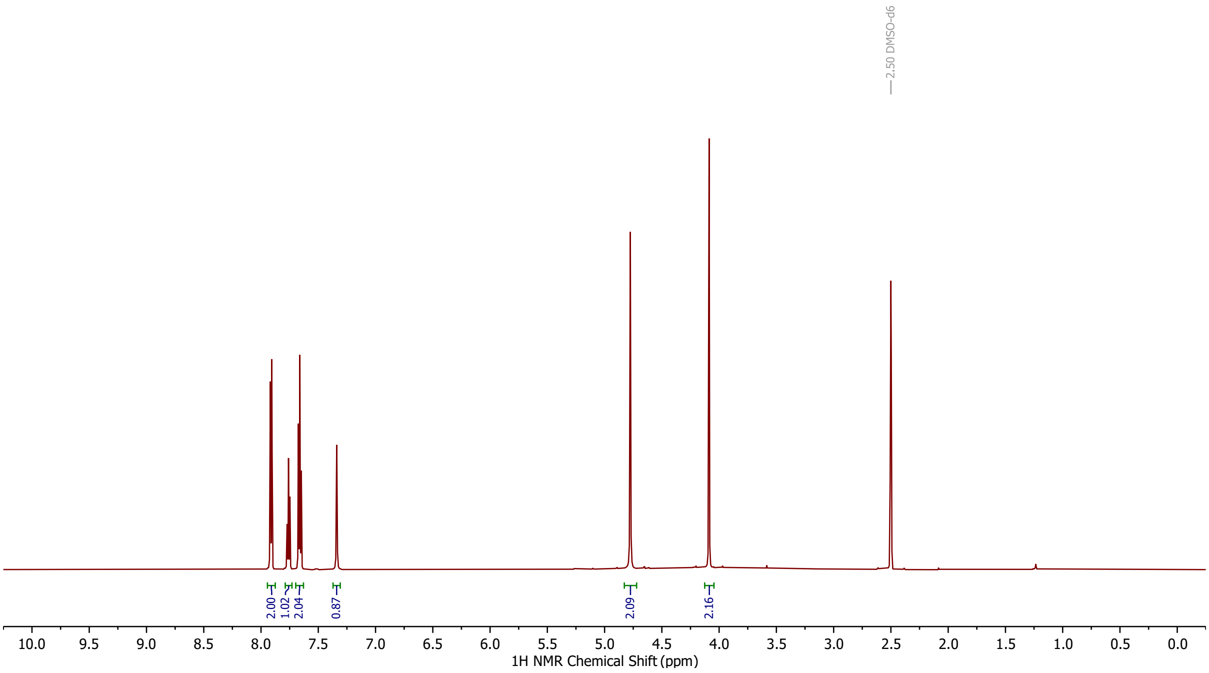

^{13}C NMR (151 MHz, 300 K, DMSO-d_6):

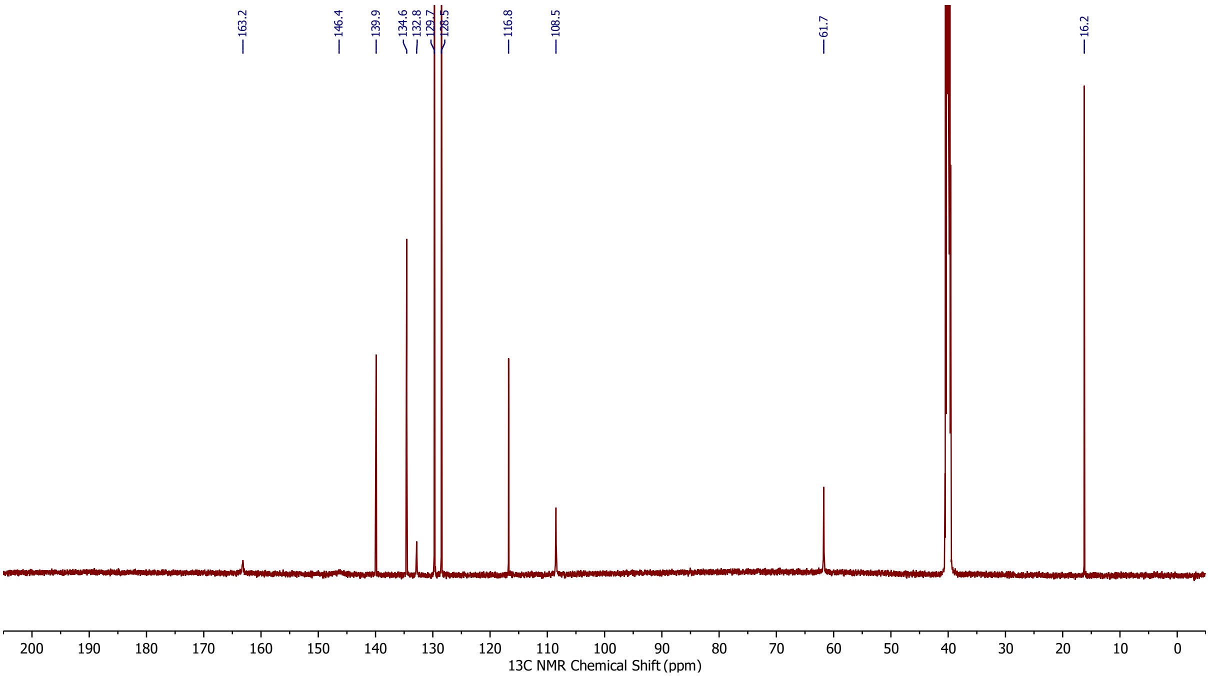

***N*-(4-(Chloromethyl)thiazol-2-yl)-2-(phenylsulfonyl)acetamide (12b)**

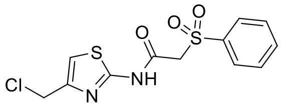

^{1}H NMR (600 MHz, 300 K, $CDCl_3$):

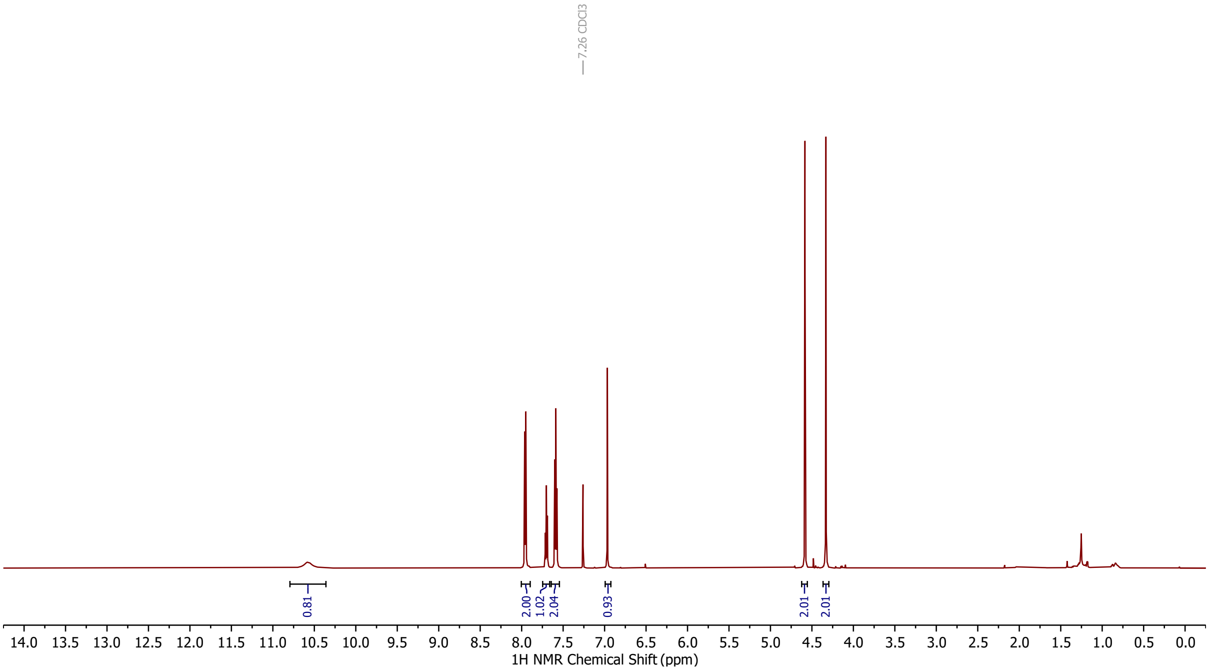

^{13}C NMR (151 MHz, 300 K, $CDCl_3$):

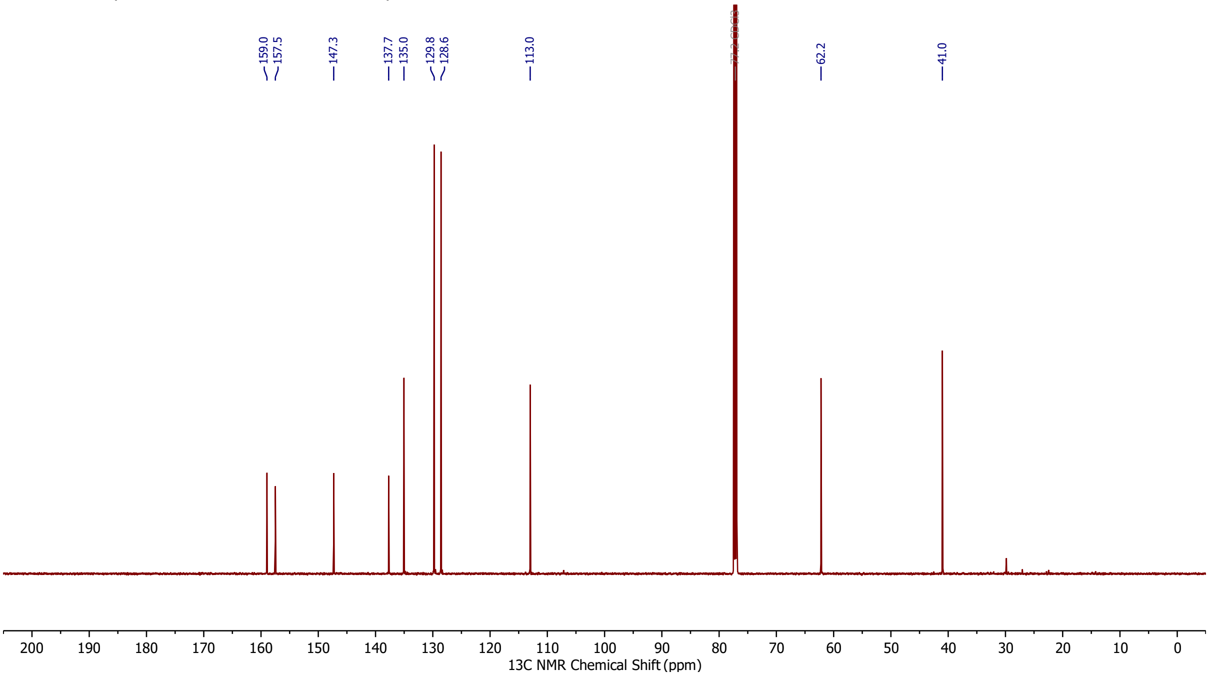

_N_-(4-((1_H_-1,2,3-Triazol-1-yl)methyl)thiazol-2-yl)-2-(phenylsulfonyl) acetamide (12c)

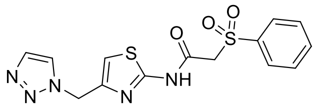

^{1}H NMR (600 MHz, 300 K, DMSO-d_6):

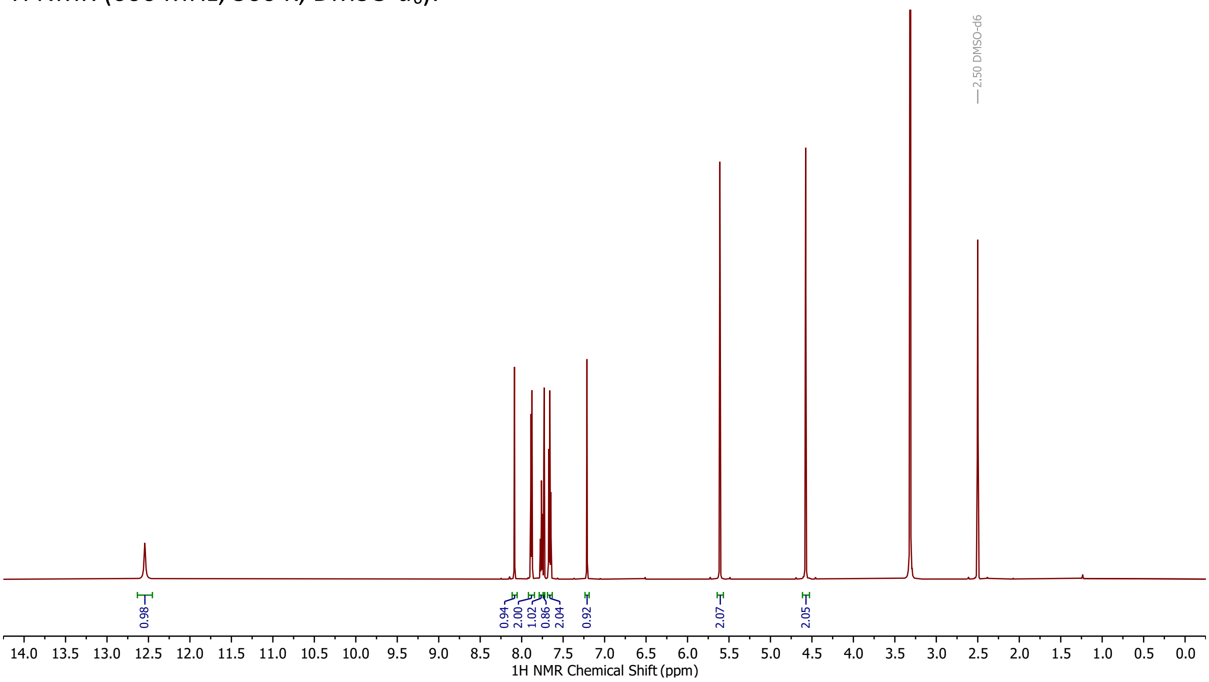

^{13}C NMR (151 MHz, 300 K, DMSO-d_6):

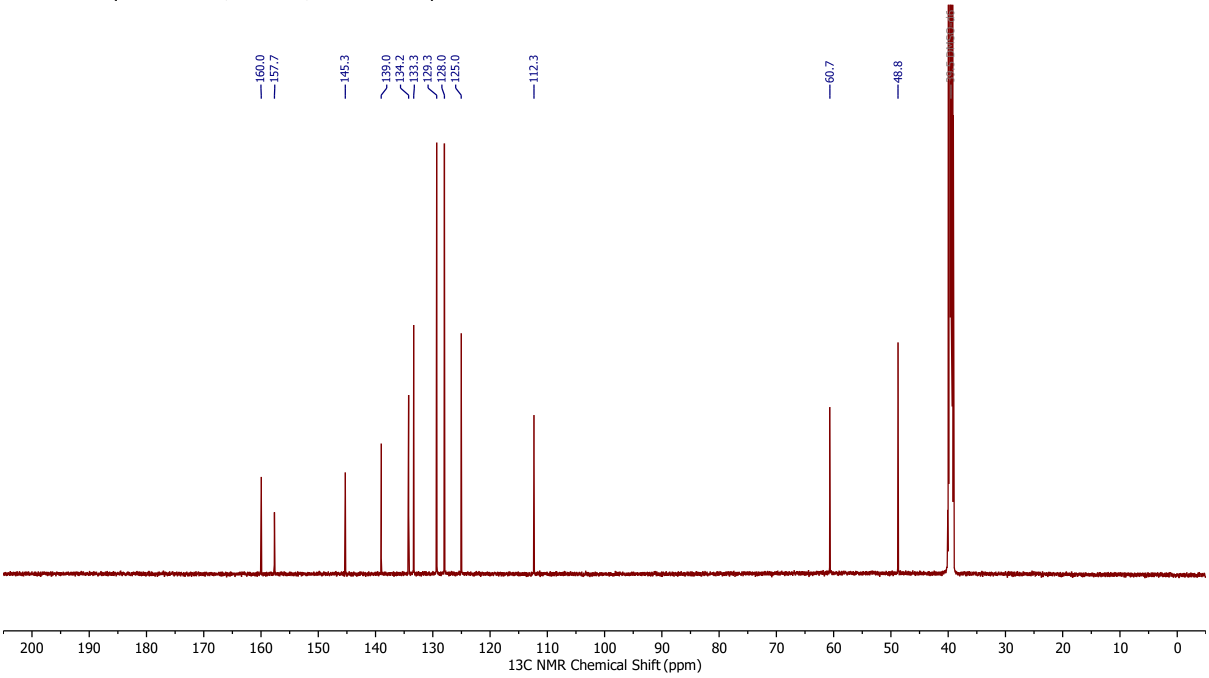

(*Z*)-*N*-(4-((1*H*-1,2,3-Triazol-1-yl)methyl)-3-hydroxythiazol-2(3*H*)-ylidene)-2-(phenylsulfonyl)acetamide (12)

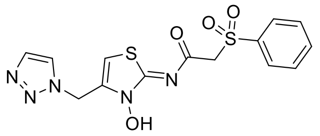

^{1}H NMR (600 MHz, 300 K, DMSO-d_6):

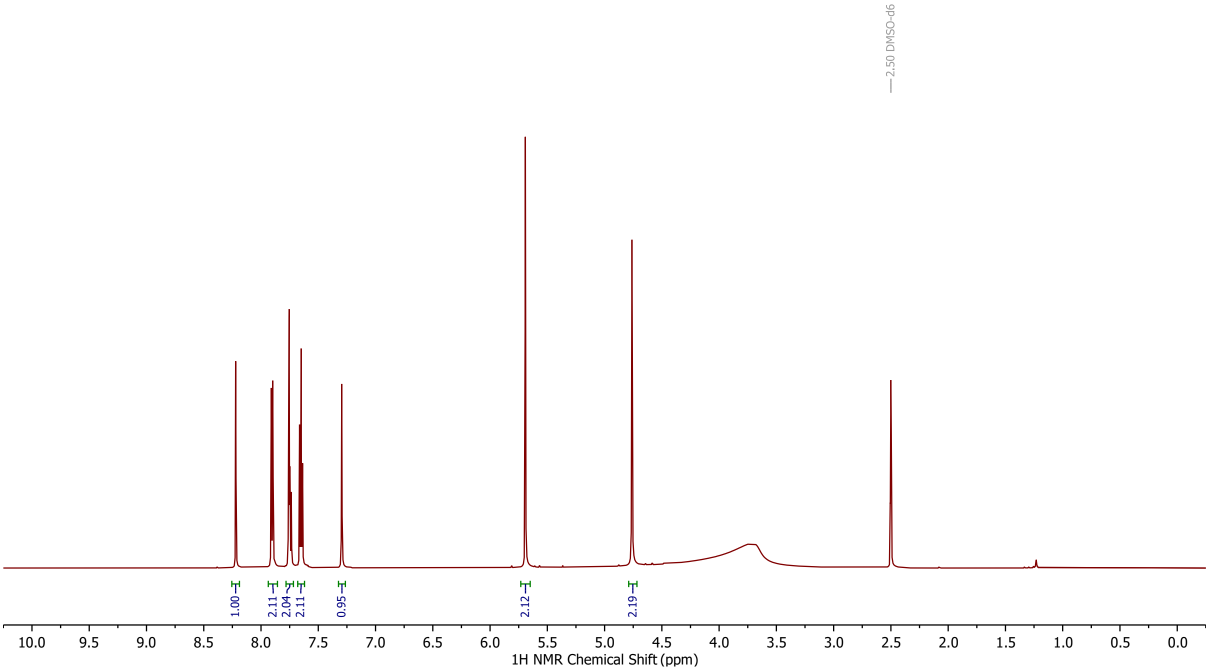

^{13}C NMR (151 MHz, 300 K, DMSO-d_6):

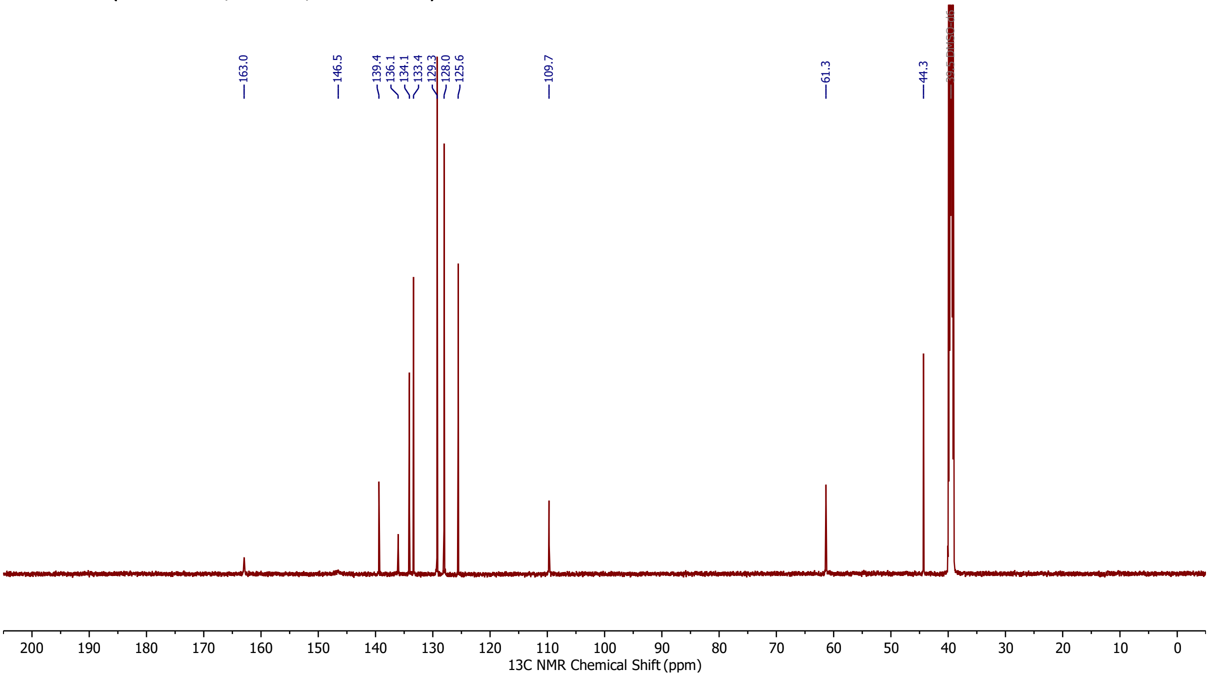

Methyl 3-(2-(2-(phenylsulfonyl)acetamido)thiazol-4-yl)propanoate (13c)

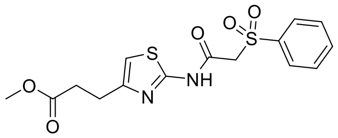

^{1}H NMR (600 MHz, 300 K, $CDCl_3$):

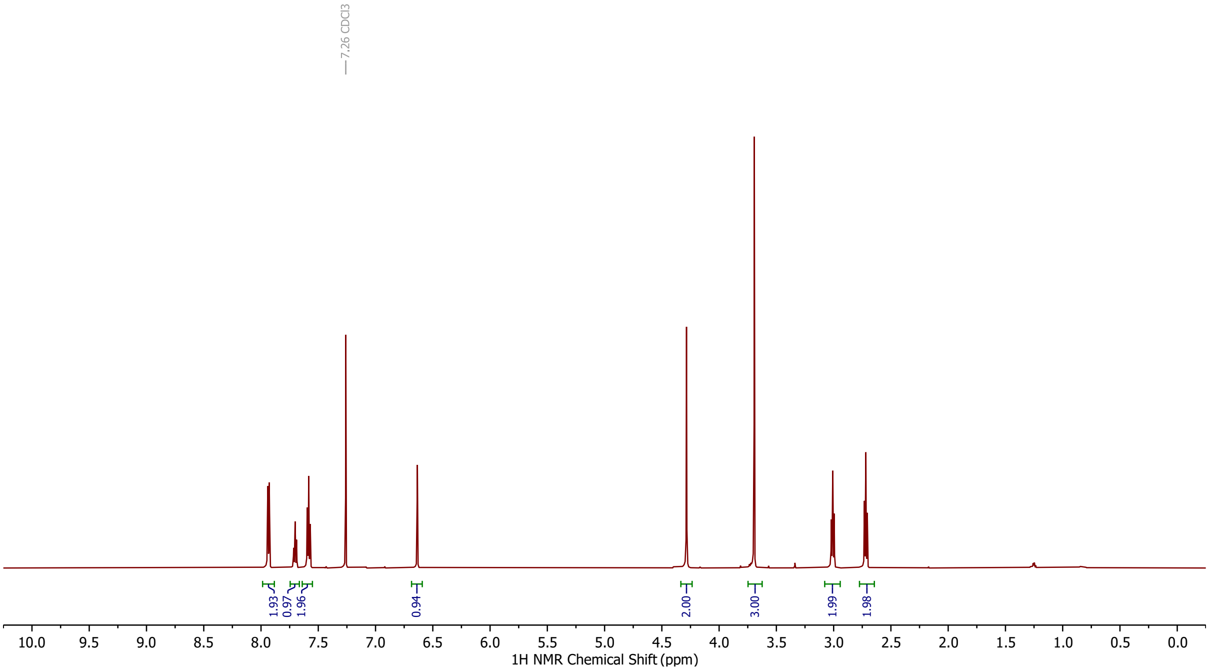

^{13}C NMR (151 MHz, 300 K, $CDCl_3$):

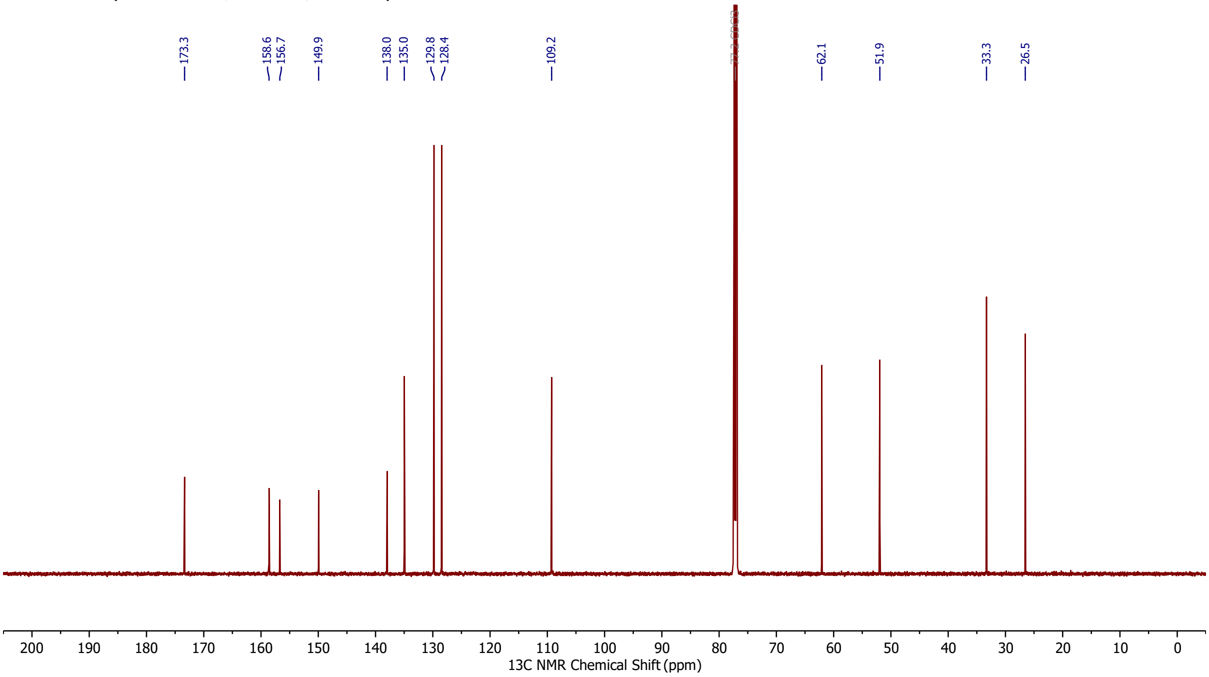

Methyl (*Z*)-3-(3-hydroxy-2-((2-(phenylsulfonyl)acetyl)imino)-2,3-dihydrothiazol-4-yl)propanoate (13d)

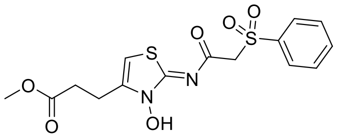

^{1}H NMR (600 MHz, 300 K, $CDCl_3$):

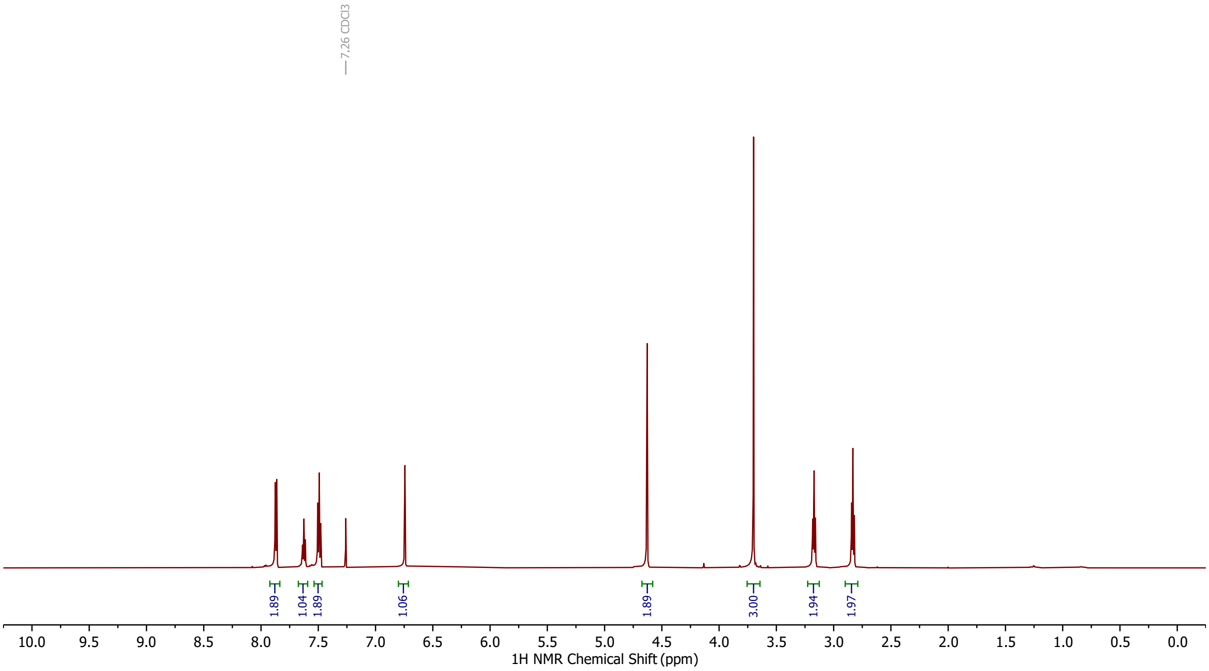

^{13}C NMR (151 MHz, 300 K, $CDCl_3$):

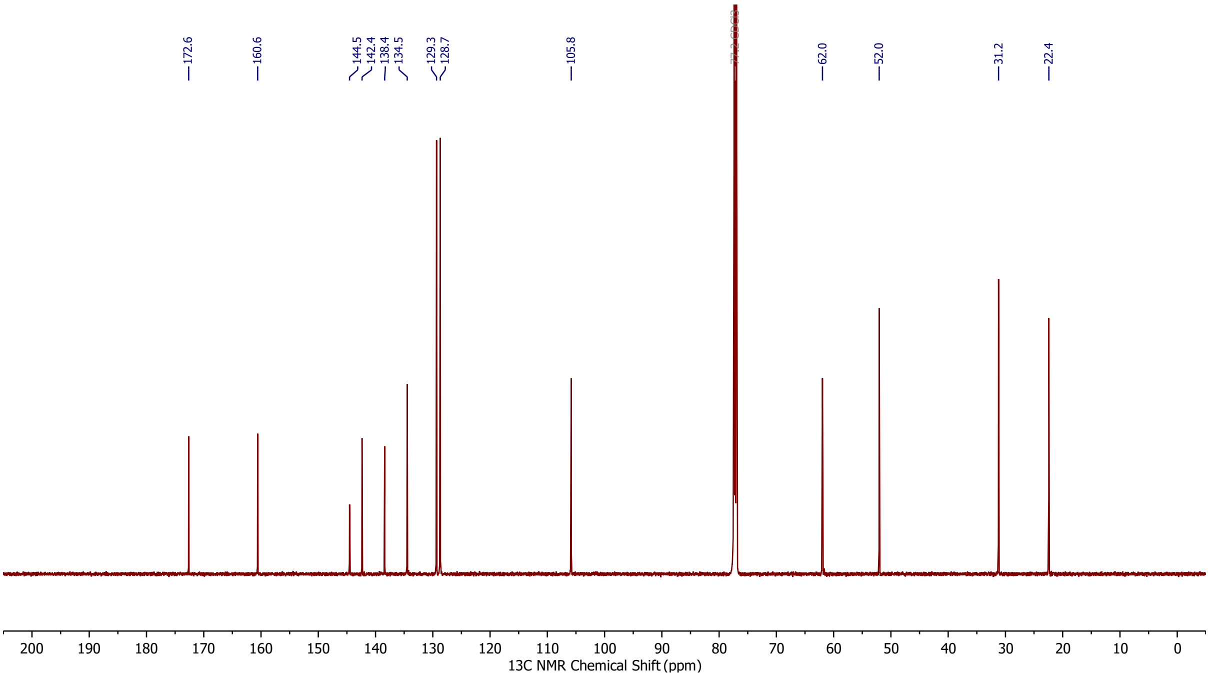

(*Z*)-3-(3-Hydroxy-2-((2-(phenylsulfonyl)acetyl)imino)-2,3-dihydrothiazol-4-yl)propanoic acid (13)

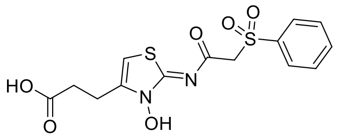

^{1}H NMR (600 MHz, 300 K, DMSO-d_6):

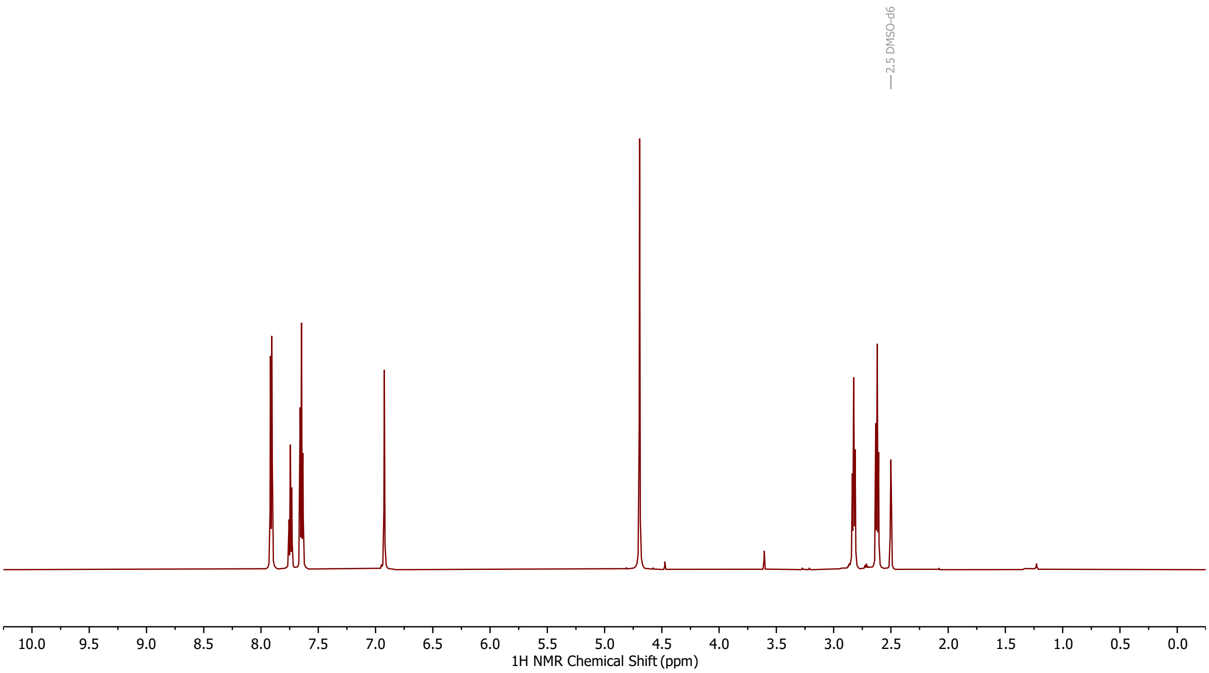

^{13}C NMR (151 MHz, 300 K, DMSO-d_6):

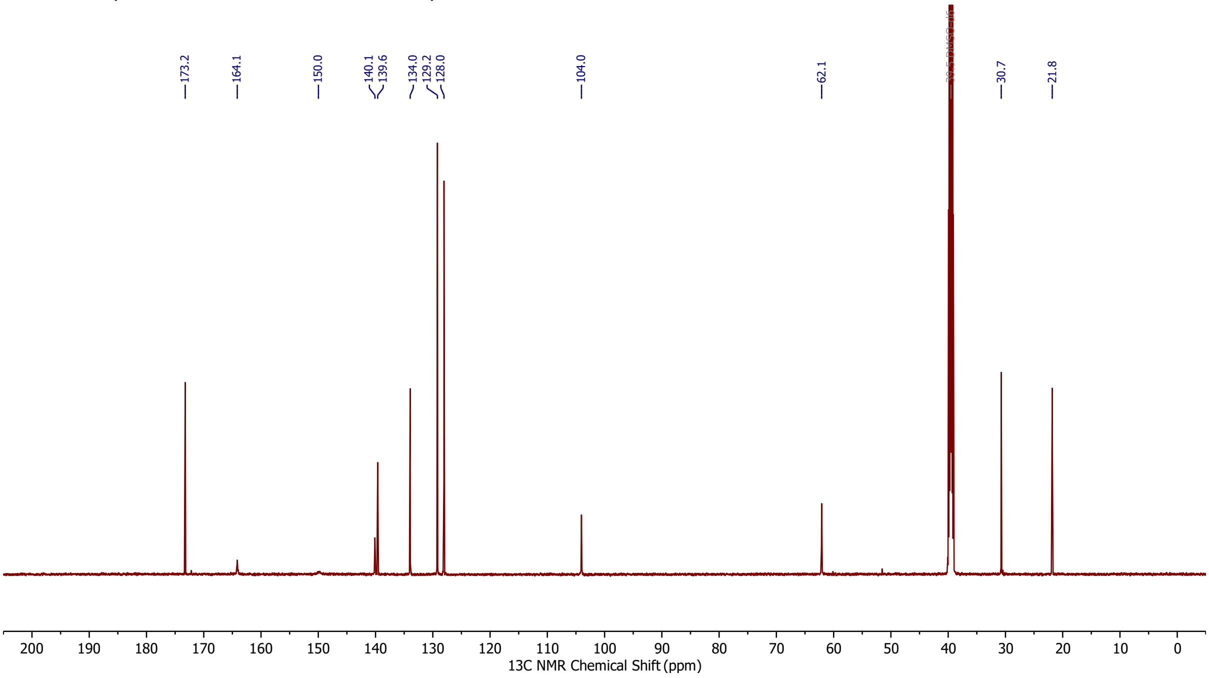

Ethyl (*Z*)-2-(2-(acetylimino)-3-hydroxy-2,3-dihydrothiazol-4-yl)acetate (14b)

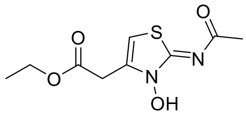

^{1}H NMR (600 MHz, 300 K, $CDCl_3$):

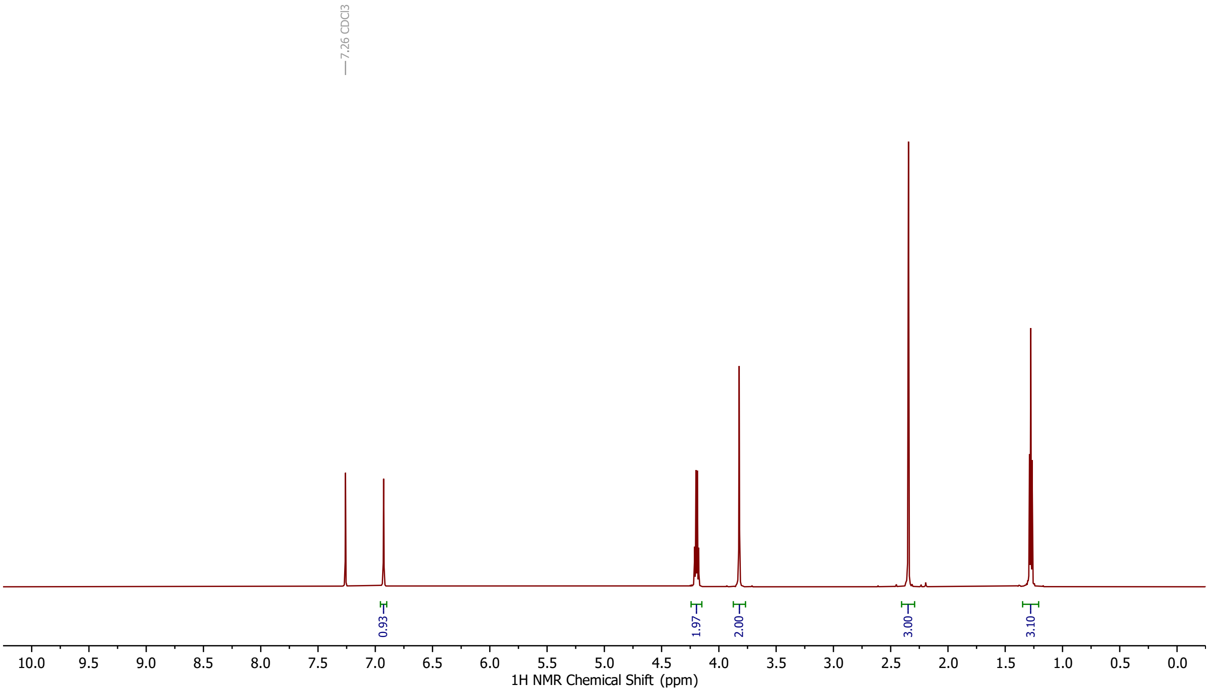

^{13}C NMR (151 MHz, 300 K, $CDCl_3$):

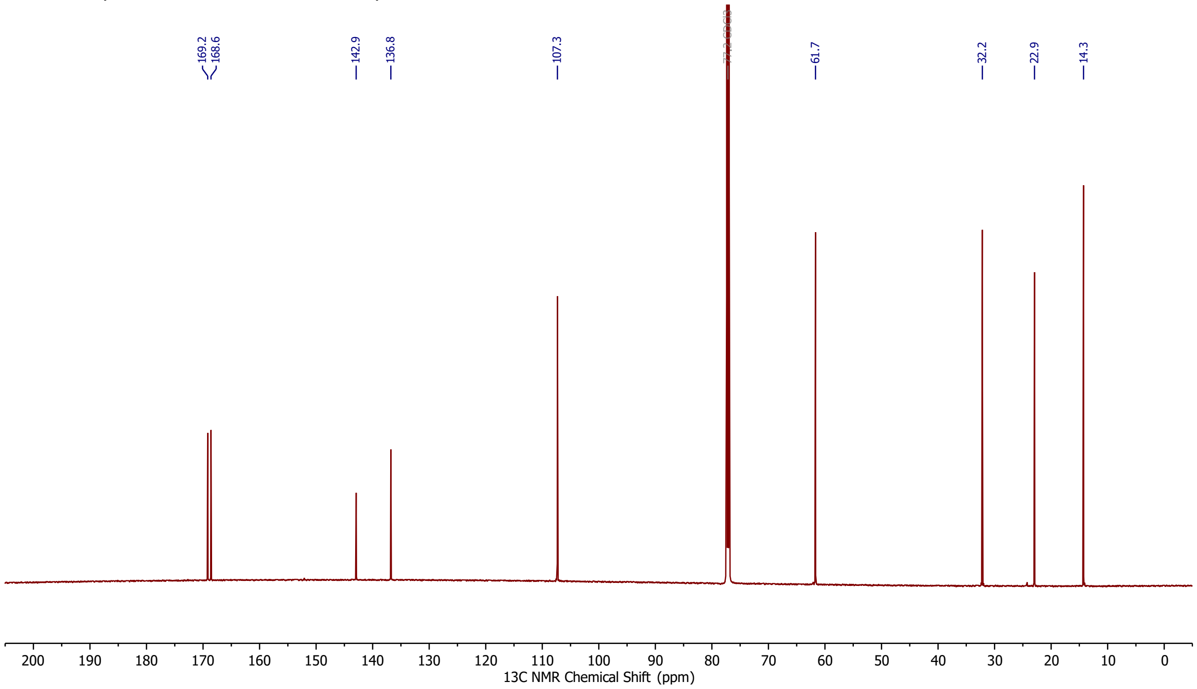

(*Z*)-2-(2-(Acetylimino)-3-hydroxy-2,3-dihydrothiazol-4-yl)acetic acid (14)

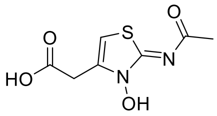

^{1}H NMR (600 MHz, 300 K, DMSO-d_6):

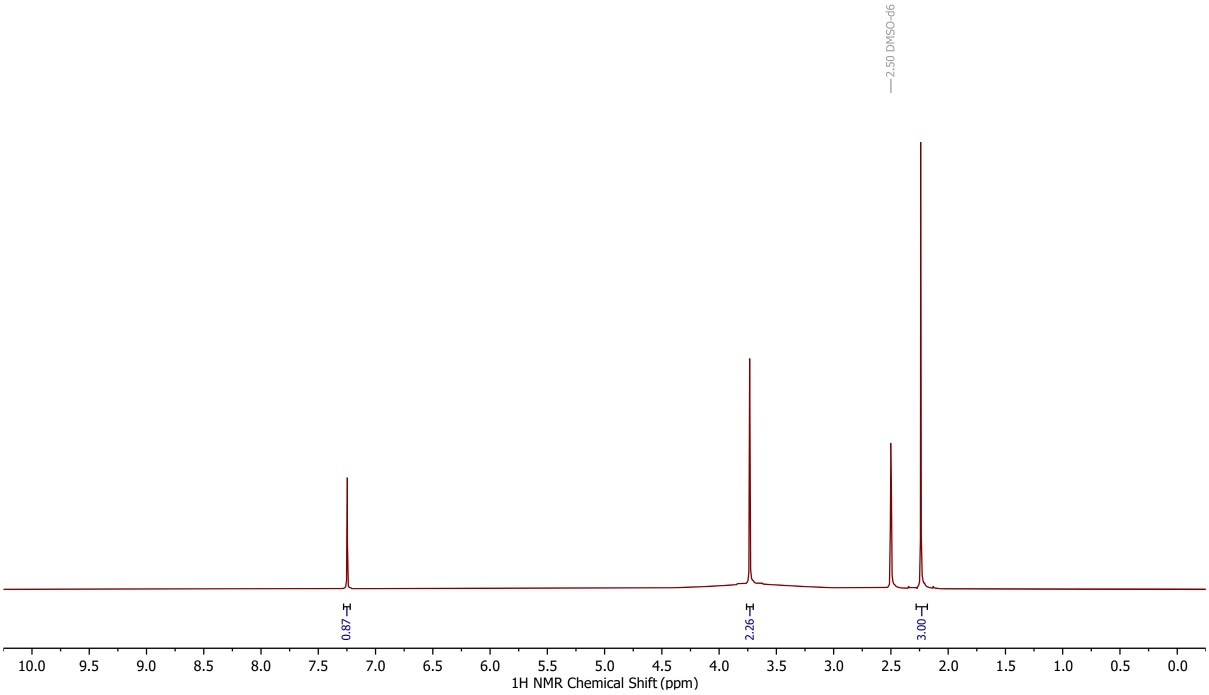

^{13}C NMR (151 MHz, 300 K, DMSO-d_6):

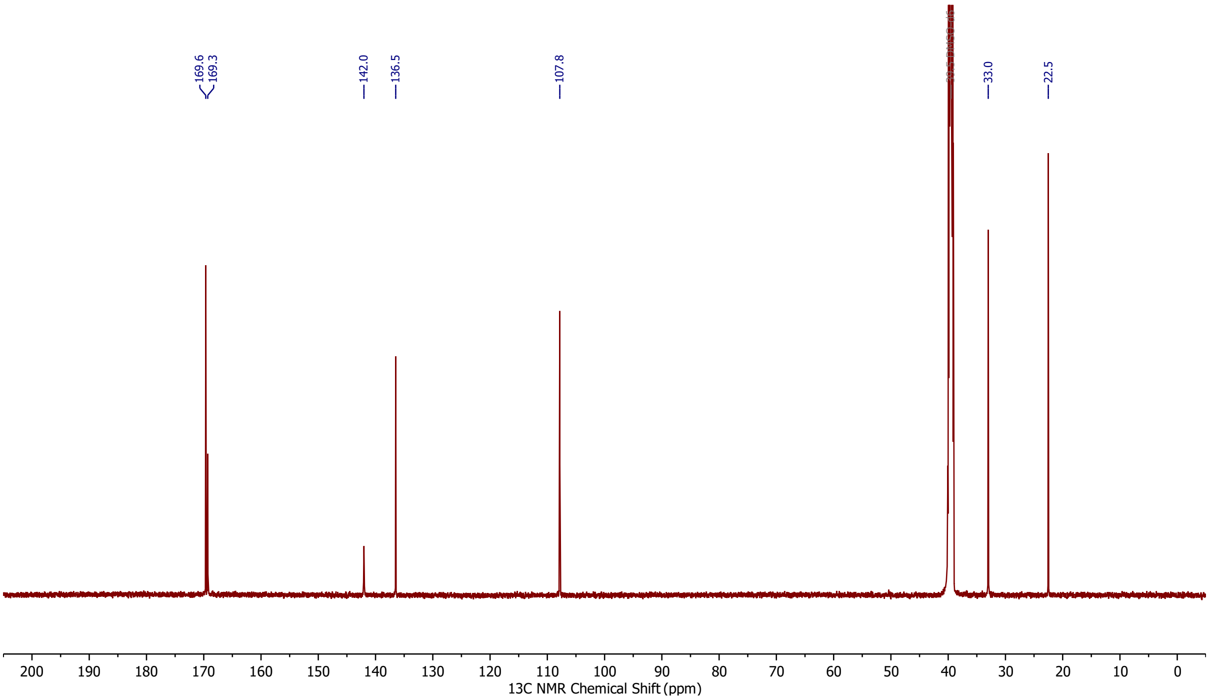

Ethyl 2-(2-(3-(phenylsulfonyl)propanamido)thiazol-4-yl)acetate (15a)

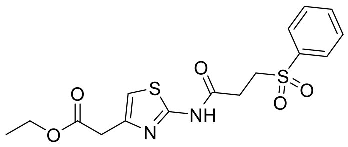

^{1}H NMR (600 MHz, 300 K, $CDCl_3$):

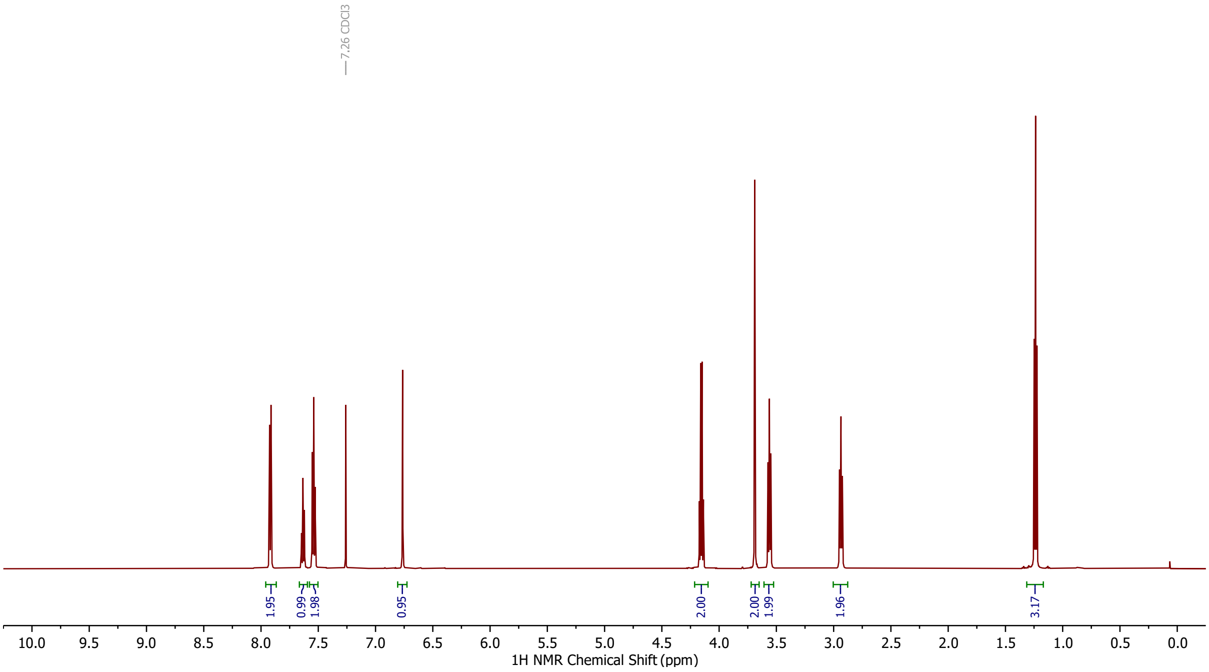

^{13}C NMR (151 MHz, 300 K, $CDCl_3$):

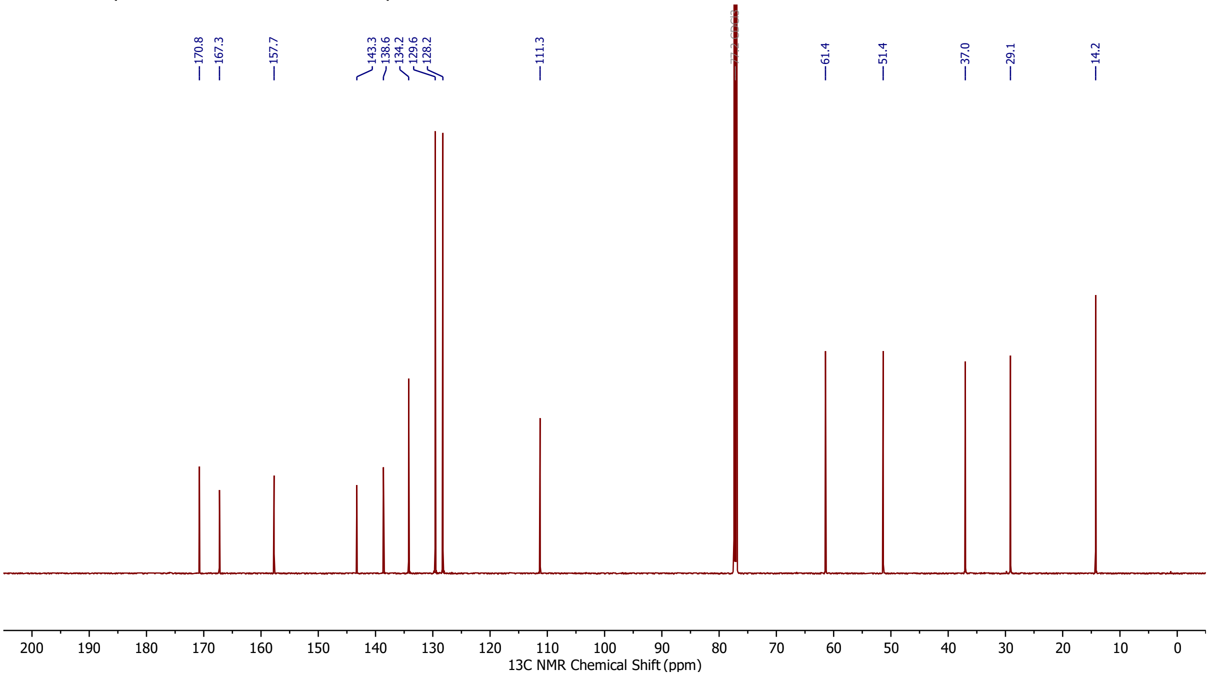

Ethyl (*Z*)-2-(3-hydroxy-2-((3-(phenylsulfonyl)propanoyl)imino)-2,3-dihydrothiazol-4-yl)acetate (15b)

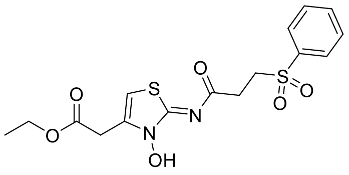

^{1}H NMR (600 MHz, 300 K, DMSO-d_6):

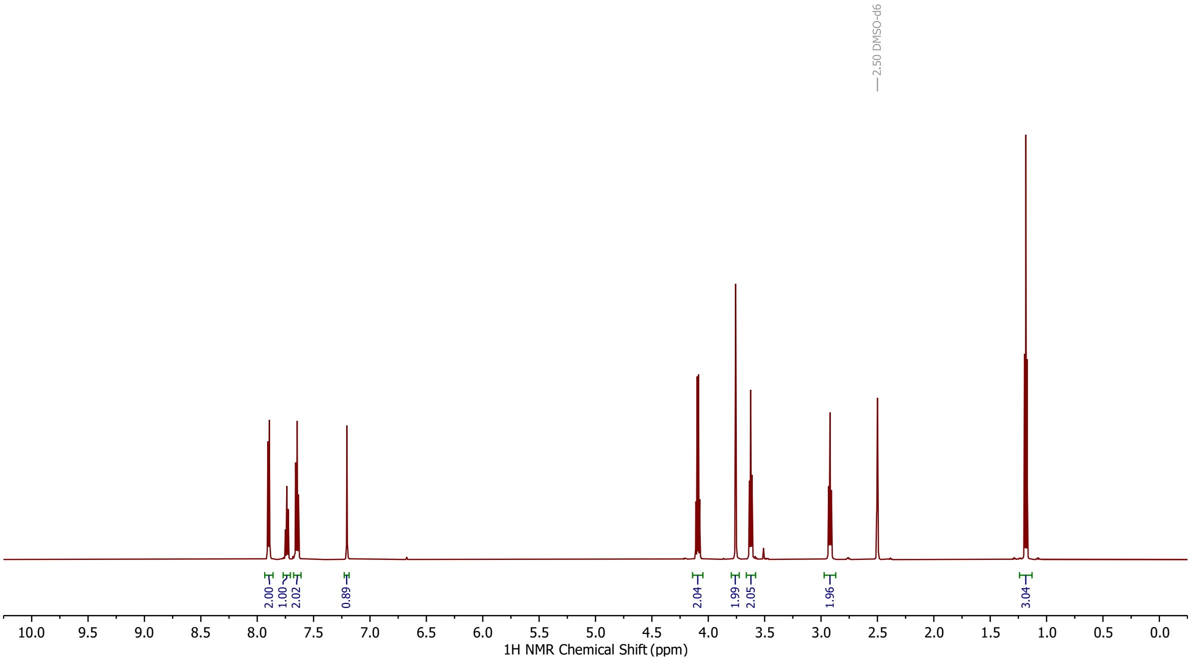

^{13}C NMR (151 MHz, 300 K, DMSO-d_6):

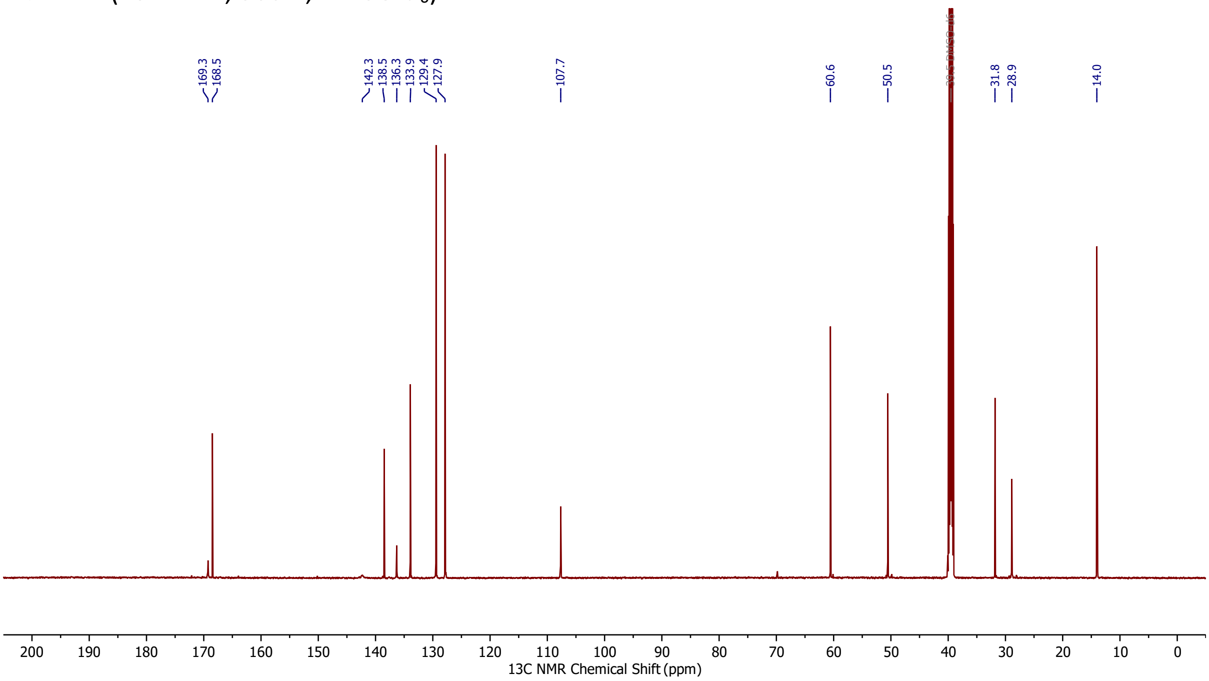

(*Z*)-2-(3-Hydroxy-2-((3-(phenylsulfonyl)propanoyl)imino)-2,3-dihydrothiazol-4-yl)acetic acid (15)

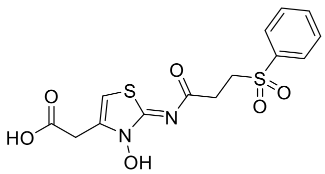

^{1}H NMR (600 MHz, 300 K, DMSO-d_6):

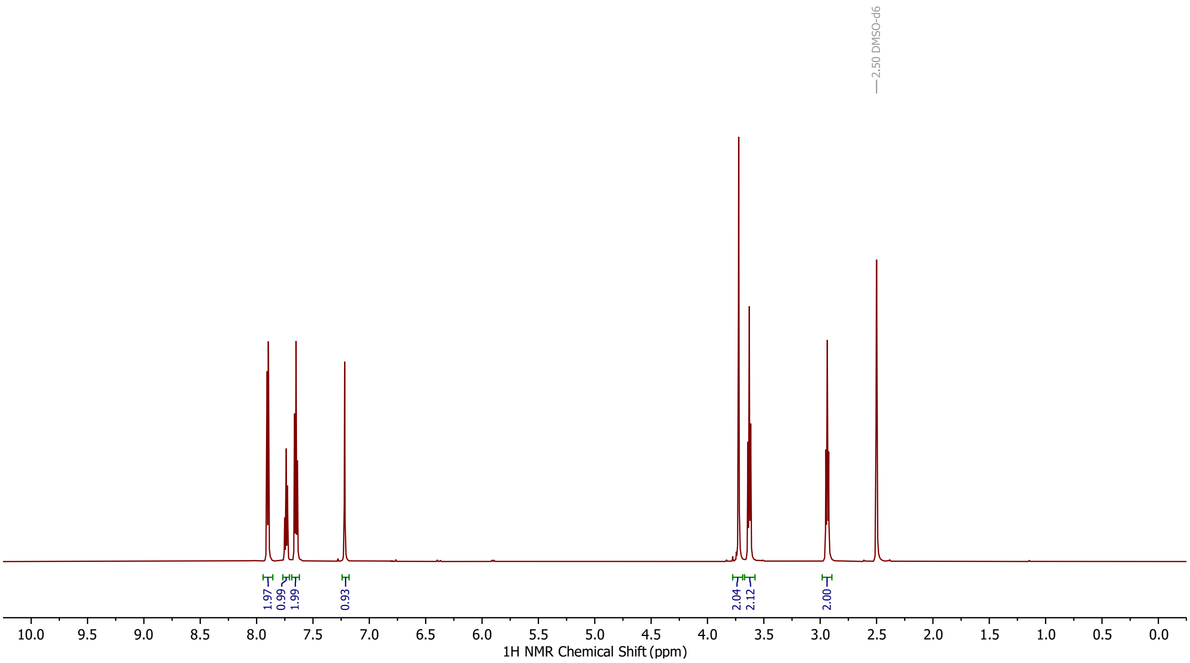

^{13}C NMR (151 MHz, 300 K, DMSO-d_6):

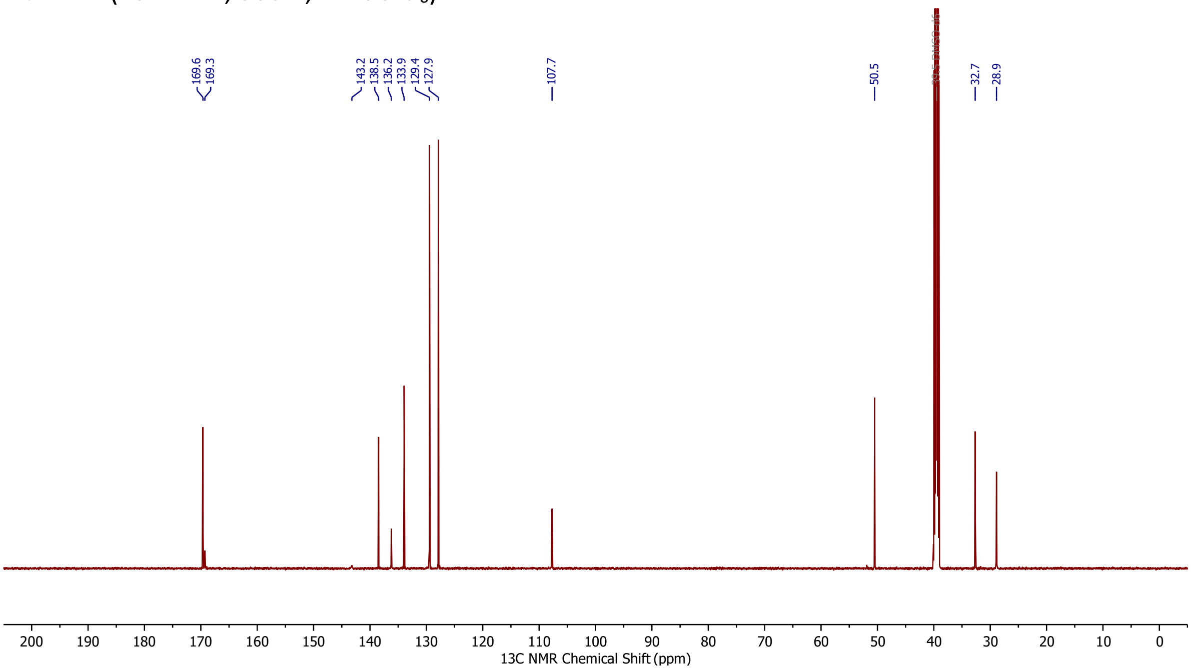

Ethyl 2-(2-(2-aminoacetamido)thiazol-4-yl)acetate (16b)

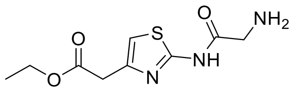

^{1}H NMR (600 MHz, 300 K, CD_3OD):

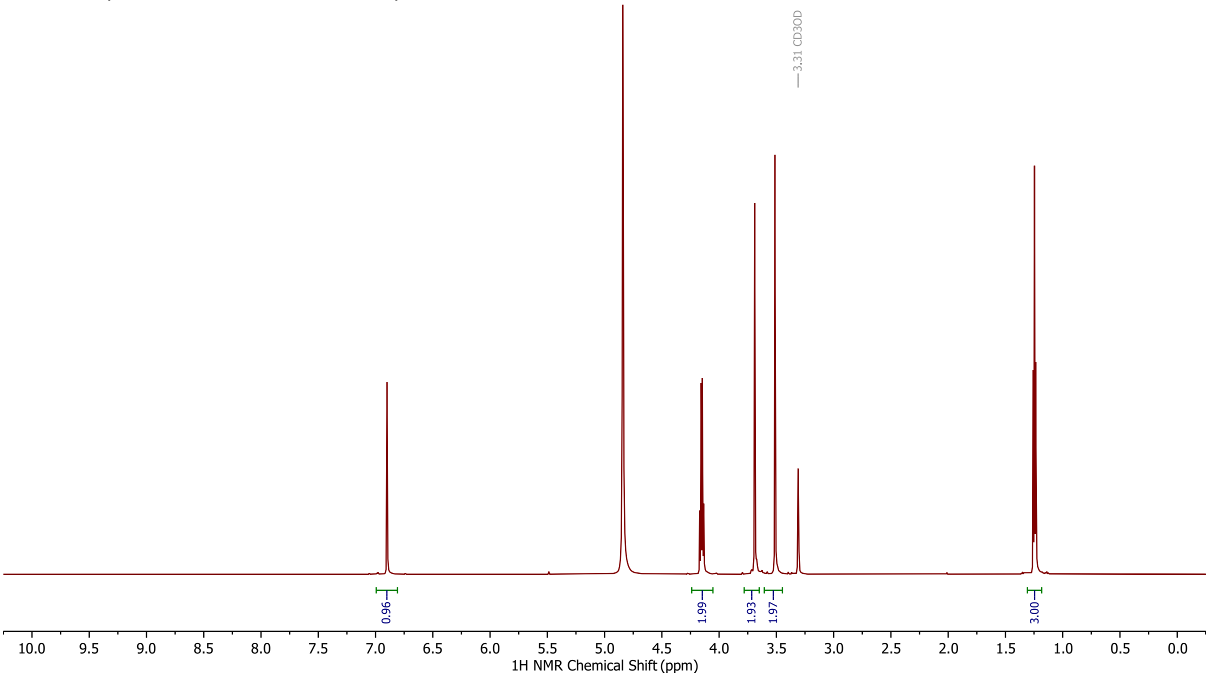

^{13}C NMR (151 MHz, 300 K, CD_3OD):

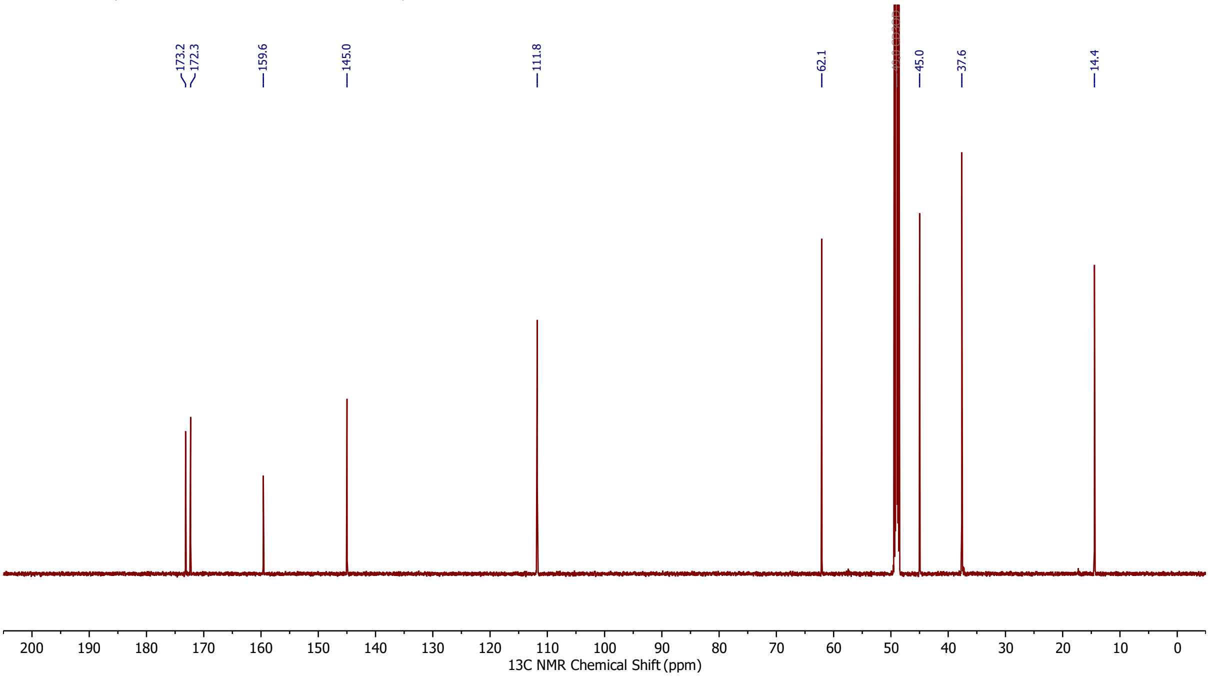

Ethyl 2-(2-(2-benzamidoacetamdo)thiazol-4-yl)acetate (16c)

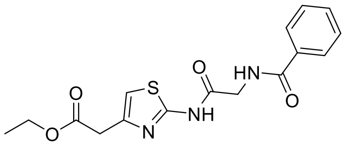

^{1}H NMR (600 MHz, 300 K, DMSO-d_6):

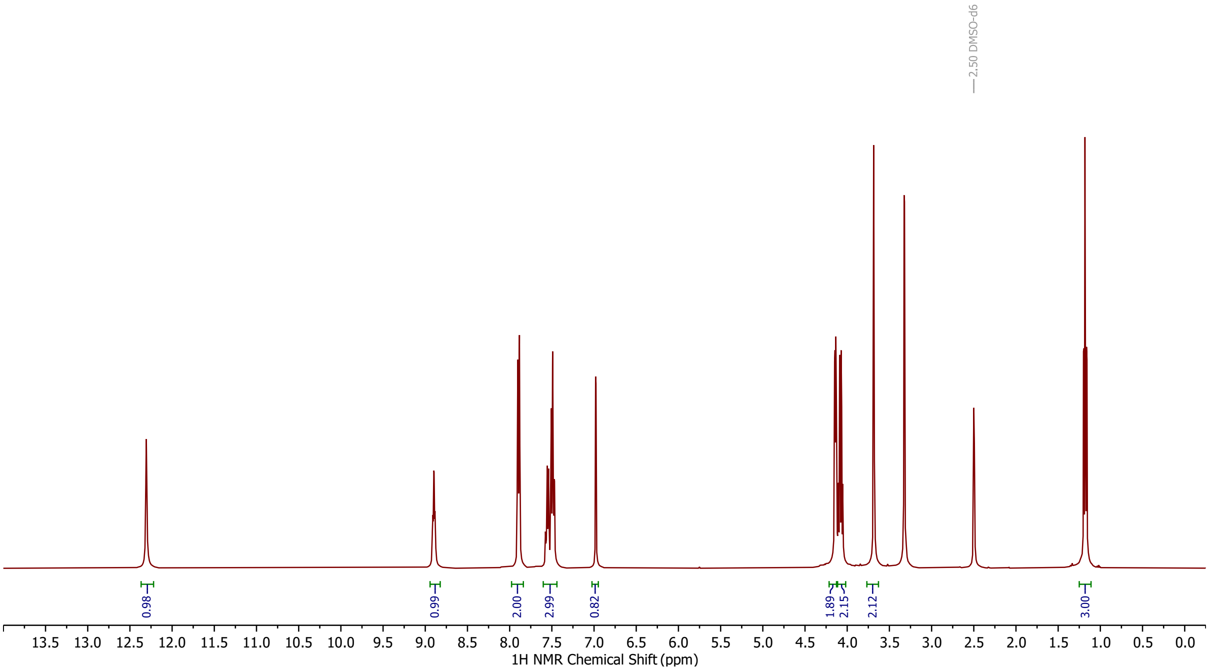

^{13}C NMR (151 MHz, 300 K, DMSO-d_6):

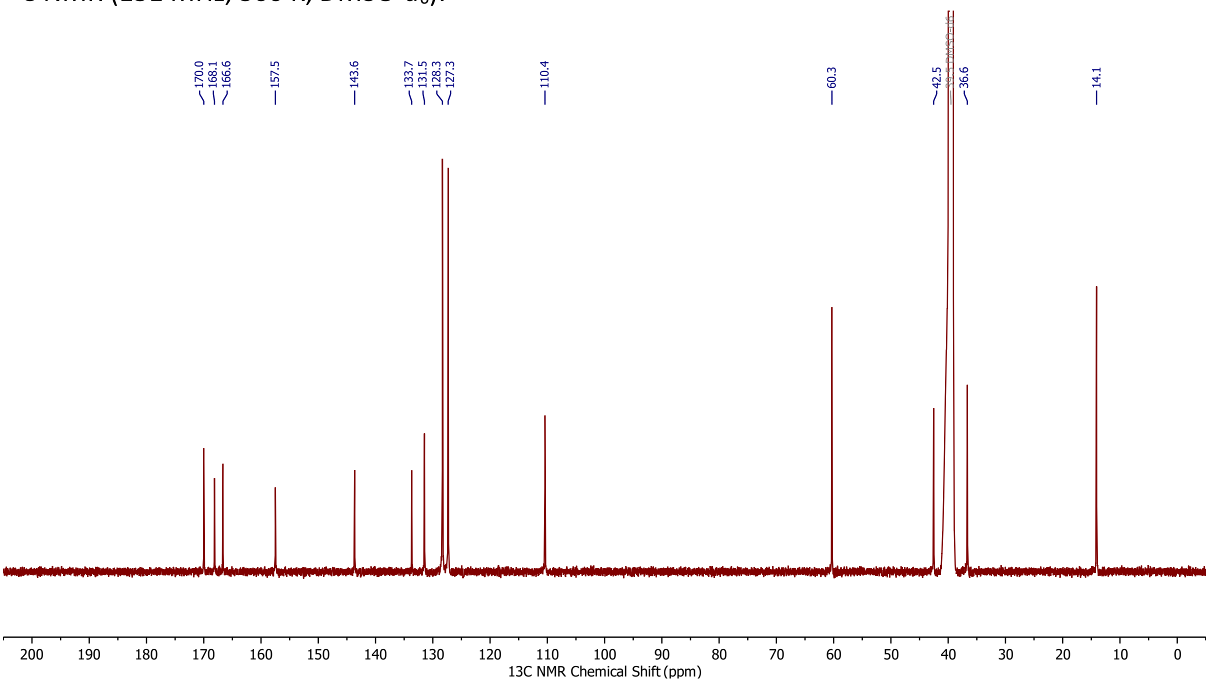

Ethyl (*Z*)-2-(2-((benzoylglycyl)imino)-3-hydroxy-2,3-dihydrothiazol-4-yl)acetate (16d)

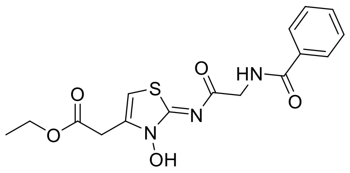

^{1}H NMR (600 MHz, 300 K, DMSO-d_6):

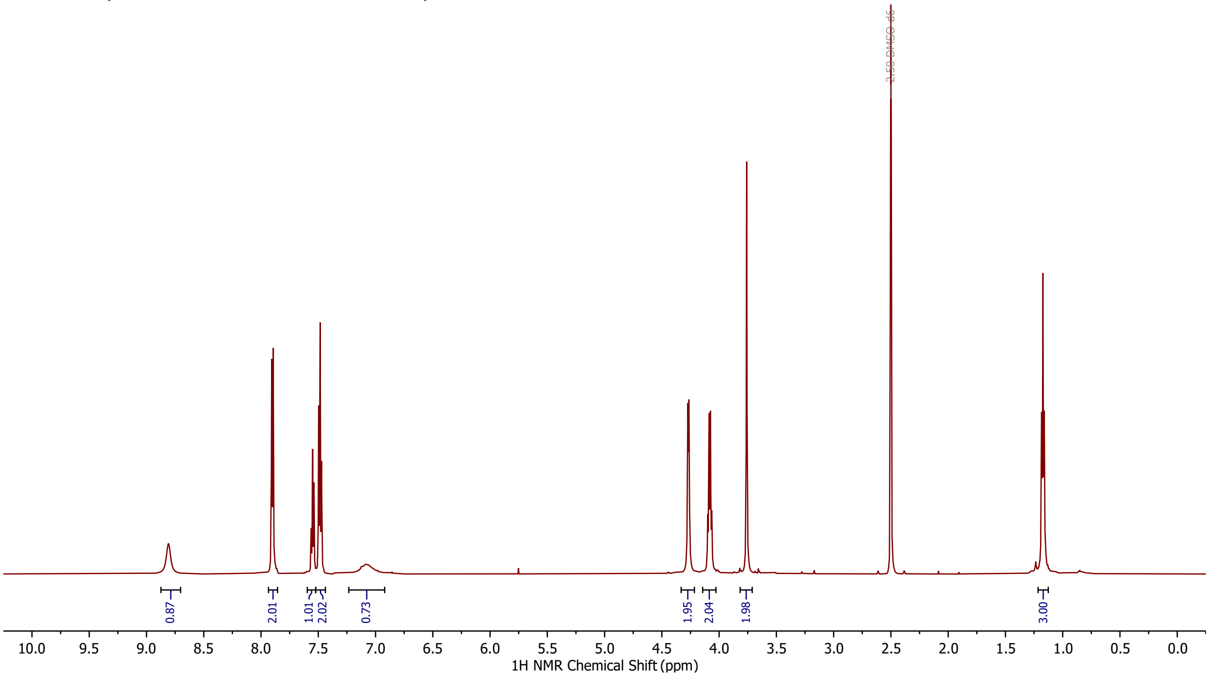

^{13}C NMR (151 MHz, 300 K, DMSO-d_6):

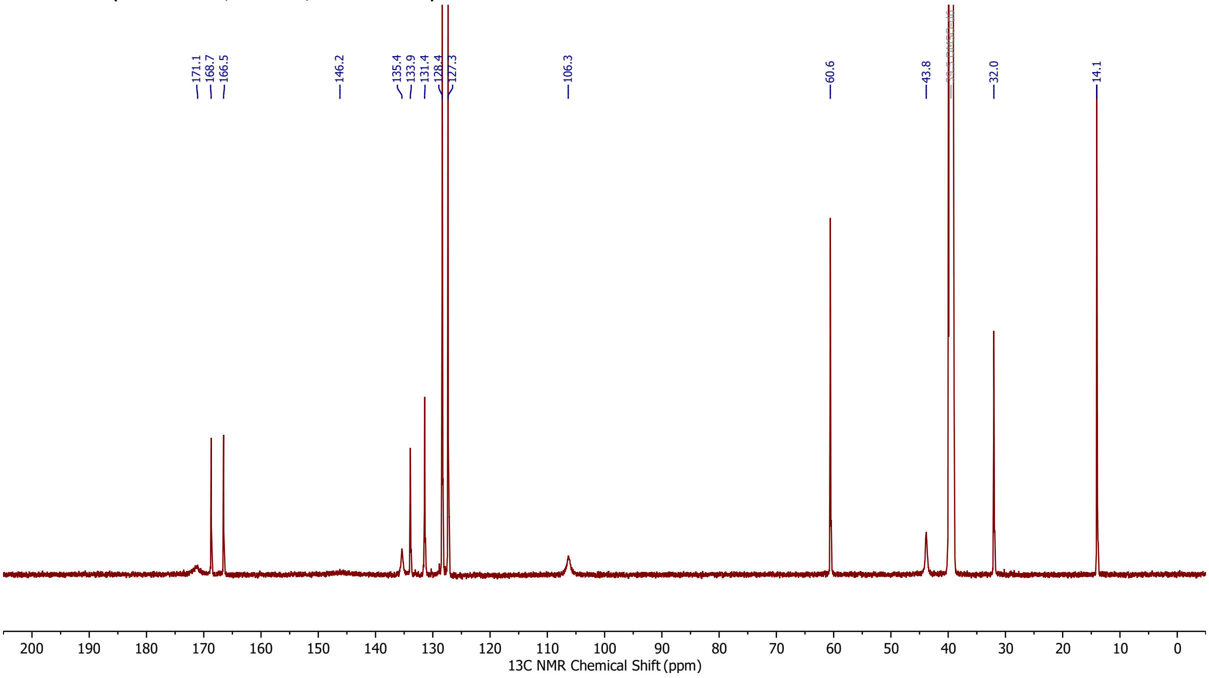

(*Z*)-2-(2-((Benzoylglycyl)imino)-3-hydroxy-2,3-dihydrothiazol-4-yl)acetic acid (16)

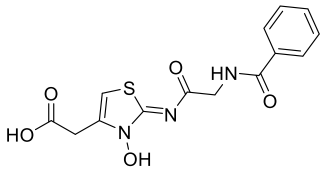

^{1}H NMR (600 MHz, 300 K, DMSO-d_6):

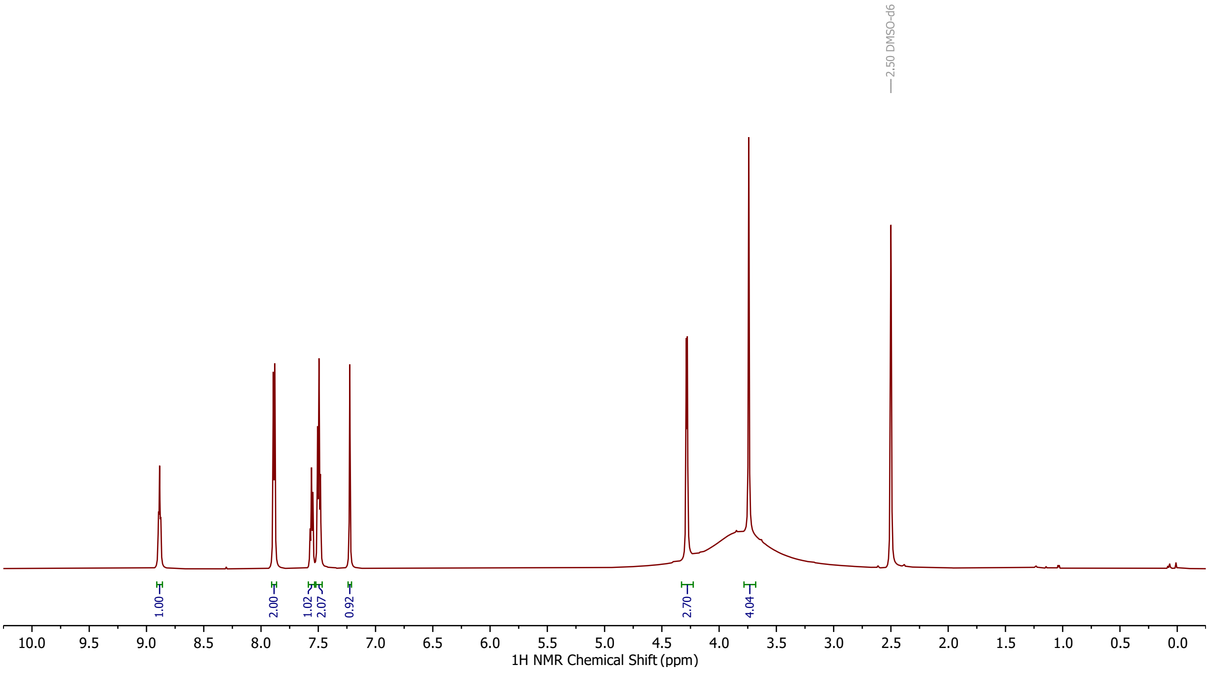

^{13}C NMR (151 MHz, 300 K, DMSO-d_6):

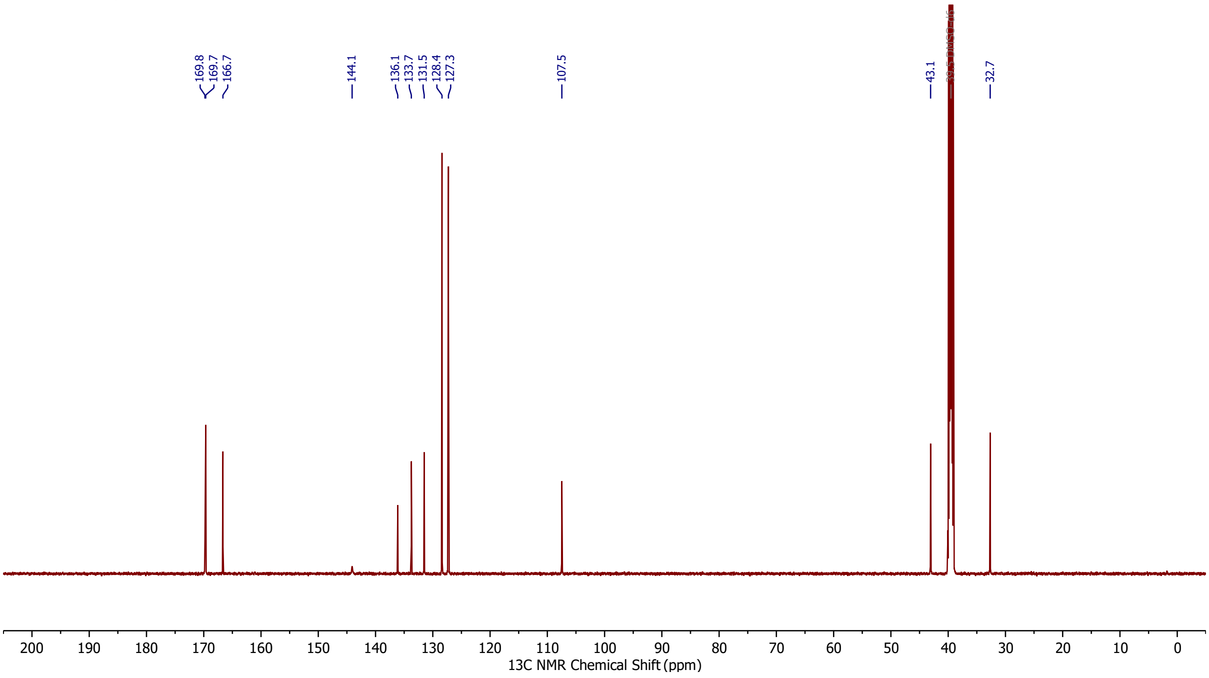

Ethyl 2-(2-(3-((*tert*-butoxycarbonyl)amino)propanamido)thiazol-4-yl)acetate (17a)

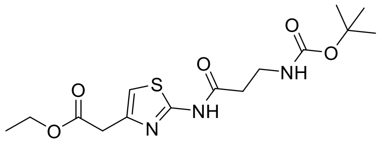

^{1}H NMR (600 MHz, 300 K, DMSO-d_6):

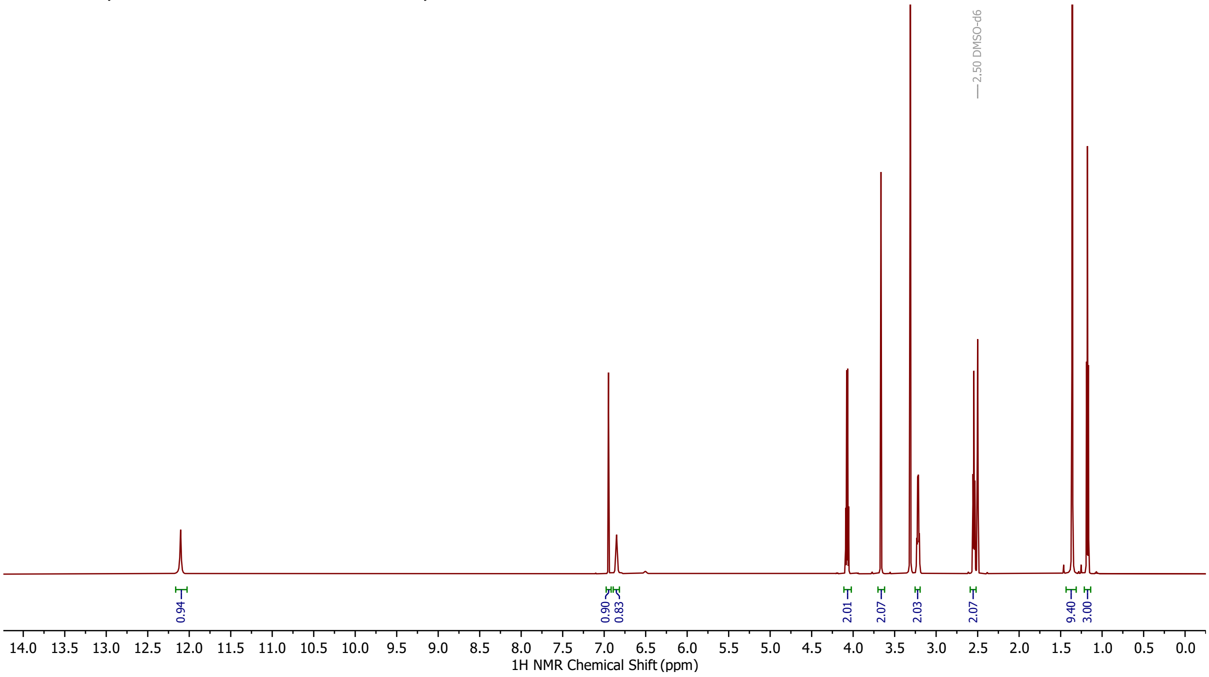

^{13}C NMR (151 MHz, 300 K, DMSO-d_6):

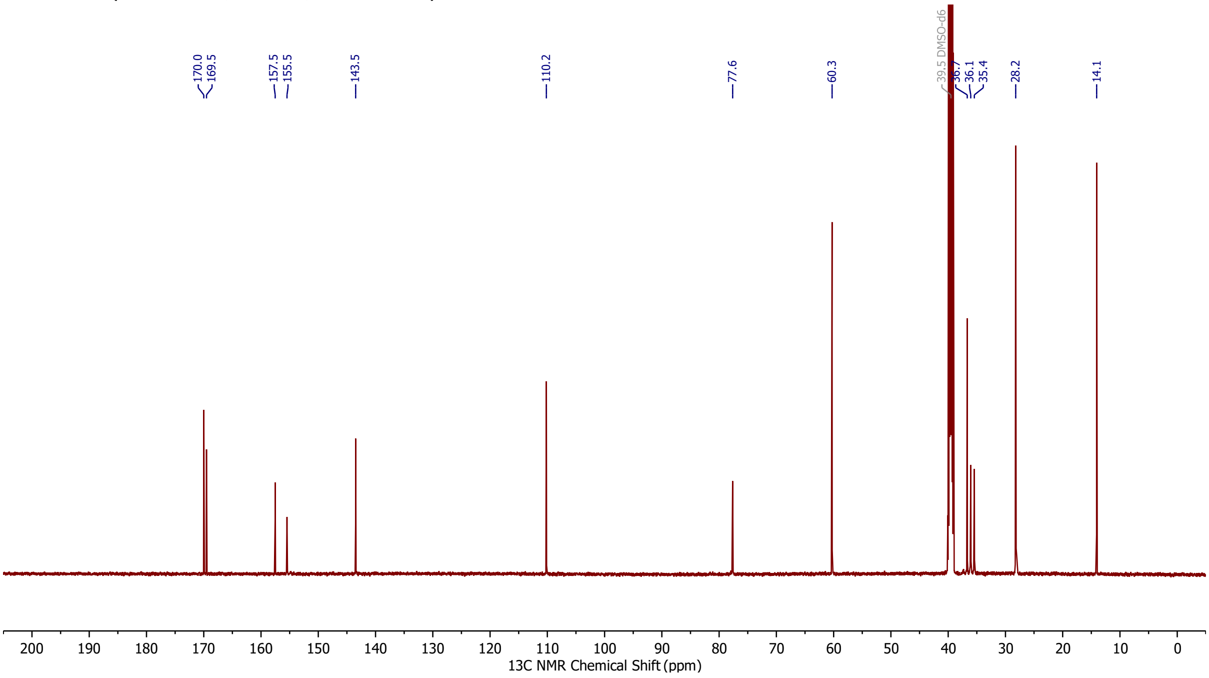

Ethyl 2-(2-(3-benzamidopropanamido)thiazol-4-yl)acetate (17c)

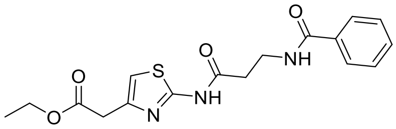

^{1}H NMR (600 MHz, 300 K, $CDCl_3$):

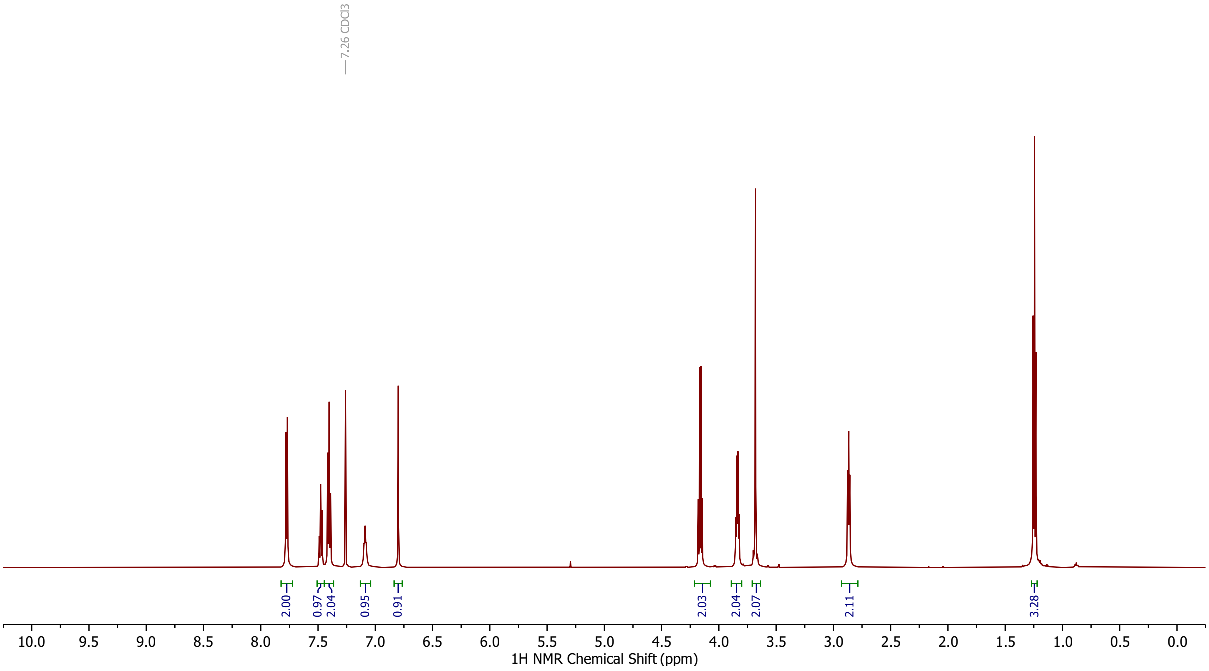

^{13}C NMR (151 MHz, 300 K, $CDCl_3$):

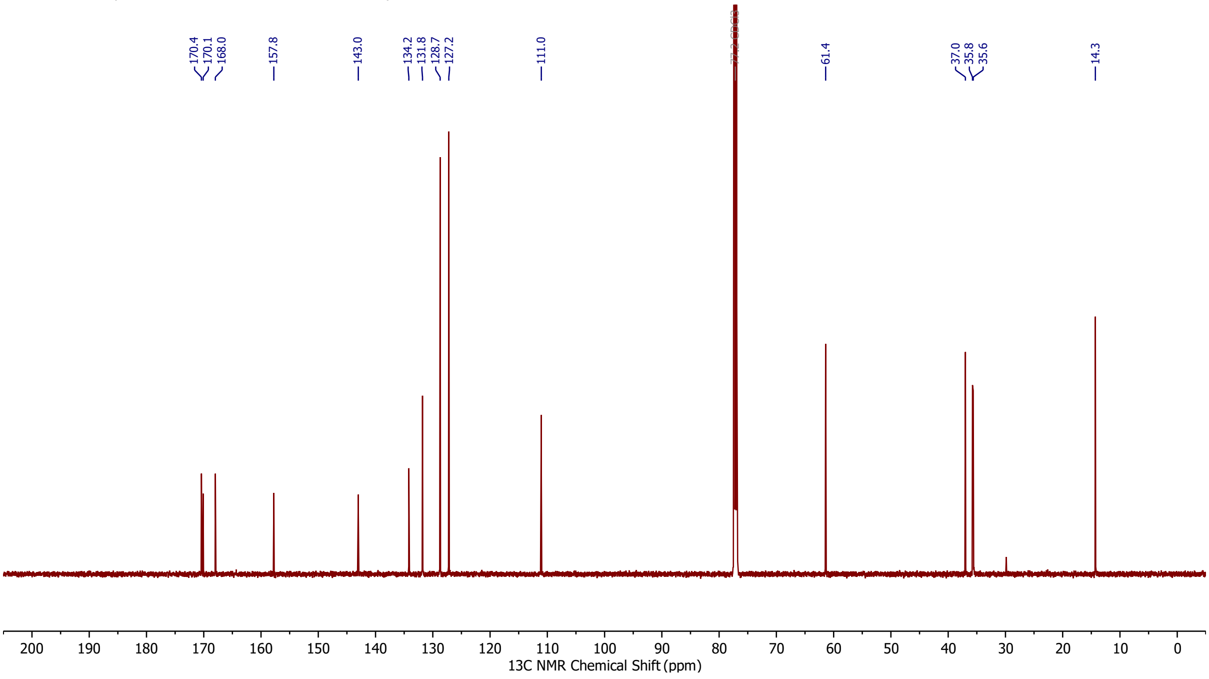

Ethyl (*Z*)-2-(2-((3-benzamidopropanoyl)imino)-3-hydroxy-2,3-dihydrothiazol-4-yl)acetate (17d)

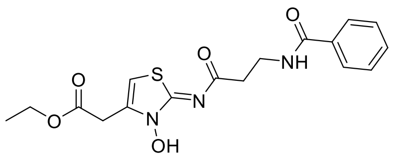

^{1}H NMR (600 MHz, 300 K, DMSO-d_6):

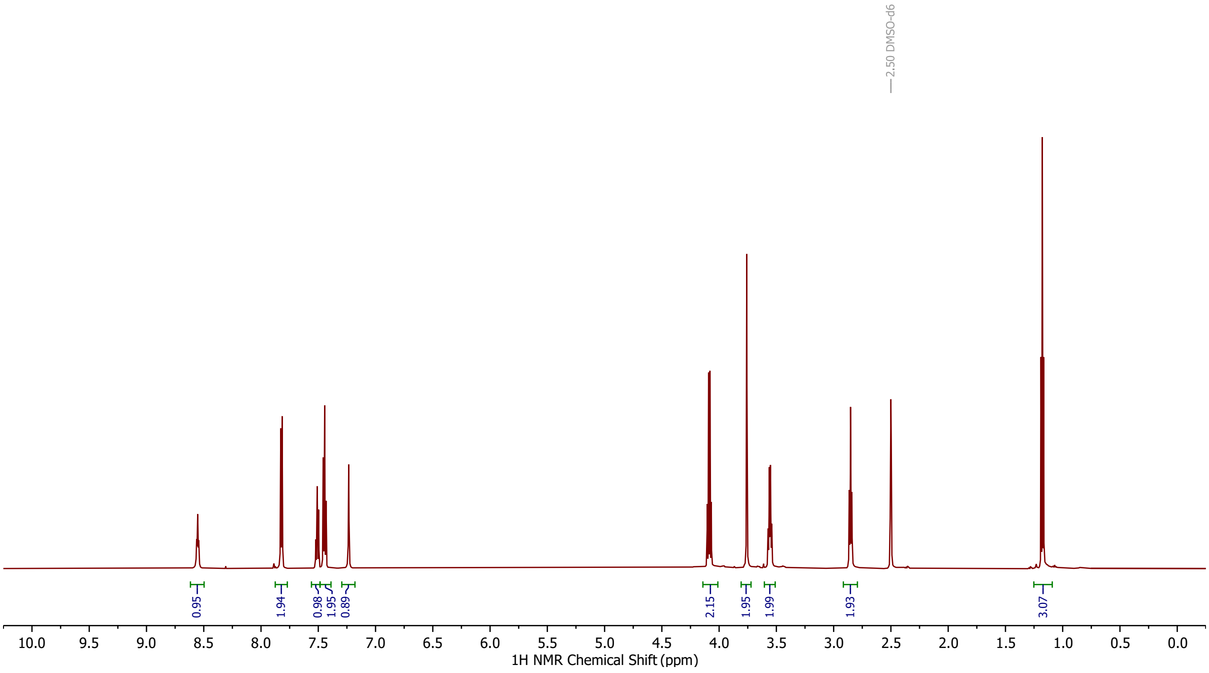

^{13}C NMR (151 MHz, 300 K, DMSO-d_6):

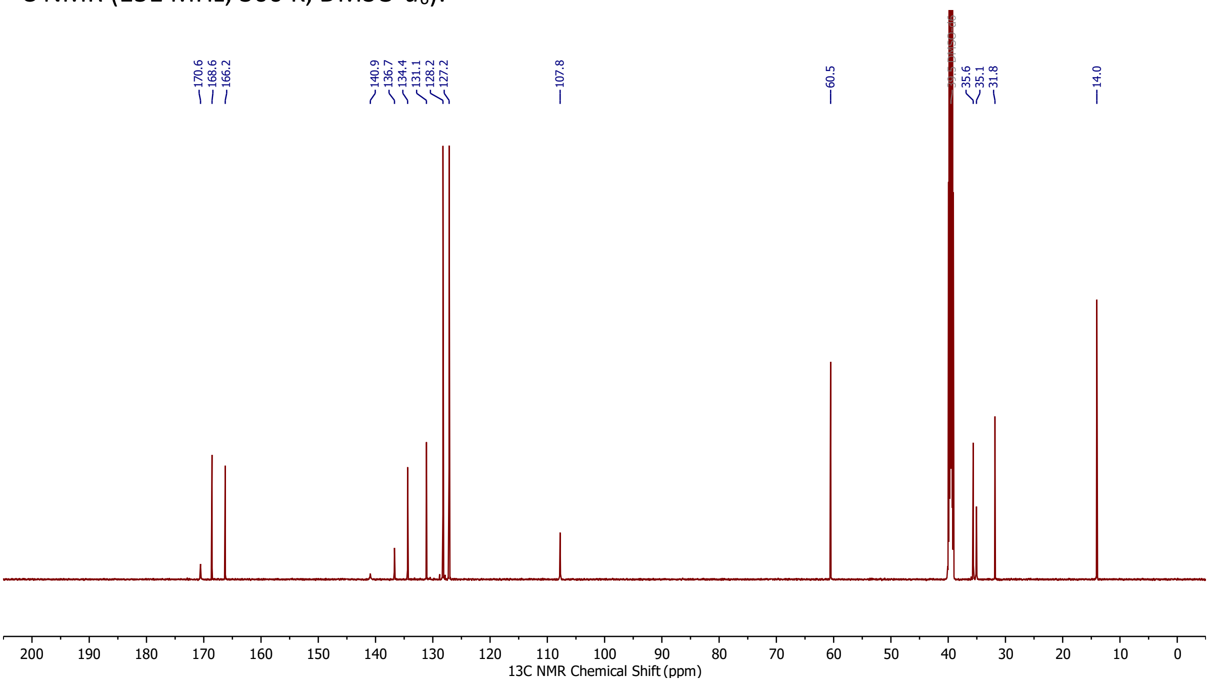

(*Z*)-2-(2-((3-Benzamidopropanoyl)imino)-3-hydroxy-2,3-dihydrothiazol-4-yl)acetic acid (17)

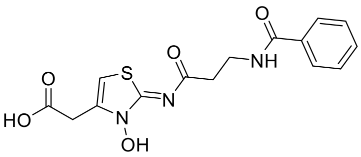

^{1}H NMR (600 MHz, 300 K, DMSO-d_6):

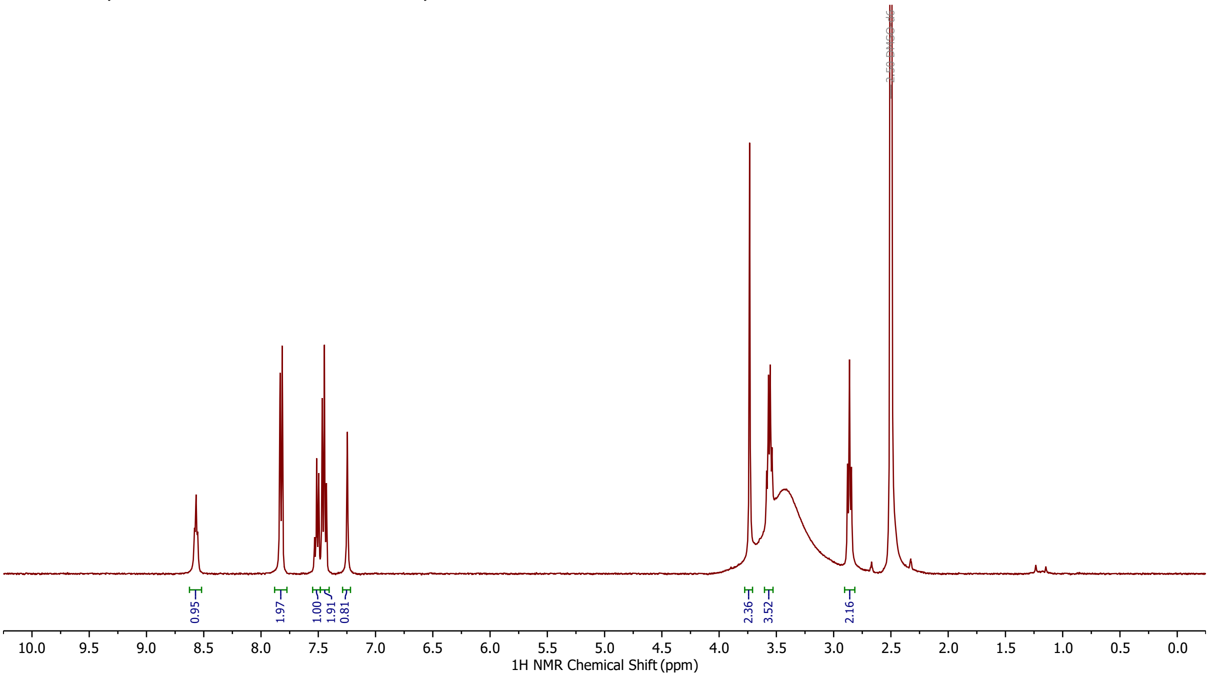

^{13}C NMR (151 MHz, 300 K, DMSO-d_6):

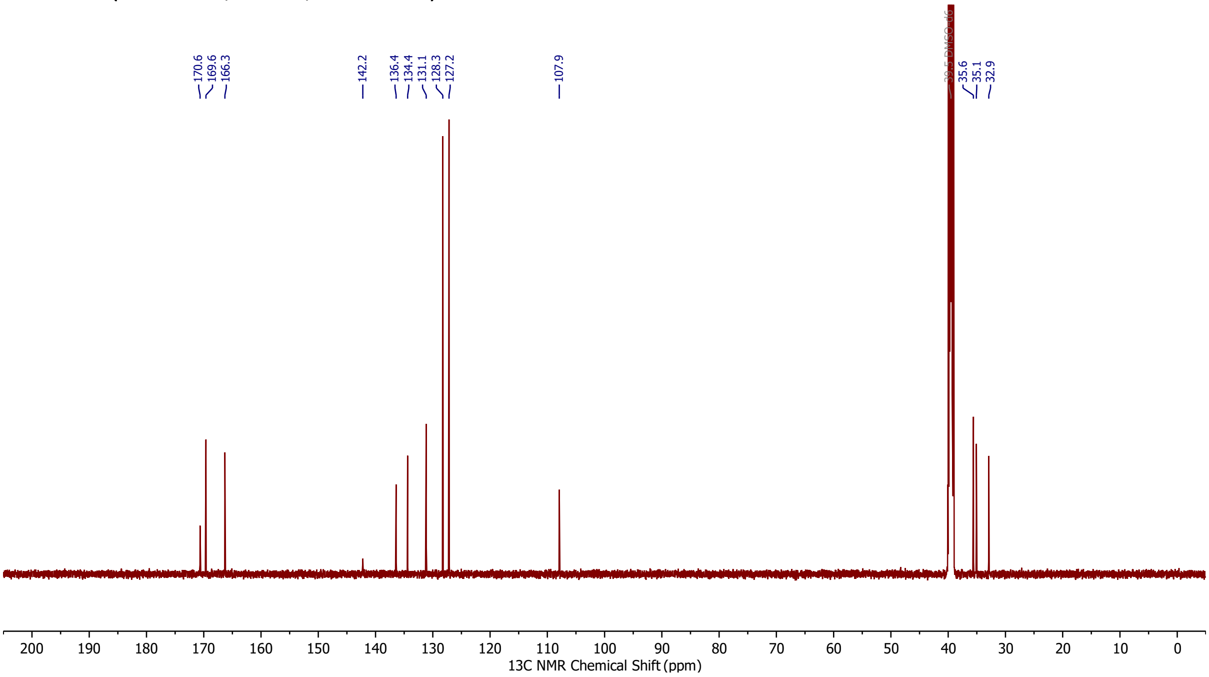

4-((4-(2-Ethoxy-2-oxoethyl)thiazol-2-yl)amino)-4-oxobutanoic acid (18a)

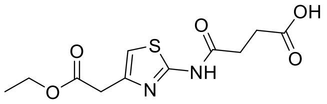

^{1}H NMR (600 MHz, 300 K, DMSO-d_6):

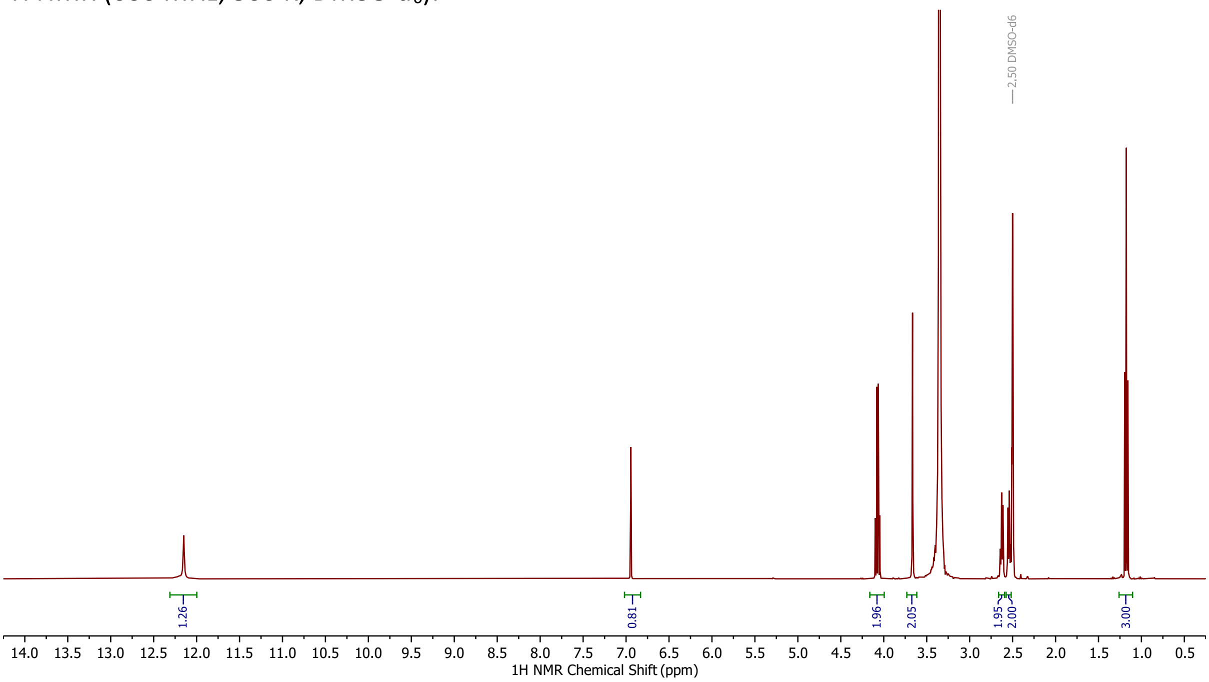

^{13}C NMR (151 MHz, 300 K, DMSO-d_6):

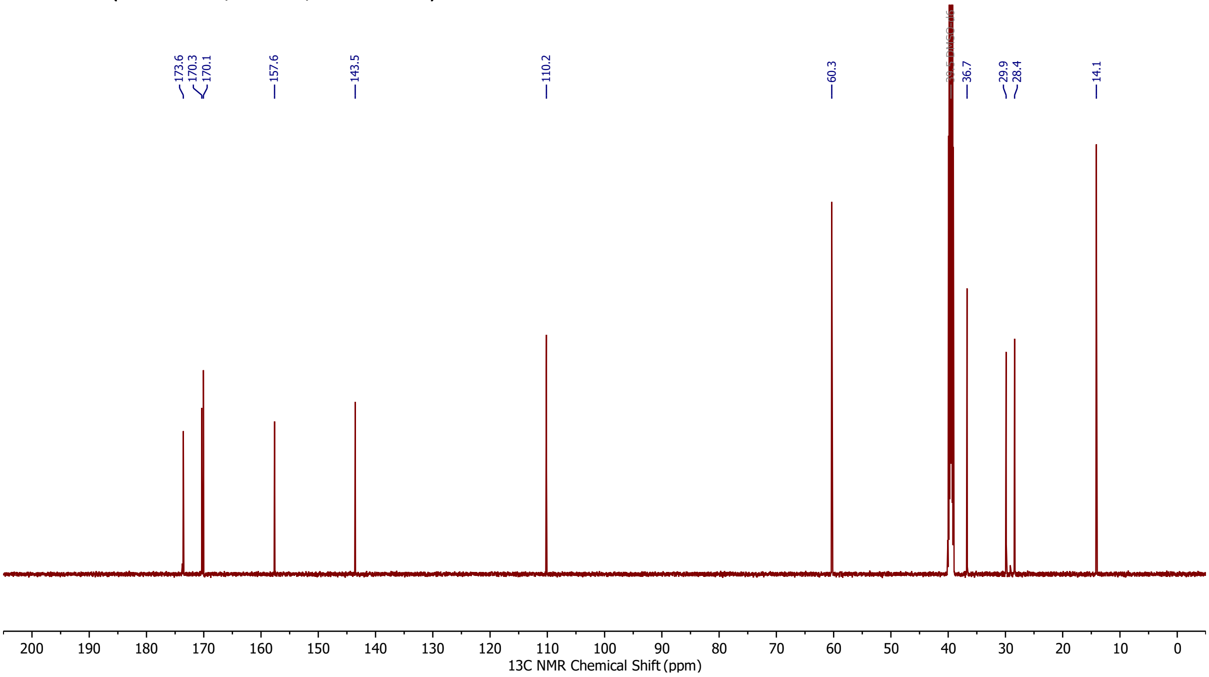

Ethyl 2-(2-(2-((tert-butoxycarbonyl)amino)acetamido)thiazol-4-yl)acetate (18b)

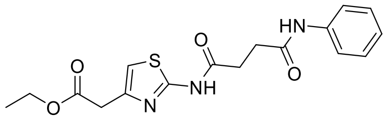

^{1}H NMR (600 MHz, 300 K, $CDCl_3$):

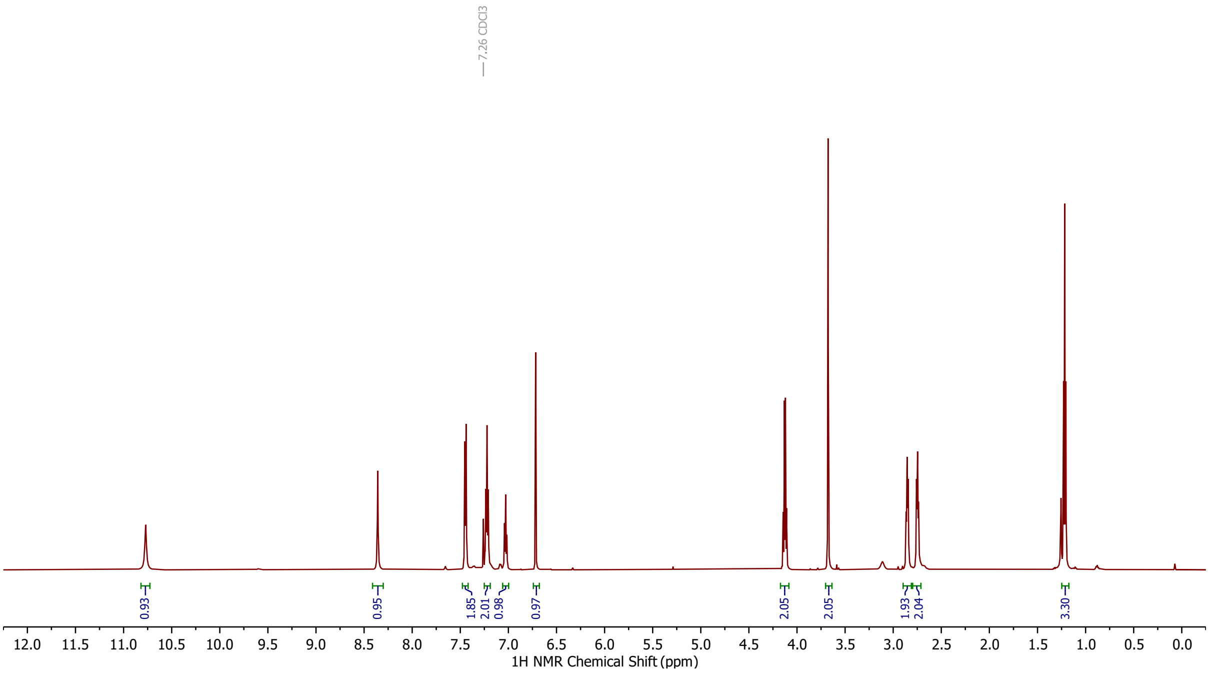

^{13}C NMR (151 MHz, 300 K, $CDCl_3$):

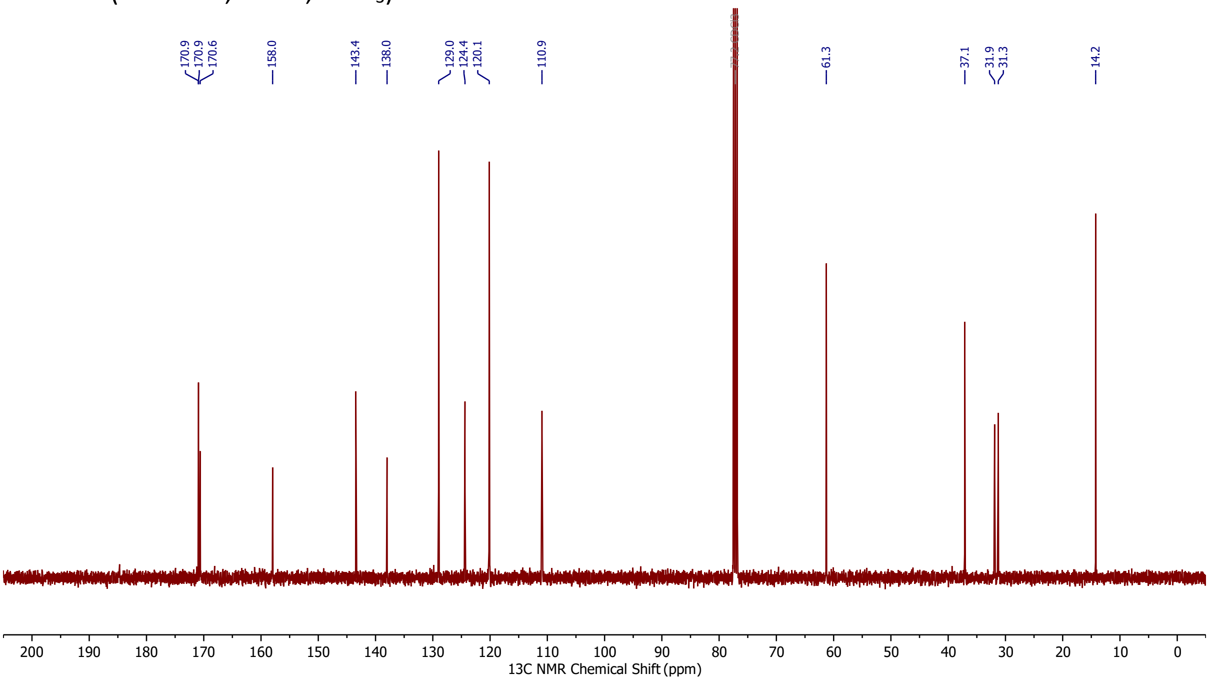

Ethyl (*Z*)-2-(3-hydroxy-2-((4-oxo-4-(phenylamino)butanoyl)imino)-2,3-dihydrothiazol-4-yl)acetate (18c)

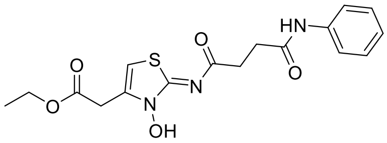

^{1}H NMR (400 MHz, 300 K, DMSO-d_6):

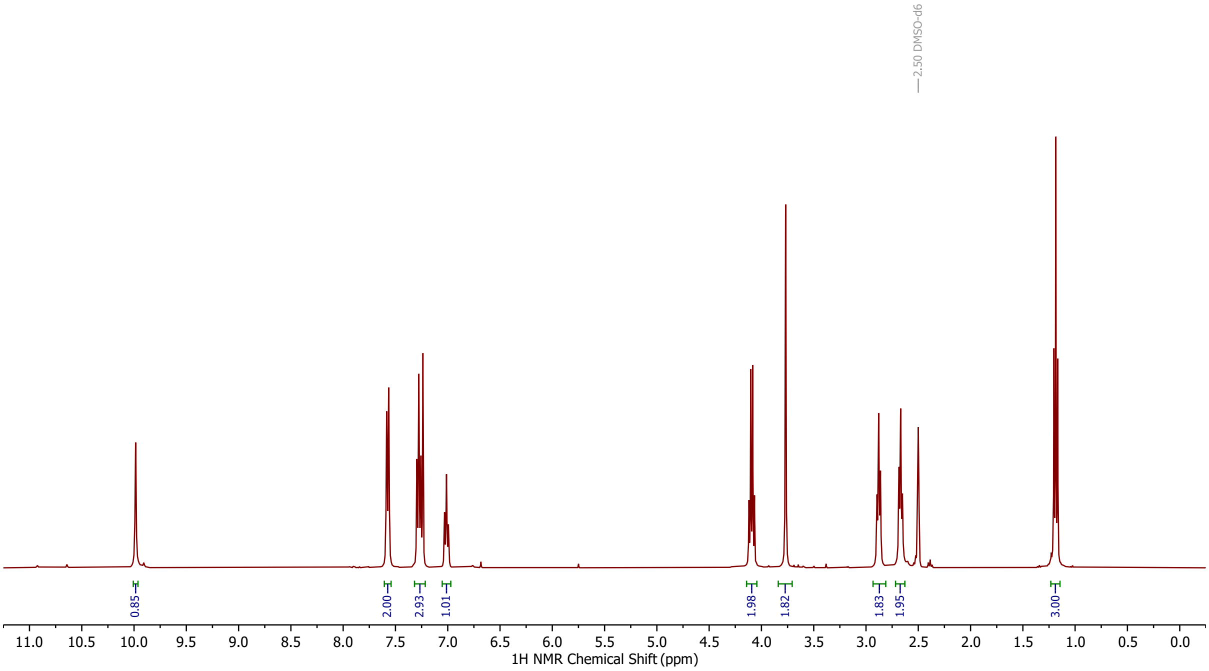

^{13}C NMR (101 MHz, 300 K, DMSO-d_6):

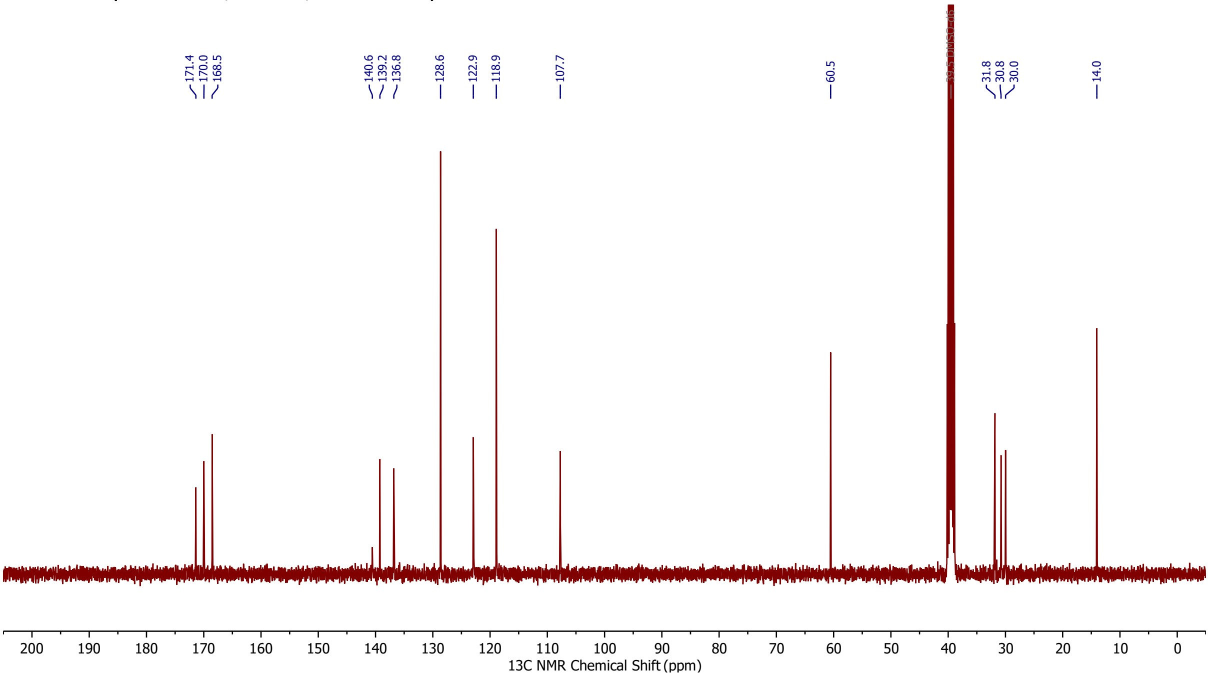

(*Z*)-2-(3-Hydroxy-2-((4-oxo-4-(phenylamino)butanoyl)imino)-2,3-dihydrothiazol-4-yl)acetic acid (18)

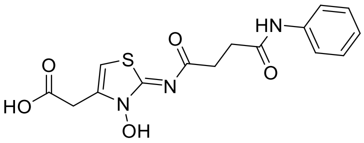

^{1}H NMR (600 MHz, 300 K, $CDCl_3$):

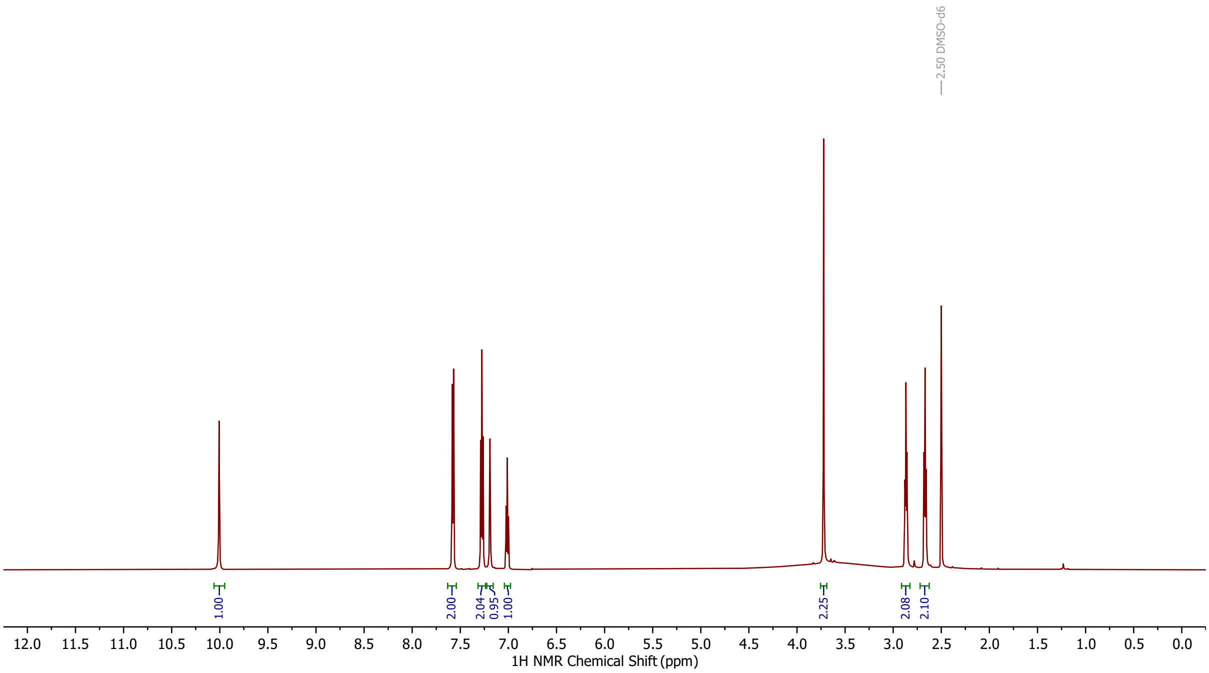

^{13}C NMR (151 MHz, 300 K, $CDCl_3$):

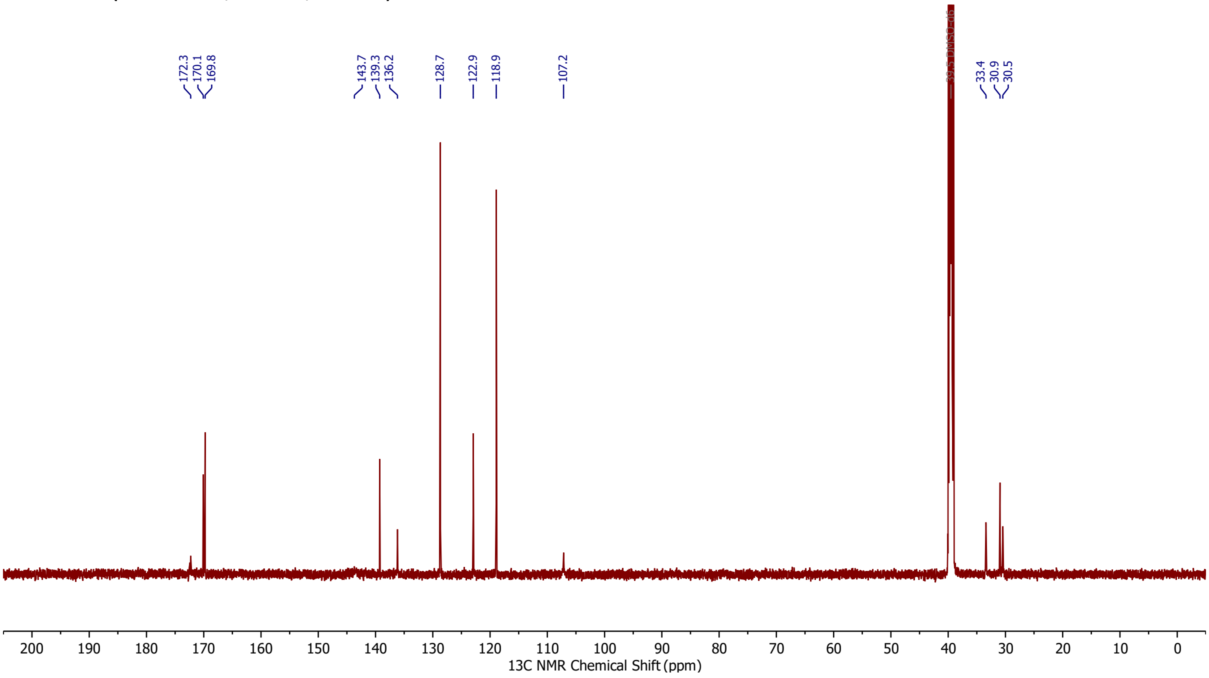

Ethyl 2-(2-(2-(phenylsulfonamido)acetamido)thiazol-4-yl)acetate (19a)

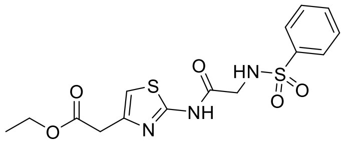

^{1}H NMR (600 MHz, 300 K, $CDCl_3$):

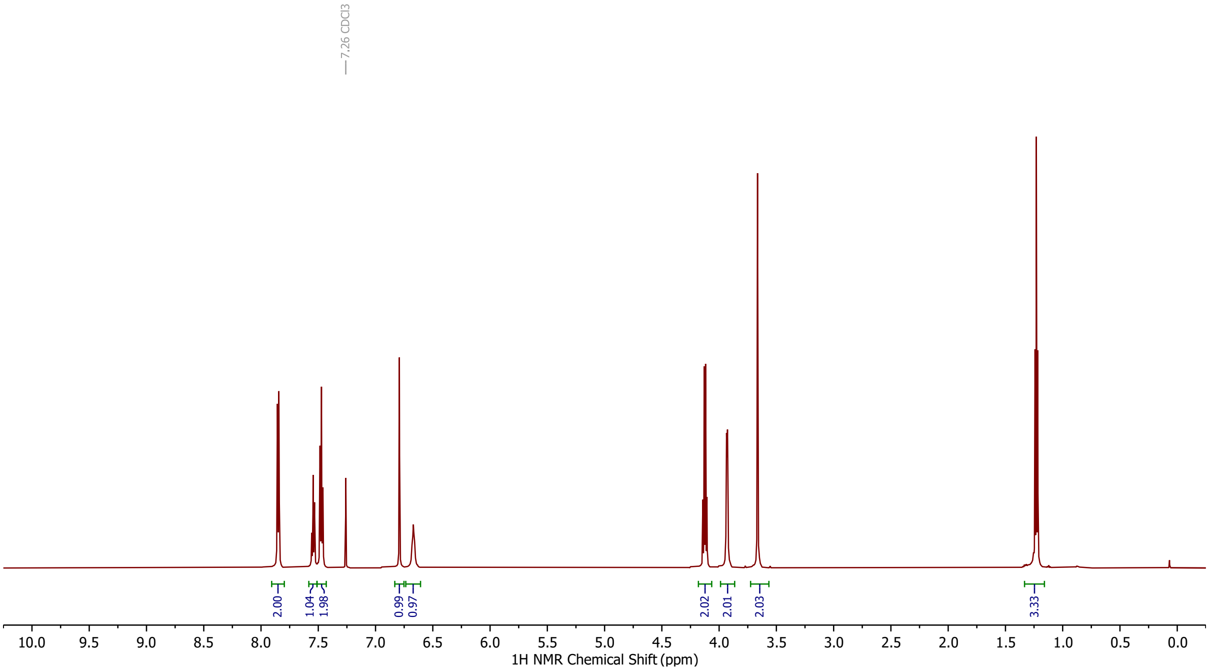

^{13}C NMR (151 MHz, 300 K, $CDCl_3$):

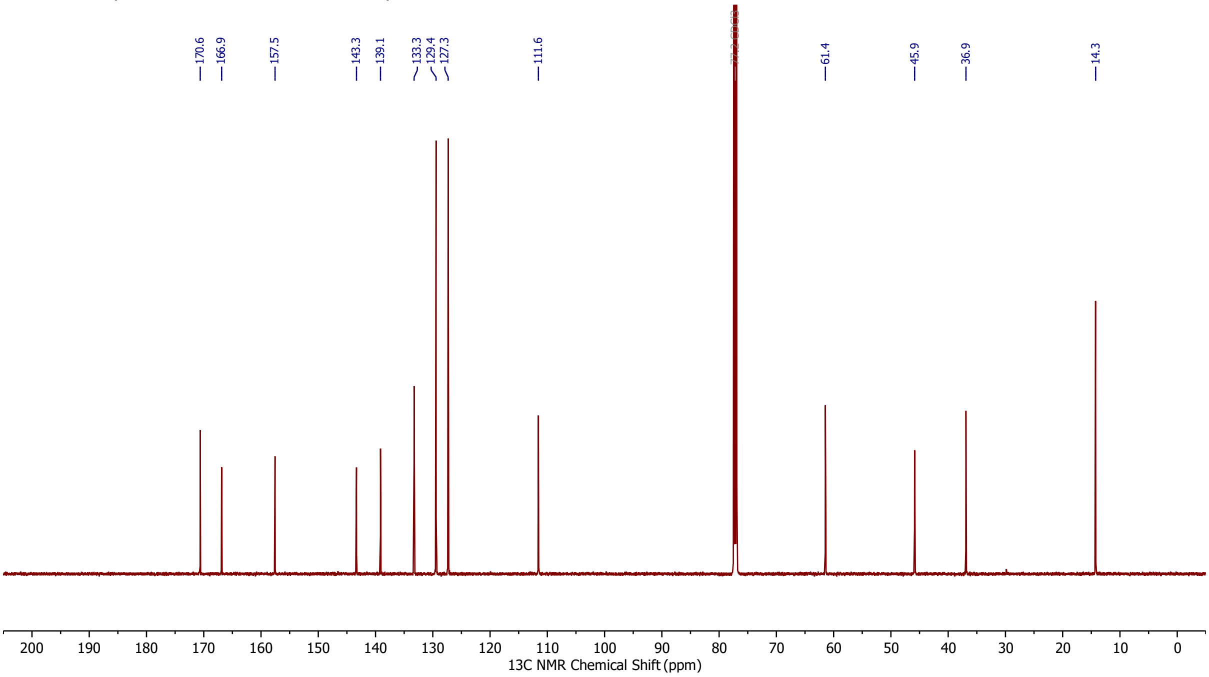

Ethyl (*Z*)-2-(3-hydroxy-2-(((phenylsulfonyl)glycyl)imino)-2,3-dihydrothiazol-4-yl)acetate (19b)

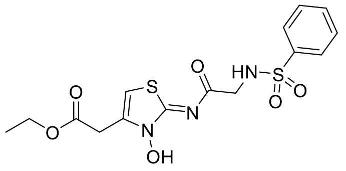

^{1}H NMR (600 MHz, 300 K, DMSO-d_6):

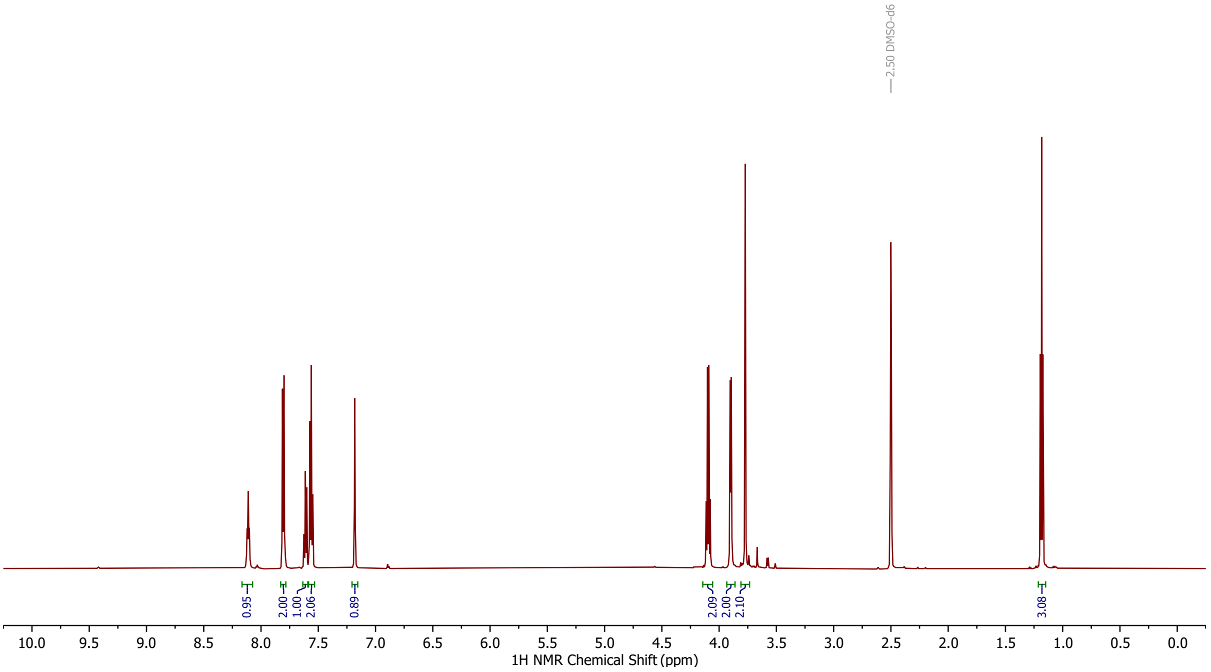

^{13}C NMR (151 MHz, 300 K, DMSO-d_6):

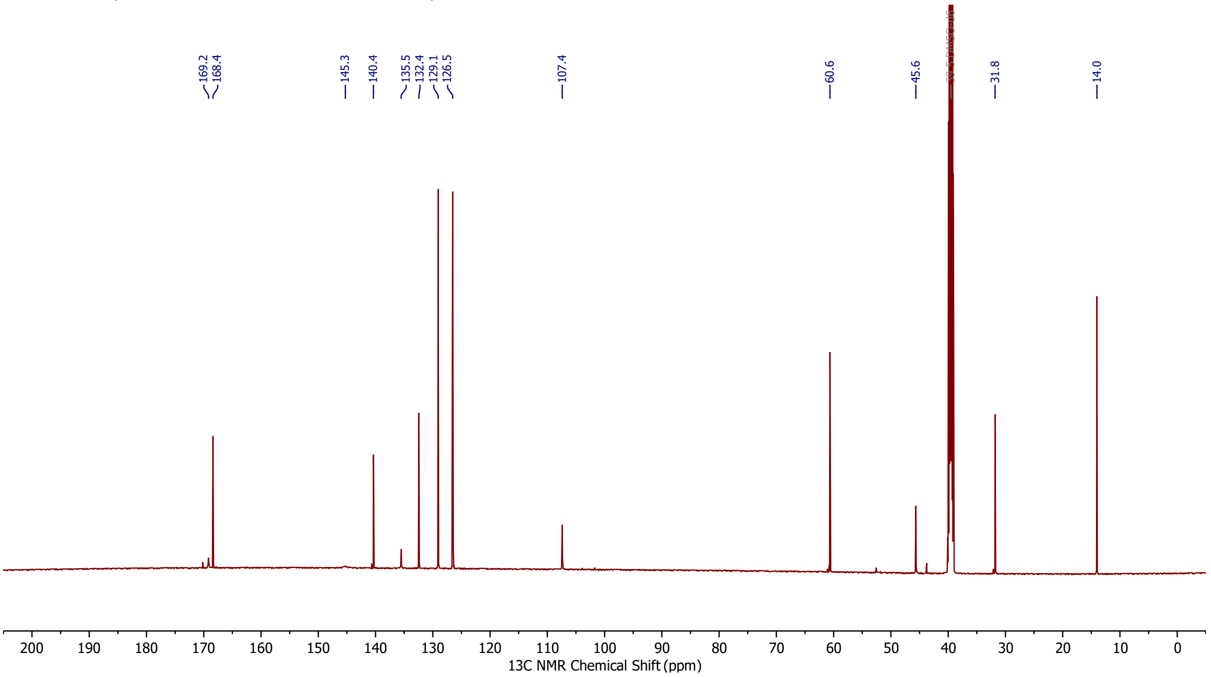

(*Z*)-2-(3-Hydroxy-2-(((phenylsulfonyl)glycyl)imino)-2,3-dihydrothiazol-4-yl)acetic acid (19)

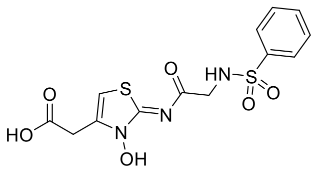

^{1}H NMR (600 MHz, 300 K, DMSO-d_6):

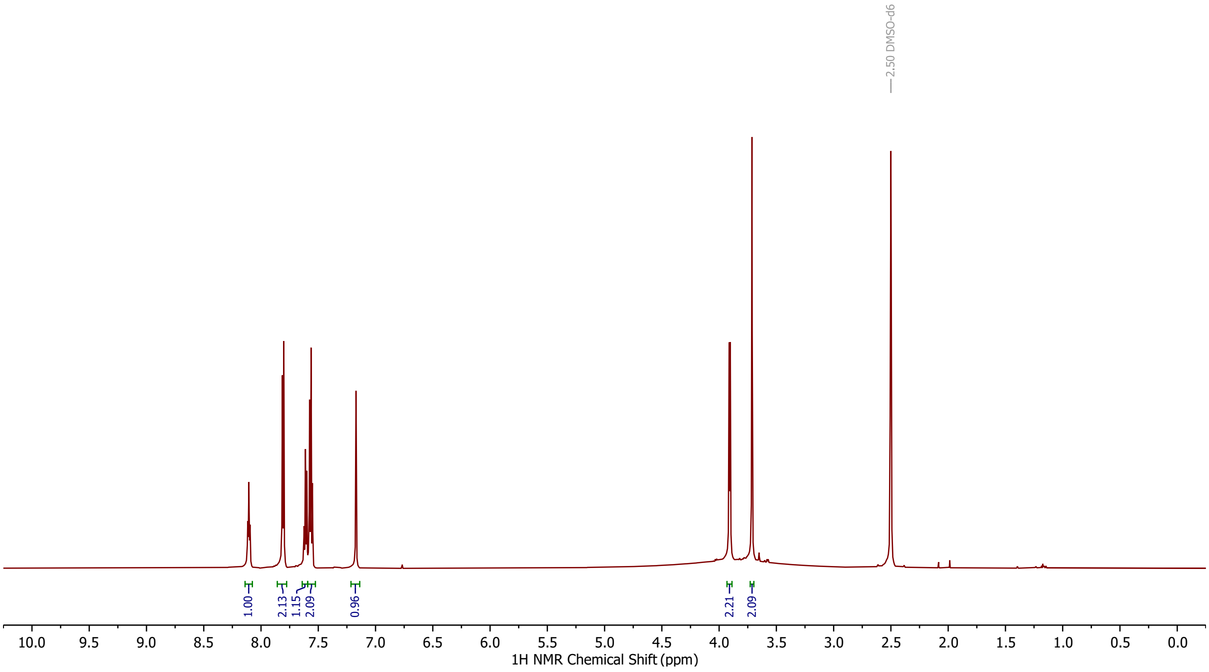

^{13}C NMR (151 MHz, 300 K, DMSO-d_6):

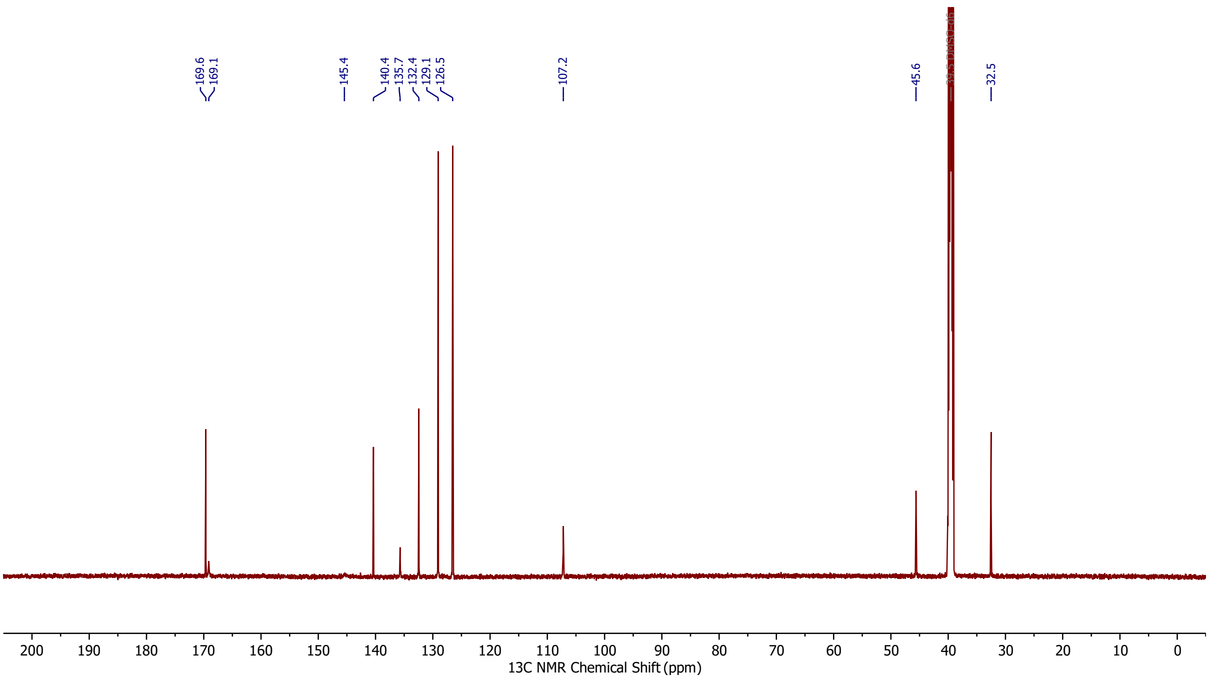

Ethyl 2-(2-(3-(phenylsulfonamido)propanamido)thiazol-4-yl)acetate (20a)

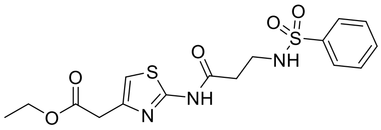

^{1}H NMR (600 MHz, 300 K, $CDCl_3$):

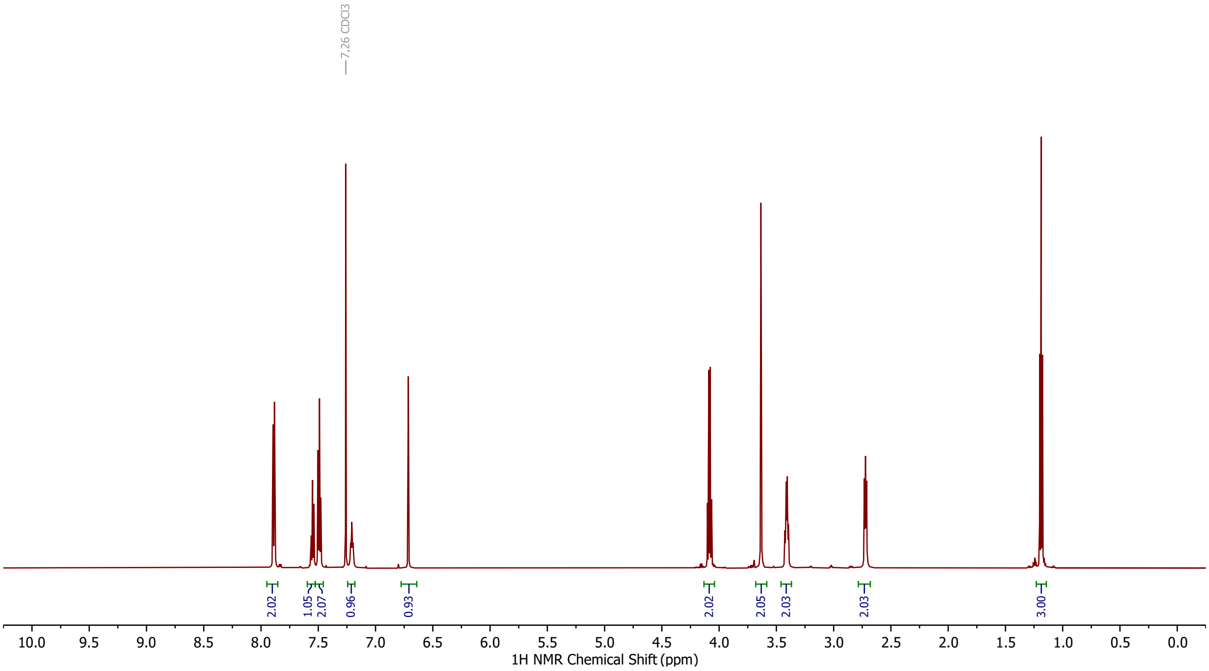

^{13}C NMR (151 MHz, 300 K, $CDCl_3$):

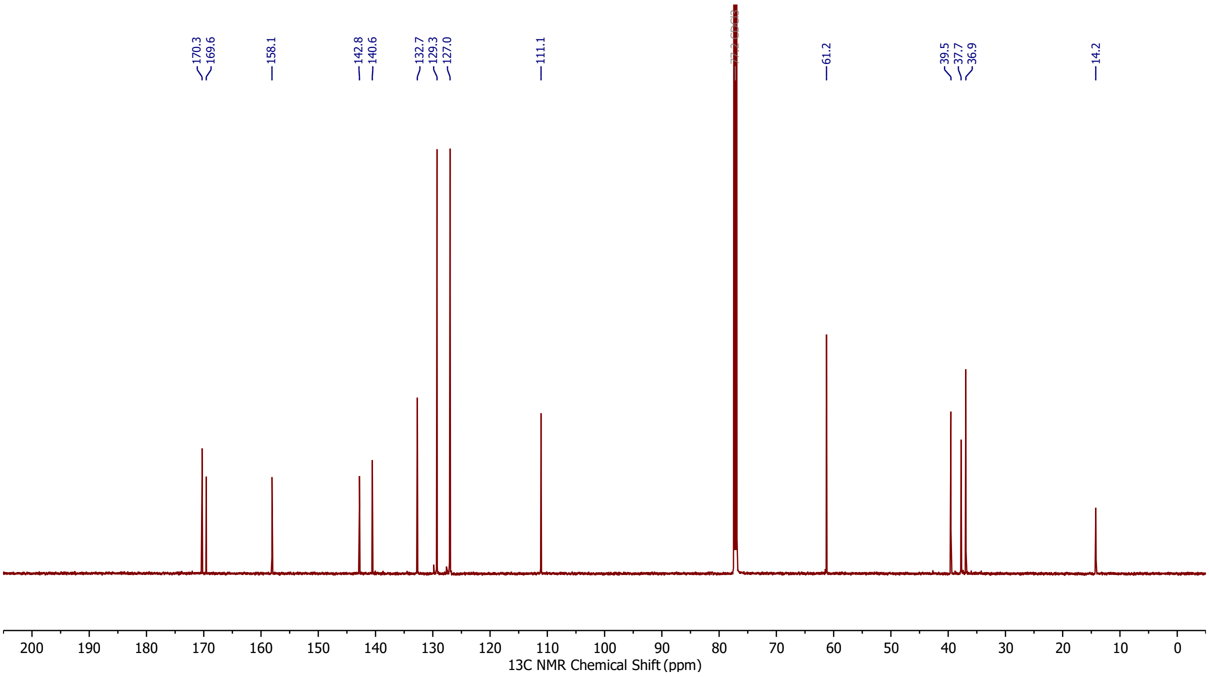

Ethyl (*Z*)-2-(2-((*N*-(tert-butoxycarbonyl)-*N*-phenylglycyl)imino)-3-hydroxy-2,3-dihydrothiazol-4-yl)acetate (20b)

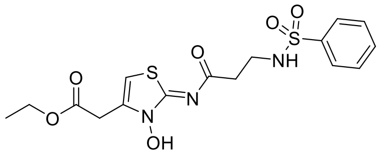

^{1}H NMR (600 MHz, 300 K, DMSO-d_6):

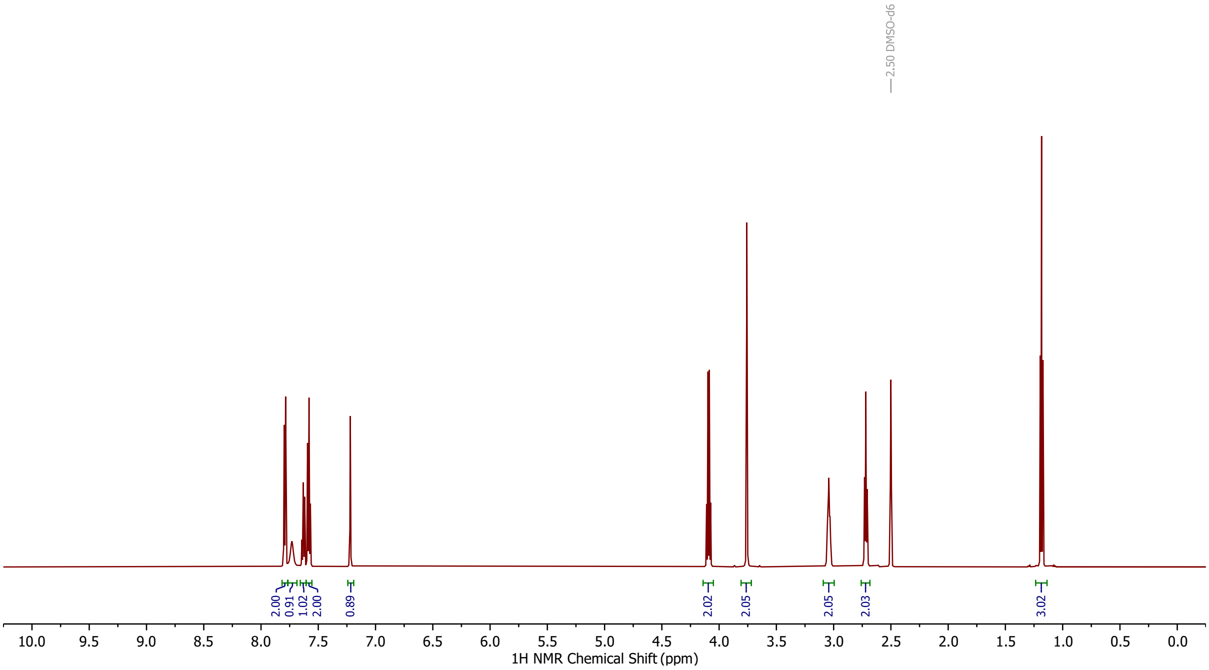

^{13}C NMR (151 MHz, 300 K, DMSO-d_6):

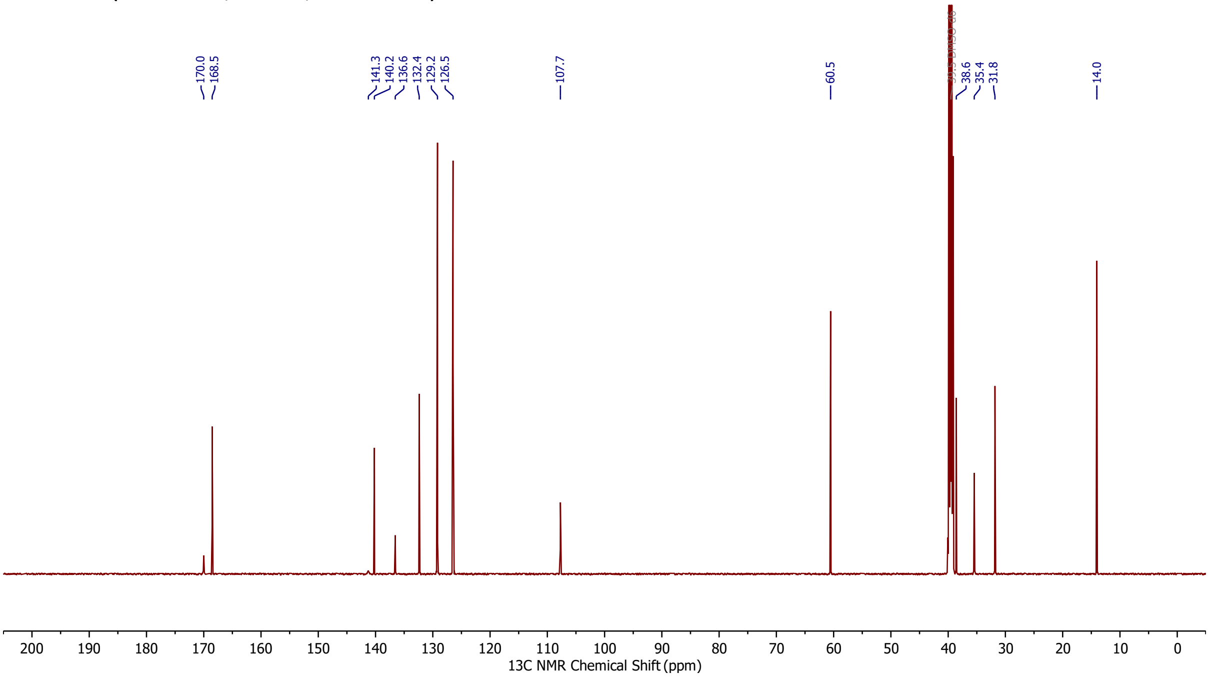

(*Z*)-2-(3-Hydroxy-2-((3-(phenylsulfonamido)propanoyl)imino)-2,3-dihydrothiazol-4-yl)acetic acid (20)

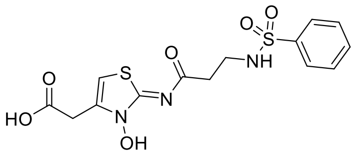

^{1}H NMR (600 MHz, 300 K, DMSO-d_6):

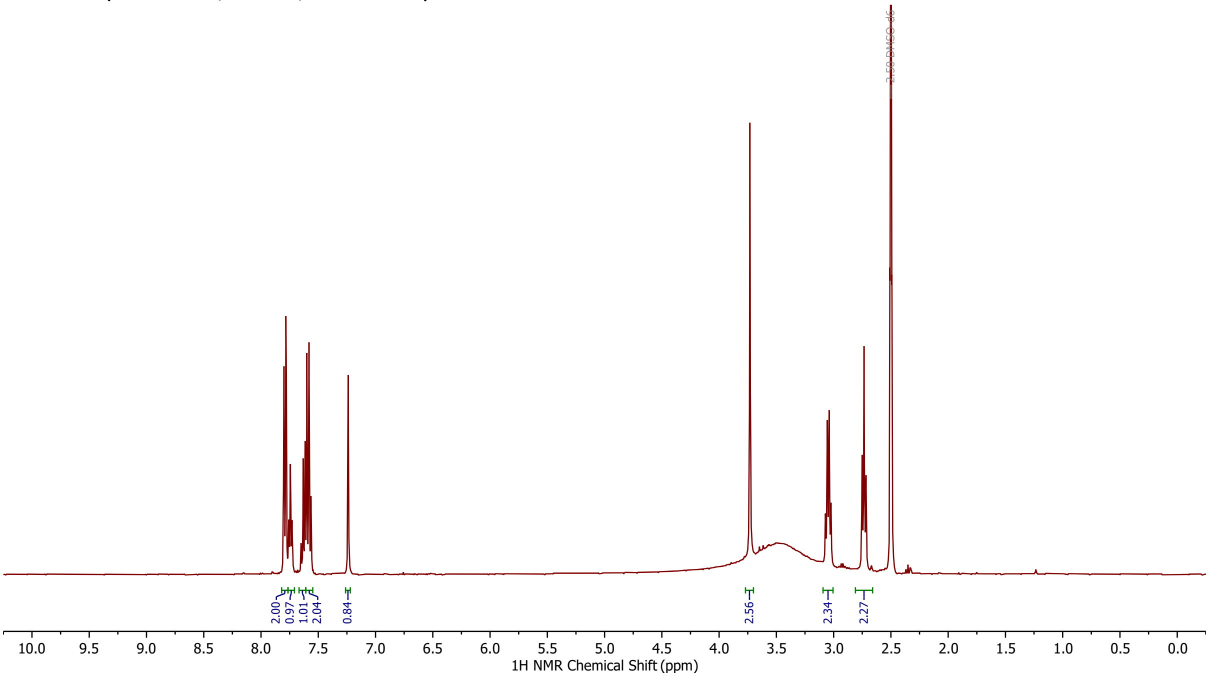

^{13}C NMR (151 MHz, 300 K, DMSO-d_6):

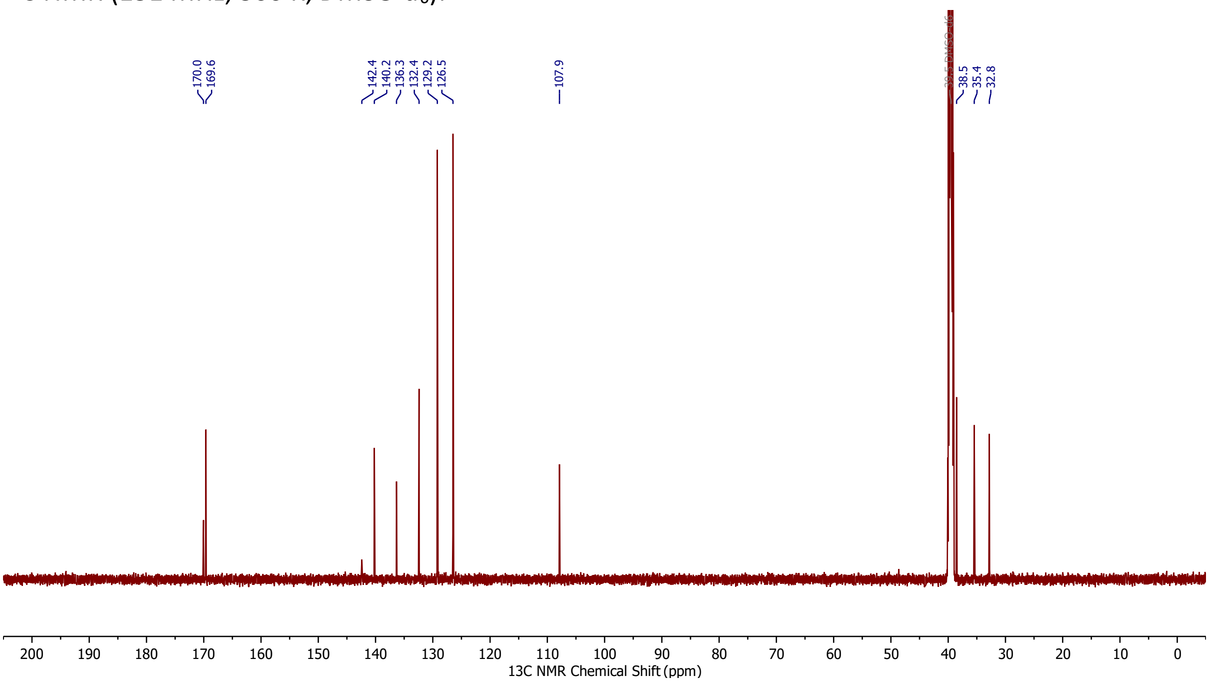

Ethyl 2-(2-(2-(*N*-phenylsulfamoyl)acetamido)thiazol-4-yl)acetate (21a)

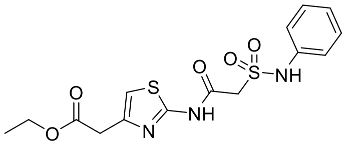

^{1}H NMR (600 MHz, 300 K, DMSO-d_6):

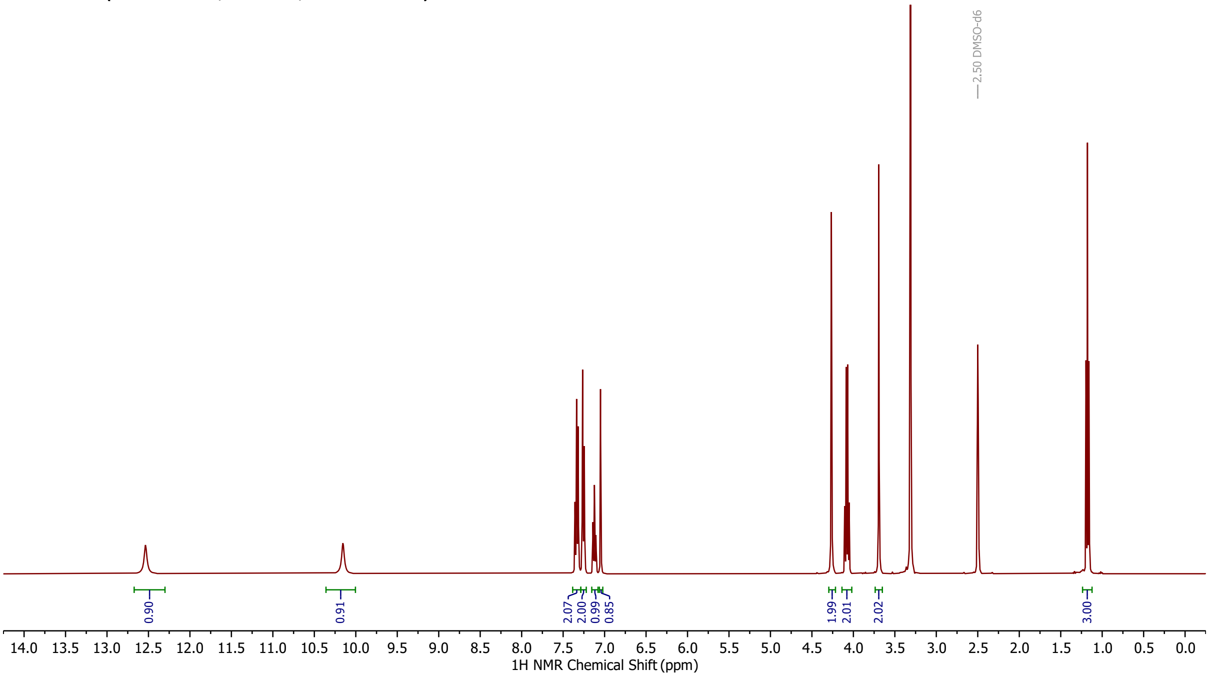

^{13}C NMR (151 MHz, 300 K, DMSO-d_6):

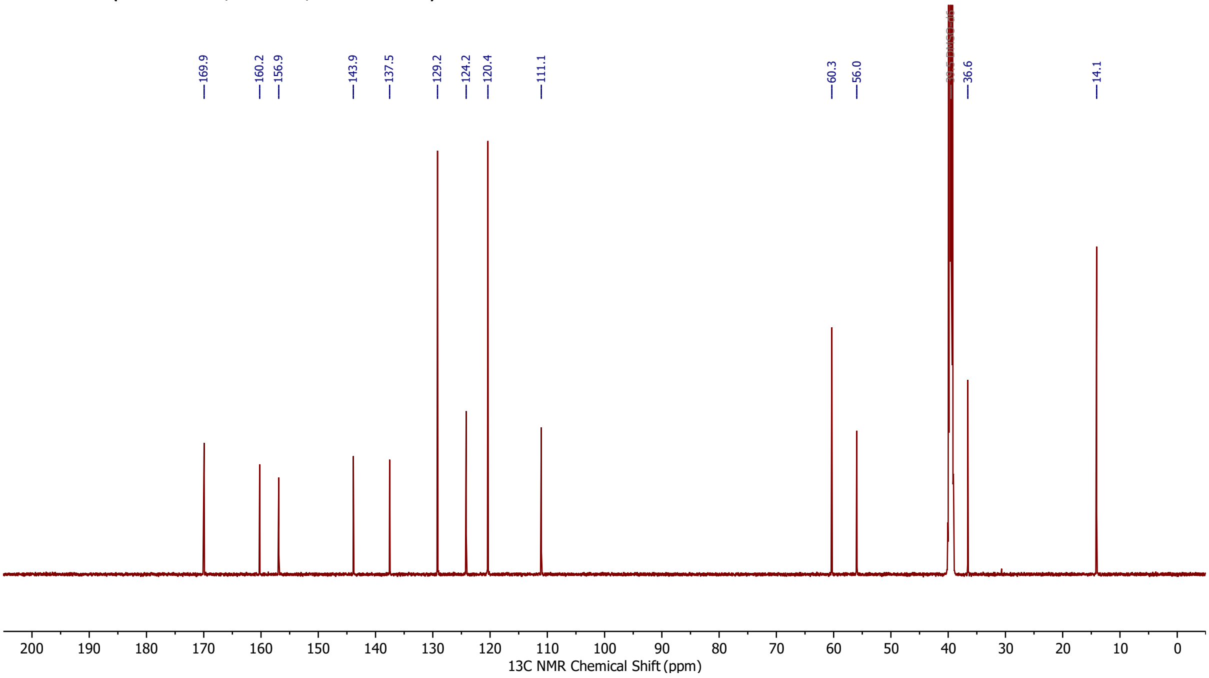

Ethyl (*Z*)-2-(3-hydroxy-2-((2-(*N*-phenylsulfamoyl)acetyl)imino)-2,3-dihydrothiazol-4-yl)acetate (21b)

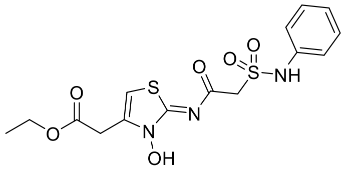

^{1}H NMR (600 MHz, 300 K, DMSO-d_6):

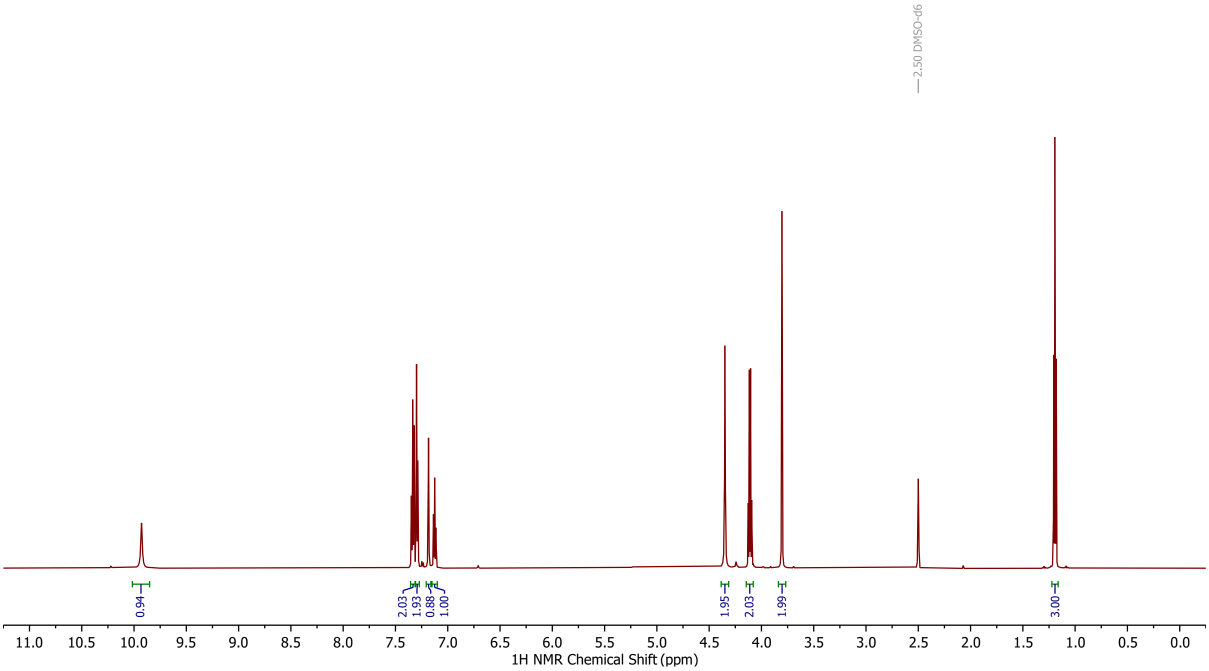

^{13}C NMR (151 MHz, 300 K, DMSO-d_6):

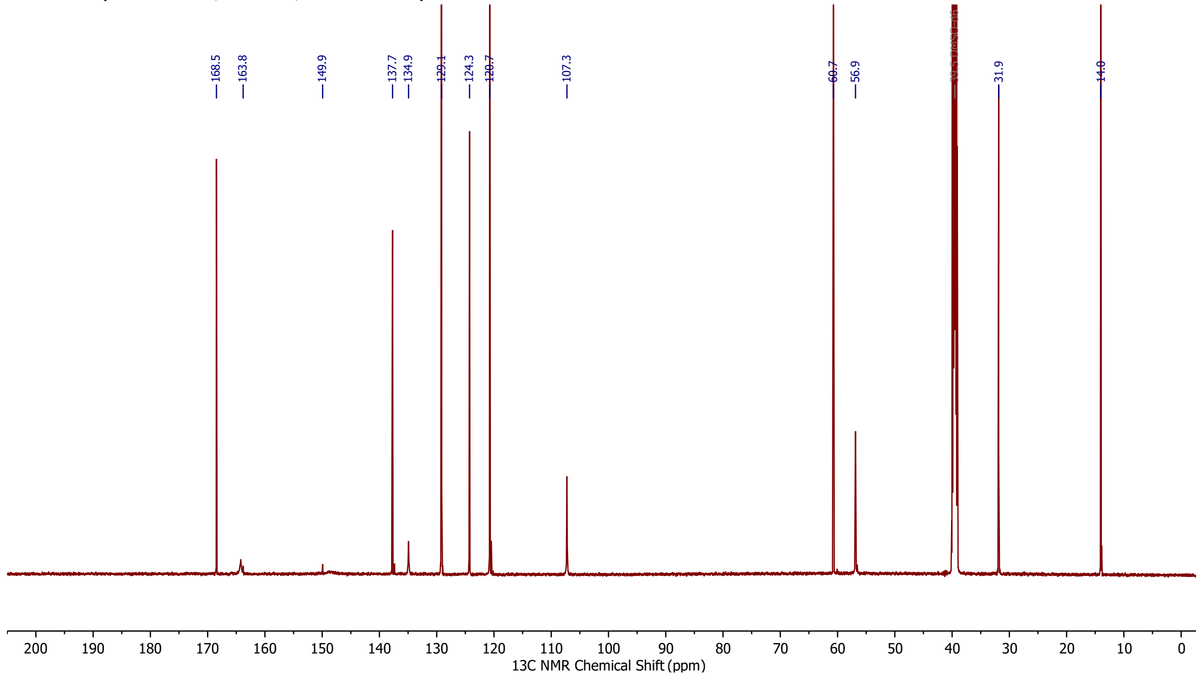

(*Z*)-2-(3-Hydroxy-2-((*N*-methyl-*N*-(methylsulfonyl)glycyl)imino)-2,3-dihydrothiazol-4-yl)acetic acid (21)

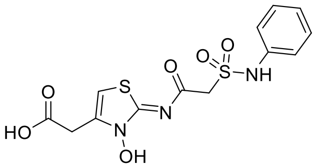

^{1}H NMR (600 MHz, 300 K, DMSO-d_6):

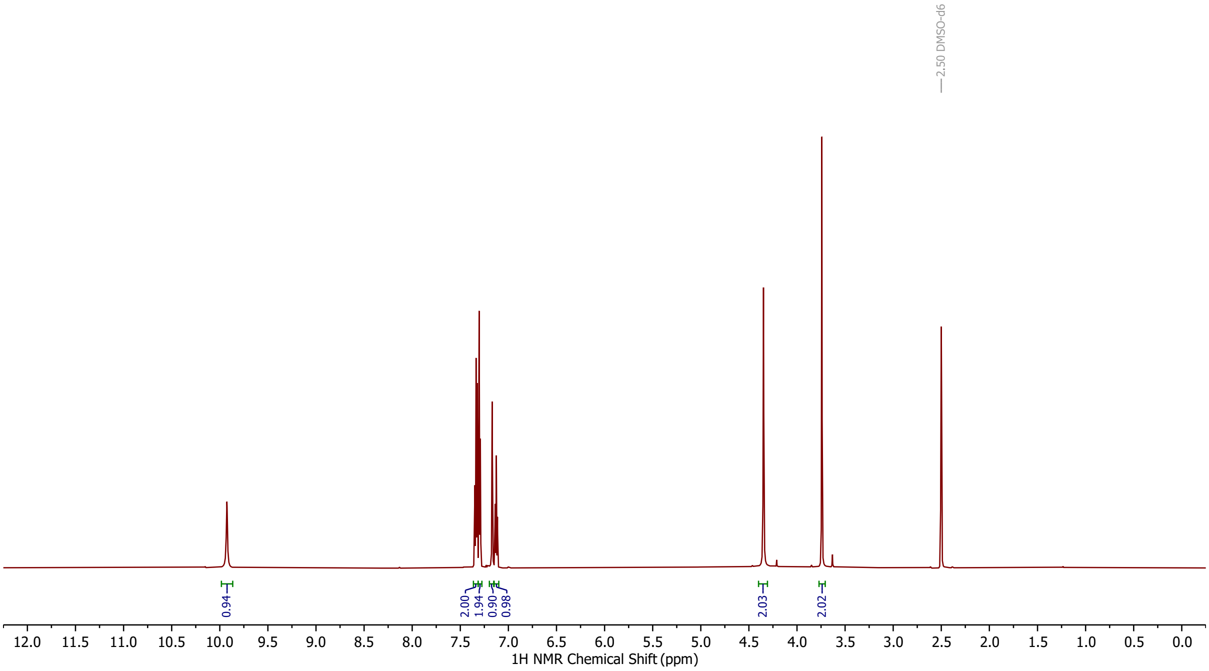

^{13}C NMR (151 MHz, 300 K, DMSO-d_6):

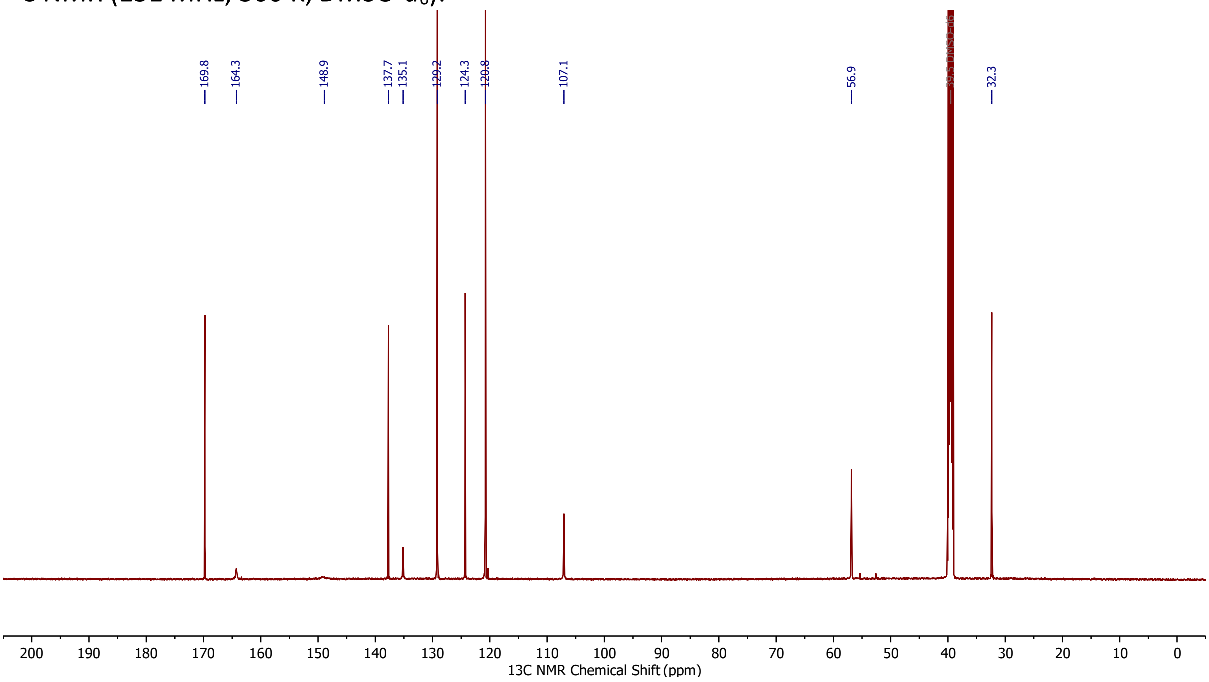

Ethyl 2-(2-(2-phenoxyacetamido)thiazol-4-yl)acetate (22a)

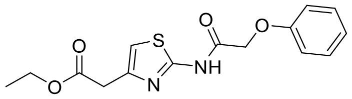

^{1}H NMR (600 MHz, 300 K, $CDCl_3$):

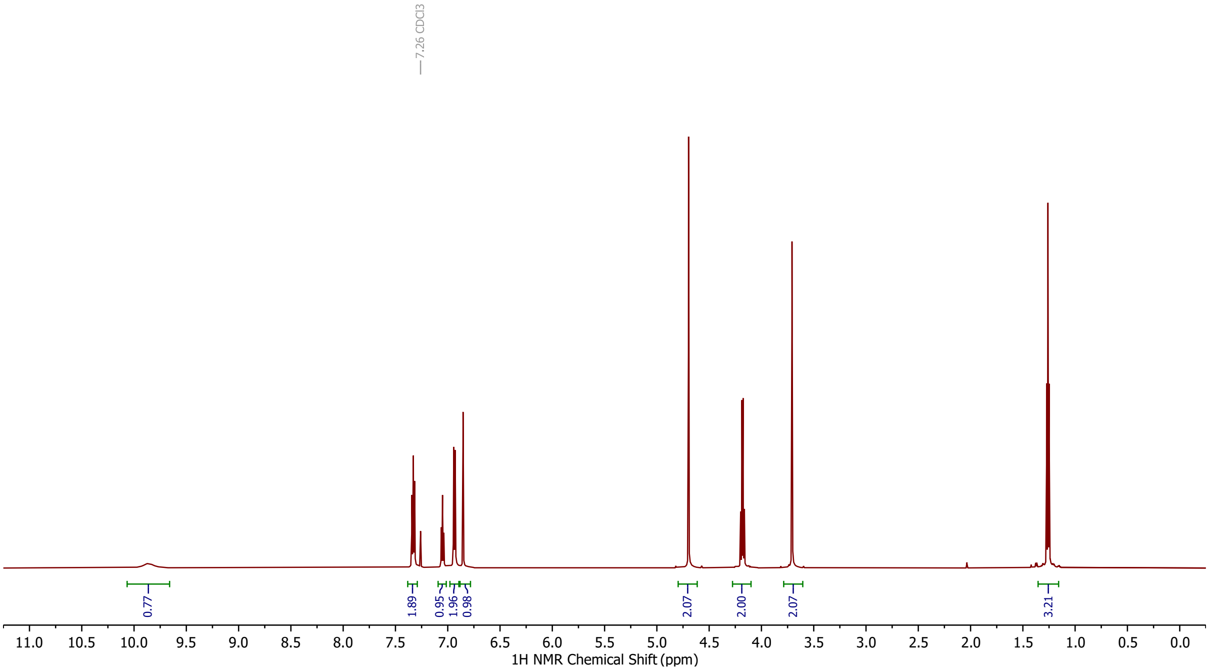

^{13}C NMR (151 MHz, 300 K, $CDCl_3$):

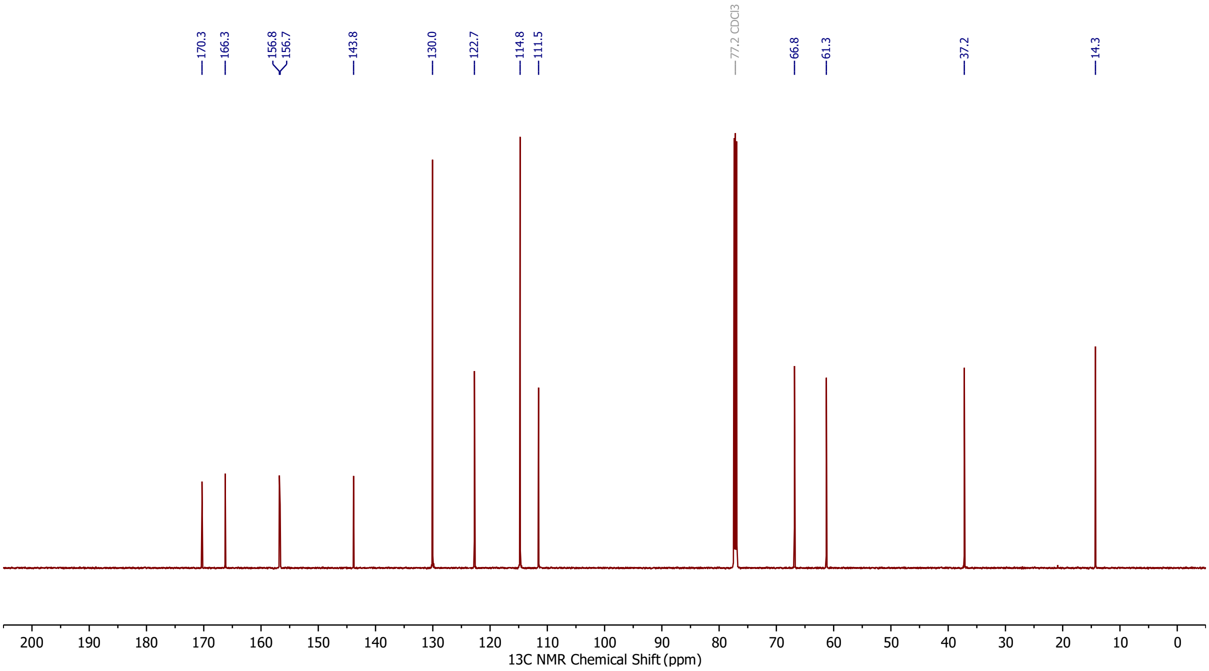

Ethyl (*Z*)-2-(3-hydroxy-2-((2-phenoxyacetyl)imino)-2,3-dihydrothiazol-4-yl)acetate (22b)

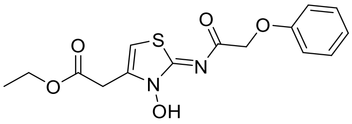

^{1}H NMR (600 MHz, 300 K, $CDCl_3$):

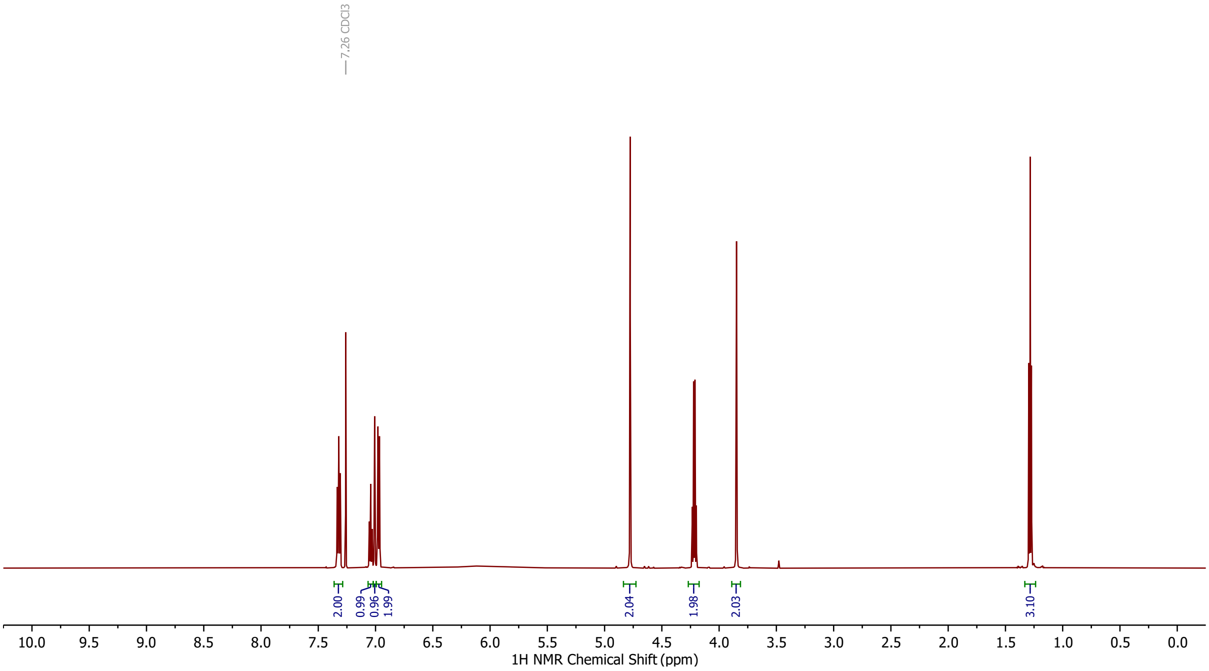

^{13}C NMR (151 MHz, 300 K, $CDCl_3$):

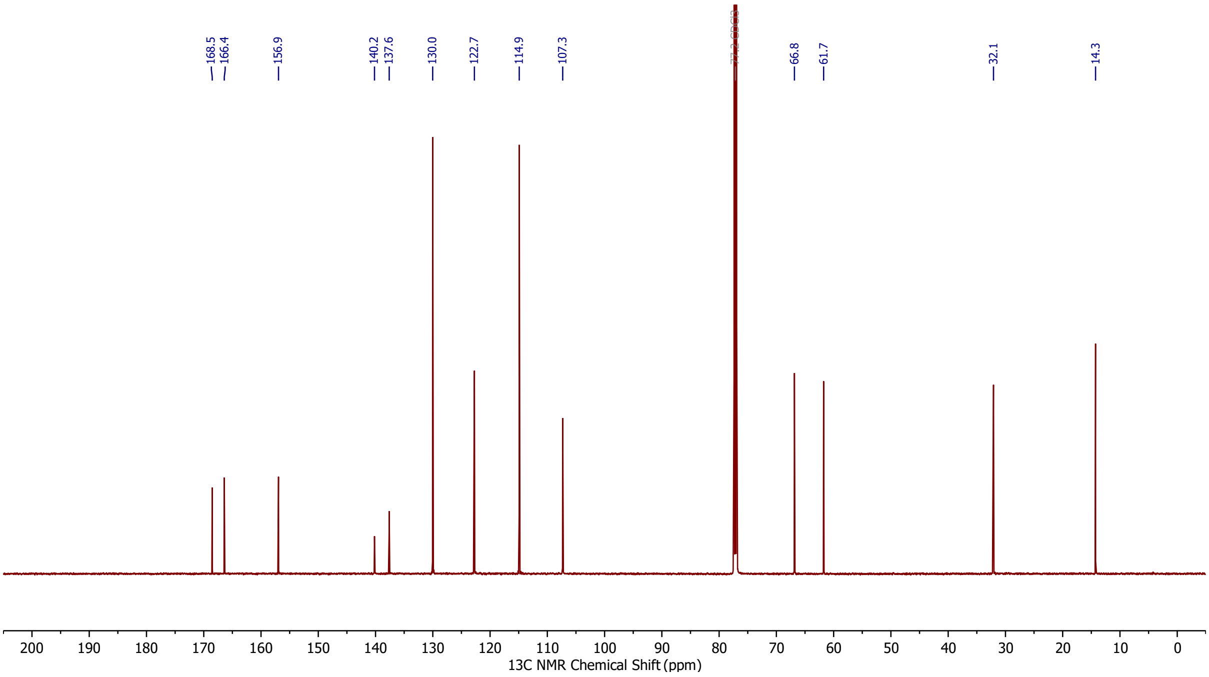

(*Z*)-2-(3-Hydroxy-2-((2-phenoxyacetyl)imino)-2,3-dihydrothiazol-4-yl)acetic acid (22)

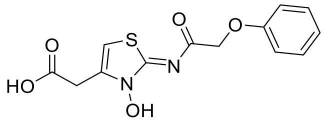

^{1}H NMR (600 MHz, 300 K, DMSO-d_6):

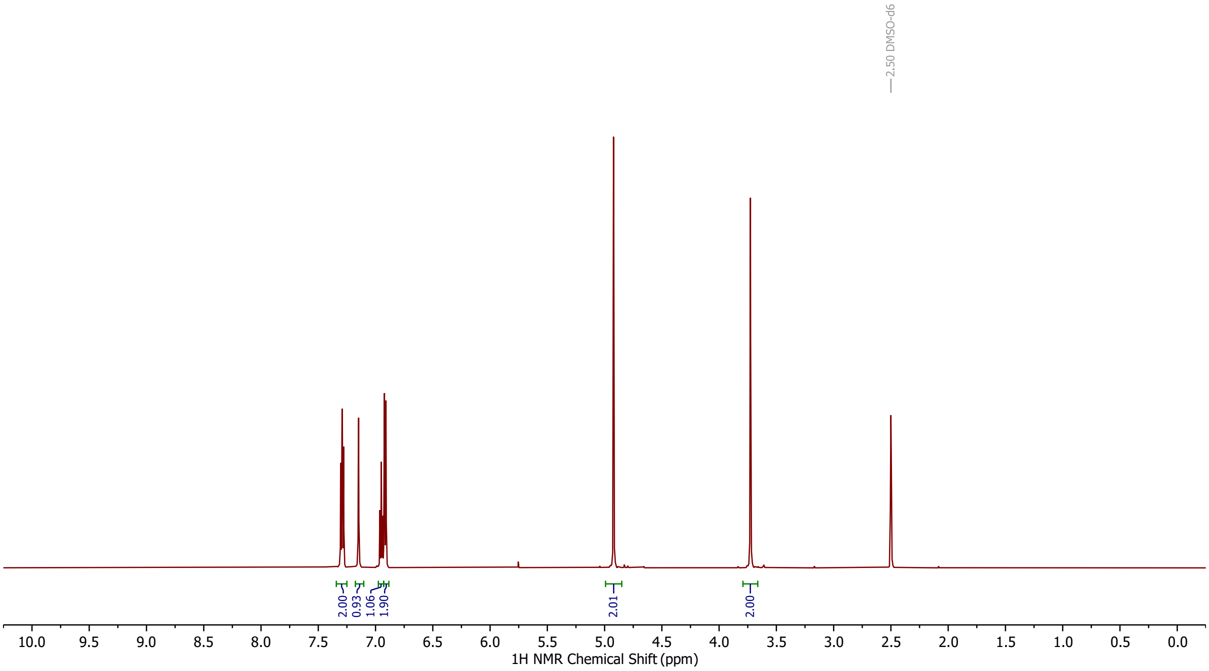

^{13}C NMR (151 MHz, 300 K, DMSO-d_6):

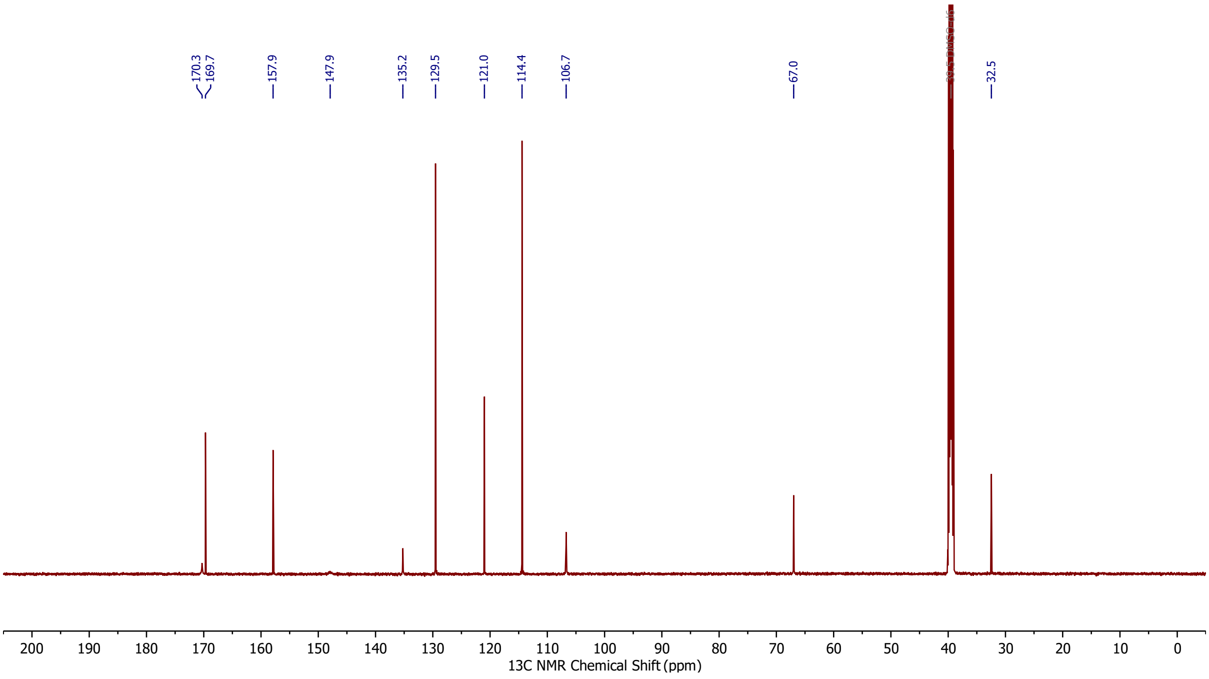

Ethyl 2-(2-(2-((*tert*-butoxycarbonyl)(phenyl)amino)acetamido)thiazol-4-yl)acetate (23a)

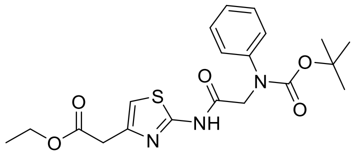

^{1}H NMR (400 MHz, 300 K, $CDCl_3$):

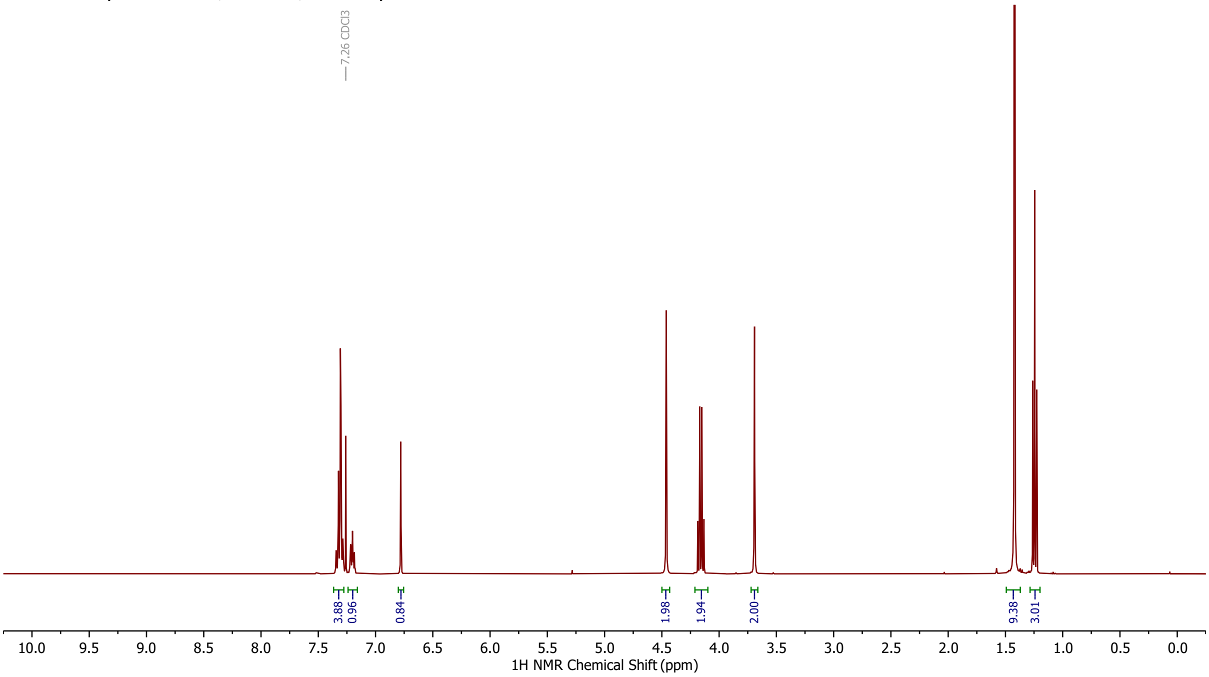

^{13}C NMR (101 MHz, 300 K, $CDCl_3$):

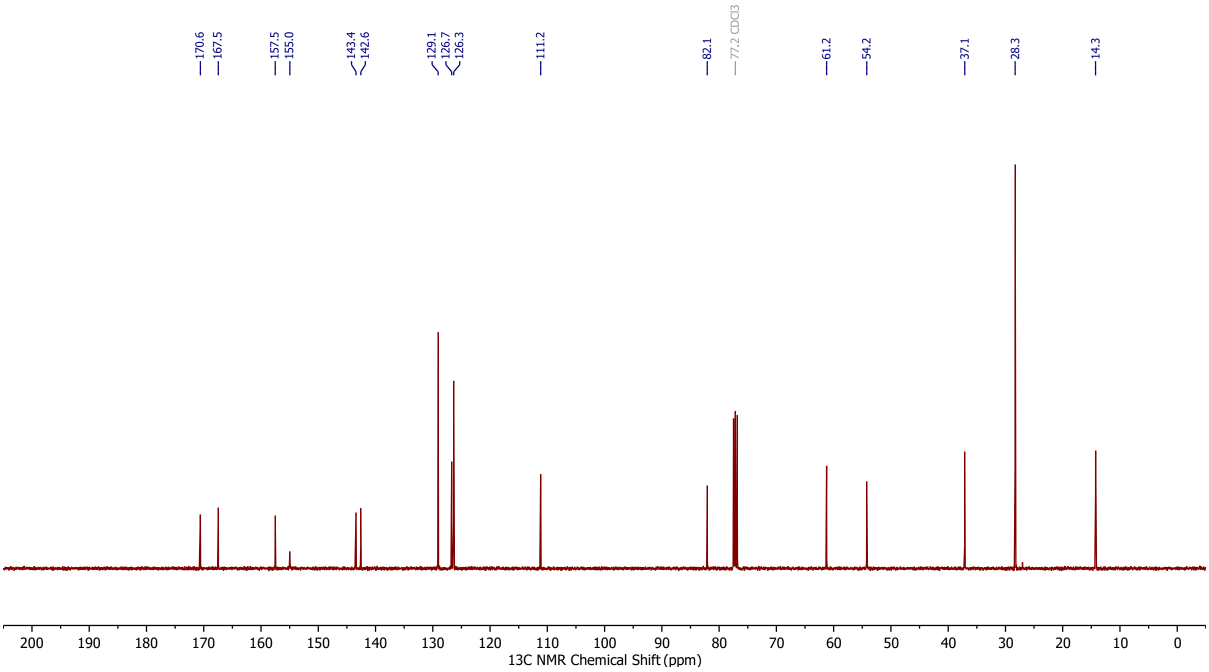

Ethyl (*Z*)-2-(2-((*N*-(tert-butoxycarbonyl)-*N*-phenylglycyl)imino)-3-hydroxy-2,3-dihydrothiazol-4-yl)acetate (23b)

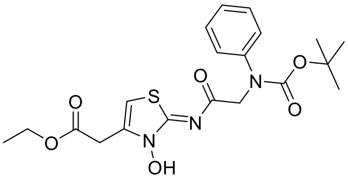

^{1}H NMR (600 MHz, 300 K, $CDCl_3$):

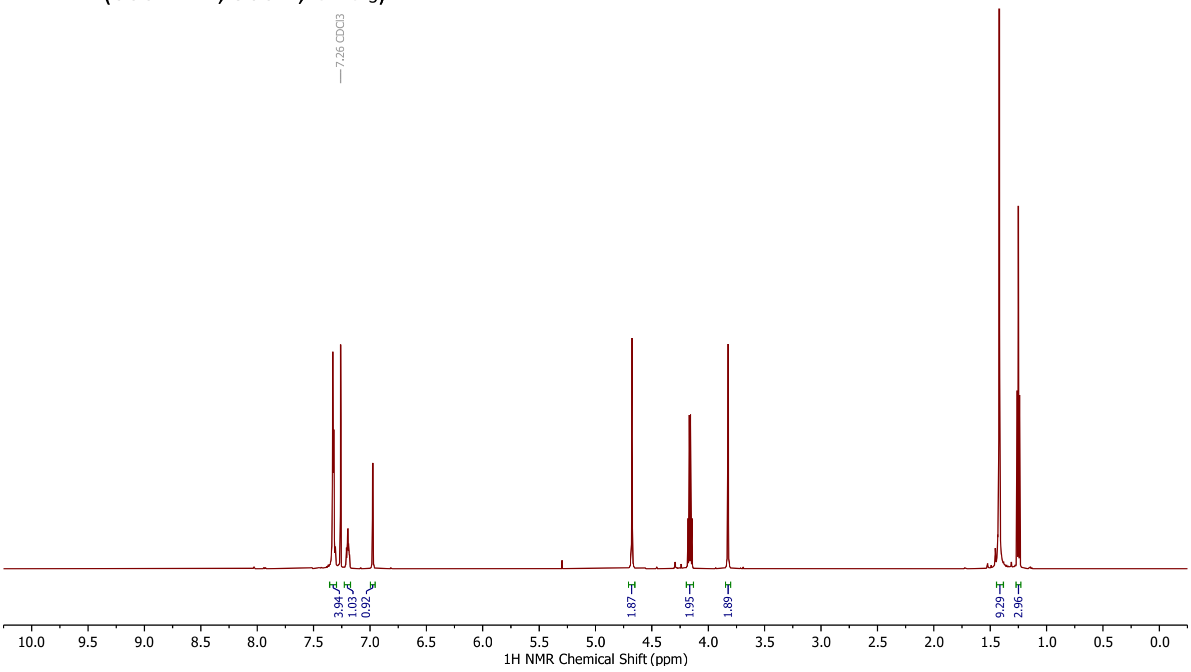

^{13}C NMR (151 MHz, 300 K, $CDCl_3$):

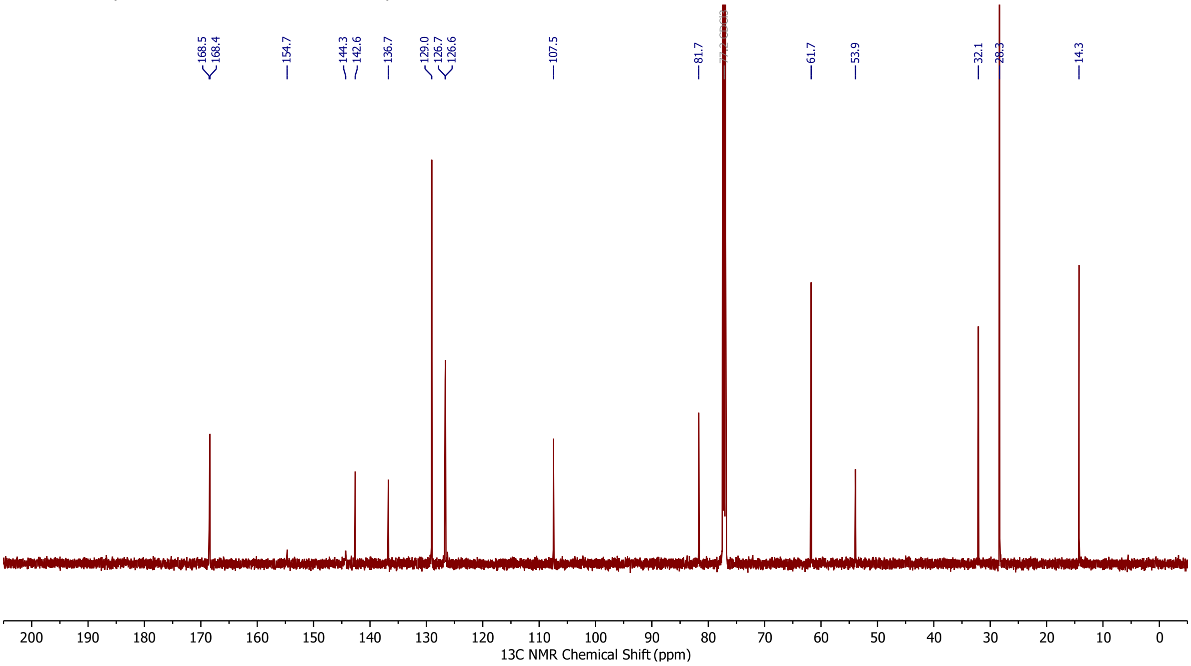

Ethyl (*Z*)-2-(3-hydroxy-2-((phenylglycyl)imino)-2,3-dihydrothiazol-4-yl)acetate (23c)

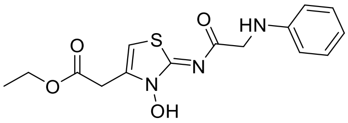

^{1}H NMR (600 MHz, 300 K, $CDCl_3$):

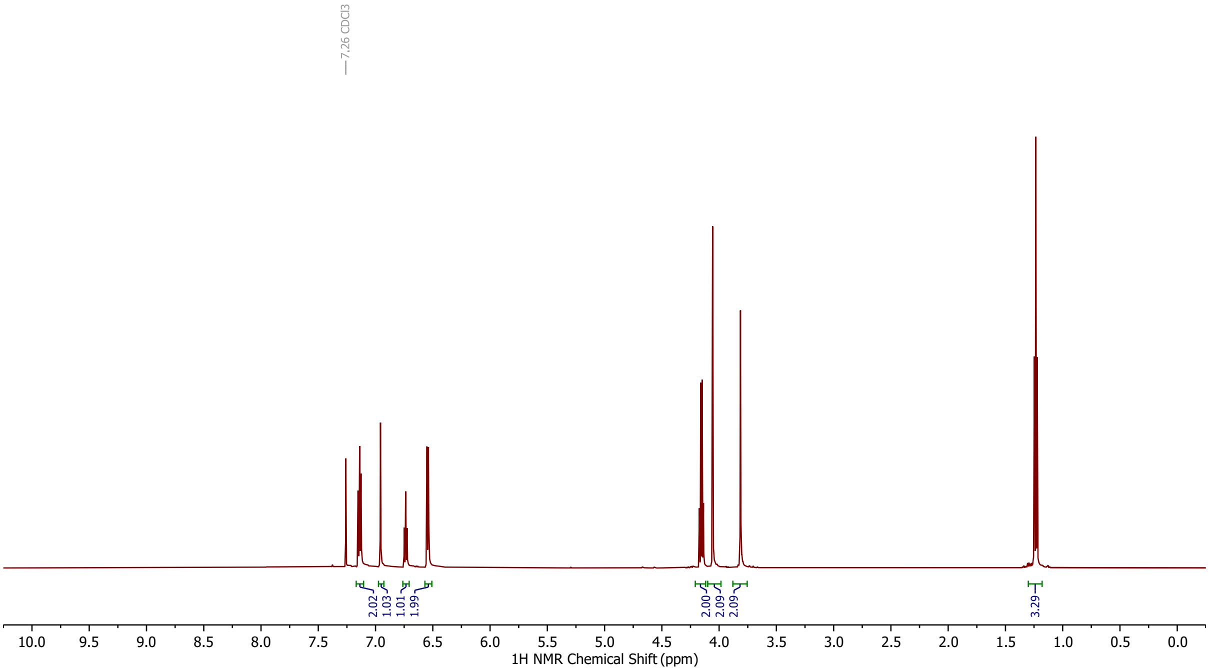

^{13}C NMR (151 MHz, 300 K, $CDCl_3$):

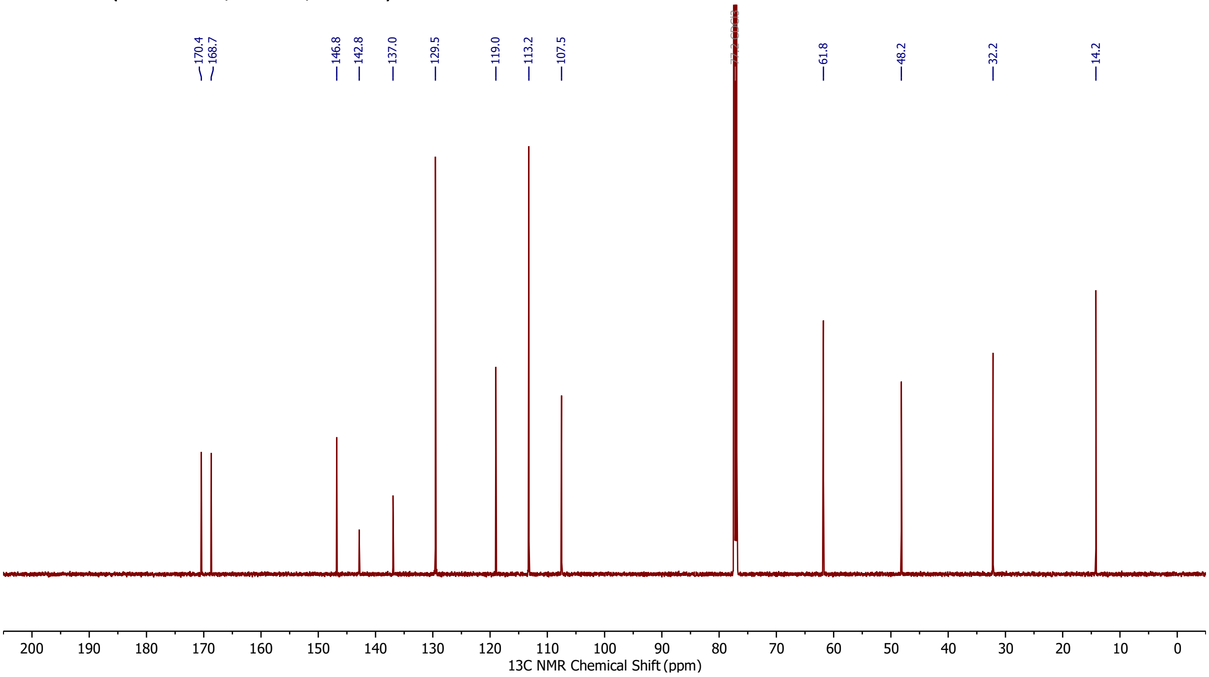

(*Z*)-2-(3-Hydroxy-2-((phenylglycyl)imino)-2,3-dihydrothiazol-4-yl)acetic acid (23)

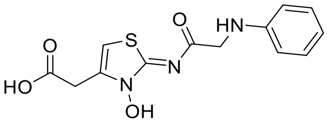

^{1}H NMR (600 MHz, 300 K, DMSO-d_6):

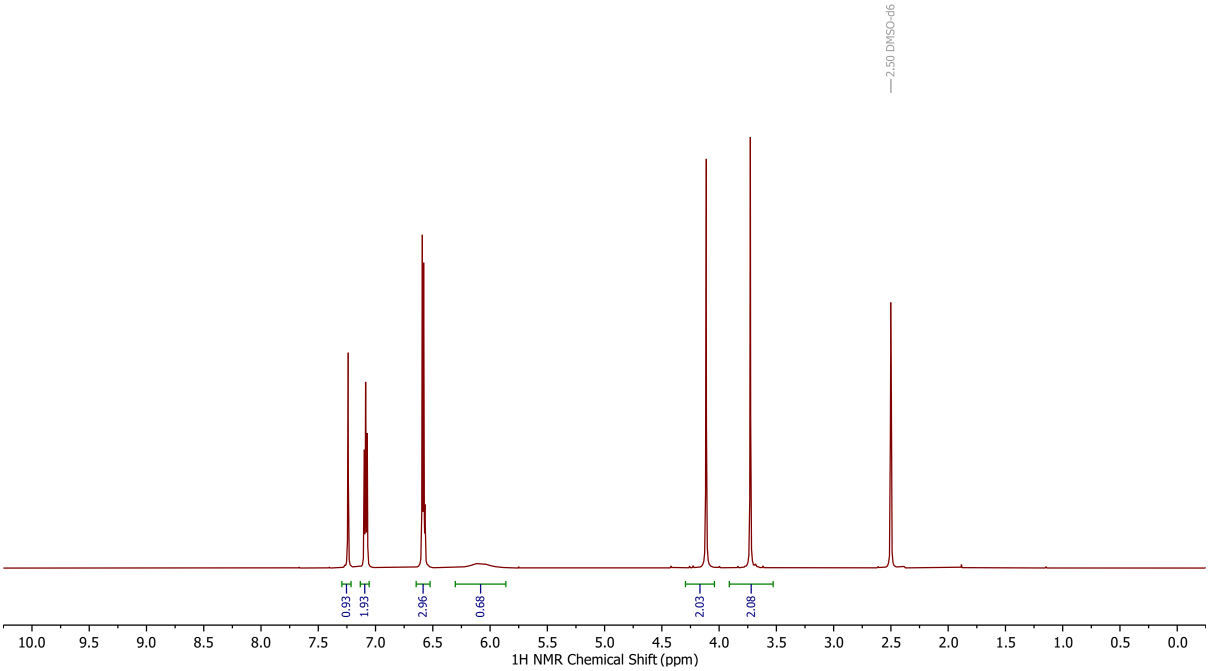

^{13}C NMR (151 MHz, 300 K, DMSO-d_6):

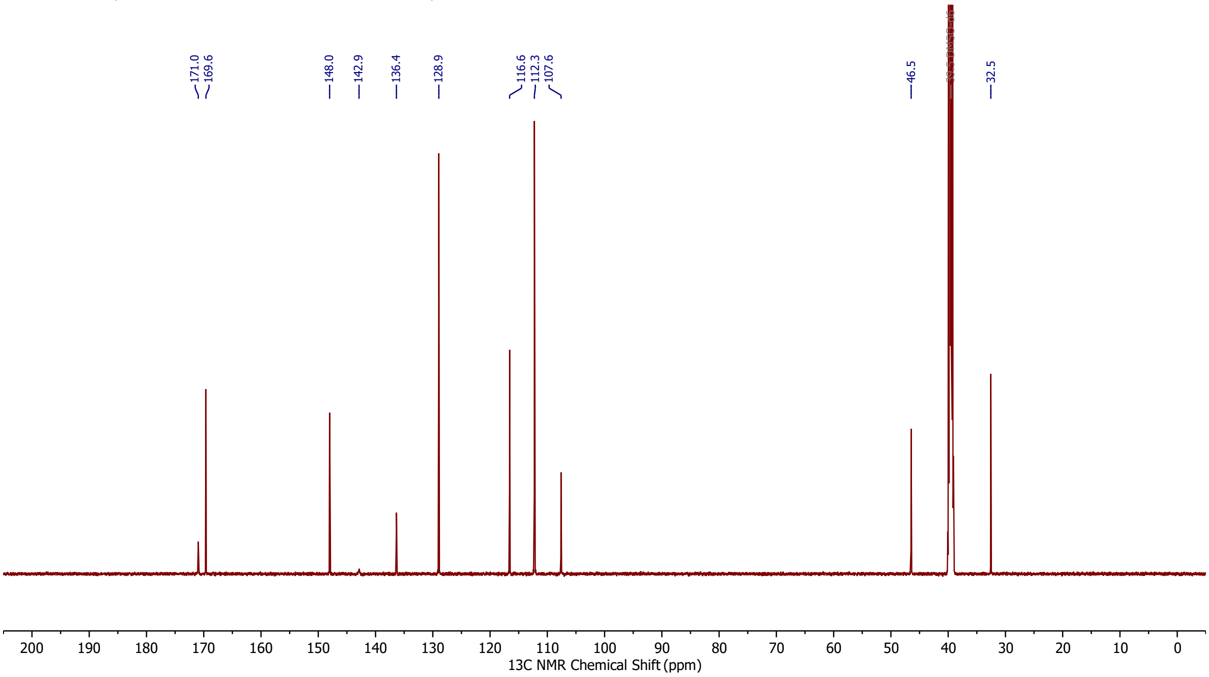

tert-Butyl 3-((4-(2-ethoxy-2-oxoethyl)thiazol-2-yl)carbamoyl)azetidine-1-carboxylate (24a)

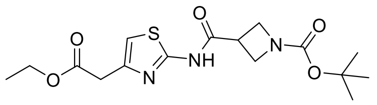

^{1}H NMR (600 MHz, 300 K, $CDCl_3$):

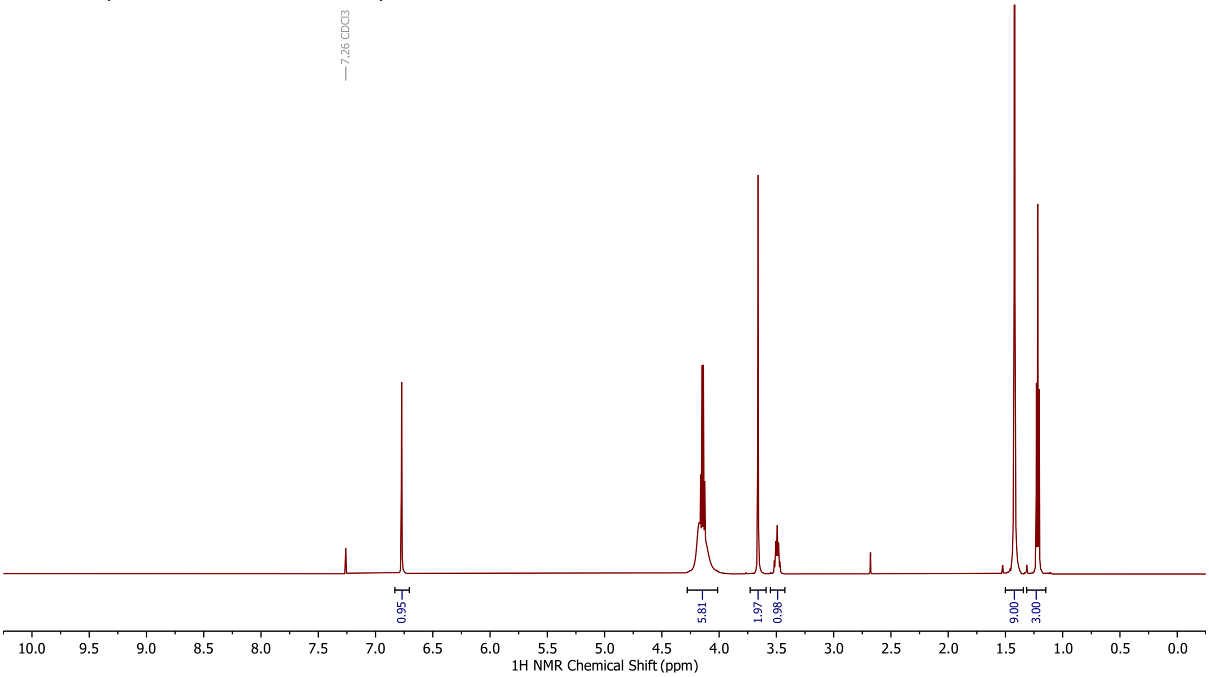

^{13}C NMR (151 MHz, 300 K, $CDCl_3$):

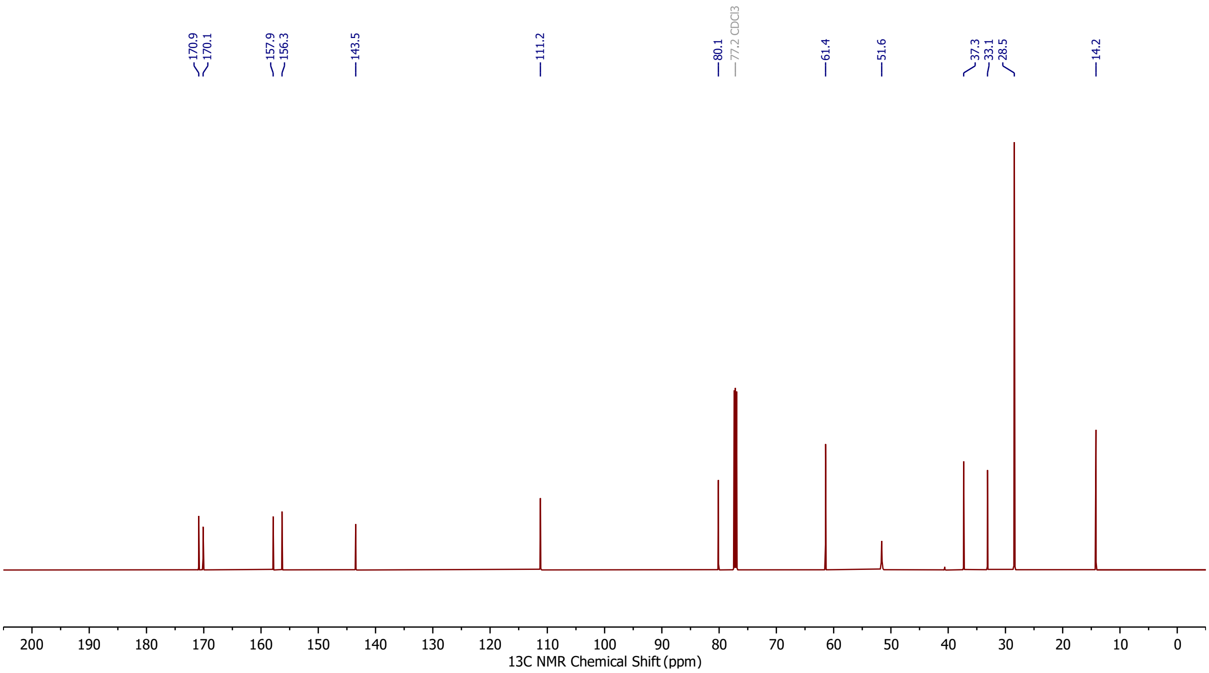

Ethyl 2-(2-(1-(phenylsulfonyl)azetidine-3-carboxamido)thiazol-4-yl)acetate (24c)

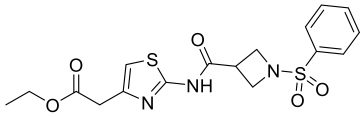

^{1}H NMR (600 MHz, 300 K, $CDCl_3$):

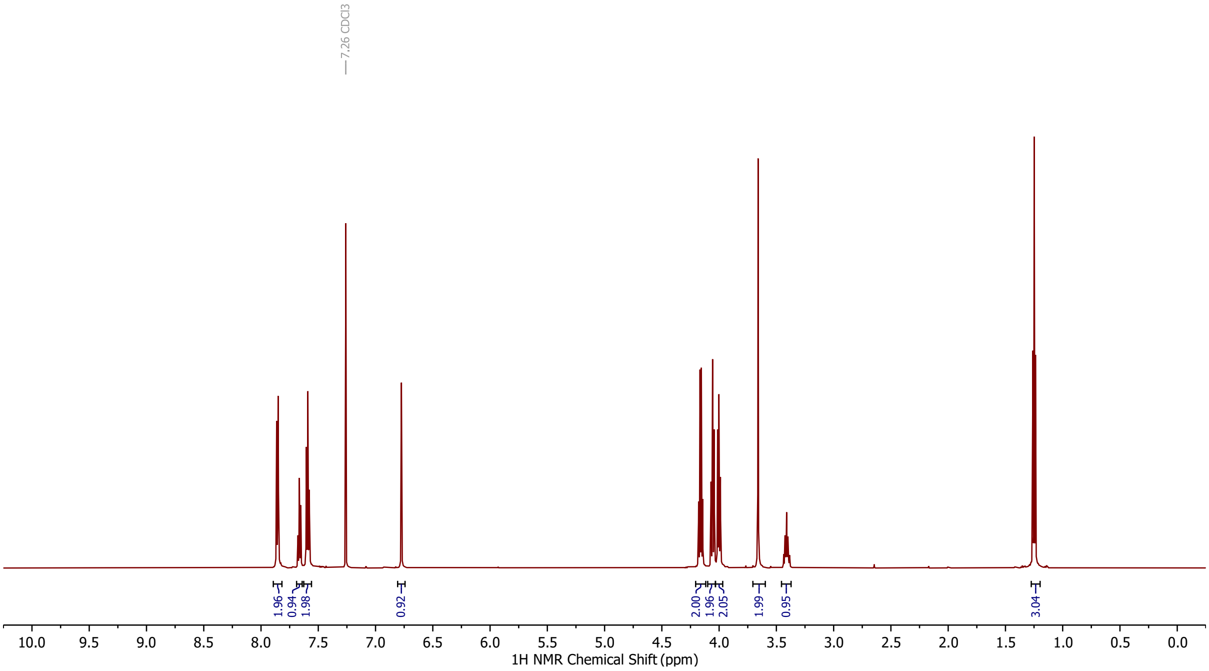

^{13}C NMR (151 MHz, 300 K, $CDCl_3$):

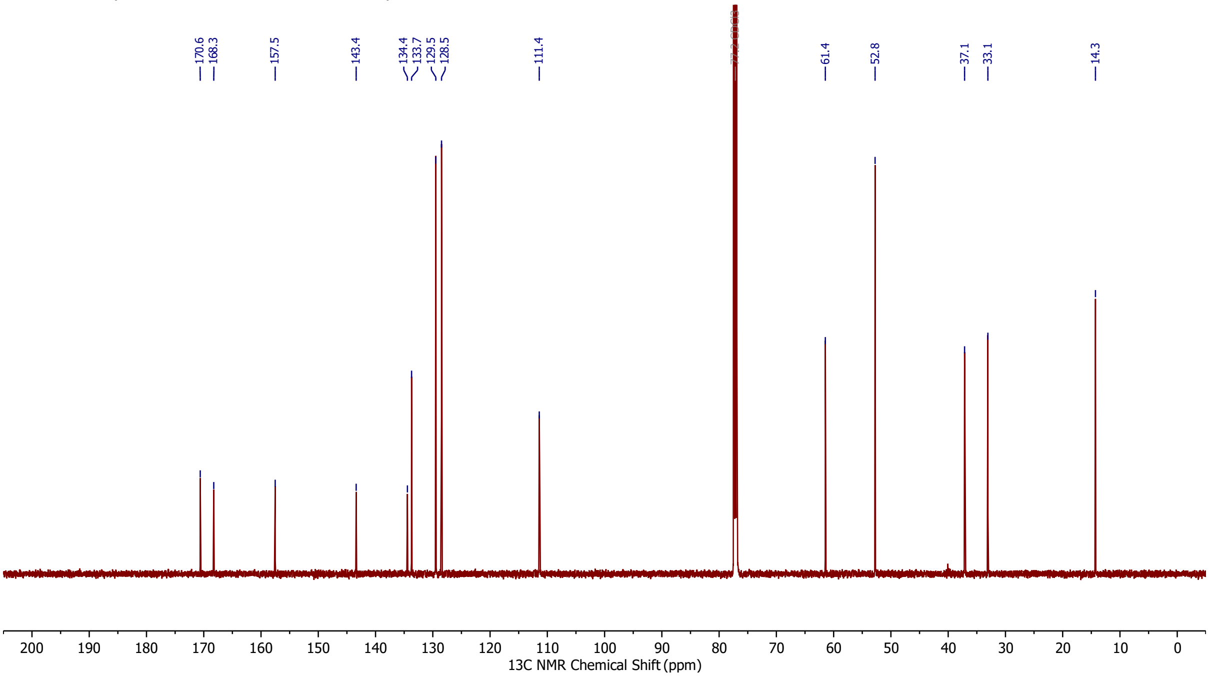

Ethyl (*Z*)-2-(3-hydroxy-2-((1-(phenylsulfonyl)azetidine-3-carbonyl)imino)-2,3-dihydrothiazol-4-yl)acetate (24d)

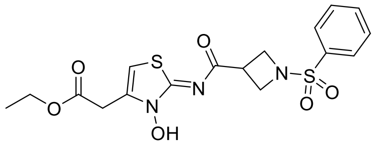

^{1}H NMR (600 MHz, 300 K, DMSO-d_6):

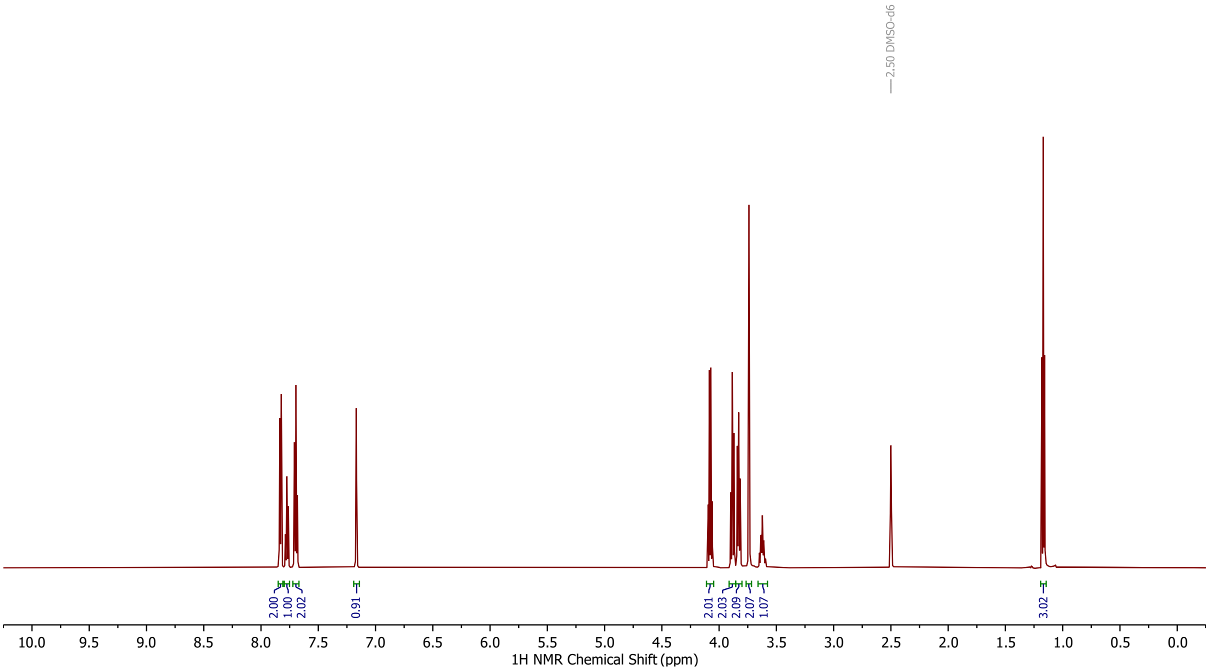

^{13}C NMR (151 MHz, 300 K, DMSO-d_6):

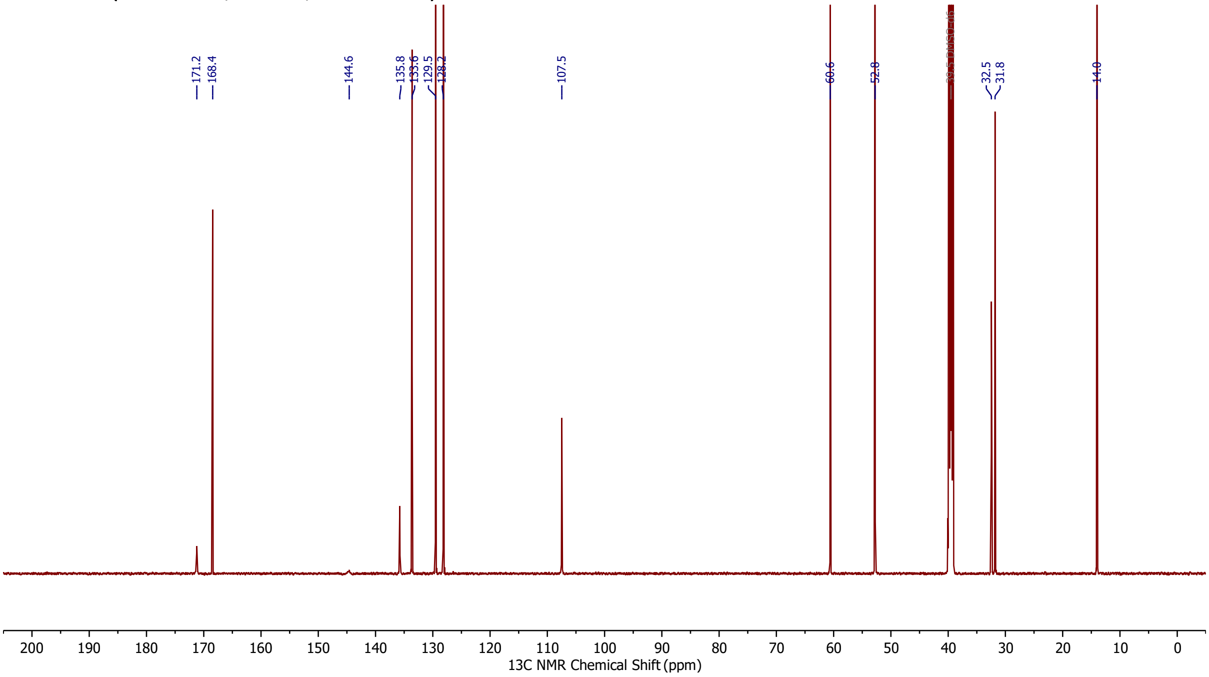

(*Z*)-2-(3-Hydroxy-2-((1-(phenylsulfonyl)azetidine-3-carbonyl)imino)-2,3-dihydrothiazol-4-yl)acetic acid (24)

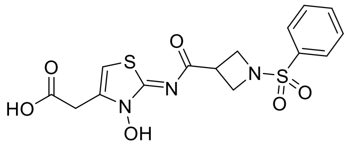

^{1}H NMR (600 MHz, 300 K, DMSO-d_6):

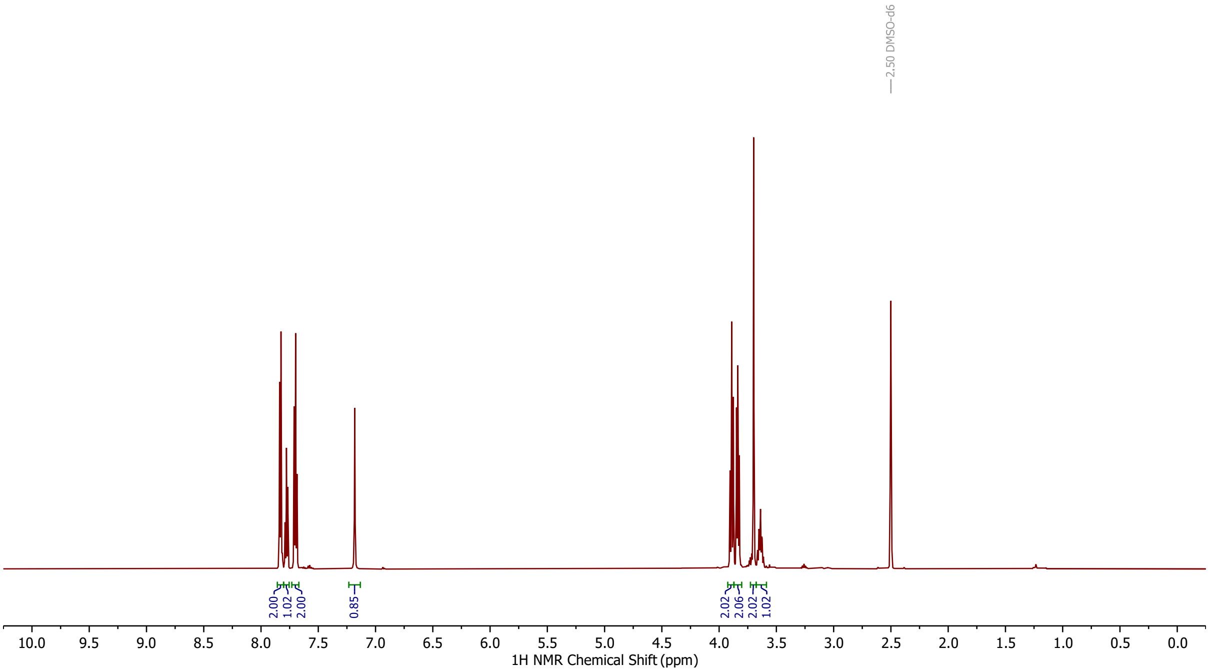

^{13}C NMR (151 MHz, 300 K, DMSO-d_6):

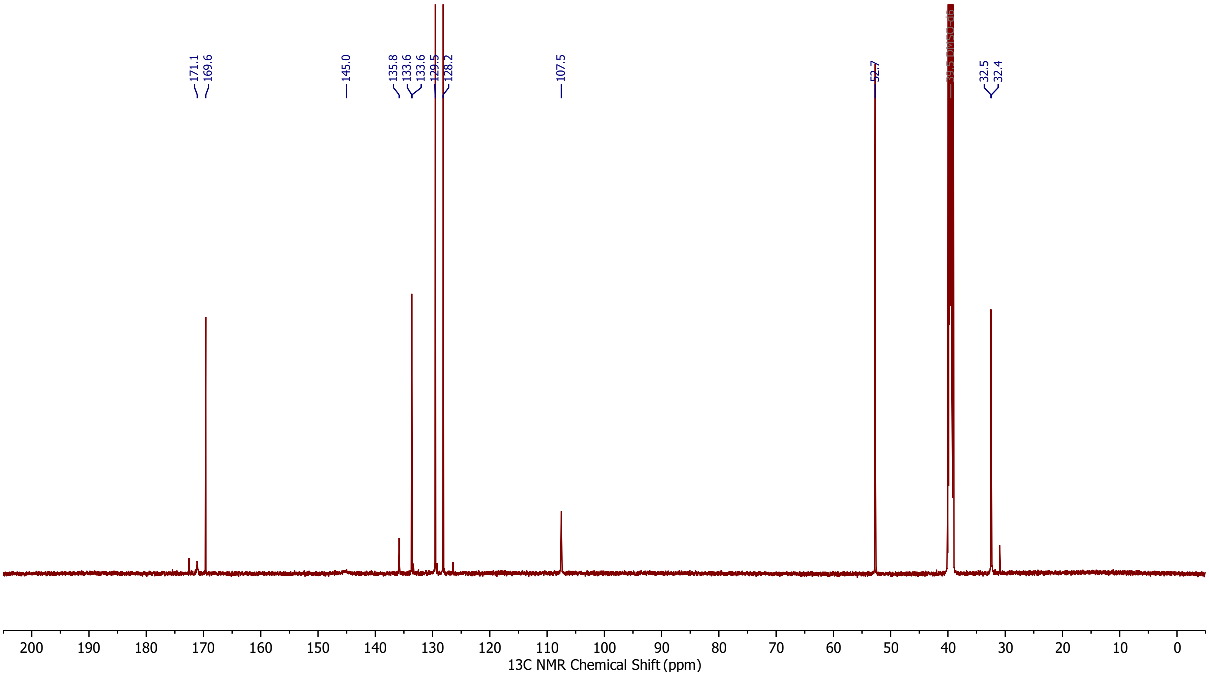

***tert*-Butyl 3-((4-(2-ethoxy-2-oxoethyl)thiazol-2-yl)carbamoyl)-3-methylazetidine-1-carboxylate (25a)**

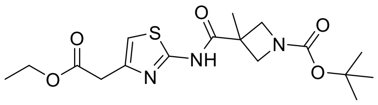

^{1}H NMR (600 MHz, 300 K, $CDCl_3$):

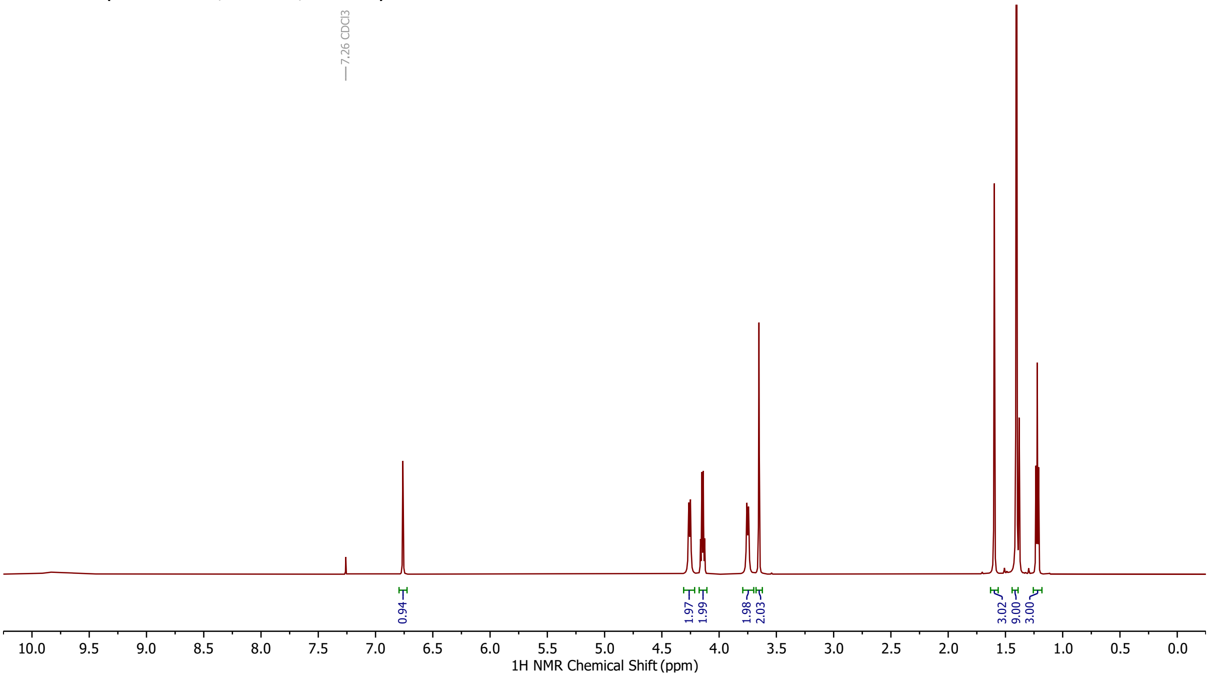

^{13}C NMR (151 MHz, 300 K, $CDCl_3$):

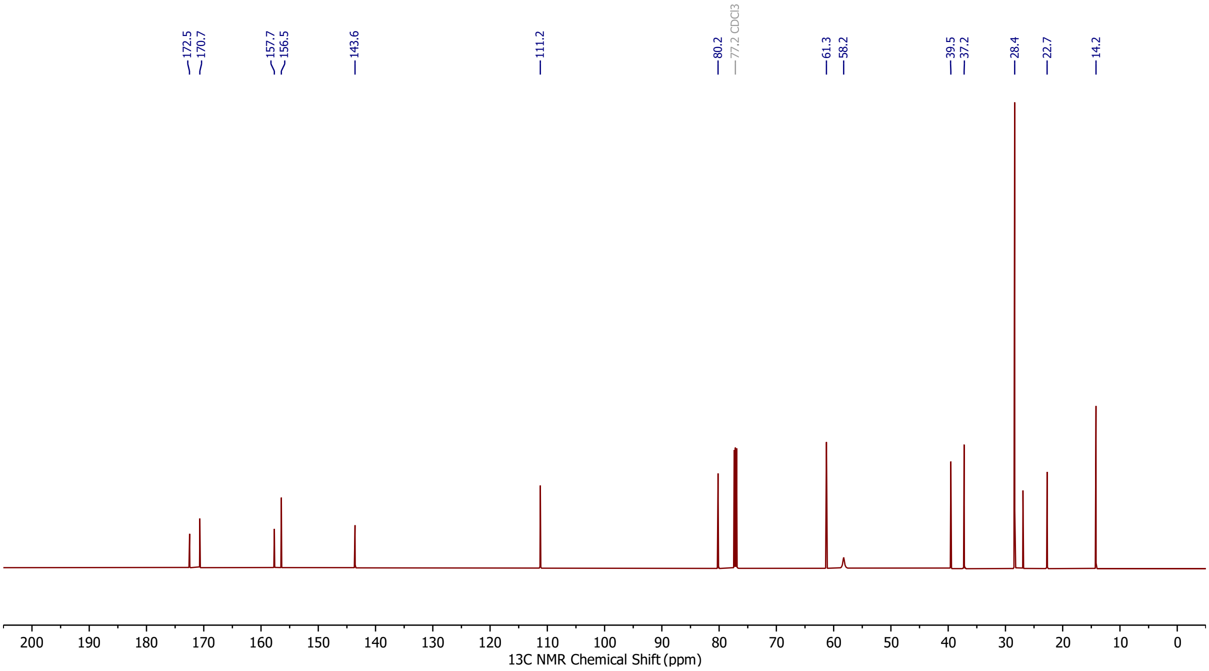

Ethyl 2-(2-(3-methyl-1-(phenylsulfonyl)azetidine-3-carboxamido)thiazol-4-yl)acetate (25c)

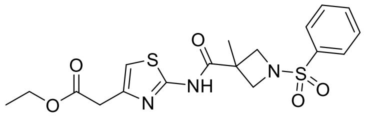

^{1}H NMR (600 MHz, 300 K, $CDCl_3$):

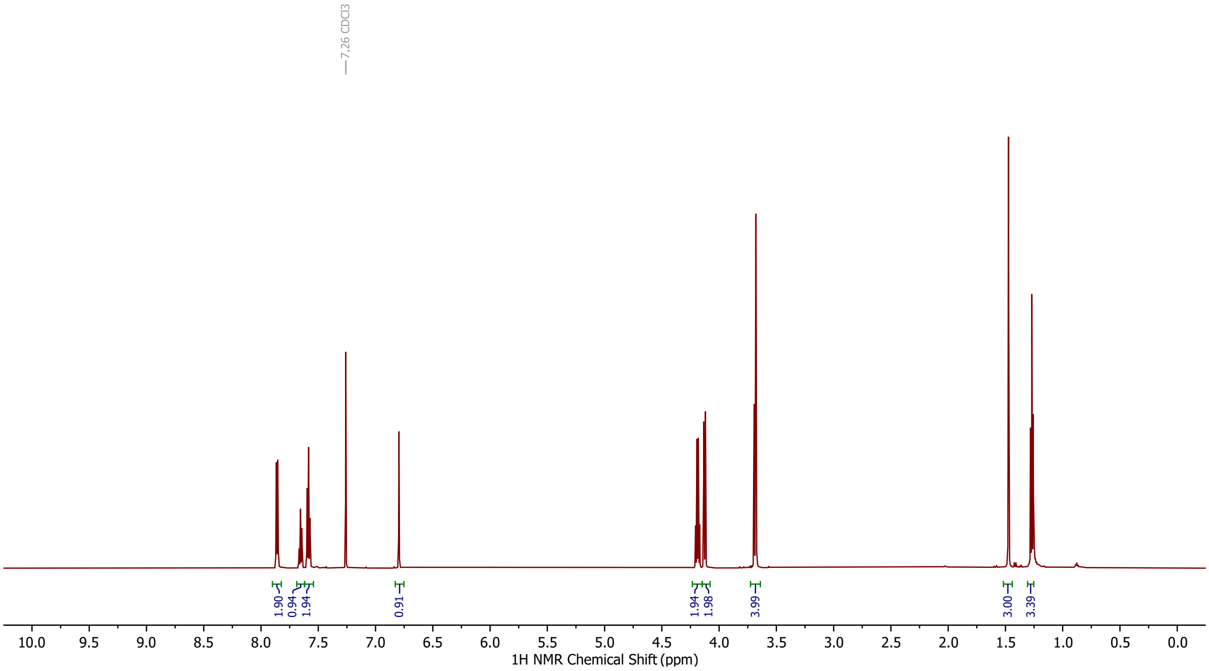

^{13}C NMR (151 MHz, 300 K, $CDCl_3$):

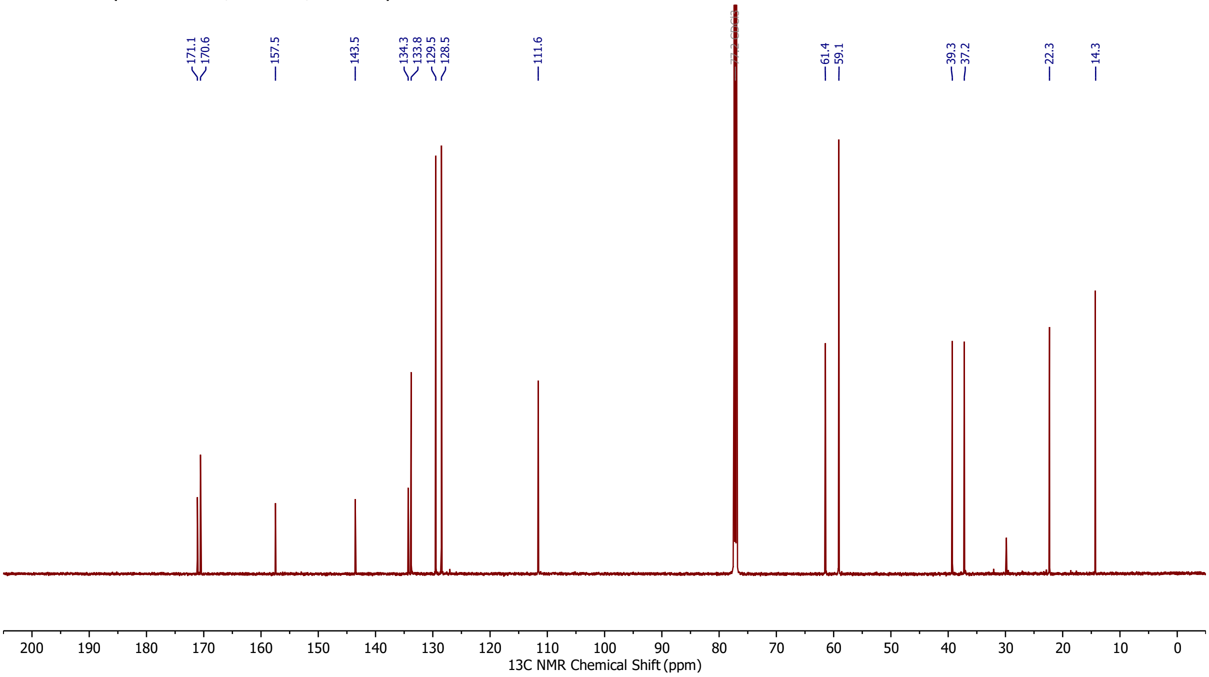

Ethyl (*Z*)-2-(3-hydroxy-2-((3-methyl-1-(phenylsulfonyl)azetidine-3-carbonyl)imino)-2,3-dihydrothiazol-4-yl)acetate (25d)

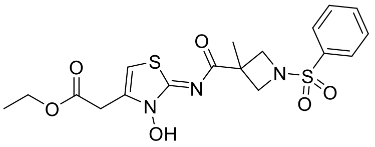

^{1}H NMR (600 MHz, 300 K, DMSO-d_6):

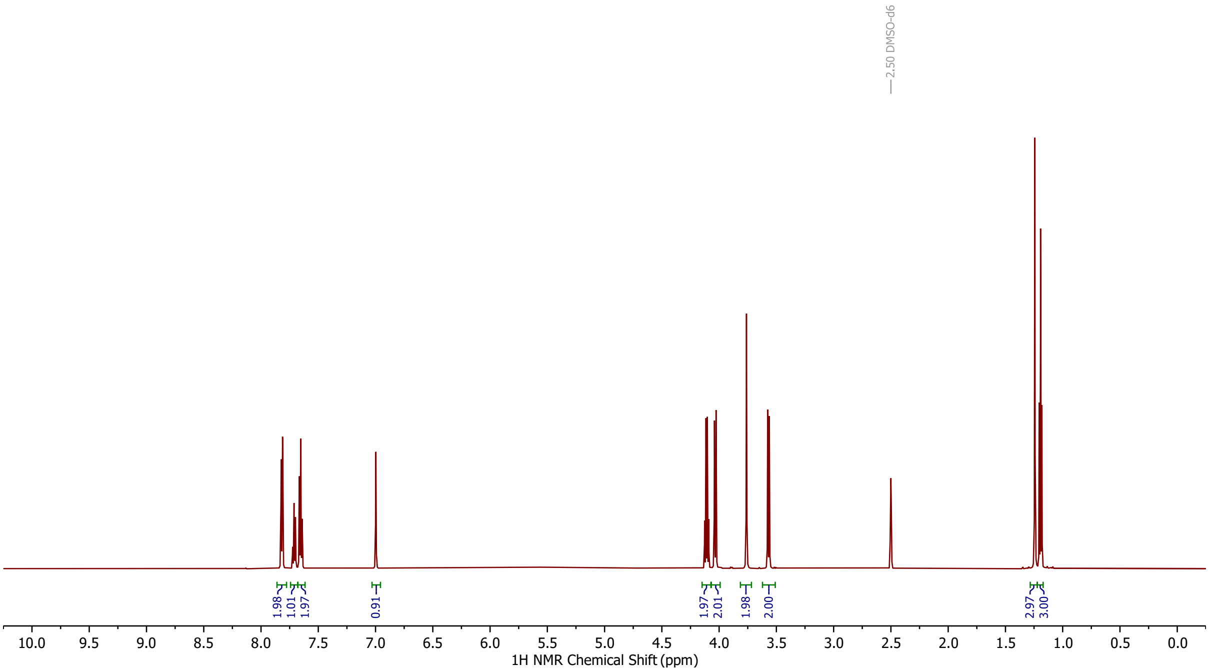

^{13}C NMR (151 MHz, 300 K, DMSO-d_6):

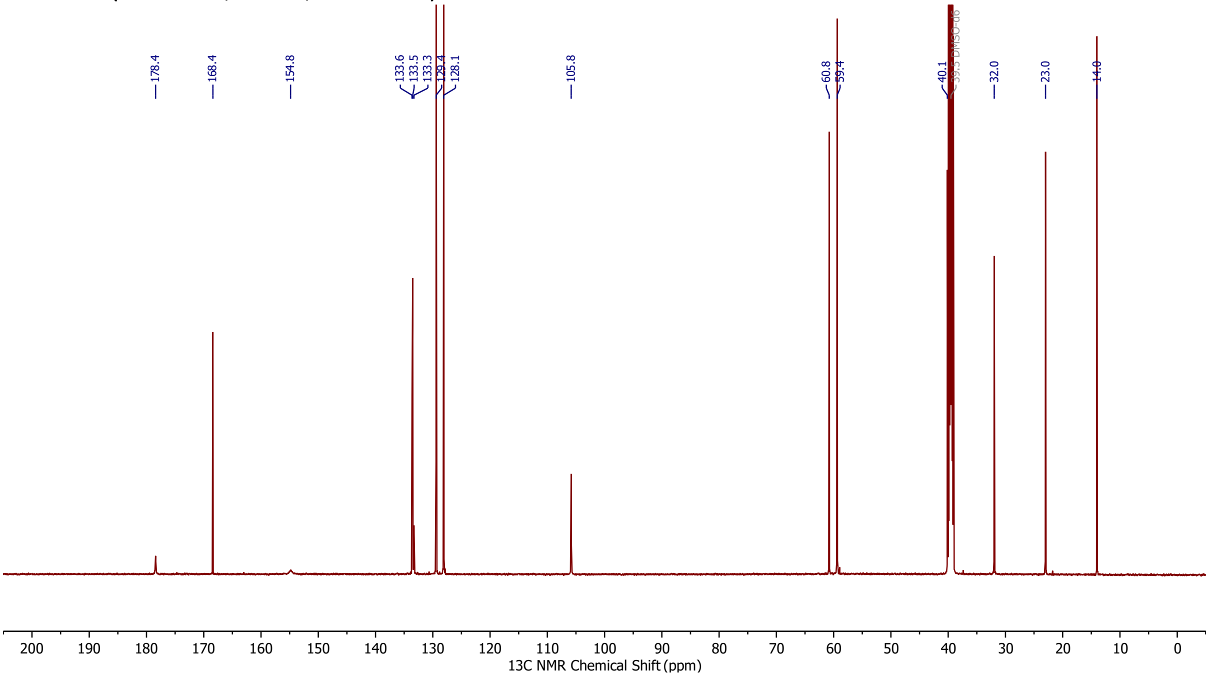

(*Z*)-2-(3-Hydroxy-2-((3-methyl-1-(phenylsulfonyl)azetidine-3-carbonyl)imino)-2,3-dihydrothiazol-4-yl)acetic acid (25)

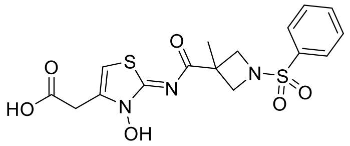

^{1}H NMR (600 MHz, 300 K, DMSO-d_6):

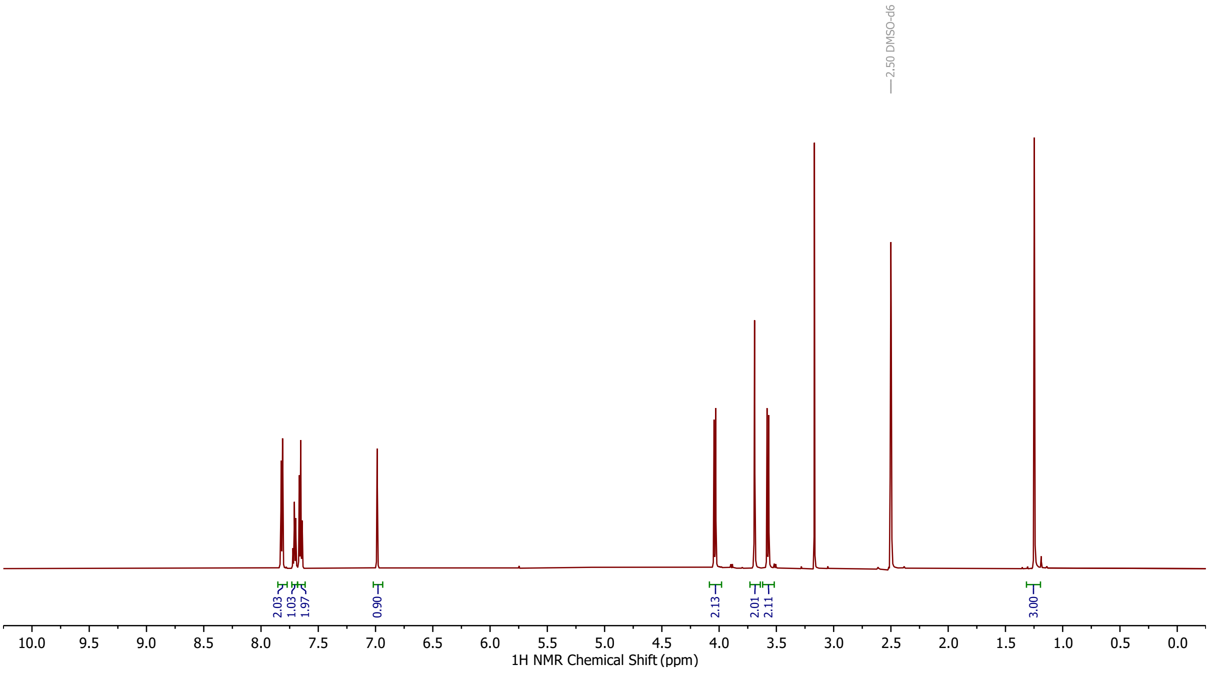

^{13}C NMR (151 MHz, 300 K, DMSO-d_6):

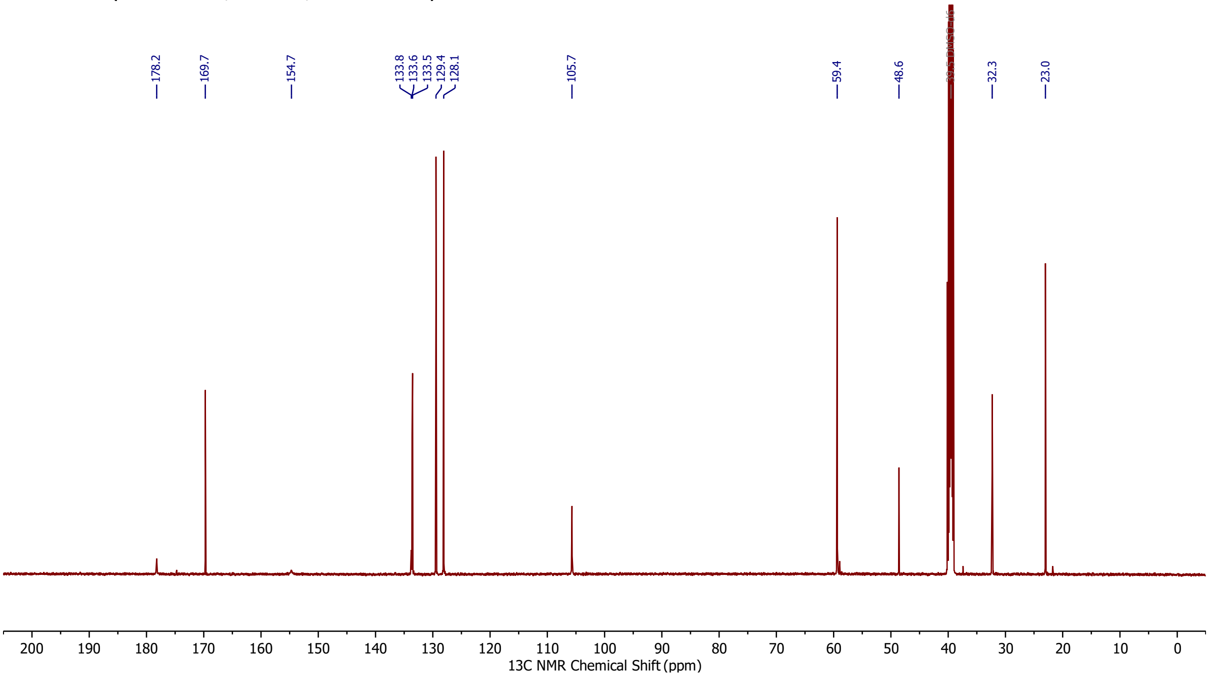

(±)-Ethyl 2-(2-(1-(phenylsulfonyl)pyrrolidine-3-carboxamido)thiazol-4-yl)acetate (26a)

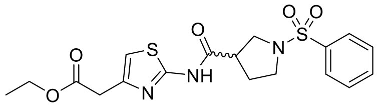

^{1}H NMR (600 MHz, 300 K, $CDCl_3$):

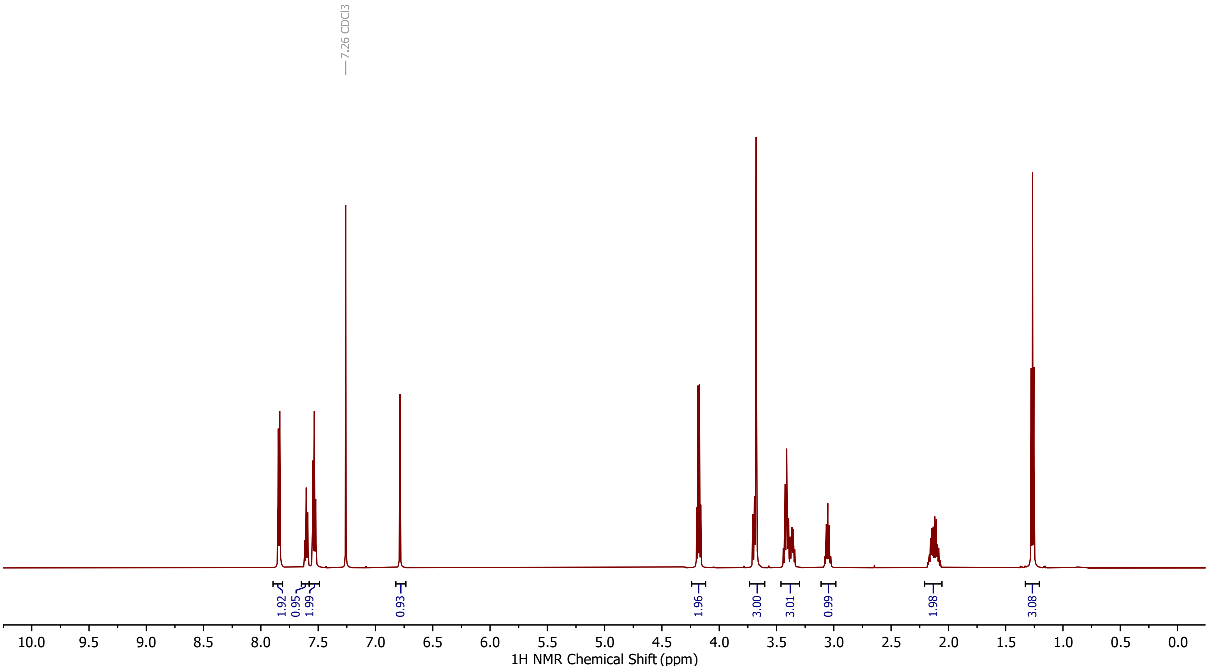

^{13}C NMR (151 MHz, 300 K, $CDCl_3$):

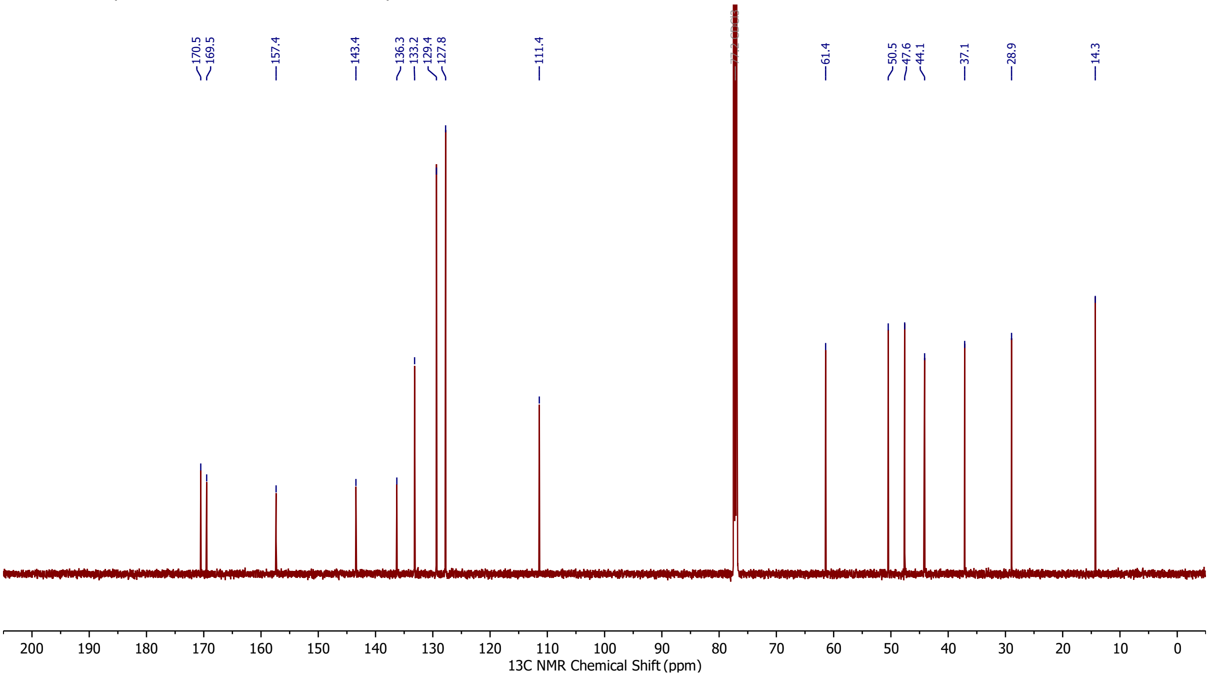

(±)-Ethyl (*Z*)-2-(3-hydroxy-2-((1-(phenylsulfonyl)pyrrolidine-3-carbonyl) imino)-2,3-dihydrothiazol-4-yl)acetate (26b)

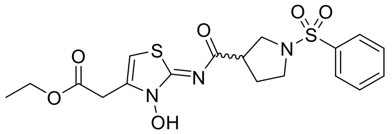

^{1}H NMR (600 MHz, 300 K, DMSO-d_6):

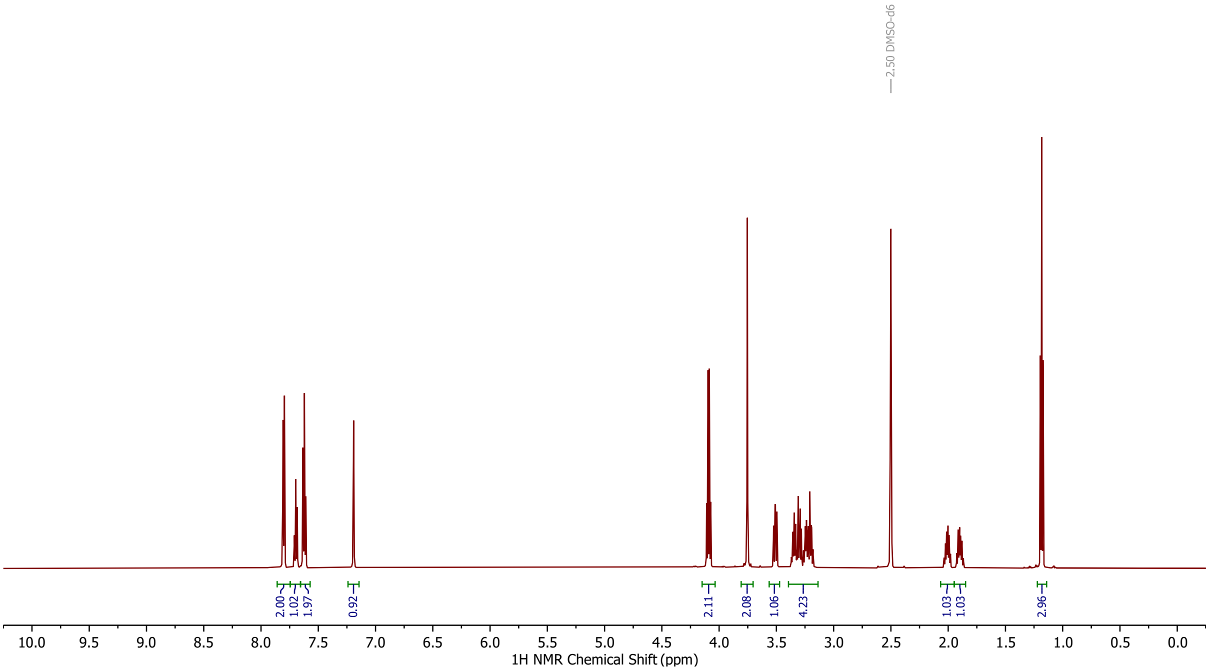

^{13}C NMR (151 MHz, 300 K, DMSO-d_6):

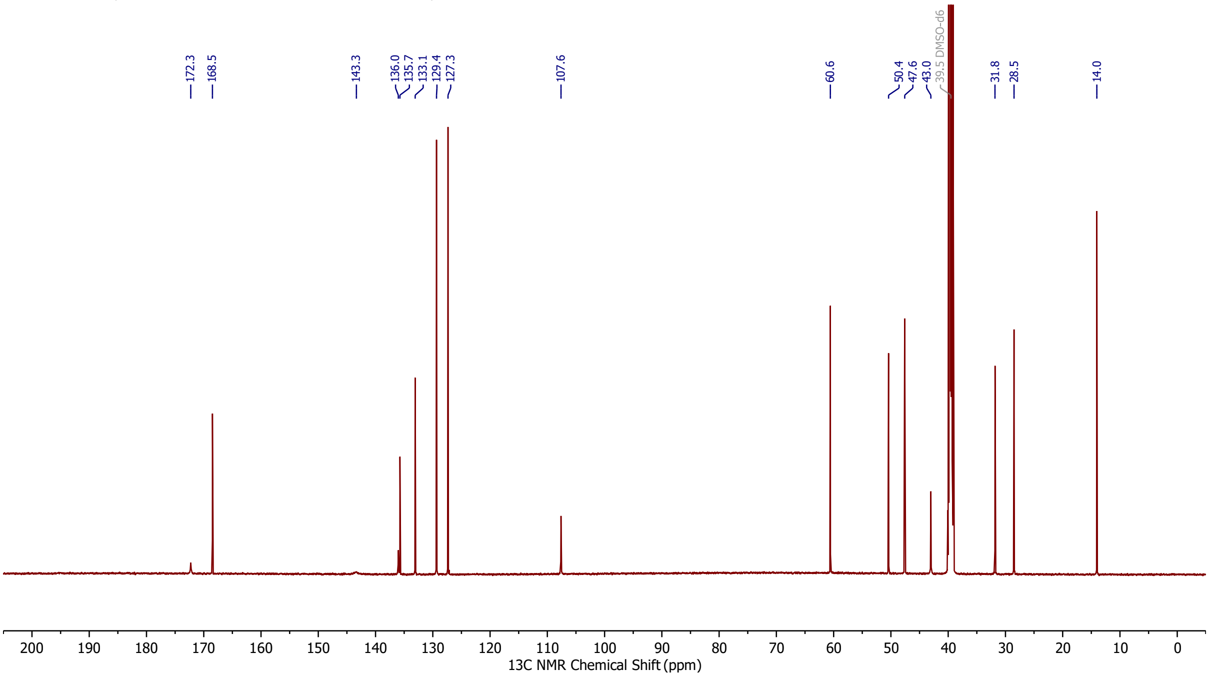

(±)-(*Z*)-2-(3-Hydroxy-2-((1-(phenylsulfonyl)pyrrolidine-3-carbonyl)imino)-2,3-dihydrothiazol-4-yl)acetic acid (26)

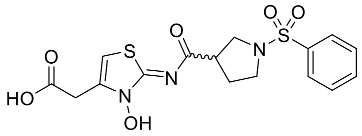

^{1}H NMR (600 MHz, 300 K, DMSO-d_6):

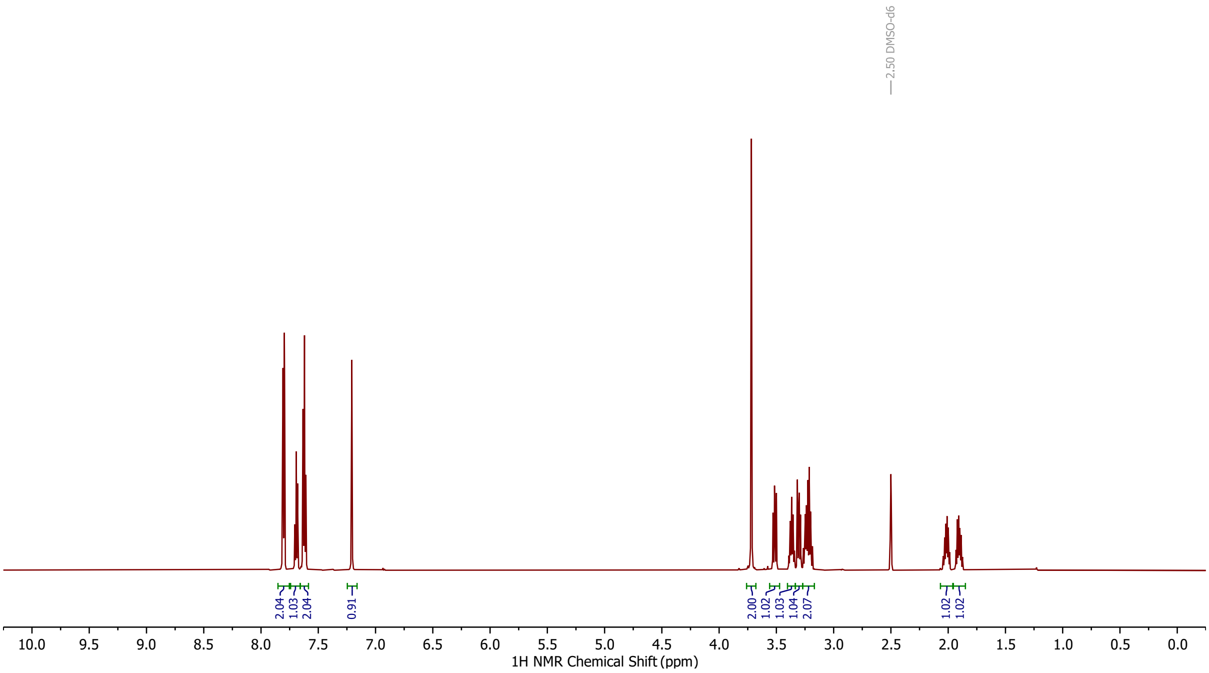

^{13}C NMR (151 MHz, 300 K, DMSO-d_6):

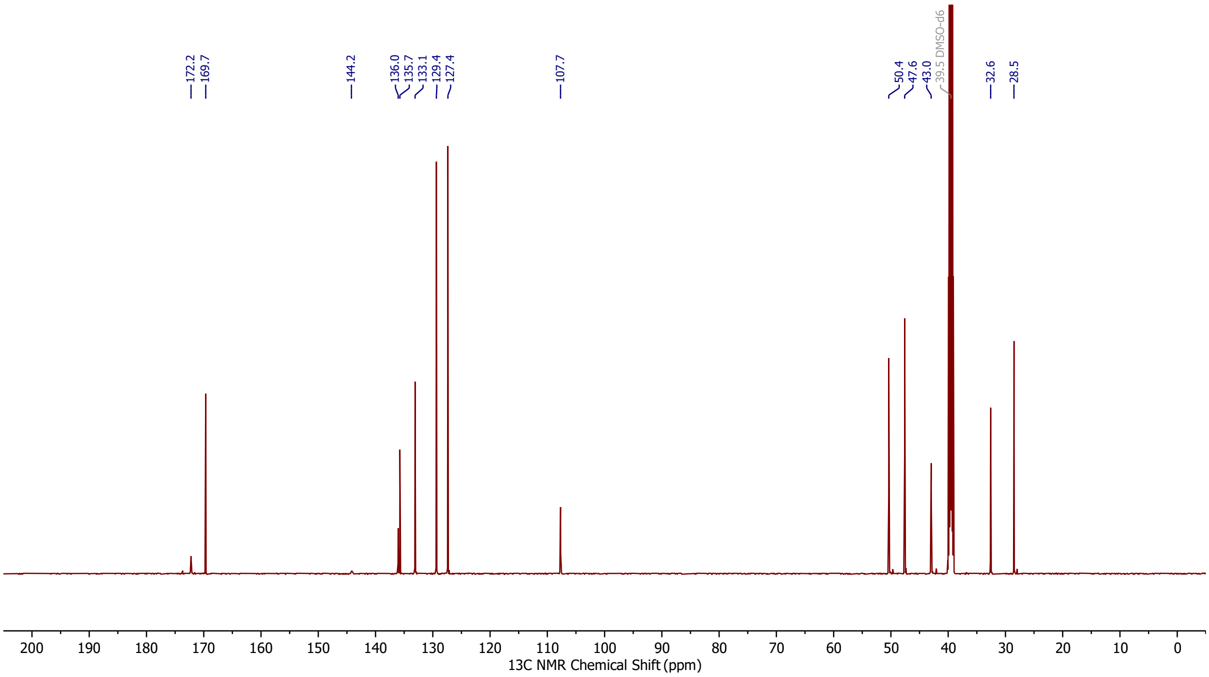

(±)-*tert*-Butyl 3-((4-(2-ethoxy-2-oxoethyl)thiazol-2-yl)carbamoyl)piperidine-1-carboxylate (27a)

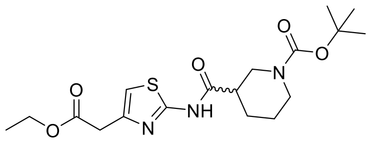

^{1}H NMR (600 MHz, 300 K, $CDCl_3$):

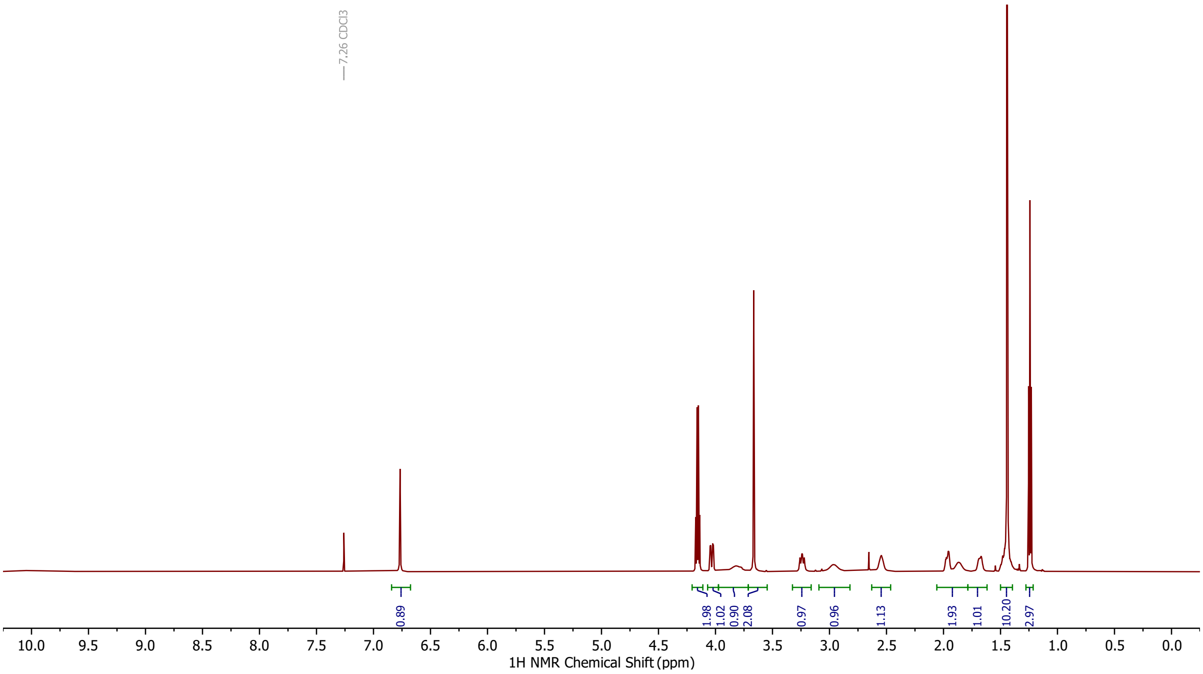

^{13}C NMR (151 MHz, 300 K, $CDCl_3$):

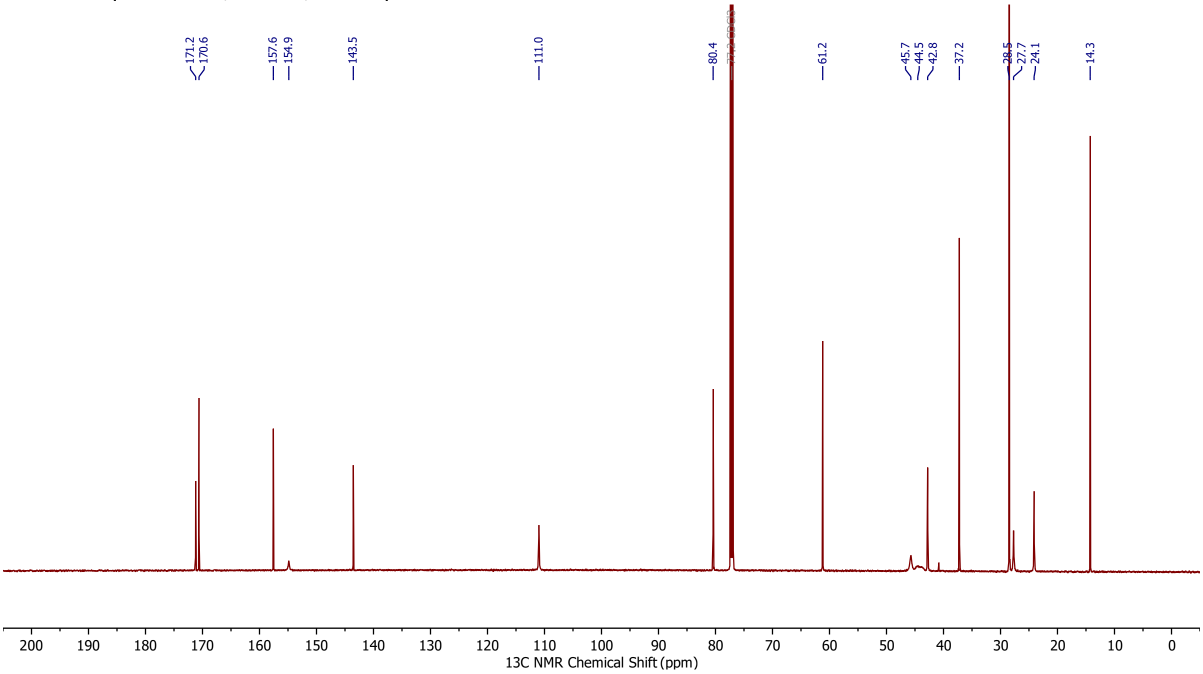

(±)-Ethyl 2-(2-(1-(phenylsulfonyl)piperidine-3-carboxamido)thiazol-4-yl)acetate (27c)

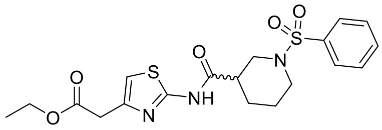

^{1}H NMR (600 MHz, 300 K, $CDCl_3$):

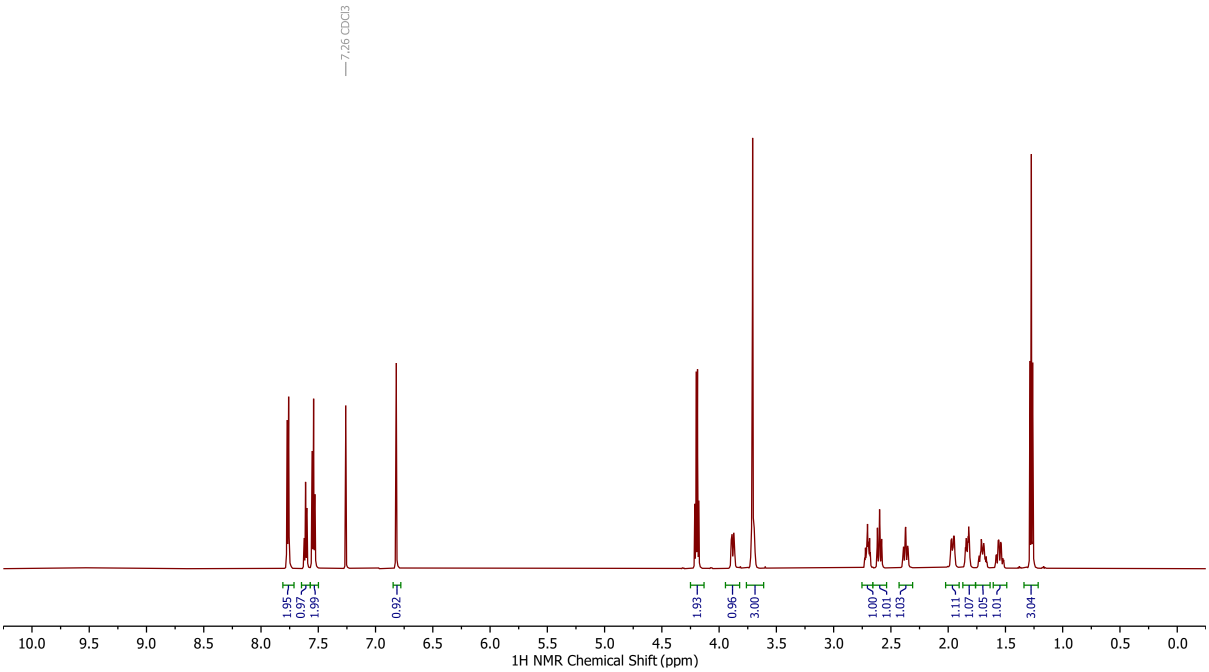

^{13}C NMR (151 MHz, 300 K, $CDCl_3$):

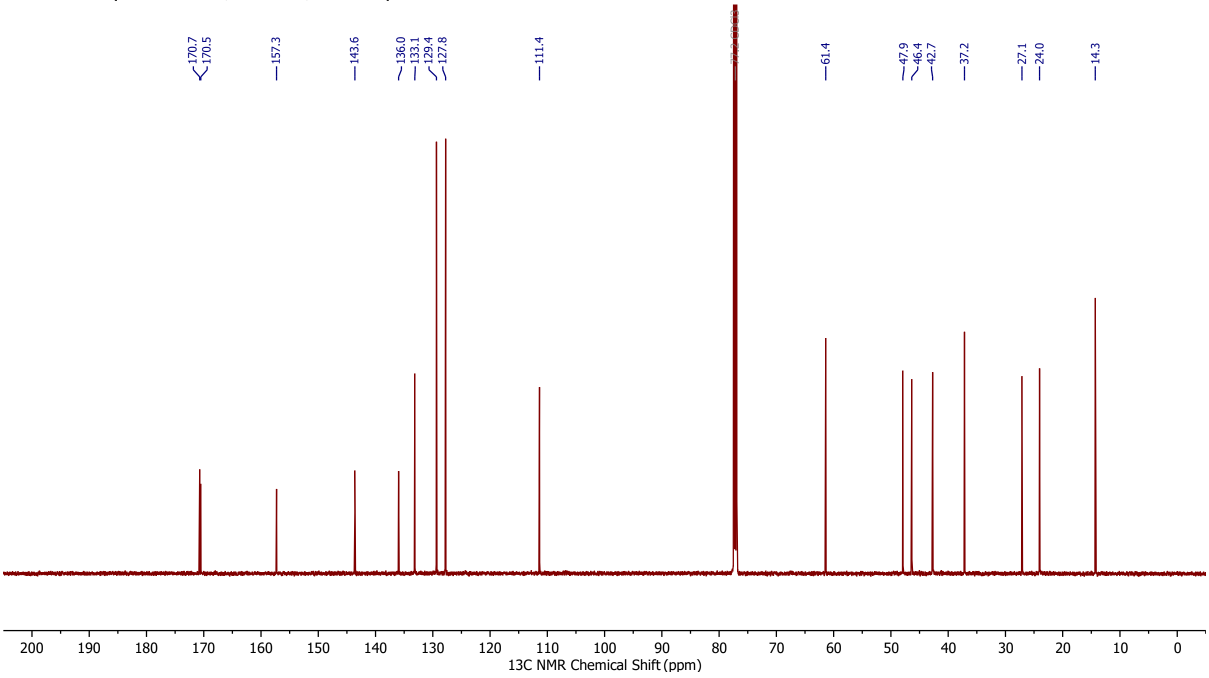

(±)-Ethyl (*Z*)-2-(3-hydroxy-2-((1-(phenylsulfonyl)piperidine-3-carbonyl) imino)-2,3-dihydrothiazol-4-yl)acetate (27d)

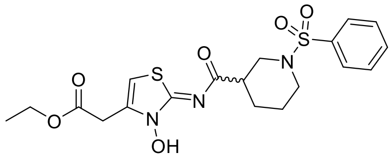

^{1}H NMR (600 MHz, 300 K, $CDCl_3$):

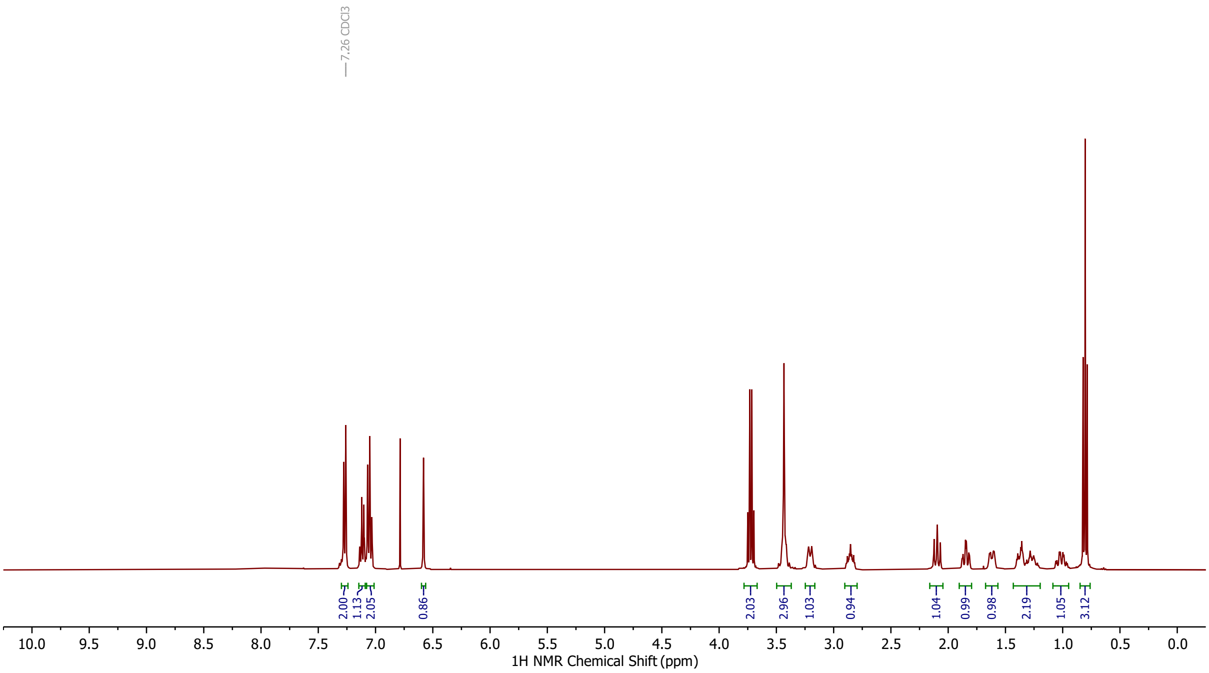

^{13}C NMR (151 MHz, 300 K, $CDCl_3$):

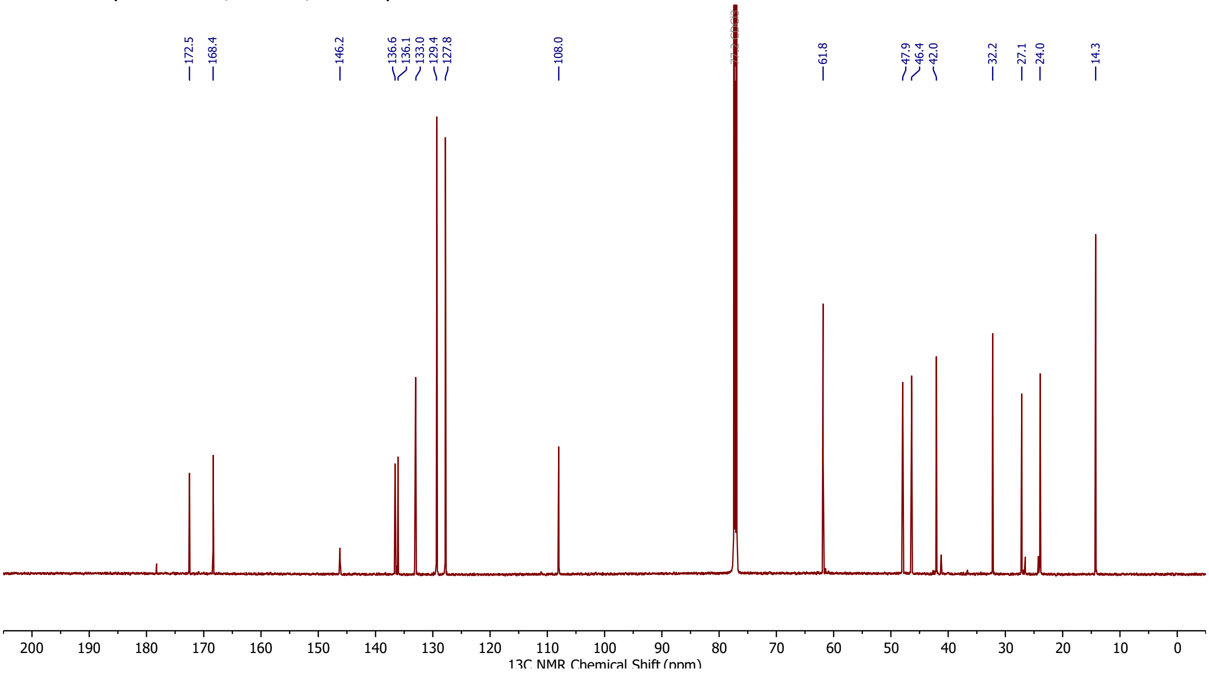

(±)-(*Z*)-2-(3-Hydroxy-2-((1-(phenylsulfonyl)piperidine-3-carbonyl)imino)-2,3-dihydrothiazol-4-yl)acetic acid (27)

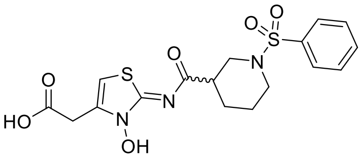

^{1}H NMR (600 MHz, 300 K, DMSO-d_6):

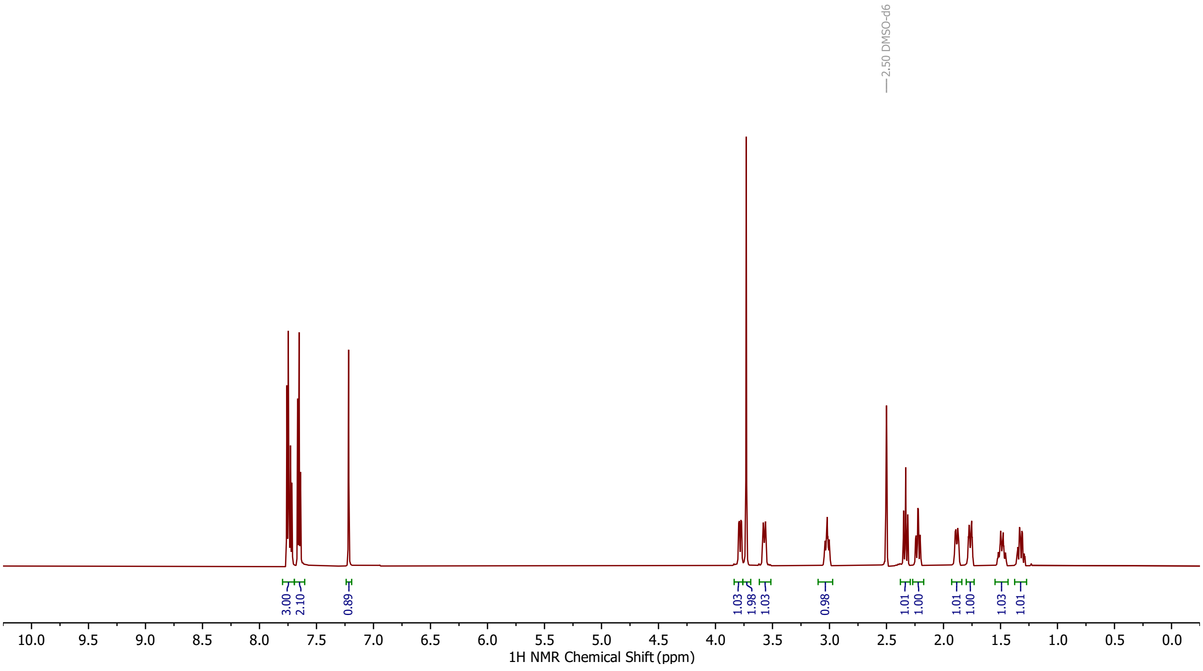

^{13}C NMR (151 MHz, 300 K, DMSO-d_6):

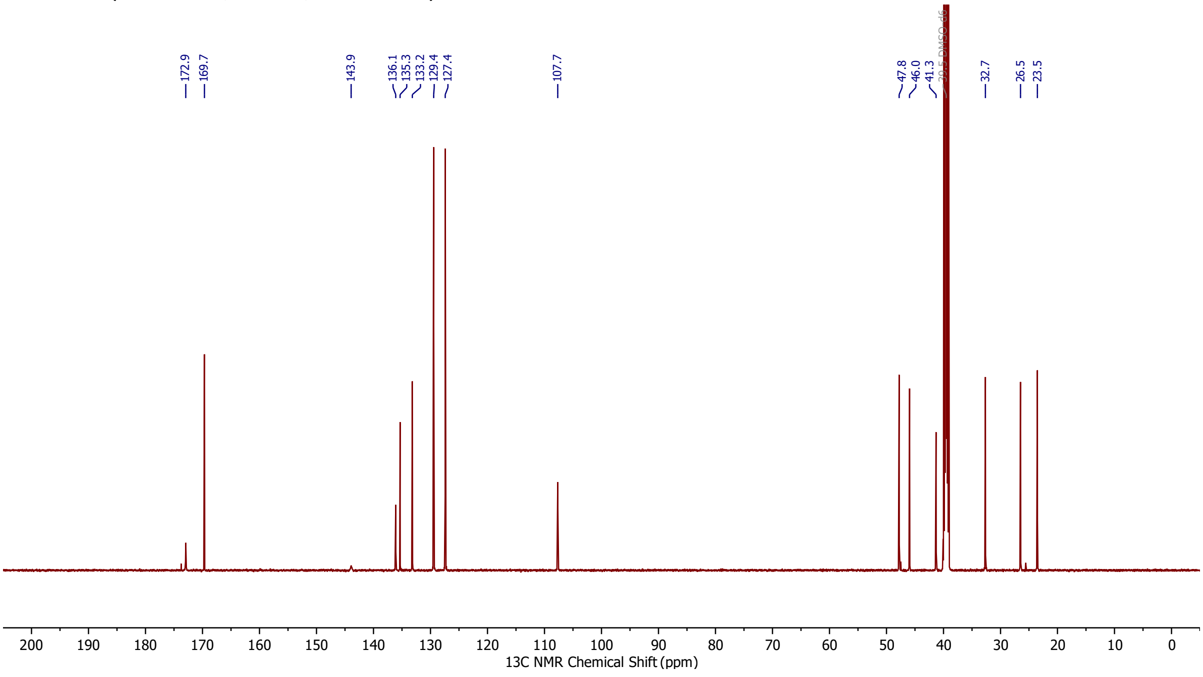

(±)-*tert*-Butyl 2-((4-(2-ethoxy-2-oxoethyl)thiazol-2-yl)carbamoyl)pyrrolidine-1-carboxylate (28a)

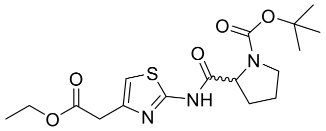

^{1}H NMR (600 MHz, 300 K, DMSO-d_6):

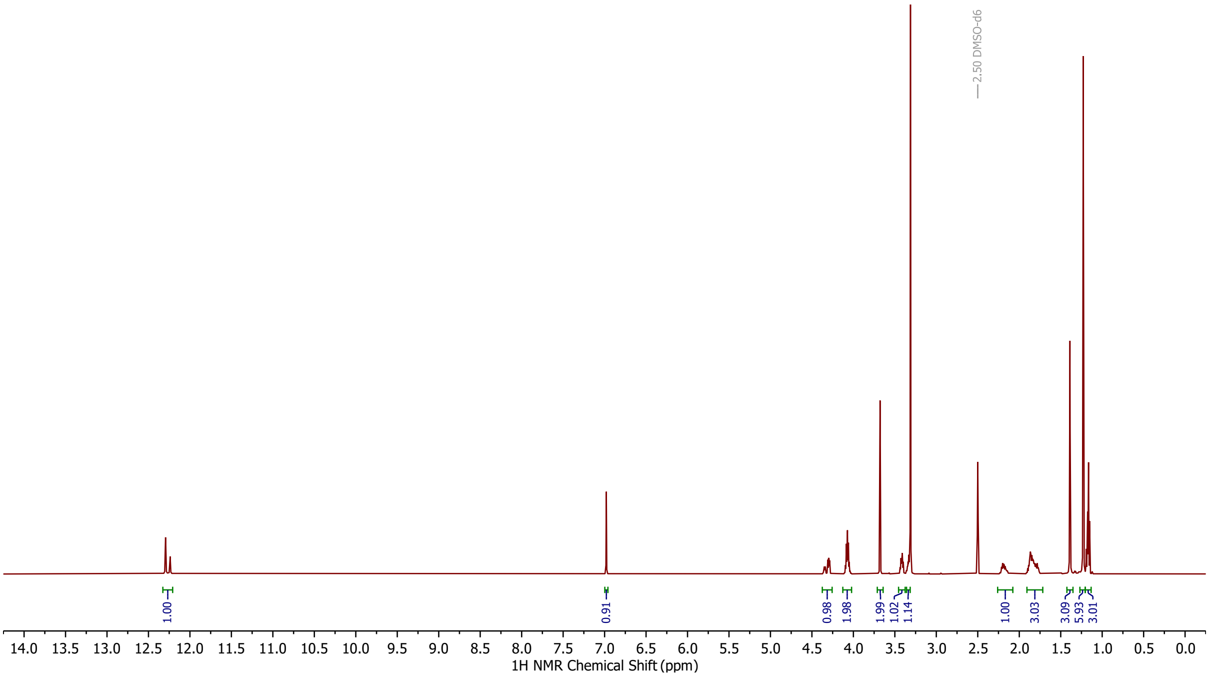

^{13}C NMR (151 MHz, 300 K, DMSO-d_6):

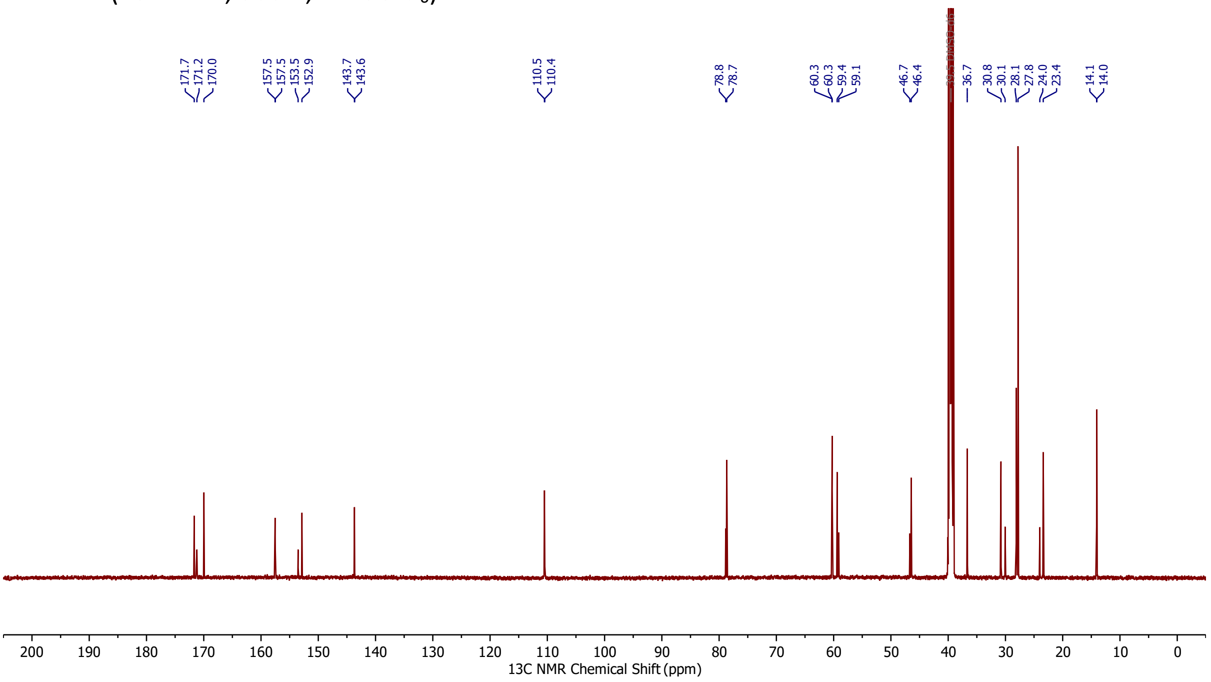

(±)-Ethyl 2-(2-(1-(phenylsulfonyl)pyrrolidine-2-carboxamido)thiazol-4-yl)acetate (28c)

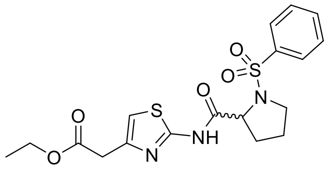

^{1}H NMR (600 MHz, 300 K, DMSO-d_6):

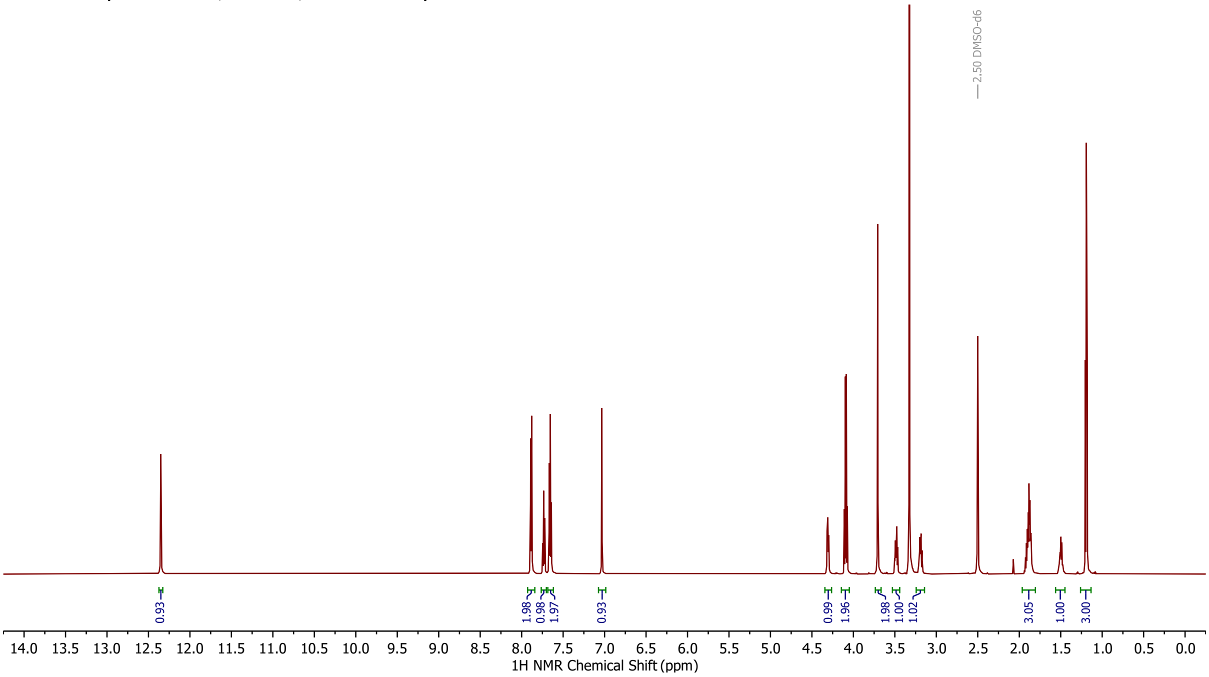

^{13}C NMR (151 MHz, 300 K, DMSO-d_6):

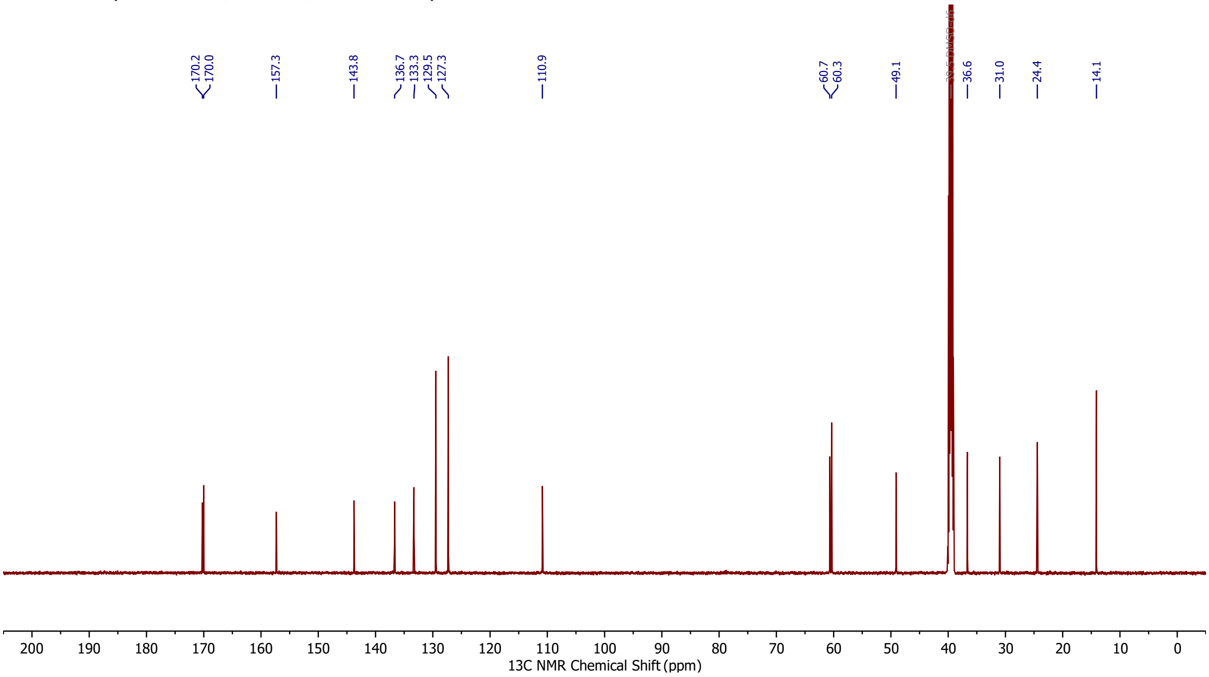

(±)-Ethyl (*Z*)-2-(3-hydroxy-2-(((phenylsulfonyl)prolyl)imino)-2,3-dihydrothiazol-4-yl)acetate (28d)

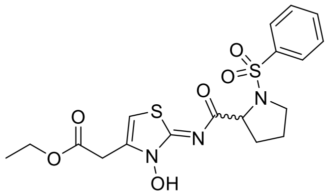

^{1}H NMR (600 MHz, 300 K, $CDCl_3$):

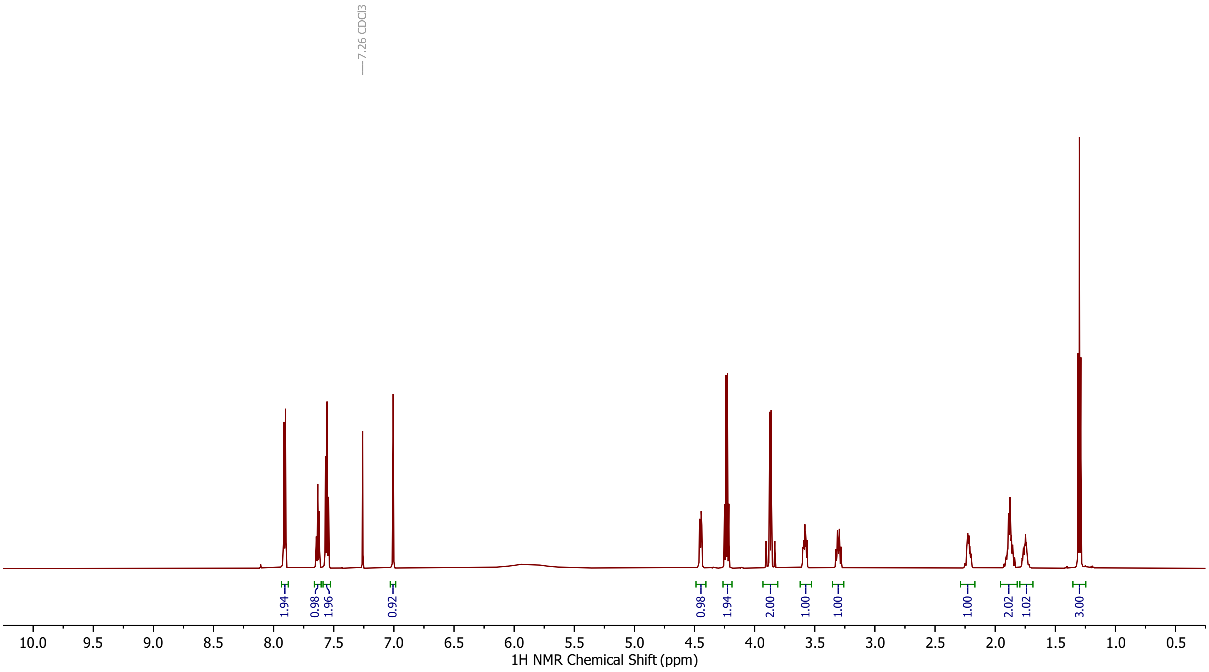

^{13}C NMR (151 MHz, 300 K, $CDCl_3$):

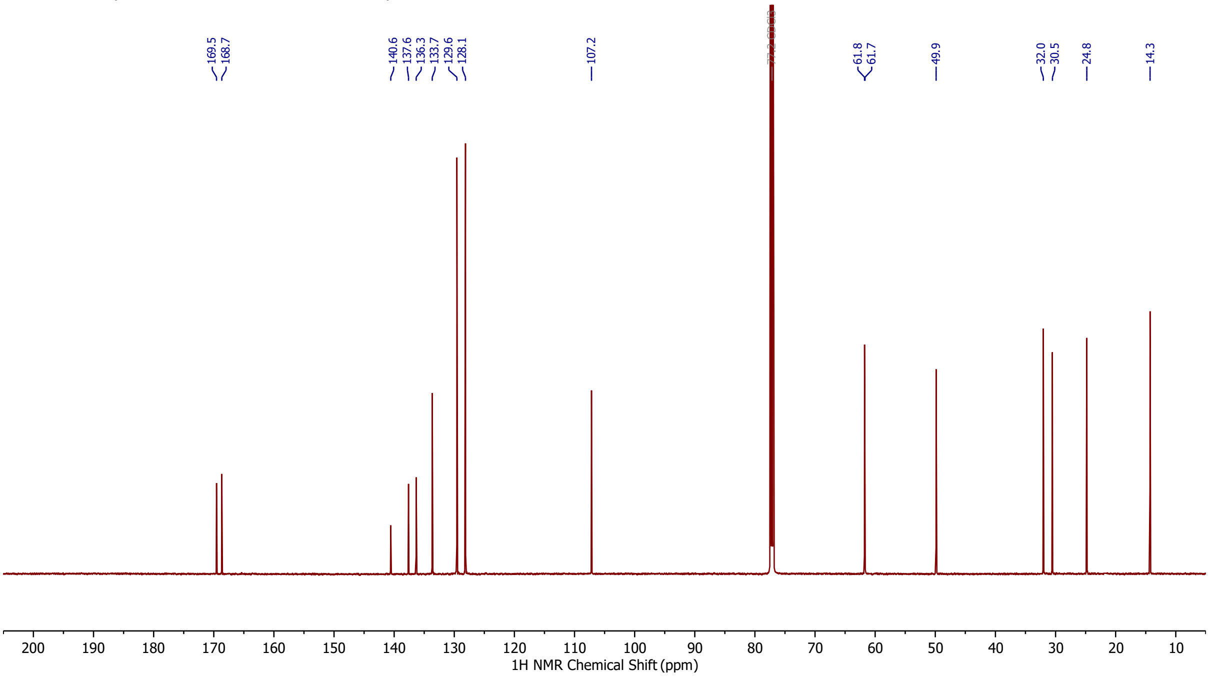

(±)-(*Z*)-2-(3-Hydroxy-2-(((phenylsulfonyl)prolyl)imino)-2,3-dihydrothiazol-4-yl)acetic acid (28)

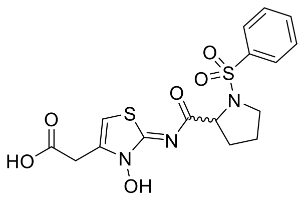

^{1}H NMR (600 MHz, 300 K, DMSO-d_6):

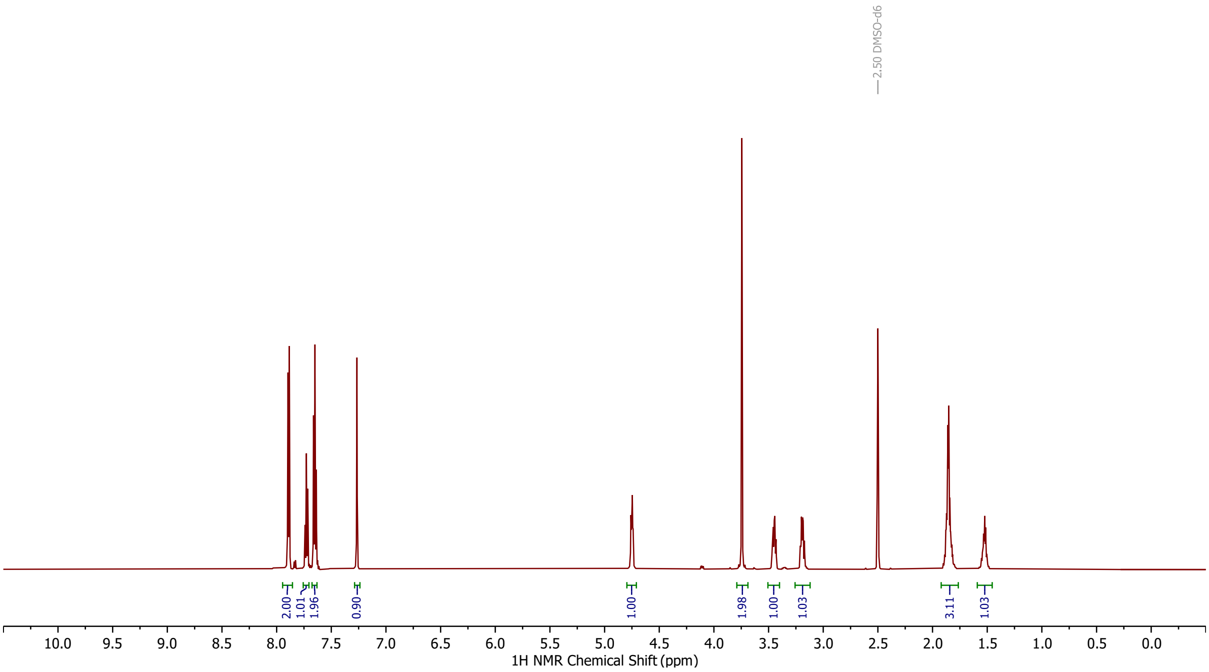

^{13}C NMR (151 MHz, 300 K, DMSO-d_6):

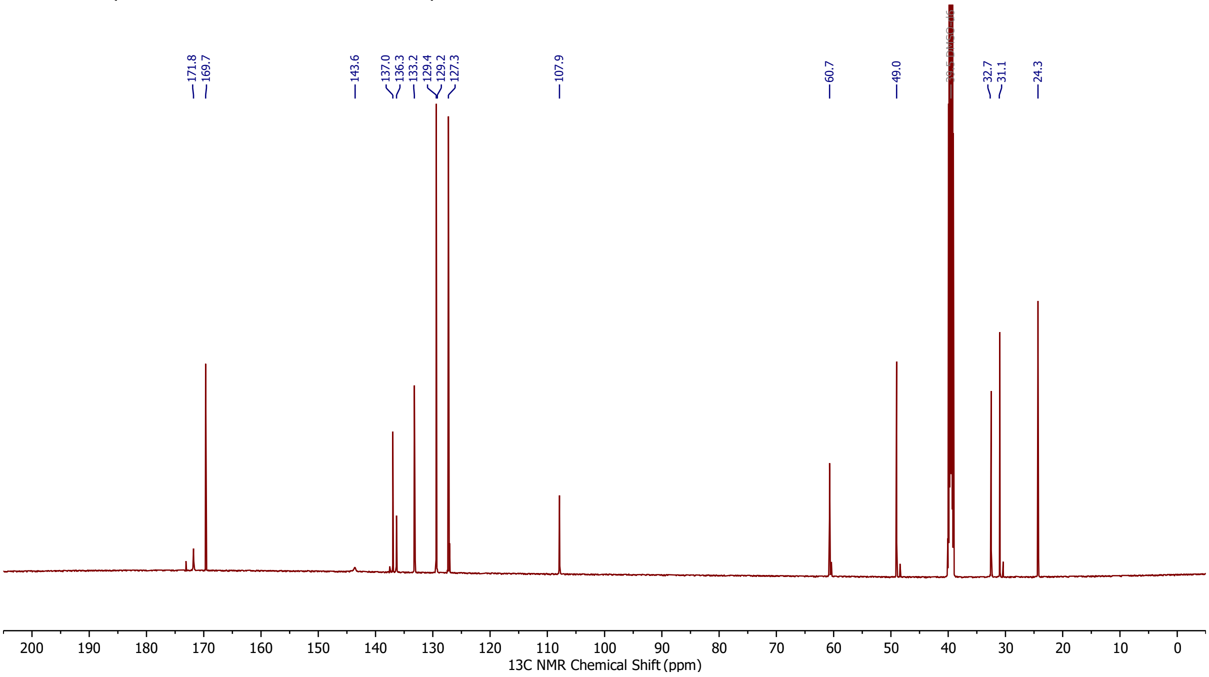

(±)-*tert*-Butyl 3-((4-(2-ethoxy-2-oxoethyl)thiazol-2-yl)carbamoyl)-3-methylpyrrolidine-1-carboxylate (29a)

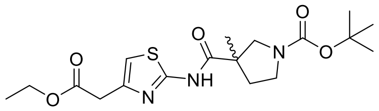

^{1}H NMR (600 MHz, 300 K, DMSO-d_6):

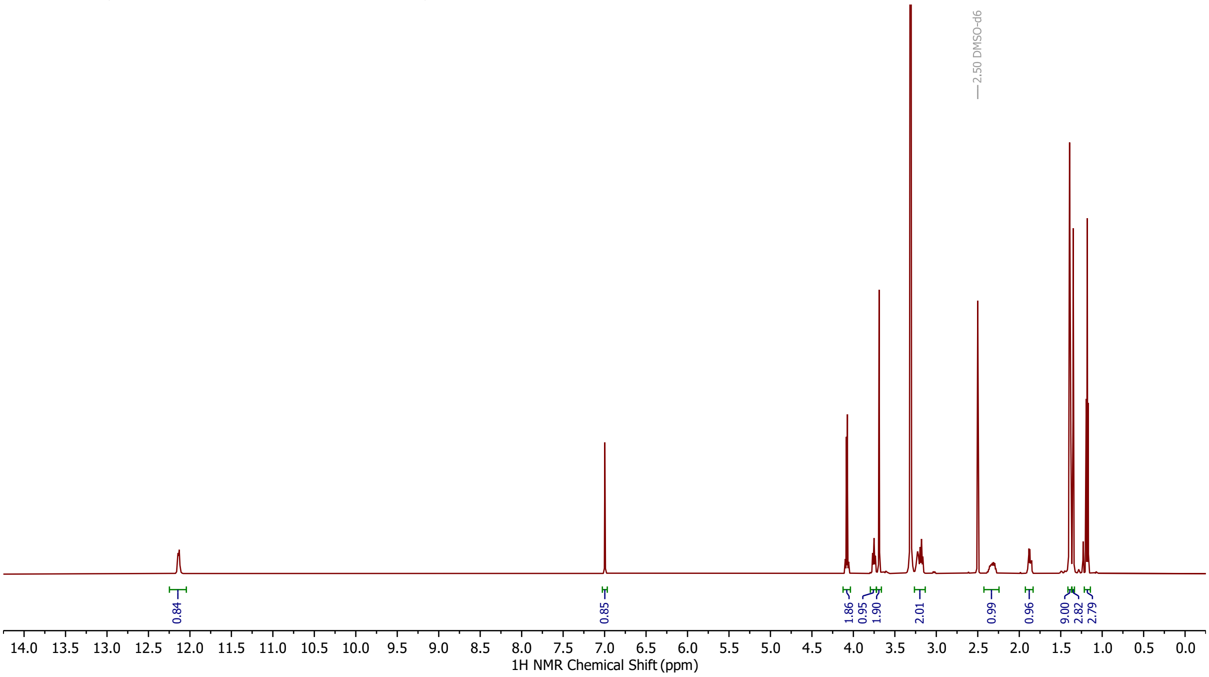

^{13}C NMR (151 MHz, 300 K, DMSO-d_6):

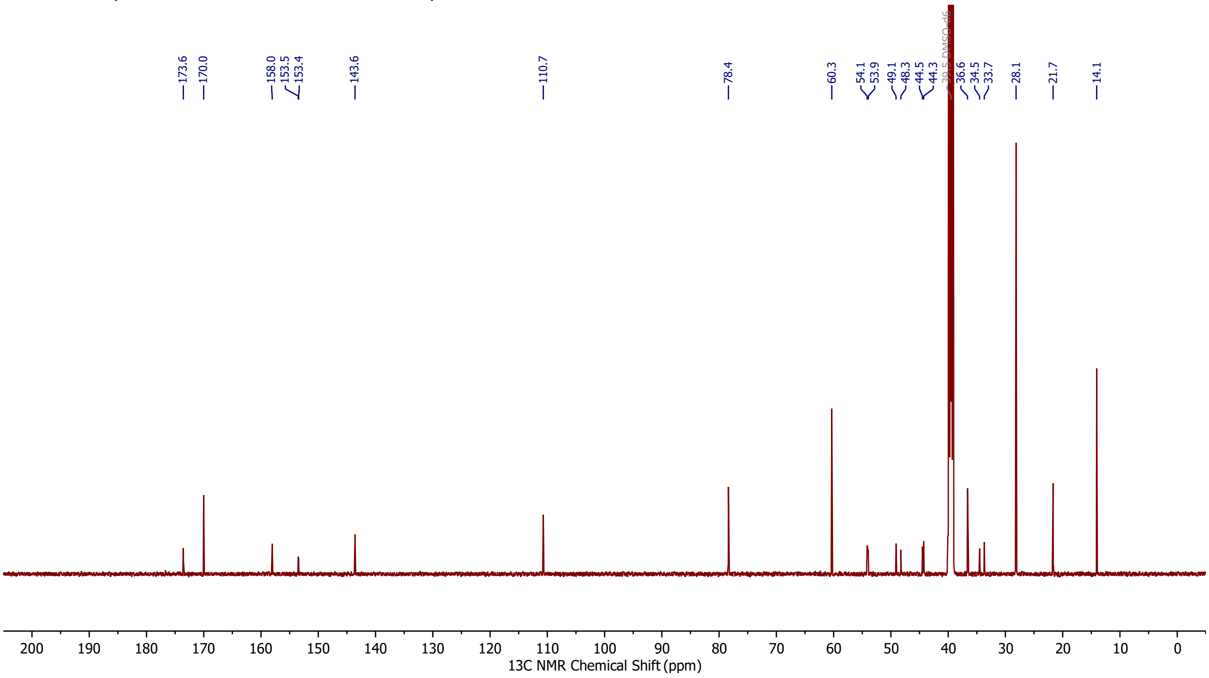

(±)-Ethyl 2-(2-(3-methyl-1-(phenylsulfonyl)pyrrolidine-3-carboxamido) thiazol-4-yl)acetate (29c)

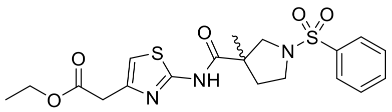

^{1}H NMR (600 MHz, 300 K, $CDCl_3$):

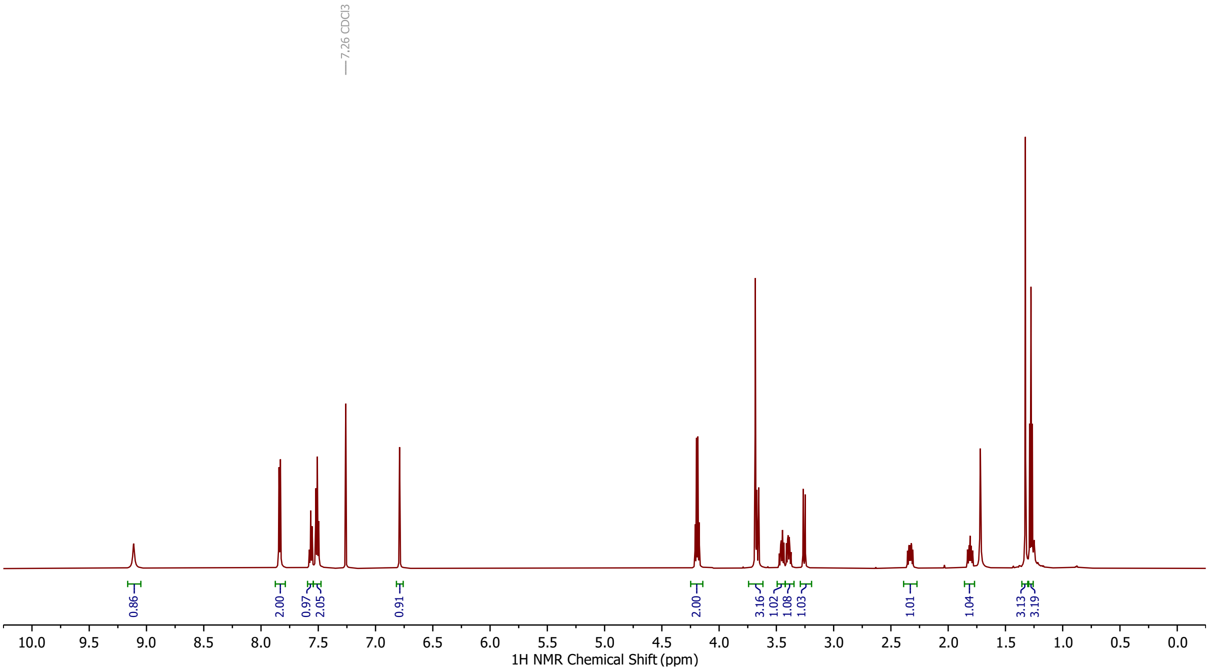

^{13}C NMR (151 MHz, 300 K, $CDCl_3$):

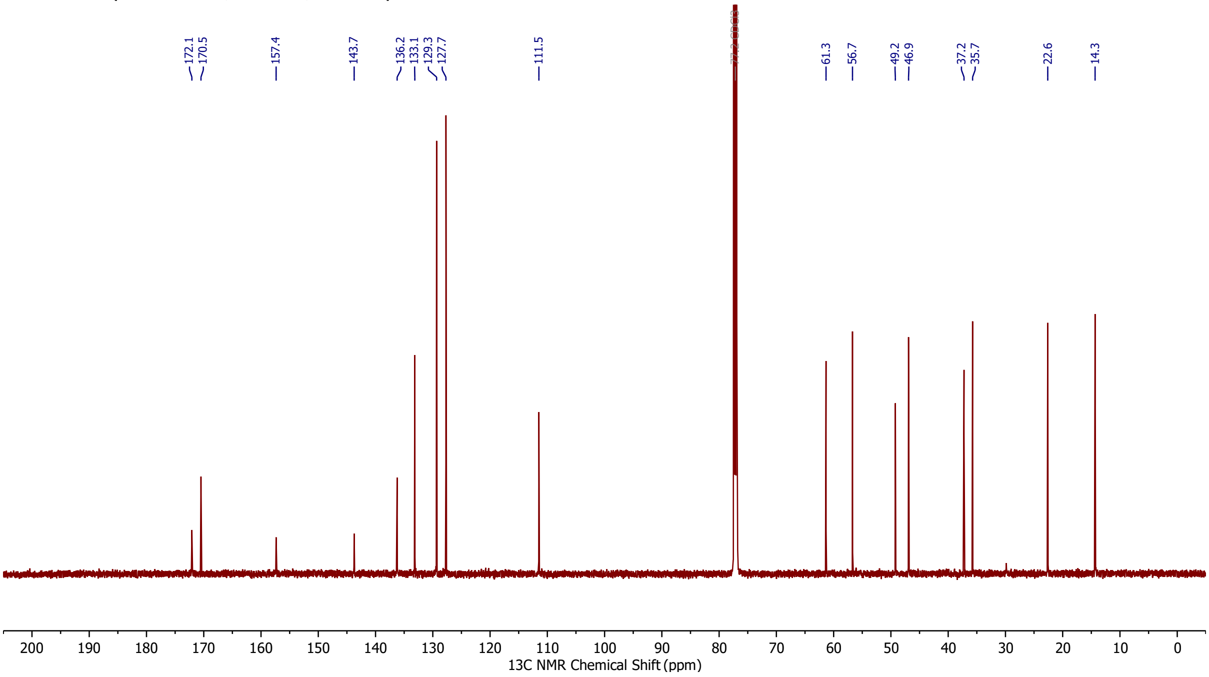

(±)-Ethyl (*Z*)-2-(3-hydroxy-2-((3-methyl-1-(phenylsulfonyl)pyrrolidine-3-carbonyl)imino)-2,3-dihydrothiazol-4-yl)acetate (29d)

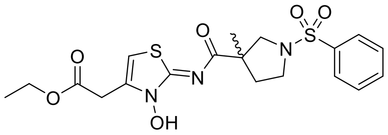

^{1}H NMR (600 MHz, 300 K, $CDCl_3$):

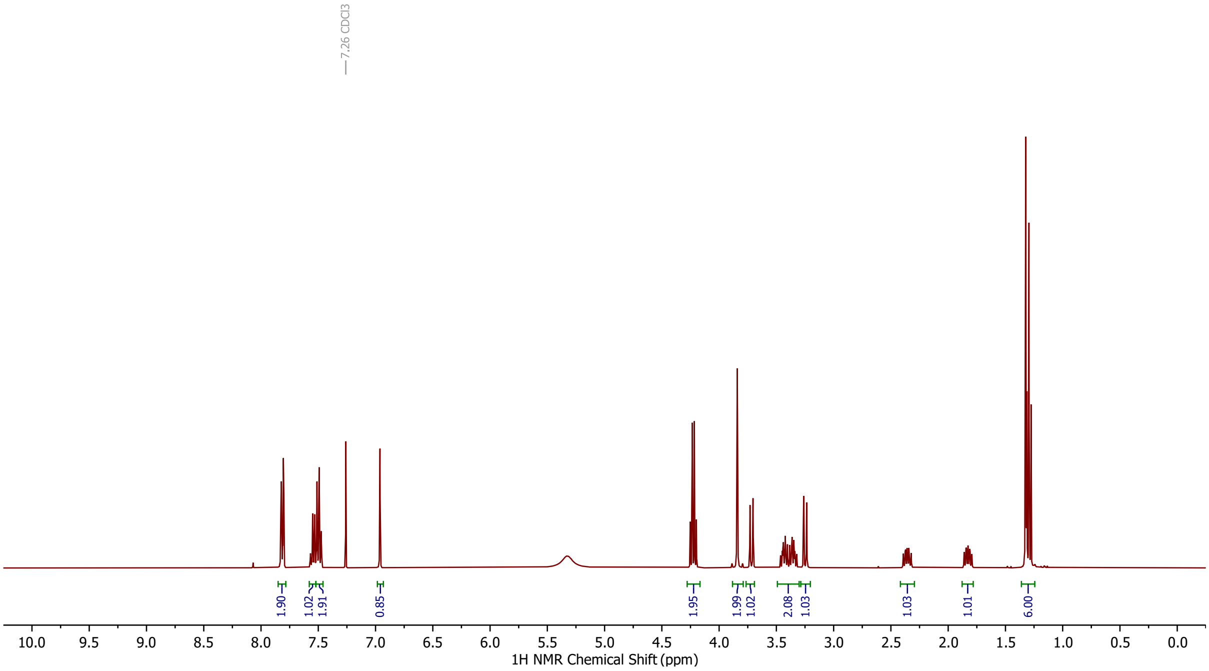

^{13}C NMR (151 MHz, 300 K, $CDCl_3$):

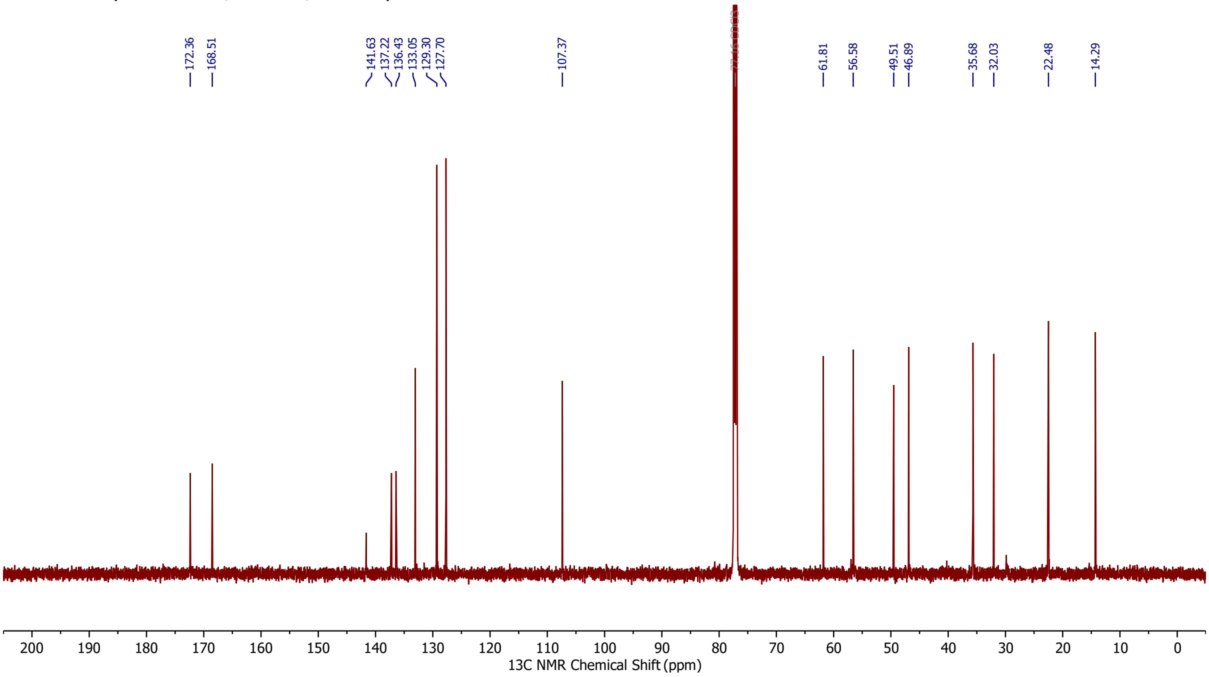

(±)-(*Z*)-2-(3-Hydroxy-2-((3-methyl-1-(phenylsulfonyl)pyrrolidine-3-carbonyl) imino)-2,3-dihydrothiazol-4-yl)acetic acid (29)

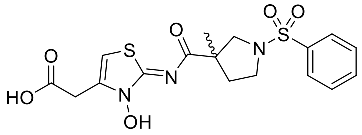

^{1}H NMR (600 MHz, 300 K, DMSO-d_6):

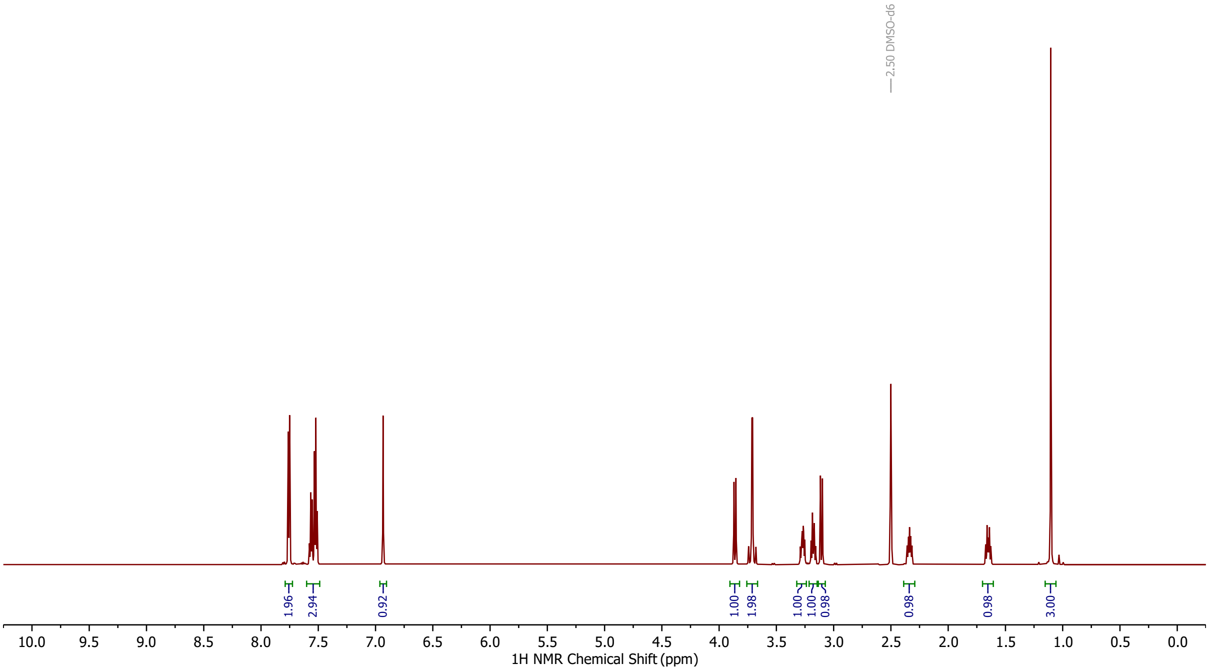

^{13}C NMR (151 MHz, 300 K, DMSO-d_6):

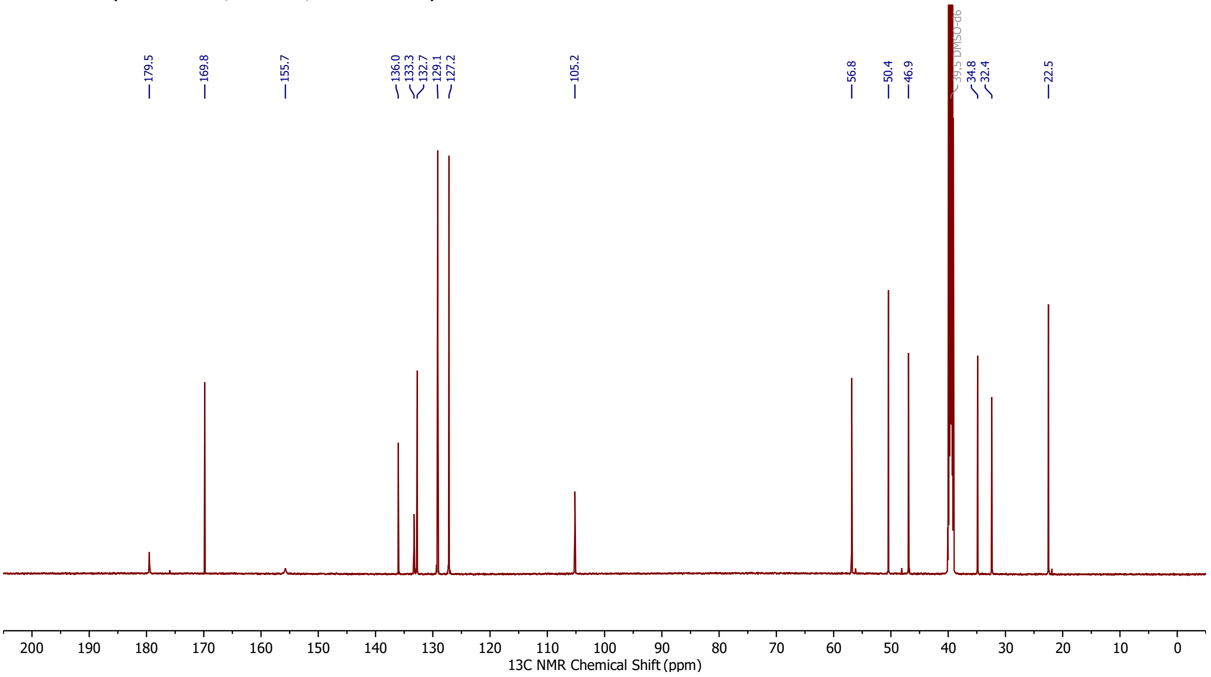

(±)-*trans*-Methyl 4-methyl-1-(phenylsulfonyl)pyrrolidine-3-carboxylate (30d)

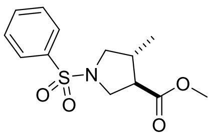

^{1}H NMR (600 MHz, 300 K, $CDCl_3$):

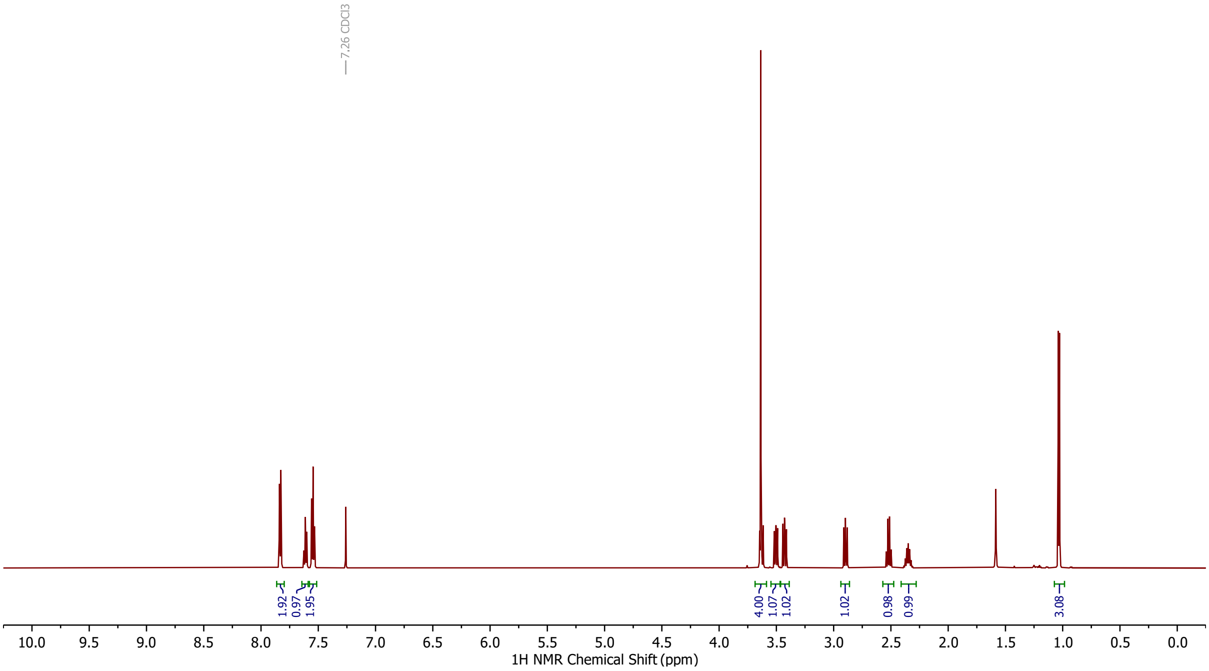

^{13}C NMR (151 MHz, 300 K, $CDCl_3$):

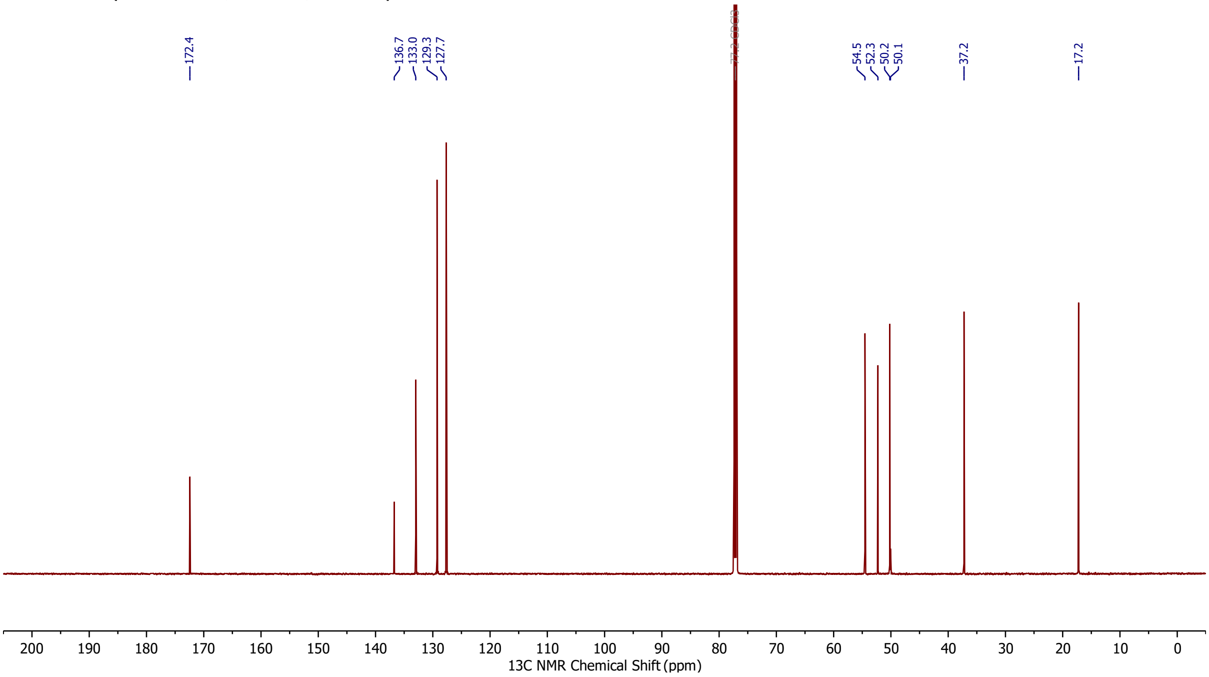

(±)-*trans*-4-Methyl-1-(phenylsulfonyl)pyrrolidine-3-carboxylic acid (30e)

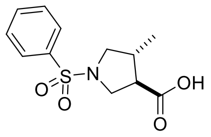

^1H NMR (600 MHz, 300 K, $CDCl_3$):

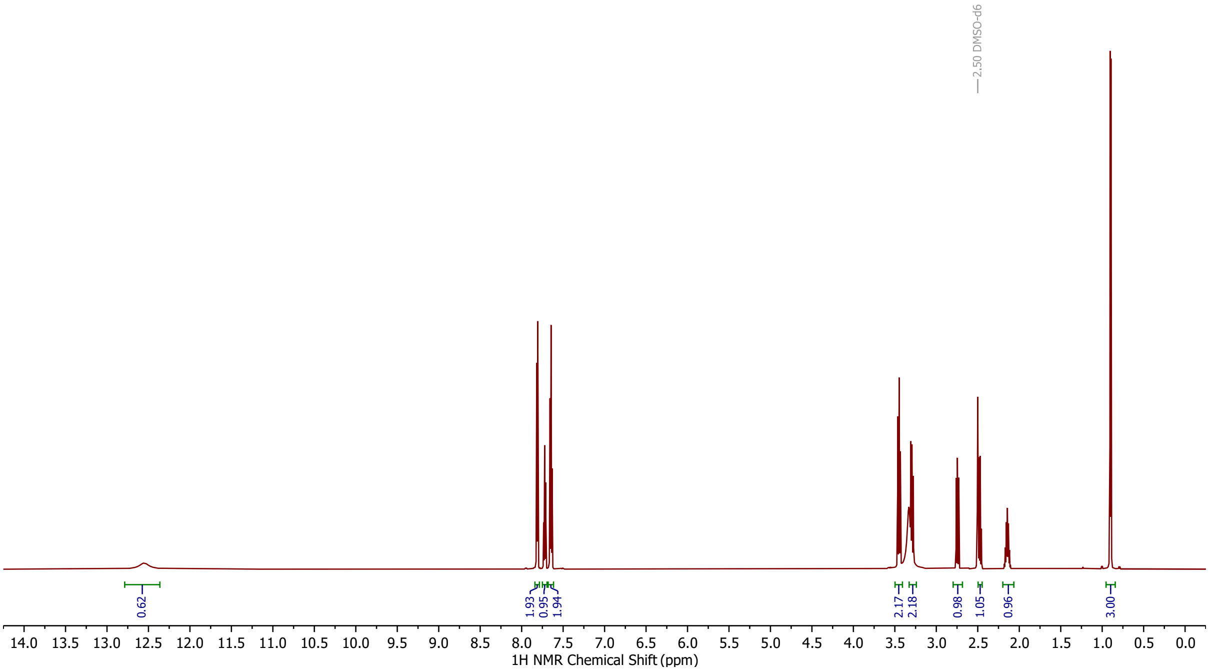

^{13}C NMR (151 MHz, 300 K, $CDCl_3$):

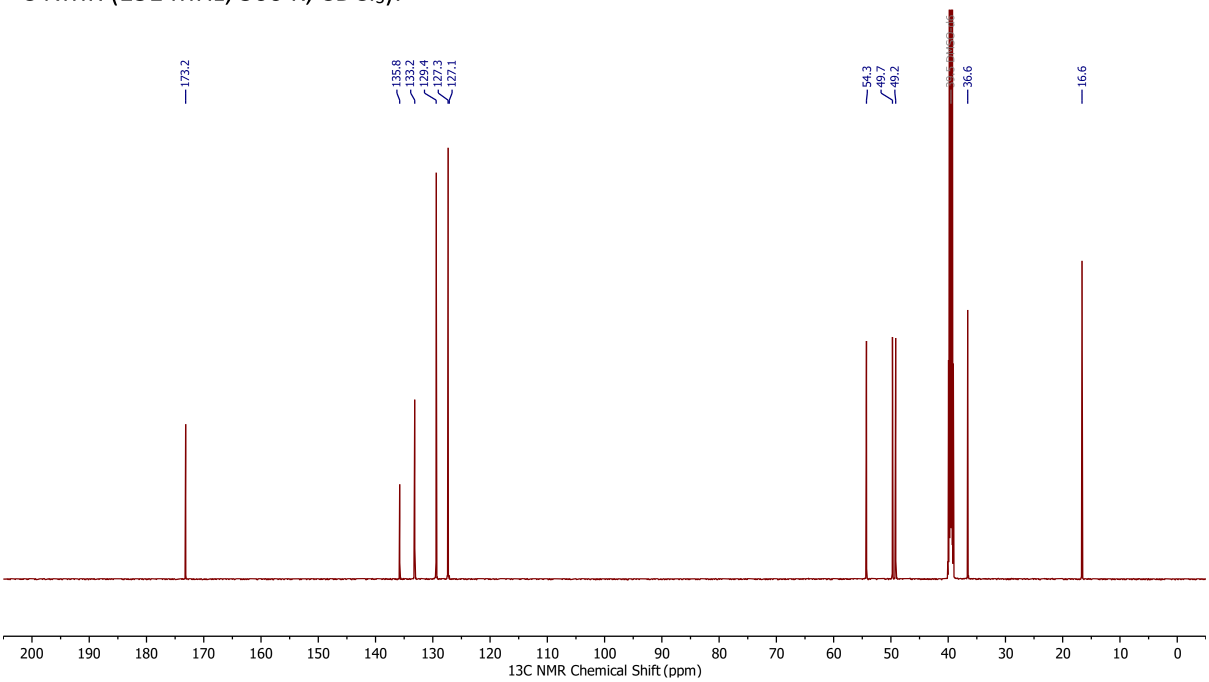

(±)-*trans*-Ethyl 2-(2-(4-methyl-1-(phenylsulfonyl)pyrrolidine-3-carboxamido)thiazol-4-yl)acetate (30f)

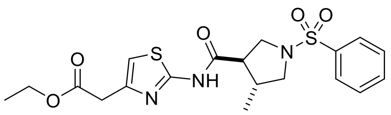

^{1}H NMR (600 MHz, 300 K, DMSO-d_6):

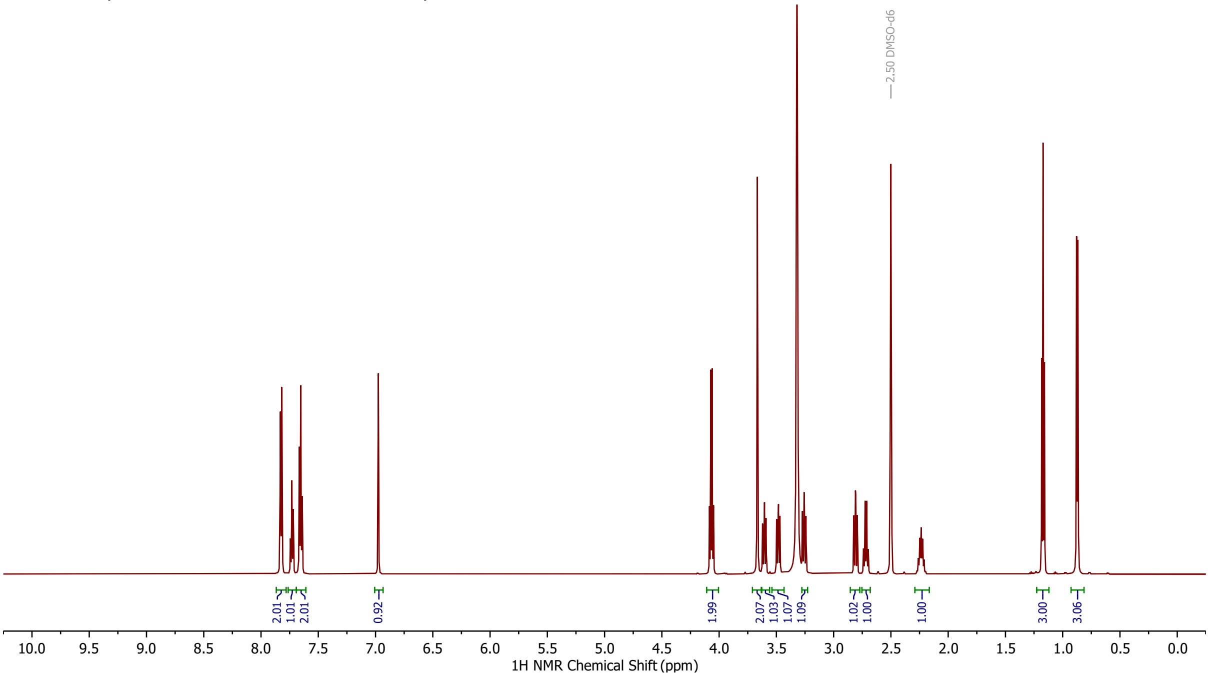

^{13}C NMR (151 MHz, 300 K, DMSO-d_6):

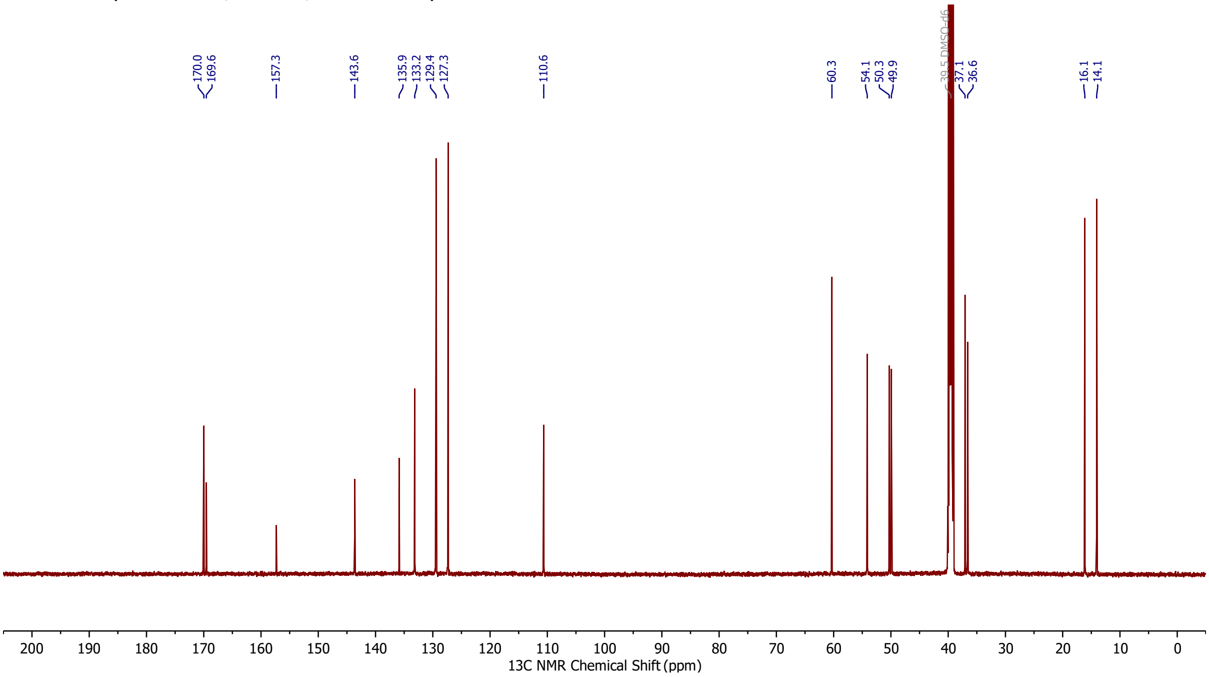

(±)-*trans*-Ethyl 2-((*Z*)-3-hydroxy-2-((4-methyl-1-(phenylsulfonyl)pyrrolidine-3-carbonyl)imino)-2,3-dihydrothiazol-4-yl)acetate (30g)

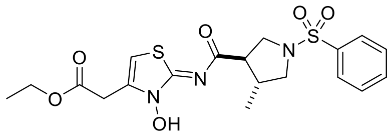

^{1}H NMR (600 MHz, 300 K, DMSO-d_6):

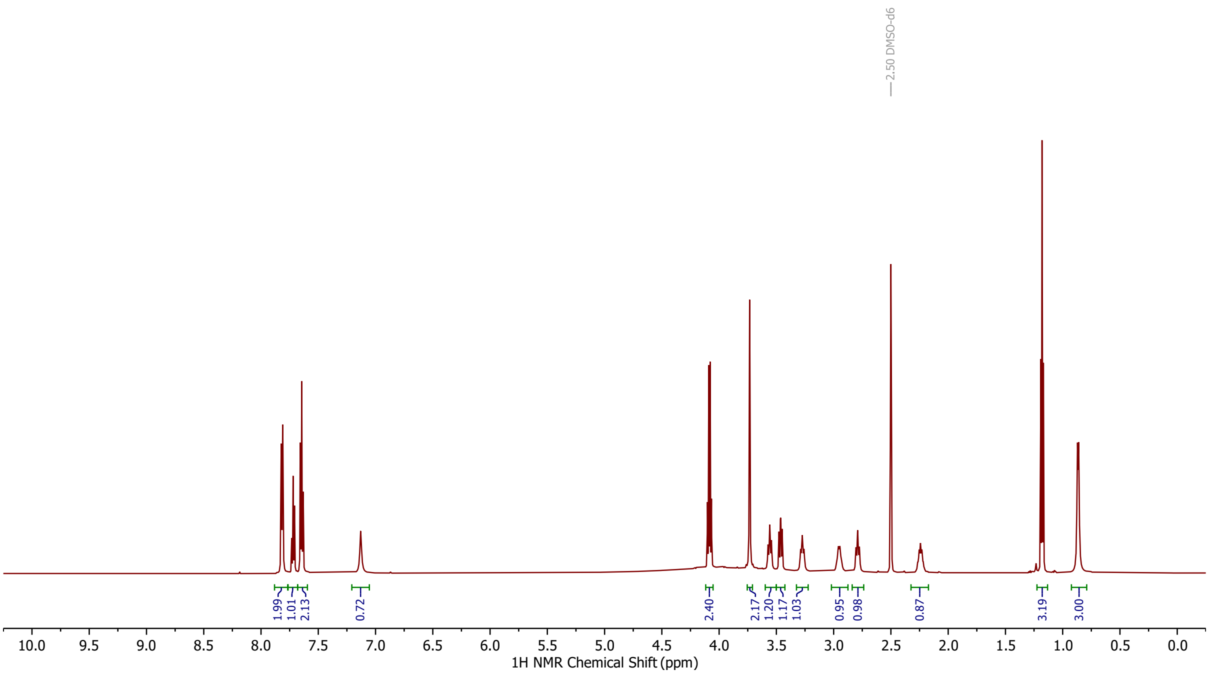

^{13}C NMR (151 MHz, 300 K, DMSO-d_6):

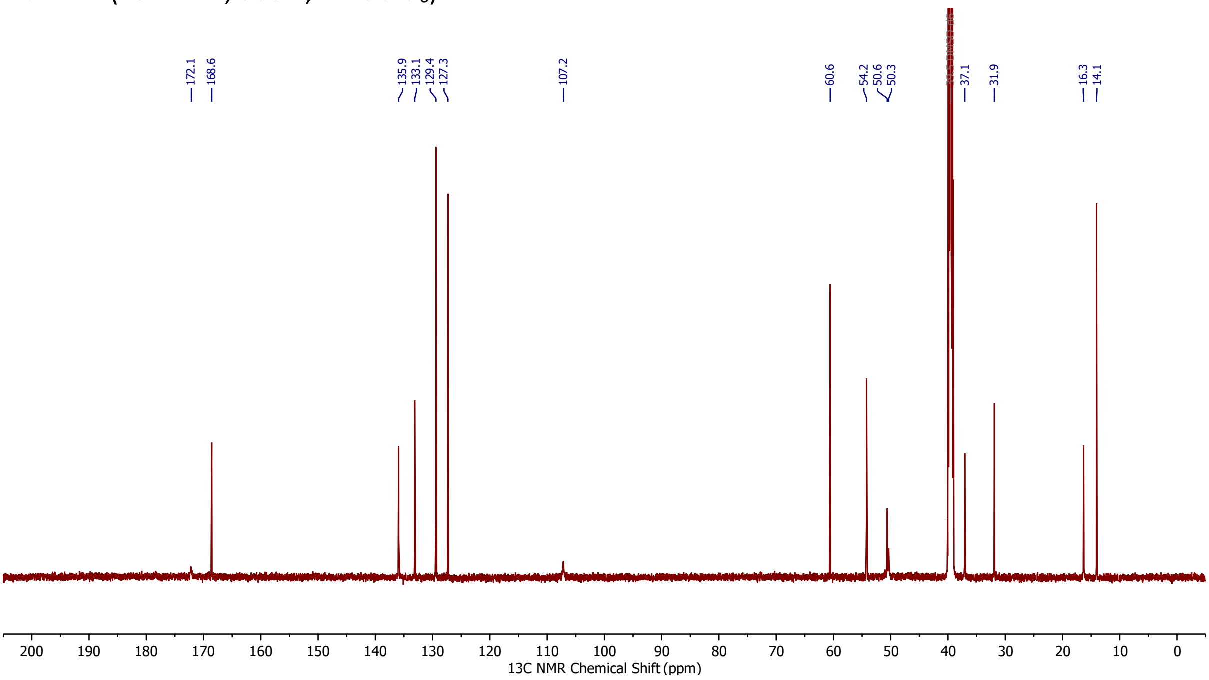

(±)-*trans*-2-((*Z*)-3-Hydroxy-2-((4-methyl-1-(phenylsulfonyl)pyrrolidine-3-carbonyl)imino)-2,3-dihydrothiazol-4-yl)acetic acid (30)

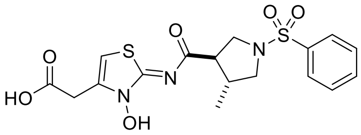

^{1}H NMR (600 MHz, 300 K, DMSO-d_6):

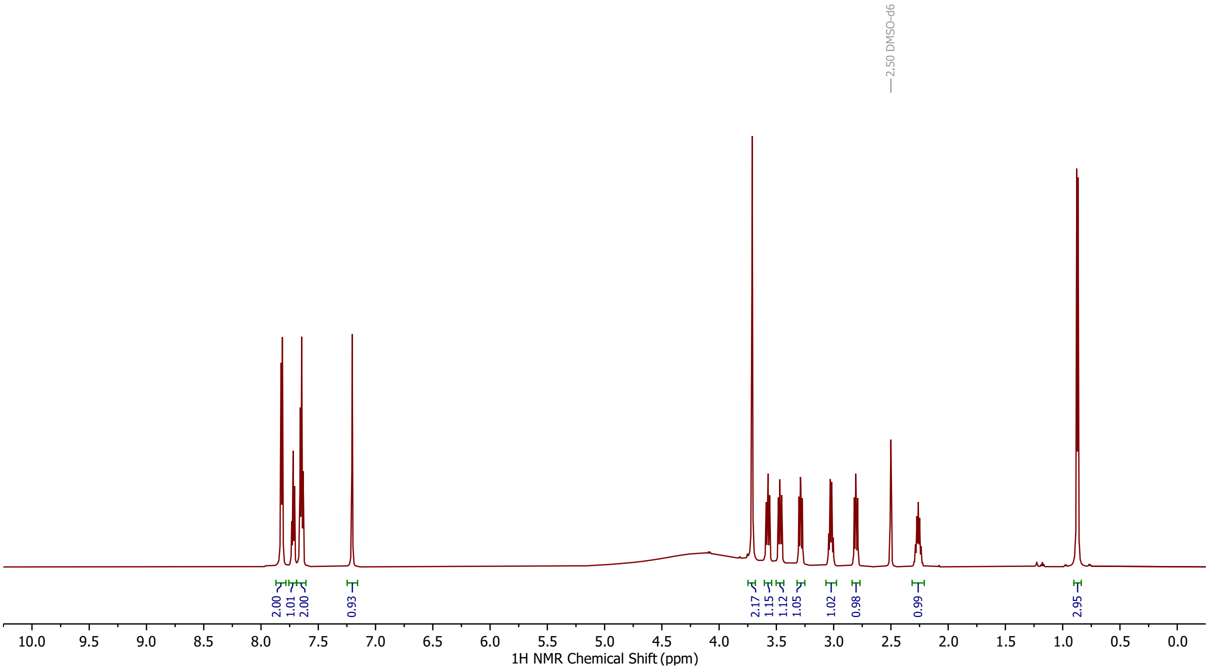

^{13}C NMR (151 MHz, 300 K, DMSO-d_6):

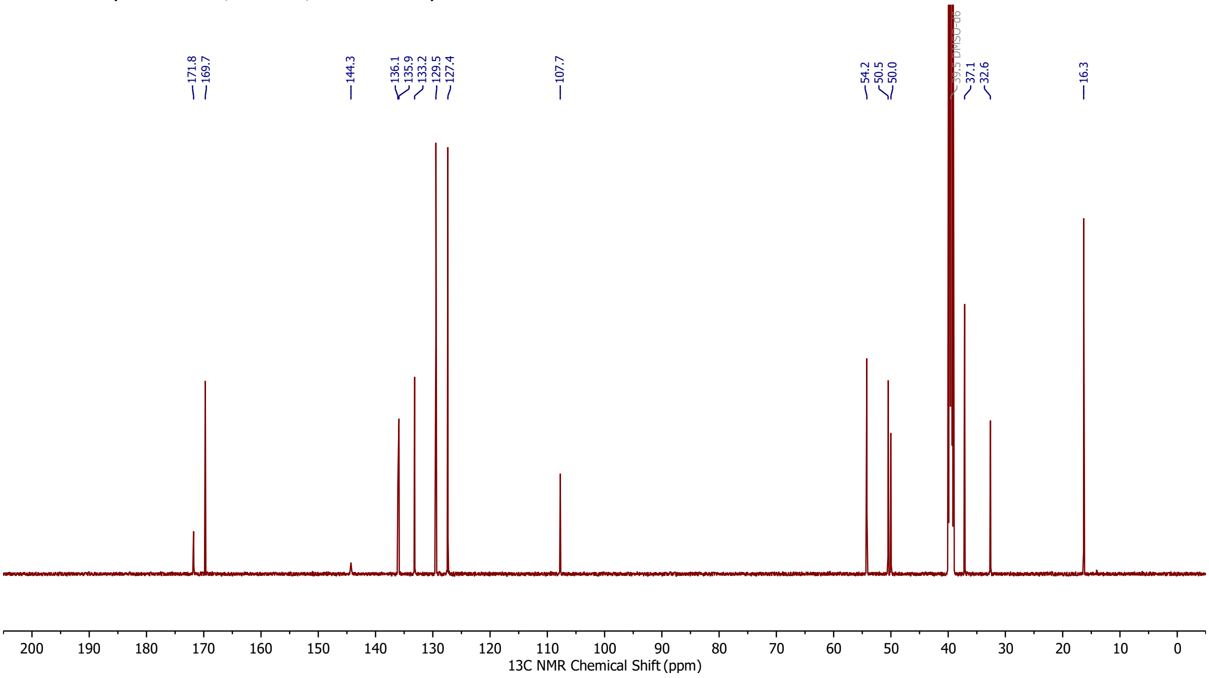

(±)-*tert*-Butyl 3-((4-(2-ethoxy-2-oxoethyl)thiazol-2-yl)carbamoyl)pyrrolidine-1-carboxylate (31)

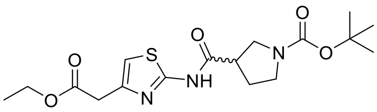

^{1}H NMR (600 MHz, 300 K, DMSO-d_6):

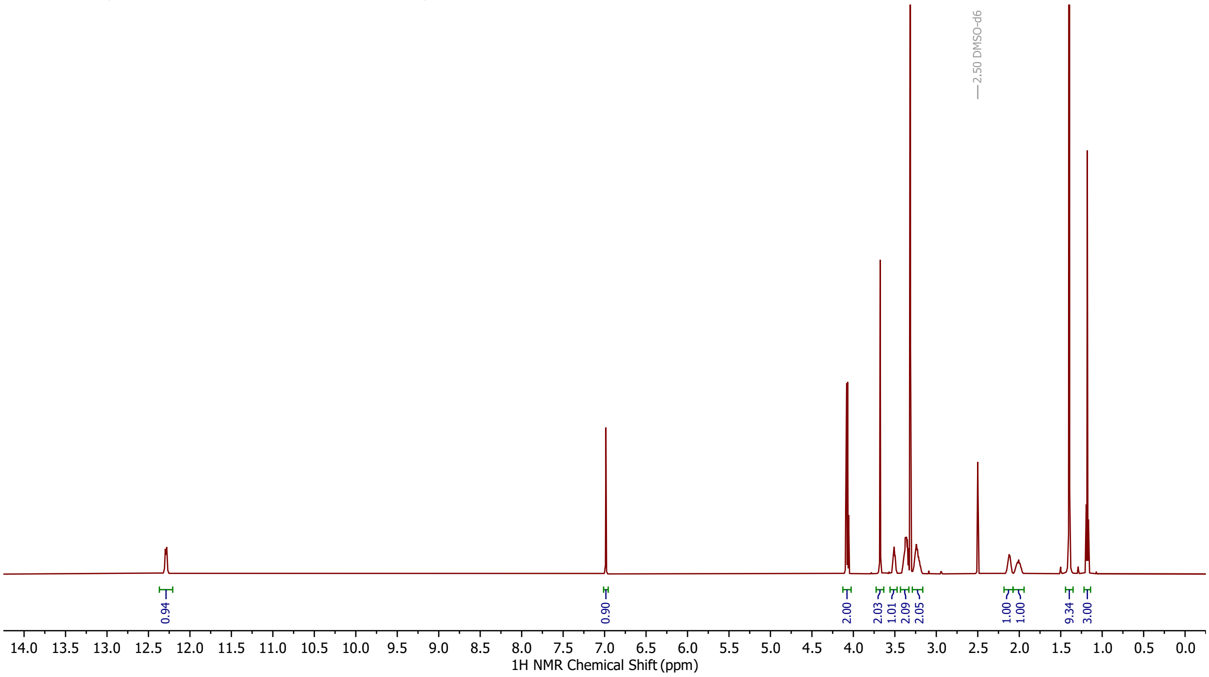

^{13}C NMR (151 MHz, 300 K, DMSO-d_6):

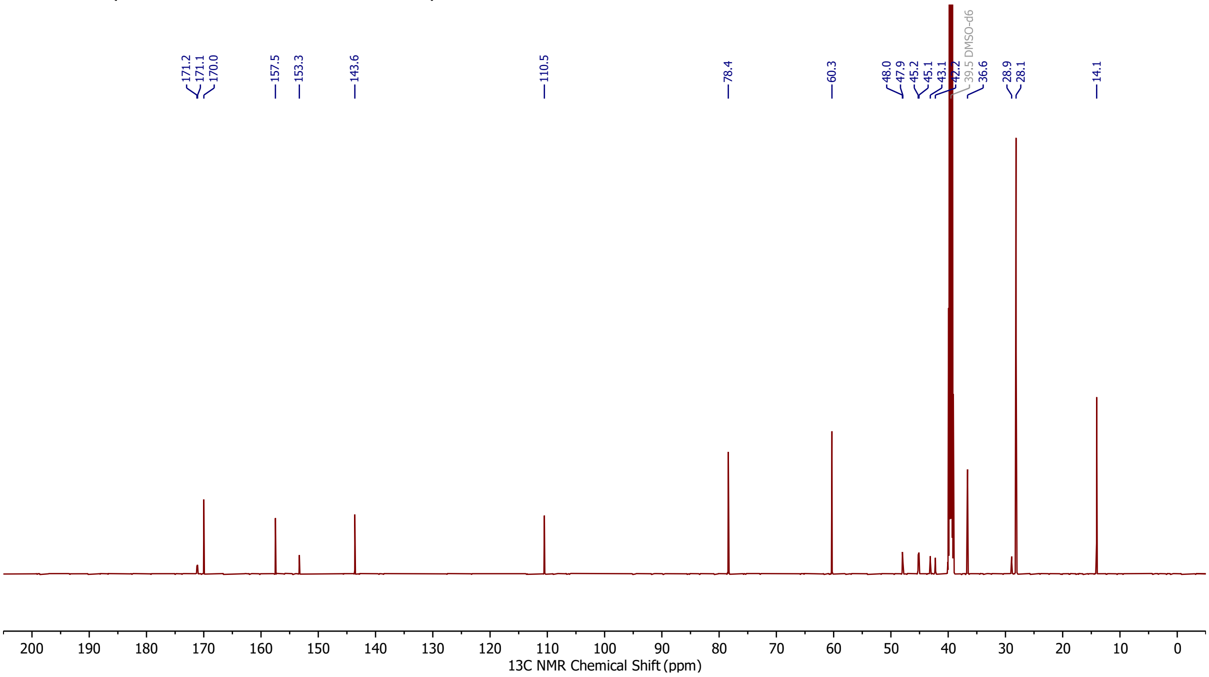

(±)-Ethyl 2-(2-(1-((2-chlorophenyl)sulfonyl)pyrrolidine-3-carboxamido)thiazol-4-yl)acetate (33a)

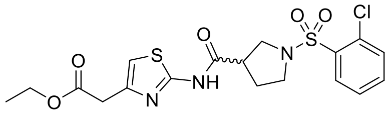

^{1}H NMR (600 MHz, 300 K, $CDCl_3$):

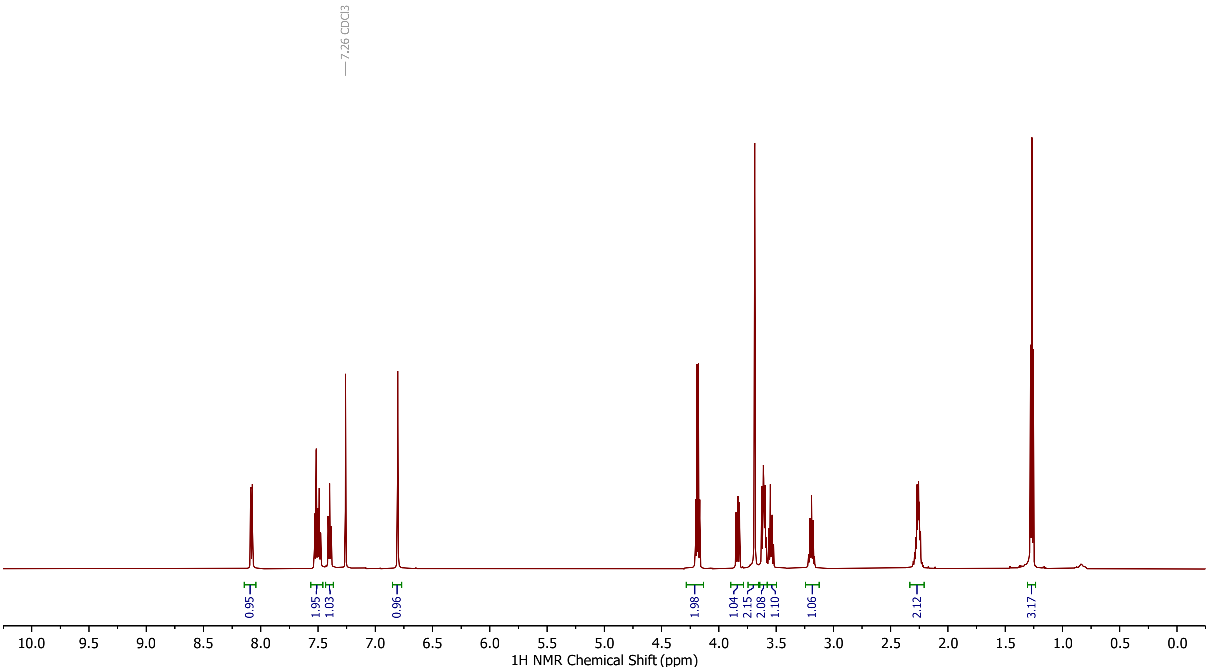

^{13}C NMR (151 MHz, 300 K, $CDCl_3$):

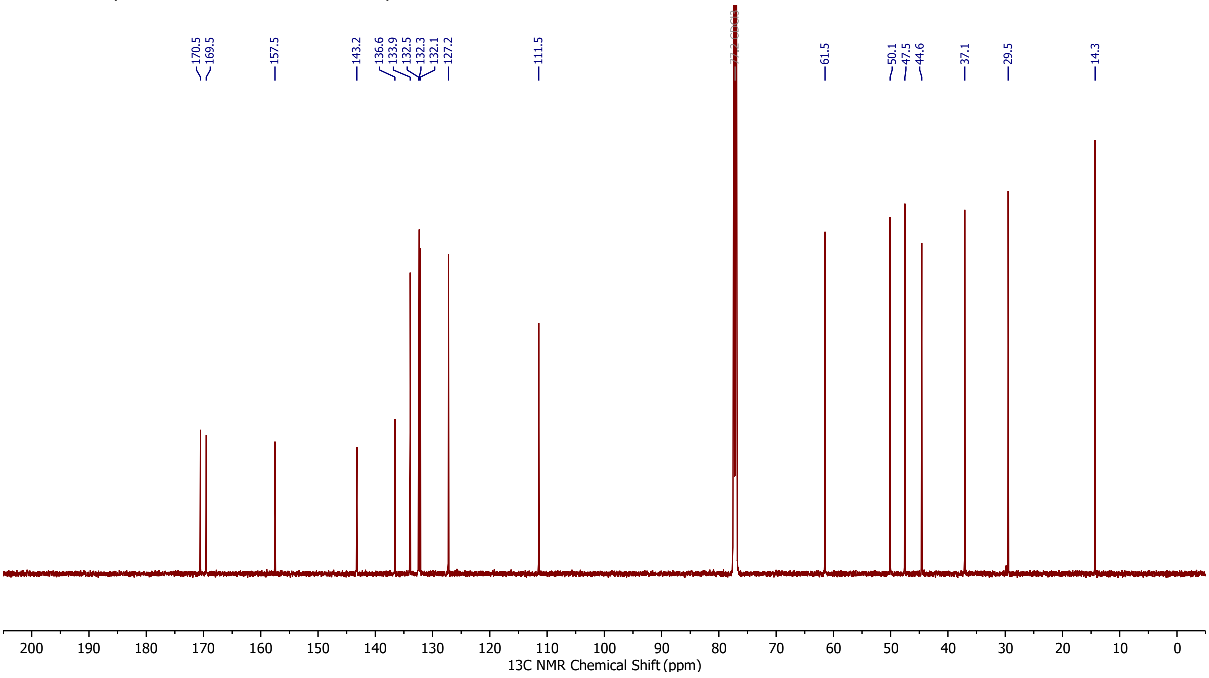

(±)-Ethyl (*Z*)-2-(2-((1-((2-chlorophenyl)sulfonyl)pyrrolidine-3-carbonyl)imino)-3-hydroxy-2,3-dihydrothiazol-4-yl)acetate (33b)

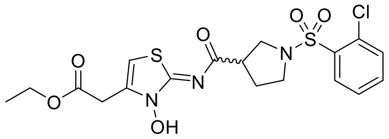

^1H NMR (600 MHz, 300 K, $CDCl_3$):

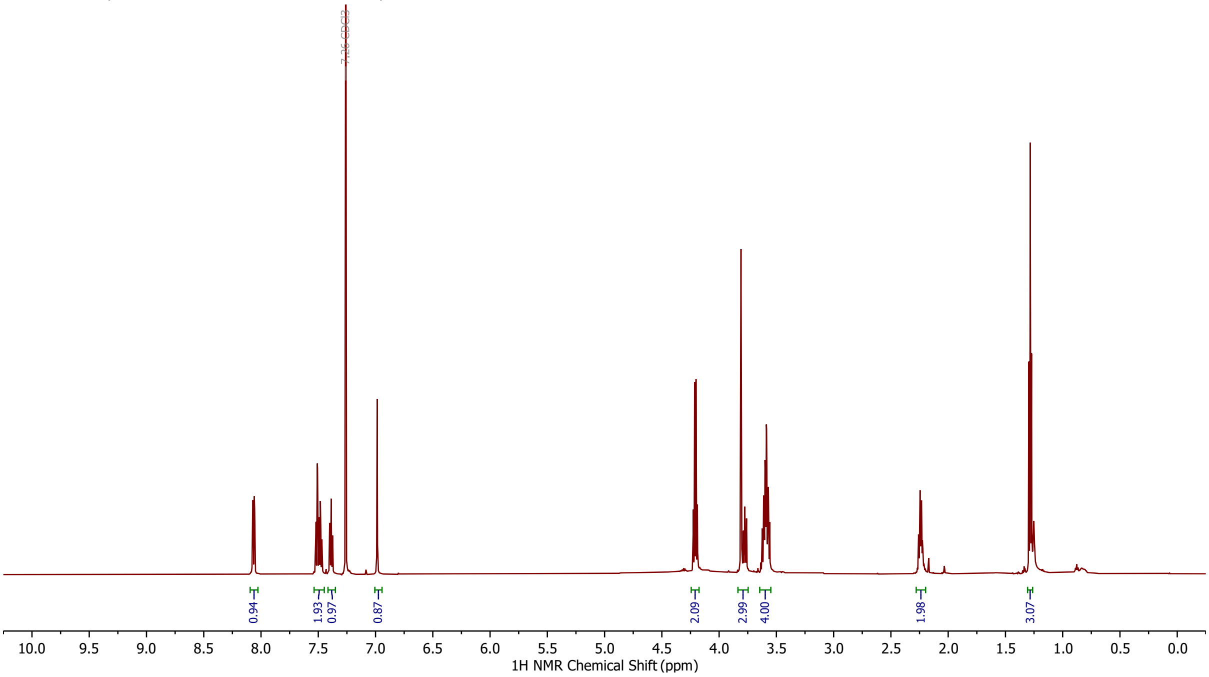

^{13}C NMR (151 MHz, 300 K, $CDCl_3$):

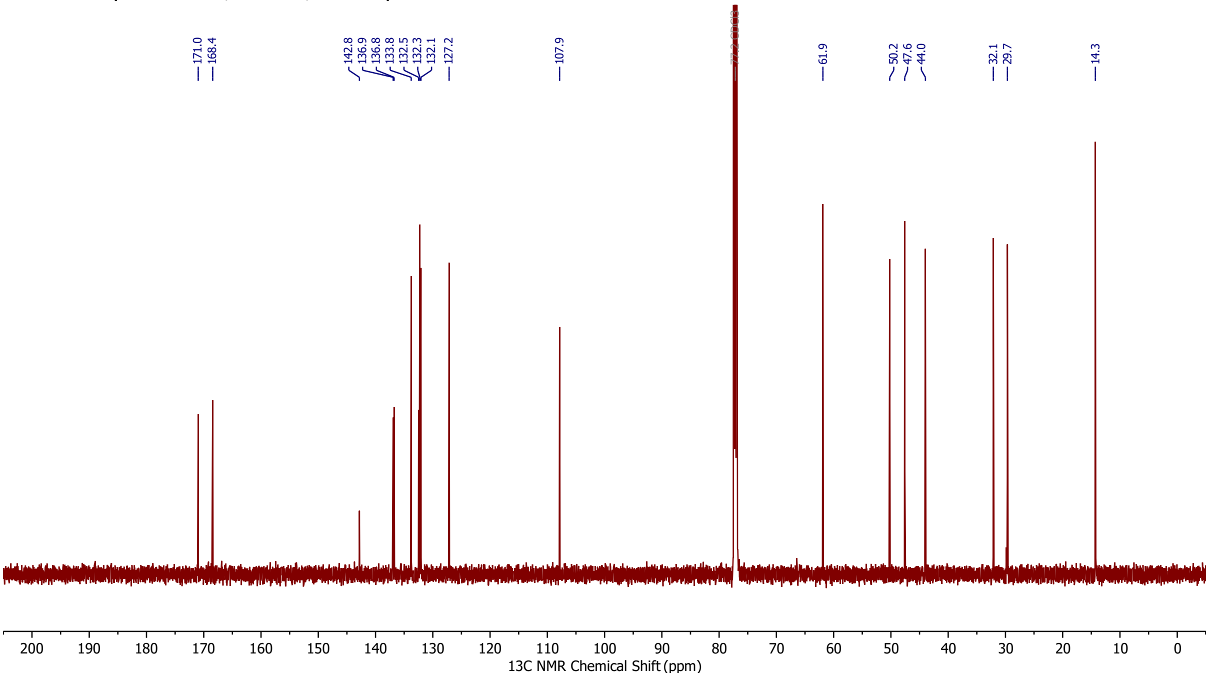

(±)-(*Z*)-2-(2-((1-((2-Chlorophenyl)sulfonyl)pyrrolidine-3-carbonyl)imino)-3-hydroxy-2,3-dihydrothiazol-4-yl)acetic acid (33)

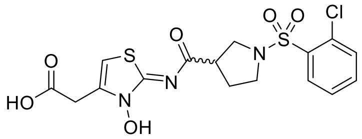

^{1}H NMR (600 MHz, 300 K, DMSO-d_6):

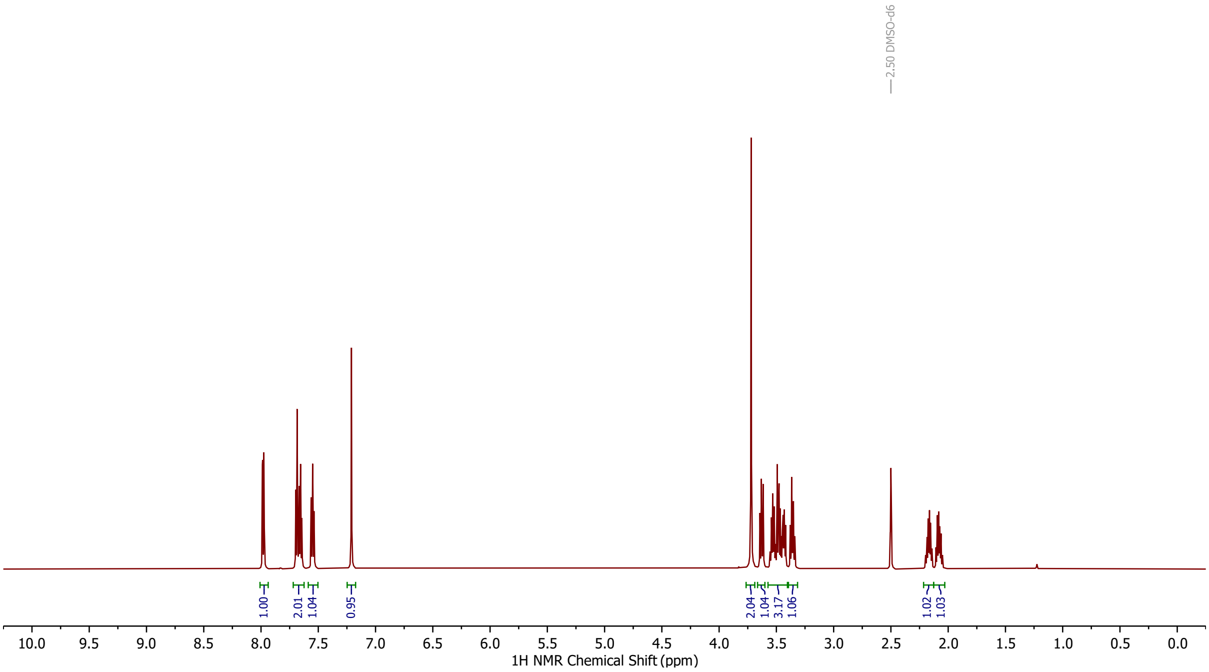

^{13}C NMR (151 MHz, 300 K, DMSO-d_6):

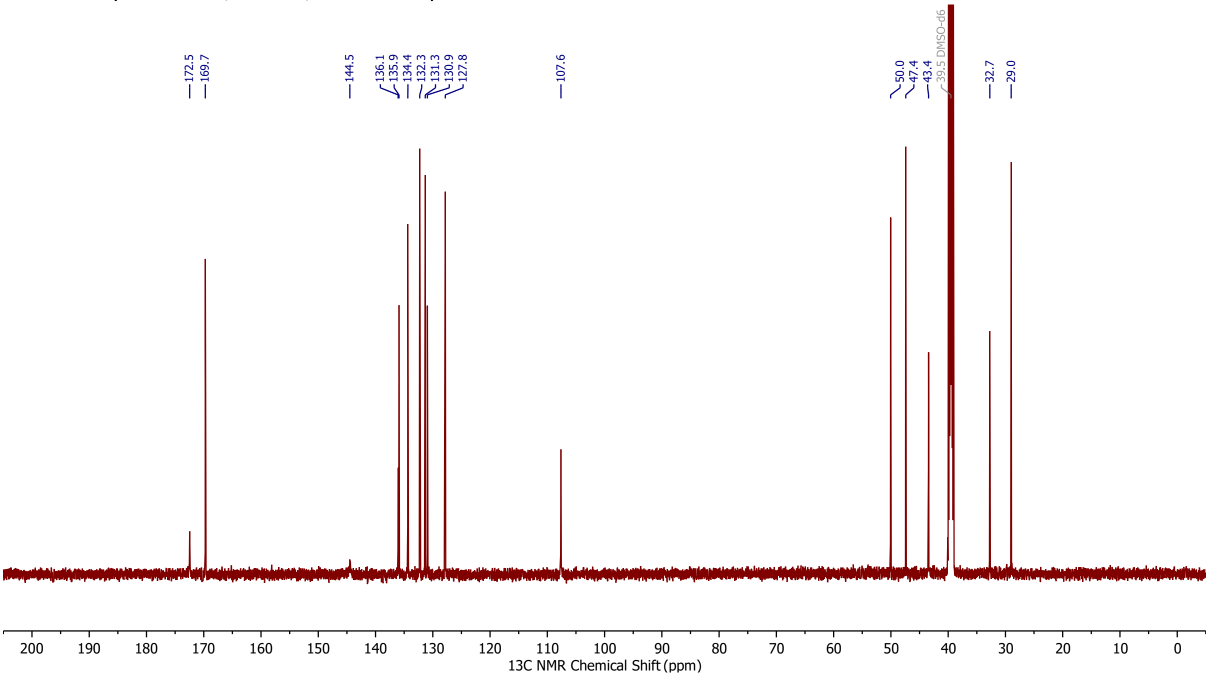

(±)-Ethyl 2-(2-(1-((3-chlorophenyl)sulfonyl)pyrrolidine-3-carboxamido)thiazol-4-yl)acetate (34a)

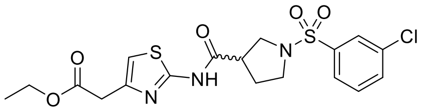

^{1}H NMR (600 MHz, 300 K, $CDCl_3$):

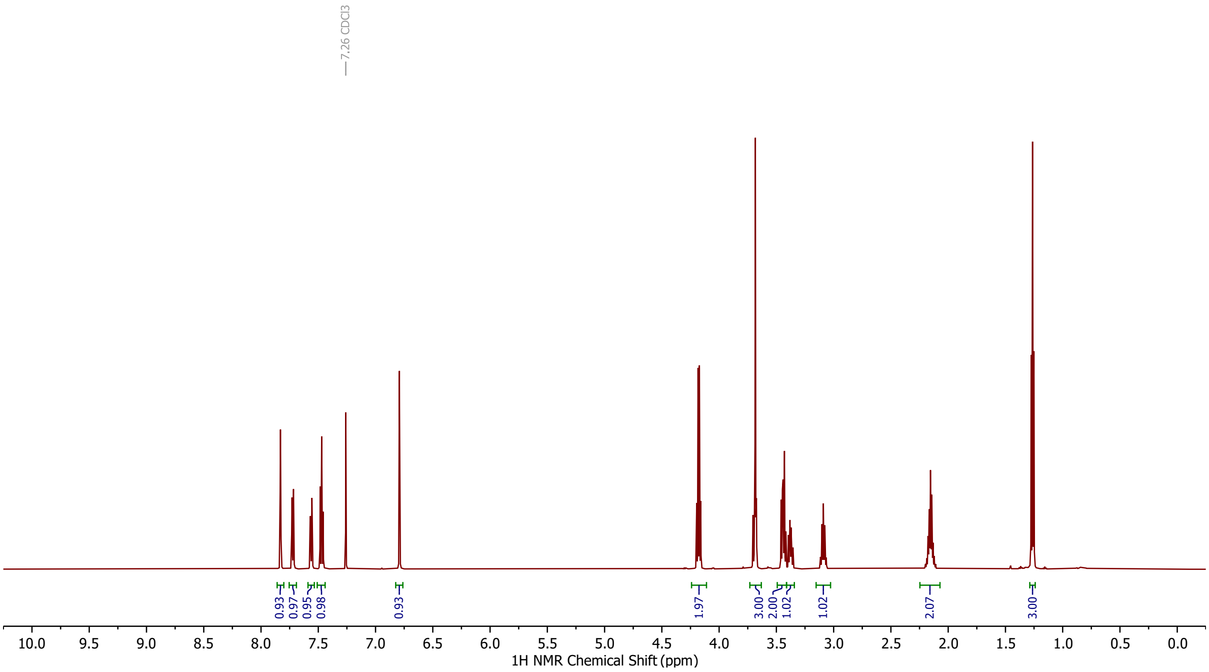

^{13}C NMR (151 MHz, 300 K, $CDCl_3$):

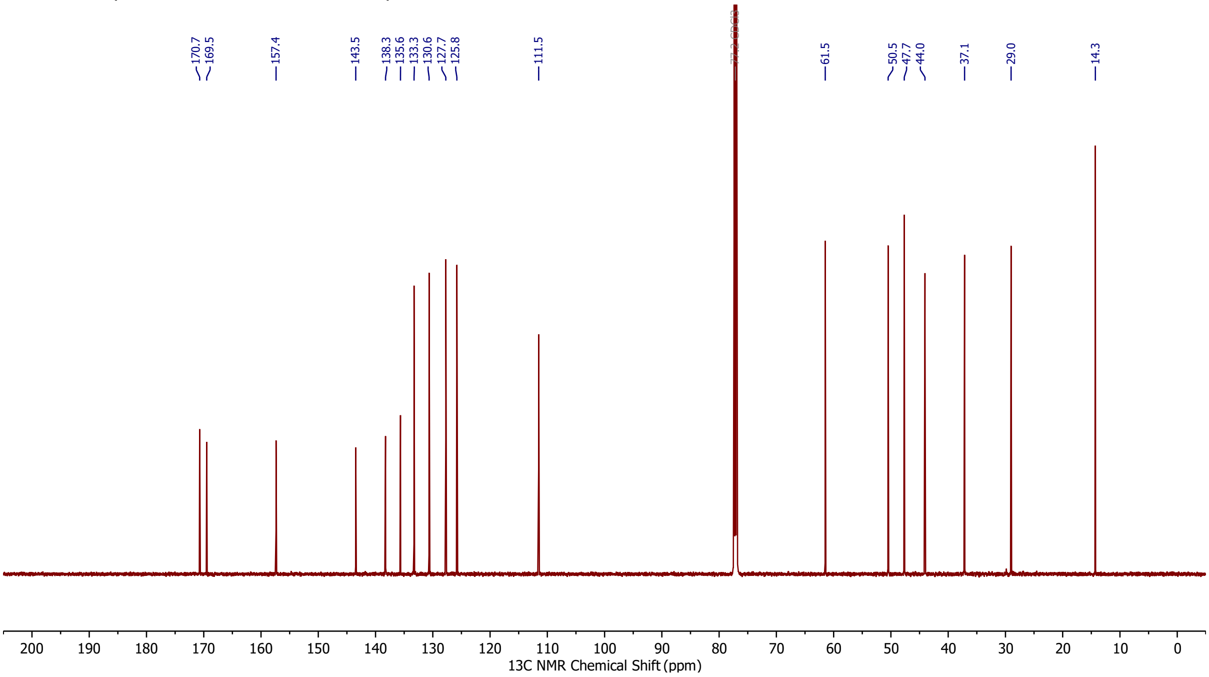

(±)-Ethyl (*Z*)-2-(2-((1-((3-chlorophenyl)sulfonyl)pyrrolidine-3-carbonyl)imino)-3-hydroxy-2,3-dihydrothiazol-4-yl)acetate (34b)

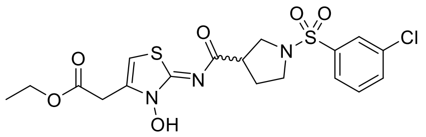

^{1}H NMR (600 MHz, 300 K, DMSO-d_6):

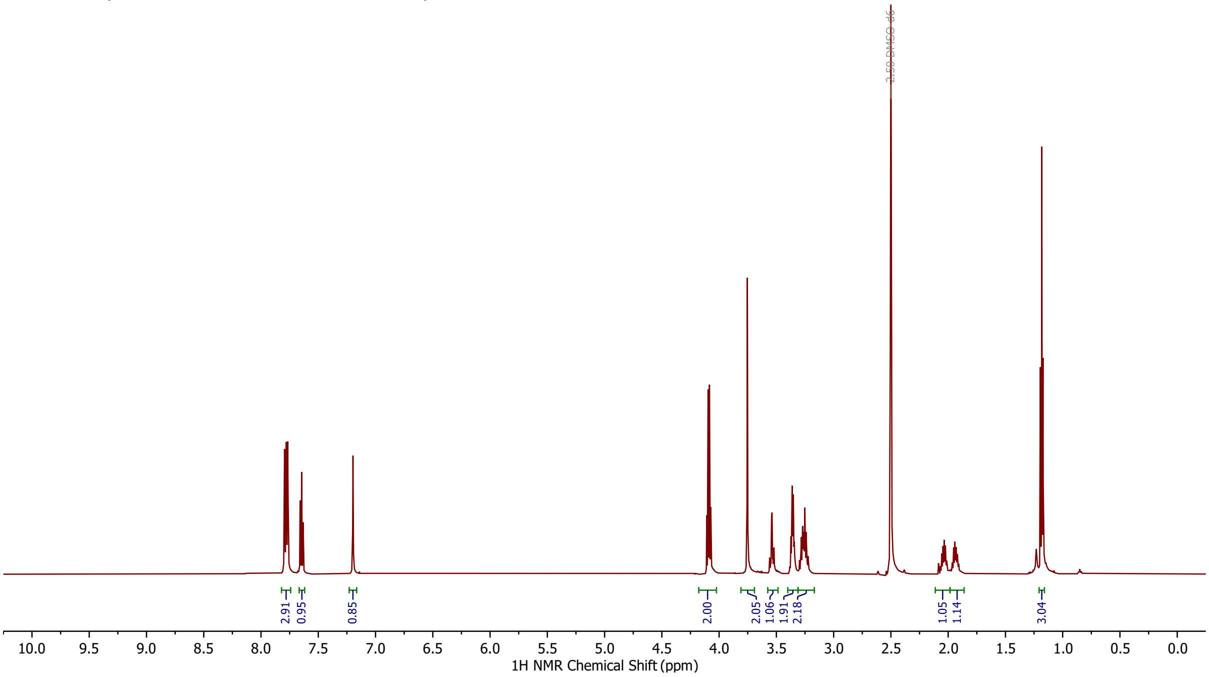

^{13}C NMR (151 MHz, 300 K, DMSO-d_6):

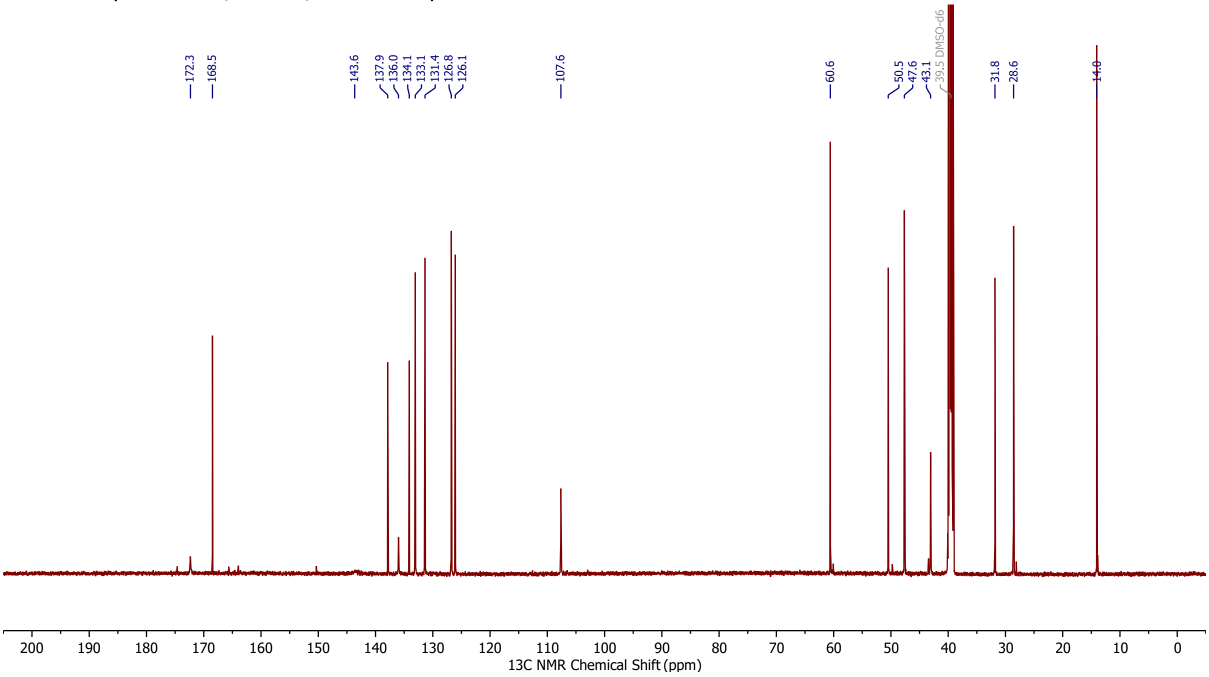

(±)-(*Z*)-2-(2-((1-((3-Chlorophenyl)sulfonyl)pyrrolidine-3-carbonyl)imino)-3-hydroxy-2,3-dihydrothiazol-4-yl)acetic acid (34)

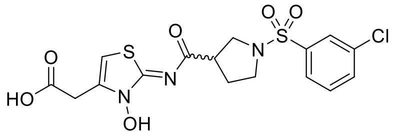

^{1}H NMR (600 MHz, 300 K, DMSO-d_6):

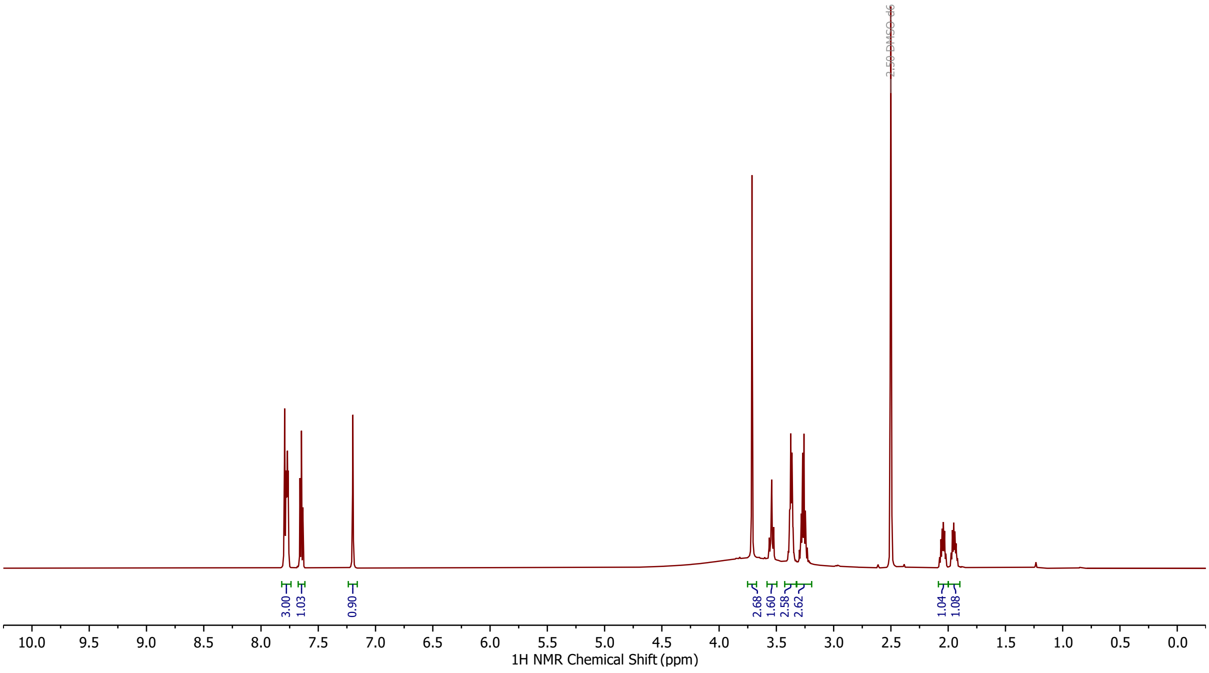

^{13}C NMR (151 MHz, 300 K, DMSO-d_6):

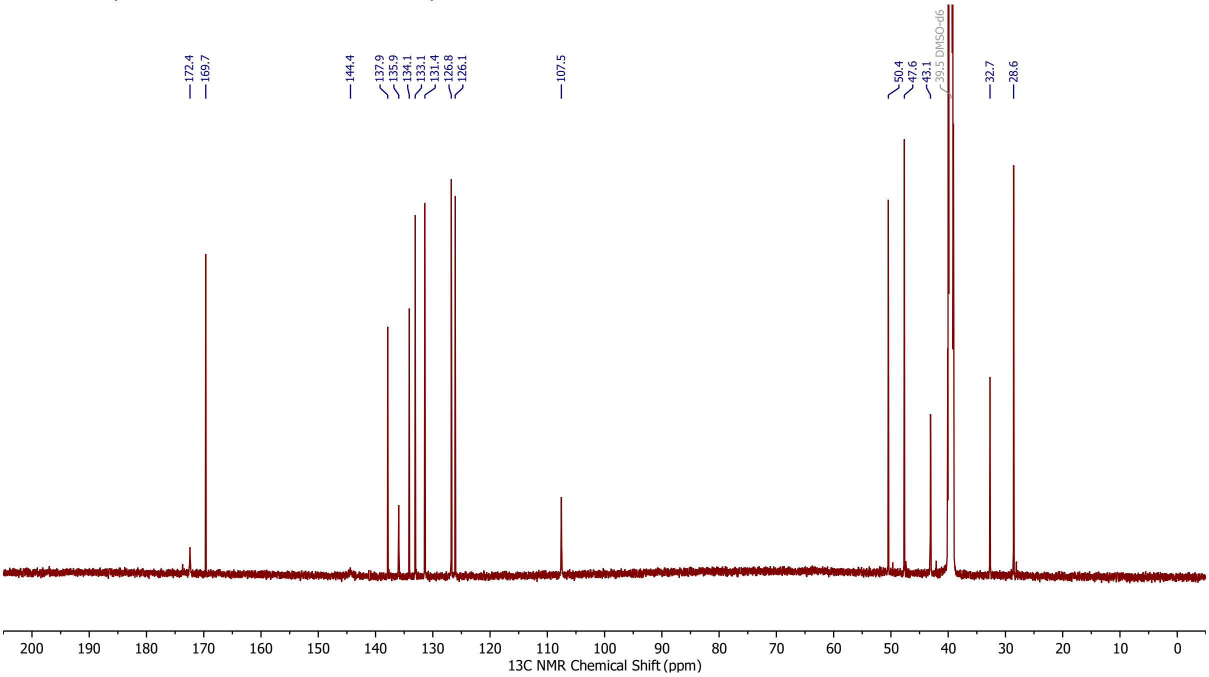

(±)-Ethyl 2-(2-(1-((4-chlorophenyl)sulfonyl)pyrrolidine-3-carboxamido)thiazol-4-yl)acetate (35a)

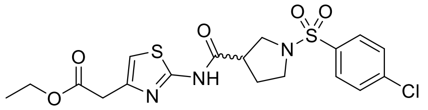

^{1}H NMR (600 MHz, 300 K, $CDCl_3$):

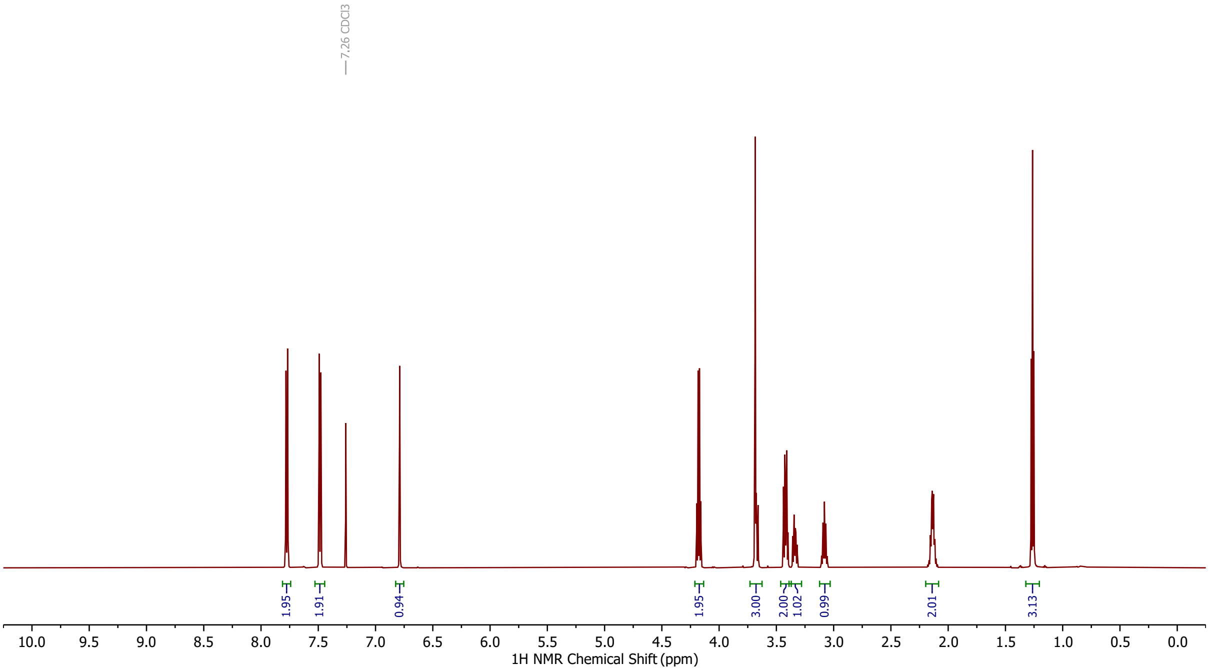

^{13}C NMR (151 MHz, 300 K, $CDCl_3$):

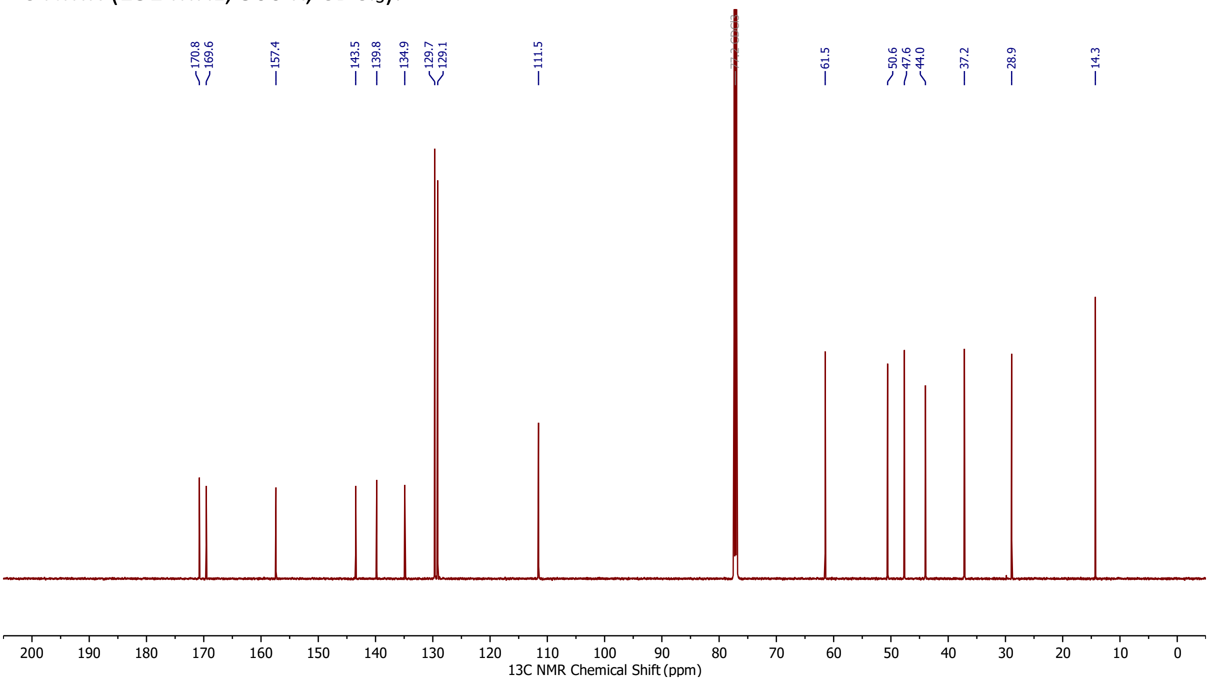

(±)-Ethyl (*Z*)-2-(2-((1-((4-chlorophenyl)sulfonyl)pyrrolidine-3-carbonyl)imino)-3-hydroxy-2,3-dihydrothiazol-4-yl)acetate (35b)

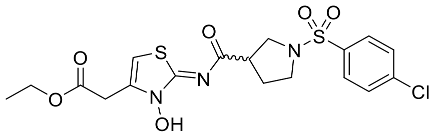

^{1}H NMR (600 MHz, 300 K, $CDCl_3$):

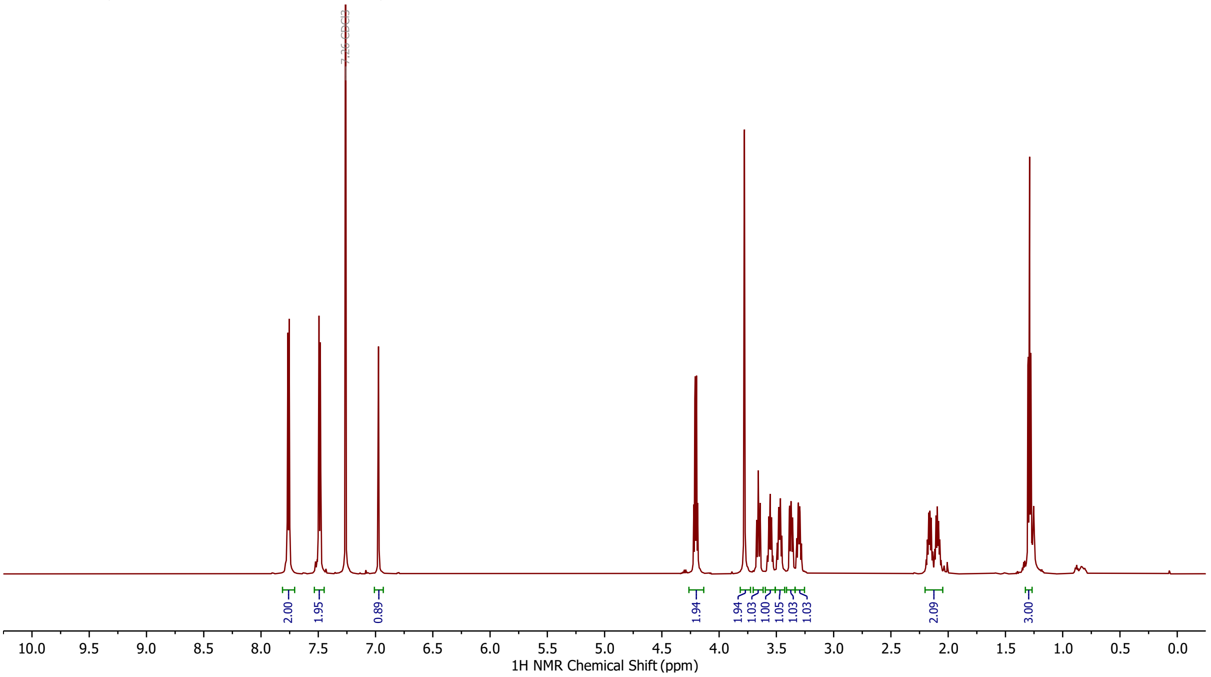

^{13}C NMR (151 MHz, 300 K, $CDCl_3$):

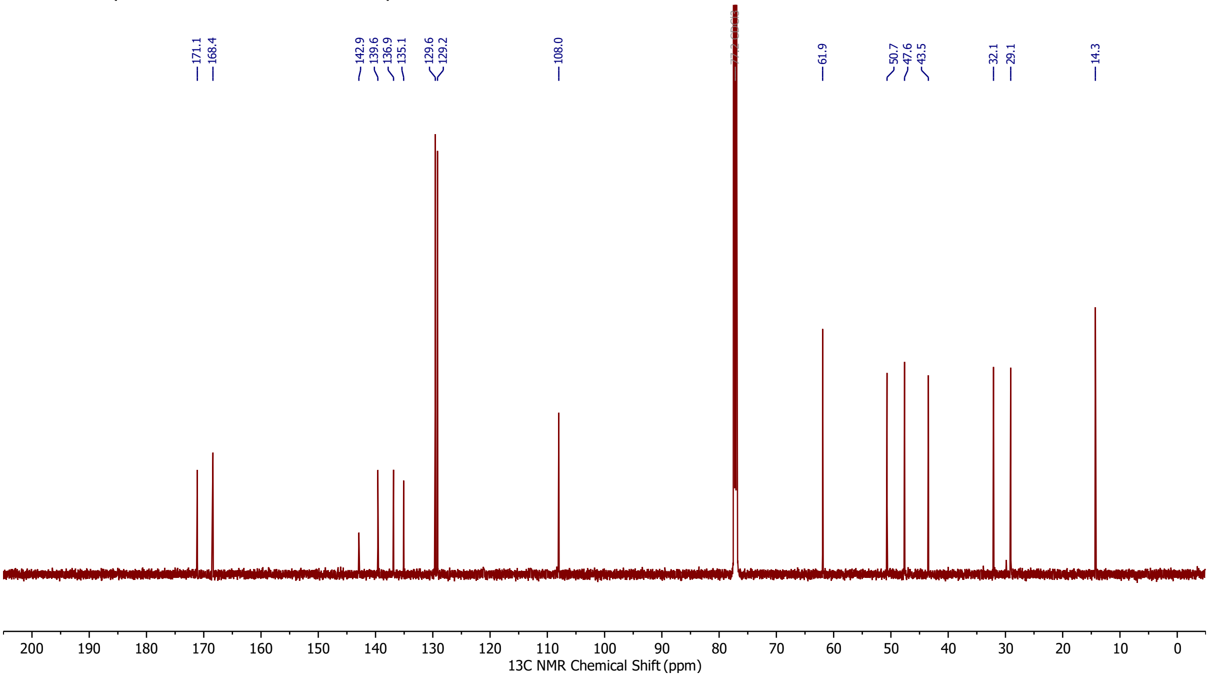

(±)-(*Z*)-2-(2-((1-((4-Chlorophenyl)sulfonyl)pyrrolidine-3-carbonyl)imino)-3-hydroxy-2,3-dihydrothiazol-4-yl)acetic acid (35)

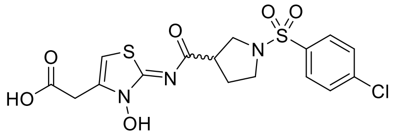

^{1}H NMR (600 MHz, 300 K, DMSO-d_6):

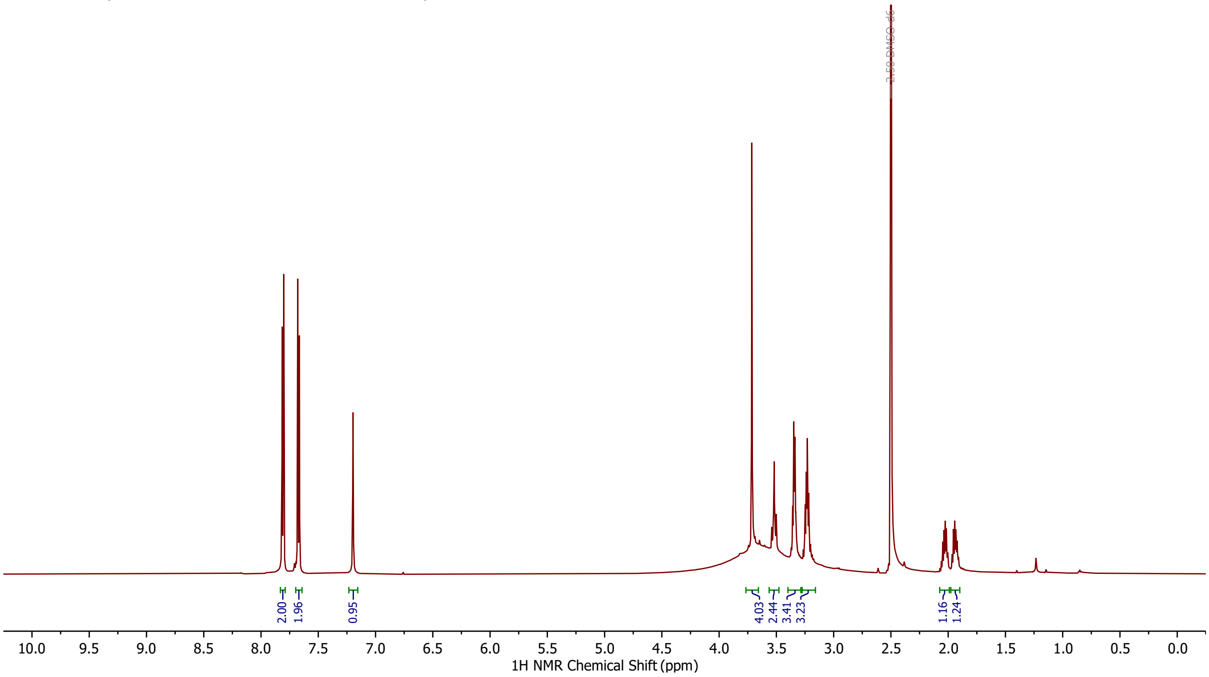

^{13}C NMR (151 MHz, 300 K, DMSO-d_6):

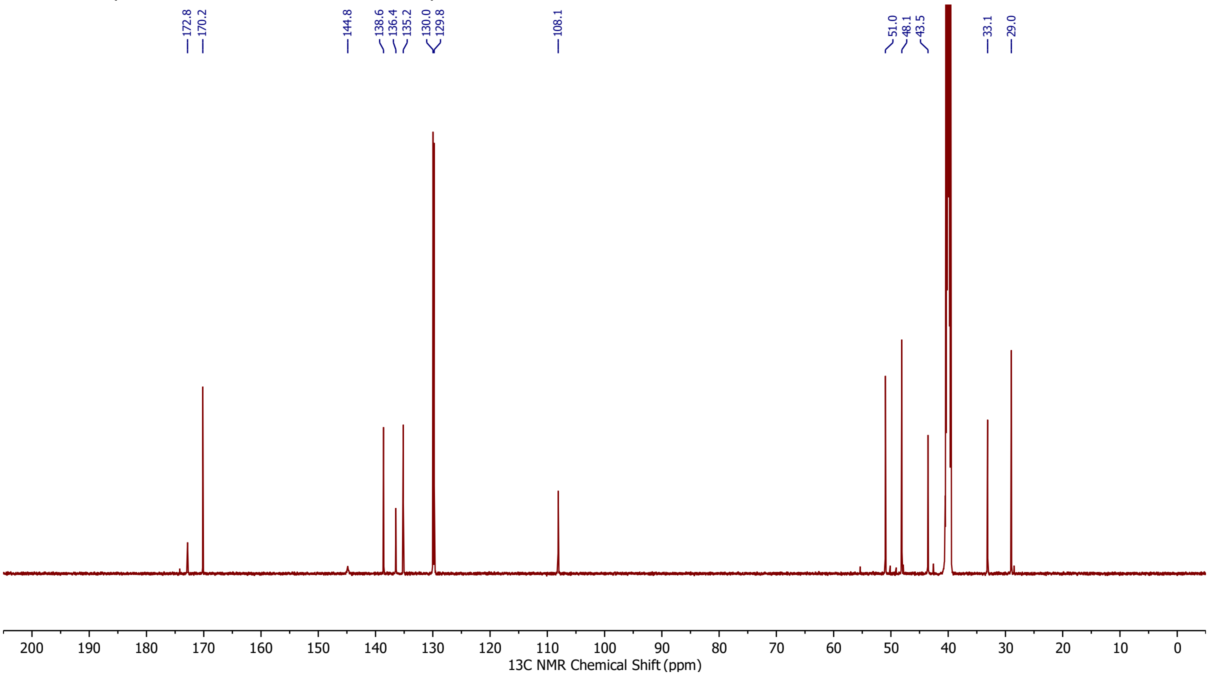

(±)-Ethyl 2-(2-(1-((4-methoxyphenyl)sulfonyl)pyrrolidine-3-carboxamido)thiazol-4-yl)acetate (36a)

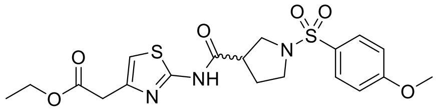

^{1}H NMR (600 MHz, 300 K, $CDCl_3$):

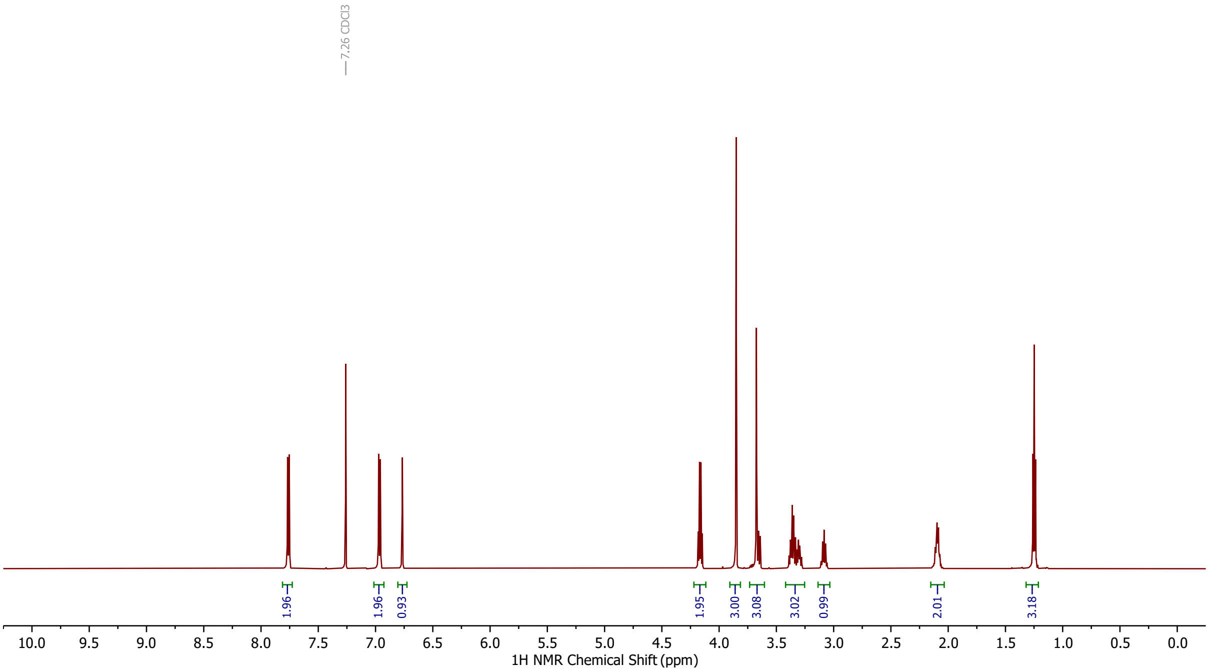

^{13}C NMR (151 MHz, 300 K, $CDCl_3$):

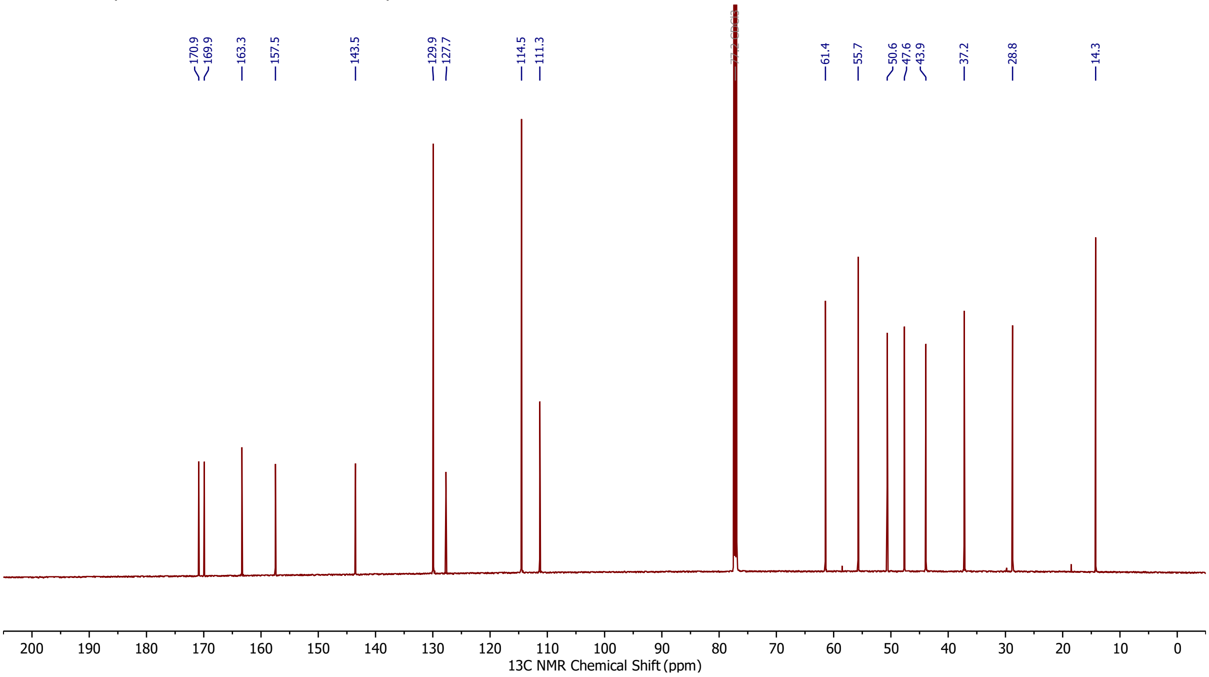

(±)-Ethyl (*Z*)-2-(3-hydroxy-2-((1-((4-methoxyphenyl)sulfonyl)pyrrolidine-3-carbonyl)imino)-2,3-dihydrothiazol-4-yl)acetate (36b)

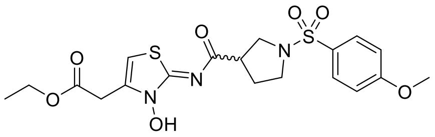

^{1}H NMR (600 MHz, 300 K, $CDCl_3$):

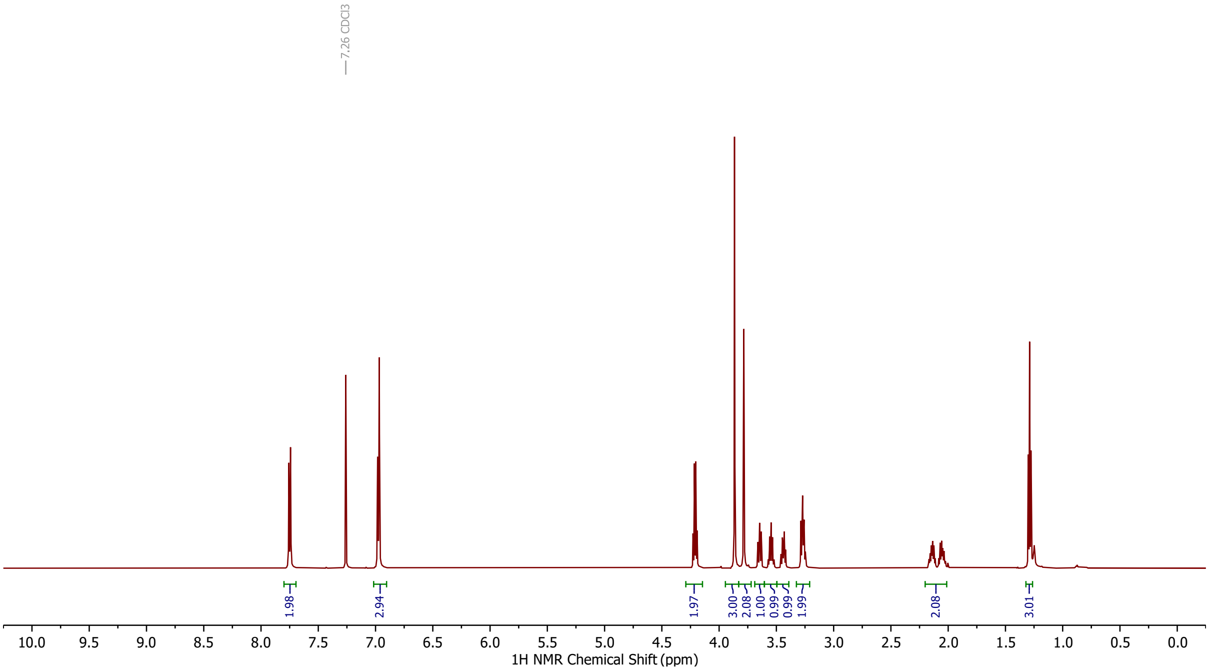

^{13}C NMR (151 MHz, 300 K, $CDCl_3$):

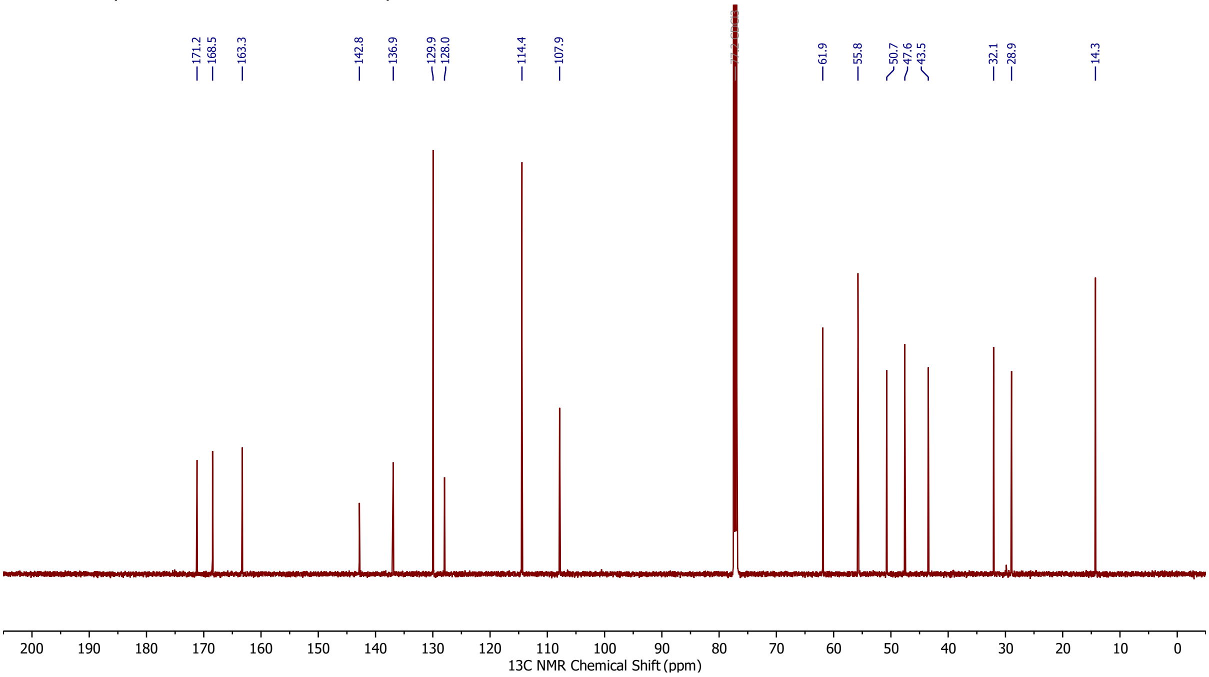

(±)-(*Z*)-2-(3-Hydroxy-2-((1-((4-methoxyphenyl)sulfonyl)pyrrolidine-3-carbonyl)imino)-2,3-dihydrothiazol-4-yl)acetic acid (36)

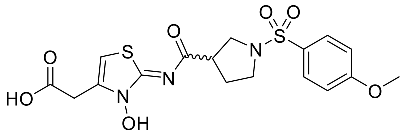

^{1}H NMR (600 MHz, 300 K, DMSO-d_6):

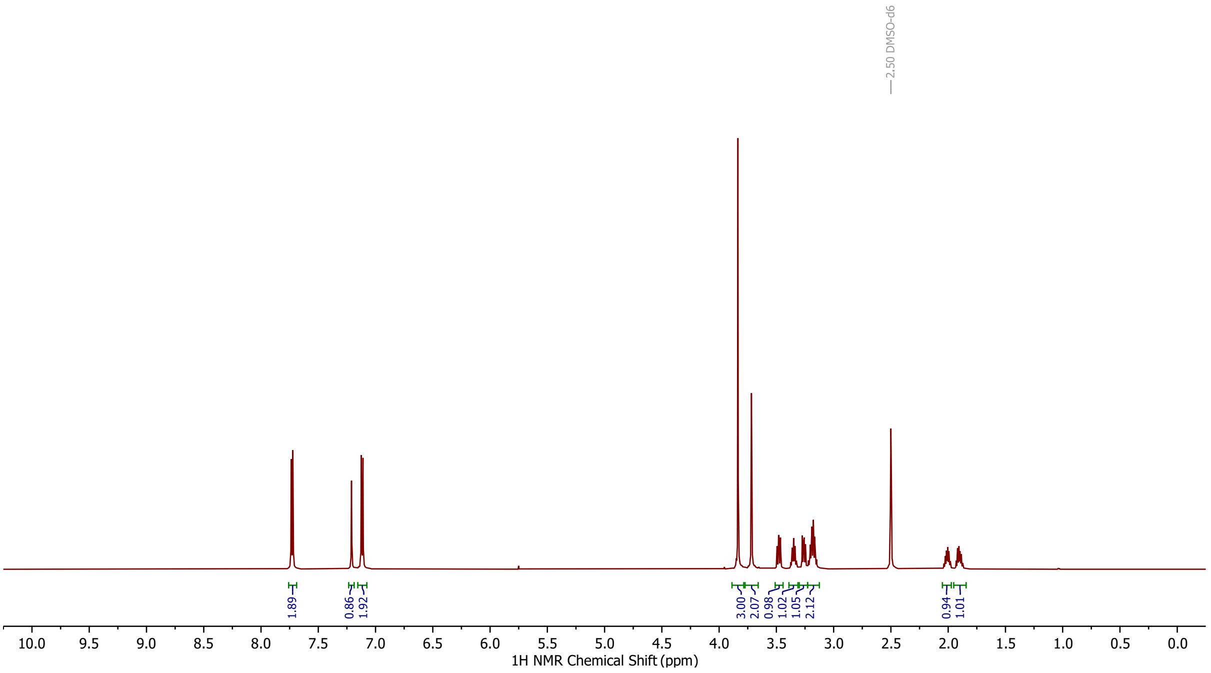

^{13}C NMR (151 MHz, 300 K, DMSO-d_6):

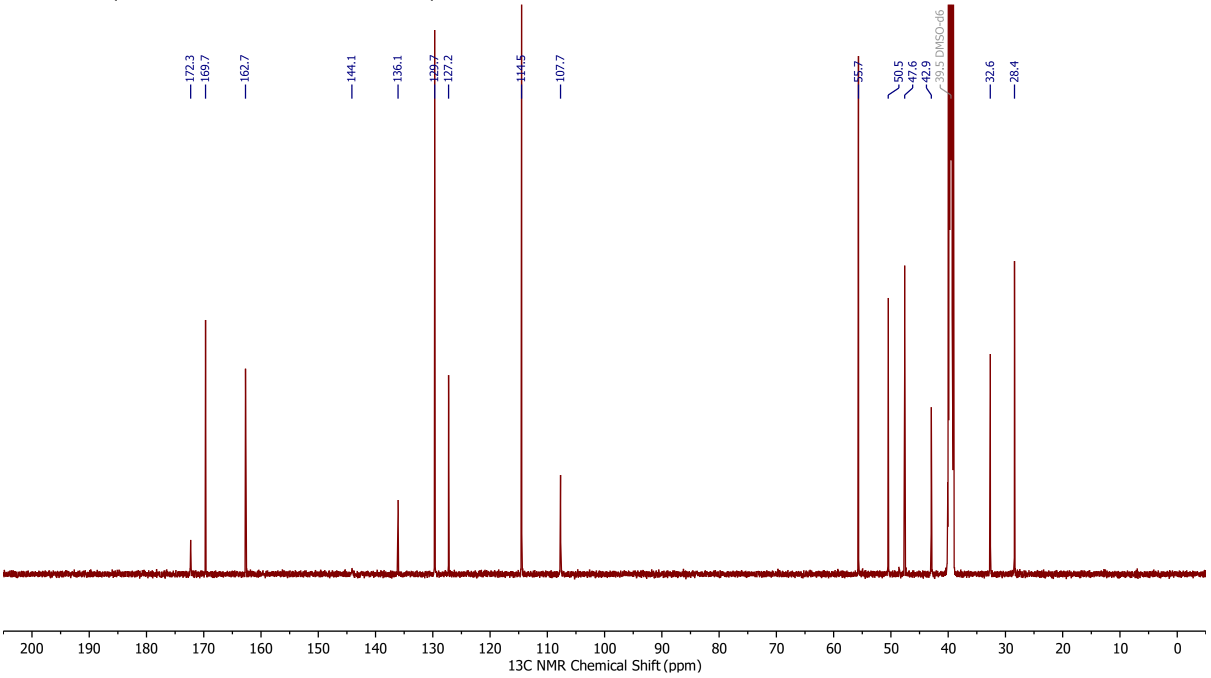

(±)-Ethyl 2-(2-(1-((4-(trifluoromethyl)phenyl)sulfonyl)pyrrolidine-3-carboxamido)thiazol-4-yl)acetate (37a)

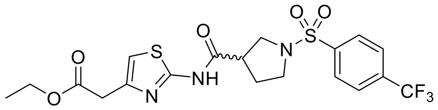

^{1}H NMR (600 MHz, 300 K, CDCl$_3$):

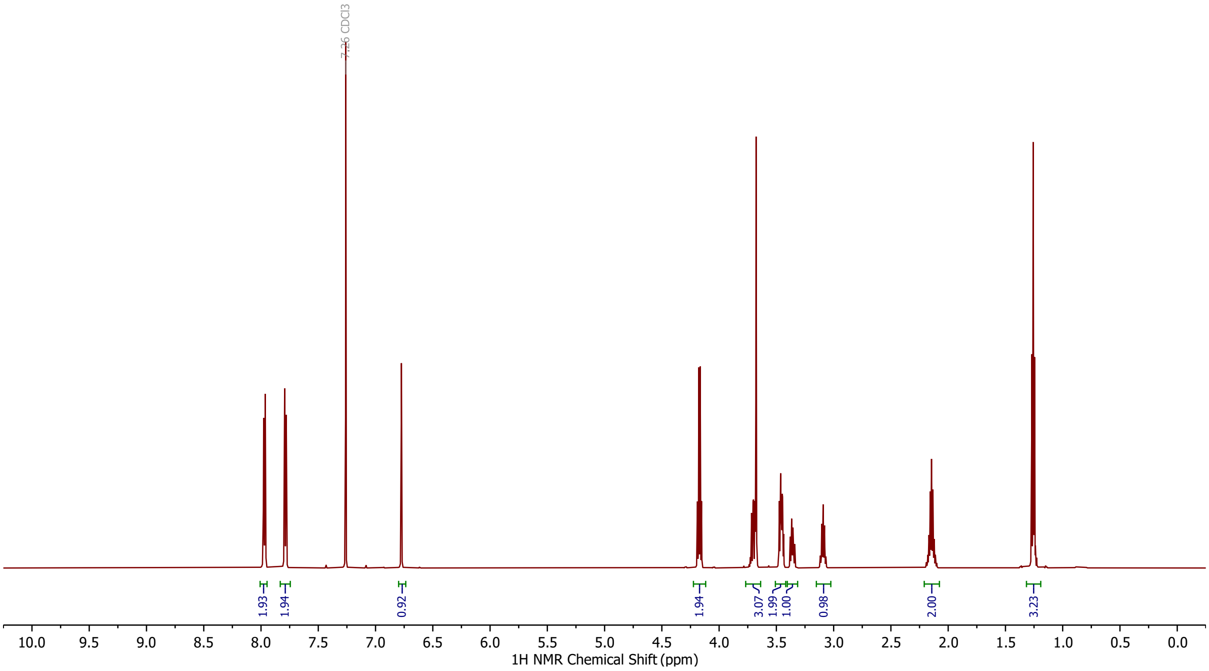

^{13}C NMR (151 MHz, 300 K, CDCl$_3$):

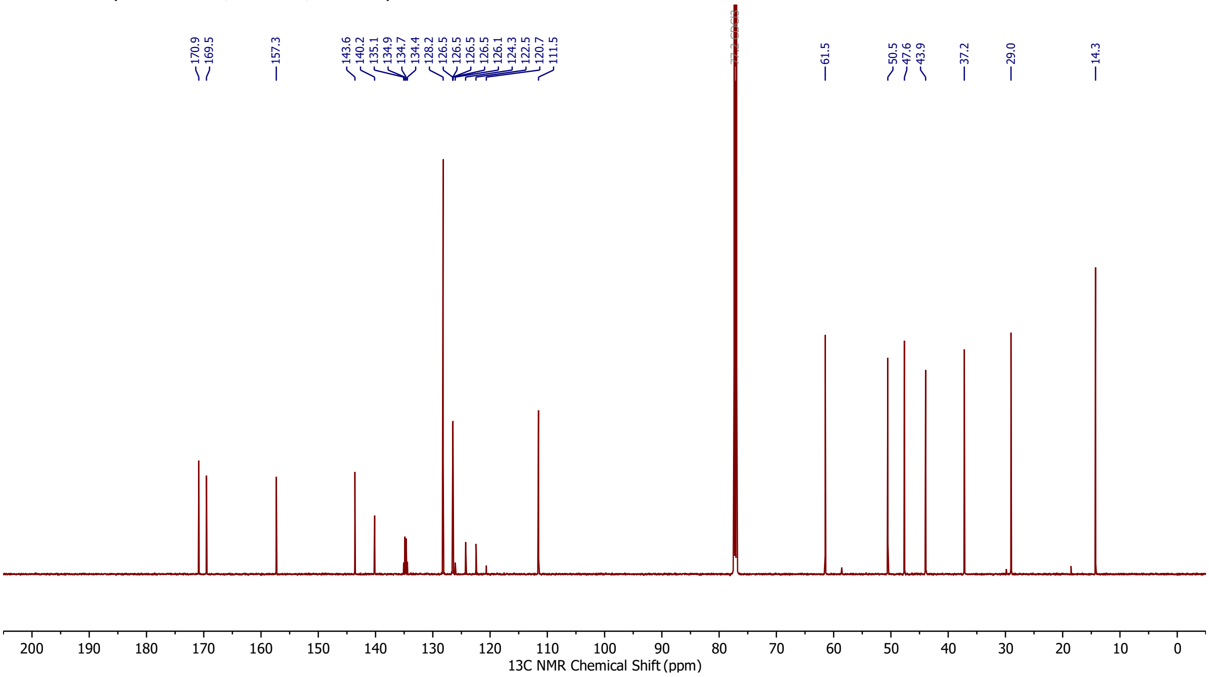

(±)-Ethyl (*Z*)-2-(3-hydroxy-2-((1-((4-(trifluoromethyl)phenyl)sulfonyl)pyrrolidine-3-carbonyl)imino)-2,3-dihydrothiazol-4-yl)acetate (37b)

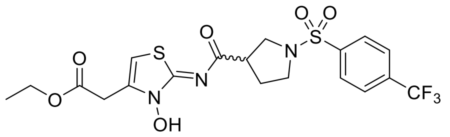

^{1}H NMR (600 MHz, 300 K, DMSO-d_6):

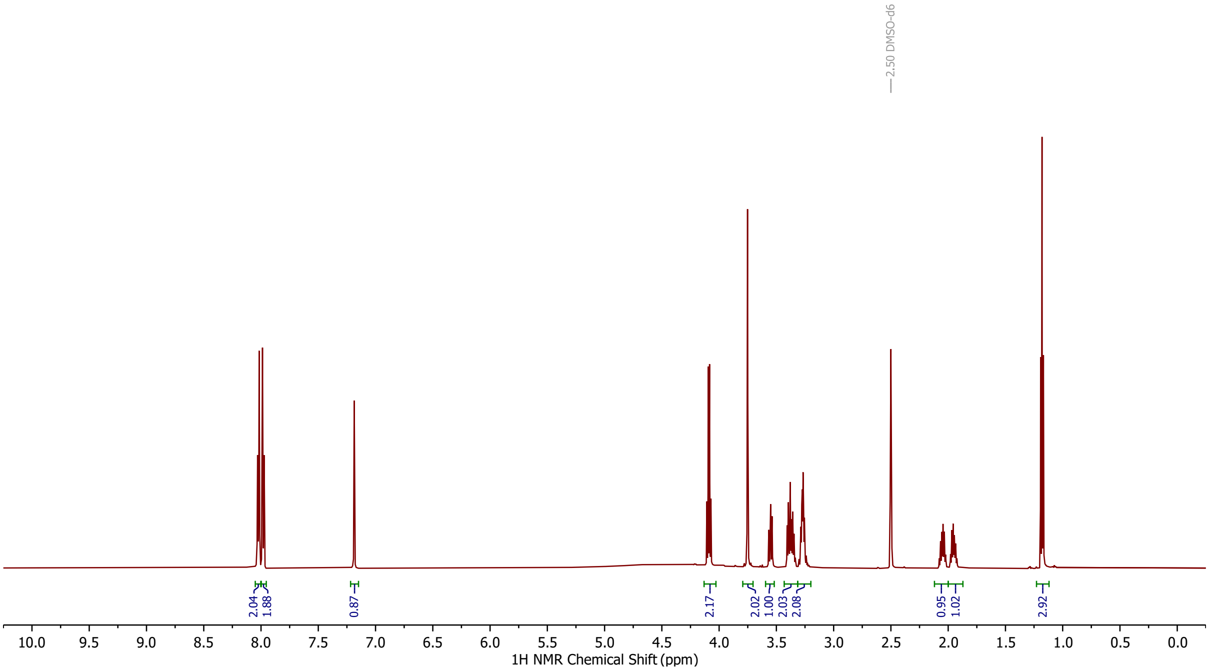

^{13}C NMR (151 MHz, 300 K, DMSO-d_6):

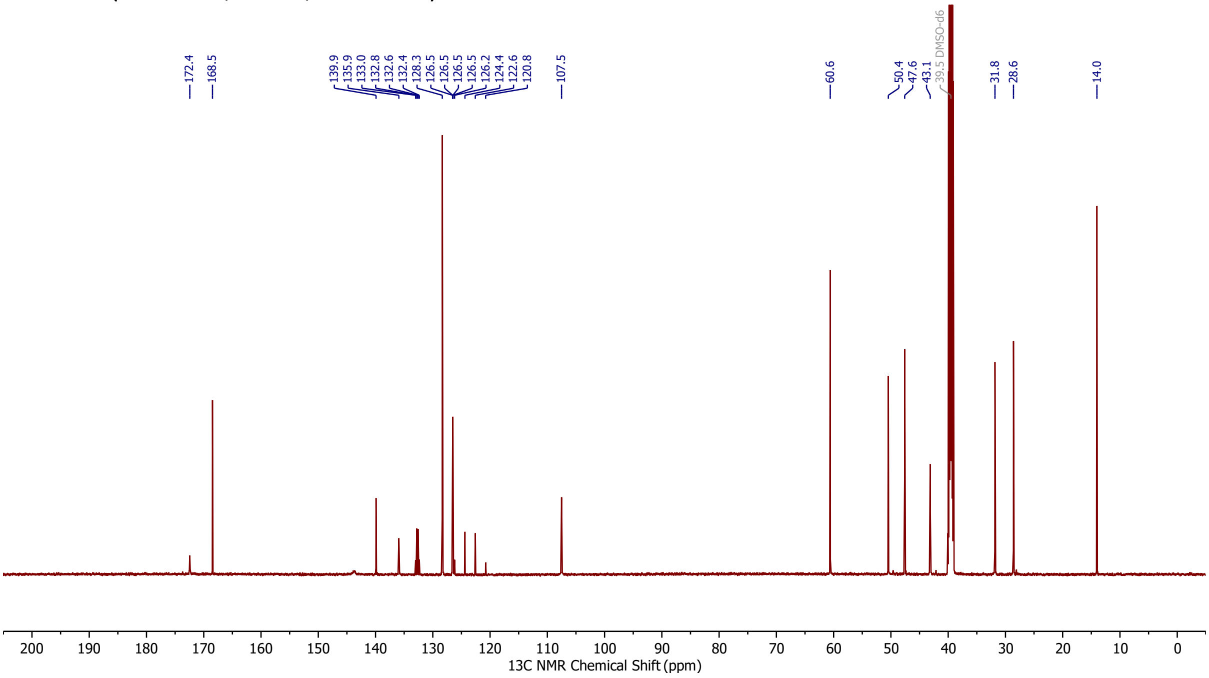

(±)-(*Z*)-2-(3-Hydroxy-2-((1-((4-(trifluoromethyl)phenyl)sulfonyl)pyrrolidine-3-carbonyl)imino)-2,3-dihydrothiazol-4-yl)acetic acid (37)

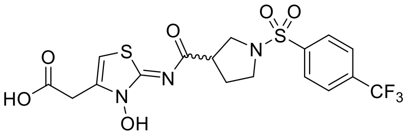

^{1}H NMR (600 MHz, 300 K, DMSO-d_6):

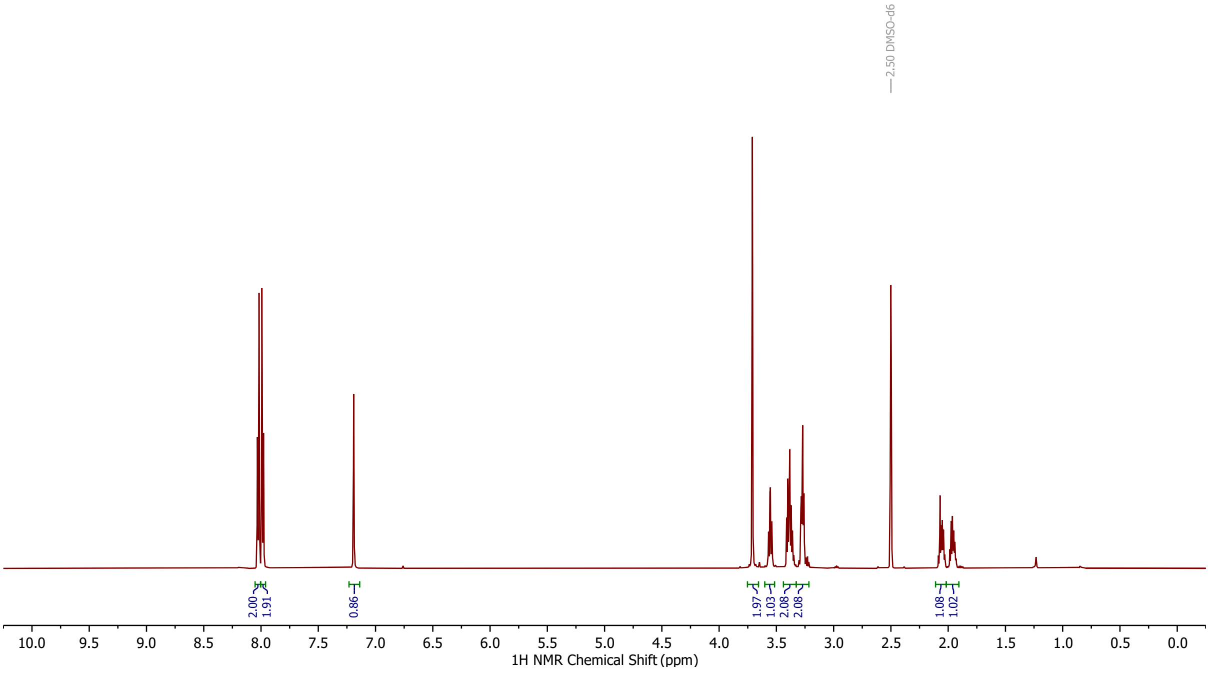

^{13}C NMR (151 MHz, 300 K, DMSO-d_6):

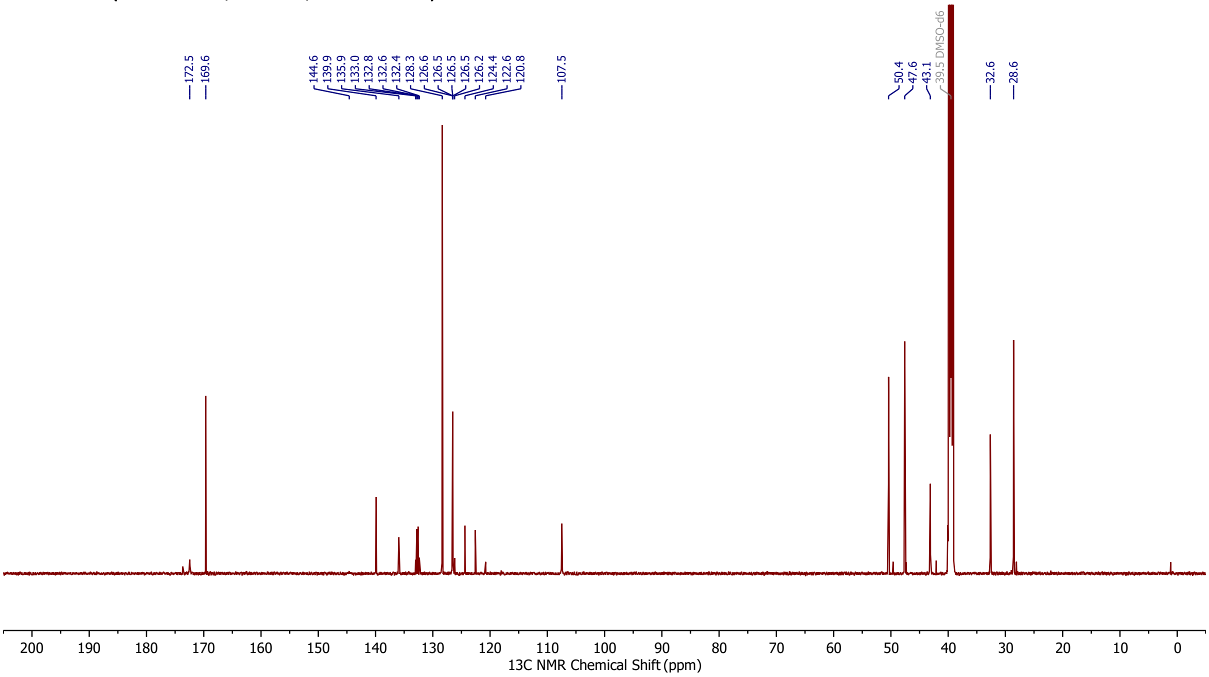

(±)-Ethyl 2-(2-(1-([1,1'-biphenyl]-4-ylsulfonyl)pyrrolidine-3-carboxamido)thiazol-4-yl)acetate (38a)

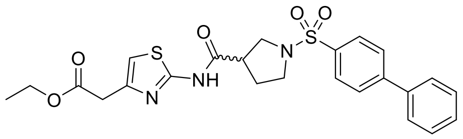

^{1}H NMR (600 MHz, 300 K, $CDCl_3$):

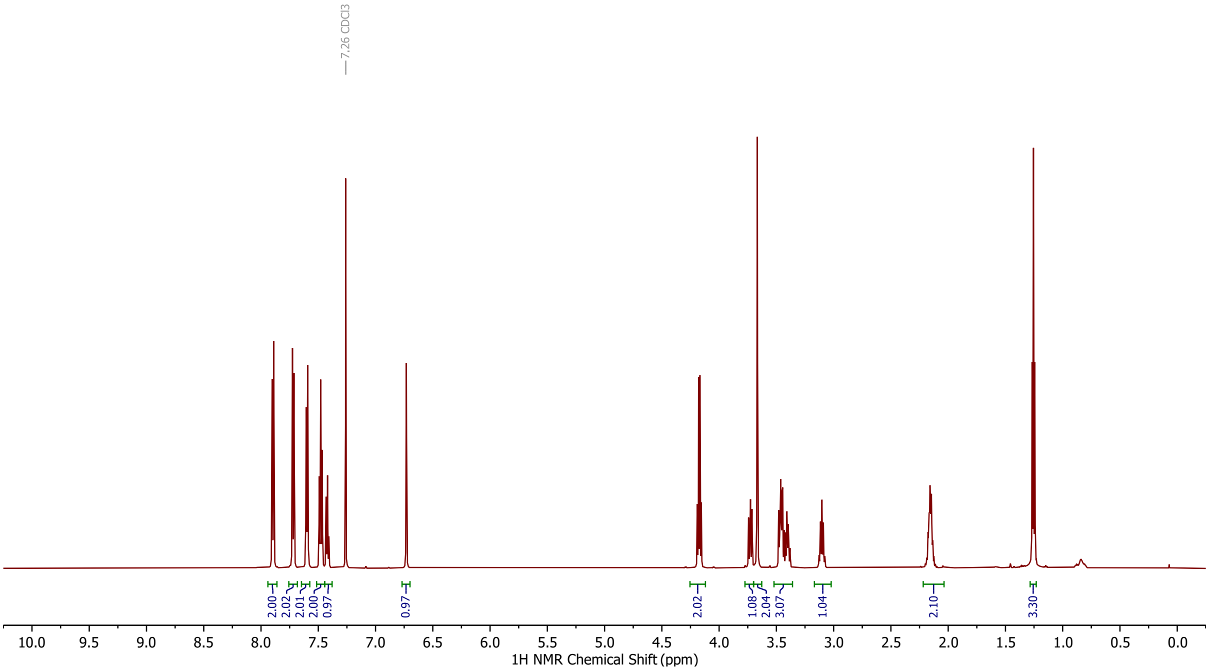

^{13}C NMR (151 MHz, 300 K, $CDCl_3$):

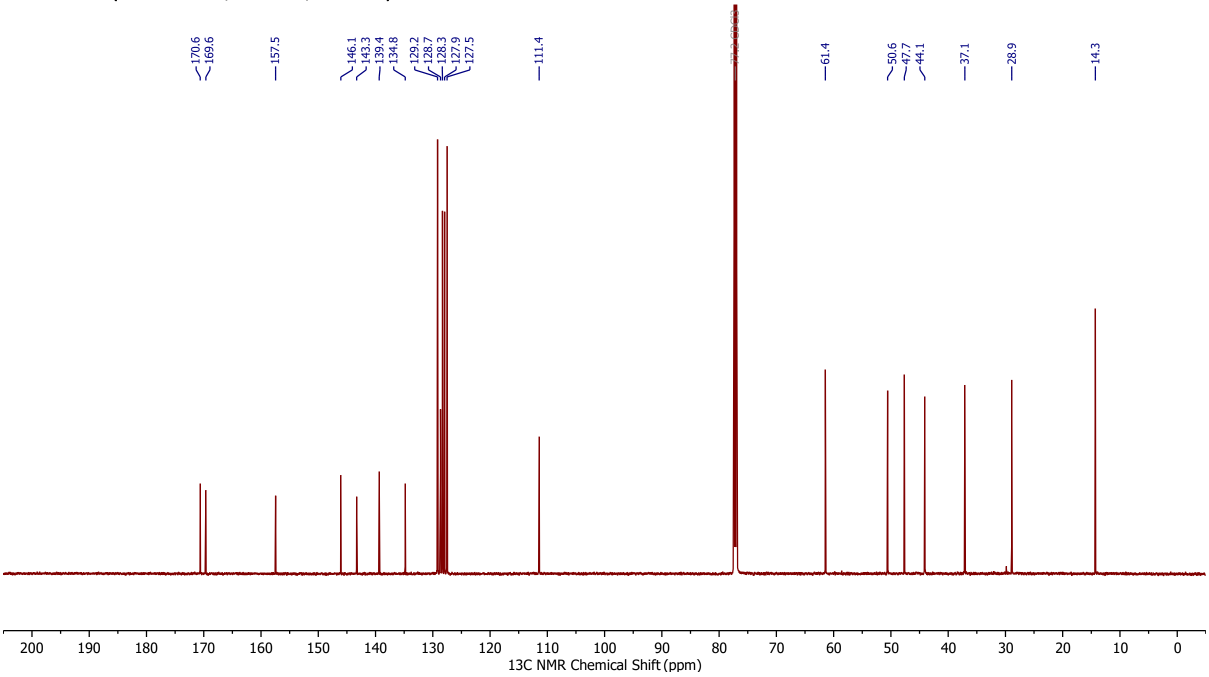

(±)-Ethyl (*Z*)-2-(2-((1-([1,1'-biphenyl]-4-ylsulfonyl)pyrrolidine-3-carbonyl)imino)-3-hydroxy-2,3-dihydrothiazol-4-yl)acetate (38b)

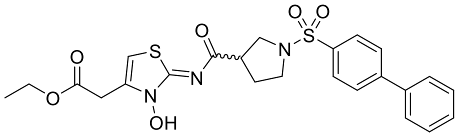

^{1}H NMR (600 MHz, 300 K, $CDCl_3$):

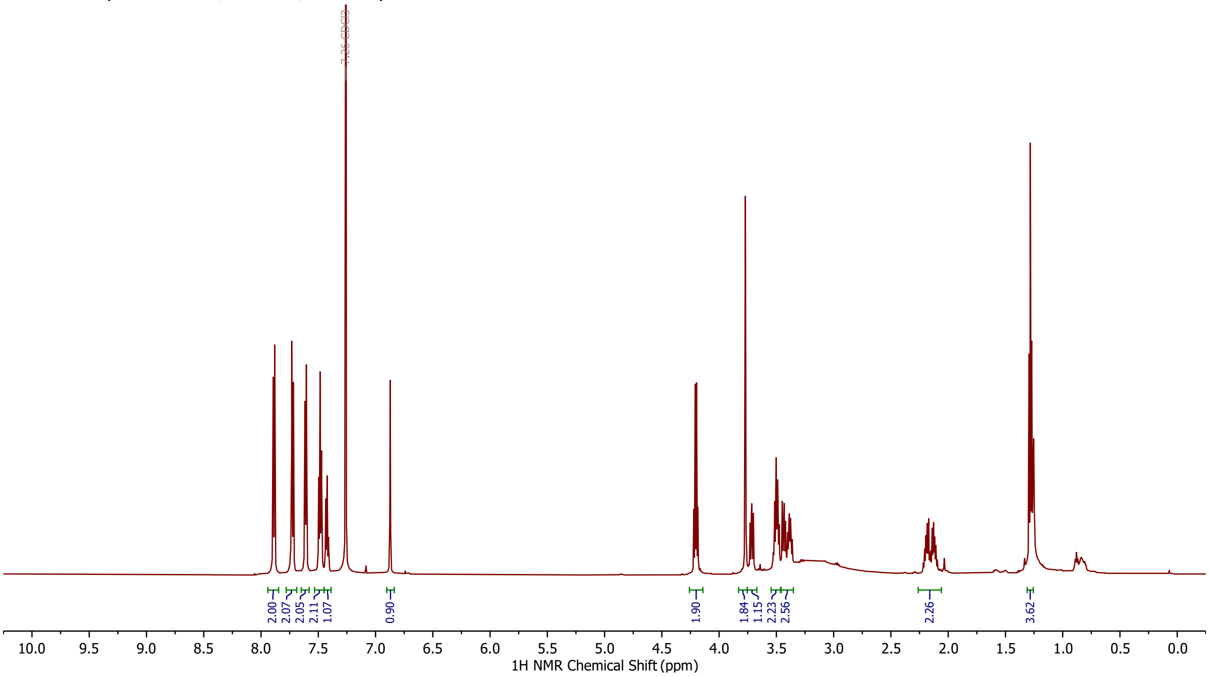

^{13}C NMR (151 MHz, 300 K, $CDCl_3$):

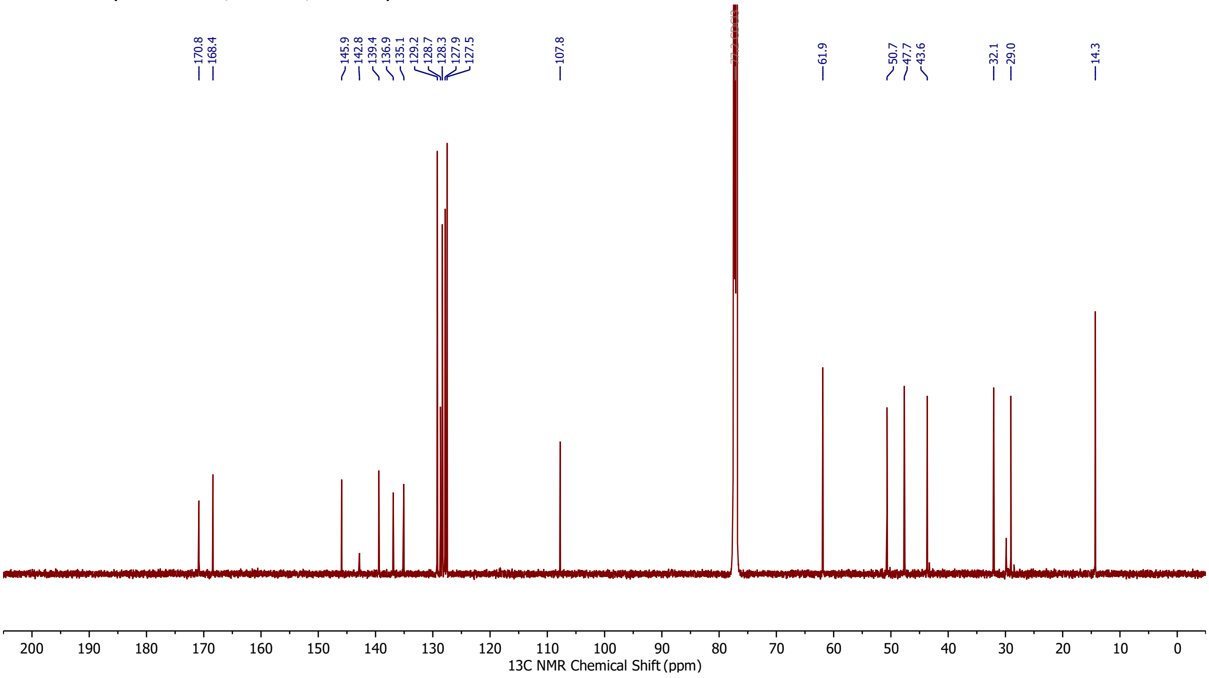

(±)-(*Z*)-2-(2-((1-([1,1'-Biphenyl]-4-ylsulfonyl)pyrrolidine-3-carbonyl)imino)-3-hydroxy-2,3-dihydrothiazol-4-yl)acetic acid (38)

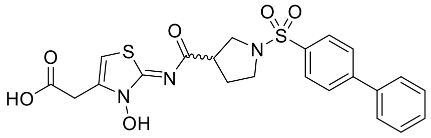

^{1}H NMR (600 MHz, 300 K, DMSO-d_6):

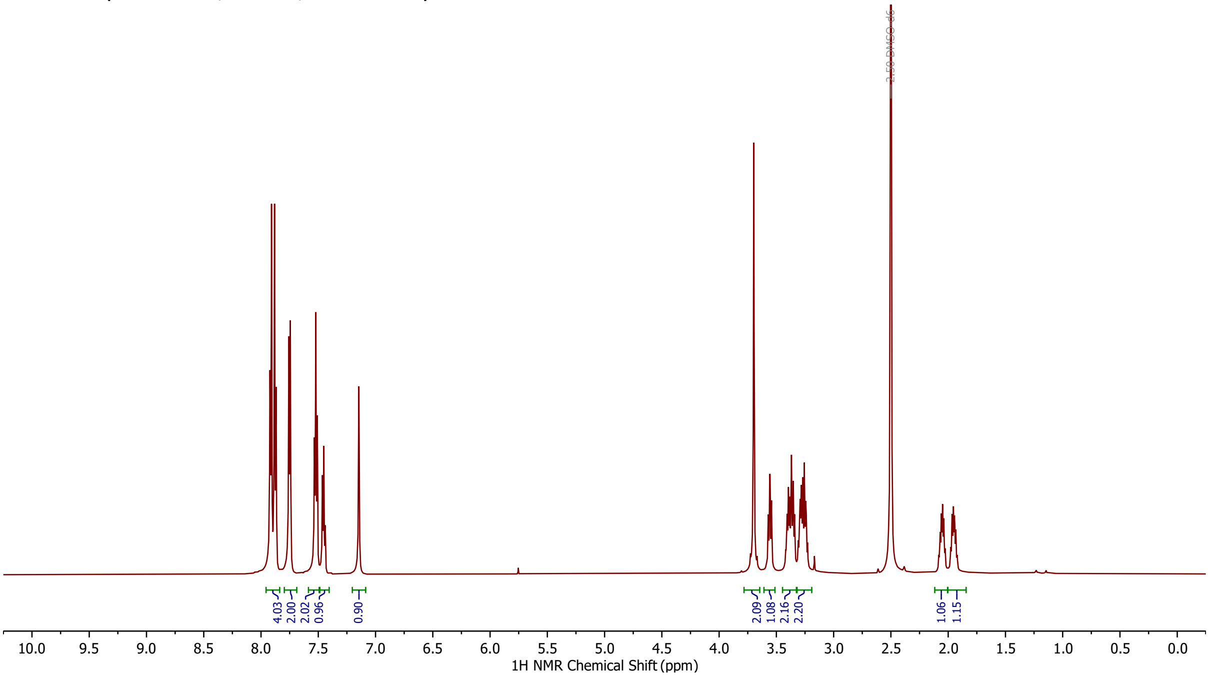

^{13}C NMR (151 MHz, 300 K, DMSO-d_6):

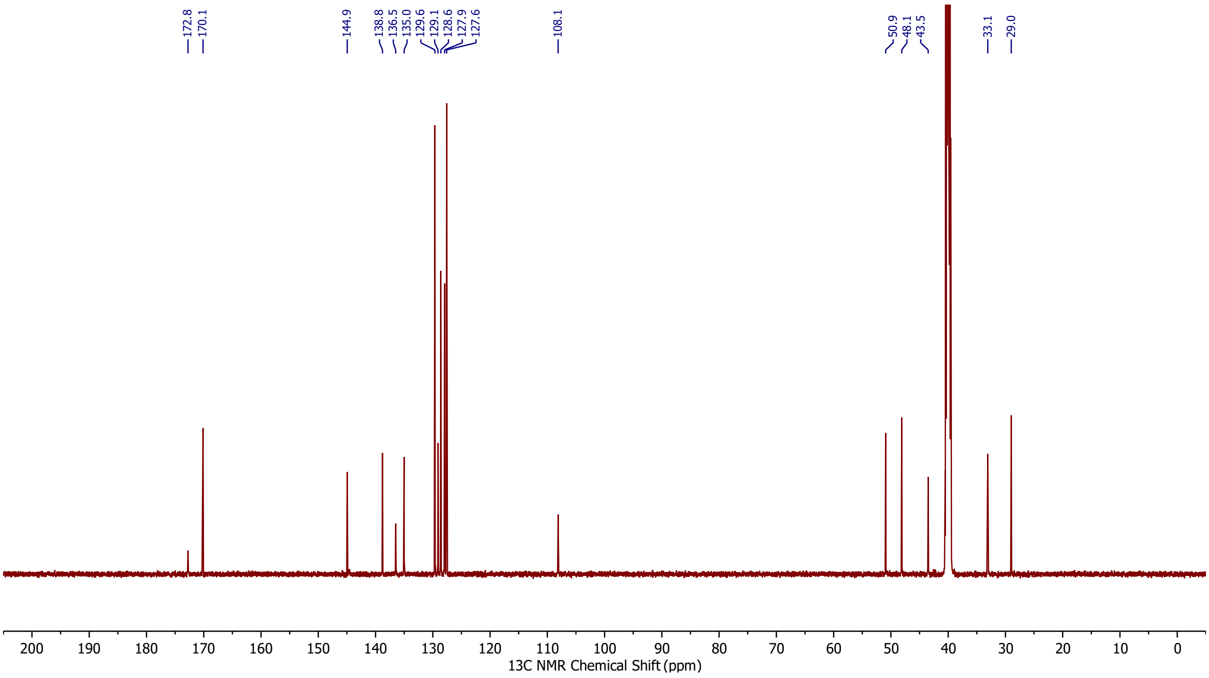

(±)-Ethyl 2-(2-(1-((4-phenoxyphenyl)sulfonyl)pyrrolidine-3-carboxamido)thiazol-4-yl)acetate (39a)

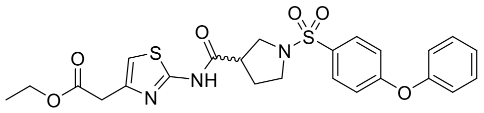

^{1}H NMR (600 MHz, 300 K, $CDCl_3$):

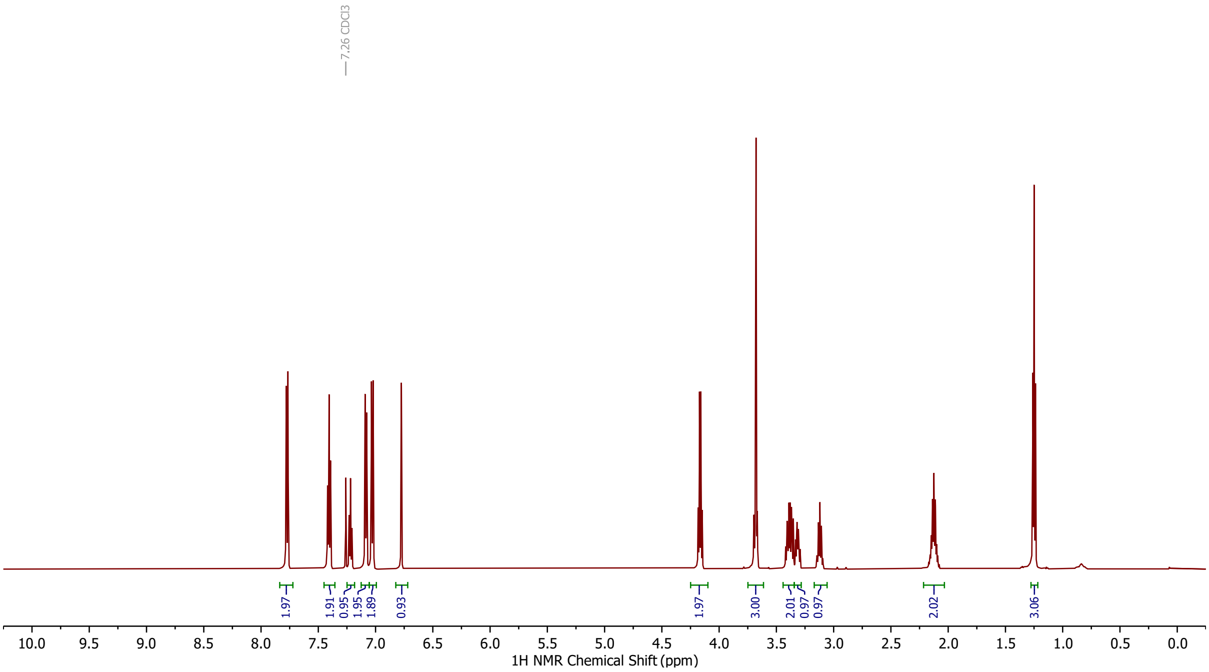

^{13}C NMR (151 MHz, 300 K, $CDCl_3$):

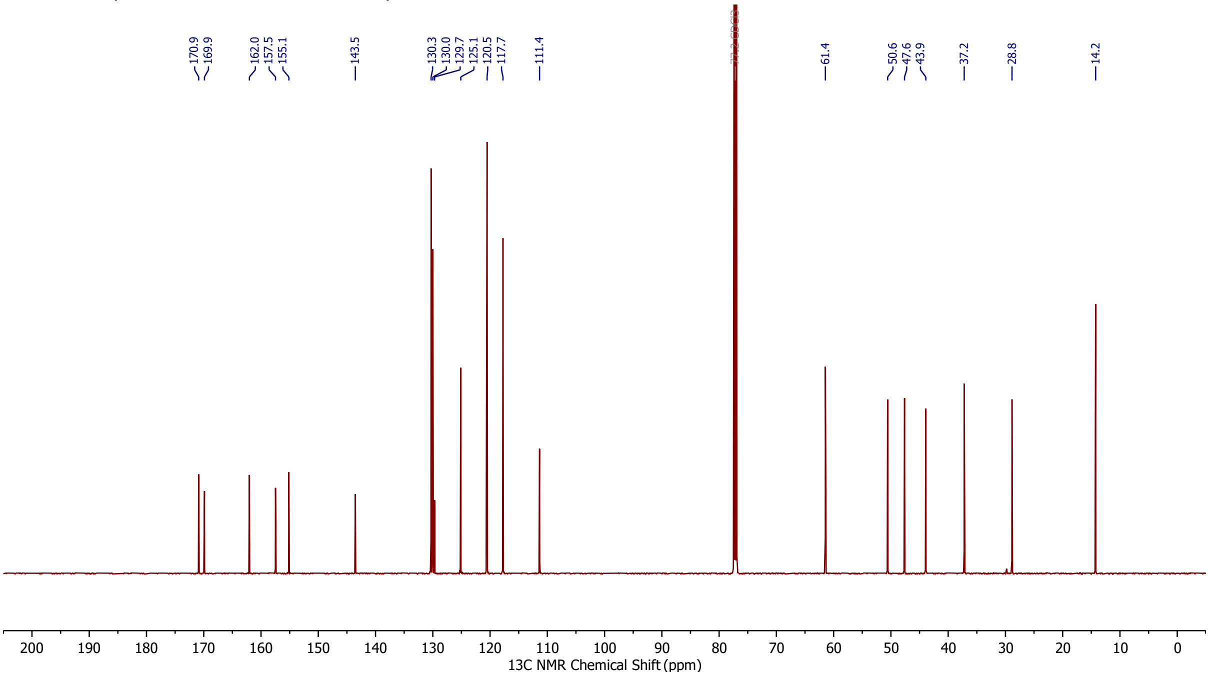

(±)-Ethyl (*Z*)-2-(3-hydroxy-2-((1-((4-phenoxyphenyl)sulfonyl)pyrrolidine-3-carbonyl)imino)-2,3-dihydrothiazol-4-yl)acetate (39b)

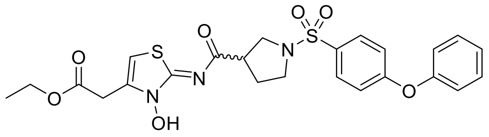

^{1}H NMR (600 MHz, 300 K, $CDCl_3$):

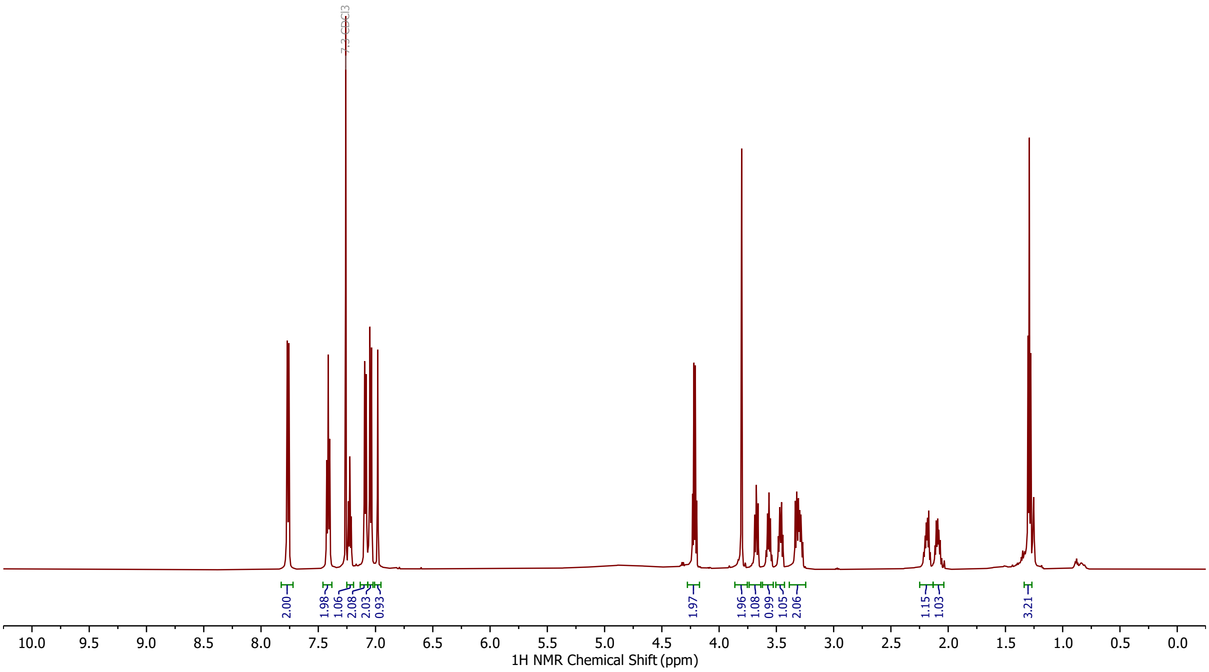

^{13}C NMR (151 MHz, 300 K, $CDCl_3$):

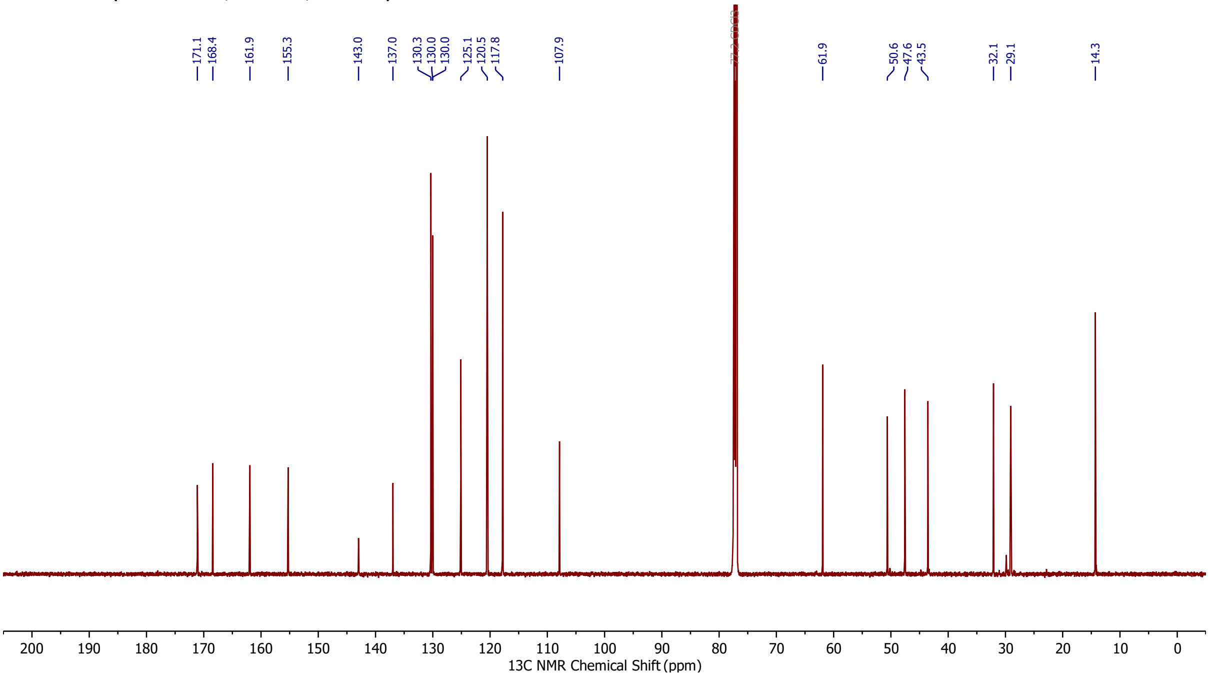

(±)-(*Z*)-2-(3-Hydroxy-2-((1-((4-phenoxyphenyl)sulfonyl)pyrrolidine-3-carbonyl)imino)-2,3-dihydrothiazol-4-yl)acetic acid (39)

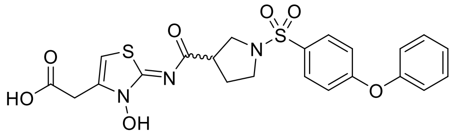

^{1}H NMR (600 MHz, 300 K, DMSO-d_6):

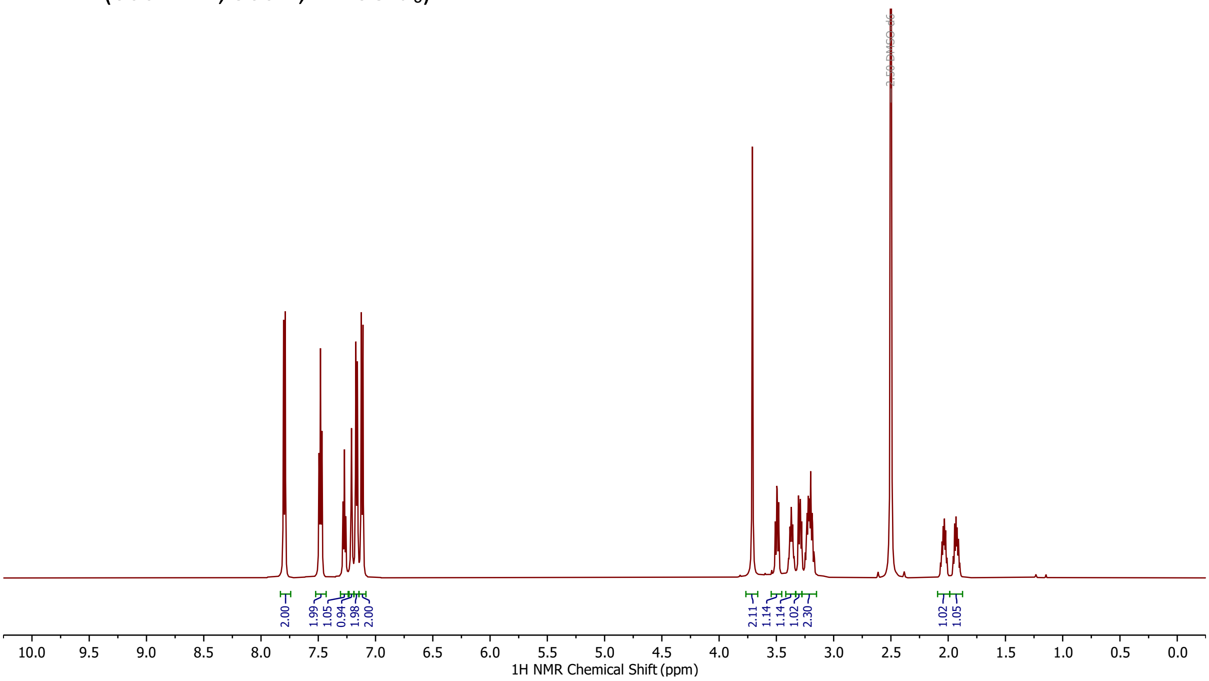

^{13}C NMR (151 MHz, 300 K, DMSO-d_6):

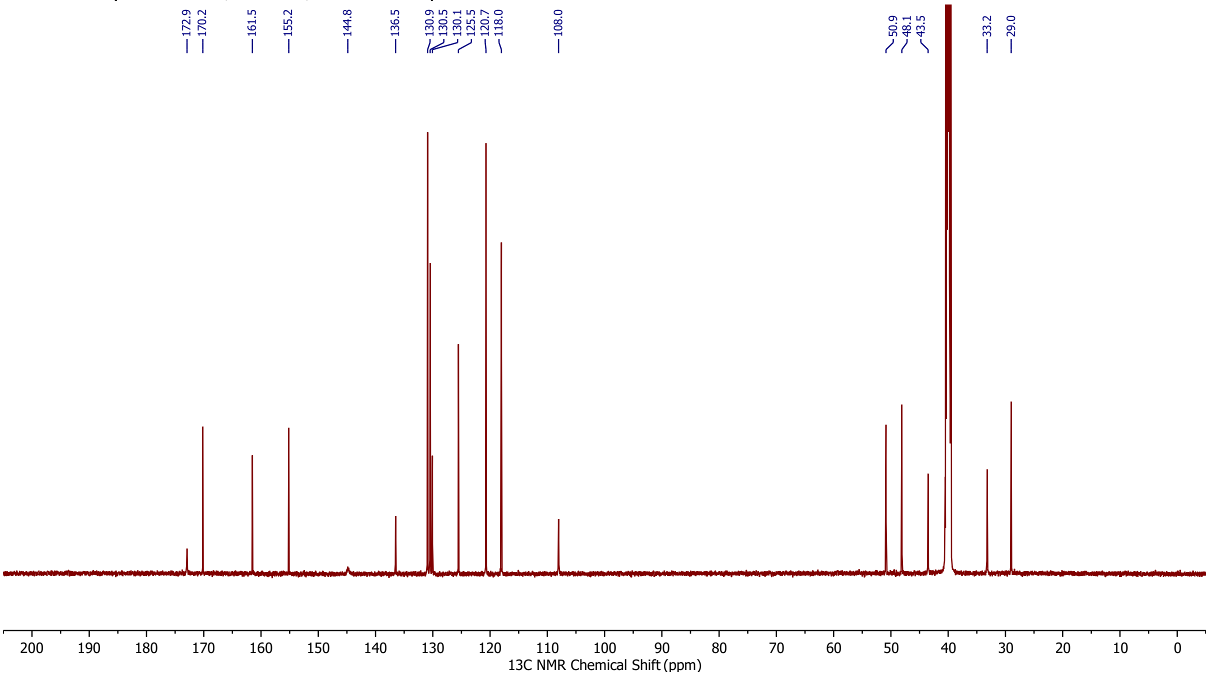

(±)-Ethyl 2-(2-(1-(naphthalen-2-ylsulfonyl)pyrrolidine-3-carboxamido)thiazol-4-yl)acetate (40a)

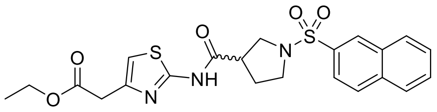

^{1}H NMR (600 MHz, 300 K, $CDCl_3$):

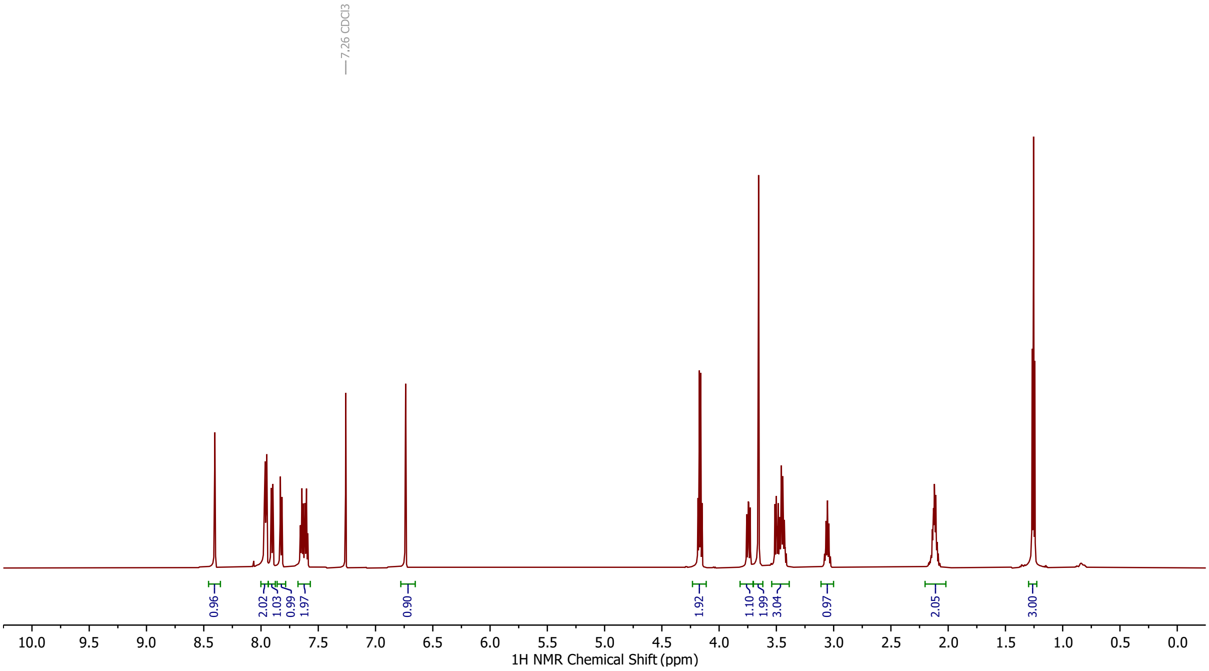

^{13}C NMR (151 MHz, 300 K, $CDCl_3$):

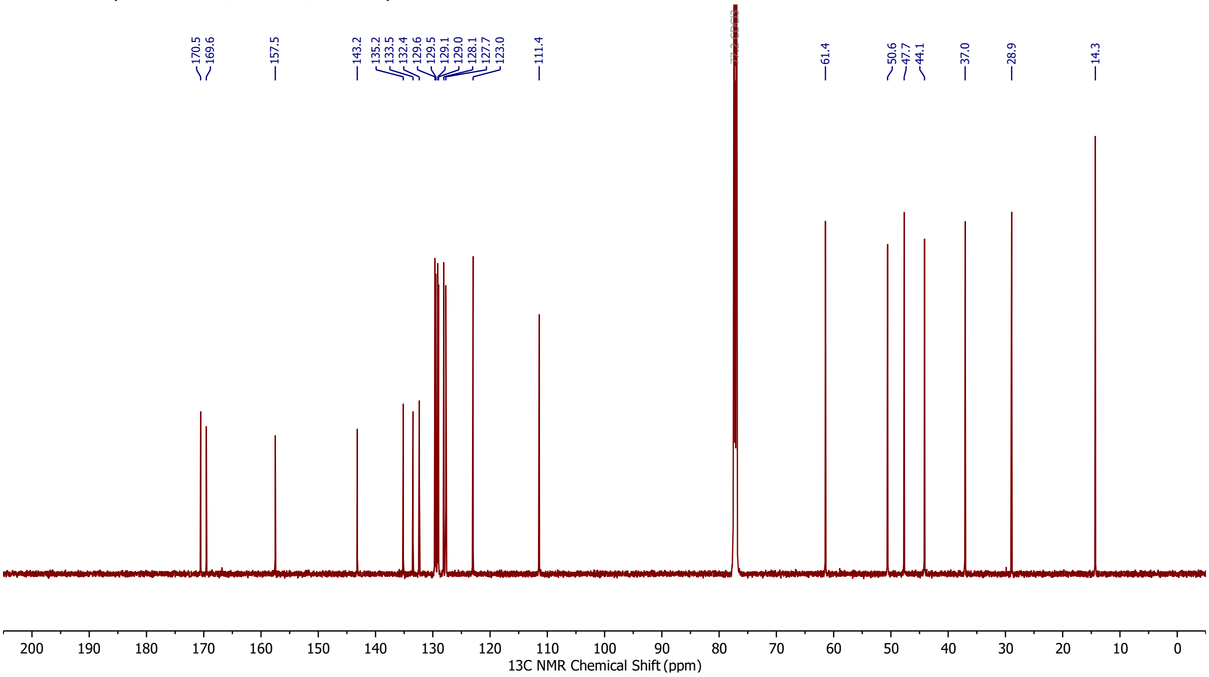

(±)-Ethyl (*Z*)-2-(3-hydroxy-2-((1-(naphthalen-2-ylsulfonyl)pyrrolidine-3-carbonyl)imino)-2,3-dihydrothiazol-4-yl)acetate (40b)

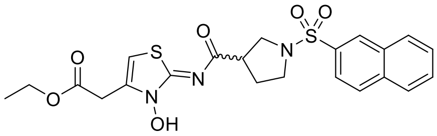

^{1}H NMR (600 MHz, 300 K, $CDCl_3$):

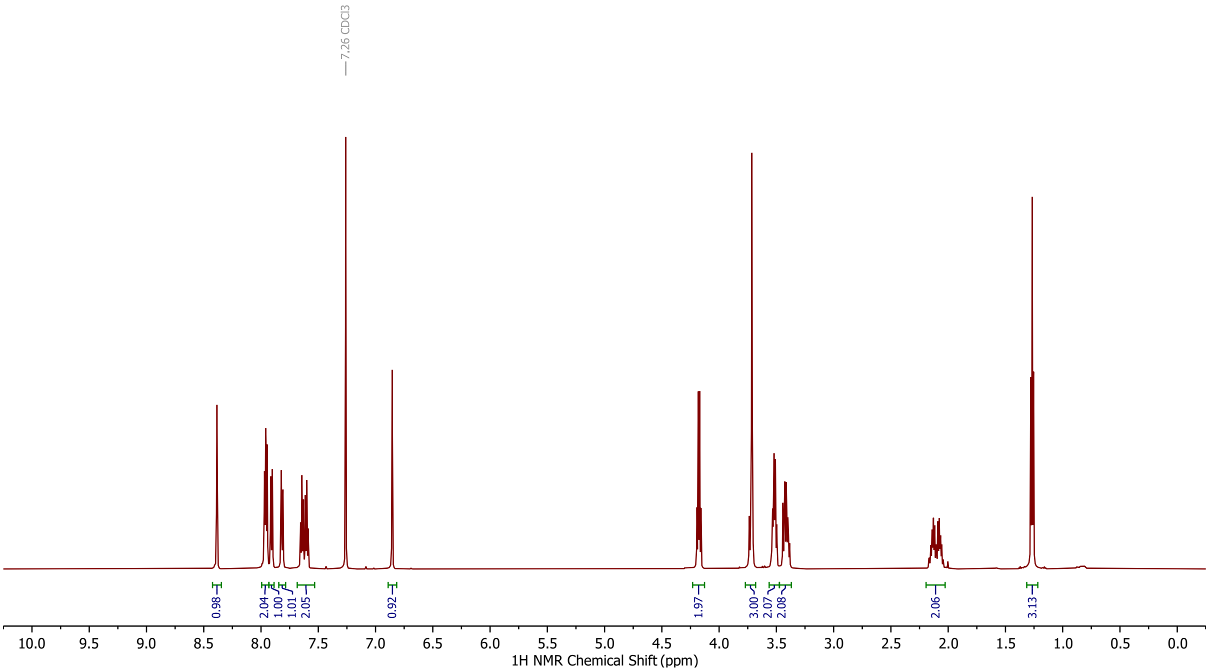

^{13}C NMR (151 MHz, 300 K, $CDCl_3$):

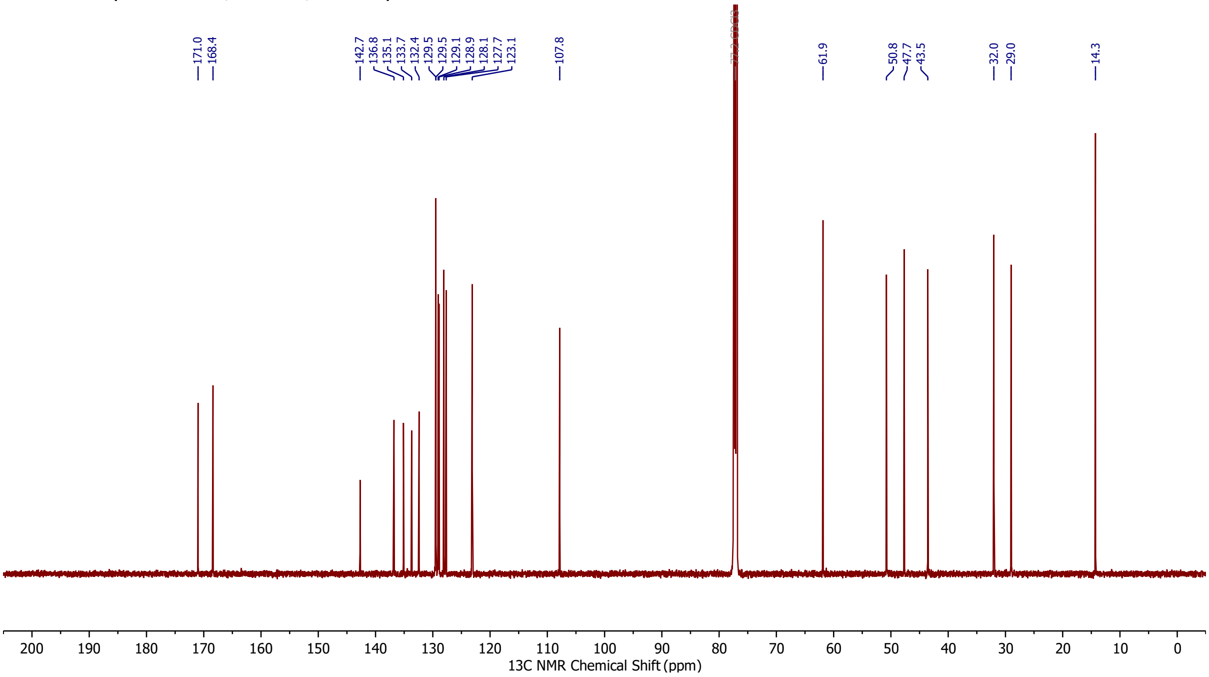

(±)-(*Z*)-2-(3-Hydroxy-2-((1-(naphthalen-2-ylsulfonyl)pyrrolidine-3-carbonyl)imino)-2,3-dihydrothiazol-4-yl)acetic acid (40)

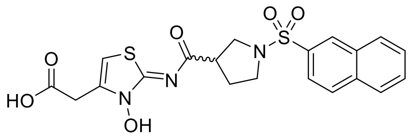

^{1}H NMR (600 MHz, 300 K, DMSO-d_6):

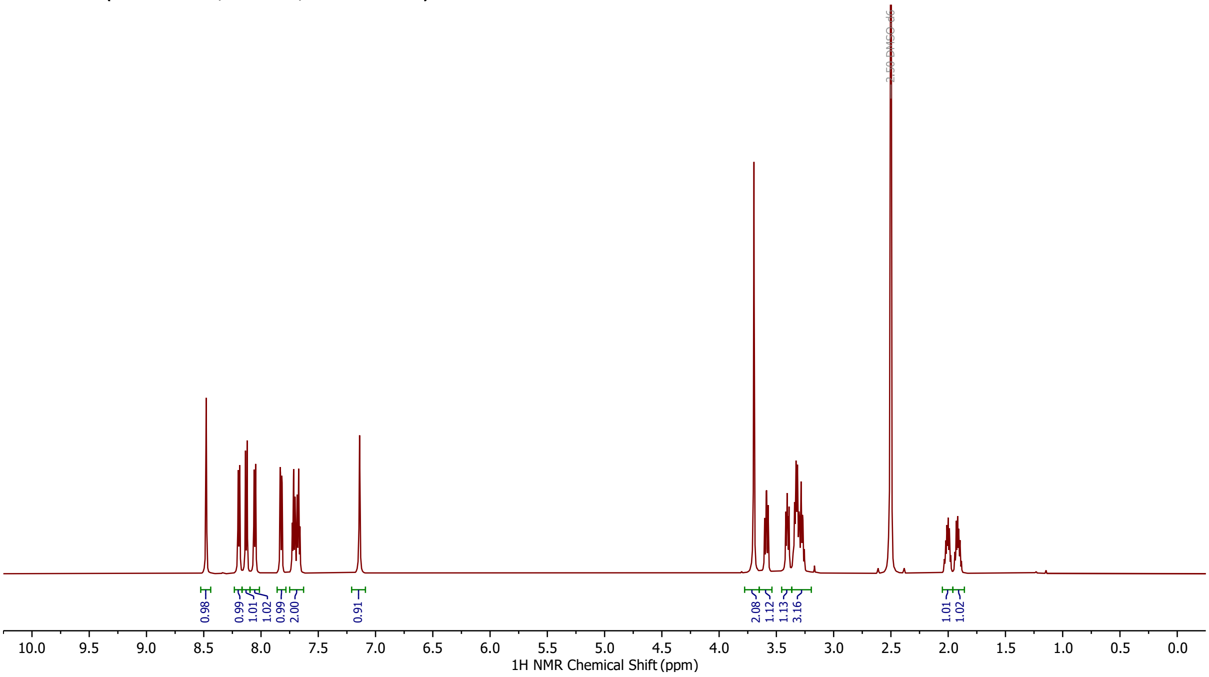

^{13}C NMR (151 MHz, 300 K, DMSO-d_6):

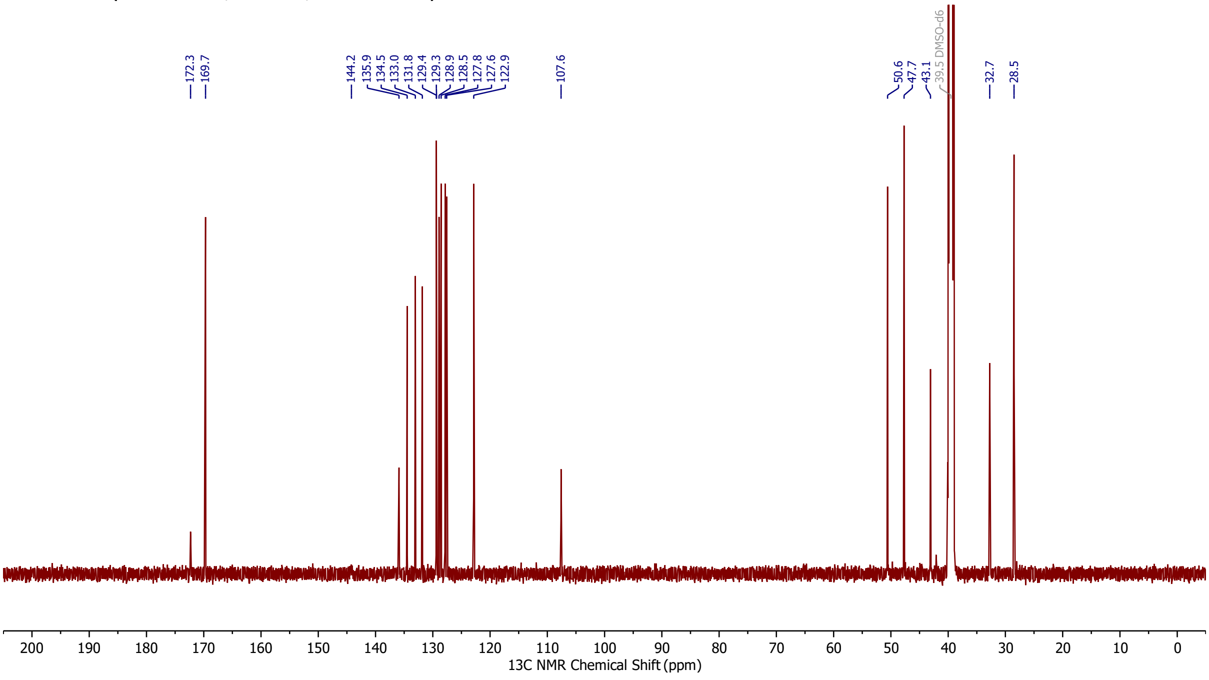

(±)-Ethyl 2-(2-(1-(thiophen-2-ylsulfonyl)pyrrolidine-3-carboxamido)thiazol-4-yl)acetate (41a)

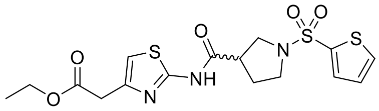

^{1}H NMR (600 MHz, 300 K, $CDCl_3$):

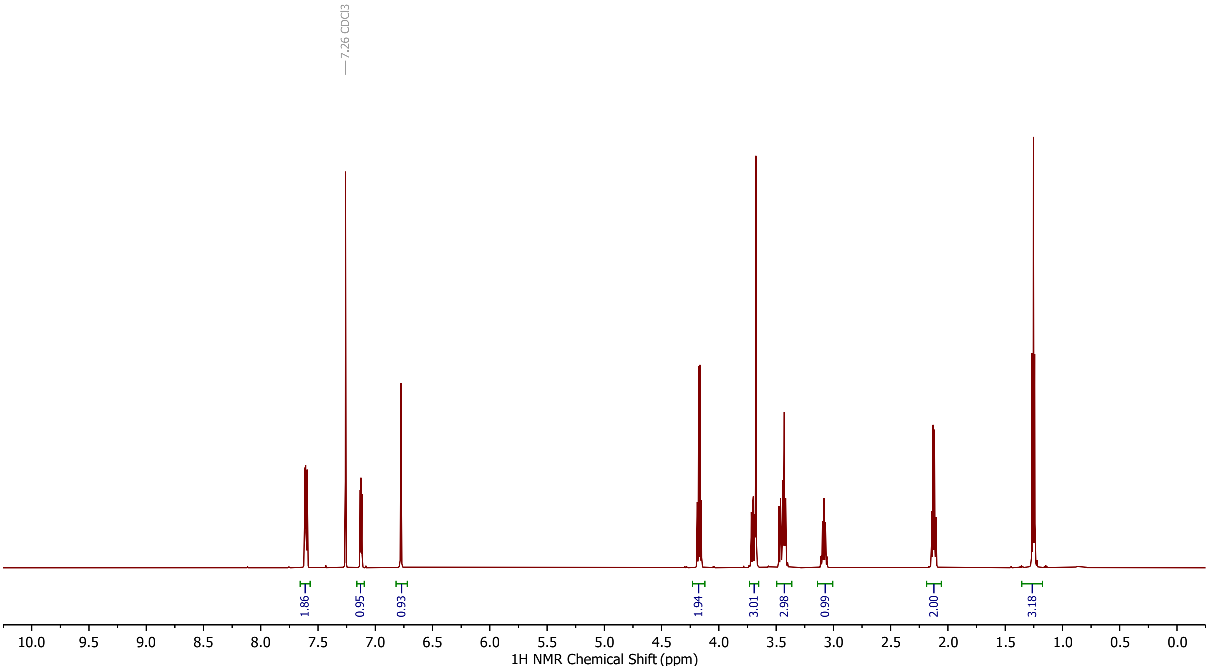

^{13}C NMR (151 MHz, 300 K, $CDCl_3$):

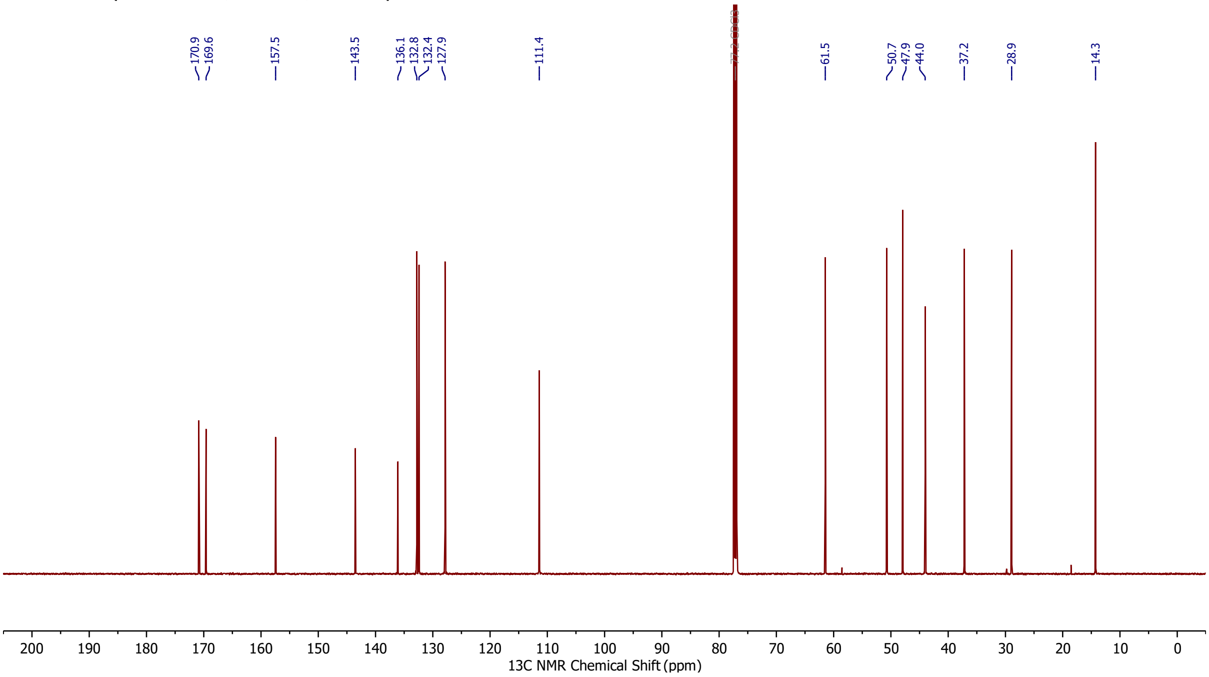

(±)-Ethyl (*Z*)-2-(3-hydroxy-2-((1-(thiophen-2-ylsulfonyl)pyrrolidine-3-carbonyl)imino)-2,3-dihydrothiazol-4-yl)acetate (41b)

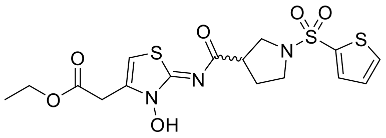

^{1}H NMR (600 MHz, 300 K, DMSO-d_6):

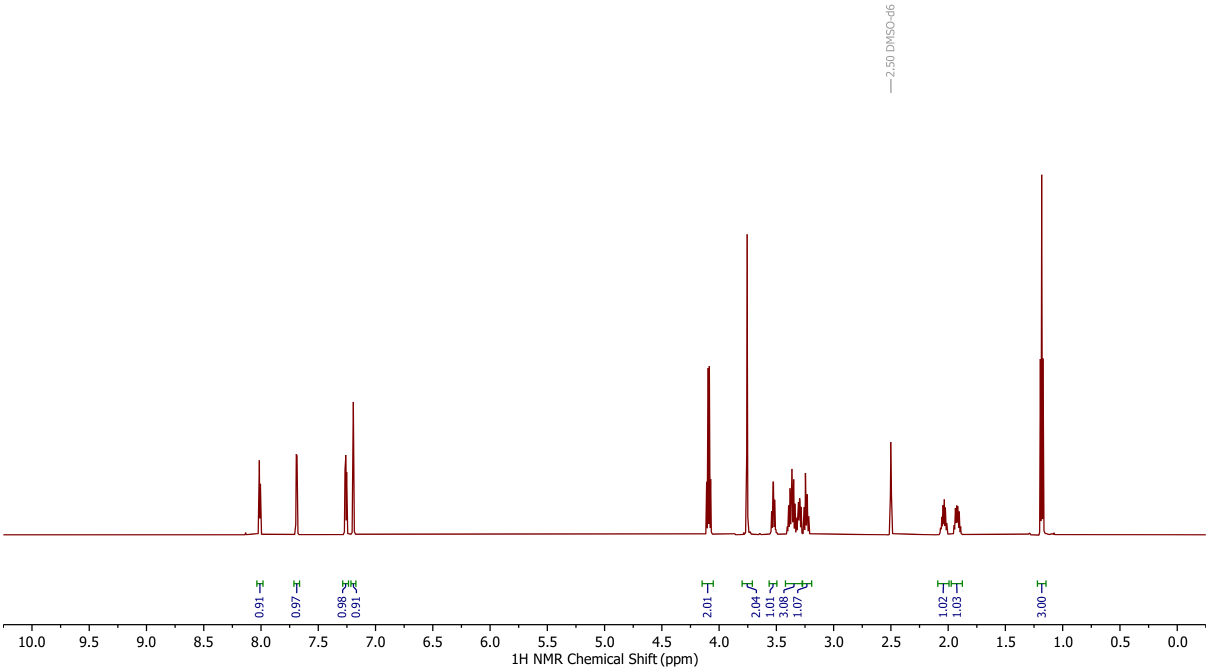

^{13}C NMR (151 MHz, 300 K, DMSO-d_6):

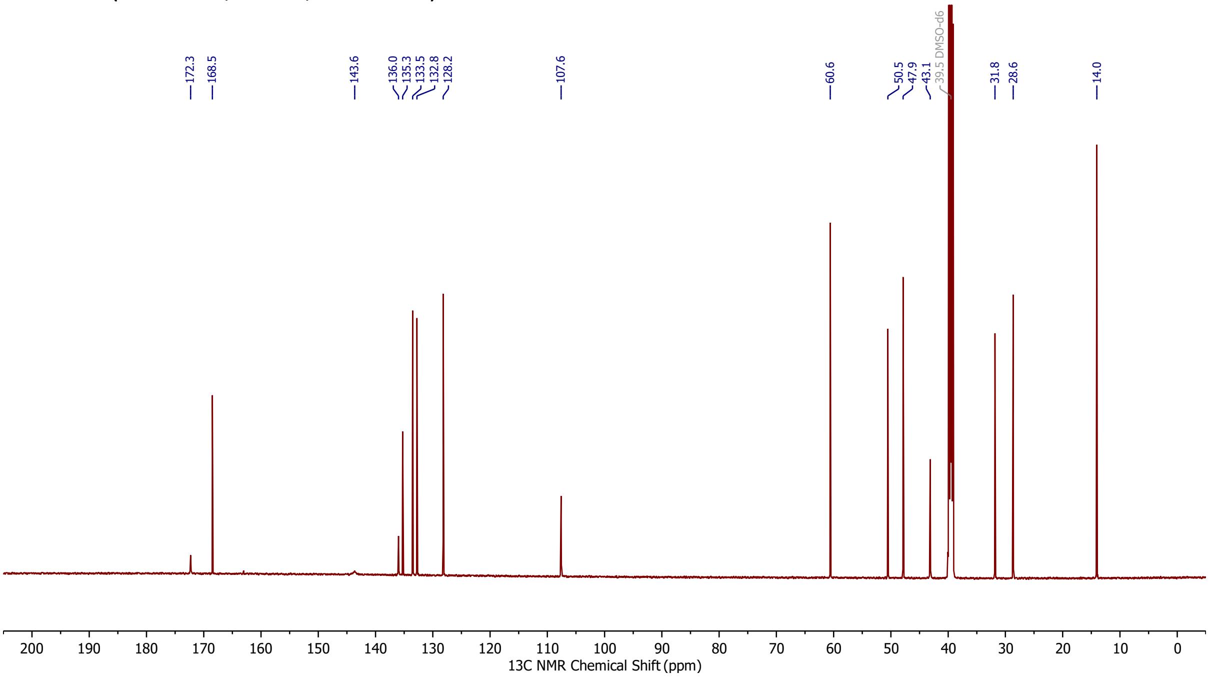

(±)-(*Z*)-2-(3-Hydroxy-2-((1-(thiophen-2-ylsulfonyl)pyrrolidine-3-carbonyl)imino)-2,3-dihydrothiazol-4-yl)acetic acid (41)

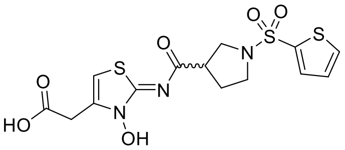

^{1}H NMR (600 MHz, 300 K, DMSO-d_6):

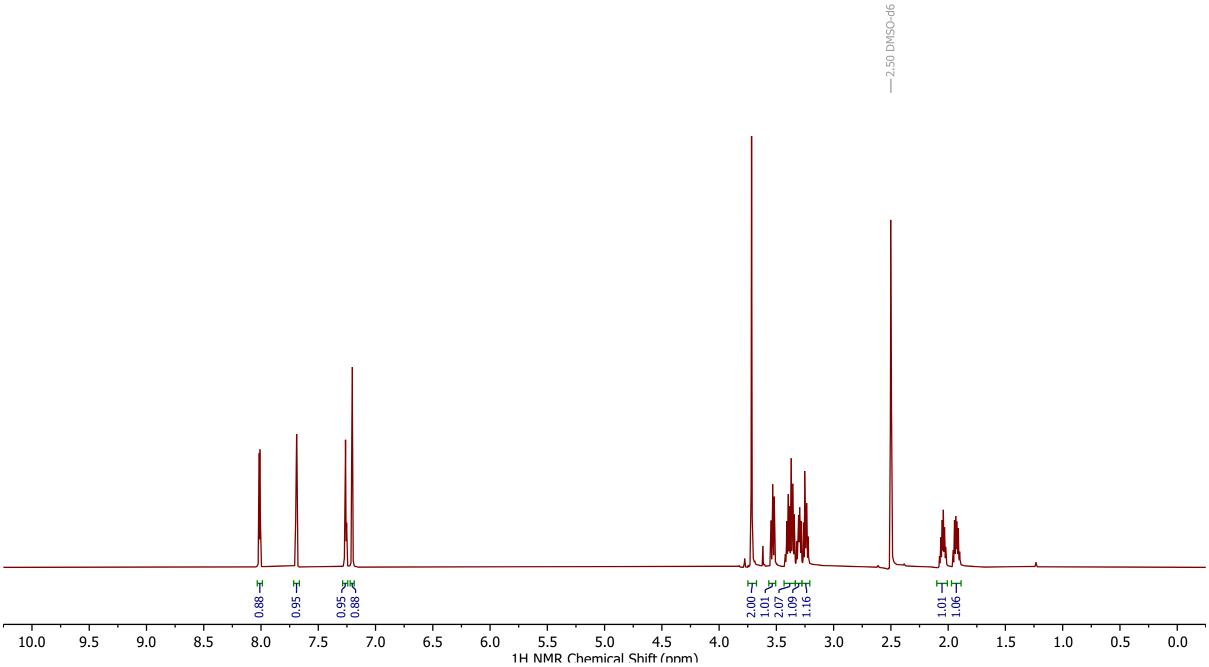

^{13}C NMR (151 MHz, 300 K, DMSO-d_6):

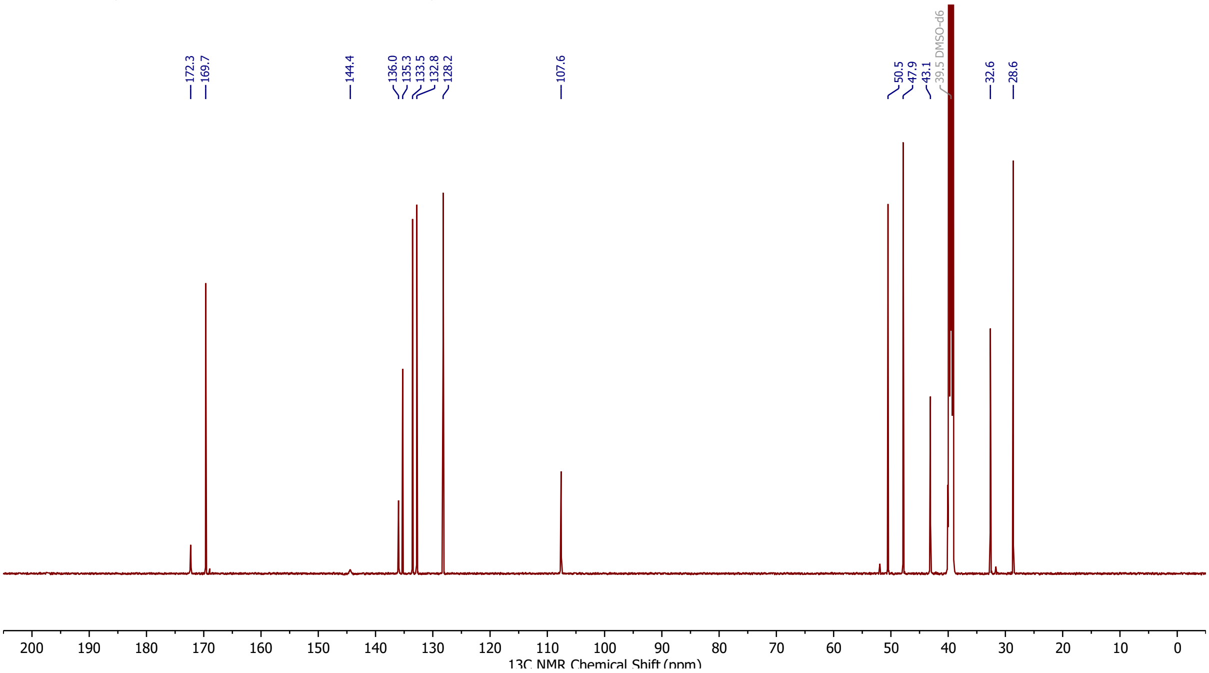

(±)-Ethyl 2-(2-(1-(benzylsulfonyl)pyrrolidine-3-carboxamido)thiazol-4-yl)acetate (42a)

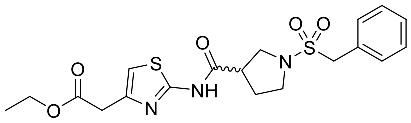

^{1}H NMR (600 MHz, 300 K, $CDCl_3$):

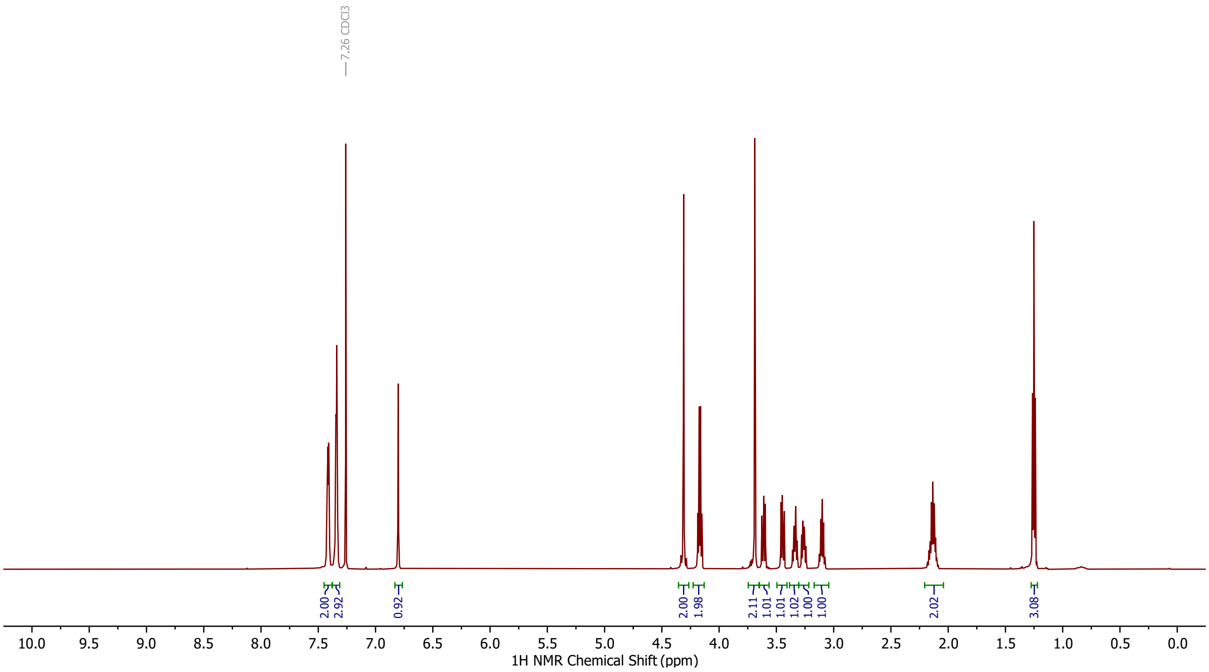

^{13}C NMR (151 MHz, 300 K, $CDCl_3$):

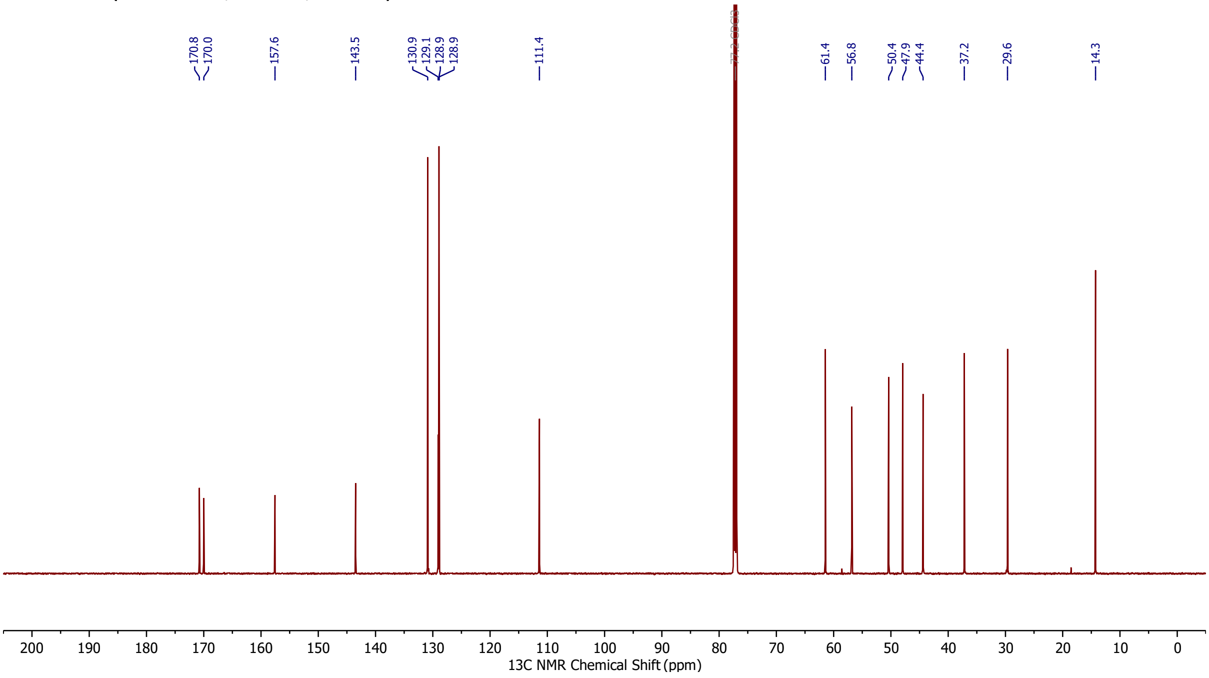

(±)-Ethyl (*Z*)-2-(2-((1-(benzylsulfonyl)pyrrolidine-3-carbonyl)imino)-3-hydroxy-2,3-dihydrothiazol-4-yl)acetate (42b)

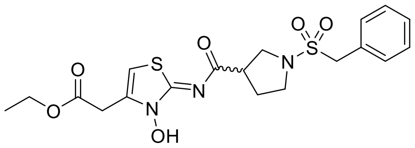

^{1}H NMR (600 MHz, 300 K, DMSO-d_6):

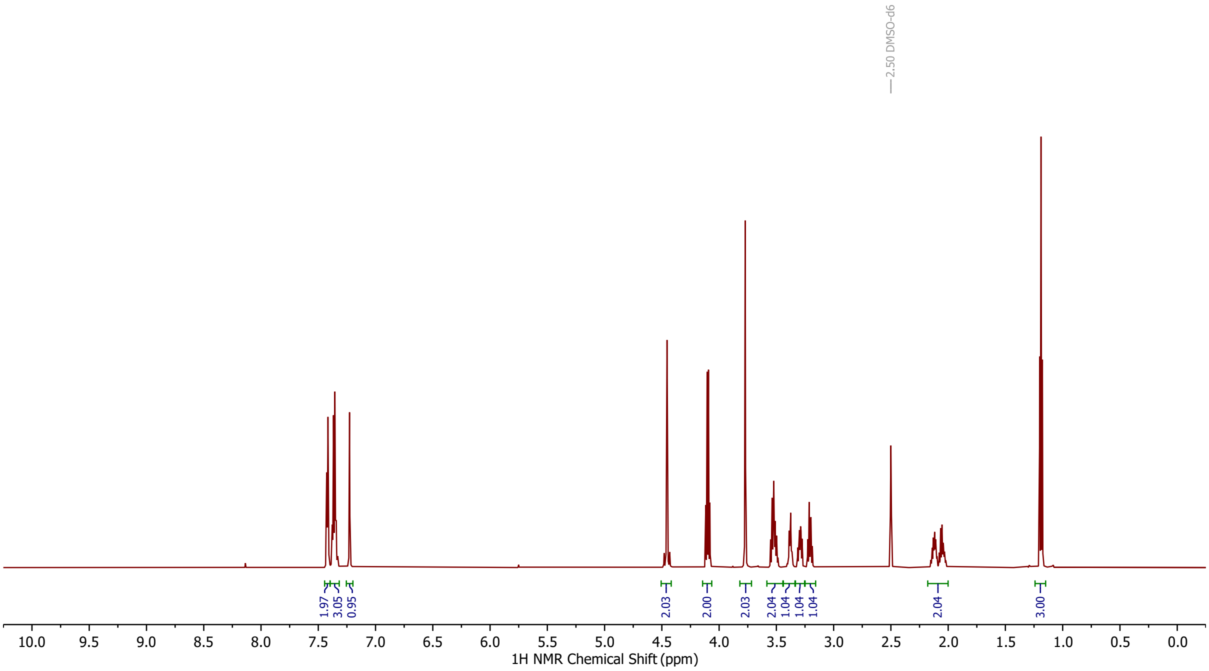

^{13}C NMR (151 MHz, 300 K, DMSO-d_6):

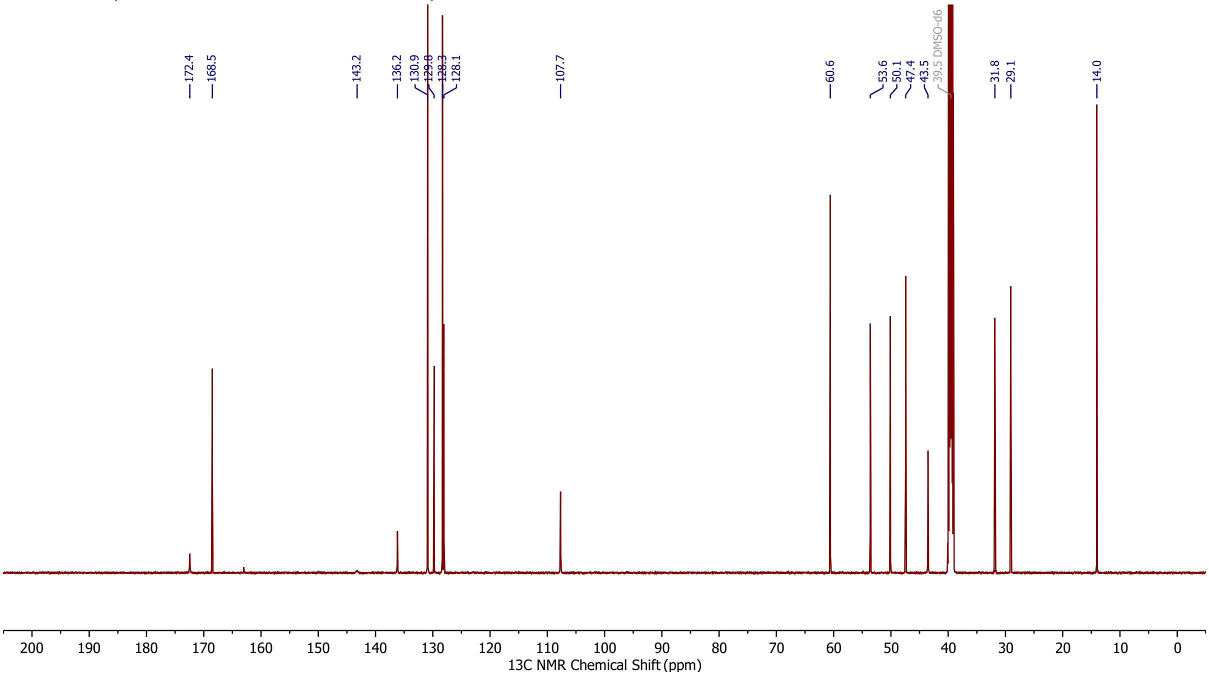

(±)-(*Z*)-2-(2-((1-(Benzylsulfonyl)pyrrolidine-3-carbonyl)imino)-3-hydroxy-2,3-dihydrothiazol-4-yl)acetic acid (42)

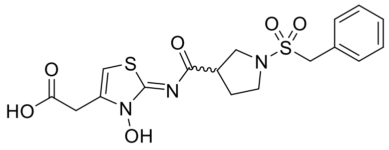

^{1}H NMR (600 MHz, 300 K, DMSO-d_6):

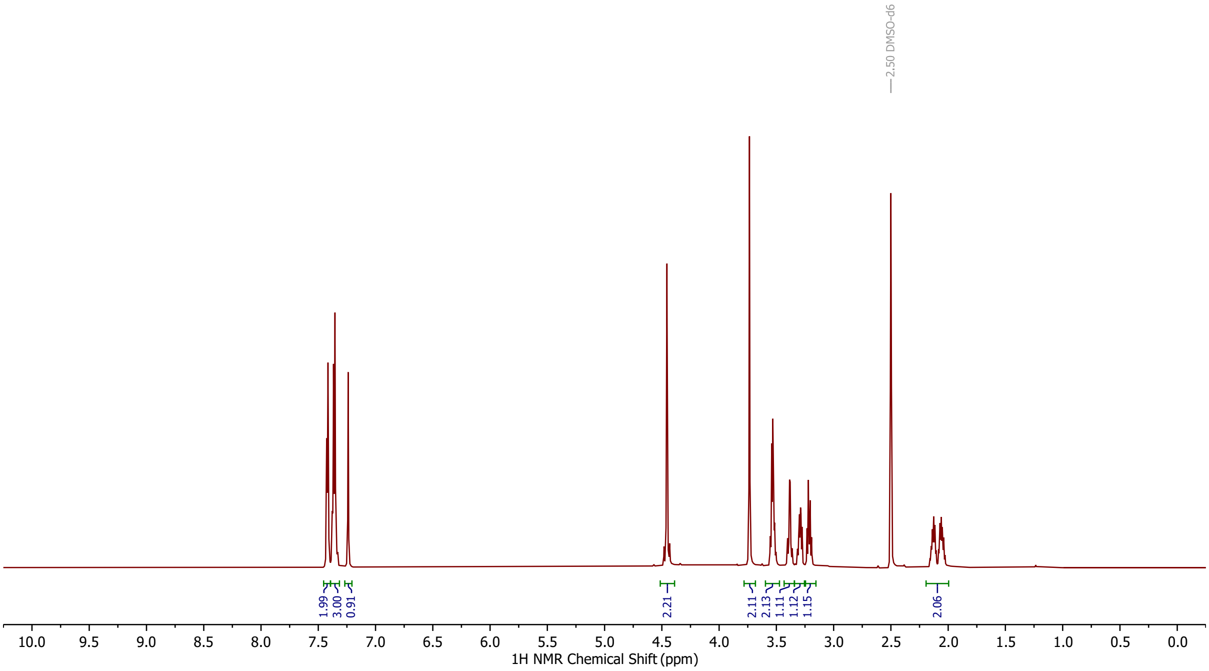

^{13}C NMR (151 MHz, 300 K, DMSO-d_6):

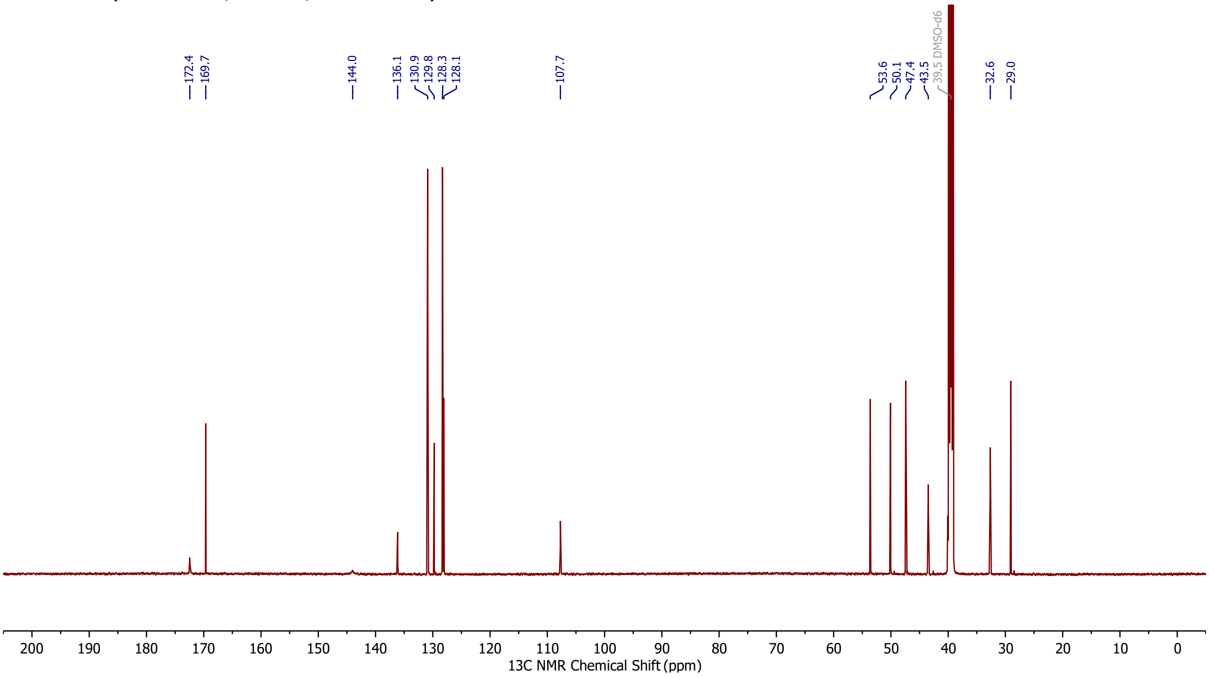

(±)-Ethyl 2-(2-(1-(cyclopentylsulfonyl)pyrrolidine-3-carboxamido)thiazol-4-yl)acetate (43a)

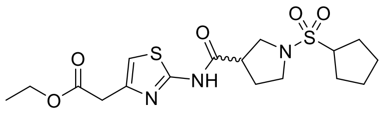

^{1}H NMR (600 MHz, 300 K, $CDCl_3$):

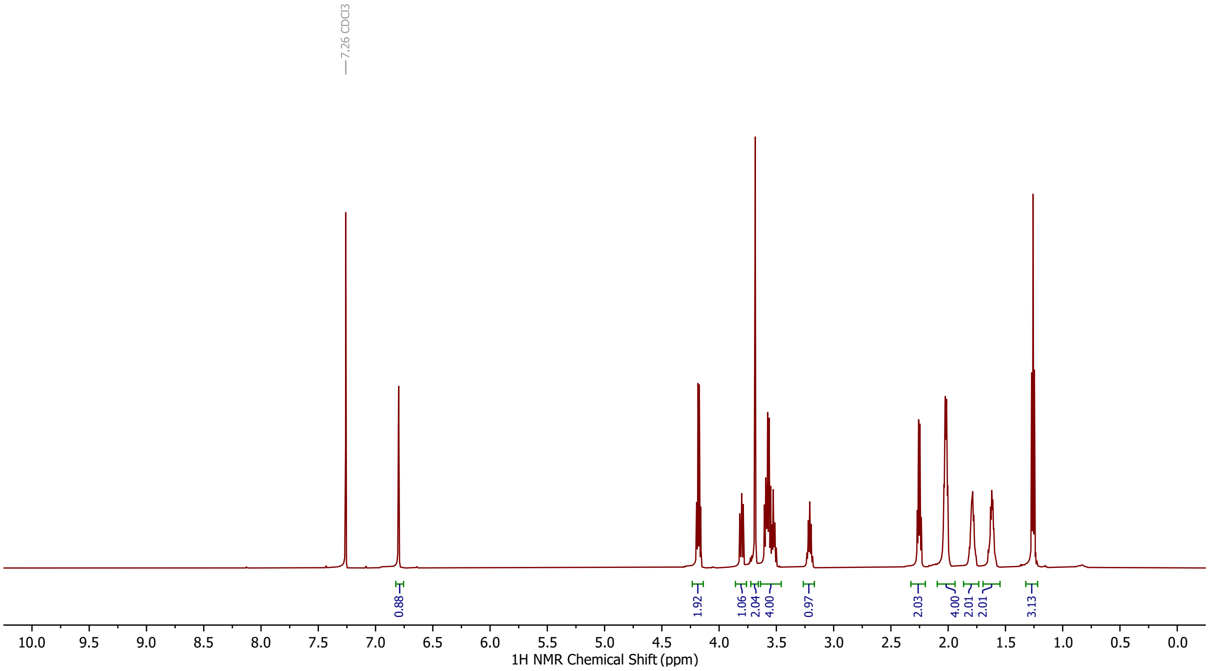

^{13}C NMR (151 MHz, 300 K, $CDCl_3$):

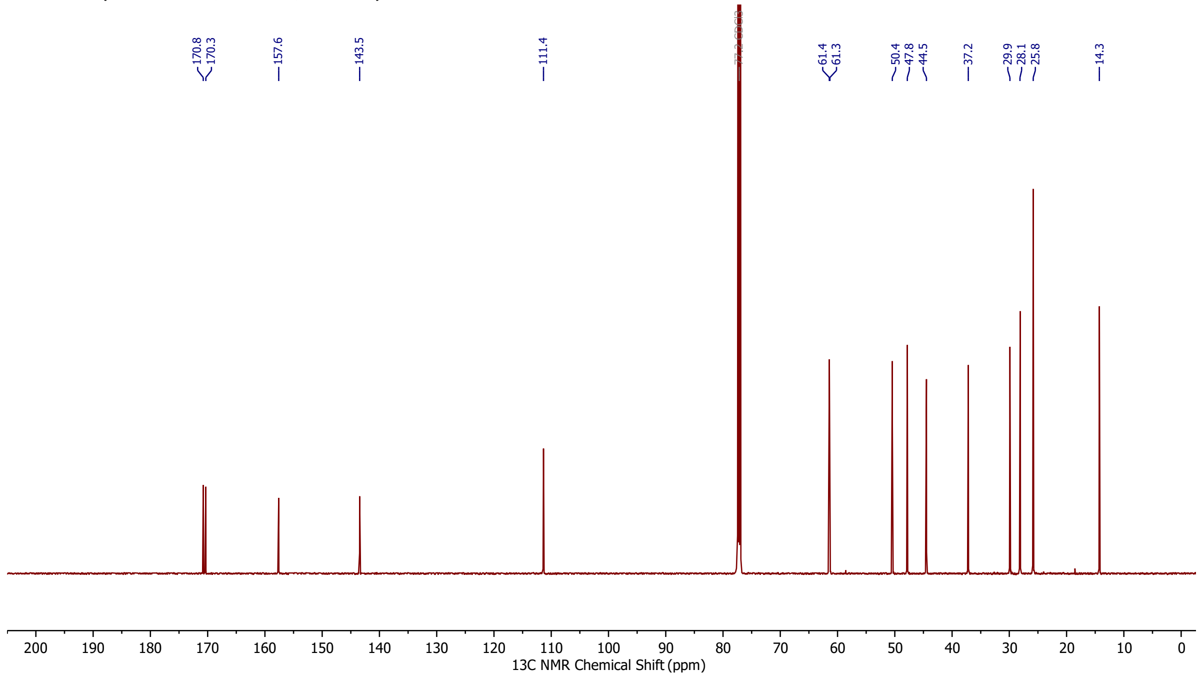

(±)-Ethyl (*Z*)-2-(2-((1-(cyclopentylsulfonyl)pyrrolidine-3-carbonyl)imino)-3-hydroxy-2,3-dihydrothiazol-4-yl)acetate (43b)

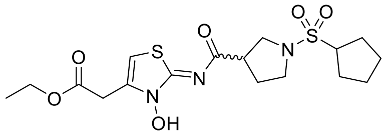

^{1}H NMR (600 MHz, 300 K, DMSO-d_6):

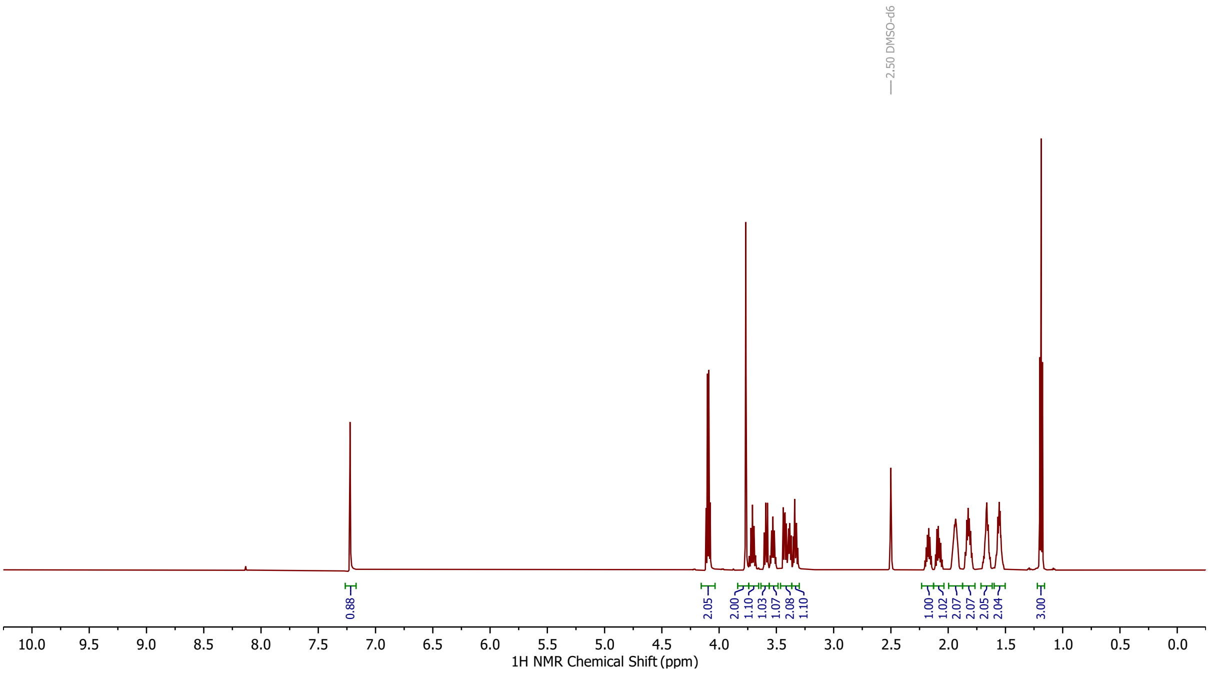

^{13}C NMR (151 MHz, 300 K, DMSO-d_6):

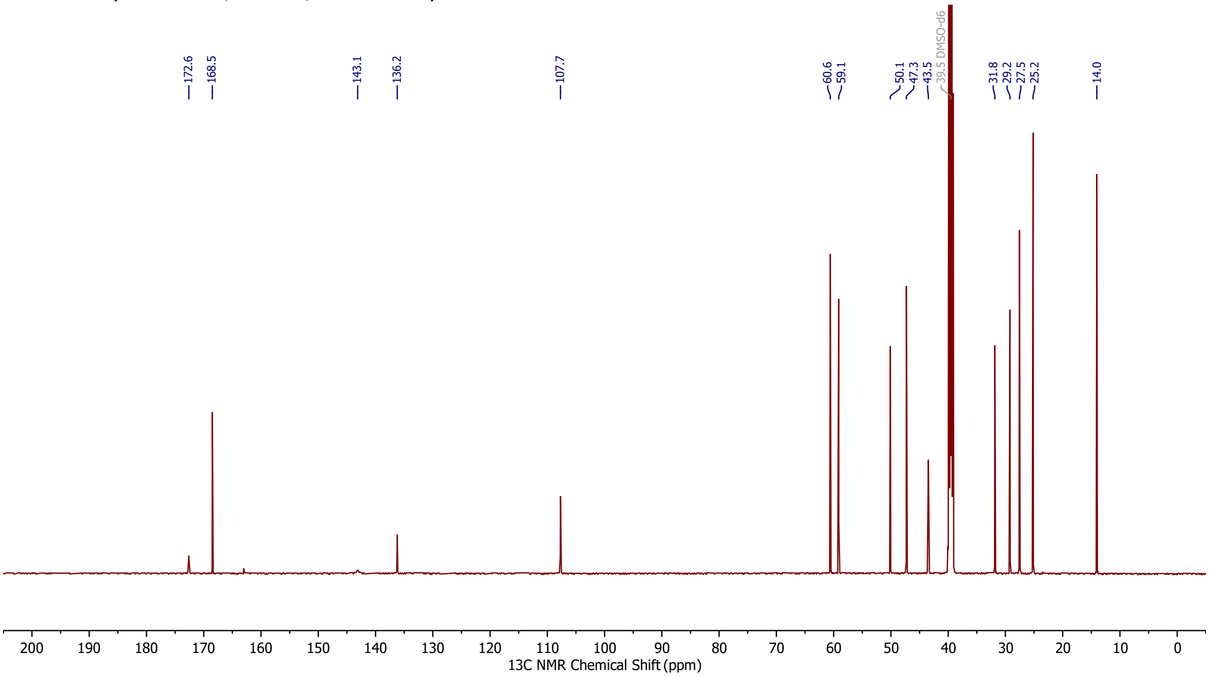

(±)-(*Z*)-2-(2-((1-(Cyclopentylsulfonyl)pyrrolidine-3-carbonyl)imino)-3-hydroxy-2,3-dihydrothiazol-4-yl)acetic acid (43)

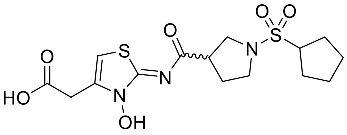

^{1}H NMR (600 MHz, 300 K, DMSO-d_6):

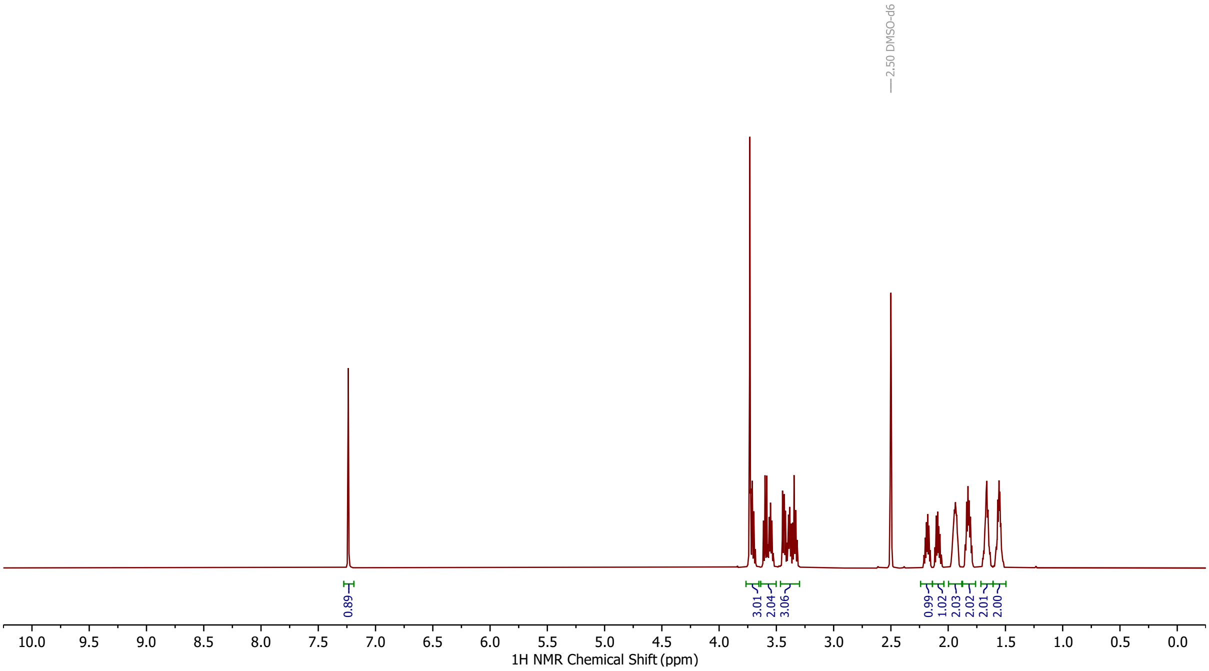

^{13}C NMR (151 MHz, 300 K, DMSO-d_6):

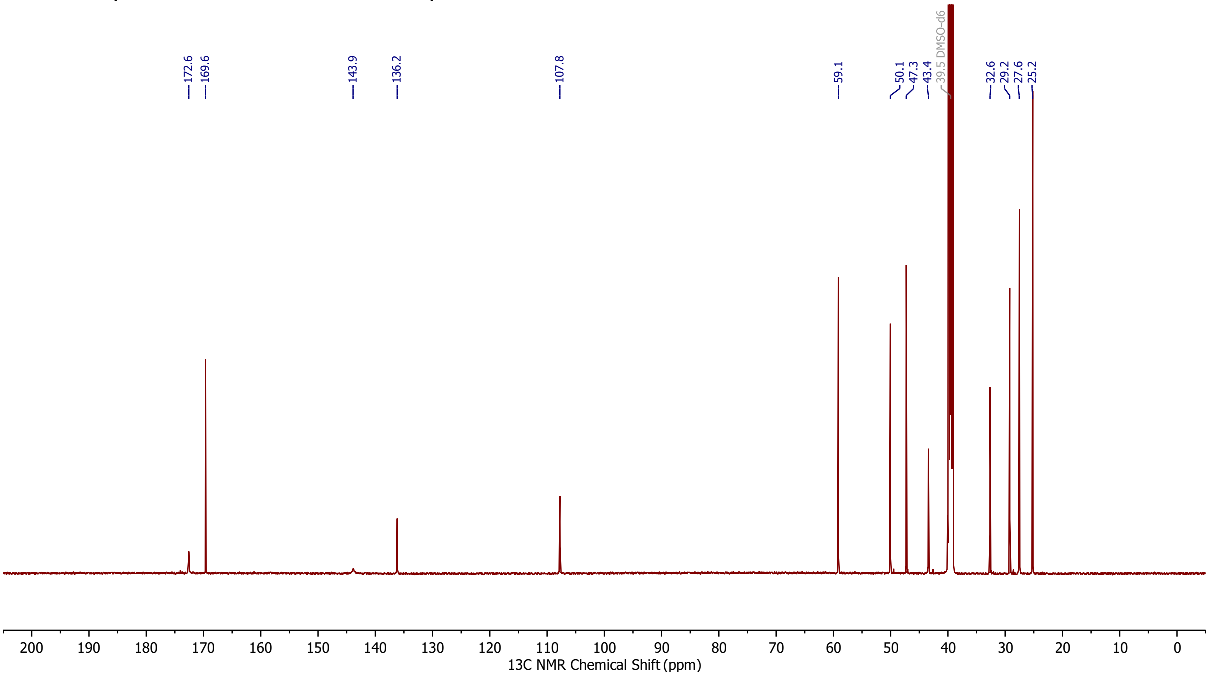

10. References

1. J. P. Holt-Martyn, R. Chowdhury, A. Tumber, T.-L. Yeh, M. I. Abboud, K. Lippl, C. T. Lohans, G. W. Langley, W. Figg Jr., M. A. McDonough, C. W. Pugh, P. J. Ratcliffe and C. J. Schofield, Structure-activity relationship and crystallographic studies on 4-hydroxypyrimidine HIF prolyl hydroxylase domain inhibitors, *ChemMedChem*, 2020, **15**, 270-273.

2. Y. Nakashima, L. Brewitz, A. Tumber, E. Salah and C. J. Schofield, 2-Oxoglutarate derivatives can selectively enhance or inhibit the activity of human oxygenases, *Nat. Commun.*, 2021, **12**, 6478.

3. C. M. Tegley, V. N. Viswanadhan, K. Biswas, M. J. Frohn, T. A. N. Peterkin, C. Chang, R. W. Bürli, J. H. Dao, H. Veith, N. Rogers, S. C. Yoder, G. Biddlecome, P. Tagari, J. R. Allen and R. W. Hungate, Discovery of novel hydroxy-thiazoles as HIF-α prolyl hydroxylase inhibitors: SAR, synthesis, and modeling evaluation, *Bioorg. Med. Chem. Lett.*, 2008, **18**, 3925-3928.

4. M. C. Chan, N. E. Ilott, J. Schödel, D. Sims, A. Tumber, K. Lippl, D. R. Mole, C. W. Pugh, P. J. Ratcliffe, C. P. Ponting and C. J. Schofield, Tuning the transcriptional response to hypoxia by inhibiting hypoxia-inducible factor (HIF) prolyl and asparaginyl hydroxylases, *J. Biol. Chem.*, 2016, **291**, 20661-20673.

5. Deven V. Parmar, Kevinkumar A. Kansagra, Jatin C. Patel, Shuchi N. Joshi, Nitin S. Sharma, Apeksha D. Shelat, Nirav B. Patel, Vishal B. Nakrani, Farheen A. Shaikh, Harilal V. Patel and ZYAN1 Trial Investigators, Outcomes of desidustat treatment in people with anemia and chronic kidney disease: a phase 2 study, *Am. J. Nephrol.*, 2019, **49**, 470-478.

6. S. Kato, N. Ochiai, H. Takano, F. Io, N. Takayama, H. Koretsune, E.-i. Kunioka, S. Uchida and K. Yamamoto, TP0463518, a novel prolyl hydroxylase inhibitor, specifically induces erythropoietin production in the liver, *J. Pharmacol. Exp. Ther.*, 2019, **371**, 675-683.

7. J. Zhou, J. Li, D. M. Rosenbaum, J. Zhuang, C. Poon, P. Qin, K. Rivera, J. Lepore, R. N. Willette, E. Hu and F. C. Barone, The prolyl 4-hydroxylase inhibitor GSK360A decreases post-stroke brain injury and sensory, motor, and cognitive behavioral deficits, *PLoS One*, 2017, **12**, e0184049.

8. T. D. Barrett, H. L. Palomino, T. I. Brondstetter, K. C. Kanelakis, X. Wu, P. V. Haug, W. Yan, A. Young, H. Hua, J. C. Hart, D.-T. Tran, H. Venkatesan, M. D. Rosen, H. M. Peltier, K. Sepassi, M. C. Rizzolio, S. D. Bembenek, T. Mirzadegan, M. H. Rabinowitz and N. P. Shankley, Pharmacological characterization of 1-(5-chloro-6-(trifluoromethoxy)-1*H*-benzoimidazol-2-yl)-1*H*-pyrazole-4-carboxylic acid (JNJ-42041935), a potent and selective hypoxia-inducible factor prolyl hydroxylase inhibitor, *Mol. Pharmacol.*, 2011, **79**, 910-920.

9. Y. Ogoshi, T. Matsui, I. Mitani, M. Yokota, M. Terashita, D. Motoda, K. Ueyama, T. Hotta, T. Ito, Y. Hase, K. Fukui, K. Deai, H. Yoshiuchi, S. Ito and H. Abe, Discovery of JTZ-951: a HIF prolyl hydroxylase inhibitor for the treatment of renal anemia, *ACS Med. Chem. Lett.*, 2017, **8**, 1320-1325.

10. J. S. Debenham, C. Madsen-Duggan, M. J. Clements, T. F. Walsh, J. T. Kuethe, M. Reibarkh, S. P. Salowe, L. M. Sonatore, R. Hajdu, J. A. Milligan, D. M. Visco, D. Zhou, R. B. Lingham, D. Stickens, J. A. DeMartino, X. Tong, M. Wolff, J. Pang, R. R. Miller, E. C. Sherer and J. J. Hale, Discovery of *N*-[Bis(4-methoxyphenyl)methyl]-4-hydroxy-2-(pyridazin-3-yl) pyrimidine-5-carboxamide (MK-8617), an orally active pan-inhibitor of Hypoxia-Inducible Factor Prolyl Hydroxylase 1–3 (HIF PHD1–3) for the treatment of anemia, *J. Med. Chem.*, 2016, **59**, 11039-11049.

11. M. A. McDonough, L. A. McNeill, M. Tilliet, C. A. Papamicaël, Q.-Y. Chen, B. Banerji, K. S. Hewitson and C. J. Schofield, Selective inhibition of factor inhibiting hypoxia-inducible factor, *J. Am. Chem. Soc.*, 2005, **127**, 7680-7681.

12. J.-H. Zhang, T. D. Y. Chung and K. R. Oldenburg, A simple statistical parameter for use in evaluation and validation of high throughput screening assays, *J. Biomol. Screen.*, 1999, **4**, 67-73.

13. C. Lee, S. J. Kim, D. G. Jeong, S. M. Lee and S. E. Ryu, Structure of human FIH-1 reveals a unique active site pocket and interaction sites for HIF-1 and von Hippel-Lindau, *J. Biol. Chem.*, 2003, **278**, 7558-7563.

14. C. E. Dann III, R. K. Bruick and J. Deisenhofer, Structure of factor-inhibiting hypoxia-inducible factor 1: an asparaginyl hydroxylase involved in the hypoxic response pathway, *Proc. Natl. Acad. Sci. U.S.A.*, 2002, **99**, 15351-15356.

15. J. M. Elkins, K. S. Hewitson, L. A. McNeill, J. F. Seibel, I. Schlemminger, C. W. Pugh, P. J. Ratcliffe and C. J. Schofield, Structure of factor-inhibiting hypoxia-inducible factor (HIF) reveals mechanism of oxidative modification of HIF-1α, *J. Biol. Chem.*, 2003, **278**, 1802-1806.

16. M. S. Islam, T. M. Leissing, R. Chowdhury, R. J. Hopkinson and C. J. Schofield, 2-Oxoglutarate-dependent oxygenases, *Annu. Rev. Biochem.*, 2018, **87**, 585-620.

17. W. Aik, M. A. McDonough, A. Thalhammer, R. Chowdhury and C. J. Schofield, Role of the jelly-roll fold in substrate binding by 2-oxoglutarate oxygenases, *Curr. Opin. Struct. Biol.*, 2012, **22**, 691-700.

18. P. E. Pergola, B. S. Spinowitz, C. S. Hartman, B. J. Maroni and V. H. Haase, Vadadustat, a novel oral HIF stabilizer, provides effective anemia treatment in nondialysis-dependent chronic kidney disease, *Kidney Int.*, 2016, **90**, 1115-1122.

19. T.-L. Yeh, Thomas M. Leissing, M. I. Abboud, C. C. Thinnes, O. Atasoylu, J. P. Holt-Martyn, D. Zhang, A. Tumber, K. Lippl, C. T. Lohans, I. K. H. Leung, H. Morcrette, I. J. Clifton, T. D. W. Claridge, A. Kawamura, E. Flashman, X. Lu, P. J. Ratcliffe, R. Chowdhury, C. W. Pugh and C. J. Schofield, Molecular and cellular mechanisms of HIF prolyl hydroxylase inhibitors in clinical trials, *Chem. Sci.*, 2017, **8**, 7651-7668.

20. B. Cascella, S. G. Lee, S. Singh, J. M. Jez and L. M. Mirica, The small molecule JIB-04 disrupts O_2 binding in the Fe-dependent histone demethylase KDM4A/JMJD2A, *Chem. Commun.*, 2017, **53**, 2174-2177.

21. G. Jones, P. Willett, R. C. Glen, A. R. Leach and R. Taylor, Development and validation of a genetic algorithm for flexible docking, *J. Mol. Biol.*, 1997, **267**, 727-748.

22. R. Chowdhury, J. I. Candela-Lena, M. C. Chan, D. J. Greenald, K. K. Yeoh, Y.-M. Tian, M. A. McDonough, A. Tumber, N. R. Rose, A. Conejo-Garcia, M. Demetriades, S. Mathavan, A. Kawamura, M. K. Lee, F. van Eeden, C. W. Pugh, P. J. Ratcliffe and C. J. Schofield, Selective small molecule probes for the hypoxia inducible factor (HIF) prolyl hydroxylases, *ACS Chem. Biol.*, 2013, **8**, 1488-1496.

23. R. Chowdhury, M. I. Abboud, T. E. McAllister, B. Banerji, B. Bhushan, J. L. Sorensen, A. Kawamura and C. J. Schofield, Use of cyclic peptides to induce crystallization: case study with prolyl hydroxylase domain 2, *Sci. Rep.*, 2020, **10**, 21964.

24. J. H. Dao, R. J. M. Kurzeja, J. M. Morachis, H. Veith, J. Lewis, V. Yu, C. M. Tegley and P. Tagari, Kinetic characterization and identification of a novel inhibitor of hypoxia-inducible factor prolyl hydroxylase 2 using a time-resolved fluorescence resonance energy transfer-based assay technology, *Anal. Biochem.*, 2009, **384**, 213-223.

25. R. Chowdhury, I. K. H. Leung, Y.-M. Tian, M. I. Abboud, W. Ge, C. Domene, F.-X. Cantrelle, I. Landrieu, A. P. Hardy, C. W. Pugh, P. J. Ratcliffe, T. D. W. Claridge and C. J. Schofield, Structural basis for oxygen degradation domain selectivity of the HIF prolyl hydroxylases, *Nat. Commun.*, 2016, **7**, 12673.

26. L. Brewitz, Y. Nakashima and C. J. Schofield, Synthesis of 2-oxoglutarate derivatives and their evaluation as cosubstrates and inhibitors of human aspartate/asparagine-β-hydroxylase, *Chem. Sci.*, 2021, **12**, 1327-1342.

27. P. A. Del Rizzo, S. Krishnan and R. C. Trievel, Crystal structure and functional analysis of JMJD5 indicate an alternate specificity and function, *Mol. Cell. Biol.*, 2012, **32**, 4044-4052.

28. S. E. Wilkins, M. S. Islam, J. M. Gannon, S. Markolovic, R. J. Hopkinson, W. Ge, C. J. Schofield and R. Chowdhury, JMJD5 is a human arginyl C-3 hydroxylase, *Nat. Commun.*, 2018, **9**, 1180.

29. M. A. McDonough, V. Li, E. Flashman, R. Chowdhury, C. Mohr, B. M. R. Liénard, J. Zondlo, N. J. Oldham, I. J. Clifton, J. Lewis, L. A. McNeill, R. J. M. Kurzeja, K. S. Hewitson, E. Yang, S. Jordan, R. S. Syed and C. J. Schofield, Cellular oxygen sensing: Crystal structure of hypoxia-inducible factor prolyl hydroxylase (PHD2), *Proc. Natl. Acad. Sci. U.S.A.*, 2006, **103**, 9814-9819.

30. A. Makena, S. S. van Berkel, C. Lejeune, R. J. Owens, A. Verma, R. Salimraj, J. Spencer, J. Brem and C. J. Schofield, Chromophore-linked substrate (CLS405): probing metallo-β-lactamase activity and inhibition, *ChemMedChem*, 2013, **8**, 1923-1929.

31. H. Wissmann and H.-J. Kleiner, New peptide synthesis, *Angew. Chem., Int. Ed.*, 1980, **19**, 133-134.

32. L. A. Carpino, 1-Hydroxy-7-azabenzotriazole. An efficient peptide coupling additive, *J. Am. Chem. Soc.*, 1993, **115**, 4397-4398.

33. G. M. Atkins Jr. and E. M. Burgess, The reactions of an *N*-sulfonylamine inner salt, *J. Am. Chem. Soc.*, 1968, **90**, 4744-4745.

34. T. D. Downes, S. P. Jones, H. F. Klein, M. C. Wheldon, M. Atobe, P. S. Bond, J. D. Firth, N. S. Chan, L. Waddelove, R. E. Hubbard, D. C. Blakemore, C. De Fusco, S. D. Roughley, L. R. Vidler, M. A. Whatton, A. J.-A. Woolford, G. L. Wrigley and P. O'Brien, Design and Synthesis of 56 Shape-Diverse 3D Fragments, *Chem. Eur. J.*, 2020, **26**, 8969-8975.

35. I. Pfeffer, L. Brewitz, T. Krojer, S. A. Jensen, G. T. Kochan, N. J. Kershaw, K. S. Hewitson, L. A. McNeill, H. Kramer, M. Münzel, R. J. Hopkinson, U. Oppermann, P. A. Handford, M. A. McDonough and C. J. Schofield, Aspartate/asparagine-β-hydroxylase crystal structures reveal an unexpected epidermal growth factor-like domain substrate disulfide pattern, *Nat. Commun.*, 2019, **10**, 4910.

36. A. Tumber, E. Salah, L. Brewitz, T. P. Corner and C. J. Schofield, Kinetic and inhibition studies on human Jumonji-C (JmjC) domain-containing protein 5, *RSC Chem. Biol.*, 2023, **4**, 399-413.

37. S. S. Ng, K. L. Kavanagh, M. A. McDonough, D. Butler, E. S. Pilka, B. M. R. Lienard, J. E. Bray, P. Savitsky, O. Gileadi, F. von Delft, N. R. Rose, J. Offer, J. C. Scheinost, T. Borowski, M. Sundstrom, C. J. Schofield and U. Oppermann, Crystal structures of histone demethylase JMJD2A reveal basis for substrate specificity, *Nature*, 2007, **448**, 87-91.

38. L. Brewitz, A. Tumber, I. Pfeffer, M. A. McDonough and C. J. Schofield, Aspartate/asparagine-β-hydroxylase: a high-throughput mass spectrometric assay for discovery of small molecule inhibitors, *Sci. Rep.*, 2020, **10**, 8650.

39. S. E. Hutchinson, M. V. Leveridge, M. L. Heathcote, P. Francis, L. Williams, M. Gee, J. Munoz-Muriedas, B. Leavens, A. Shillings, E. Jones, P. Homes, S. Baddeley, C.-w. Chung, A. Bridges and

A. Argyrou, Enabling lead discovery for histone lysine demethylases by high-throughput RapidFire mass spectrometry, *J. Biomol. Screen.*, 2012, **17**, 39-48.

40. G. Winter, xia2: an expert system for macromolecular crystallography data reduction, *J. Appl. Crystallogr.*, 2010, **43**, 186-190.

41. A. J. McCoy, R. W. Grosse-Kunstleve, P. D. Adams, M. D. Winn, L. C. Storoni and R. J. Read, Phaser crystallographic software, *J. Appl. Crystallogr.*, 2007, **40**, 658-674.

42. D. Liebschner, P. V. Afonine, M. L. Baker, G. Bunkoczi, V. B. Chen, T. I. Croll, B. Hintze, L.-W. Hung, S. Jain, A. J. McCoy, N. W. Moriarty, R. D. Oeffner, B. K. Poon, M. G. Prisant, R. J. Read, J. S. Richardson, D. C. Richardson, M. D. Sammito, O. V. Sobolev, D. H. Stockwell, T. C. Terwilliger, A. G. Urzhumtsev, L. L. Videau, C. J. Williams and P. D. Adams, Macromolecular structure determination using X-rays, neutrons and electrons: recent developments in Phenix, *Acta Crystallogr. D*, 2019, **75**, 861-877.

43. M. Yang, A. P. Hardy, R. Chowdhury, N. D. Loik, J. S. Scotti, J. S. O. McCullagh, T. D. W. Claridge, M. A. McDonough, W. Ge and C. J. Schofield, Substrate selectivity analyses of Factor Inhibiting Hypoxia-Inducible Factor, *Angew. Chem.*, 2013, **125**, 1744-1748.

44. P. Emsley and K. Cowtan, Coot: model-building tools for molecular graphics, *Acta Crystallogr. D*, 2004, **60**, 2126-2132.

45. PyMOL, The PyMOL Molecular Graphics System, Version 2.0, *Schrödinger. LLC*, 2017.

46. D. Liebschner, P. V. Afonine, N. W. Moriarty, B. K. Poon, O. V. Sobolev, T. C. Terwilliger and P. D. Adams, Polder maps: improving OMIT maps by excluding bulk solvent, *Acta Crystallogr. D*, 2017, **73**, 148-157.

47. K. Zebisch, V. Voigt, M. Wabitsch and M. Brandsch, Protocol for effective differentiation of 3T3-L1 cells to adipocytes, *Anal. Biochem.*, 2012, **425**, 88-90.

48. M. Ishiyama, H. Tominaga, M. Shiga, K. Sasamoto, Y. Ohkura and K. Ueno, A combined assay of cell viability and in vitro cytotoxicity with a highly water-soluble tetrazolium salt, neutral red and crystal violet, *Biol. Pharm. Bull.*, 1996, **19**, 1518-1520.

49. H. M. Berman, J. Westbrook, Z. Feng, G. Gilliland, T. N. Bhat, H. Weissig, I. N. Shindyalov and P. E. Bourne, The protein data bank, *Nucleic Acids Res.*, 2000, **28**, 235-242.

50. J. M. Word, S. C. Lovell, J. S. Richardson and D. C. Richardson, Asparagine and glutamine: using hydrogen atom contacts in the choice of side-chain amide orientation, *J. Mol. Biol.*, 1999, **285**, 1735-1747.

51. V. B. Chen, W. B. Arendall III, J. J. Headd, D. A. Keedy, R. M. Immormino, G. J. Kapral, L. W. Murray, J. S. Richardson and D. C. Richardson, MolProbity: all-atom structure validation for macromolecular crystallography, *Acta Crystallogr. D*, 2010, **66**, 12-21.

52. C. R. Søndergaard, M. H. Olsson, M. Rostkowski and J. H. Jensen, Improved treatment of ligands and coupling effects in empirical calculation and rationalization of pKa values, *J. Chem. Theory Comput.*, 2011, **7**, 2284-2295.

53. L. Brewitz, A. Tumber, A. Thalhammer, E. Salah, K. E. Christensen and C. J. Schofield, Synthesis of novel pyridine-carboxylates as small-molecule inhibitors of human Aspartate/Asparagine-β-Hydroxylase, *ChemMedChem*, 2020, **15**, 1139-1149.

54. K. Wu, K. Zhou, Y. Wang, Y. Zhou, N. Tian, Y. Wu, D. Chen, D. Zhang, X. Wang, H. Xu and X. Zhang, Stabilization of HIF-1α by FG-4592 promotes functional recovery and neural protection in experimental spinal cord injury, *Brain Res.*, 2016, **1632**, 19-26.

55. J. L. Ariazi, K. J. Duffy, D. F. Adams, D. M. Fitch, L. Luo, M. Pappalardi, M. Biju, E. H. DiFilippo, T. Shaw, K. Wiggall and C. Erickson-Miller, Discovery and preclinical characterization of GSK1278863 (daprodustat), a small molecule hypoxia inducible factor–prolyl hydroxylase inhibitor for anemia, *J. Pharmacol. Exp. Ther.*, 2017, **363**, 336-347.

56. H. Beck, M. Jeske, K. Thede, F. Stoll, I. Flamme, M. Akbaba, J.-K. Ergüden, G. Karig, J. Keldenich, F. Oehme, H.-C. Militzer, I. V. Hartung and U. Thuss, Discovery of molidustat (BAY 85-3934): a small-molecule oral HIF-prolyl hydroxylase (HIF-PH) inhibitor for the treatment of renal anemia, *ChemMedChem*, 2018, **13**, 988-1003.

57. M. C. Chan, O. Atasoylu, E. Hodson, A. Tumber, I. K. H. Leung, R. Chowdhury, V. Gómez-Pérez, M. Demetriades, A. M. Rydzik, J. Holt-Martyn, Y.-M. Tian, T. Bishop, T. D. W. Claridge, A. Kawamura, C. W. Pugh, P. J. Ratcliffe and C. J. Schofield, Potent and selective triazole-based inhibitors of the hypoxia-inducible factor prolyl-hydroxylases with activity in the murine brain, *PLoS One*, 2015, **10**, e0132004.

58. M. V. R. Reddy, M. R. Mallireddigari, V. R. Pallela, S. C. Cosenza, V. K. Billa, B. Akula, D. R. C. V. Subbaiah, E. V. Bharathi, A. Padgaonkar, H. Lv, J. M. Gallo and E. P. Reddy, Design, synthesis, and biological evaluation of (*E*)-*N*-aryl-2-arylethenesulfonamide analogues as potent and orally bioavailable microtubule-targeted anticancer agents, *J. Med. Chem.*, 2013, **56**, 5562-5586.

59. K. Vong, T. Yamamoto, T.-c. Chang and K. Tanaka, Bioorthogonal release of anticancer drugs via gold-triggered 2-alkynylbenzamide cyclization, *Chem. Sci.*, 2020, **11**, 10928-10933.

60. C. R. Moyes, R. Berger, S. D. Goble, B. Harper, D.-M. Shen, L. Wang, A. Bansal, P. N. Brown, A. S. Chen, K. H. Dingley, J. Di Salvo, A. Fitzmaurice, L. N. Gichuru, A. L. Hurley, N. Jochnowitz, R. R. Miller, S. Mistry, H. Nagabukuro, G. M. Salituro, A. Sanfiz, A. S. Stevenson, K. Villa, B. Zamlynny, M. Struthers, A. E. Weber and S. D. Edmondson, Design, synthesis, and evaluation of conformationally restricted acetanilides as potent and selective β3 adrenergic receptor agonists for the treatment of overactive bladder, *J. Med. Chem.*, 2014, **57**, 1437-1453.

61. S. Ferorelli, C. Abate, M. P. Pedone, N. A. Colabufo, M. Contino, R. Perrone and F. Berardi, Synthesis and binding assays of novel 3,3-dimethylpiperidine derivatives with various lipophilicities as σ_1 receptor ligands, *Biorg. Med. Chem.*, 2011, **19**, 7612-7622.

62. C. Bolchi, M. Pallavicini, S. K. Bernini, G. Chiodini, A. Corsini, N. Ferri, L. Fumagalli, V. Straniero and E. Valoti, Thiazole-and imidazole-containing peptidomimetic inhibitors of protein farnesyltransferase, *Bioorg. Med. Chem. Lett.*, 2011, **21**, 5408-5412.

63. Z. Iqbal, Z. Ashraf, M. Abas, M. N. Tahir, E. Jabeen, R. Z. Paracha, M. Nisar and S. Ahmad, Synthesis, crystal structure and DNA binding interactions of ethyl 2-(2-acetamidothiazol-4-yl) acetate: Theoretical and experimental investigations, *J. Mol. Struct.*, 2019, **1198**, 126903.

64. Y. Liu, L. Zhang, J. Gong, H. Fang, A. Liu, G. Du and W. Xu, Design, synthesis, and biological activity of thiazole derivatives as novel influenza neuraminidase inhibitors, *J. Enzyme Inhib. Med. Chem.*, 2011, **26**, 506-513.

65. P. R. Verhoest, C. Proulx-Lafrance, M. Corman, L. Chenard, C. J. Helal, X. Hou, R. Kleiman, S. Liu, E. Marr, F. S. Menniti, C. J. Schmidt, M. Vanase-Frawley, A. W. Schmidt, R. D. Williams, F. R. Nelson, K. R. Fonseca and S. Liras, Identification of a brain penetrant PDE9A inhibitor utilizing prospective design and chemical enablement as a rapid lead optimization strategy, *J. Med. Chem.*, 2009, **52**, 7946-7949.
